# Ruthenium-Catalyzed Monoselective C–H Methylation and *d*_3_-Methylation of Arenes

**DOI:** 10.1021/jacsau.2c00399

**Published:** 2022-10-21

**Authors:** Ashley Hogg, Matthew Wheatley, Pablo Domingo-Legarda, Asier Carral-Menoyo, Naomi Cottam, Igor Larrosa

**Affiliations:** Department of Chemistry, School of Natural Sciences, University of Manchester, Oxford Road, Manchester M13 9PL, United Kingdom

**Keywords:** catalysis, ruthenium, methylation, C−H activation, late-stage functionalization, ammonium salts

## Abstract

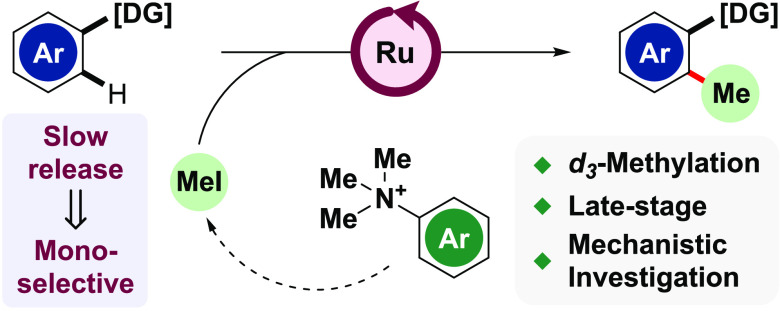

Site-selective installation of C–Me bonds remains a powerful and sought-after tool to alter the chemical and pharmacological properties of a molecule. Direct C–H functionalization provides an attractive means of achieving this transformation. Such protocols, however, typically utilize harsh conditions and hazardous methylating agents with poor applicability toward late-stage functionalization. Furthermore, highly monoselective methylation protocols remain scarce. Herein, we report an efficient monoselective, directed *ortho*-methylation of arenes using *N,N,N*-trimethylanilinium salts as noncarcinogenic, bench-stable methylating agents. We extend this protocol to *d*_3_-methylation in addition to the late-stage functionalization of pharmaceutically active compounds. Detailed kinetic studies indicate the rate-limiting in situ formation of MeI is integral to the observed reactivity.

## 1 Introduction

The direct methylation of C–H bonds is a transformation of significant interest to the organic chemistry community and has been the subject of intense research efforts over the last 30 years.^1^ This interest stems, in part, from the “magic methyl effect”, a phenomenon where the installation of a single methyl group can drastically affect the biological properties of pharmaceutical molecules. For example, the introduction of two methyl groups in the *N*^3^-phenyl group of *N*^3^,*N*^6^-diaryl-pyrazolo[3,4-*d*]pyrimidine-3,6-diamine derivatives translates into a 1333-fold boost in the potency in ACK1 tyrosine kinase inhibition.^2^ Specifically, the installation of methyl groups can alter the metabolic stability, conformation, and solubility of bioactive molecules.^3−5^

**Scheme 1:**
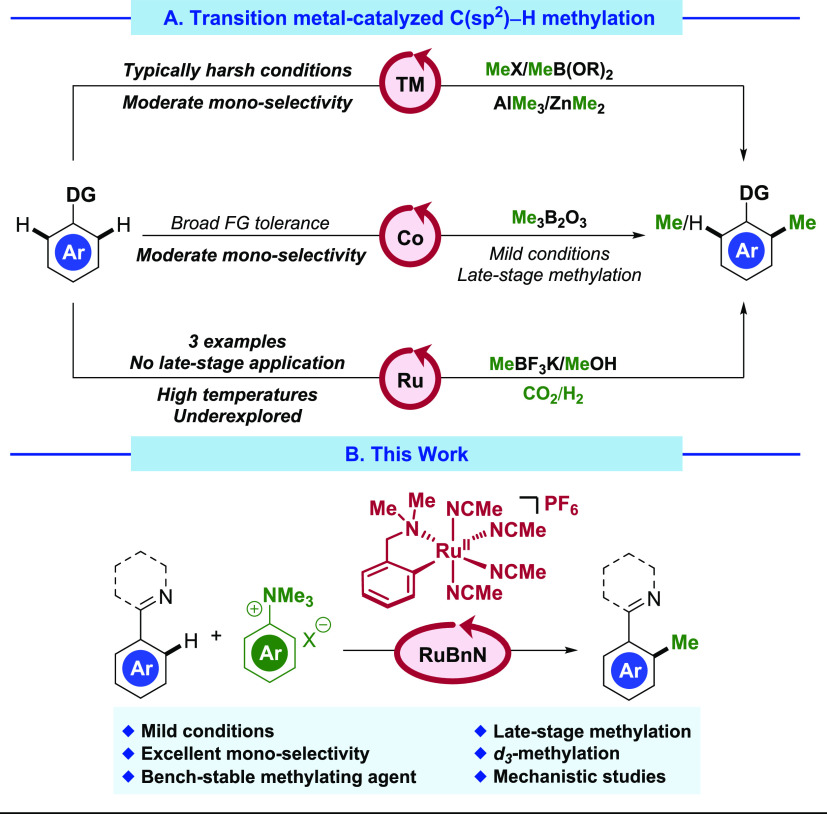
*Ortho*-Selective Directed C–H Methylation of Arenes^24,31−33^

Despite the importance of this transformation, there remains a need for more efficient synthetic methods to convert C–H bonds into C–Me bonds, as many current approaches suffer from the requirement of high temperatures, use of harsh reagents, and poor selectivity,^6−8^ issues that often limit their potential application toward late-stage methylation. Indeed, only one example to date has achieved broadly monoselective late-stage methylation without the preselection of *ortho*- and *meta*-functionalized substrates. Pilarski et al. developed a mechanochemical rhodium-catalyzed C–H methylation protocol using MeBF_3_K as the nucleophilic methyl source.^9^ Such transformations have been achieved using a number of transition metals including palladium,^10−15^ iron,^16−18^ manganese,^19,20^ nickel,^21^ iridium,^22^ cobalt,^6,23,24^ and rhodium,^25−28^ among others (Scheme 1A, top). In addition, significant progress has been made in the transition metal-free methylation of arenes.^29,30^ In 2020, Ackermann et al. reported a cobalt-catalyzed methylation protocol that addressed several of these limitations. A range of complex pharmaceutical molecules were *ortho*-methylated under relatively mild conditions with impressive functional group tolerance (Scheme 1A, center).^24^ This protocol, however, produces methane and ethane in a sealed system at 100 °C, posing a significant safety risk. Furthermore, only a poor-to-modest mono- vs bis-methylation selectivity was observed.

An area that has received significantly less attention is the ruthenium-catalyzed C–H methylation of arenes, in contrast to ruthenium′s ubiquity in C–H alkylation manifolds. Indeed, there are only three examples to date of ruthenium-catalyzed C–H methylation, with substrates limited to naphthols, pyrroles, and indoles (Scheme 1A, bottom).^31−33^ This lack of examples can be due to the fact that commonly used *N*-containing directing groups are not compatible with strong methylating agents such as MeI or MeOTf, being methylated at the nitrogen and making the desired directed C–H methylation impossible. In addition, these methods rely on high temperatures (120–160 °C), along with either the use or the evolution of hydrogen gas in pressurized systems^31,33^ Recently, our group reported the monoselective alkylation of directing group-containing arenes with primary alkyl halides under unprecedentedly mild conditions, enabled by the cyclometalated ruthenium precatalyst **RuBnN**.^34,35^ We hypothesized that by using an electrophilic methylating agent in concert with **RuBnN**, we would be able to develop the first broadly applicable ruthenium-catalyzed C–H methylation reaction (Scheme 1B).

## 2 Results and Discussion

### 2.1 Method Development

We began our investigation by attempting the methylation of 2-phenylpyridine (**1a**) with MeI in the presence of **RuBnN** (Table 1, entry 1). **3aa** and **4aa** were obtained in 41 and 9% yields, respectively, with a poor mass balance. ^1^H NMR analysis of the crude revealed significant *N*-methylation accounting for the lost material (see Figure S177). The reaction of **1a** with MeOTf (Table 1, entry 2) afforded 13% **3aa** and only traces of **4aa**, again with *N*-methylation being observed (see Figure S178). We hypothesized that the high electrophilicity of MeI and MeOTf was responsible for the poor C- vs N-chemoselectivity. In contrast, the less electrophilic primary and secondary alkyl bromides can undergo Ru-catalyzed *ortho*-alkylation chemoselectively.^34,36^ Quaternary ammonium salts have been well-explored as arylating agents and substrates for transition metal-catalyzed cross-coupling reactions;^37−40^ however, they are an underutilized class of reagents for C–H functionalization. A single report by Chatani and co-workers showed that *N,N,N*-trimethylanilinium salts are suitable methylating reagents for the nickel-catalyzed *ortho*-directed C(sp_2_)–H methylation of aromatic amides bearing an 8-aminoquinoline directing group.^41^ Such reagents offer significant advantages as bench-stable, noncarcinogenic, easy-to-handle solids. We were, therefore, delighted to observe a drastic increase in both the yield of **3aa** and in the mono-methylation selectivity when **2a** was employed (Table 1, entry 3). In the absence of **RuBnN** (Table 1, entry 4), the reaction did not proceed. In the absence of Na_2_CO_3_ (Table 1, entry 5), a marked decrease in reactivity was also observed. Performing the reaction in the absence of NaI (Table 1, entry 6) caused a minor reduction in yield, affording methylation products **3aa** and **4aa** in 71% and 3% yields, respectively. To explore the importance of a halogen source for this reaction, the PhNMe_3_PF_6_ salt was used in place of **2a** in the absence of NaI. In this case, only 6% of the desired product **3aa** was observed (Table 1, entry 7). Reducing the temperature to 40 °C resulted in extremely poor reactivity (Table 1, entry 8). Replacing **RuBnN** with [Ru(*p*-cymene)Cl_2_]_2_ as the precatalyst caused a reduction in the overall yield alongside a significant reduction in the monoselectivity (Table 1, entry 9).

**Table 1:**
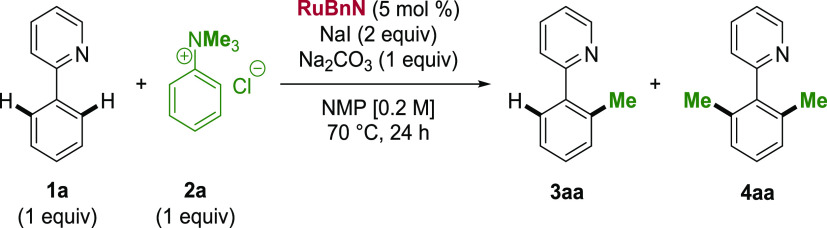
Initial Reaction Optimization

entry	deviation from standard conditions	**1a** (%)^a^	**3aa** (%)^a^	**4aa** (%)^a^	ratio **3aa/4aa**
1	MeI instead of **2a**	8^b^	41^b^	9^b^	5:1
2	MeOTf instead of **2a**	57^b^	13^b^	trace	
**3**	**none**	**6**	**91**	**3**	**>20:1**
4	no RuBnN	88	0	0	
5	no Na_2_CO_3_	87	7	trace	>20:1
6	no NaI	12	71	3	>20:1
7	PhNMe_3_PF_6_ instead of **2a**, no NaI	94	6	0	
8	40 °C	93	4	0	
9	[Ru(*p*-cymene)Cl_2_]_2_^c^	29	54	11	5:1

a Yields calculated by GC-FID using hexadecane as the internal standard.

b *N*-methylation of **1a**, **3aa**, and **4aa** observed by ^1^H NMR analysis in 2, 17, and 3%, respectively, for entry 1 and 7, 3, and 0% for entry 2.

c 2.5 mol % was used.

**Scheme 2:**
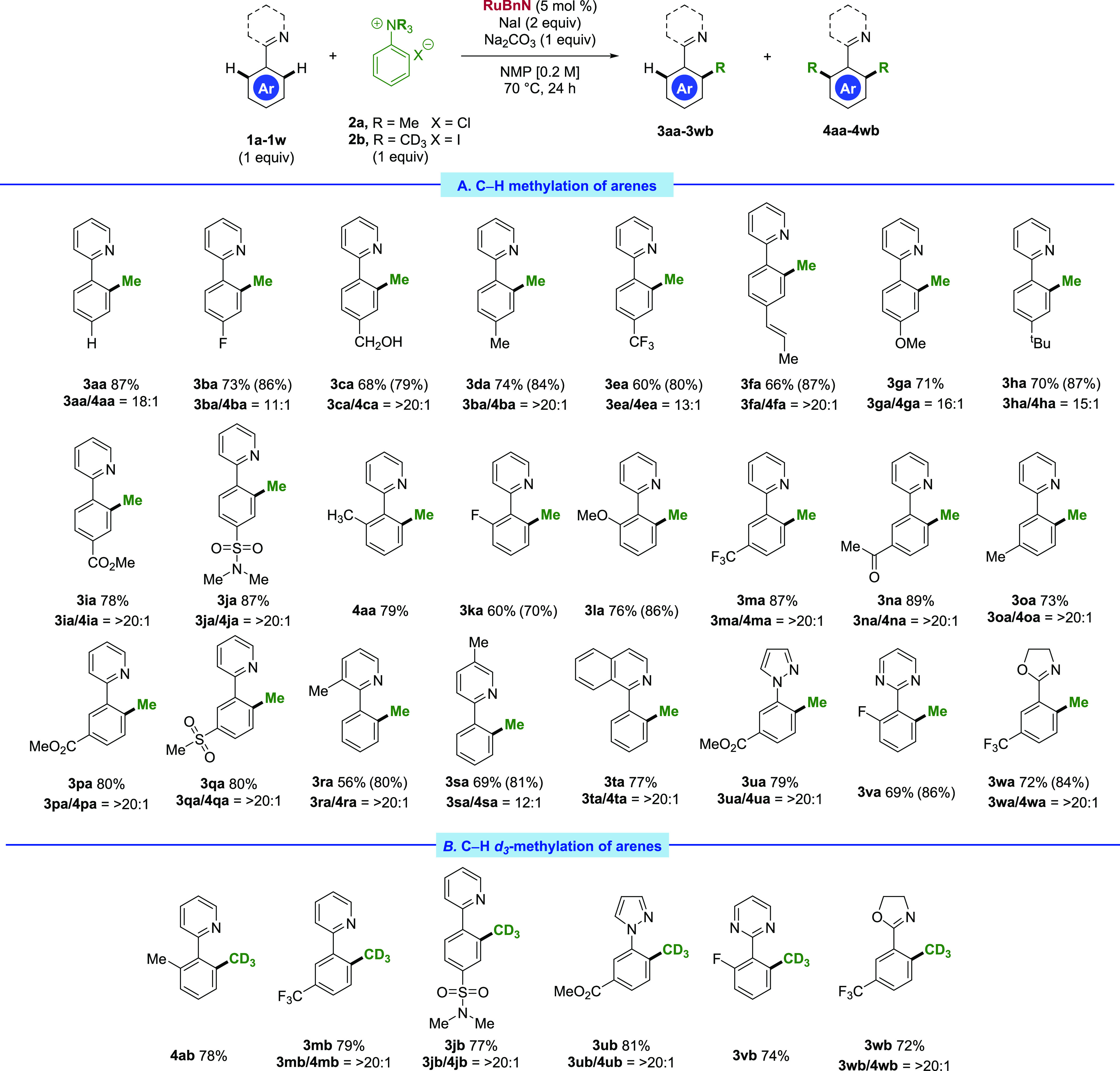
Substrate Scope for the Methylation and *d*_3_-Methylation of Directing Group-Containing Arenes Stated values correspond to the isolated yield of the pure monomethylated product **3aa**–**3wb**. Values within parentheses indicate yields of the monomethylated product in the crude mixture calculated by ^1^H NMR (NMR analysis using 1,3,5-trimethoxybenzene as the internal standard for yield losses of more than 10% during purification). **3**/**4** ratios correspond to the crude ratio, before purification.

With the optimized conditions in hand, we turned our attention to the substrate scope (Scheme 2). A wide variety of electron-donating and electron-withdrawing substituents were tolerated in the *ortho*-, *meta*- and *para*-positions of the arene partner (**3aa**–**3qa**). Synthetically relevant functional groups such as benzylic alcohols (**3ca**), olefins (**3fa**), esters (**3ia**), and sulfonamides (**3ja**), all delivered the desired monomethylation product in excellent yields with remarkably high selectivity toward monomethylation. The effects of changing the substitution pattern on the arene were also examined. Even though the methylation reaction is highly monoselective, o*rtho*-substituted phenylpyridines still reacted smoothly under the reaction conditions to give access to the methylated products **4aa**, **3ka**, and **3la**. Electron-withdrawing and electron-donating substituents were tolerated in the *meta*-position, with **3ma**–**3qa** being formed in good-to-excellent yields. The reaction tolerated carbonyl functionalities in the *meta*-position (**3na**) with no observed α-methylation, a known reaction mode for substrate **2a** in the presence of iodide and base.^42^ Furthermore, the reaction was shown to tolerate the medicinally relevant sulfone functional group, with **3qa** formed in 79% yield. The reaction was also tolerant of substitution on the pyridine ring; 3- and 5-methyl phenylpyridines **1r** and **1s** afforded products **3ra** and **3sa** in 56 and 61% isolated yields, respectively.

Subsequently, a range of *N*(sp^2^)-containing substrates were tested for their directing-group capabilities. Isoquinoline performed comparably to pyridine, with **3ta** being formed in 77% yield. Pyrazole (**3ua**) and pyrimidine (**3va**) direction was also showcased, affording the respective products in 79 and 69% yields. Interestingly, this reaction was also successful with oxazolines (**3wa**), an incompatible directing group in previous **RuBnN** alkylation manifolds using unactivated alkyl halides,^34,43^ with 72% of the desired product isolated. Thus far, all attempts to use our conditions to achieve C(sp^3^)–H methylation (principally 2-isopropylpyridine) have proven unsuccessful, with no C(sp^3^)–H methylation observed in any case.

Site-selective installation of a *d*_3_-methyl group, particularly in the late stage, represents a valuable yet underexplored class of transformations.^44^ Incorporation of a −CD_3_ group in place of −CH_3_ into pharmaceutically active compounds is known to potentially reduce metabolic toxicity, susceptibility to oxidative metabolism, and undesired drug interactions.^45−47^ We hypothesized that our protocol could be extended to *ortho*-directed *d*_3_-methylation. Experimental data matched our hypothesis with comparable yields observed with respect to the analogous methylation reactions. *ortho*- (**4ab**), *meta*- (**3mb**), and *para*- (**3jb**) substituted phenylpyridines were well-tolerated, as were other previously compatible directing groups such as pyrazoles (**3ub**), pyrimidines (**3vb**), and oxazolines (**3wb**).

**Scheme 3:**
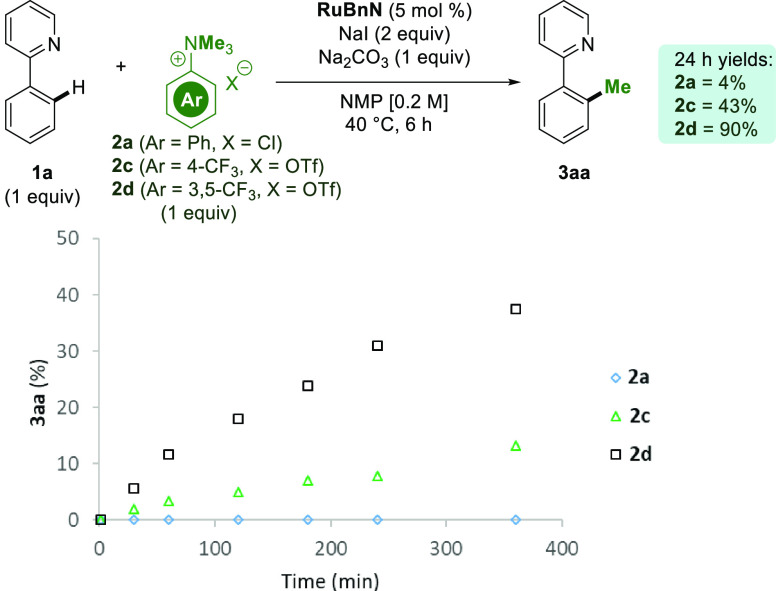
Exploration of Electron-Deficient Ammonium Salts

Despite the broad functional-group tolerance and efficient reactivity demonstrated with substituted phenylpyridines and simple directing group-containing arenes, employing **2a** for late-stage methylation proved unfruitful, with only trace amounts of methylation observed across most substrates. The group of Reid^48^ recently showcased an extensive study on the rates of decomposition for various trimethylammonium salts, concluding that *O*–methylation proceeded by the slow release of MeI as the active electrophilic methyl source. Electron-deficient ammonium salts were observed to decompose faster and were significantly more reactive. Inspired by these findings, we studied the reactivity of several electron-deficient anilinium salts, which were shown to be markedly more reactive in our reaction (Scheme 3). When **2a** was used at 40 °C, only trace amounts of the product were observed after 6 h. The use of **2c** in place of **2a** drastically increased the observed rate of methylation, with **2d** further enhancing the rate. When the reaction was left for 24 h using **2d**, **3aa** was obtained in an excellent yield. Importantly, the proposed faster in situ release of MeI occurred with no observed loss in monoselectivity.

**Scheme 4:**
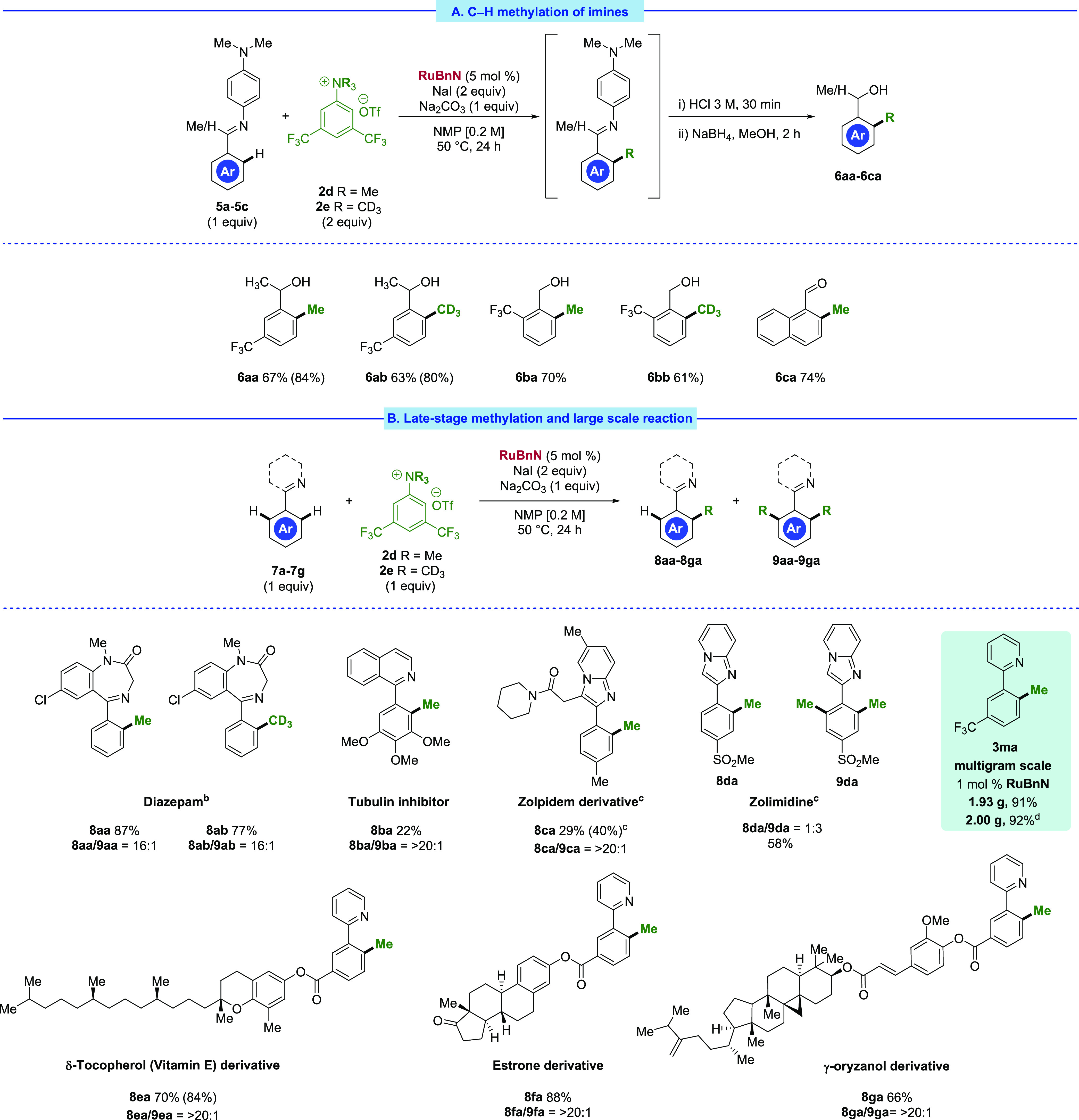
Expanded Substrate Scope Using Optimized Salts **2d** and **2e** Stated values correspond to the isolated yield of the pure monomethylated product **3aa**–**3wb**. Values within parentheses indicate yields of the monomethylated product in the crude mixture calculated by ^1^H NMR (NMR analysis using 1,3,5-trimethoxybenzene as the internal standard for yield losses of more than 10% during purification). **3**/**4** ratios correspond to the crude ratio, before purification. 70 °C instead of 50 °C. 0.1 equiv of NaI. Reaction carried out in acetone instead of NMP.

Encouraged by these results, we refocused our attention toward the methylation and *d*_3_-methylation of substrates that reacted sluggishly with **2a**, instead employing the newly optimized salt **2d** (Scheme 4). Gratifyingly, imines served as capable directing groups for methylation with **2d**; methylation of the ketimine **5a** at 50 °C afforded **6aa** in 67% isolated yield. As a result of their volatile nature, ketone and aldehyde products afforded post-hydrolysis were reduced and isolated as benzylic alcohols. Using the ammonium salt **2e**, the analogous *d*_3_-methylated product **6ab** was isolated in a comparable 63% yield. Aldimine direction was harnessed in the methylation and *d*_3_-methylation reaction of **5b**, affording methylated (**6ba**) and *d*_3_-methylated (**6bb**) products in good yields. In addition, the reaction of **5c** afforded the desired methylated aldehyde **6ca** after hydrolysis in a 74% isolated yield.

Subsequently, we directed our attention toward the late-stage functionalization and derivatization of pharmaceutical and other biologically active compounds. Both methylation and *d*_3_-methylation of diazepam proceeded efficiently, affording methylated **8aa** in 87% isolated yield and *d*_3_-methylated **8ab** in 77% isolated yield. Reaction with **8ba**, a known tubulin inhibitor scaffold,^49^ afforded the desired monomethylated product in a modest yield of 22%, illustrating tolerance toward highly electron-rich substrates such as **7b**. The monomethylated zolpidem derivative **8ca** was isolated in a reasonable yield of 29% and with excellent monoselectivity. Significant reactivity was additionally observed with zolimidine, although with reduced selectivity, yielding monomethylated **8da** in 14% and bismethylated **9da** in 44% yield. In addition, tocopherol (**8ea**)-, estrone (**8fa**)-, and oryzanol (**8ga**)-derived methylated products were synthesized effectively, with respective isolated yields of 70, 88, and 66%.^50^ Although **2d** also yielded good results for the compounds addressed in Scheme 2, we limited the use of this salt for challenging substrates, as **2a** is cheap and readily commercially available. On the other hand, maintaining low temperatures was found to be key to obtaining good results for drugs and imine derivatives. Poor yields were observed when these substrates were subjected to the reaction with the salt **2d** at 70 °C (see Table S13).

Furthermore, the method is susceptible to straightforward scale-up and significant lowering of the catalyst loading. Excellent reactivity was observed on the multigram scale methylation of **1m** using only 1 mol % **RuBnN**, affording 1.93 g of **3ma** in 91% isolated yield. In addition, the reaction can be carried out in acetone, avoiding the need for NMP, in an equally high conversion, with 92% of **3ma** isolated

### 2.2 Mechanistic Studies

**Figure 1:**
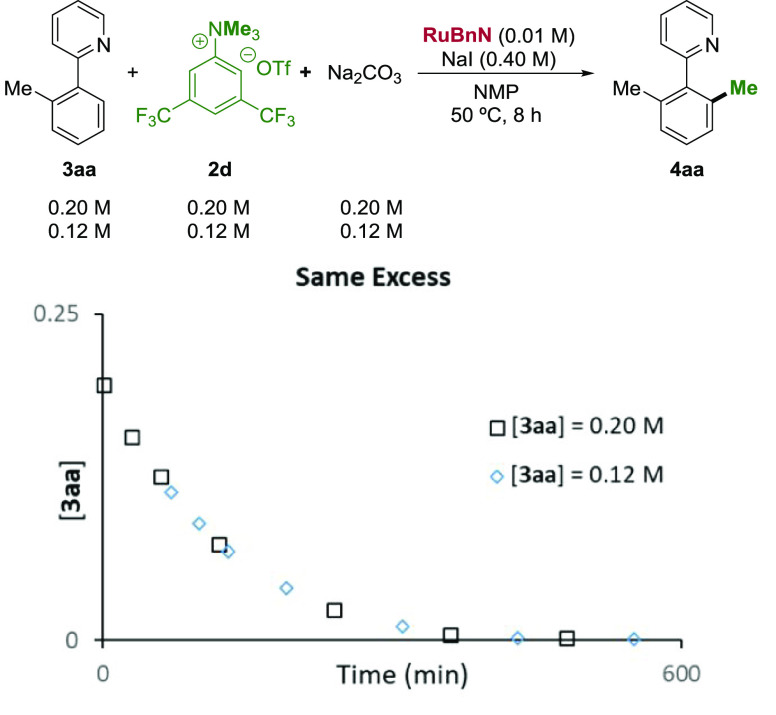
Same excess experiment.

**Figure 2:**
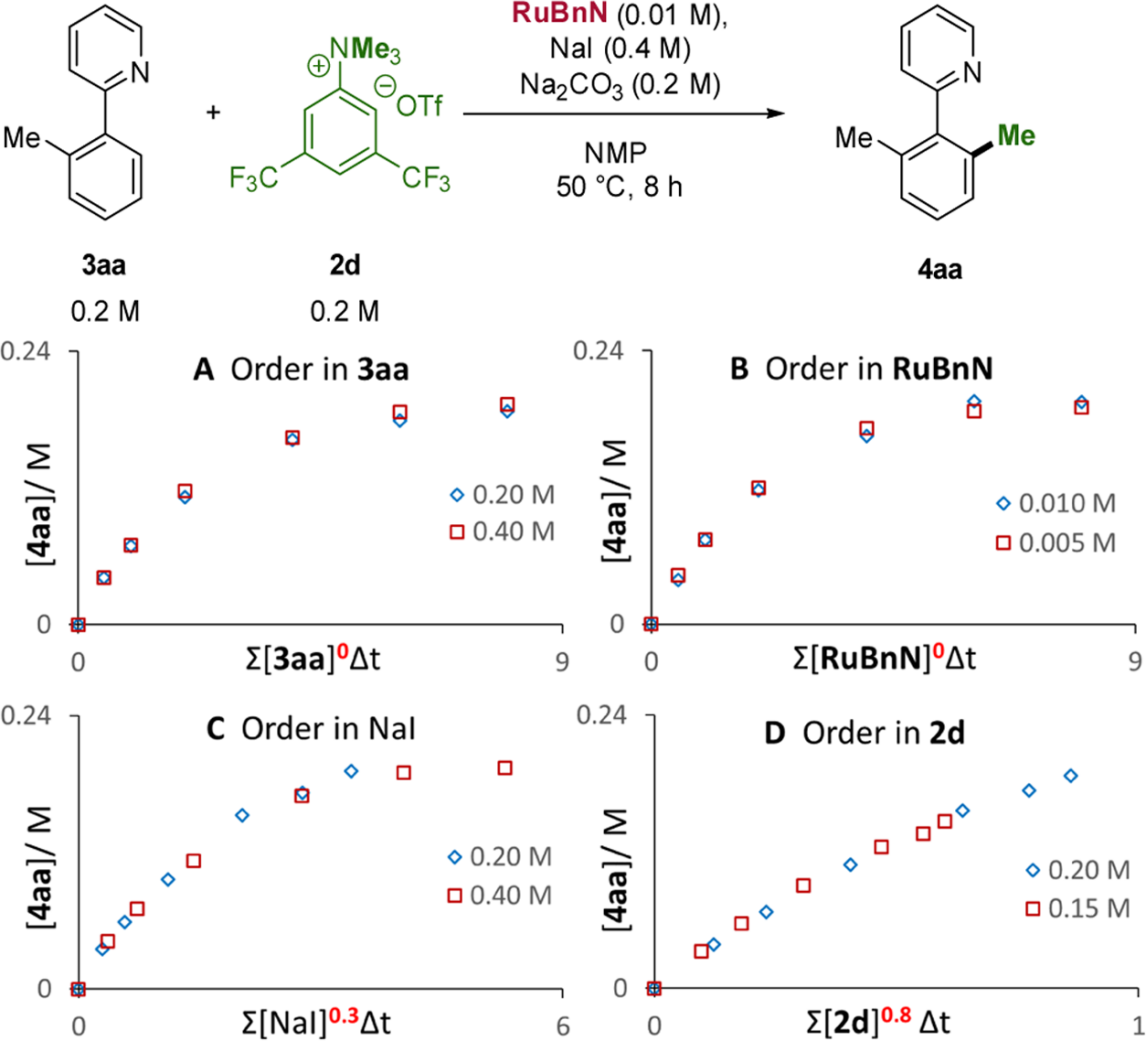
VTNA analysis.

To gain insight into the reaction mechanism, a series of experiments were designed. First, the same excess experiment was carried out to determine whether catalyst decomposition or product inhibition occurs during the reaction of **3aa** with **2d** (Figure 1).^51^ A perfect overlay was found, indicating that neither catalyst decomposition nor product inhibition is prevalent. Subsequently, kinetic orders of the reaction components for the reaction of **3aa** with **2d** were determined by applying the variable time normalization analysis (VTNA) method developed by Burés et al.^52−54^ Our analysis revealed reaction orders of 0.8 in ammonium salt, 0.3 in NaI, and 0 in the catalyst, base, and starting material (Figure 2 and Section 8 in the Supporting Information). These orders are not consistent with an on-cycle rate-determining step and, instead, indicate that the slow formation of MeI from the reaction of **2d** with NaI is a probable off-cycle, rate-determining step.

**Scheme 5:**
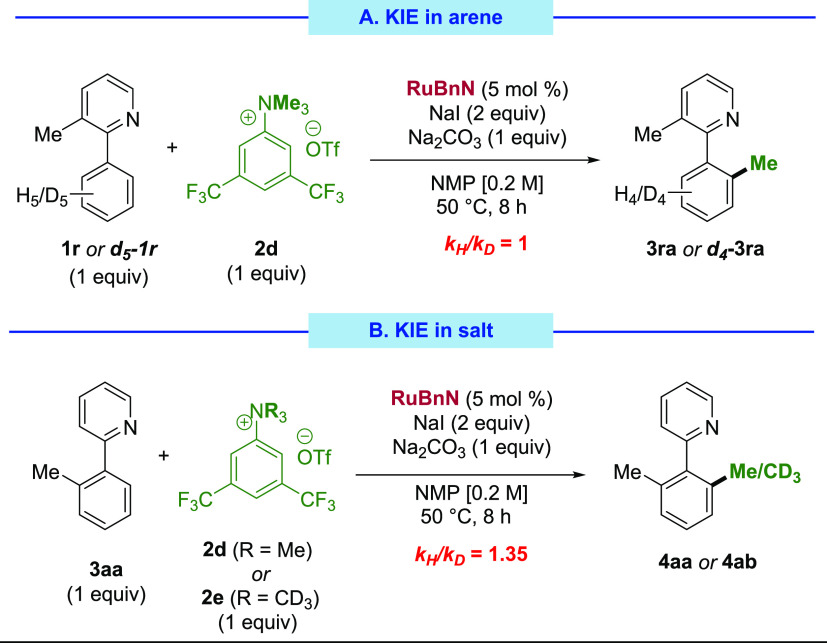
KIE Experiments

Two experiments were designed to measure the H/D kinetic isotopic effects (KIE) within our reaction (Scheme 5). When the rate of reaction of **1r** with **2d** was compared with that of ***d***_**5**_**-1r** with **2d** via parallel kinetic runs, a KIE of 1 was measured. This is in accordance with the previously measured order 0 on the directing group-containing arene using VTNA (Scheme 5A), indicating that the C–H activation step does not influence the rate of the reaction. On the other hand, we also performed parallel kinetic runs comparing the reaction between **3aa** and **2d** with that between **3aa** and the *d*_9_-ammonium salt **2e**. This experiment revealed a KIE of 1.35, or 1.12, per deuterium in each methyl group (Scheme 5B). This value is within the expected range for a secondary KIE and is consistent with our hypothesis of an off-cycle rate-determining step involving the reaction of the ammonium salt **2d** with NaI.^55^

**Scheme 6:**
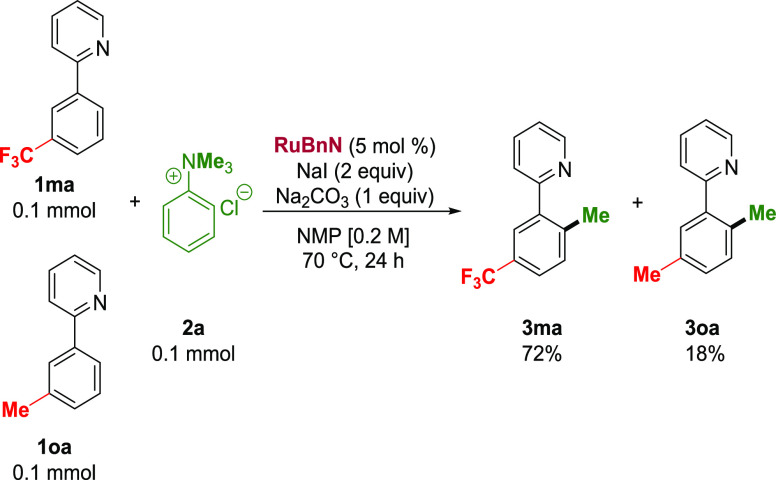
Competition Experiment between Electron-Rich and Electron-Deficient Directing Group-Containing Arenes

A competition experiment carried out between electron-deficient (**1ma**) and electron-rich (**1oa**) phenylpyridines revealed a major bias toward methylation of the electron-deficient substrate,^56^ with a 4:1 (*m*-CF3: *m*-Me) ratio observed (Scheme 6). This ratio may arise from differing thermodynamic stabilities of the corresponding bis-cycloruthenated intermediates or from their differing reactivities toward the oxidative addition with MeI.

**Scheme 7:**
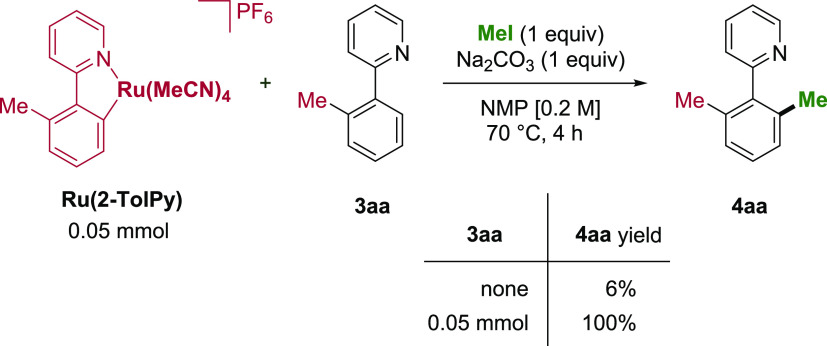
Stoichiometric Reaction with the Mono-Cyclometalated Complex Ru(2-TolPy)

Previous studies carried out by our group outlined the requirement of a key bis-cyclometalated ruthenium intermediate to afford significant reactivity in both *ortho*-arylation and alkylation manifolds.^34,36,57^ To assess whether a similar pathway is followed, the stoichiometric reactivity of the mono-cycloruthenated complex **Ru(2-TolPy)** was probed (Scheme 7). Using MeI as the methylating agent, the reaction afforded **4aa** in only 6% yield. However, when 1 equiv of **3aa** was added to the reaction, full conversion to **4aa** was observed, indicating that the catalytic process relies on the in situ formation of the bis-cyclometalated ruthenium species.

**Figure 3:**
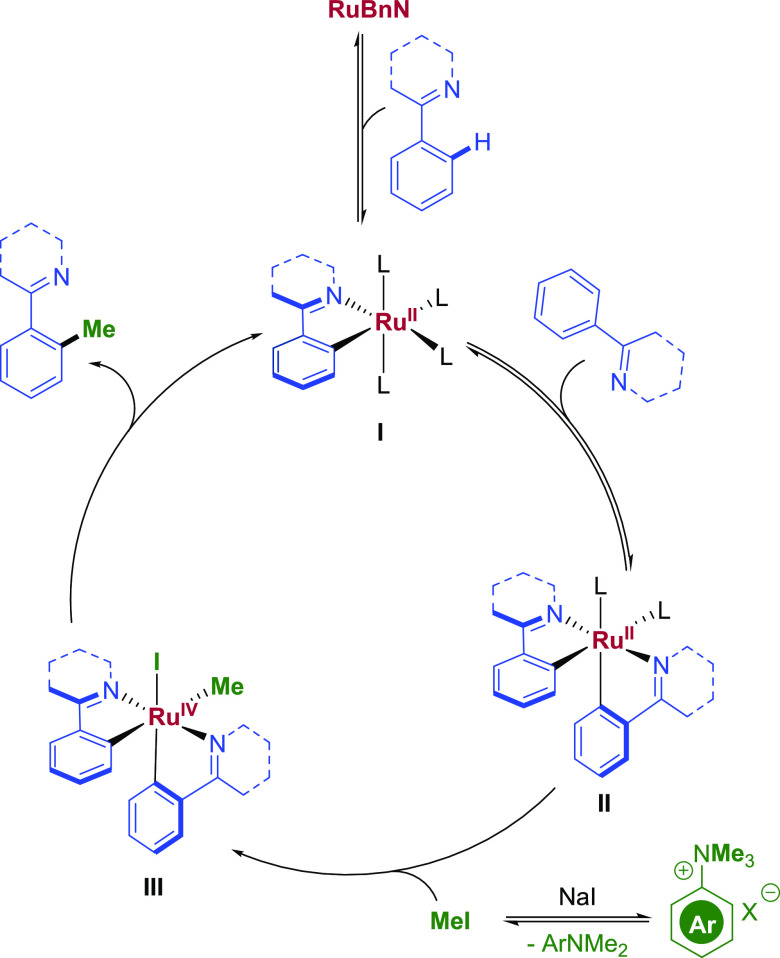
Proposed catalytic cycle: L = MeCN or NMP.

Accounting for the observed kinetic and mechanistic data, we propose the reaction mechanism outlined in Figure 3. First, the precatalyst **RuBnN** undergoes initial C–H activation of the directing group-containing arene, forming the mono-cyclometalated complex **I**. A second C–H activation step on a second molecule of the substrate follows to form **II**, as likely the resting state of the catalytic species (consistent with the KIE and orders in **3aa** and base). An off-cycle reaction of the quaternary anilinium salt with NaI liberates MeI as the rate-determining step (consistent with the order zero in the Ru catalyst). Complex **II** undergoes oxidative addition with MeI to form intermediate **III**, which subsequently reductively eliminates to form the desired product closing the catalytic cycle.

## 3 Conclusions

In summary, we report an efficient protocol for the ruthenium-catalyzed *ortho*-methylation of directing group-containing arenes. Employing quaternary anilinium salts as the electrophilic methylating agent has enabled previously unprecedented high mono-selectivities across a range of substrates with *d*_3_-methylation, late-stage functionalization, and catalyst loadings as low as 1 mol %. A detailed mechanistic investigation reveals that the rate-limiting in situ formation of MeI underpins the observed reactivity and selectivity. We envisage that quaternary ammonium salts could be used more generally as a class of functional group transfer reagents to access enhanced reactivity and selectivity in future ruthenium-catalyzed C–H functionalization protocols.

## Supporting Information Available

Experimental procedures and analytical data of the new compounds (PDF)

## Dedication

Dedicated to the memory of Prof. Dr. Kilian Muñiz.
