# Supplementary material for: Ruthenium-Catalyzed Monoselective C–H Methylation and d3-Methylation of Arenes
Source: JACS Au. 2022 Oct 21;2(11):2529–38. doi: 10.1021/jacsau.2c00399 (PMC9709947; doi:10.1021/jacsau.2c00399)
Supplement: Supplementary file 1 — au2c00399_si_001.pdf [file au2c00399_si_001.pdf]

Supplemental information

Ruthenium-Catalyzed Mono-Selective C–H Methylation and d_3-Methylation of Arenes

Ashley Hogg,[1] Matthew Wheatley,[1] Pablo Domingo-Legarda,[1] Asier Carral-Menoyo,[1] Naomi Cottam[1] and Igor Larrosa*[1]

[1] School of Natural Sciences, Department of Chemistry, University of Manchester, Oxford Road, Manchester M13 9PL, United Kingdom
[*] Corresponding Author. **Email**: Igor.Larrosa@manchester.ac.uk

Table of Contents

1. GENERAL INFORMATION S3

2. PREPARATION OF RUTHENIUM COMPLEXES S5

3. GENERAL PROCEDURES S9

4. OPTIMIZATION OF REACTION CONDITIONS S17

4. SYNTHESIS OF STARTING MATERIALS S26

5. CHARACTERIZATION DATA FOR MONO-METHYLATED PRODUCTS. S41

6. LARGE SCALE REACTION S90

7. MECHANISTIC STUDIES S92

8. KINETIC EXPERIMENTS S96

9. NMR SPECTRA S109

1. General Information

All of the methylation reactions were set up in an argon-filled glovebox with oven-dried crimp-cap microwave vials (10 mL). The reactions were then capped (using PK100 20MM BUTYL SEPTA) and taken outside the glovebox to run. All starting materials were purchased from Acros (Fisher), Aldrich (Merck), Alpha Aesar (Fisher) and Fluorochem and were used without further purification, unless otherwise stated. All solvents and liquid reagents were degassed with 3 freeze-pump-thaw cycles. Purification by flash column chromatography was carried out on silica gel, particle size 40-63 mm, using a Biotage Isolera™ Four, with SNAP 50 g cartridge, using the stated solvent system at a flow rate of 10 ml/min. Purification by preparative reverse-phase HPLC was carried out on an Agilent 1260 Infinity (column: ACE 10 C-18-Ar; 250 x 21.2 mm) using the stated solvent system at a flow rate of 22 mL min^{-1}. Purification by preparative normal-phase HPLC was carried out on an Agilent 1260 Infinity (column: ACE 10 SIL 250 x 21.2 mm) using the stated solvent system at a flow rate of 22 mL min^{-1}. All HPLC samples were fully dissolved in sufficient amounts of the respective mobile phase employed at the beginning of the gradient, with total separation occurring over multiple runs of 0.1 mL injection of the crude mixture.

High resolution mass spectra were performed by the School of Chemistry Mass Spectrometry Service (University of Manchester) employing a Thermo Finnigan MAT95XP spectrometer. IR spectra were recorded using a Thermo Scientific Nicolet iS5 FTIR machine, relevant bands are quoted in cm^{-1}. ^{1}H NMR, ^{19}F NMR and ^{13}C NMR spectra were recorded at 400, 500 or 600 MHz on Bruker instruments. ^{1}H NMR are referenced to the residual solvent peak at 7.26 ppm ($CDCl_3$), 5.32 ppm (CD_2Cl_2), 2.05 ppm ($(CD_3)_2CO$), or 1.94 ppm (CD_3CN). ppm values are quoted to 2 decimal places, with coupling constants (J) to the nearest 0.1 Hz. ^{13}C NMR spectra were recorded at 151, 126 or 101 MHz and quoted in ppm to 1 decimal place

with coupling constants (*J*) to the nearest 0.1 Hz. The spectra were referenced to the residual solvent peak at 77.2 ppm ($CDCl_3$), 53.5 ppm (CD_2Cl_2) 39.5 ppm ($(CD_3)_2CO$) or 1.3 ppm (CD_3CN). ^{19}F NMR spectra recorded at 471 or 376 MHz in $CDCl_3$ and quoted in ppm to 1 decimal place with coupling constants (*J*) to the nearest 0.1 Hz. ^{2}H NMR are referenced to the residual solvent peak at 7.26 ppm ($CDCl_3$), 5.32 ppm (CD_2Cl_2), 2.05 ppm ($(CD_3)_2CO$), or 1.94 ppm (CD_3CN). ppm values are quoted to 2 decimal places, with coupling constants (*J*) to the nearest 0.1 Hz.

2. Preparation of ruthenium complexes

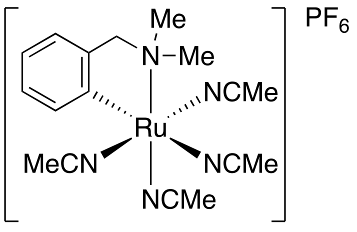

RuBnN

RuBnN:[1] An oven dried 100 mL Ace pressure tube equipped with a stirring bar was transferred to a glove box, then $[RuCl_2(benzene)]_2$ (440 mg, 0.88 mmol, 0.55 equiv), NaOH (96.0 mg, 2.40 mmol, 1.50 equiv), KPF_6 (589 mg, 3.20 mmol, 2 equiv), *N,N*-dimethylbenzylamine (216 mg, 1.60 mmol, 1 equiv) and MeCN (10 mL, 0.16 M) were added. The tube was sealed, transferred out of the box and placed in an oil bath at 45 °C and the reaction was stirred for 3 h. Upon completion, the reaction crude was loaded in an aluminium oxide (Al_2O_3, neutral) column conditioned with CH_2Cl_2, and quickly eluted with MeCN under N_2 collecting the yellow/orange band. The solution was concentrated under reduced pressure and then quickly precipitated with Et_2O affording a yellow solid/orange solid, which was promptly transferred to a glove box as it decomposes turning green/blue if exposed to air. This solid and MeCN (20 mL) were added to an oven dried 100 mL Ace pressure tube equipped with a stirring bar and the reaction was stirred for 24 h at 100 °C. After this time, the reaction mixture was filtered through a short plug of aluminium oxide, eluted with MeCN, concentrated under vacuum, and precipitated with Et2O/pentane (1:1) affording **RuBnN** as an off-white solid (653 mg, 75 %). **RuBnN** must be kept in a glove box as it quickly decomposes turning blue/black if exposed to air. The complexes are subjected to quantitative ^1H NMR after their synthesis. They are generally in the region of 99% pure by this measure. If they are of lower purity, then the complex should be dissolved in MeCN inside the glovebox and filtered through a small plug

of alumina. Then it should be concentrated under vacuum and crashed out with Et_2O/Pentane (1:1).

^{1}H NMR (400 MHz, CD_3CN) δ 7.56 (d, *J* = 7.6 Hz, 1H), 6.88-6.95 (m, 2H), 6.76 (app. t, *J* = 7.4 Hz, 1H), 3.64 (s, 2H), 2.46 (s, 6H), 2.42 (s, 3H), 2.23 (s, 6H), 1.97 (s, 3H).

^{13}C NMR (100 MHz, CD_3CN) δ 175.2, 149.6, 138.6, 125.3, 123.3, 123.1, 121.5, 121.2, 73.6, 53.7, 4.4, 4.2.

^{19}F NMR (376 MHz, CD_3CN) δ −72.9 (d, *J* = 705.4 Hz).

IR ν_{max} (neat/cm^{-1}) 3048, 2977, 2926, 2897, 2855, 2265, 1575, 1472, 1442, 831, 748

HRMS (ESI^{+}) calcd. for $C_{15}H_{21}N_4Ru^{+}$ [M–MeCN– PF_6]$^{+}$: 359.0804. Mass found: 359.0798.

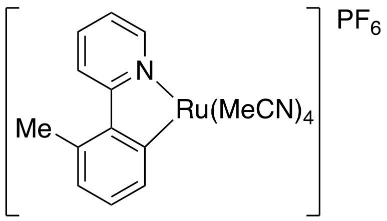

Ru(2-TolPy)

Ru(2-TolPy):[1] An oven dried 100 mL Ace pressure tube equipped with a stirring bar was transferred to a glove box, then $[RuCl_2(p\text{-cymene})]_2$ (490 mg, 0.80 mmol, 0.5 equiv), KOAc (236 mg, 2.40 mmol, 1.5 equiv), KPF_6 (589 mg, 3.20 mmol, 2 equiv), 2-(*o*-tolyl)pyridine (271 mg, 1.60 mmol, 1 equiv) and MeCN (10 mL, 0.16 M) were added. The tube was sealed, transferred out of the box, placed in an oil bath at 100 °C and stirred for 24 h. Upon completion, the reaction crude was loaded in an aluminium oxide (Al_2O_3, neutral) column conditioned with CH_2Cl_2 and quickly eluted with CH_3CN/CH_2Cl_2 (1:1) using N_2 in replacement of compressed air collecting the yellow/orange band. The solution was concentrated under reduced pressure and then quickly precipitated with Et_2O affording the desired **Ru(2-Tol-Py)** (879 mg, 95 %) as a yellow/orange solid. The complex has to be promptly transferred to a glove box as it decomposes turning green if exposed to air. The complexes are subjected to quantitative ^{1}H NMR after their synthesis. They are generally in the region of 99% pure by this measure. If they are of lower purity, then the complex should be dissolved in MeCN inside the glovebox and filtered through a small plug of alumina. Then it should be concentrated under vacuum and crashed out with Et_2O/Pentane (1:1).

^{1}H NMR (500 MHz, CD_3CN) δ 9.05 (d, *J* = 5.5 Hz, 1H), 8.09 (d, *J* = 7.5 Hz, 1H), 7.88 (d, *J* = 7.5 Hz, 1H), 7.75 (app. t, *J* = 7.5 Hz, 1H), 7.14 (app. t, *J* = 6.5 Hz, 1H), 6.94 (app. t, *J* = 7.5 Hz, 1H), 6.74 (d, *J* = 7.5 Hz, 1H), 2.68 (s, 3H), 2.50 (s, 3H), 2.00 (s, 6H), 1.96 (s, 3H)

^{13}**C NMR** (125 MHz, CD_3CN) δ 187.7, 169.9, 153.8, 146.4, 136.9, 136.6, 136.1, 127.5, 126.4, 123.7, 122.9, 121.7, 121.4, 24.7, 4.4, 3.8 (signals for both carbons of one of the MeCN ligands were not observed)

^{19}**F NMR** (470 MHz, CD_3CN) δ –73.0 (d, *J* = 705.9 Hz)

IR ν_{max} (neat/cm^{-1}) 3046, 2274, 1605, 1498, 1269, 1169, 830, 768

HRMS (ESI$^+$) calcd for $C_{18}H_{19}N_4Ru$ $[M\text{-}MeCN\text{-}PF_6]^+$: 393.0648, found 393.0635.

3. General Procedures

General Procedure A: RuBnN catalyzed *ortho-* C–H methylation of directing group-containing arenes using quaternary ammonium salts as methylating agents.

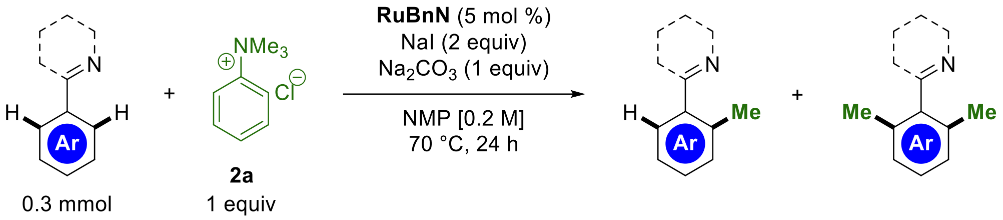

Scheme S1. Reaction Scheme for **General Procedure A**.

All reactions were set up inside an argon-filled glovebox. To an oven-dried microwave vial (10 mL) sealed with a two-piece crimp-camp seal (butyl rubber septa, 20mm) and containing a magnetic stirring bar, was added **RuBnN** (8.10 mg, 5 mol %, 0.015 mmol), NaI (89.9 mg 0.60 mmol, 2 equiv), *N*,*N*,*N*-trimethylanilinium chloride **2a** (56 mg, 0.30 mmol, 1 equiv), Na_2CO_3 (32 mg, 0.30 mmol, 1 equiv), directing group-containing arene (0.30 mmol, 1 equiv), and NMP ([0.2 M], 1.5 mL). The vial was capped, removed from the glovebox, and stirred at the stated temperature for 24 hours. Upon completion, the vial was decapped and the crude reaction mixture was extracted with Et_2O (50 mL) and washed with water (3 x 30 mL). The organic layers were combined, washed with brine, dried over Na_2SO_4 and excess solid filtered. The crude product mixture was then loaded onto a silica gel column and purified by flash column chromatography to afford pure product.

General Procedure B: RuBnN catalyzed *ortho*- C–H methylation of directing group-containing complex molecules using electron-deficient quaternary ammonium salts as methylating agents.

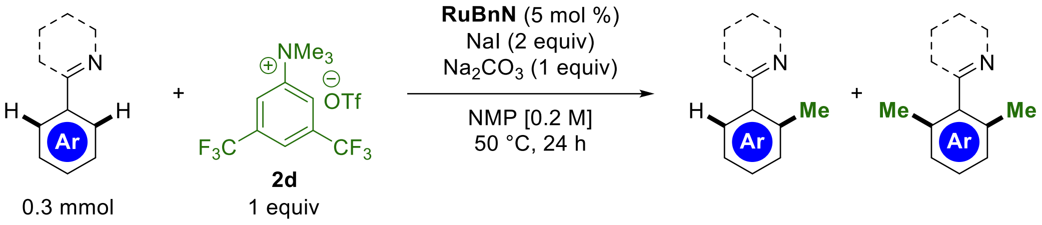

Scheme S2. Reaction Scheme for **General Procedure B**.

All reactions were set up inside an argon-filled glovebox. To an oven-dried microwave vial (10 mL) sealed with a two-piece crimp-camp seal (butyl rubber septa, 20mm) and containing a magnetic stirring bar, was added **RuBnN** (8.10 mg, 5 mol %, 0.015 mmol), NaI (90 mg, 0.60 mmol, 2 equiv), *N*,*N*,*N*-trimethyl-3,5-bis(trifluoromethyl)anilinium triflate **2d** (126 mg, 0.300 mmol, 1 equiv), Na_2CO_3 (32 mg, 0.30 mmol, 1 equiv), directing group-containing arene (0.30 mmol, 1 equiv), and NMP ([0.2 M], 1.5 mL). The vial was capped, removed from the glovebox, and stirred at the stated temperature for 24 hours. Upon completion, the vial was decapped and the crude reaction mixture was extracted with Et_2O (50 mL) and washed with water (3 x 30 mL). The organic layers were combined, washed with brine, dried over Na_2SO_4 and excess solid filtered. The crude product mixture was then loaded onto a silica gel column and purified by flash column chromatography to afford pure product.

General Procedure C: RuBnN catalyzed *ortho*- C–H d_3-methylation of directing group-containing arenes using quaternary ammonium salts as methylating agents.

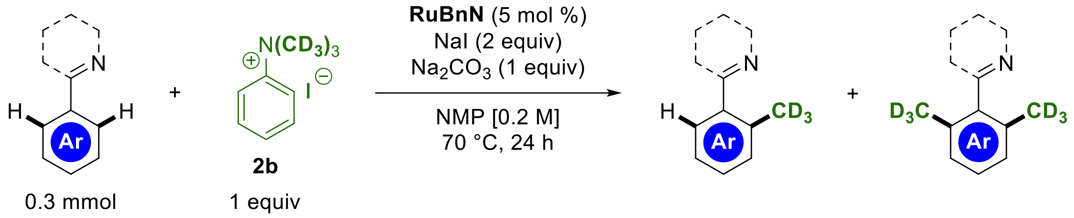

Scheme S3. Reaction Scheme for **General Procedure C**.

All reactions were set up inside an argon-filled glovebox. To an oven-dried microwave vial (10 mL) sealed with a two-piece crimp-camp seal (butyl rubber septa, 20mm) and containing a magnetic stirring bar, was added **RuBnN** (8.10 mg, 5 mol %, 0.015 mmol), NaI (90 mg, 0.60 mmol, 2 equiv), *N*,*N*,*N*-tri(d_3-methyl)anilinium iodide **2b** (56 mg, 0.30 mmol, 1 equiv), Na_2CO_3 (32 mg, 0.30 mmol, 1 equiv), directing group-containing arene (0.30 mmol, 1 equiv), and NMP ([0.2 M], 1.5 mL). The vial was capped, removed from the glovebox, and stirred at the stated temperature for 24 hours. Upon completion, the vial was decapped and the crude reaction mixture was extracted with Et_2O (50 mL) and washed with water (3 x 30 mL). The organic layers were combined, washed with brine, dried over Na_2SO_4 and excess solid filtered. The crude product mixture was then loaded onto a silica gel column and purified by flash column chromatography to afford pure product. *Contamination of the reaction mixture with H_2O leads to H/D exchange in the final d_3-methylated products. Extra care should be taken to exclude H_2O during the reaction setup.*

General Procedure D: RuBnN catalyzed directed *ortho-* C–H *d_3*-methylation of directing group-containing complex molecules using electron-deficient quaternary ammonium salts as methylating agents.

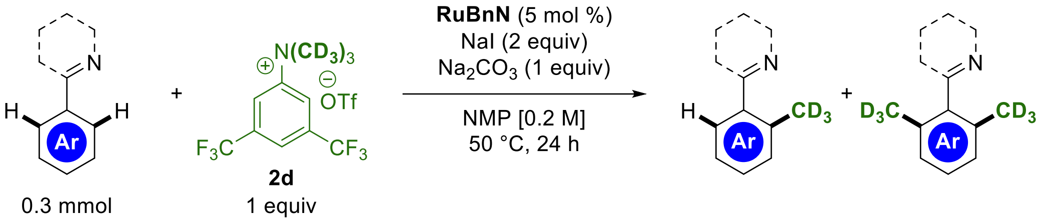

Scheme S4. Reaction Scheme for **General Procedure D**.

All reactions were set up inside an argon-filled glovebox. To an oven-dried microwave vial (10 mL) sealed with a two-piece crimp-camp seal (butyl rubber septa, 20mm) and containing a magnetic stirring bar, was added **RuBnN** (8.10 mg, 5 mol %, 0.015 mmol), NaI (90 mg, 0.60 mmol, 2 equiv), *N*,*N*,*N*-tri(*d_3*-methyl)-3,5-bis(trifluoromethyl)anilinium triflate **2e** (126 mg, 0.300 mmol, 1 equiv), Na_2CO_3 (32 mg, 0.30 mmol, 1 equiv), directing group-containing arene (0.30 mmol, 1 equiv), and NMP ([0.2 M], 1.5 mL). The vial was capped, removed from the glovebox, and stirred at the stated temperature for 24 hours. Upon completion, the vial was decapped and the crude reaction mixture was extracted with Et_2O (50 mL) and washed with water (3 x 30 mL). The organic layers were combined, washed with brine, dried over Na_2SO_4 and excess solid filtered. The crude product mixture was then loaded onto a silica gel column and purified by flash column chromatography to afford pure product. *Contamination of the reaction mixture with H_2O leads to H/D exchange in the final d_3-methylated products. Extra care should be taken to exclude H_2O during the reaction setup.*

General Procedure E: EDC coupling for the formation of esters.

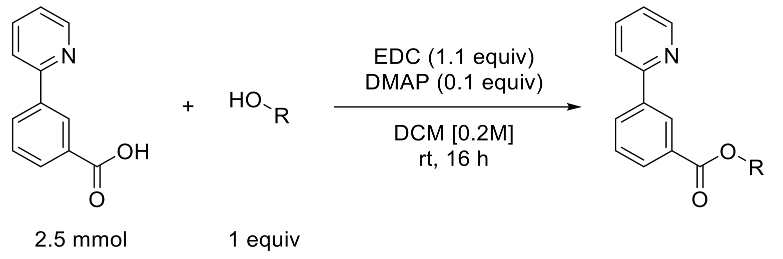

Scheme S5. Reaction Scheme for **General Procedure E**

To an 50 mL round bottom flask equipped with a magnetic stirrer bar was added 3-(pyridin-2-yl)benzoic acid (0.50 g, 2.5 mmol), EDC (0.54 mg, 2.8 mmol, 1.1 equiv), DMAP (31 mg, 0.25 mmol, 0.1 equiv), and the corresponding alcohol (1 equiv). DCM was subsequently added [13 mL, 0.2 M]. The flask was sealed using a suba-seal and stirred at room temperature for 16 h. Upon completion, the reaction was diluted with water (10 mL) and extracted into DCM (3 x 10 mL). The crude mixture was dried under vacuum, loaded onto a silica gel column, and purified using flash column chromatography to afford pure product.

General Procedure F: synthesis of 2-arylpyridine substrates

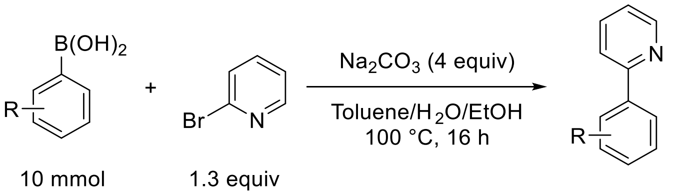

Scheme S6. Reaction Scheme for **General Procedure F**

A modification of the Chang procedure was used.[2] To a solution of 2-bromopyridine derivative (10 mmol) in toluene (35 mL), EtOH (7.5 mL), and H_2O (35 mL) was added Na_2CO_3 (9.5 g, 90 mmol, 9 equiv), $Pd(PPh_3)_4$ (347 mg, 0.300 mmol, 0.03 equiv) and boronic acid (13.0 mmol) under argon in a 250 mL two-necked flask equipped with a magnetic stirrer bar. The reaction mixture was refluxed overnight. Upon completion, the reaction was cooled to room temperature and diluted with aqueous NH_4Cl (15 mL). Organics were extracted with ethyl acetate (3x30 mL) with the organic extracts combined and washed with saturated brine (30 mL), dried over $MgSO_4$, and evaporated in vacuum to afford the crude product, which was subsequently purified by flash column chromatography on silica gel to obtain pure product.

General Procedure G: synthesis of ketimine substrate

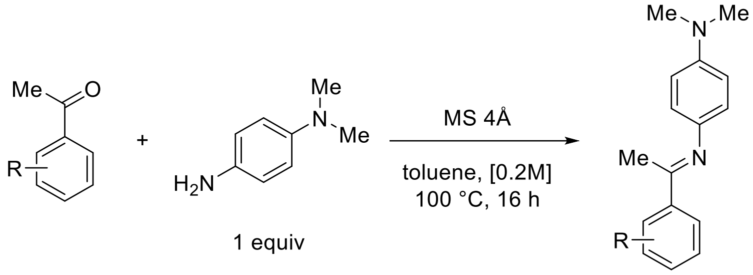

Scheme S7. Reaction Scheme for **General Procedure G**.

To an oven-dried sealed tube equipped with a magnetic stirring bar was added ketone (1.0 equiv), *N,N*-Dimethyl-*p*-phenylenediamine (1.2 equiv), 4Å molecular sieves (600 mg $mmol^{-1}$), and toluene ([0.2 M]). The mixture was heated at 100 °C overnight, after which the reaction was allowed to cool to room temperature and concentrated in vacuo. The solid residue was washed with cold pentane and recrystallized in the stated solvent mixture to yield the corresponding ketimine substrate.

General Procedure H: synthesis of aldimine substrates

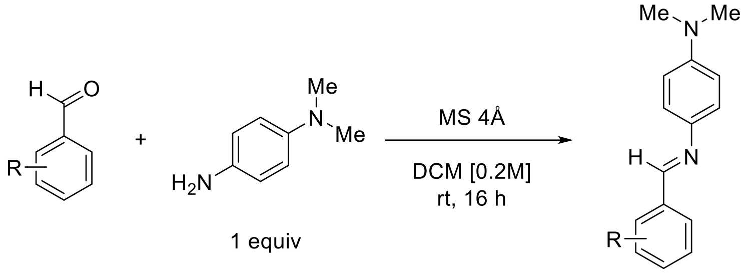

Scheme S8. Reaction Scheme for **General Procedure H**.

To an oven-dried round bottom flask equipped with a magnetic stirring bar was added the aldehyde (1.0 equiv), *N,N*-Dimethyl-*p*-phenylenediamine (1.0-1.2 equiv), molecular sieves, and DCM ([0.2 M]). The mixture was stirring at room temperature overnight, after which the reaction was concentrated in vacuo. The solid residue was washed with cold pentane and recrystallized in the stated solvent mixture to yield the corresponding aldimine substrate.

4. Optimization of Reaction Conditions

Table S1. Screening of electrophilic methyl sources

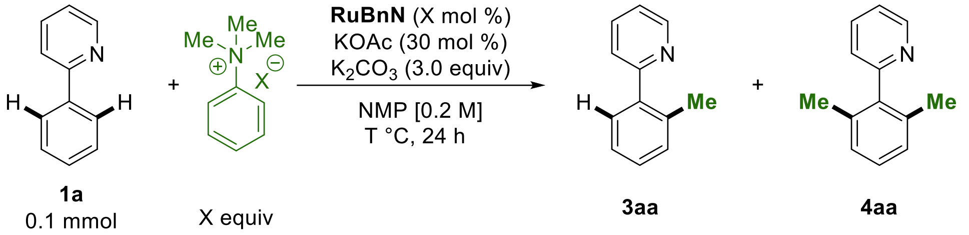

Entry	X (equiv amm. salt)	RuBnN (mol %)	T (°C)	1a (%)[a]	3aa (%)[a]	4aa (%)[a]
**1**	I (1)	10	50	66	14	trace
**2**	I (1.5)	10	50	56	19	trace
**3**	I (2)	10	50	61	25	trace
**4**	I (2)	5	50	59	21	trace
**5**	I (3)	10	50	45	38	1
**6[a]**	I (2)	10	50	27	31	0
**7**	I (1)	10	70	7	80	4
**8**	I (2)	10	70	traces	36	53
**9**	I (3)	10	70	traces	2	61
**10**	I (1)	5	70	5	64	4
**11**	Br (1)	10	70	6	71	4
**12**	Br (2)	10	50	67	10	traces
**13**	PF_6 (2)	10	50	69	1	0

[a]Yield obtained by GC-FID using hexadecane as the internal standard. [b]KTFA used instead of KOAc.

Table S2. Additives screen

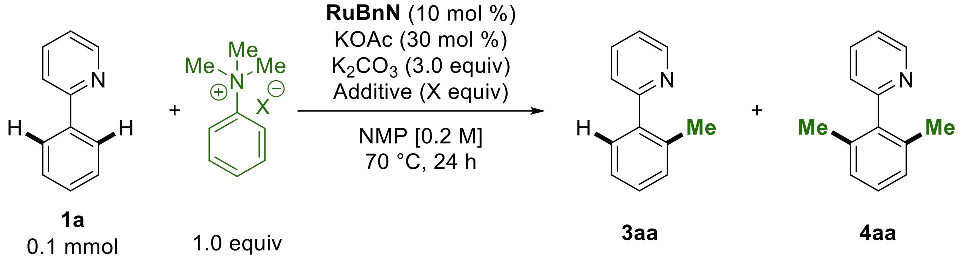

Entry	X	Additive (equiv)	1a (%)[a]	3aa (%)[a]	4aa (%)[a]
**1**	PF_6	KI (0.1)	70	14	trace
**2**	PF_6	KI (1)	33	54	1
**3**	Br	KI (0.3)	5	75	4
**4**	Br	KI (1)	5	81	5
**5**	Br	CsI (0.3)	12	75	3
**6**	Br	CsI (1)	7	83	4
**7**	Br	CsBr (0.3)	7	76	4
**8**	Br	CsBr (1)	11	73	3
**9[a]**	**Br**	**CsI (1.5)**	**13**	**84**	**4**
**10[a]**	Br	CsI (2)	11	82	4
**11[a]**	Br	CsI (3)	13	84	4
**12**	Br	TBAI (0.3)	15	71	3
**13**	Br	TBAI (1)	15	73	4

[a]Yield obtained by GC-FID using hexadecane as the internal standard. [b] 5 mol % of RuBnN used.

Table S3. Carboxylates screen

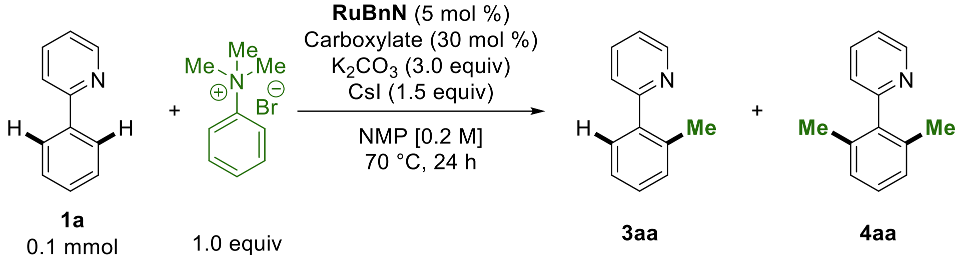

Entry	Carboxylate	1a (%)[a]	3aa (%)[a]	4aa (%)[a]
**1**	-	3	76	7
**2**	KOAc	18	81	3
**3**	NaOAc	18	79	3
**4**	LiOAc	10	81	4
**5**	CsOAc	25	72	3
**6**	$Cu(OAc)_2$	99	traces	-
**7**	AgOAc	72	12	9
**8**	KOCOPh	14	85	4
**9**	$PhP(O)O_2K_2$	3	85	7
**10**	NaOPiv	26	64	2
**11**	CsOPiv	28	70	3
**12**	LiTFA	4	85	6
**13**	**NaTFA**	**5**	**86**	**5**
**14**	KTFA	2	83	7

[a]Yield obtained by GC-FID using hexadecane as the internal standard.

Table S4. Base screen

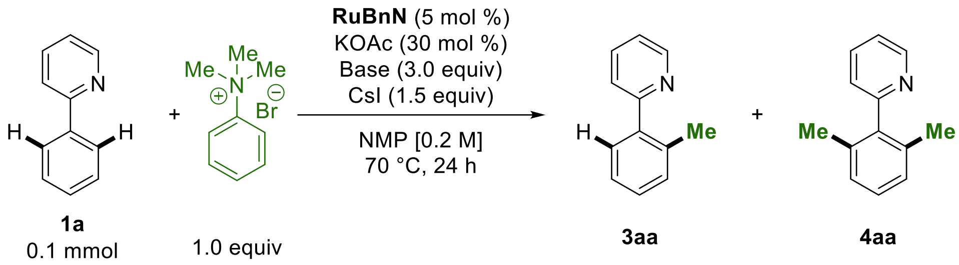

Entry	Base	1a (%)[a]	3aa (%)[a]	4aa (%)[a]
**1**	-	39	25	traces
**2**	K_2CO_3	18	81	3
**3**	Li_2CO_3	14	83	2
**4**	**Na_2CO_3**	**14**	**86**	**3**
**5**	Cs_2CO_3	44	49	3
**6**	K_3PO_4	19	79	3

[a]Yield obtained by GC-FID using hexadecane as the internal standard.

Table S5. Iodide salts screen

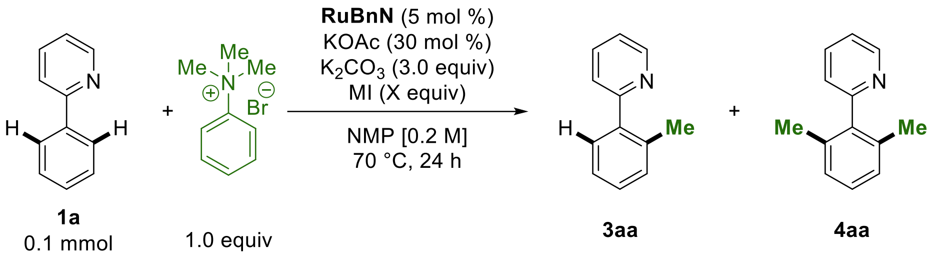

Entry	MI (equiv)	1a (%)[a]	3aa (%)[a]	4aa (%)[a]
**1**	LiI (1.5)	8	86	4
**2**	NaI (1.5)	6	86	4
**3**	KI (1.5)	7	84	4
**4**	CsI (1.5)	18	81	3
**5**	TBAI (1.5)	23	66	4
**6**	NaI (0.3)	22	70	3
**7**	NaI (1)	10	86	4
**7**	**NaI (2)**	**6**	**89**	**4**

[a]Yield obtained by GC-FID using hexadecane as the internal standard.

Table S6. NaTFA equivalents screen

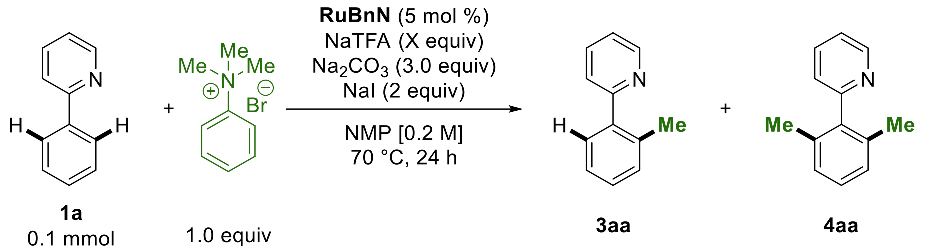

Entry	NaTFA (equiv)	1a (%)[a]	3aa (%)[a]	4aa (%)[a]
**1**	**0.3**	**6**	**84**	**5**
**2**	0.5	5	85	6
**3**	0.7	7	84	5
**4**	1	5	78	6

[a]Yield obtained by GC-FID using hexadecane as the internal standard.

Table S7. Base equivalents screen

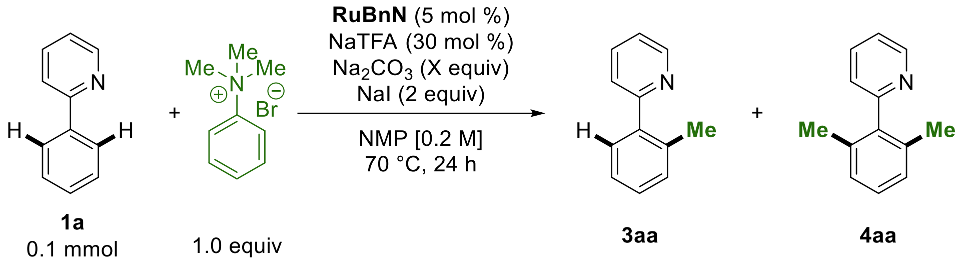

Entry	Na_2CO_3 (equiv)	1a (%)[a]	3aa (%)[a]	4aa (%)[a]
**1**	2	5	84	6
**2**	3	6	84	5
**3**	4	6	84	5
**4**	5	5	84	6

[a]Yield obtained by GC-FID using hexadecane as the internal standard.

Table S8. Ammonium salt counter anion screen

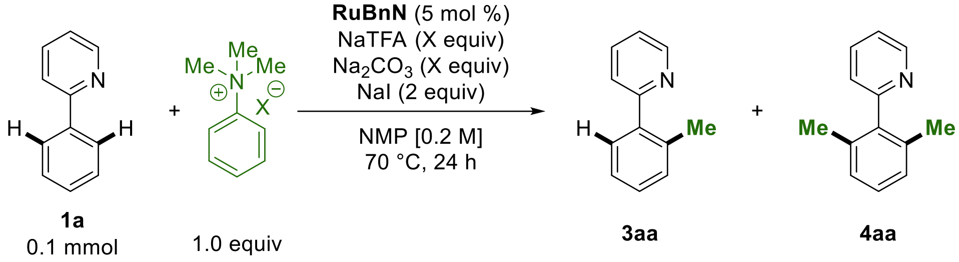

Entry	X	NaTFA (equiv)	Base (equiv)	1a (%)[a]	3aa (%)[a]	4aa (%)[a]
**1**	Br	0.5	2	10	86	3
**2**	Br	0.2	2	8	88	4
**3**	Br	0	2	9	86	3
**4**	Cl	0.5	2	7	85	3
**5**	Cl	0.2	2	4	90	4
**6**	Cl	0	2	4	91	5
**7**	**Cl**	**0**	**1**	**6**	**91**	**3**

[a]Yield obtained by GC-FID using hexadecane as the internal standard.

Table S9. Other electrophilic methyl sources screen using optimized reaction conditions

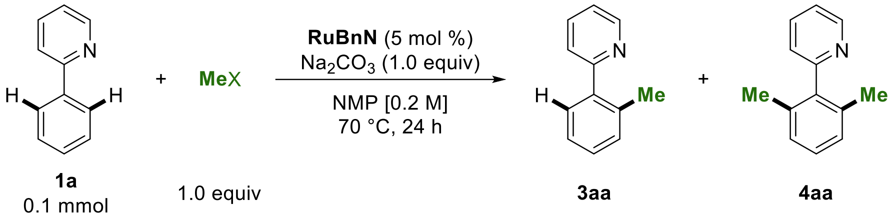

Entry	MeX	1a (%)[a]	3aa (%)[a]	4aa (%)[a]	*N*-Me 1a (%)[b]	*N*-Me 3aa (%)[b]	*N*-Me 4aa (%)[b]
**1**	**$PhNMe_3Cl$**	**6**	**91**	**3**	**-**	**-**	**-**
**2**	MeI	8	41	9	2	17	3
**3**	MeOTf	57	13	trace	7	3	-

[a]Yield obtained by GC-FID using hexadecane as the internal standard. [b] Yield obtained by ^{1}H-NMR using 1,3,5-trimethoxybenzene as the internal standard.

Table S10. Solvent screen using the optimized reaction conditions.

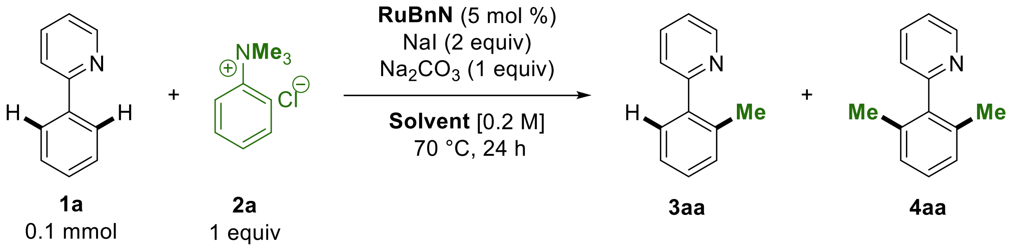

Entry	Solvent	1a (%)	3aa (%)	4aa (%)
**1**	**NMP**	**15**	**84**	**2**
**2**	Acetone	22	71	trace
**3**	Dioxane	68	28	trace
**4**	THF	78	16	trace
**5**	*i*PrOH	88	6	-
**6**	Cyrene	79	4	-
**7**	HFIP	96	1	-
**8**	DCM	91	1	-

Yields obtained by GC-FID using hexadecane as the internal standard.

Table S11. Comparison of ruthenium pre-catalysts in the standard reaction.

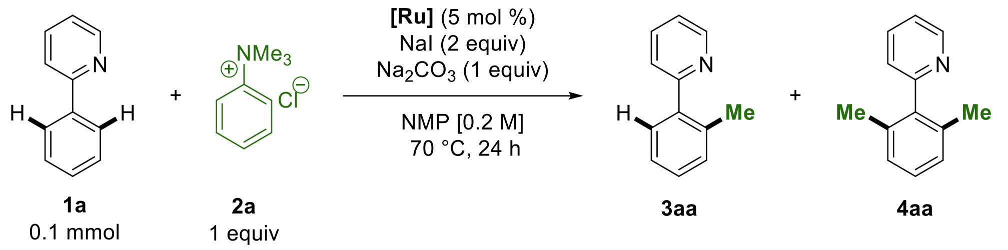

Entry	[Ru]	1a (%)	3aa (%)	4aa (%)
**1**	**RuBnN**	**6**	**91**	**3**
**2**	$[Ru(Cl_2)$*p*-cymene$]_2$[a]	29	54	11

Yields obtained by GC-FID using hexadecane as the internal standard. [a]2.5 mol % was used.

Table S12. Comparison of salt additives in the standard reaction.

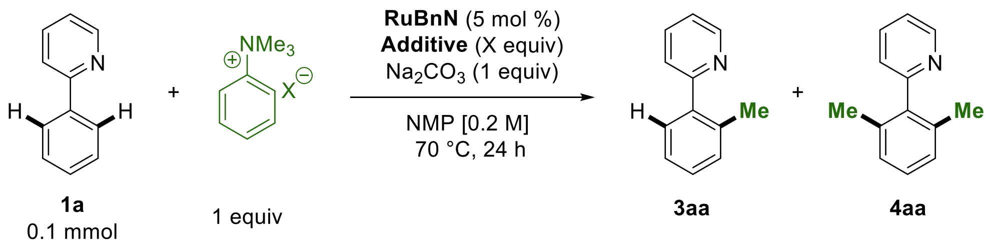

Entry	X	Additive	1a (%)	3aa (%)	4aa (%)
**1**	PF_6	-	94	6	-
**2**	PF_6	2 equiv NaI	8	82	4
**3**	PF_6	2 equiv NaCl	60	39	trace
**4**	Cl	-	13	71	3
**5**	Cl	2 equiv NaI	6	91	3
**6**	Cl	1 equiv NaCl	6	80	5
**7**	Cl	1 equiv KPF_6	75	16	-
**8**	Cl	2 equiv KPF_6	83	8	-

Yields obtained by GC-FID using hexadecane as the internal standard.

Table S13. Comparison of ammonium salts 2a and 2d under different conditions

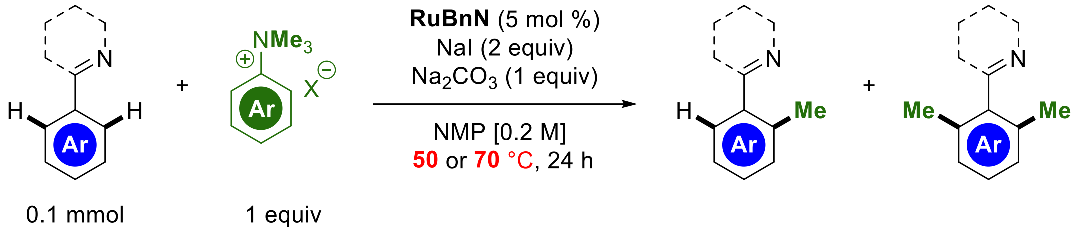

Entry	Substrate	T (°C)	Salt	SM (%)	Mono (%)	Bis (%)
1	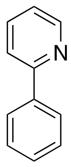	50	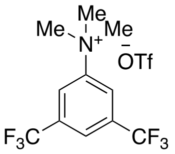	5	88	2
2		70		1	92	4
3		50	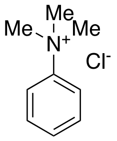	81	18	0.5
4		70		3	91	3
5	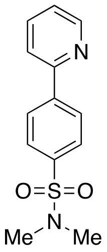	50	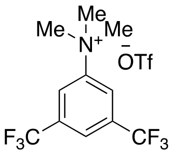	2	84	2
6		70		2	88	-
7		50	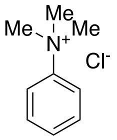	90	8	-
8		70		4	87	3
9	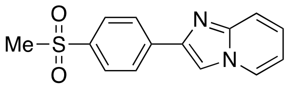	50	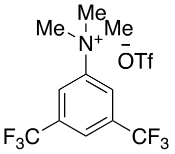	30	16	42
10		70		87	8	1
11	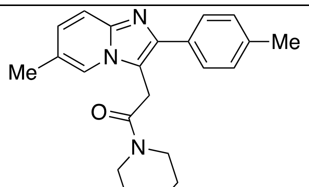	50	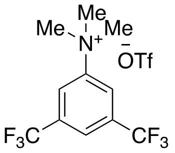	22	12	-
12		70		51	40	-
13	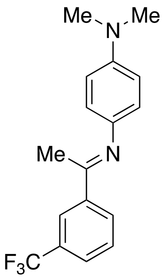	50	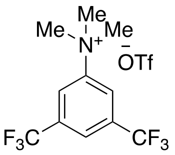	-	84	-
14		70		48	42	-

Yield obtained by ^{1}H-NMR using 1,3,5-trimethoxybenzene as the internal standard.

4. Synthesis of Starting Materials

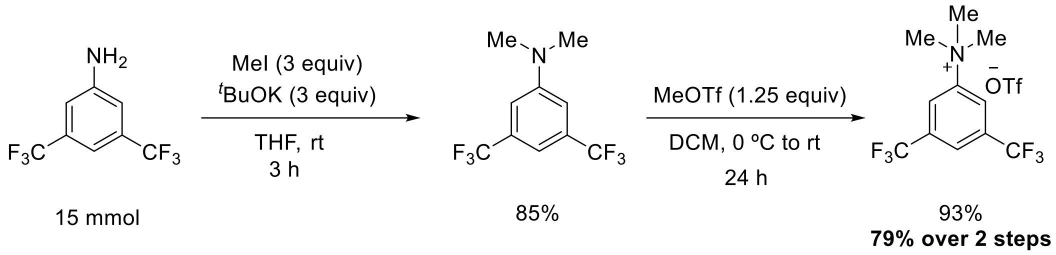

Procedure for the preparation of *N,N*-Dimethyl-3,5-bis(trifluoromethyl)aniline

A modification of the procedure reported by Roberts and Jones was employed.[3] A round bottom flask was charged with *t*BuOK (5.1 g, 45 mmol, 3 equiv) and 3 evacuation-refill cycles were performed with argon. To this was added anhydrous THF (120 mL) followed by 3,5-bis(trifluoromethyl)aniline (2.4 mL, 15 mmol) and the suspension stirred. Methyl iodide (2.8 mL, 45 mmol, 3 equiv) was added slowly over the course of 30 seconds and the reaction stirred at room-temperature for 3 hours. Excess *t*BuOK was filtered off and THF removed by rotary evaporation until ~ 5 mL remained. The reaction mixture was then diluted with Et_2O (30 mL) and deionised water (30 mL), the organic layer separated, and the aqueous layer extracted with Et_2O (3x30 mL). The combined organic extracts were washed with brine (50 mL), dried with Na_2SO_4 and concentrated to yield the desired crude product, which was subsequently purified using flash column chromatography (98:2 Hexane:EtOAc) to afford *N,N*-Dimethyl-3,5-bis(trifluoromethyl)aniline as an off-white solid (3.3 g, 85 %). Spectroscopic data match those previously reported.[4]

^{1}H NMR (400 MHz, $CDCl_3$) δ 7.15 (s, 1H), 7.01 (s, 2H), 3.05 (s, 6H).

^{13}C NMR (101 MHz, $CDCl_3$) δ 150.6, 132.3 (q, *J* = 32.5 Hz), 123.9 (q, *J* = 274.0 Hz), 111.3 (q, *J* = 3.9 Hz), 109.2 (hept., *J* = 3.9 Hz), 40.5.

^{19}F NMR (376 MHz, $CDCl_3$) δ -63.1.

Procedure for the preparation of *N,N,N*-trimethyl-3,5-bis(trifluoromethyl)anilinium triflate 2d

N,*N*-Dimethyl-3,5-bis(trifluoromethyl)aniline (3.29 g, 12.7 mmol) was added to a round bottom flask and 3 evacuation-refill cycles were performed with nitrogen. Anhydrous dichloromethane (20 mL) was subsequently added, and the resulting solution stirred. Methyl trifluoromethylsulfonate (1.74 mL, 15.9 mmol, 1.25 equiv) was added dropwise and the reaction stirred at room-temperature for 24 hours. The observed white precipitate was filtered, washed with excess Et_2O (40 mL) and dried in a vacuum desiccator. Product **2d** was afforded as a colourless solid (4.99 g, 93 %).

^{1}H NMR (400 MHz, $(CD_3)_2CO$) δ 8.82 (s, 2H), 8.37 (s, 1H), 4.08 (s, 9H).

^{13}C NMR (126 MHz, $(CD_3)_2CO$) δ 148.7, 133.1 (q, *J* = 34.5 Hz), 124.6 (hept, *J* = 3.5 Hz), 122.7 (q, *J* = 2.5 Hz), 122.7 (q, *J* = 273.3 Hz), 122.2 (q, *J* = 321.6 Hz), 57.2.

^{19}F NMR (471 MHz, $(CD_3)_2CO$) δ -63.2, -79.1.

Melting Point 221 ºC.

HRMS (ESI$^+$) calcd for $C_{11}H_{12}NF_6$ $[M+H]^+$: 272.0868, found 272.0856.

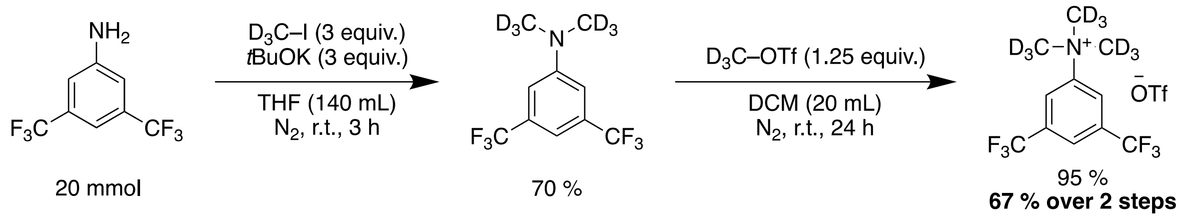

Procedure for the preparation of *N,N*-bis(trideuteromethyl)-3,5-bis(trifluoromethyl)aniline

A modification of the procedure reported by Roberts and Jones was employed.[3] A round bottom flask was charged with *t*BuOK (6.73 g, 60.0 mmol, 3 equiv,) and 3 evacuation-refill cycles were performed with argon. To this was added anhydrous THF (140 mL) followed by 3,5-bis(trifluoromethyl)aniline (3.12 mL, 20.0 mmol) and the suspension stirred. Trideuteromethyl iodide (3.73 mL, 60.0 mmol, 3 equiv) was added slowly over the course of 30 seconds and the reaction stirred at room-temperature for 3 hours. Excess *t*BuOK was filtered off and THF removed by rotary evaporation until ~ 5 mL remained. The reaction mixture was then diluted with Et_2O (30 mL) and deionised water (30 mL), the organic layer separated, and the aqueous layer extracted with Et_2O (3x30 mL). The combined organic extracts were washed with brine (50 mL), dried with Na_2SO_4 and concentrated to yield the desired crude product, which was subsequently purified using flash column chromatography (98:2 Hexane:EtOAc) to afford *N,N*-bis(trideutereomethyl)-3,5-bis(trifluoromethyl)aniline as an off-white solid (3.69 g, 70 %).

^{1}H NMR (400 MHz, $CDCl_3$) δ 7.14 (s, 1H), 6.99 (s, 2H).

^{2}H NMR (61 MHz, $CDCl_3$) δ 3.02.

^{13}C NMR (126 MHz, $CDCl_3$) δ 150.7, 132.3 (q, *J* = 32.3 Hz), 123.8 (q, *J* = 272.8 Hz), 111.1 (q, *J* = 4.2 Hz), 108.9 (sept. *J* = 4.0 Hz), 39.4 (sept., *J* = 20.7).

^{19}F NMR (376 MHz, $CDCl_3$) δ -63.1.

Melting Point 62-63 ºC.

HRMS (ESI$^+$) calcd for $C_{10}H_4D_6NF_6$ $[M+H]^+$: 264.1089, found 264.1084.

Procedure for the preparation of *N,N,N*-tris(trideuteromethyl)-3,5-bis(trifluoromethyl)anilinium triflate 2e

N,*N*-Bis(trideuteromethyl)-3,5-bis(trifluoromethyl)aniline (2.63 g, 10.0 mmol) was added to a round bottom flask and 3 evacuation-refill cycles were performed with nitrogen. Anhydrous dichloromethane (20 mL) was subsequently added, and the resulting solution stirred. Trideuteromethyl trifluoromethylsulfonate (1.41 mL, 12.5 mmol, 1.25 equiv) was added dropwise and the reaction stirred at room-temperature for 24 hours. The observed white precipitate was filtered, washed with excess Et_2O (40 mL) and dried in a vacuum desiccator. Product **2e** was afforded as a colourless solid (4.10 g, 95 %).

^{1}H NMR (400 MHz, $(CD_3)_2CO$) δ δ 8.83 (s, 2H), 8.39 (s, 1H).

^{2}H NMR (77 MHz, $(CD_3)_2CO$) δ 4.81.

^{13}C NMR (126 MHz, $(CD_3)_2CO$) δ 149.0, 133.7 (q, *J* = 34.5 Hz), 125.1 (sept., *J* = 3.6 Hz), 123.3 (q, *J* = 3.6 Hz), 123.2 (q, *J* = 273.0 Hz), 121.7 (q, *J* = 320.9 Hz), 56.7 (sept, *J* = 22.1 Hz).

^{19}F NMR (376 MHz, $(CD_3)_2CO$) δ -63.2, -79.0

Melting Point 223-224 °C.

HRMS (ESI^+) calcd for $C_{11}H_3D_9NF_6$ $[M+H]^+$: 281.1433, found 281.1422.

Preparation of starting materials

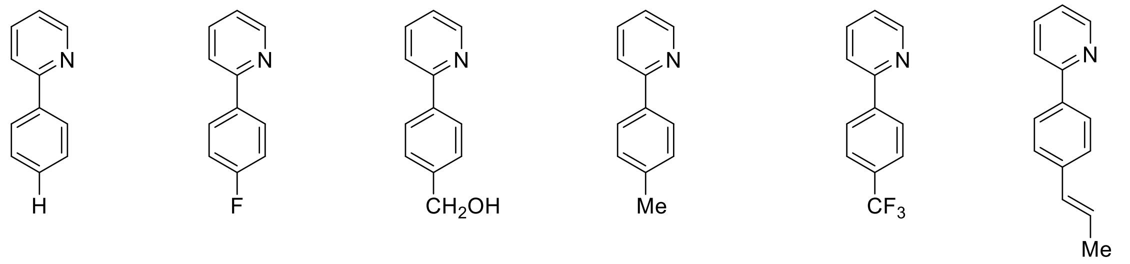

1a **1b** **1c** **1d** **1e** **1f**

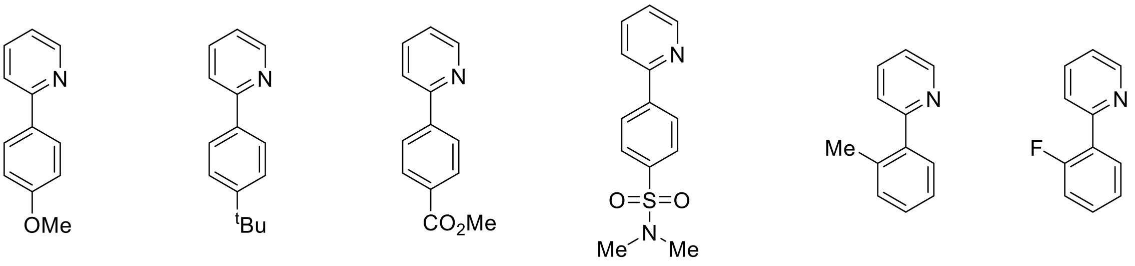

1g **1h** **1i** **1j** **3aa** **1k**

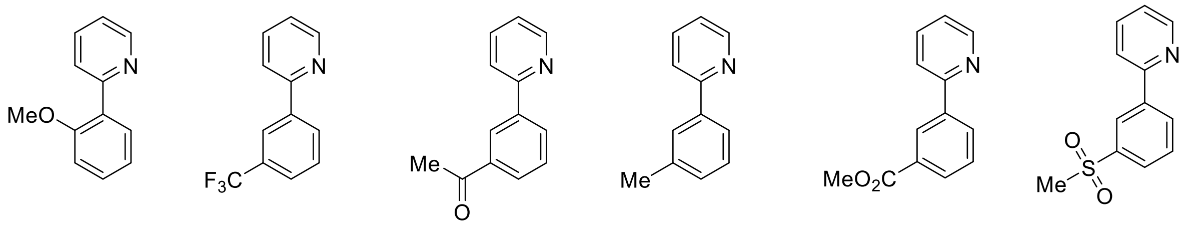

1l **1m** **1n** **1o** **1p** **1q**

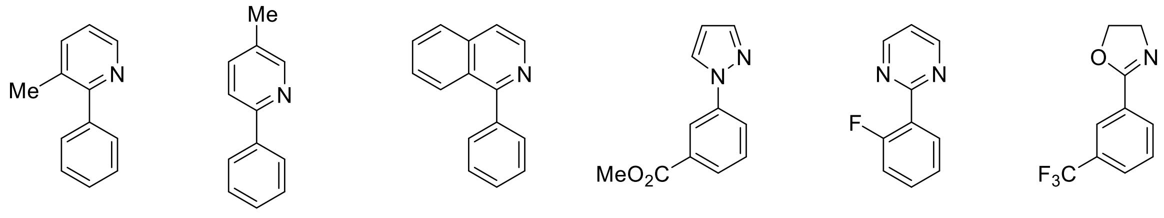

1r **1s** **1t** **1u** **1v** **1w**

The following 2-phenylpyridines were either bought and used as sold from Fluorochem (**1a** and **1r)** or prepared according to known literature procedures. Compounds **1b,**[5] **1d,**[6] **1e,**[7] **1g,**[6] **1h,**[8] **1i,**[6] **3aa,**[2] **1k,**[6] **1l,**[9] **1m,**[10] **1n,**[10] **1o,**[2] **1p,**[11] **1s,**[2] **1t**[12], and **1v** [13] and were prepared by Suzuki-Miyaura coupling reactions between 2-bromopyridine and the corresponding boronic acids, under various conditions (referenced). For **1c**, the corresponding aldehyde was prepared by Suzuki-Miyaura coupling,[2] which was subsequently reduced to the alcohol.[14] This aldehyde was also used in a Wittig reaction to synthesise **1f.**[15] **1u** was prepared by a copper-catalyzed

amination using the corresponding aryl iodide.[16] **1w** was prepared by a copper-NHC catalyzed reaction between 2-aminoethanol and the 3-(trifluoromethyl)benzonitrile.[17] Compounds **1j** and **1q** are novel syntheses and are outlined below.

***N,N*-Dimethyl-4-(pyridine-2-yl)benzenesulfonamide 1j**

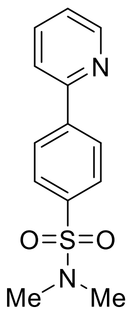

1j

The title compound was synthesised as outlined in **General Procedure F** using (4-(*N,N*-dimethylsulfamoyl)phenyl)boronic acid (2.3 g, 10 mmol) . Purification using flash column chromatography (70:30 Hexane:EtOAc) afforded **1j** as a colourless solid (2.0 g, 75 %).

^{1}H NMR (400 MHz, $CDCl_3$) δ 8.74 (ddd, *J* = 4.8, 1.7, 1.0 Hz, 1H), 8.17 (app. dt, *J* = 8.6, 1.9 Hz, 2H), 7.88 (app. dt, *J* = 8.6, 1.9 Hz, 2H), 7.76-7.85 (m, 2H), 7.33 (ddd, *J* = 7.0, 4.8, 1.6 Hz, 1H), 2.73 (s, 6H).

^{13}C NMR (126 MHz, $CDCl_3$) δ 155.7, 150.2, 143.7, 137.2, 135.7, 128.4, 127.6, 123.4, 121.2, 38.1.

Melting Point 95-96 ºC

HRMS (ESI^+) calcd for $C_{13}H_{15}O_2N_2S$ $[M+H]^+$: 263.0849. Found: 263.0848.

2-(3-(methylsulfonyl)phenylpyridine 1q

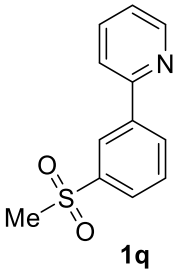

1q

The title compound was synthesised as outlined in **General Procedure F** using ((3-(methylsulfonyl)phenyl)boronic acid (2.0 g, 10 mmol) . Purification using flash column chromatography (80:20 Hexane:EtOAc) afforded **1q** as a colourless solid (1.9 g, 80%).

^{1}H NMR (400 MHz, $CDCl_3$) δ 8.73 (ddd, *J* = 4.8, 1.6, 1.1 Hz, 1H), 8.58 (app. t, *J* = 1.7 Hz, 1H), 8.32 (ddd, *J* = 7.8, 1.7, 1.1 Hz, 1H), 7.99 (ddd, *J* = 7.8, 1.8, 1.1 Hz, 1H), 7.76-7.86 (m, 2H), 7.69 (app. t, *J* = 7.9 Hz, 1H), 7.32 (ddd, *J* = 6.2, 4.8, 2.4 Hz, 1H), 3.12 (s, 3H).

^{13}C NMR (101 MHz, $CDCl_3$) δ 155.3, 150.2, 141.4, 141.1, 137.3, 132.1, 130.0, 127.7, 126.0, 123.3, 120.9, 44.7.

Spectroscopic data match those previously reported.[18]

(*E*)-*N,N*-dimethyl-4-((1-(3-(trifluoromethyl)phenyl)ethylidene)amino)aniline 5a

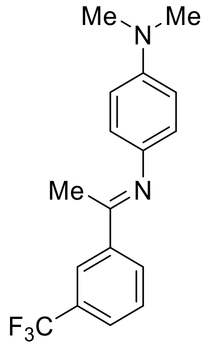

5a

The title compound was synthesised as outlined in **General Procedure G** using 3-(trifluoromethyl)acetophenone (0.94 g, 5.0 mmol) and *N,N*-Dimethyl-*p*-phenylenediamine (0.82 g, 6.0 mmol, 1.2 equiv). Crystallization from pentane:Et_2O (5:1) afforded **5a** as a yellow solid (0.95 g, 62%). Spectroscopic data match those previously reported.[19]

^{1}H NMR (400 MHz, $CDCl_3$) δ 8.25 (s, 1H), 8.15 (d, *J* = 7.8 Hz, 1H), 7.69 (d, *J* = 7.8 Hz, 1H), 7.55 (t, *J* = 7.8 Hz, 1H), 6.79 (s, 4H), 2.96 (s, 6H), 2.32 (s, 3H).

^{13}C NMR (101 MHz, $CDCl_3$) 163.3, 147.9, 130.9 (d, *J* = 32.3 Hz), 130.4, 128.9, 126.6 (d, *J* = 3.7 Hz), 124.3 (d, *J* = 272.5 Hz), 124.1 (d, *J* = 3.8 Hz), 121.3, 113.4, 41.2, 17.4.

(*E*)-*N,N*-dimethyl-4-((2-(trifluoromethyl)benzylidene)amino)aniline 5b

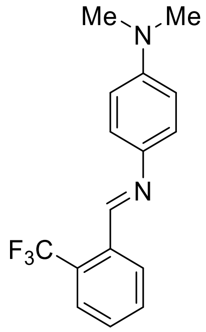

5b

The title compound was synthesised as outlined in **General Procedure H** using 2-(trifluoromethyl)benzaldehyde (0.87 g, 5.0 mmol) and *N,N*-Dimethyl-*p*-phenylenediamine (0.68 g, 5.0 mmol, 1 equiv). Crystallization from pentane:Et_2O (5:1) afforded **5b** as an orange solid (1.3 g, 86%).

1H NMR (500 MHz, CDCl3) δ 8.89 (s, 1H), 8.46 (d, J = 7.7 Hz, 1H), 7.71 (d, J = 7.7 Hz, 1H), 7.63 (app. t, J = 7.7 Hz, 1H), 7.50 (app. t, J = 7.7 Hz, 1H), 7.32 (d, J = 8.8 Hz, 2H), 6.78 (d, J = 8.8 Hz, 2H), 3.01 (s, 6H).

13C NMR (126 MHz, CDCl3) δ 151.4 (q, J = 1.4 Hz), 150.1, 140.4, 135.1 (q, J = 1.3 Hz), 132.1, 129.8, 129.2 (q, J = 30.8 Hz), 128.1, 125.8 (q, J = 5.7 Hz), 124.5 (q, J = 274.1 Hz), 122.8, 112.8, 40.7.

19F NMR (471 MHz, CDCl3) δ -57.1.

Melting Point 76-78 ^{o}C

HRMS (ESI^+) calcd for $C_{16}H_{16}N_2F_3$ $[M+H]^+$: 293.1260, found 293.1265.

(*E*)-*N,N*-dimethyl-4-((naphthalen-1-ylmethylene)amino)aniline 5c

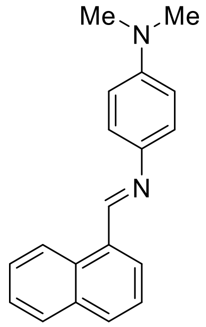

5c

The title compound was synthesised as outlined in **General Procedure H** using 1-naphthaldehyde (0.78 g, 5.0 mmol) and *N,N*-Dimethyl-*p*-phenylenediamine (0.68 g, 5.0 mmol, 1 equiv). Crystallization from pentane:Et_2O (5:1) afforded **5c** as an light brown solid (1.1 g, 73%).

^{1}H NMR (400 MHz, $CDCl_3$) δ 9.20 (s, 1H), 9.01 (d, *J* = 8.4 Hz, 1H), 8.12 (d, *J* = 7.2 Hz, 1H), 7.93 (t, *J* = 8.4 Hz, 2H), 7.65 – 7.52 (m, 3H), 7.37 (d, *J* = 8.8 Hz, 2H), 6.82 (d, *J* = 8.8 Hz, 2H), 3.01 (s, 6H).

^{13}C NMR (126 MHz, CDCl3) δ 155.5, 149.7, 141.7, 134.0, 132.3, 131.6, 131.1, 128.8, 128.7, 127.2, 126.1, 125.5, 124.3, 122.4, 113.1, 40.9.

Melting Point 74-76 °C

HRMS (ESI$^+$) calcd for $C_{19}H_{19}N_2$ $[M+H]^+$: 275.1543, found 275.1552.

2-(6-methyl-2-(p-tolyl)imidazo[1,2-a]pyridin-3-yl)-1-(piperidin-1-yl)ethan-1-one

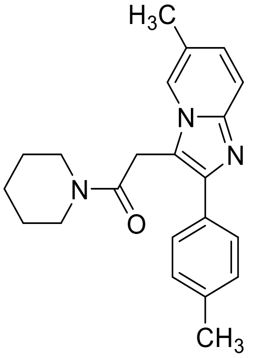

The title compound was synthesised from zolpidic acid in two steps:

Step I. A 50 mL round-bottom flask was charged with zolpidic acid (1.0 g, 3.6 mmol, 1.0 equiv), dry DCM (11 mL) and 2 drops of DMF. The suspension was cooled to 0 °C and stirred for 5 minutes. Then oxalyl chloride (0.4 mL, 1.3 equiv) was added dropwise to the reaction mixture and stirred at room temperature for 12 hours. The resulting mixture was concentrated under reduced pressure to afford acyl chloride as a light brown solid which was used directly without further purification for the next step.

Step II. To a solution of piperidine (0.46 mL, 1.30 equiv) and triethyl amine (0.76 mL, 1.50 equiv) in dry DCM (8 mL) crude acyl chloride (1 equiv) in DCM (3 mL) was added dropwise at 0 °C and the reaction mixture was stirred at room temperature for 12 hours. Then water (20 mL) was added to the mixture and extracted with DCM (3 x 30 mL). The combined of the organic layers was washed withsaturated aqueous $NaHCO_3$ (30 mL) solution followed by water (30 mL). The organic layer was dried over $MgSO_4$ and concentrated under reduced pressure. Purification using flash column chromatography (30:70 Hexane:EtOAc) afforded **7c** as a light brown solid (938 mg, 75%).

^{1}H NMR (400 MHz, $CDCl_3$) δ 8.06 (s, 1H), 7.57 – 7.50 (m, 3H), 7.28-7.24 (m, 2H), 7.03 (dd, J = 9.2, 1.3 Hz, 1H), 4.09 (s, 2H), 3.56 – 3.48 (m, 2H), 3.25 – 3.16 (m, 2H), 2.40 (s, 3H), 2.34 (s, 3H), 1.56 – 1.49 (m, 2H), 1.48 – 1.40 (m, 2H), 1.23 – 1.15 (m, 2H).

^{13}C NMR (101 MHz, $CDCl_3$) δ 166.6, 144.3, 143.8, 137.6, 132.0, 129.4, 128.5, 127.6, 122.4, 121.8, 116.7, 113.9, 47.2, 43.4, 30.6, 26.5, 25.8, 24.4, 21.4, 18.6.

Spectroscopic data match those previously reported.[20]

(*R*)-2,8-dimethyl-2-((4*R*,8*R*)-4,8,12-trimethyltridecyl)chroman-6-yl 3-(pyridin-2-yl)benzoate 7e

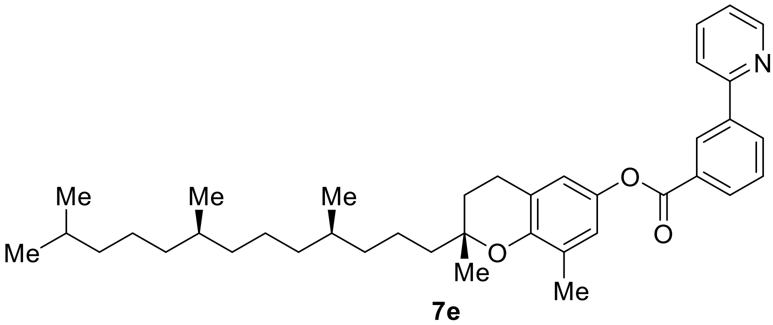

7e

The title compound was synthesised as outlined in **General Procedure E** using δ-tocopherol (1.0 g, 2.5 mmol, 1 equiv). Purification using flash column chromatography (90:10 Hexane:EtOAc) afforded **7e** as a colourless oil (1.3 g, 85%).

^{1}H NMR (400 MHz, $CDCl_3$) δ 8.77 (app. t, *J* = 1.6 Hz, 1H), 8.75 – 8.72 (m, 1H), 8.32 – 8.29 (m, 1H), 8.25 – 8.21 (m, 1H), 7.84 – 7.76 (m, 2H), 7.62 (t, *J* = 7.8 Hz, 1H), 7.28 (ddd, *J* = 6.8, 4.8, 1.8 Hz, 1H), 6.83 (d, *J* = 2.5 Hz, 1H), 6.78 (d, *J* = 2.7 Hz, 1H), 2.83 – 2.73 (m, 2H), 2.19 (s, 3H), 1.89 – 1.71 (m, 2H), 1.65 – 1.33 (m, 9H), 1.33 – 1.01 (m, 15H), 0.93 – 0.82 (m, 12H).

^{13}C NMR (101 MHz, $CDCl_3$) δ 165.8, 156.5, 150.0, 150.0, 142.8, 140.0, 137.0, 131.9, 130.6 (2C), 129.2, 128.5, 127.5, 122.7, 121.4, 121.2, 120.8, 119.3, 76.3, 40.3, 39.5, 37.6, 37.6(2C), 37.4, 32.9, 32.8, 31.2, 28.1, 24.9, 24.6, 24.4, 22.9, 22.8, 22.6, 21.1, 19.9, 19.8, 16.3.

HRMS (ESI^+) calcd for $C_{39}H_{54}NO_3$ $[M+H]^+$: 584.4104, found 584.4100.

(8*S*,9*R*,13*R*,14*R*)-13-methyl-17-oxo-7,8,9,11,12,13,14,15,16,17-decahydro-6H cyclopenta[a]phenanthren-3-yl 3-(pyridin-2-yl)benzoate 7f

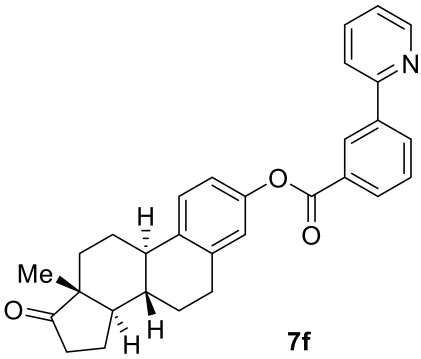

7f

The title compound was synthesised as outlined in **General Procedure E** using estrone (679 mg, 2.50 mmol, 1 equiv). Purification using flash column chromatography (80:20 Hexane:EtOAc) afforded **7f** as a colourless solid (900 mg, 80 %).

^{1}H NMR (400 MHz, CDCl$_3$) δ 8.79 (t, *J* = 1.6 Hz, 1H), 8.76 – 8.70 (m, 1H), 8.34 – 8.28 (m, 1H), 8.27 – 8.20 (m, 1H), 7.86 – 7.77 (m, 2H), 7.63 (t, *J* = 7.8 Hz, 1H), 7.35 (d, *J* = 8.4 Hz, 1H), 7.29 (ddd, *J* = 6.8, 4.8, 2.0 Hz, 1H), 7.01 (dd, *J* = 8.4, 2.5 Hz, 1H), 6.97 (d, *J* = 2.4 Hz, 1H), 3.00 – 2.90 (m, 2H), 2.52 (dd, *J* = 18.9, 8.6 Hz, 1H), 2.48 – 2.41 (m, 1H), 2.38 – 2.29 (m, 1H), 2.21 – 1.95 (m, 4H), 1.70 – 1.59 (m, 3H), 1.56 – 1.42 (m, 3H), 0.93 (s, 3H).

^{13}C NMR (101 MHz, CDCl$_3$) δ 220.9, 165.5, 156.4, 150.0, 149.0, 140.1, 138.2, 137.6, 137.1, 132.1, 130.7, 130.4, 129.2, 128.6, 126.6, 122.8, 121.9, 120.8, 119.0, 50.6, 48.1, 44.3, 38.1, 36.0, 31.7, 29.6, 26.5, 25.9, 21.7, 14.0.

Melting Point 156-158 ºC.

HRMS (ESI$^+$) calcd for $C_{30}H_{30}NO_3$ [M+H]$^+$: 452.2226, found 452.2235.

2-methoxy-4-((*E*)-3-oxo-3-(((2a*R*,3*R*,5b*S*,7a*R*,9*S*,11a*R*,12a*S*)-2a,5b,8,8-tetramethyl-3-((*R*)-6-methyl-5-methyleneheptan-2-yl)tetradecahydro-1H,12H-cyclopenta[a]cyclopropa[e]phenanthren-9-yl)oxy)prop-1-en-1-yl)phenyl 3-(pyridin-2-yl)benzoate 7g

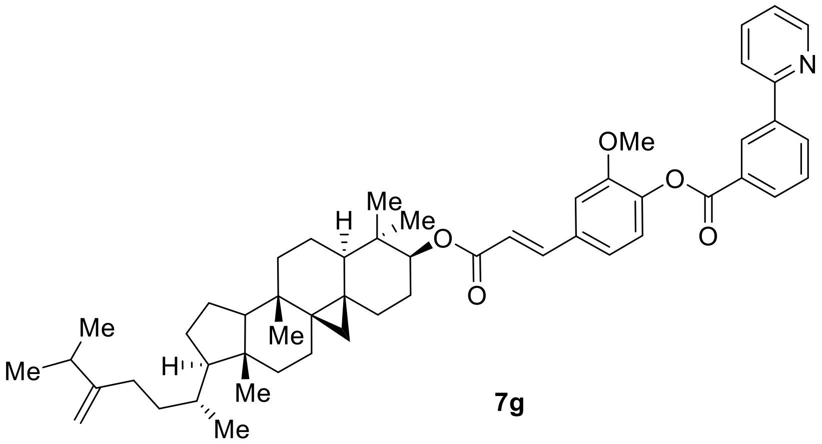

7g

The title compound was synthesised as outlined in **General Procedure E** using γ-oryzanol (1.5 g, 2.5 mmol, 1 equiv). Purification using flash column chromatography (85:15 Hexane:EtOAc) afforded **7g** as a colourless solid (1.1 g, 55 %).

^{1}H NMR (500 MHz, CDCl$_3$) δ 8.81 (s, 1H), 8.73 (d, *J* = 4.3 Hz, 1H), 8.33 (d, *J* = 7.7 Hz, 1H), 8.26 (d, *J* = 7.7 Hz, 1H), 7.88 – 7.75 (m, 2H), 7.66 (d, *J* = 15.8 Hz, 1H), 7.66 – 7.59 (m, 1H), 7.30 – 7.27 (m, 1H), 7.18 (d, *J* = 11.5 Hz, 3H), 6.43 (d, *J* = 15.9 Hz, 1H), 4-76-4.65 (m,2H), 3.86 (s, 3H), 2.28 – 2.20 (m, 1H), 2.18 – 2.07 (m, 1H), 2.05 – 1.97 (m, 1H), 1.97 – 1.84 (m, 3H), 1.75 – 1.51 (m, 10H), 1.50 – 1.23 (m, 8H), 1.21 – 0.75 (m, 26H), 0.61 (d, *J* = 3.2 Hz, 1H), 0.38 (d, *J* = 3.6 Hz, 1H).

^{13}C NMR (101 MHz, CDCl$_3$) δ 166.7, 164.4, 156.9, 156.2, 151.6, 149.9, 143.6, 141.6, 140.0, 137.0, 133.6, 132.1, 130.7, 129.7, 129.1, 128.7, 123.4, 122.7, 121.3, 120.7, 119.2, 111.3, 106.0, 80.9, 56.0, 52.3, 48.8, 47.9, 47.2, 45.3, 39.7, 36.1, 35.5, 35.0, 33.8, 32.9, 31.7, 31.3, 29.8, 28.1, 26.9, 26.5, 26.0, 25.8, 25.5(2C), 22.0, 21.9, 21.0, 20.2, 19.3, 18.3, 18.0, 15.4.

Melting Point 142-144 ⁰C.

HRMS (ESI$^+$) calcd for $C_{53}H_{68}NO_5$ [M+H]$^+$: 798.5097, found 798.5138.

5. Characterization data for mono-methylated products.

2-(*o*-tolyl)pyridine 3aa

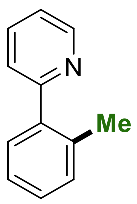

3aa

The title compound was synthesised as outlined in **General Procedure A**, using 2-phenylpyridine **1a** (47 mg, 0.30 mmol). Purification using flash column chromatography (90:10 Hexane:Et_2O) afforded **3aa** as a pale-yellow oil (44 mg, 87 %). Spectroscopic data match those previously reported.[9]

^{1}H NMR (500 MHz, $CDCl_3$) δ 8.68 (dd, *J* = 4.9, 1.8 Hz, 1H), 7.71 (td, *J* = 7.8, 1.6 Hz, 1H), 7.37 (d, *J* = 7.7 Hz, 2H), 7.18 – 7.31 (m, 4H), 2.34 (s, 3H).

^{13}C NMR (126 MHz, $CDCl_3$) δ 160.2 149.4, 140.6, 136.2, 135.9, 130.9, 129.7, 128.4, 126.0, 124.2, 121.7, 20.4.

2-(2,6-dimethylphenyl)pyridine 4aa

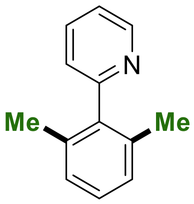

4aa

The title compound was synthesised as a by-product of the reaction outlined in **General Procedure A**, using 2-phenylpyridine **3aa** (51 mg, 0.30 mmol). Purification using flash column chromatography (90:10 Hexane:Et_2O) afforded **4aa** as a yellow-brown oil (2.7 mg, 5 %). Spectroscopic data match those previously reported.[9]

^{1}H NMR (400 MHz, $CDCl_3$) δ 8.73 (d, *J* = 4.9 Hz, 1H), 7.76 (app. td, *J* = 7.7, 1.6 Hz, 1H), 7.16 – 7.30 (m, 3H), 7.11 (d, *J* = 7.5 Hz, 2H), 2.05 (s, 6H).

^{13}C NMR (101 MHz, $CDCl_3$) δ 160.1, 149.8, 140.6, 136.4, 135.9, 128.0, 127.6, 124.6, 121.8, 20.3.

2-(2-methyl-4-fluorophenyl)pyridine 3ba

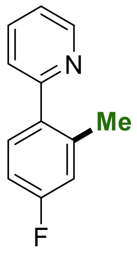

3ba

The title compound was synthesised as outlined in **General Procedure A**, using 2-(4-fluorophenyl)pyridine **1b** (52 mg, 0.30 mmol). Purification using flash column chromatography (90:10 Hexane:Et_2O) afforded **3ba** as a yellow oil (41 mg, 73 %). Spectroscopic data match those previously reported.[21]

^{1}H NMR (400 MHz, $CDCl_3$) δ 8.68 (ddd, J = 4.9, 1.7, 0.9 Hz, 1H), 7.73 (td, J = 7.7, 1.8 Hz, 1H), 7.32–7.40 (m, 2H), 7.24 (ddd, J = 7.6, 4.9, 1.1 Hz, 1H), 7.02 – 6.91 (m, 2H), 2.36 (s, 3H).

^{13}C NMR (101 MHz, $CDCl_3$) δ 163.9 (d, J = 246.3 Hz), 159.2, 149.4, 138.5 (d, J = 8.2 Hz), 136.7 (d, J = 3.0 Hz), 136.3, 131.4 (d, J = 8.5 Hz), 124.2, 121.8, 117.3 (d, J = 21.1 Hz), 112.8 (d, J = 21.2 Hz), 20.5 (d, J = 1.5 Hz).

^{19}F NMR (376 MHz, $CDCl_3$) δ -114.7

2-(2,6-dimethyl-4-fluorophenyl)pyridine 4ba

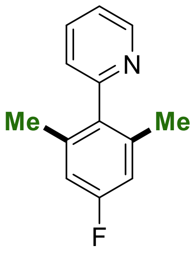

4ba

The title compound was synthesised as a by-product of the reaction outlined in **General Procedure A**, using 2-(4-fluorophenyl)pyridine **1b** (52 mg, 0.30 mmol). Purification using flash column chromatography (90:10 Hexane:Et_2O) afforded **4ba** as a yellow oil (4.8 mg, 8 %).

^{1}H NMR (500 MHz, $CDCl_3$) δ 8.72 (ddd, *J* = 4.9, 1.9, 0.9 Hz, 1H), 7.77 (td, *J* = 7.7, 1.8 Hz, 1H), 7.27 (ddd, *J* = 7.6, 4.9, 1.2 Hz, 1H), 7.21 (dt, *J* = 7.8, 1.1 Hz, 1H), 6.81 (dt, *J* = 9.5, 0.6 Hz, 2H), 2.03 (s, 6H).

^{13}C NMR (126 MHz, $CDCl_3$) δ 163.2, 160.2 (d, *J* = 265.0 Hz), 149.8, 138.5 (d, *J* = 8.3 Hz), 136.7, 136.4, 125.0, 122.1, 114.2 (d, *J* = 21.0 Hz), 20.5 (d, *J* = 1.5 Hz).

^{19}F NMR (471 MHz, $CDCl_3$) δ 115.6.

HRMS (ESI^+) calcd for $C_{13}H_{13}NF$ $[M+H]^+$: 202.1027, found 202.1019.

(3-methyl-4-(pyridine-2-yl)phenyl)methanol 3ca

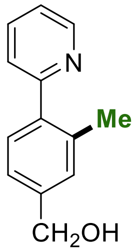

3ca

The title compound was synthesised as outlined in **General Procedure A**, using (4-(pyridin-2-yl)phenyl)methanol **1c** (56 mg, 0.30 mmol). Purification using flash column chromatography (20:1 Hexane:EtOAc) afforded **3ca** as a pale-yellow oil (41 mg, 68 %).

^{1}H NMR (400 MHz, $CDCl_3$) δ 8.69 (ddd, *J* = 4.9. 1.7, 0.9 Hz, 1H), 7.75 (td, *J* = 7.7, 1.8 Hz, 1H), 7.38 (app. t, *J* = 8.0 Hz, 2H), 7.16 – 7.32 (m, 3H), 4.69 (s, 2H), 2.36 (s, 3H). *O-**H** peak not observed due to H/D exchange in $CDCl_3$.*

^{13}C NMR (101 MHz, $CDCl_3$) δ 159.9, 149.3, 141.3, 139.3, 136.4, 136.1, 130.0, 129.3, 124.4, 124.3, 121.8, 65.0, 20.4.

IR ν_{max} (neat/cm^{-1}): 3255 (br), 2920, 2859, 1588, 1561, 1467, 1428, 1409, 1054, 1025, 996, 830, 786, 748, 721.

HRMS (ESI^{+}) calcd for $C_{13}H_{14}NO$ $[M+H]^{+}$: 200.1070, found 200.1072.

2-(2,4-dimethylphenyl)pyridine 3da

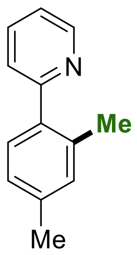

3da

The title compound was synthesised as outlined in **General Procedure A** using 2-(*p*-tolyl)pyridine **1d** (51 mg, 0.30 mmol). Purification using flash column chromatography (90:10 Hexane:Et_2O) afforded **3da** as a yellow oil (41 mg, 74 %). Spectroscopic data match those previously reported.[22]

^{1}H NMR (400 MHz, $CDCl_3$) δ 8.69 (ddd, *J* = 4.9, 1.7, 0.9 Hz, 1H), 7.72 (td, *J* = 7.7, 1.8 Hz, 1H), 7.38 (d, *J* = 7.9 Hz, 1H), 7.31 (d, *J* = 7.6 Hz, 1H), 7.22 (ddd, *J* = 7.5, 4.9, 1.1 Hz, 1H), 7.10 (s, 1H), 7.09 (d, *J* = 8.8 Hz, 1H), 2.37 (s, 3H), 2.35 (s, 3H).

^{13}C NMR (101 MHz, $CDCl_3$) δ 160.2, 149.3, 138.1, 137.8, 136.1, 135.7, 131.6, 129.8, 126.7, 124.2, 121.5, 21.3, 20.4.

2-(2-methyl-4-trifluoromethylphenyl)pyridine 3ea

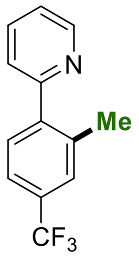

3ea

The title compound was synthesised as outlined in **General Procedure A**, using 2-(4-(trifluoromethyl)phenyl)pyridine **1e** (67 mg, 0.30 mmol). Purification using flash column chromatography (90:10 Hexane:Et_2O) afforded **3ea** as a yellow oil (43 mg, 60 %). Spectroscopic data match those previously reported.[22]

^{1}H NMR (400 MHz, $CDCl_3$) δ 8.72 (ddd, J = 4.9, 1.7, 0.9 Hz, 1H), 7.78 (td, J = 7.7, 1.8 Hz, 1H), 7.47-7.57 (m, 3H), 7.40 (dt, J = 7.9. 1.0 Hz, 1H), 7.29 (ddd, J = 7.6, 4.9, 1.1 Hz, 1H), 2.41 (s, 3H).

^{13}C NMR (101 MHz, $CDCl_3$) δ 158.8, 149.6, 143.9, 136.9, 136.5, 130.4 (q, J = 32.2 Hz), 130.2, 127.6 (q, J = 3.8 Hz), 124.3 (q, J = 272.1 Hz), 124.1, 122.8 (q, J = 3.8 Hz), 122.4, 20.5.

^{19}F NMR (471 MHz, $CDCl_3$) δ -62.6.

2-(2,6-dimethyl-4-trifluoromethylphenyl)pyridine 4ea

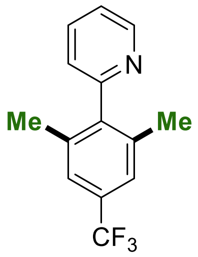

4ea

The title compound was synthesised as a by-product of the reaction outlined in **General Procedure A**, using 2-(4-(trifluoromethyl)phenyl)pyridine **1e** (67 mg, 0.30 mmol). Purification using flash column chromatography (90:10 Hexane:Et_2O) afforded **4ea** as a pale-yellow oil (4.5 mg, 6 %).

^{1}H NMR (400 MHz, $CDCl_3$) δ 8.74 (d, J = 4.7 Hz, 1H), 7.79 (td, J = 7.7, 1.7 Hz, 1H), 7.36 (s, 2H), 7.30 (dd, J = 7.5, 5.0 Hz, 1H), 7.21 (d, J = 7.8 Hz, 1H), 2.09 (s, 6H).

^{13}C NMR (101 MHz, $CDCl_3$) δ 158.8, 150.1, 143.9, 137.0, 136.7, 130.1 (q, J = 31.9 Hz), 124.39 (q, J = 3.7 Hz), 124.36 (q, J = 272.2 Hz), 124.2, 122.3, 20.4.

^{19}F NMR (376 MHz, $CDCl_3$) δ -62.6

HRMS (ESI$^+$) calcd for $C_{14}H_{13}NF_3$ $[M+H]^+$: 252.0995, found 252.0987.

(*E*)-2-(2-methyl-4-(prop-1-en-1-yl)phenyl)pyridine 3fa

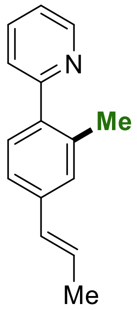

3fa

The title compound was synthesised as outlined in **General Procedure A**, using (*E*)-2-(4-(prop-1-en-1-yl)phenyl)pyridine **1f** (59 mg, 0.30 mmol). Purification using flash column chromatography (90:10 Hexane:Et_2O) afforded **3fa** as a yellow-brown oil (41 mg, 66 %).

^{1}H NMR (400 MHz, $CDCl_3$) δ 8.71 (ddd, *J* = 4.9, 1.7, 0.9 Hz, 1H), 7.74 (td, *J* = 7.7, 1.8 Hz, 1H), 7.41 (d, *J* = 7.9 Hz, 1H), 7.37 (d, *J* = 8.5 Hz, 1H), 7.20–7.33 (m, 3H), 6.44 (dd, *J* = 15.8, 1.4 Hz, 1H), 6.31 (dq, *J* = 15.8, 6.4 Hz, 1H), 2.39 (s, 3H), 1.92 (dd, *J* = 6.4, 1.4 Hz, 3H).

^{13}C NMR (101 MHz, $CDCl_3$) δ 160.0, 149.3, 139.0, 138.0, 136.1, 135.9, 130.8, 130.0, 128.5, 126.2, 124.2, 123.4, 121.6, 20.5, 18.7.

HRMS (ESI$^+$) calcd for $C_{15}H_{16}N$ $[M+H]^+$: 210.1277, found 210.1277.

2-(2-methyl-4-methoxyphenyl)pyridine 3ga

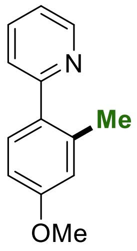

3ga

The title compound was synthesised as outlined in **General Procedure A**, using 2-(4-methoxyphenyl)pyridine **1g** (56 mg, 0.30 mmol). Purification using flash column chromatography (90:10 Hexane:Et_2O) afforded **3ga** as a yellow oil (42 mg, 71 %). Spectroscopic data match those previously reported.[22]

^{1}H NMR (400 MHz, $CDCl_3$) δ 8.67 (d, *J* = 4.9 Hz, 1H), 7.71 (td, *J* = 7.7, 1.8 Hz, 1H), 7.31-7.41 (m, 2H), 7.20 (ddd, *J* = 7.5, 4.9, 1.0 Hz, 1H), 6.77 – 6.87 (m, 2H), 3.83 (s, 3H), 2.38 (s, 3H).

^{13}C NMR (101 MHz, $CDCl_3$) δ 159.8, 159.5, 149.2, 137.4, 136.0, 133.3, 131.0, 124.1, 121.2, 116.2, 111.3, 55.3, 20.7.

2-(2,6-dimethyl-4-methoxyphenyl)pyridine 4ga

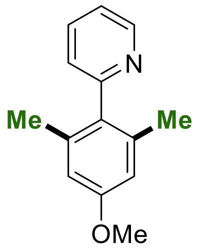

4ga

The title compound was synthesised as a by-product of the reaction outlined in **General Procedure A**, using 2-(4-methoxyphenyl)pyridine **1g** (56 mg, 0.30 mmol). Purification using flash column chromatography (90:10 Hexane:Et_2O) afforded **4ga** as a colourless oil (3.2 mg, 5 %). Spectroscopic data match those previously reported.[23]

^{1}H NMR (400 MHz, $CDCl_3$) δ 8.70 (d, J = 4.9 Hz, 1H), 7.73 (td, J = 7.7, 1.6 Hz, 1H), 7.13-7.29 (m, 2H), 6.65 (s, 2H), 3.81 (s, 3H), 2.03 (s, 6H).

^{13}C NMR (101 MHz, $CDCl_3$) δ 159.9, 159.0, 149.7, 137.4, 136.3, 133.6, 125.1, 121.6, 113.0, 55.3, 20.6.

2-(2-methyl-4-*tert*-butylphenyl)pyridine 3ha

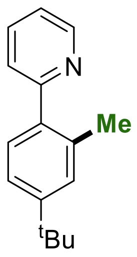

3ha

The title compound was synthesised as outlined in **General Procedure A**, using 2-(4-(*tert*-butyl)phenyl)pyridine **1h** (63 mg, 0.30 mmol). Purification using flash column chromatography (90:10 Hexane:Et_2O) afforded **3ha** as a yellow oil (47 mg, 70 %).

^{1}H NMR (400 MHz, $CDCl_3$) δ 8.68 (ddd, *J* = 4.9, 1.7, 0.9 Hz, 1H), 7.72 (td, *J* = 7.7, 1.8 Hz, 1H), 7.41 (dt, *J* = 7.9, 1.9 Hz, 1H), 7.32-7.38 (m, 1H), 7.27-7.32 (m, 2H), 7.22 (ddd, *J* = 7.6, 4.9, 1.1 Hz, 1H), 2.38 (s, 3H), 1.35 (s, 9H).

^{13}C NMR (126 MHz, $CDCl_3$) δ 160.2, 151.3, 149.3, 137.8, 136.1, 135.3, 129.5, 127.9, 124.2, 123.0, 121.5, 34.6, 31.5, 20.7.

HRMS (ESI$^+$) calcd for $C_{16}H_{20}N$ $[M+H]^+$: 226.1590, found 226.1592.

Methyl 3-methyl-4-(pyridin-2-yl)benzoate 3ia

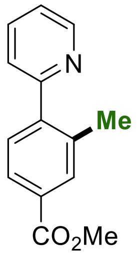

3ia

The title compound was synthesised as outlined in **General Procedure A**, using methyl 4-(pyridin-2-yl)benzoate **1i** (64 mg, 0.30 mmol). Purification using flash column chromatography (80:10:10 Hexane:EtOAc:DCM) afforded **3ia** as a yellow oil (50 mg, 78 %). Spectroscopic data match those previously reported.[23]

^{1}H NMR (400 MHz, $CDCl_3$) δ 8.71 (d, *J* = 4.9 Hz, 1H), 7.96 (s, 1H), 7.93 (dd, *J* = 8.0, 1.6 Hz, 1H), 7.76 (td, *J* = 7.7, 1.8 Hz, 1H), 7.46 (d, *J* = 7.9 Hz, 1H), 7.40 (dt, *J* = 7.8, 1.0 Hz, 1H), 7.28 (ddd, *J* = 7.6, 4.9, 1.1, 1H), 3.92 (s, 3H), 2.40 (s, 3H).

^{13}C NMR (126 MHz, $CDCl_3$) δ 167.2, 159.1, 149.5, 144.8, 136.4, 136.3, 132.0, 129.88, 129.86, 127.2, 124.2, 122.3, 52.2, 20.4.

***N,N*-Dimethyl-4-(pyridine-2-yl)benzenesulfonamide 3ja**

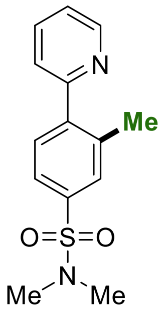

3ja

The title compound was synthesised as outlined in **General Procedure A**, using *N,N*-dimethyl-4-(pyridin-2-yl)benzenesulfonamide **1j** (79 mg, 0.30 mmol). Purification using flash column chromatography (65:35 Hexane:EtOAc) afforded **3ia** as a yellow oil (71 mg, 87%).

^{1}H NMR (500 MHz, $CDCl_3$) δ 8.73 (ddd, *J* = 4.9, 1.7, 0.9 Hz, 1H), 7.82 (td, *J* = 7.7, 1.8 Hz, 1H), 7.63-7.73 (m, 2H), 7.55 (d, *J* = 7.9 Hz, 1H), 7.43 (d, *J* = 7.9 Hz, 1H), 7.33 (ddd, *J* = 7.6, 4.9, 1.1 Hz, 1H), 2.73 (s, 6H), 2.43 (s, 3H).

^{13}C NMR (126 MHz, $CDCl_3$) δ 158.4, 149.6, 144.7, 137.5, 136.8, 135.1, 130.5, 130.0, 125.4, 124.1, 122.7, 38.1, 20.6.

HRMS (ESI$^+$) calcd for $C_{14}H_{17}O_2N_2S$ [M+H]$^+$: 277.1005, found 277.0995.

2-(2,6-dimethylphenyl)pyridine 4aa

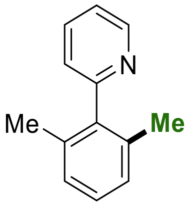

4aa

The title compound was synthesised as outlined in **General Procedure A**, using 2-(*o*-tolyl)pyridine **3aa** (51 mg, 0.30 mmol). Purification using flash column chromatography (90:10 Hexane:Et_2O) afforded **4aa** as a yellow-brown oil (43 mg, 79 %). Spectroscopic data match those previously reported.[9]

^{1}H NMR (400 MHz, $CDCl_3$) δ 8.73 (d, *J* = 4.9 Hz, 1H), 7.76 (td, *J* = 7.7, 1.7 Hz, 1H), 7.16–7.30 (m, 3H), 7.11 (d, *J* = 7.7 Hz, 2H), 2.05 (s, 6H).

^{13}C NMR (101 MHz, $CDCl_3$) δ 160.1, 149.8, 140.6, 136.4, 135.9, 128.0, 127.6, 124.6, 121.8, 20.3.

2-(2-methyl-6-fluorophenyl)pyridine 3ka

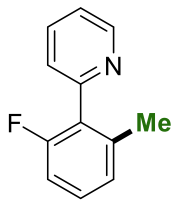

3ka

The title compound was synthesised as outlined in **General Procedure A**, using 2-(2-fluorophenyl)pyridine **1k** (52 mg, 0.30 mmol). Purification using flash column chromatography (90:10 Hexane:Et_2O) afforded **3ka** as a yellow oil (34 mg, 60 %). Spectroscopic data match those previously reported.[24]

^{1}H NMR (400 MHz, $CDCl_3$) δ 8.73 (ddd, *J* = 4.9, 1.6, 0.9 Hz, 1H), 7.77 (td, *J* = 7.7, 1.8 Hz, 1H), 7.32-7.40 (m, 1H), 7.25– 7.32 (m, 2H), 7.07 (d, *J* = 7.6 Hz, 1H), 6.99 (t, *J* = 8.9 Hz, 1H), 2.21 (s, 3H).

^{13}C NMR (101 MHz, $CDCl_3$) δ 161.6 (d, *J* = 245.4 Hz), 154.4, 149.7, 139.2 (d, *J* = 2.7 Hz), 136.3, 129.4 (d, *J* = 9.2 Hz), 128.4 (d, *J* = 15.5 Hz), 126.0 (d, *J* = 3.2 Hz), 125.6 (d, *J* = 1.9 Hz), 122.4, 113.1 (d, *J* = 22.6 Hz), 19.9 (d, *J* = 2.7 Hz).

^{19}F NMR (376 MHz, $CDCl_3$) δ -117.2;

2-(2-methyl-5-methoxyphenyl)pyridine 3la

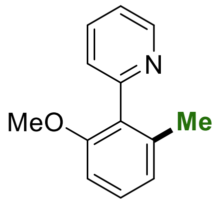

3la

The title compound was synthesised as outlined in **General Procedure A**, using 2-(2-methoxyphenyl)pyridine **1l** (56 mg, 0.30 mmol). Purification using flash column chromatography (3:1 Hexane:Et_2O) afforded **3la** as a yellow oil (45 mg, 76 %). Spectroscopic data match those previously reported.[9]

^{1}H NMR (400 MHz, $CDCl_3$) δ 8.70 (d, *J* = 4.9 Hz, 1H), 7.71 (td, *J* = 7.8, 1.4 Hz, 1H), 7.17–7.30 (m, 3H), 6.88 (d, *J* = 7.7 Hz, 1H), 6.81 (d, *J* = 8.3 Hz, 1H), 3.68 (s, 3H), 2.06 (s, 3H).

^{13}C NMR (101 MHz, $CDCl_3$) δ 157.2, 157.0, 149.5, 137.9, 136.0, 129.9, 128.9, 125.6, 122.7, 121.7, 108.5, 55.8, 19.9.

2-(2-methyl-5-trifluoromethylphenyl)pyridine 3ma

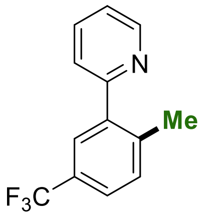

3ma

The title compound was synthesised as outlined in **General Procedure A**, using (3-(trifluoromethyl)phenyl)pyridine **1m** (67 mg, 0.30 mmol). Purification using flash column chromatography (90:10 Hexane:Et_2O) afforded **3ma** as a yellow oil (56 mg, 79 %). Spectroscopic data match those previously reported.[22]

^{1}H NMR (400 MHz, $CDCl_3$) δ 8.72 (ddd, *J* = 4.9, 1.6, 0.9 Hz, 1H), 7.78 (td, *J* = 7.7, 1.8 Hz, 1H), 7.66 (s, 1H), 7.55 (dd, *J* = 8.1, 1.5 Hz, 1H), 7.36-7.45 (m, 2H), 7.30 (ddd, *J* = 7.6, 4.9, 1.1 Hz, 1H), 2.42 (s, 3H).

^{13}C NMR (101 MHz, $CDCl_3$) δ 158.7, 149.6, 141.1, 140.2 (q, *J* = 1.4 Hz), 136.6, 131.3, 128.5 (q, *J* = 32.5 Hz), 126.7 (q, *J* = 3.8 Hz), 125.0 (q, *J* = 3.7 Hz), 124.4 (q, *J* = 272.2 Hz), 124.2, 122.4, 20.5.

^{19}F NMR (471 MHz, $CDCl_3$) δ -62.3.

2-(2-methyl-5-acetylphenyl)pyridine 3na

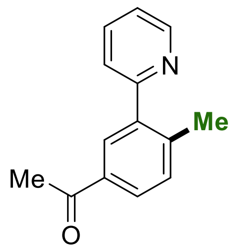

3na

The title compound was synthesised as outlined in **General Procedure A**. Purification using using 1-(3-(pyridin-2-yl)phenyl)ethan-1-one **1n** (59 mg, 0.30 mmol). Purification using flash column chromatography (12:1:1 Hexane:EtOAc:DCM) afforded **3na** as an orange oil (56 mg, 89 %).

^{1}H NMR (400 MHz, $CDCl_3$) δ 8.71 (ddd, *J* = 4.9, 1.6, 0.9 Hz, 1H), 7.98 (d, *J* = 1.9 Hz, 1H), 7.89 (dd, *J* = 8.0, 1.9 Hz, 1H), 7.77 (td, *J* = 7.7, 1.8 Hz, 1H), 7.42 (dd, *J* = 7.8, 1.0 Hz, 1H), 7.37 (d, *J* = 8.0 Hz, 1H), 7.28 (ddd, *J* = 7.6, 4.9, 1.1 Hz, 1H), 2.60 (s, 3H), 2.41 (s, 3H).

^{13}C NMR (101 MHz, $CDCl_3$) δ 197.8, 159.1, 149.5, 141.9, 140.8, 136.5, 135.2, 131.2, 129.9, 128.1, 124.2, 122.2, 26.7, 20.7.

HRMS (ESI^+) calcd for $C_{14}H_{14}NO$ $[M+H]^+$: 212.1070, found 212.1070.

2-(2,5-dimethylphenyl)pyridine 3oa

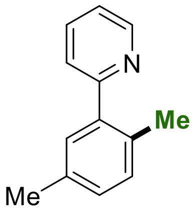

3oa

The title compound was synthesised as outlined in **General Procedure A**, using 2-(*m*-tolyl)pyridine **1o** (51 mg, 0.30 mmol). Purification using flash column chromatography (90:10 Hexane:Et_2O) afforded **3oa** as a yellow-brown oil (40 mg, 73 %). Spectroscopic data match those previously reported.[25]

^{1}H NMR (500 MHz, $CDCl_3$) δ 8.72 (ddd, *J* = 4.9. 1.7, 0.9 Hz, 1H), 7.74 (td, *J* = 7.7, 1.8 Hz, 1H), 7.41 (dt, *J* = 7.8, 1.0 Hz, 1H), 7.28 – 7.22 (m, 2H), 7.19 (d, *J* = 7.8 Hz, 1H), 7.14 (dd, *J* = 7.8, 1.5 Hz, 1H), 2.38 (s, 3H), 2.35 (s, 3H).

^{13}C NMR (126 MHz, $CDCl_3$) δ 160.2, 149.3, 140.3, 136.1, 135.4, 132.6, 130.8, 130.4, 129.1, 124.2, 121.6, 21.0, 19.9.

Methyl 4-methyl-3-(pyridine-2-yl)benzoate 3pa

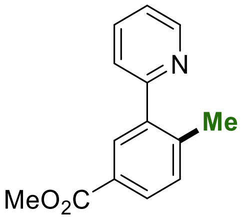

3pa

The title compound was synthesised as outlined in **General Procedure A**, using methyl 3-(pyridin-2-yl)benzoate **1p** (64 mg, 0.30 mmol). Purification using flash column chromatography (9:1:1 Hexane:EtOAc:DCM) afforded **3pa** as a yellow oil (54 mg, 80 %). Spectroscopic data match those previously reported.[26]

^{1}H NMR (500 MHz, $CDCl_3$) δ 8.70 (d, J = 4.5 Hz, 1H), 8.07 (s, 1H), 7.96 (d, J = 8.0 Hz, 1H), 7.75 (t, J = 7.7 Hz, 1H), 7.41 (d, J = 7.8 Hz, 1H), 7.34 (d, J = 7.9 Hz, 1H), 7.20-7.30 (m, 1H), 3.89 (s, 3H), 2.41 (s, 3H).

^{13}C NMR (101 MHz, $CDCl_3$) δ 167.0, 159.1, 149.4, 141.6, 140.6, 136.5, 131.0, 131.0, 129.3, 128.0, 124.1, 122.1, 52.1, 20.6.

2-(2-methyl-5-(methylsulfonyl)phenyl)pyridine 3qa

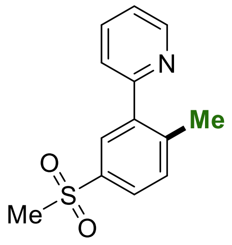

3qa

The title compound was synthesised as outlined in **General Procedure A**, using 2-(3-(methylsulfonyl)phenyl)pyridine **1q** (70 mg, 0.30 mmol). Purification using flash column chromatography (6:4 Hexane:EtOAc) afforded **3qa** as an off-white solid (59 mg, 80 %).

^{1}H NMR (400 MHz, $CDCl_3$) δ 8.70 (ddd, *J* = 4.9, 1.6, 0.9 Hz, 1H), 7.95 (d, *J* = 2.0 Hz, 1H), 7.85 (dd, *J* = 8.0, 2.0 Hz, 1H), 7.79 (td, *J* = 7.7, 1.8 1H), 7.47 (d, *J* =8.0 Hz, 1H), 7.42 (dt, *J* = 7.8, 1.0 Hz, 1H), 7.31 (ddd, *J* = 7.6, 4.9, 1.1 Hz, 1H), 3.05 (s, 3H), 2.44 (s, 3H).

^{13}C NMR (101 MHz, $CDCl_3$) δ 158.0, 149.6, 142.9, 141.7, 138.3, 136.7, 131.9, 128.7, 127.0, 124.2, 122.7, 44.7, 20.7.

HRMS (ESI^+) calcd for $C_{13}H_{14}NO_2S$ $[M+H]^+$: 248.0740, found 248.0739.

3-methyl-2-phenylpyridine 3ra

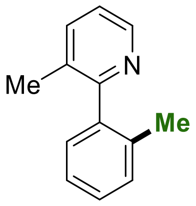

3ra

The title compound was synthesised as outlined in **General Procedure A**, using 3-methyl-2-phenylpyridine **1r** (51 mg, 0.30 mmol). Purification using flash column chromatography (95:5 Hexane:EtOAc) afforded **3ra** as a pale-yellow oil (31 mg, 56 %). Spectroscopic data match those previously reported.[22]

^{1}H NMR (400 MHz, CD_3CN) δ 8.47 (dd, *J* = 4.8, 1.1 Hz, 1H), 7.59 (ddd, *J* = 7.7, 1.7, 0.7 Hz, 1H), 7.22-7.34 (m, 3H), 7.19 (dd, *J* = 7.7, 4.8 Hz, 1H), 7.13 (d, *J* = 7.4 Hz, 1H), 2.08 (s, 3H), 2.06 (s, 3H).

^{13}C NMR (101 MHz, CD_3CN) δ 160.0, 147.0, 141.0, 138.0, 136.1, 131.9, 130.5, 128.8, 128.2, 125.9, 122.5, 19.5, 19.2.

HRMS (ESI^{+}) calcd for $C_{13}H_{14}N$ $[M+H]^{+}$: 184.1121, found 184.1121.

3-Methyl-2-(2-methylphenyl-3,4,5,6-*d*₄)pyridine *d*₄-3ra

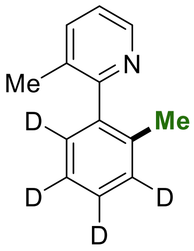

***d*₄-3ra**

The title compound was synthesised as outlined in **General Procedure A**, using 3-methyl-2-(d_5-phenyl)pyridine d_5-**1r** (51 mg, 0.30 mmol). Purification using flash column chromatography (95:5 Hexane:EtOAc) afforded ***d*₄-3ra** as a pale-yellow oil (30 mg, 53 %). Spectroscopic data match those previously reported.[9]

^{1}H NMR (500 MHz, $CDCl_3$) δ 8.51 (dd, J = 4.8, 1.0 Hz, 1H), 7.59 (dd, J = 7.7, 1.0 Hz, 1H), 7.20 (dd, J = 7.7, 4.8 Hz, 1H), 2.11 (s, 3H), 2.08 (s, 3H).

^{13}C NMR (126 MHz, $CDCl_3$) δ 159.7, 146.8, 140.2, 137.9, 135.5, 131.7, 129.9 (t, J = 24.0 Hz), 128.5, 127.6, (t, J = 24.3 Hz), 125.3 (t, J = 24.4 Hz), 122.3, 19.4, 19.1.

2-(*ortho*-tolyl)-5-methylpyridine 3sa

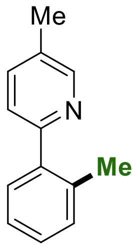

3sa

The title compound was synthesised as outlined in **General Procedure A**, using 5-methyl-2-phenylpyridine **1s** (51 mg, 0.30 mmol). Purification using flash column chromatography (90:10 Hexane:Et_2O) afforded **3sa** as a yellow oil (38 mg, 69 %). Spectroscopic data match those previously reported.[9]

^{1}H NMR (400 MHz, $CDCl_3$) δ 8.49-8.53 (m, 1H), 7.53 (ddd, *J* = 8.0, 2.3, 0.7 Hz, 1H), 7.32–7.41 (m, 1H), 7.20–7.32 (m, 4H), 2.37 (s, 3H), 2.35 (s, 3H).

^{13}C NMR (126 MHz, $CDCl_3$) δ 157.3, 149.7, 140.5, 136.8, 135.8, 131.1, 130.8, 129.7, 128.1, 125.9, 123.6, 20.4, 18.3.

2-(2,6-dimethylphenyl)-3-methylpyridine 4sa

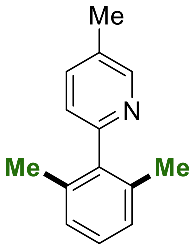

4sa

The title compound was synthesised as a by-product of the reaction outlined in **General Procedure A**, using 5-methyl-2-phenylpyridine **1s** (51 mg, 0.30 mmol). Purification using flash column chromatography (90:10 Hexane:Et_2O) afforded **4sa** as a yellow oil (4.1 mg, 7 %). Spectroscopic data match those previously reported.[9]

^{1}H NMR (400 MHz, $CDCl_3$) δ 8.55 (s, 1H), 7.56 (d, J = 7.9 Hz, 1H), 7.04-7.22 (m, 4H), 2.40 (s, 3H), 2.04 (s, 6H).

^{13}C NMR (101 MHz, $CDCl_3$) δ 157.1, 150.2, 140.6, 136.9, 136.1, 131.0, 127.8, 127.6, 124.0, 20.3, 18.4.

2-(*ortho*-tolyl)isoquinoline 3ta

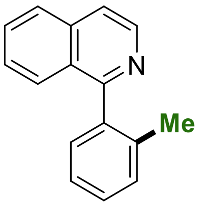

3ta

The title compound was synthesised as outlined in **General Procedure A**, using 1-phenylisoquinoline **1t** (62 mg, 0.30 mmol). Purification using flash column chromatography (8:1:1 Hexane:EtOAc:DCM) afforded **3ta** as a yellow oil (51 mg, 77 %). Spectroscopic data match those previously reported.[27]

^{1}H NMR (400 MHz, $CDCl_3$) δ 8.63 (d, *J* = 5.8 Hz, 1H), 7.82-7.90 (m, 1H), 7.61-7.70 (m, 3H), 7.46 (ddd, *J* = 8.4, 6.8, 1.2 Hz, 1H), 7.29–7.42 (m, 4H), 2.08 (s, 3H).

^{13}C NMR (101 MHz, $CDCl_3$) δ 161.5, 142.2, 139.0, 136.4, 136.4, 130.3, 130.0, 129.6, 128.4, 127.49, 127.46, 127.2, 126.9, 125.6, 119.9, 19.8.

Methyl 4-methyl-3-(1*H*-pyrazol-1-yl)benzoate 3ua

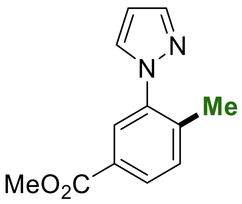

3ua

The title compound was synthesised as outlined in **General Procedure A**, using methyl 3-(1*H*-pyrazol-1-yl)benzoate **1u** (52 mg, 0.30 mmol). Purification using flash column chromatography (90:10 Hexane:Et_2O) afforded **3ua** as a yellow oil (51 mg, 79 %).

^{1}H NMR (400 MHz, $CDCl_3$) δ 7.92-8.00 (m, 2H), 7.72 (d, *J* = 1.6 Hz, 1H), 7.62 (d, *J* = 2.3 Hz, 1H), 7.37 (d, *J* = 7.9 Hz, 1H), 6.44 (app. t, *J* = 2.1 Hz, 1H), 3.89 (s, 3H), 2.30 (s, 3H).

^{13}C NMR (101 MHz, $CDCl_3$) δ 166.2, 140.7, 140.1, 139.4, 131.6, 130.6, 129.3, 129.0, 127.3, 106.7, 52.3, 18.6.

HRMS (ESI$^+$) calcd for $C_{12}H_{13}N_2O_2$ $[M+H]^+$: 217.0972, found 217.0974.

2-(2-methyl-6-fluorophenyl)pyrimidine 3va

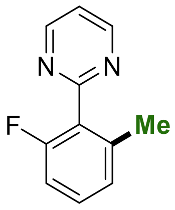

3va

The title compound was synthesised as outlined in **General Procedure A**, using 2-(2-fluorophenyl)pyrimidine **1v** (52 mg, 0.30 mmol). Purification using flash column chromatography (6:2:2 Hexane:EtOAc:DCM) afforded **3va** as a yellow oil (37 mg, 69 %).

^{1}H NMR (500 MHz, $CDCl_3$) δ 8.85 (d, *J* = 4.9 Hz, 2H), 7.29 – 7.22 (m, 2H), 7.04 (d, *J* = 7.6 Hz, 1H), 6.97 (t, *J* = 9.0 Hz, 1H), 2.20 (s, 3H).

^{13}C NMR (126 MHz, $CDCl_3$) δ 164.1, 160.2 (d, *J* = 247.4 Hz), 157.3, 138.8 (d, *J* = 2.5 Hz), 130.1 (d, *J* = 9.1 Hz), 127.4 (d, *J* = 14.7), 126.0 (d, *J* = 3.1 Hz), 119.5, 113.3 (d, *J* = 22.1 Hz), 19.6 (d, *J* = 2.7 Hz).

^{19}F NMR (471 MHz, $CDCl_3$) δ -117.6.

HRMS (ESI$^+$) calcd for $C_{11}H_{10}N_2F$ $[M+H]^+$: 189.0823, found 189.0826.

2-(2-methyl-5-trifluoromethylphenyl)oxazoline 3wa

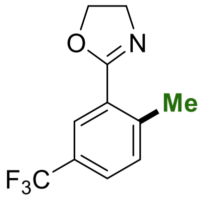

3wa

The title compound was synthesised as outlined in **General Procedure A**, using methyl 2-(3-(trifluoromethyl)phenyl)-4,5-dihydrooxazole **1w** (43 mg, 0.30 mmol). Purification using flash column chromatography (90:10 Hexane:Et_2O) afforded **3wa** as a pale-yellow solid (49 mg, 72 %).

^{1}H NMR (400 MHz, $CDCl_3$) δ 8.09 (s, 1H), 7.57 (dd, *J* = 8.0, 1.5 Hz, 1H), 7.36 (d, *J* = 8.0 Hz, 1H), 4.41 (t, *J* = 9.6 Hz, 2H), 4.12 (t, *J* = 9.6 Hz, 2H), 2.66 (s, 3H).

^{13}C NMR (101 MHz, $CDCl_3$) δ 164.0, 143.1, 131.9, 128.3 (q, *J* = 32.8 Hz), 127.9, 127.0 (q, *J* = 3.9 Hz), 127.0 (q, *J* = 2.6 Hz), 124.3 (q, *J* = 272.7 Hz), 67.1, 55.7, 22.1.

^{19}F NMR (376 MHz, $CDCl_3$) δ -62.5.

HRMS (ESI^+) calcd for $C_{11}H_{11}F_3NO$ $[M+H]^+$: 230.0787, found 230.0778.

2-(2-trideuteromethyl-6-methylphenyl)pyridine 4ab

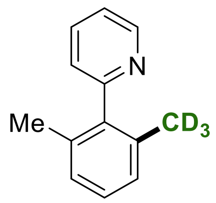

4ab

The title compound was synthesised as outlined in **General Procedure C**, using 2-phenylpyridine **1a** (47 mg, 0.30 mmol). Purification using flash column chromatography (98:2 Hexane:Et_2O) afforded **4ab** as a pale-yellow oil (43 mg, 78 %).

^{1}H NMR (400 MHz, $CDCl_3$) δ 8.73 (ddd, *J* = 4.8, 1.6, 1.0 Hz, 1H), 7.76 (td, *J* = 7.7, 1.8 Hz, 2H), 7.16-7.30 (m, 3H), 7.12 (d, *J* = 7.5 Hz, 2H), 2.06 (s, 3H).

^{2}H NMR (61 MHz, $CDCl_3$) δ 2.03 (s).

^{13}C NMR (101 MHz, $CDCl_3$) δ 160.0, 149.8, 140.6, 136.3, 135.8, 135.7, 127.9, 127.58, 127.56, 124.5, 121.7, 20.3, 19.6 (sept, *J* = 19.3 Hz).

HRMS (ESI$^+$) calcd for $C_{13}H_{12}D_3N$ $[M+H]^+$: 187.1309, found 187.1304.

2-(2-trideuteromethyl-5-trifluoromethylphenyl)pyridine 3mb

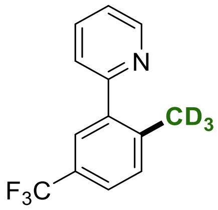

3mb

The title compound was synthesised as outlined in **General Procedure C**, using (3-(trifluoromethyl)phenyl)pyridine **1m** (67 mg, 0.30 mmol). Purification using flash column chromatography (98:2 Hexane Et_2O) afforded **3mb** as a pale-yellow oil (57 mg, 79%).

^{1}H NMR (500 MHz, $CDCl_3$) δ 8.72 (ddd, *J* = 4.9, 1.7, 0.9 Hz, 1H), 7.78 (td, *J* = 7.7, 1.8 Hz, 1H), 7.67 (d, *J* = 1.4 Hz, 1H), 7.52-7.58 (m, 1H) 7.36-7.44 (m, 2H), 7.27-7.33 (ddd, *J* = 7.6, 4.9, 1.1 Hz, 1H).

^{2}H NMR (77 MHz, $CDCl_3$) δ 2.40 (s).

^{13}C NMR (126 MHz, $CDCl_3$) δ 158.7, 149.6, 141.1, 140.1, 136.6, 131.3, 128.5 (q, *J* = 32.7 Hz), 127.6, 126.6 (q, *J* = 3.7 Hz), 125.0 (q, *J* = 3.7 Hz), 124.4 (q, *J* = 271.6 Hz), 124.2, 122.4, 19.7 (sept, *J* = 19.3 Hz).

^{19}F NMR (471 MHz, $CDCl_3$) δ -62.3.

HRMS (ESI$^+$) calcd for $C_{13}H_8D_3NF_3$ $[M+H]^+$: 241.1026, found 241.1025.

***N*,*N*-dimethyl-3-trideuteromethyl -4-(pyridin-2-yl)benzenesulfonamide 3jb**

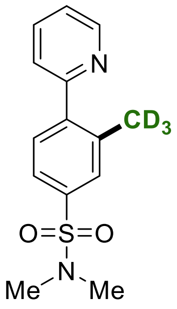

3jb

The title compound was synthesised as outlined in **General Procedure C**, using using *N,N*-dimethyl-4-(pyridin-2-yl)benzenesulfonamide **1j** (79 mg, 0.30 mmol). Purification using flash column chromatography (65:35 Hexane:EtOAc) afforded **3jb** as a orange-brown oil (64 mg, 77 %).

^{1}H NMR (400 MHz, $CDCl_3$) δ 8.72 (ddd, *J* = 4.9, 1.8, 1.0 Hz, 1H), 7.81 (td, *J* = 7.7, 1.8 Hz, 1H), 7.65-7.72 (m, 2H), 7.56 (d, *J* = 7.9 Hz, 1H), 7.42 (dt, *J* = 7.9, 1.0 Hz, 1H), 7.32 (ddd, *J* = 7.6, 4.9, 1.1 Hz, 1H), 2.73 (s, 6H).

^{2}H NMR (77 MHz, $CDCl_3$) δ 2.41 (s).

^{13}C NMR (101 MHz, $CDCl_3$) δ 158.4, 149.6, 144.8, 137.3, 136.7, 135.1, 130.4, 129.9, 125.4, 124.2, 122.7, 38.1, 19.8 (sept, *J* = 19.6 Hz).

HRMS (ESI$^+$) calcd for $C_{14}H_{13}D_3O_2N_2NaS$ $[M+Na]^+$: 302.1013, found 201.1008.

Methyl 4-(trideuteromethyl)-3-(1*H*-pyrazol-1-yl)benzoate 3ub

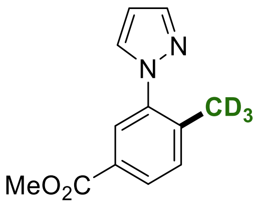

3ub

The title compound was synthesised as outlined in **General Procedure C**, using using using methyl 3-(1*H*-pyrazol-1-yl)benzoate **1u** (52 mg, 0.30 mmol). Purification using flash column chromatography (96:4 Hexane:EtOAc) afforded **3ub** as a colourless oil (53 mg, 81%).

^{1}H NMR (400 MHz, $CDCl_3$) δ 7.93-8.03 (m, 2H), 7.74 (d, *J* = 1.8 Hz, 1H), 7.63 (d, *J* = 2.4 Hz, 1H), 7.40 (d, *J* = 7.8 Hz, 1H), 6.46 (t, *J* = 2.1 Hz, 1H), 3.91 (s, 3H).

^{2}H NMR (77 MHz, $CDCl_3$) δ 2.30 (s).

^{13}C NMR (126 MHz, $CDCl_3$) δ 166.3, 140.8, 140.2, 139.3, 131.7, 130.7, 129.4, 129.0, 127.3, 106.7, 52.4, 17.9 (sept, *J* = 19.7 Hz).

HRMS (ESI$^+$) calcd for $C_{12}H_9D_3O_2N_2Na$ $[M+Na]^+$: 242.0979, found 242.0974.

2-(2-fluoro-6-trideuteromethylphenyl)pyrimidine 3vb

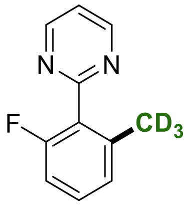

3vb

The title compound was synthesised as outlined in **General Procedure C**, using 2-(2-fluorophenyl)pyrimidine **1v** (52 mg, 0.30 mmol). Purification using flash column chromatography (90:10 Hexane:EtOAc) afforded **3va** as a colourless oil (42 mg, 74 %).

^{1}H NMR (400 MHz, $CDCl_3$) δ 8.89 (d, J = 5.0 Hz, 2H), 7.26-7.34 (m, 2H), 7.07 (dd, J = 7.7, 0.8 Hz, 1H), 7.00 (ddd, J = 9.7, 8.3, 1.0 Hz, 1H).

^{2}H NMR (77 MHz, $CDCl_3$) δ 2.22 (s).

^{13}C NMR (151 MHz, $CDCl_3$) δ 164.1, 160.5 (d, J = 248.1 Hz), 157.3, 138.7, 130.1 (d, J = 9.1 Hz), 127.4 (d, J = 14.6 Hz), 126.1 (d, J = 1.8 Hz), 119.5, 113.3 (d, J = 22.0 Hz), 18.8 (septd, J = 19.5, 2.6 Hz)

^{19}F NMR (376 MHz, $CDCl_3$) δ -117.7

HRMS (ESI^{+}) calcd for $C_{11}H_7D_3N_2F$[M+H]$^{+}$: 192.1011, found 192.1011.

2-(2-(trideuteromethyl)-5-(trifluoromethyl)phenyl)-4,5-dihydrooxazole 3wb

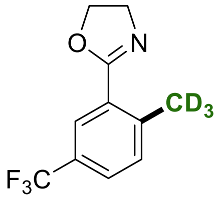

3wb

The title compound was synthesised as outlined in **General Procedure C**, using methyl 2-(3-(trifluoromethyl)phenyl)-4,5-dihydrooxazole **1w** (43 mg, 0.30 mmol). Purification using flash column chromatography (90:10 Hexane:Et_2O) afforded **3wa** as a pale-yellow solid (49 mg, 72 %).

^{1}H NMR (400 MHz, CD_3CN) δ 7.99 (d, J = 2.4 Hz, 1H), 7.62 (dd, J = 8.0, 1.4 Hz, 1H), 7.44 (d, J = 8.0 Hz, 1H), 4.34 (t, J = 9.5 Hz, 2H), 4.00 (t, J = 9.5 Hz, 2H).

^{2}H NMR (77 MHz, $CDCl_3$) δ 2.64 (s).

^{13}C NMR (101 MHz, $CDCl_3$) δ 164.0, 143.1, 131.9, 128.3 (q, *J* = 32.8 Hz), 127.9, 127.0 (m, 2C), 124.3 (q, *J* = 272.7 Hz), 67.1, 55.7, 21.6 (m).

^{19}F NMR (376 MHz, $CDCl_3$) δ -62.5.

HRMS (ESI^+) calcd for $C_{11}H_8D_3ONF_3$ $[M+H]^+$: 233.0976, found 233.0977.

1-(2-methyl-5-(trifluoromethyl)phenyl)ethan-1-ol 6aa

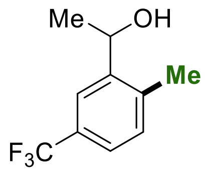

6aa

The title compound was synthesised as outlined in **General Procedure A**, using (*E*)-*N,N*-dimethyl-4-((1-(3-(trifluoromethyl)phenyl)ethylidene)amino)aniline **5a** (92 mg, 0.30 mmol) as starting material and ammonium salt **2d** (253 mg, 0.600 mmol, 2 equiv). After 72 hours, HCl (aq. 2N, 2 mL) was added to the reaction mixture, and stirred for a further 30 minutes, before being extracted with ether and the solvent removed under reduced pressure. To the reaction crude dissolved in methanol (2 mL), $NaBH_4$ (50 mg, 0.40 mmol, 1.3 equiv) were added in small portions at 0 °C. After the addition was completed, the mixture was allowed to warm to room temperature and stirred for one hour. Subsequently, the mixture was cooled to 0 °C, water was added and the MeOH was evaporated under vacuum. To the mixture 5% aqueous $NaHCO_3$ solution and EtOAc was added. The organic layer was dried over Na_2SO_4, filtered and concentrated in vacuo.. Purification using flash column chromatography (98:2 Pentane:Et_2O) afforded **6aa** as a colorless oil (41 mg, 67%). Spectroscopic data match those previously reported.[28]

^{1}H NMR (400 MHz, $CDCl_3$) δ 7.72 (s, 1H), 7.33 (d, *J* = 7.9 Hz, 1H), 7.15 (d, *J* = 7.9 Hz, 1H), 5.06 (qd, *J* = 6.3, 2.1 Hz, 1H), 2.29 (s, 3H), 1.93 (d, *J* = 2.1 Hz 1H), 1.38 (d, *J* = 6.4 Hz, 3H).

^{13}C NMR (101 MHz, $CDCl_3$) δ 144.7, 138.2 (q, *J* = 1.0 Hz), 130.70, 128.7 (q, *J* = 32.2 Hz), 124.4 (q, *J* = 271.9 Hz), 123.8 (q, *J* = 3.8 Hz), 121.6 (q, *J* = 3.8 Hz), 66.5, 24.0, 18.9.

^{19}F NMR (471 MHz, $CDCl_3$) δ -62.3

1-(2-(methyl-d_3)-5-(trifluoromethyl)phenyl)ethan-1-ol 6ab

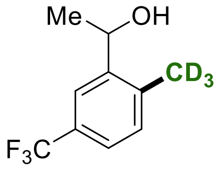

6ab

The title compound was synthesised as outlined in **General Procedure D**, using (*E*)-*N,N*-dimethyl-4-((1-(3-(trifluoromethyl)phenyl)ethylidene)amino)aniline **5a** (92 mg, 0.30 mmol) as starting material and deuterated ammonium salt **2e** (253 mg, 0.600 mmol, 2 equiv). After 72 hours, HCl (aq. 2N, 2 mL) was added to the reaction mixture, and stirred for a further 30 minutes, before being extracted with ether and the solvent removed under reduced pressure. To the reaction crude dissolved in methanol (2 mL), $NaBH_4$ (50 mg, 0.40 mmol, 1.3 equiv) were added in small portions at 0 °C. After the addition was completed, the mixture was allowed to warm to room temperature and stirred for one hour. Subsequently, the mixture was cooled to 0 °C, water was added and the MeOH was evaporated under vacuum. To the mixture 5% aqueous $NaHCO_3$ solution and EtOAc was added. The organic layer was dried over Na_2SO_4, filtered and concentrated in vacuo.. Purification using flash column chromatography (98:2 Pentane:EtO) afforded **6ab** as a colorless oil (39 mg, 63%).

^{1}H NMR (400 MHz, CDCl$_3$) δ 7.80 (s, 1H), 7.41 (dd, *J* = 7.9, 1.0 Hz, 1H), 7.23 (d, *J* = 7.9 Hz, 1H), 5.15 (q, *J* = 6.4 Hz, 1H), 1.91 (s, 1H), 1.47 (d, *J* = 6.4 Hz, 3H).

^{13}C NMR (126 MHz, CDCl$_3$) δ 144.8, 138.2, 130.8, 128.9 (q, *J* = 32.2 Hz), 124.51 (q, *J* = 271.9 Hz), 123.9 (q, *J* = 3.7 Hz), 121.7 (q, *J* = 3.8 Hz), 66.7, 24.2, 18.26 (sept, *J* = 19.4 Hz).

^{19}F NMR (471 MHz, CDCl$_3$) δ -62.3

. **^{2}H NMR (77 MHz, CDCl$_3$)** δ 2.36.

HRMS (ESI$^+$) calcd for $C_{10}H_9D_3F_3O$ $[M+H]^+$: 207.0956, found 207.0956.

(2-methyl-6-(trifluoromethyl)phenyl)methanol 6ba

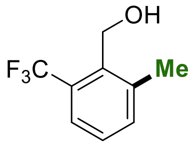

6ba

The title compound was synthesised as outlined in **General Procedure A**, using (*E*)-*N,N*-dimethyl-4-((2-(trifluoromethyl)benzylidene)amino)aniline **5b** (88 mg, 0.30 mmol) as starting material and ammonium salt **2d** (253 mg, 0.600 mmol, 2 equiv). After 72 hours, HCl (aq. 2N, 2 mL) was added to the reaction mixture, and stirred for a further 30 minutes, before being extracted with ether and the solvent removed under reduced pressure. To the reaction crude dissolved in methanol (2 mL), $NaBH_4$ (50 mg, 0.40 mmol, 1.3 equiv) were added in small portions at 0 °C. After the addition was completed, the mixture was allowed to warm to room temperature and stirred for one hour. Subsequently, the mixture was cooled to 0 °C, water was added and the MeOH was evaporated under vacuum. To the mixture 5% aqueous $NaHCO_3$ solution and EtOAc was added. The organic layer was dried over Na_2SO_4, filtered and concentrated in vacuo.. Purification using flash column chromatography (98:2 Pentane:EtO) afforded **6ba** as a colourless solid. (40 mg, 70%). m.p.: 74-76 ºC.

^{1}H NMR (400 MHz, CDCl$_3$) δ 7.43 (d, *J* = 7.7 Hz, 1H), 7.33 (d, *J* = 7.7 Hz, 1H), 7.23 (t, *J* = 7.7 Hz, 1H), 4.73 (d, *J* = 6.0 Hz, 2H), 2.45 (s, 3H), 1.62 (t, *J* = 6.0 Hz, 1H).

^{13}C NMR (101 MHz, CDCl$_3$) ^{13}C NMR (101 MHz, $CDCl_3$) δ 140.6, 136.5 (q, *J* = 1.3 Hz), 134.6 (q, *J* = 0.7 Hz), 129.2 (q, *J* = 29.4 Hz), 128.2, 124.8 (q, *J* = 274.0 Hz), 123.7 (q, *J* = 5.9 Hz), 58.7 (q, *J* = 2.4 Hz), 19.3. δ

^{19}F NMR (376 MHz, CDCl$_3$) δ -57.70.

Melting Point 74-76 ºC

HRMS (ESI$^+$) calcd for $C_9H_8F_3O$ [(M-H_2O)+H]$^+$: 173.0578, found 173.0581.

(2-(methyl-*d*$_3$)-6-(trifluoromethyl)phenyl)methanol 6bb

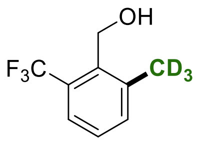

6bb

The title compound was synthesised as outlined in **General Procedure D**, using (*E*)-*N,N*-dimethyl-4-((2-(trifluoromethyl)benzylidene)amino)aniline **5b** (88 mg, 0.30 mmol) as starting material and deuterated ammonium salt **2e** (253 mg, 0.600 mmol, 2 equiv). After 72 hours, HCl (aq. 2N, 2 mL) was added to the reaction mixture, and stirred for a further 30 minutes, before being extracted with ether and the solvent removed under reduced pressure. To the reaction crude dissolved in methanol (2 mL), $NaBH_4$ (50 mg, 0.39 mmol, 1.3 equiv) were added in small portions at 0 °C. After the addition was completed, the mixture was allowed to warm to room temperature and stirred for one hour. Subsequently, the mixture was cooled to 0 °C, water was added and the MeOH was evaporated under vacuum. To the mixture 5% aqueous $NaHCO_3$ solution and EtOAc was added. The organic layer was dried over Na_2SO_4, filtered and concentrated in vacuo.. Purification using flash column chromatography(98:2 Pentane:EtO) afforded **6bb** as a colourless solid (35 mg, 61%).

^{1}H NMR (400 MHz, $CDCl_3$) δ 7.45 (d, *J* = 7.8 Hz, 1H), 7.35 (d, *J* = 7.5 Hz, 1H), 7.25 (m, 1H), 4.74 (d, *J* = 6.3 Hz, 2H), 1.53 (t, *J* = 6.3 Hz, 1H)

^{13}C NMR (126 MHz, $CDCl_3$) 140.5, 136.6, 134.7, 129.2 (q, *J* = 29.4 Hz), 128.2, 124.8 (q, *J* = 274.0 Hz), 123.7 (q, *J* = 5.9 Hz), 58.7 (q, *J* = 2.4 Hz), 18.5 (m).

^{19}F NMR (471 MHz, $CDCl_3$) δ -57.7.

^{2}H NMR (77 MHz, $CDCl_3$) δ 2.51

Melting Point 78-80 ºC

HRMS (ESI^+) calcd for $C_9H_5D_3F_3$ $[(M-H_2O)+H]^+$: 176.0766, found 176.0768.

2-methyl-1-naphthaldehyde 6ca

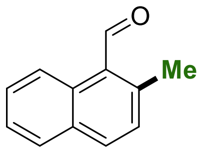

6ca

The title compound was synthesised as outlined in **General Procedure A**, using (*E*)-*N,N*-dimethyl-4-((naphthalen-1-ylmethylene)amino)aniline **5c** (82 mg, 0.30 mmol) as starting material and ammonium salt **2d** (253 mg, 0.600 mmol, 2 equiv). After 72 hours, HCl (aq. 2N, 2 mL) was added to the reaction mixture, and stirred for a further 30 minutes, before being extracted with ether and the solvent removed under reduced pressure. Purification using flash column chromatography (98:2 Pentane:EtO) afforded **6ca** as a colourless solid (38 mg, 74%).

^{1}H NMR (400 MHz, $CDCl_3$) δ 10.96 (s, 1H), 8.97 (d, *J* = 8.7 Hz, 1H), 7.93 (d, *J* = 8.4 Hz, 1H), 7.83 (d, *J* = 8.1 Hz, 1H), 7.67 – 7.59 (m, 1H), 7.50 (t, *J* = 7.4 Hz, 1H), 7.33 (d, *J* = 8.4 Hz, 1H), 2.81 (s, 3H).

^{13}C NMR (101 MHz, $CDCl_3$) δ 193.4, 142.8, 134.5, 132.6, 131.5, 129.9, 128.8, 128.5, 128.5, 126.0, 124.4, 20.2

Melting Point 44-46 ^{o}C

HRMS (ESI^+) calcd for $C_{12}H_{11}O$ $[M+H]^+$: 171.0804, found 171.0804.

7-chloro-1-methyl-5-(*o*-tolyl)-1,3-dihydro-2*H*-benzo[*e*[[1,4]diazepin-2-one (*Diazepam*) 8aa

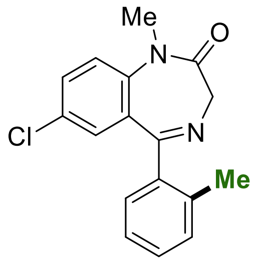

8aa

The title compound was synthesised as outlined in **General Procedure B**, using Diazepam **7a** (28 mg, 0.10 mmol). Purification using flash column chromatography (70:30 Hexane:EtOAc) afforded impure product as a colourless solid. Further purification by preparative normal phase HPLC (20 to 50 % EtOAc in Hexane over 35 minutes) afforded the title product as a colourless solid (26 mg, 86 %). Spectroscopic data match those previously reported.[29]

^{1}H NMR (400 MHz, $CDCl_3$) δ 7.47 (dd, *J* = 8.8, 2.5 Hz, 1H), 7.31-7.39 (m, 2H), 7.24-7.30 (m, 2H), 7.19 (d, *J* = 7.7 Hz, 1H), 7.06 (d, *J* = 2.4 Hz, 1H), 4.86 (d, *J* = 10.9 Hz, 1H), 3.81 (d, *J* = 20.9 Hz, 1H), 3.43 (s, 3H), 1.98 (s, 3H).

^{13}C NMR (126 MHz, $CDCl_3$) δ 171.1, 169.9, 141.8, 138.7, 136.4, 131.8, 131.6, 131.0, 130.0, 129.8(3), 129.8(1), 129.1, 126.2, 122.7, 56.8, 35.0, 20.1.

Melting Point 139-141 ^{o}C

7-chloro-1-methyl-5-(*o*-trideuteromethylphenyl)-1,3-dihydro-2*H*-benzo[*e*[[1,4]diazepin-2-one (*Diazepam*) 8ab

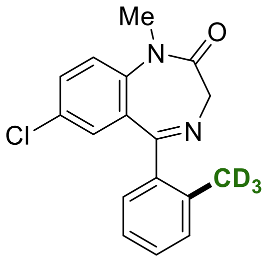

8ab

The title compound was synthesised as outlined in **General Procedure*J* B**, using Diazepam **7a** (28 mg, 0.10 mmol). Purification using flash column chromatography (70:30 Hexane:EtOAc) afforded impure product as a colourless solid. Further purification by preparative normal phase HPLC (20 to 50 % EtOAc in Hexane over 35 minutes) afforded the title product as a colourless solid (26 mg, 86 %).

^{1}H NMR (400 MHz, $CDCl_3$) δ 7.44 (dd, *J* = 8.8, 2.5 Hz, 1H), 7.28-7.35 (m, 2H), 7.20-7.27 (m, 2H), 7.12-7.18 (m, 1H), 7.02 (d, *J* = 2.5 Hz, 1H), 4.82 (d, *J* = 10.9 Hz, 1H), 3.78 (d, *J* = 10.9 Hz, 1H), 3.40 (s, 3H).

^{2}H NMR (61 MHz, $CDCl_3$) δ 1.95 (s).

^{13}C NMR (101 MHz, $CDCl_3$) δ 170.9, 169.9, 141.7, 138.8, 136.3, 131.9, 131.5, 131.0, 129.9, 129.77, 129.76, 129.1, 126.1, 122.7, 56.9, 34.9, 19.3 (sept, *J* = 19.6 Hz).

Melting Point ºC 141-142 ºC

HRMS (ESI$^+$) calcd for $C_{17}H_{13}D_3ON_2Cl$ $[M+H]^+$: 302.1134, found 302.1134.

2-(2-methyl-3,4,5-trimethoxyphenyl)isoquinoline 8ba

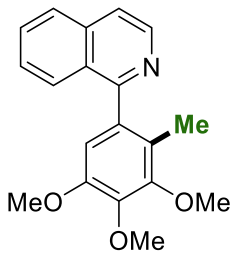

8ba

The title compound was synthesised as outlined in **General Procedure B**, using 1-(3,4,5-trimethoxyphenyl)isoquinoline **7b** (88 mg, 0.30 mmol). Purification using flash column chromatography (70:30 Hexane:EtOAc) afforded **8ba** as a colourless oil (21 mg, 22 %).

^{1}H NMR (400 MHz, $CDCl_3$) δ 8.60 (d, *J* = 5.7 Hz, 1H), 7.89 (d, *J* = 8.4 Hz, 1H), 7.64–7.73 (m, 3H), 7.48-7.55 (m, 1H), 6.69 (s, 1H), 3.96 (s, 3H), 3.92 (s, 3H), 3.82 (s, 3H), 1.87 (s, 3H).

^{13}C NMR (126 MHz, $CDCl_3$) δ 161.2, 152.2, 151.4, 142.5, 142.4, 136.5, 134.7, 130.3, 127.7, 127.6, 127.5, 127.0, 123.2, 120.1, 109.1, 61.1, 60.8, 56.2, 13.0.

HRMS (ESI^+) calcd for $C_{19}H_{20}O_3N$ $[M+H]^+$: 310.1438, found 310.1432.

(2-(2,4-dimethylphenyl)-6-methylimidazo[1,2-a]pyridin-3-yl)(piperidin-1-yl)methanone 8ca

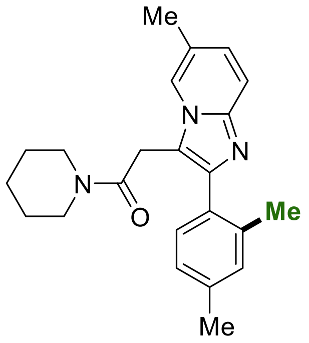

8ca

The title compound was synthesised as outlined in **General Procedure B**, using Zolpidem derivative **7c** (33 mg, 0.10 mmol). Purification using flash column chromatography (90:10 EtOAc:Hexane) afforded impure product as a colourless solid. Further purification by preparative reverse phase HPLC (30-60 % (80:20:0.1 MeCN:H_2O:TFA) in H_2O over 30 minutes) afforded the title product as a light yellow oil (11 mg, 29%).

^{1}H NMR (400 MHz, CD_3CN) δ 8.22 (s, 1H), 8.03 (d, *J* = 9.2 Hz, 1H), 7.73 (dd, *J* = 9.2, 1.3 Hz, 1H), 7.24 (s, 1H), 7.12-7.22 (m, 2H), 3.90 (s, 2H), 3.44-3.51 (m, 2H), 3.34-3.42 (m, 2H), 2.46 (d, *J* = 0.7 Hz, 3H), 2.39 (s, 3H), 2.23 (s, 3H), 1.58-1.68 (m, 2H), 1.45-1.55 (m, 4H)

^{13}C NMR (101 MHz, CD_3CN) δ 165.9, 141.8, 139.5, 139.1, 136.1, 134.5, 132.5, 131.7, 127.9, 127.8, 125.5, 124.3, 120.4, 112.8, 47.4, 43.7, 29.0, 27.0, 26.3, 25.0, 21.4, 19.8, 18.2.

HRMS (ESI^+) calcd for $C_{23}H_{28}ON_3$ $[M+H]^+$: 362.2227, found 362.2229.

2-(2-methyl-4-(methylsulfonyl)phenyl)imidazo[1,2-a]pyridine 8da and 2-(2,6-dimethyl-4-(methylsulfonyl)phenyl)imidazo[1,2-a]pyridine 9da

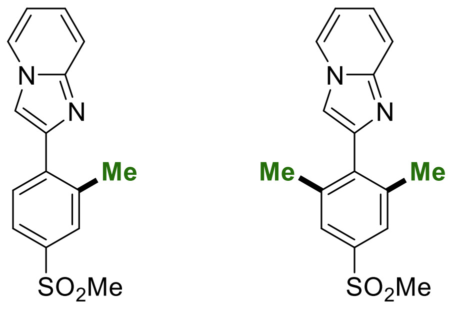

8da **9da**

The title compound was synthesised as outlined in **General Procedure B**, using Zolimidine **7d** (27 mg, 0.10 mmol). Purification using flash column chromatography (80:20 EtOAc:Hexane) afforded impure product as a colourless solid. Further purification by Reverse Phase HPLC (10-50 % (80:20:0.1 MeCN:H_2O:TFA) in H_2O over 25 minutes) afforded a mixture 1:3 of products **8da** and **9da** respectevely as a colourless solid (26 mg, 58%).

*NMR data for major product **9da**:*

^{1}H NMR (400 MHz, $CDCl_3$) δ 8.21 – 8.17 (m, 1H), 7.84 (d, J = 14.4 Hz, 1H), 7.68 (s, 2H), 7.52 (s, 1H), 7.26 – 7.20 (m, 1H), 6.88 – 6.83 (m, 1H), 3.06 (s, 3H), 2.25 (s, 6H).

^{13}C NMR (101 MHz, $CDCl_3$) δ 145.4, 142.8, 139.9, 139.8, 139.5, 125.9, 125.7, 124.7, 117.82, 112.6, 110.8, 44.6, 20.9.

HRMS 8da (ESI$^+$) calcd for $C_{15}H_{15}O_2N_2S$ $[M+H]^+$: 287.0849, found 287.0850.

HRMS 9da (ESI$^+$) calcd for $C_{16}H_{17}O_2N_2S$ $[M+H]^+$: 301.1005, found 301.1004.

(*R*)-2,8-dimethyl-2-((4*R*,8*R*)-4,8,12-trimethyltridecyl)chroman-6-yl 4-methyl-3-(pyridin-2-yl)benzoate 8ea

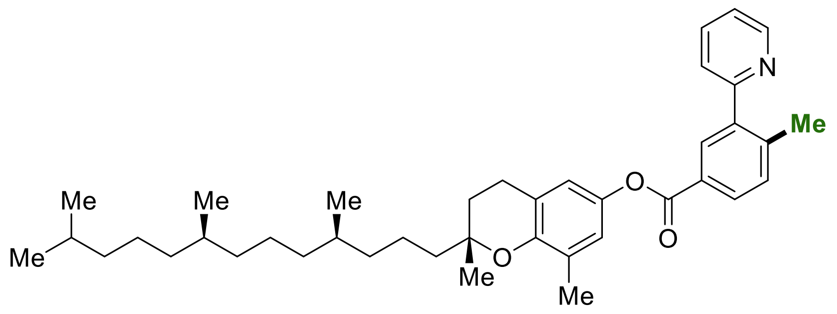

8ea

The title compound was synthesised as outlined in **General Procedure B**, using δ-tocopherol derivative **7e** (116 mg, 0.200 mmol). Purification using flash column chromatography (90:10 Hexane:EtOAc) afforded the title product as a colourless oil (84 mg, 70%).

^{1}H NMR (400 MHz, CDCl$_3$) δ 8.75 – 8.71 (m, 1H), 8.22 (d, *J* = 1.8 Hz, 1H), 8.11 (dd, *J* = 8.0, 1.8 Hz, 1H), 7.78 (td, *J* = 7.8, 1.8 Hz, 1H), 7.45 (d, *J* = 7.8 Hz, 1H), 7.41 (d, *J* = 8.0 Hz, 1H), 7.28 (ddd, *J* = 7.6, 4.9, 1.0 Hz, 1H), 6.81 (d, *J* = 2.4 Hz, 1H), 6.75 (d, *J* = 2.5 Hz, 1H), 2.82 – 2.69 (m, 2H), 2.46 (s, 3H), 2.18 (s, 3H), 1.88 – 1.70 (m, 2H), 1.65 – 1.00 (m, 26H), 0.96 – 0.78 (m, 12H).

^{13}C NMR (101 MHz, CDCl$_3$) δ 165.8, 159.1, 149.9, 149.4, 142.8, 142.2, 140.8, 136.5, 131.4, 131.2, 129.9, 127.8, 127.4, 124.2, 122.2, 121.4, 121.1, 119.3, 76.2, 40.2, 39.5, 37.6, 37.5(2C), 37.4, 32.9, 32.8, 31.1, 28.1, 24.9, 24.6, 24.4, 22.8, 22.7, 22.6, 21.1, 20.7, 19.9, 19.8, 16.3.

HRMS (ESI$^+$) calcd for $C_{40}H_{56}NO_3$ [M+H]$^+$: 598.4260, found 598.4272.

(8*S*,9*R*,13*R*,14*R*)-13-methyl-17-oxo-7,8,9,11,12,13,14,15,16,17-decahydro-6H-cyclopenta[a]phenanthren-3-yl 4-methyl-3-(pyridin-2-yl)benzoate 8fa

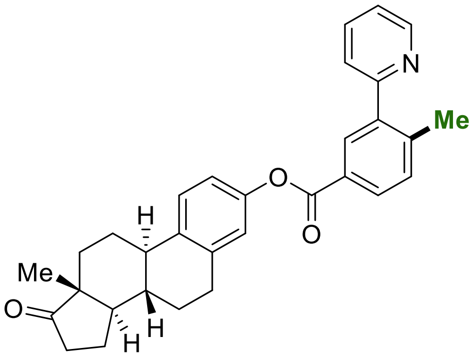

8fa

The title compound was synthesised as outlined in **General Procedure B**, using estrone derivative **7f** (45 mg, 0.10 mmol). Purification using flash column chromatography (90:10 Hexane:EtOAc) afforded the title product as a colourless solid (41 mg, 88%).

^{1}H NMR (400 MHz, CDCl$_3$) δ 8.75 – 8.70 (m, 1H), 8.22 (d, *J* = 1.8 Hz, 1H), 8.11 (dd, *J* = 8.0, 1.9 Hz, 1H), 7.79 (td, *J* = 7.7, 1.8 Hz, 1H), 7.45 (d, *J* = 7.8 Hz, 1H), 7.42 (d, *J* = 8.0 Hz, 1H), 7.34 – 7.27 (m, 2H), 6.97 (dd, *J* = 8.4, 2.5 Hz, 1H), 6.94 (d, *J* = 2.3 Hz, 1H), 2.97 – 2.89 (m, 2H), 2.51 (dd, *J* = 18.9, 8.6 Hz, 1H), 2.46 (s, 3H), 2.45 – 2.39 (m, 1H), 2.35 – 2.26 (m, 1H), 2.20 – 1.94 (m, 4H), 1.72 – 1.40 (m, 6H), 0.92 (s, 3H).

^{13}C NMR (101 MHz, CDCl$_3$) δ 220.9, 165.4, 159.0, 149.5, 149.0, 142.4, 140.9, 138.1, 137.5, 136.5, 131.5, 131.2, 130.0, 127.5, 126.5, 124.2, 122.2, 121.8, 119.0, 50.5, 48.1, 44.3, 38.1, 36.0, 31.7, 29.5, 26.5, 25.9, 21.7, 20.8, 14.0.

Melting Point 116-118 ^{o}C

HRMS (ESI^{+}) calcd for $C_{31}H_{32}NO_3$ [M+H]$^{+}$: 466.2382, found 466.2392.

2-methoxy-4-((*E*)-3-oxo-3-(((2a*R*,3*R*,5b*S*,7a*R*,9*S*,11a*R*,12a*S*)-2a,5b,8,8-tetramethyl-3-((*R*)-6-methyl-5-methyleneheptan-2-yl)tetradecahydro-1*H*,12*H*-cyclopenta[a]cyclopropa[e]phenanthren-9-yl)oxy)prop-1-en-1-yl)phenyl 4-methyl-3-(pyridin-2-yl)benzoate 8ga

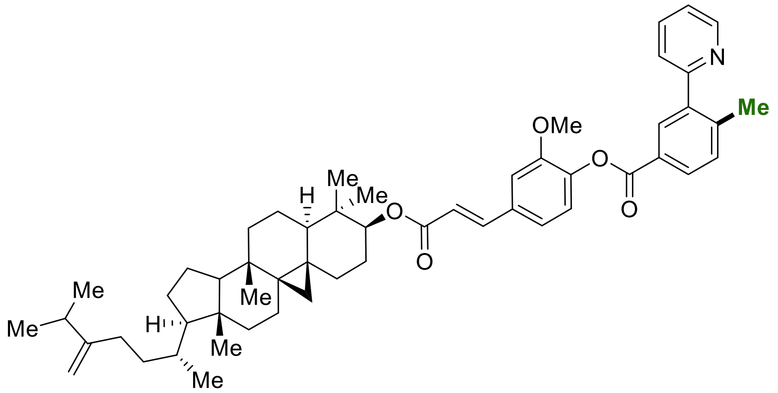

8ga

The title compound was synthesised as outlined in **General Procedure B**, using γ-oryzanol derivative **7g** (80 mg, 0.10 mmol). Purification using flash column chromatography (90:10 Hexane:EtOAc) afforded the title product as a colourless solid (53 mg, 66%).

^{1}H NMR (400 MHz, CDCl$_3$) δ 8.72 (dd, *J* = 4.8, 0.8 Hz, 1H), 8.24 (d, *J* = 1.8 Hz, 1H), 8.13 (dd, *J* = 7.9, 1.8 Hz, 1H), 7.78 (td, *J* = 7.7, 1.8 Hz, 1H), 7.65 (d, *J* = 15.9 Hz, 1H), 7.46 (d, *J* = 6.9 Hz, 1H), 7.45 – 7.41 (m, 1H), 7.34 – 7.27 (m, 1H), 7.19 – 7.10 (m, 3H), 6.41 (d, *J* = 15.9 Hz, 1H), 4.69 (d, *J* = 20.0 Hz, 2H), 3.84 (s, 3H), 2.46 (s, 3H), 2.31 – 1.83 (m, 7H), 1.79 – 1.07 (m, 23H), 1.03 (dd, *J* = 6.8, 2.2 Hz, 5H), 0.98 (d, *J* = 1.8 Hz, 6H), 0.94 – 0.83 (m, 11H), 0.61 (d, J = 4.2 Hz, 1H), 0.37 (d, J = 4.1 Hz, 1H).

^{13}C NMR (101 MHz, CDCl$_3$) δ 166.7, 164.3, 158.9, 156.9, 151.6, 149.4, 143.6, 142.5, 141.7, 140.8, 136.4, 133.5, 131.6, 131.2, 130.1, 126.9, 124.1, 123.4, 122.1, 121.3, 119.1, 111.3, 106.0, 80.8, 56.0, 52.3, 48.8, 47.9, 47.2, 45.3, 39.7, 36.1, 35.5, 35.0, 33.8, 32.9, 31.7, 31.3, 29.8, 28.2, 26.9, 26.5, 26.0, 25.8, 25.5(2C), 22.0, 21.9, 21.0, 20.7, 20.2, 19.3, 18.3, 18.0, 15.4.

Melting Point 104-106 ^{o}C

HRMS (ESI$^+$) calcd for $C_{54}H_{70}NO_5$ [M+H]$^+$: 812.5254, found 812.5262.

6. Large Scale reaction

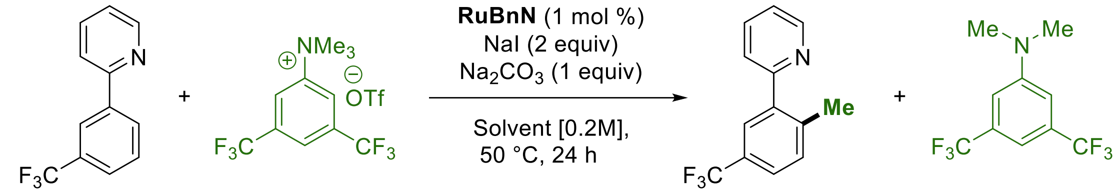

1m 9 mmol, 2.01 g

2d 9 mmol, 3,79 g

3ma NMP 1.93 g, 91%; Acetone 2.0 g, 92%

1.81 g, 78%

Scheme S9. Large scale reaction.

Product **3ma** was synthesised following a modification of **General Procedure A**, using (3-(trifluoromethyl)phenyl)pyridine **1m** (2.0 g, 9.0 mmol), *N,N,N*-Trimethyl-3,5-bis(trifluoromethyl)anilinium triflate **2d** (3.8 g, 9.0 mmol, 1 equiv), sodium carbonate (0.96 g, 9.0 mmol, 1 equiv), NaI (2.7 g, 18 mmol, 2 equiv), and **RuBnN** (48 mg, 1 mol %, 0.09 mmol), in NMP or acetone.

For the reaction in NMP: Purification using flash column chromatography (90:10 Hexane:Et_2O) afforded **3ma** as a yellow oil (1.93 g, 91%) and *N,N*-dimethyl-3,5-bis(trifluoromethyl)aniline as a colourless solid (1.80 g, 78%).

For the reaction in Acetone: Purification using flash column chromatography (90:10 Hexane:Et_2O) afforded **3ma** as a yellow oil (2.01 g, 92%) and *N,N*-dimethyl-3,5-bis(trifluoromethyl)aniline as a colourless solid (1.81 g, 78%).

7. Mechanistic Studies

7.1. Competition experiments between electron-rich and poor aromatics

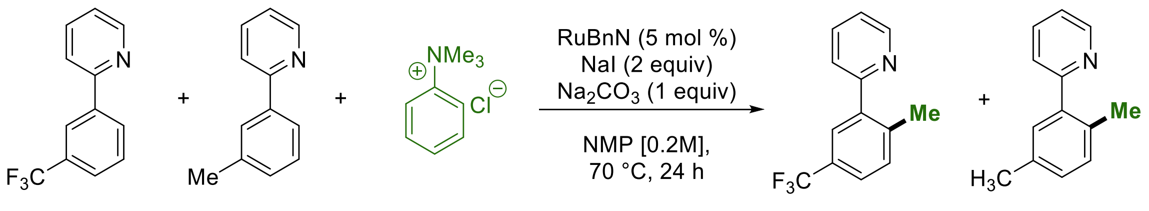

1oa 0.1 mmol | **1ma** 0.1 mmol | **2a** 0.1 mmol | **3ma** 72% | **3oa** 18%

Scheme S10. *meta*-substituted 2-phenylpyridine competition experiment

In an argon-filled glove box, an oven-dried crimp-cap microwave vial equipped with a magnetic stirring bar was charged with **1o** (22 mg, 0.20 mmol), **1m** (17 mg, 0.20 mmol), **2a** (17 mg, 0.20 mmol), NaI (30 mg, 0.20 mmol), Na_2CO_3 (11 mg, 0.10 mmol), **RuBnN** (2.70 mg, 0.005 mmol), and NMP (0.5 mL). The vial was then capped, removed from the glovebox, and stirred at 70 °C for 24 h. After this time, 1 mL of a stock solution containing 1,3,5-trimethoxybenzene internal standard in $CDCl_3$ ([0.067 M])was added. The reaction was then filtered through a short plug of celite into an NMR tube. Analysis of the crude using ^1H NMR, with reference to the spectra of pure compounds, showed the formation of **3ma** (72%) and **3oa** (18%).

7.2. Stoichiometric experiments from mono-cyclometallated intermediate using CH_3I

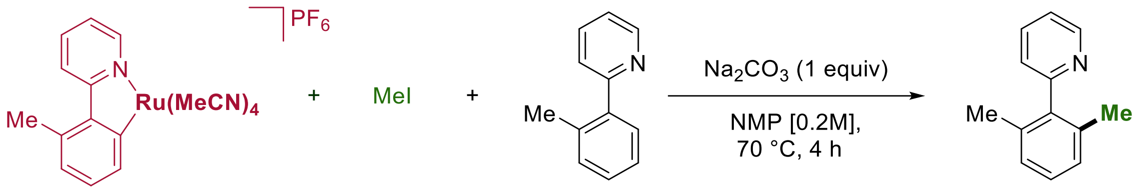

Ru(2-TolPy)		3aa	4aa
0.05 mmol	0.05 mmol	0 mmol	6%
		0.05 mmol	100%

Scheme S11. Stoichiometric ruthenium experiments using CH_3I and complex **Ru(2-TolPy)**

In an argon-filled glove box, 2x oven-dried crimp-cap microwave vials equipped with a magnetic stirring bars were each charged with **Ru(2-TolPy)** (29 mg, 0.05 mmol), methyl iodide (3.00 μL, 0.05 mmol), Na_2CO_3 (5.3 mg, 0.05 mmol), NMP (5 mL) and hexadecane as internal standard. Subsequently, 2-(*o*-tolyl)pyridine **3aa** (8.5 mg, 0.05 mmol)was added to only one vial. Both vials were then capped, removed from the glovebox, and stirred at 70 °C for 4 h. Aliquots were taken, quenched with 1 mL of a 1% pyridine in EtOAc solution, and filtered through a short plug of silica. The aliquots were then analysed by GC-FID using the pre-added hexadecane as the internal standard.

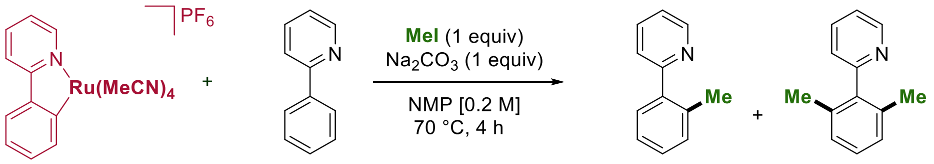

RuPPy 0.05 mmol — **1a** — **3aa** — **4aa**

**1a**	**3aa** yield	**4aa** yield
none	0%	0%
0.05 mmol	88%	5%

Scheme 12. Stoichiometric ruthenium experiments using CH_3I and complex **RuPPy**

In an argon-filled glove box, 2x oven-dried crimp-cap microwave vials equipped with a magnetic stirring bars were each charged with **RuPPy** (27 mg, 0.05 mmol), methyl iodide (3.00

µL, 0.05 mmol), Na_2CO_3 (5.3 mg, 0.05 mmol), NMP (5 mL) and hexadecane as internal standard. Subsequently, 2-phenylpyridine **1a** (7.8 mg, 0.05 mmol) was added to only one vial. Both vials were then capped, removed from the glovebox, and stirred at 70 °C for 4 h. Aliquots were taken, quenched with 1 mL of a 1% pyridine in EtOAc solution, and filtered through a short plug of silica. The aliquots were then analysed by GC-FID using the pre-added hexadecane as the internal standard.

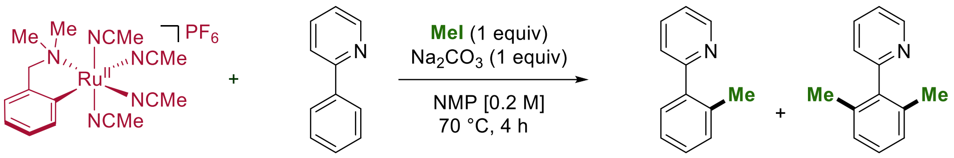

RuBnN **1a** **3aa** **4aa**

**1a**	**3aa** yield	**4aa** yield
0.05 mmol	0%	0%
0.10 mmol	91%	4%

Scheme 13. Stoichiometric ruthenium experiments using CH_3I and complex **RuBnN**

In an argon-filled glove box, 2x oven-dried crimp-cap microwave vials equipped with a magnetic stirring bars were each charged with **RuBnN** (28 mg, 0.05 mmol), methyl iodide (3.00 µL, 0.05 mmol), Na_2CO_3 (5.3 mg, 0.05 mmol), NMP (5 mL) and hexadecane as internal standard. Subsequently, 2-phenylpyridine **1a** (7.8 mg, 0.05 mmol) was added to only one vial. Both vials were then capped, removed from the glovebox, and stirred at 70 °C for 4 h. Aliquots were taken, quenched with 1 mL of a 1% pyridine in EtOAc solution, and filtered through a short plug of silica. The aliquots were then analysed by GC-FID using the pre-added hexadecane as the internal standard.

7.3. Stoichiometric experiments from mono-cyclometallated intermediate using salt 2a

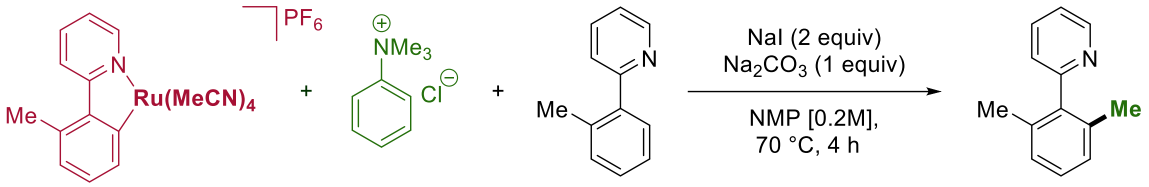

**Ru(2-TolPy)**	**2a**	**3aa**	**4aa**
0.05 mmol	0.05 mmol	0 mmol<br>0.05 mmol	< 0.5%<br>32%

Scheme S14. Stoichiometric ruthenium experiments using ammonium salt **2a.**

In an argon-filled glove box, 2x oven-dried crimp-cap microwave vials equipped with a magnetic stirring bars were charged with **Ru(2-TolPy)** (29 mg, 0.05 mmol), ammonium salt **2a** (8.5 mg, 0.05 mmol), NaI (15 mg 0.05 mmol), Na_2CO_3 (5.3 mg, 0.05 mmol) and NMP (5 mL) containing hexadecane as an internal standard. Subsequently, 2-(*o*-tolyl)pyridine **3aa** (28.2 mg, 0.05 mmol) was added to only one vial. Both vials were then capped, removed from the glovebox, and stirred at 70 °C for 4 h. Aliquots were taken, quenched with 1 mL of a 1% pyridine in EtOAc solution, and filtered through a short plug of silica. The aliquots were then analysed by GC-FID using the pre-added hexadecane as the internal standard.

8. Kinetic Experiments

8.1. Same excess experiment

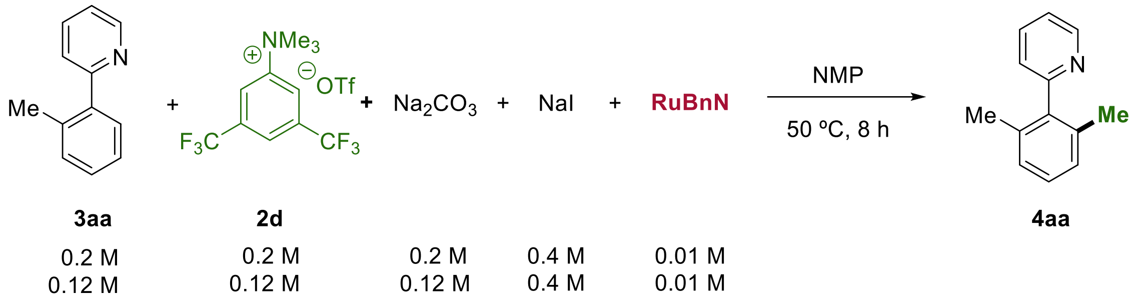

Scheme S15. Same excess experiment

In an argon-filled glove box, 2x oven-dried crimp-cap microwave vials equipped with a magnetic stirring bars were charged with NaI (60 mg, 0.40 mmol) in NMP (500 μL) containing hexadecane as an internal standard. Then, different amounts of the rest of substrates were added to the vials. For vial A: 2-(*o*-tolyl)pyridine **3aa** (34 mg, 0.20 mmol), ammonium salt **2d** (84 mg, 0.20 mmol) and Na_2CO_3 (21 mg, 0.20 mmol. For vial B: 2-(*o*-tolyl)pyridine **3aa** (20 mg, 0.12 mmol), ammonium salt **2d** (51 mg, 0.12 mmol) and Na_2CO_3 (13 mg, 0.12 mmol). The reaction was then heated at 50 °C inside the glove box for 20 minutes with a stirring rate of 500 rpm, before a solution of **RuBnN** (5.4 mg, 0.01 mmol, 5 mol %) in 500 μL of NMP was added at t = 0 min to start the reaction. Aliquots of approximately 20 μL were then taken throughout the first 8 h of the reaction at specified time points. Each aliquot was added to approximately 0.5 mL of a solution of 1% pyridine in EtOAc (v/v), before being passed through a short plug of silica, using EtOAc as eluent into a GC vial. The reaction was then monitored by GC-FID, using the pre-added hexadecane as internal standard.

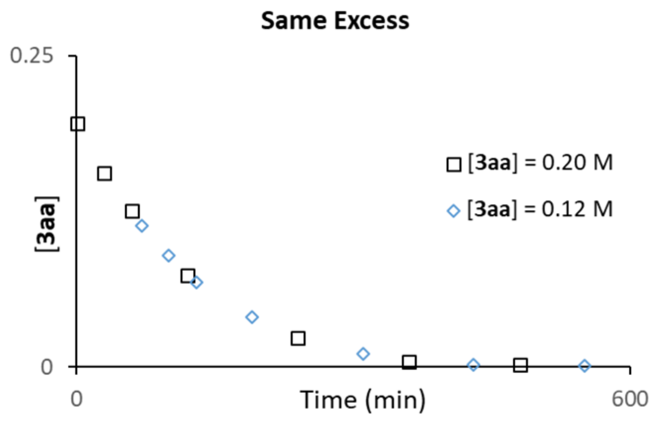

Figure S1. Same excess kinetic profile.

The overlap between the temporal reaction profiles with concentrations 0.20 M and 0.12 M suggests that there is neither catalyst decomposition nor product inhibition.

8.2. General Procedure for kinetic experiments employing 2-(o-tolyl)pyridine 3aa and ammonium salt 2d

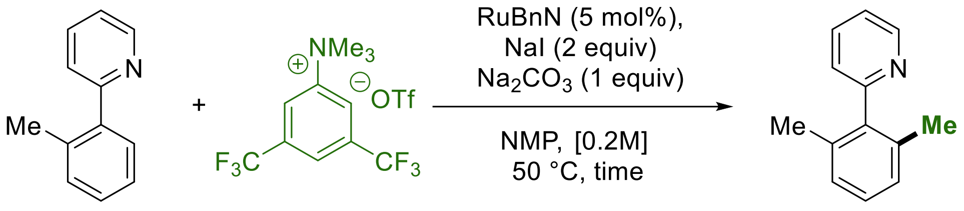

3aa 0.2 mmol **2d** 0.2 mmol **4aa**

Scheme S16. General Procedure for kinetic experiments.

2d (84 mg, 0.20 mmol, 1 equiv), NaI (60 mg, 0.40 mmol, 2 equiv) and Na_2CO_3 (20 mg, 0.20 mmol, 1 equiv) were weighed out in an argon-filled glovebox into a 10 mL microwave vial. A stock solution of **3aa** and hexadecane internal standard in NMP was prepared. This was added to the vial via microsyringe, with the vial subsequently capped with a suba-seal. The reaction was heated at 50 °C inside the glove box for 20 minutes with a stirring rate of 500 rpm, before a solution of **RuBnN** (5.4 mg, 0.01 mmol, 5 mol %) in 500 µL of NMP was added at t = 0 min to start the reaction. Aliquots of approximately 20 µL were then taken throughout the first 8 h of the reaction at specified time points. Each aliquot was added to approximately 0.5 mL of a solution of 1% pyridine in EtOAc (v/v), before being passed through a short plug of silica, using EtOAc as eluent, into a GC vial for analysis. The reaction was then monitored by GC-FID, using the pre-added hexadecane as the internal standard.

Determination of Order in Catalyst

The order in catalyst has been determined using normalized time scale analysis. Reactions were carried out with different concentrations of catalyst (2.5 and 5.0 mol %) and their temporal profiles were normalized according to the catalyst loading raised to the power of the order in the catalyst. All the resulting curves were plotted together and the correct order in catalyst is the one that causes the curves to overlay.

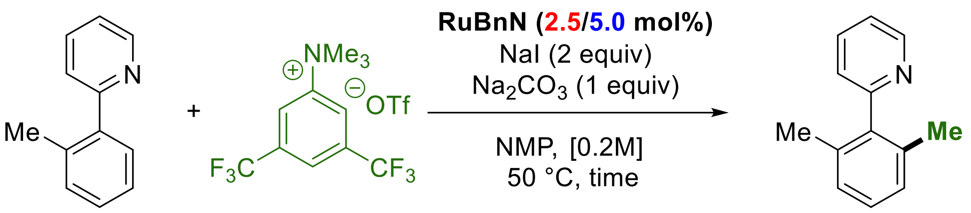

3aa 0.2 mmol   **2d** 0.2 mmol   **4aa**

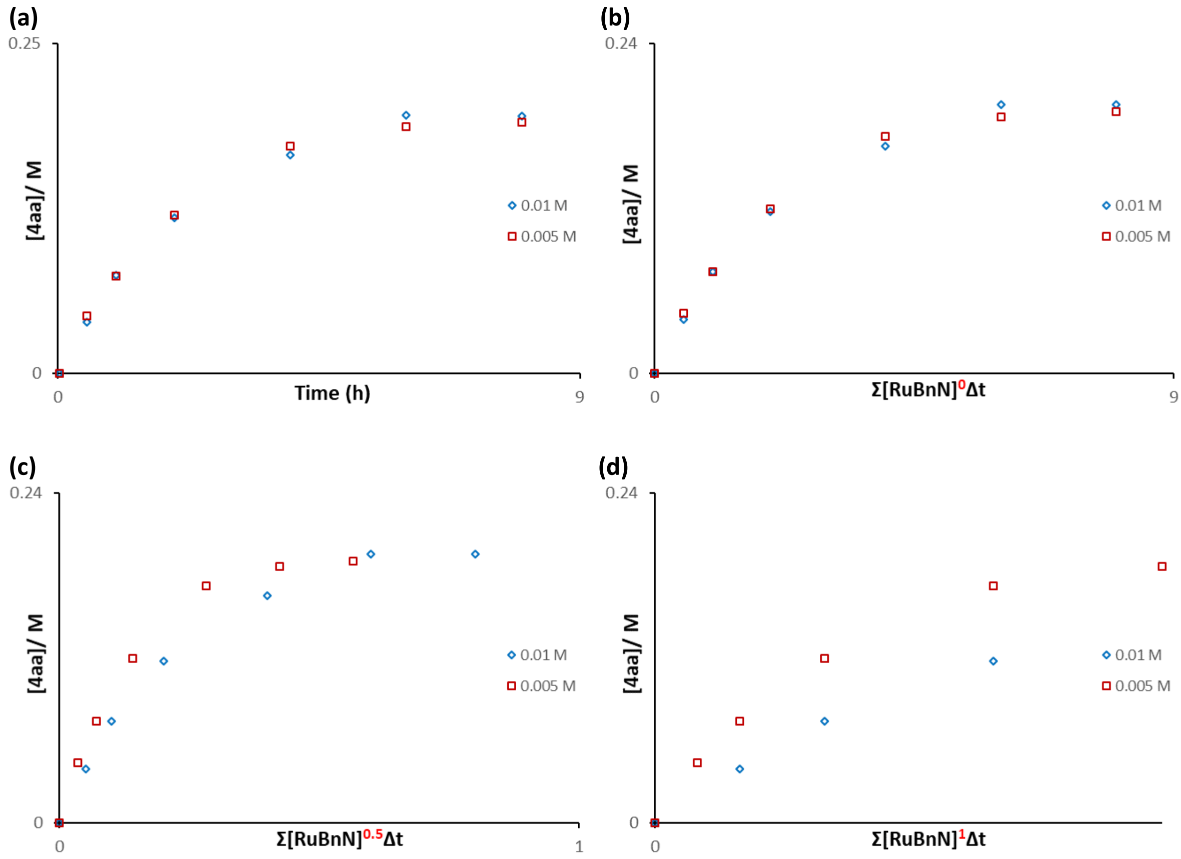

Figure S2. Determination of order in catalyst.

(a) Temporal reaction profiles of reactions carried out with 5/2.5 mol % of [**RuBnN**]; (b) Normalized time scale profiles for order 0 in [**RuBnN**]; (c) Normalized time scale profiles for order 0.5 in [**RuBnN**]; (d) Normalized time scale profiles for order 1.0 in [**RuBnN**].

The overlap between the temporal reaction profiles with catalyst loadings of 5.0 and 2.5 mol % suggests that the order in [**RuBnN**] is 0 at these concentrations.

Determination of Order in Reagents

Determination of the order in 3aa

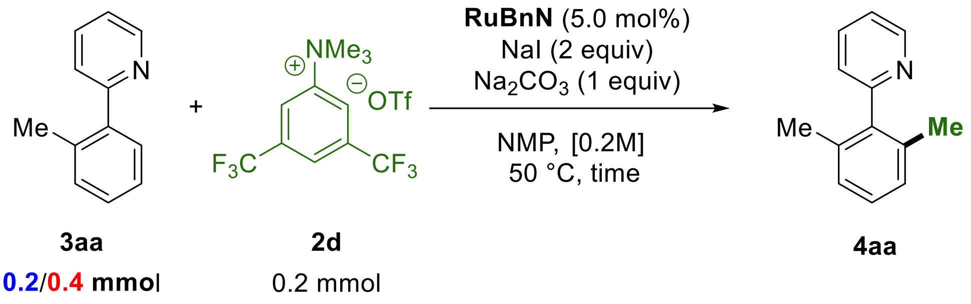

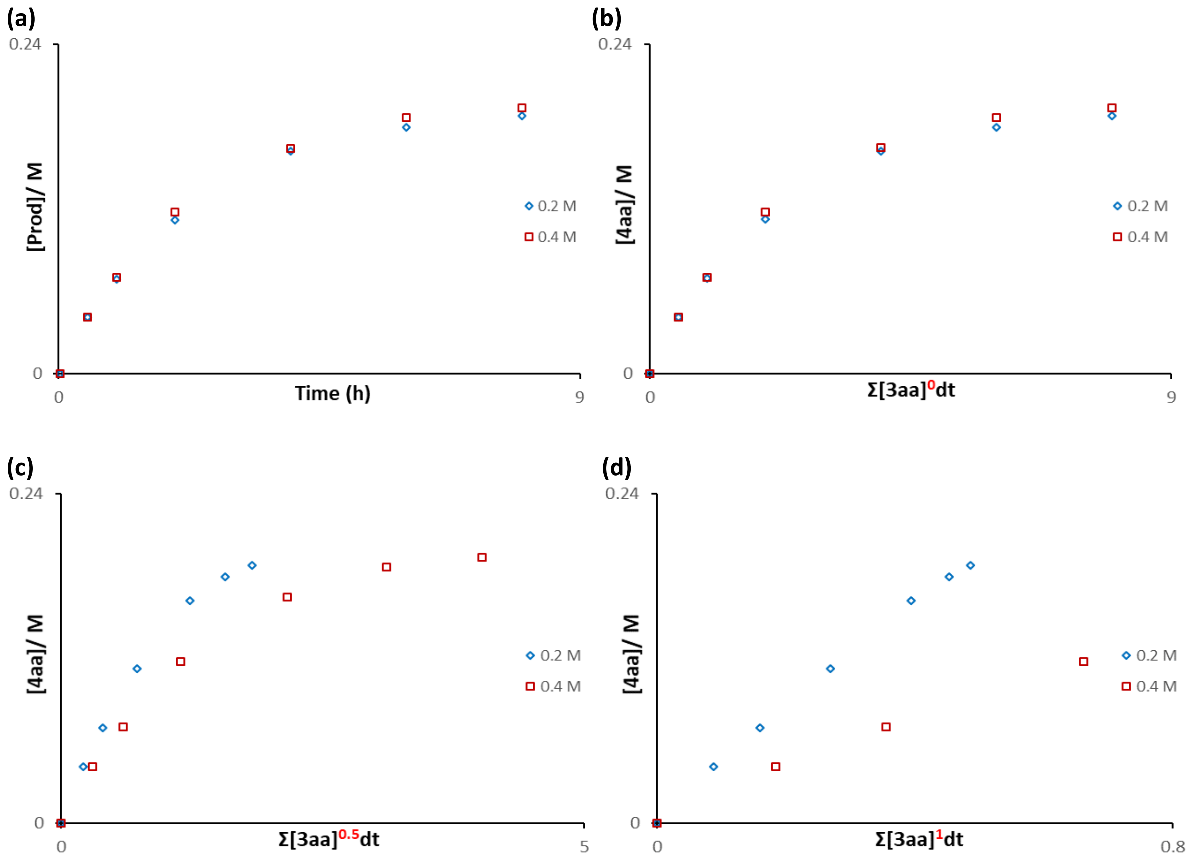

Figure S3. Determination of order in **3aa**.

(a) Temporal reaction profiles of reactions carried out with 0.2/0.4 mmol of **3aa**; (b) Normalized time scale profiles for order 0 in [**3aa**]; (c) Normalized time scale profiles for order 0.5 in [**3aa**]; (d) Normalized time scale profiles for order 1.0 in [**3aa**].

The overlap between normalised time scale reaction profiles for these two reactions with differing concentrations of **3aa** shows an order of 0.

Determination of the order in 2d

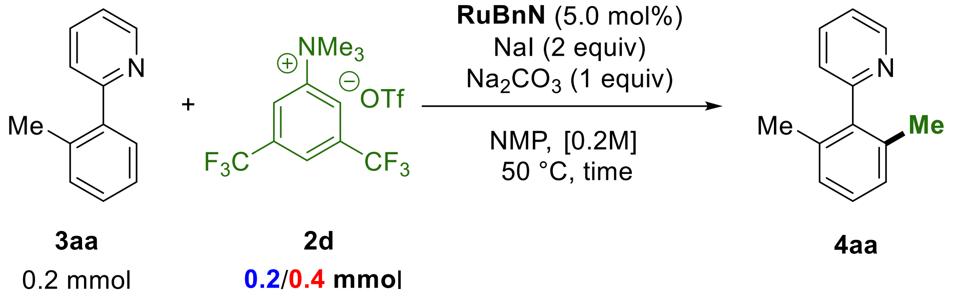

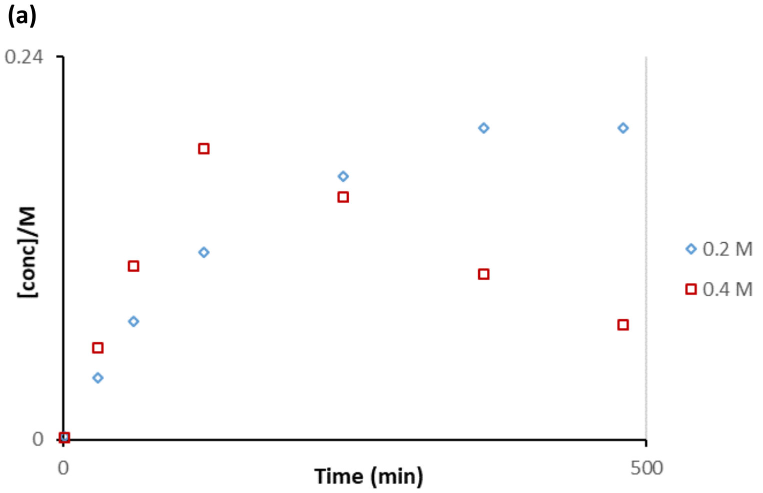

Figure S4. Kinetic profile using 1 and 2 equivalent of ammonium salt **2d**.

The reaction using 2 equivalents of ammonium salt was much faster in the first 2 hours. However, both product and recovery of the reaction started dropping due to *N*-methylation of product **4aa**. To calculate the order in ammonium salt, sub-stoichiometric amounts of salt had to be used to avoid the over methylation reaction in the nitrogen.

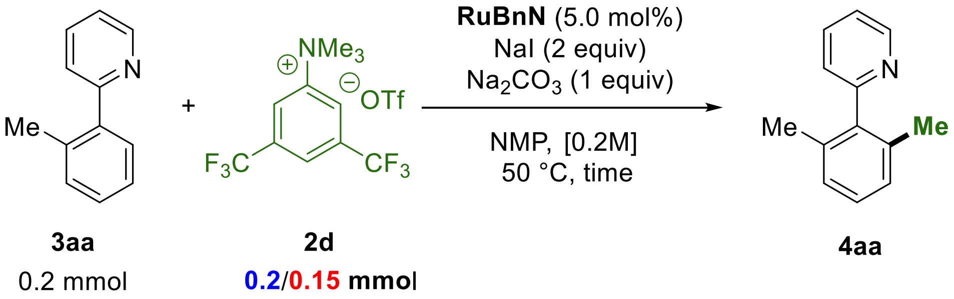

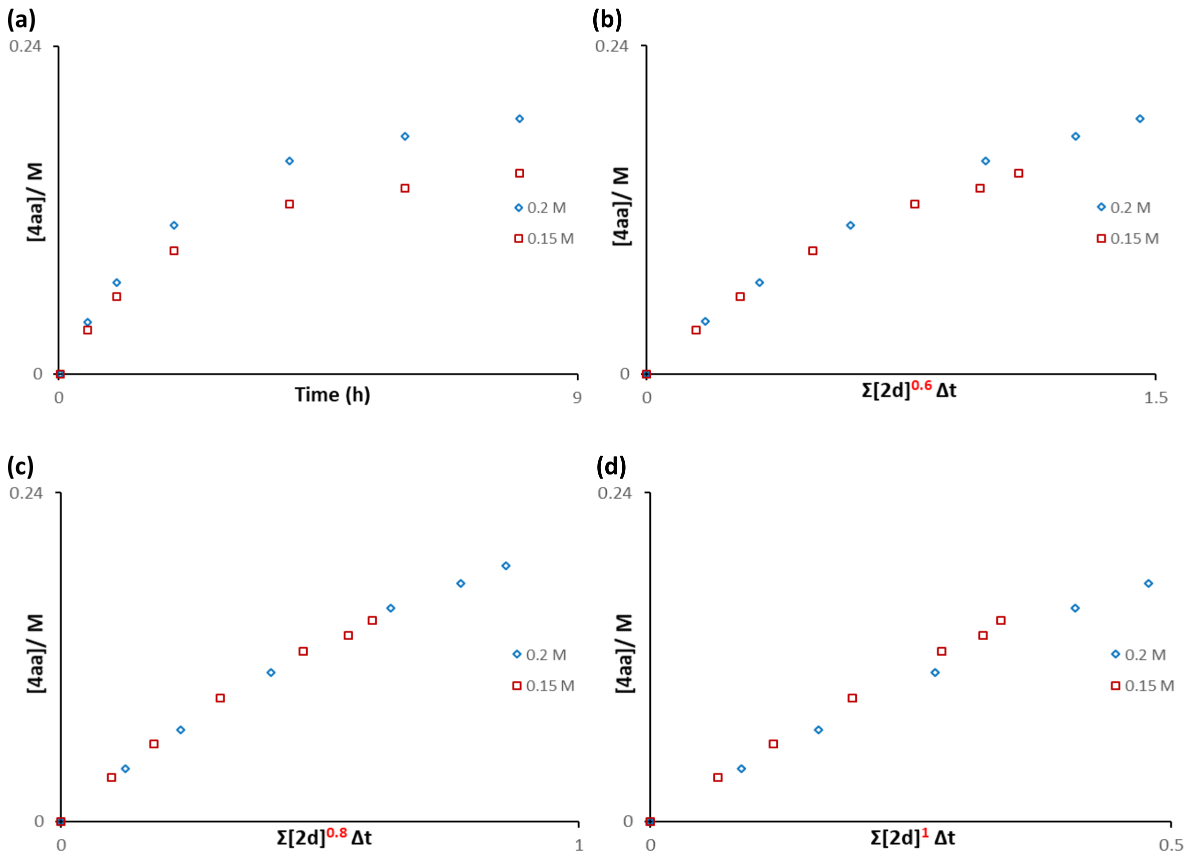

Figure S5. Determination of order in **2d**.

(a) Temporal reaction profiles of reactions carried out with 0.2/0.15 mmol of **2d**; (b) Normalized time scale profiles for order 0.6 in [**2d**]; (c) Normalized time scale profiles for order 0.8 in [**2d**]; (d) Normalized time scale profiles for order 1.0 in [**2d**].

The overlap between normalised time scale reaction profiles for these two reactions with differing concentrations of **3aa** shows an order of 0.8.

Determination of the order in NaI

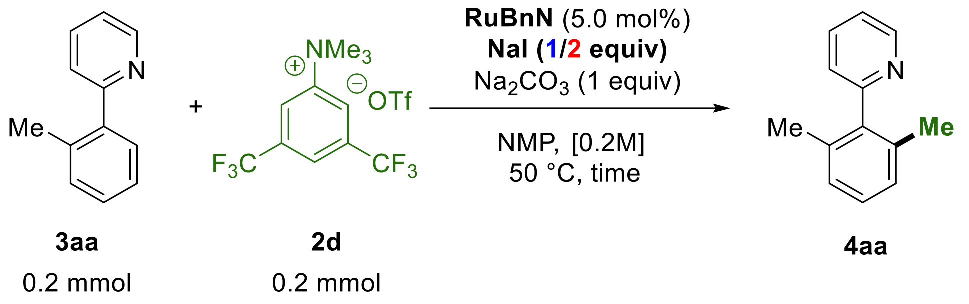

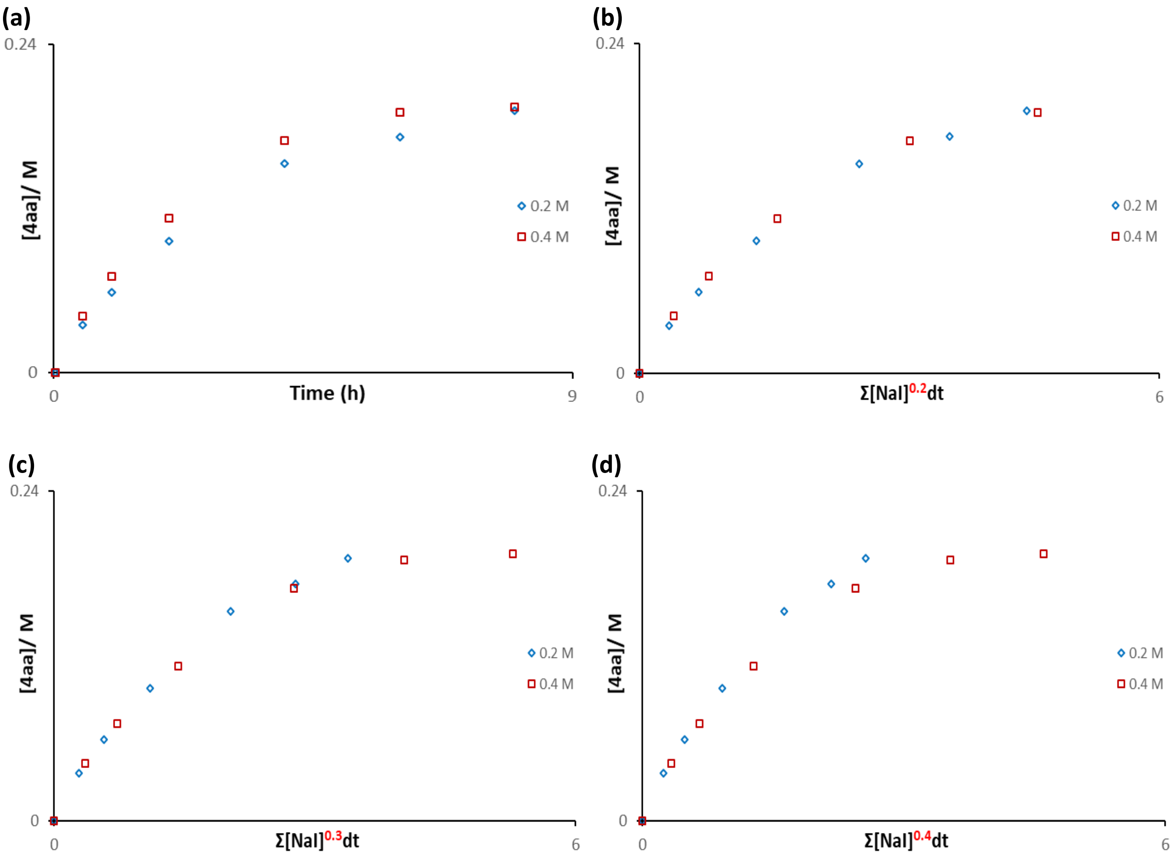

Figure S6. Determination of order in NaI.

(a) Temporal reaction profiles of reactions carried out with 0.2/0.4 mmol of NaI; (b) Normalized time scale profiles for order 0.2 in [NaI]; (c) Normalized time scale profiles for order 0.3 in [NaI]; (d) Normalized time scale profiles for order 0.4 in [NaI].

The overlap between normalised time scale reaction profiles for these two reactions with differing concentrations of **3aa** shows an order of 0.3.

Determination of the order in Base

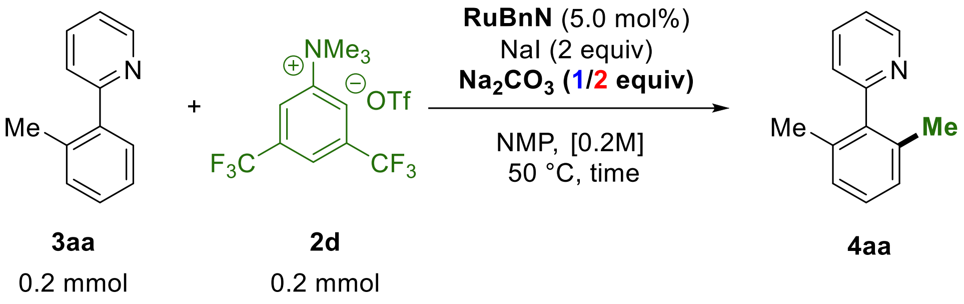

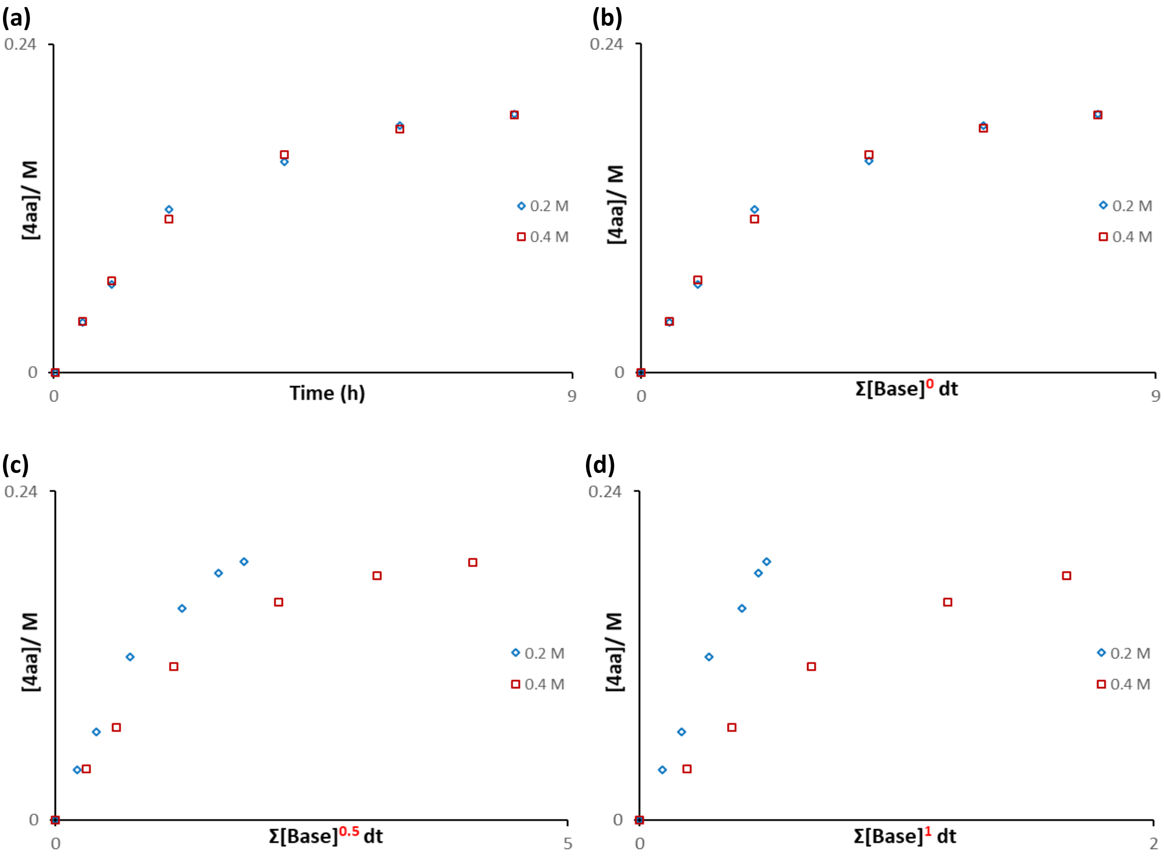

Figure S7. Determination of order in Base.

(a) Temporal reaction profiles of reactions carried out with 0.2/0.4 mmol of Na_2CO_3; (b) Normalized time scale profiles for order 0 in [Na_2CO_3]; (c) Normalized time scale profiles for order 0.5 in [Na_2CO_3]; (d) Normalized time scale profiles for order 1.0 in [Na_2CO_3].

The overlap between normalised time scale reaction profiles for these two reactions with differing concentrations of **3aa** shows an order of 0.

8.3. Determination of Kinetic Isotope Effect (KIE)

KIE in arene moiety

Two identical reactions were carried out in parallel (detailed in scheme below), set up identically exception of the deuterated/non-deuterated substrate (**1r**/***d*₅-1r**). Ammonium salt **2d** (84 mg, 0.20 mmol, 1 equiv), NaI (60 mg, 0.40 mmol, 2 equiv) and Na_2CO_3 (20 mg, 0.20 mmol, 1 equiv) were weighed out into a 10 mL microwave vial in an argon-filled glovebox. Stock solutions in NMP were prepared for **1r**/**d_5-1r** and hexadecane as internal standard, and these were added to the appropriate vial via microsyringe. The reaction was then heated at 50 °C inside the glove box for 20 min with a stirring rate of 500 rpm, after which, a solution of **RuBnN** (5.4 mg, 0.01 mmol, 5 mol %) in 500 µL of NMP was added at t = 0 minutes. Aliquots of approximately 50 µL were then taken throughout the first 8 h of the reaction at specified time points. Each aliquot was added to approximately 0.5 mL of a solution of 1% pyridine in EtOAc (v/v), before being passed through a short plug of silica, using EtOAc as eluent, into a GC vial. The reaction was then monitored by GC-FID, using the pre-added hexadecane as internal standard.

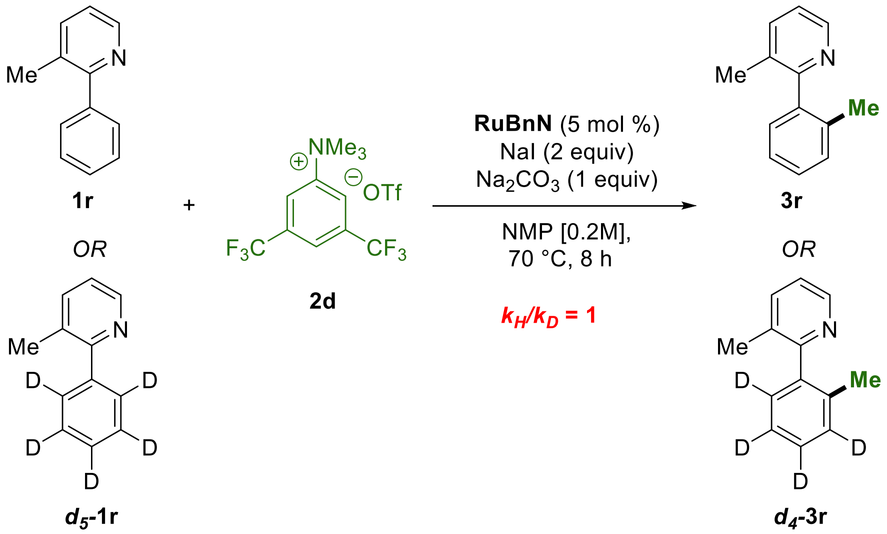

Scheme 17. Parallel reactions for determination of kinetic isotope effect.

The value for the KIE has been determined using normalized time scale analysis. The reactions were carried out and their temporal profiles were normalized according to the KIE value. All the resulting curves were plotted together and the correct KIE is the one that causes the curves to overlay.

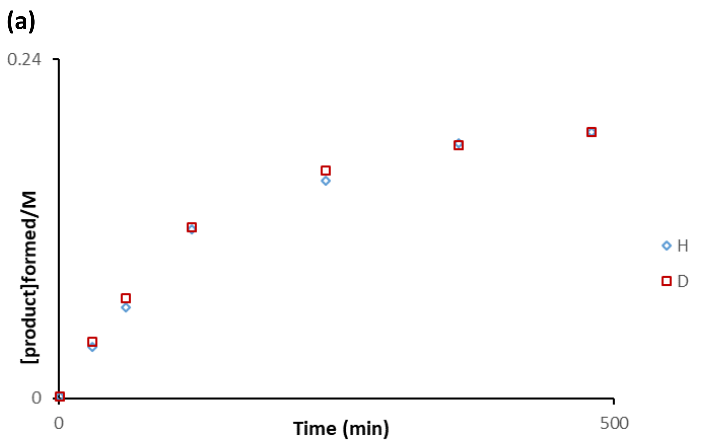

Figure S8. Determination of kinetic isotope effect.

(a) Temporal reaction profiles of reactions carried out with **1r** and ***d*$_5$-1r**

KIE in ammonium salt

Two identical reactions were carried out in parallel (detailed in scheme below), set up identically with the exception of the deuterated/non-deuterated ammonium salt. **2d** or **2e** (84 or 86 mg respectively, 0.20 mmol, 1 equiv), NaI (60 mg, 0.40 mmol, 2 equiv) and Na_2CO_3 (20 mg, 0.20 mmol, 1 equiv) were weighed out in the glovebox into a 10 mL microwave vial. A stock solution in NMP was prepared for **3aa** and hexadecane as internal standard, and this was added to the vials via microsyringe. The reaction was then heated at 50 °C inside the glove box for 20 min with a stirring rate of 500 rpm, before a solution of **RuBnN** (5.4 mg, 0.01 mmol, 5 mol %) in 500 μL of NMP was added at t = 0 minutes. Aliquots of approximately 50 μL were then taken throughout the first 8 h of the reaction at specified time points. Each aliquot was added to approximately 0.5 mL of a solution of 1% pyridine in EtOAc (v/v), before being passed through a short plug of silica, using EtOAc as eluent, into a GC vial. The reaction was then monitored by GC-FID, using the pre-added hexadecane as internal standard.

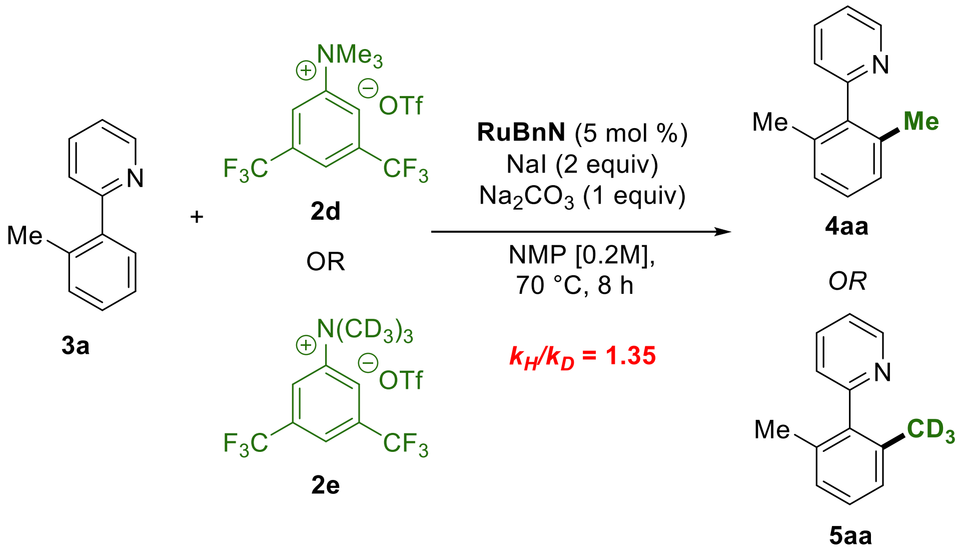

Scheme 18. Parallel reactions for determination of kinetic isotope effect.

The value for the KIE has been determined using normalized time scale analysis. The reactions were carried out and their temporal profiles were normalized according to the KIE value. All

the resulting curves were plotted together and the correct KIE is the one that causes the curves to overlay.

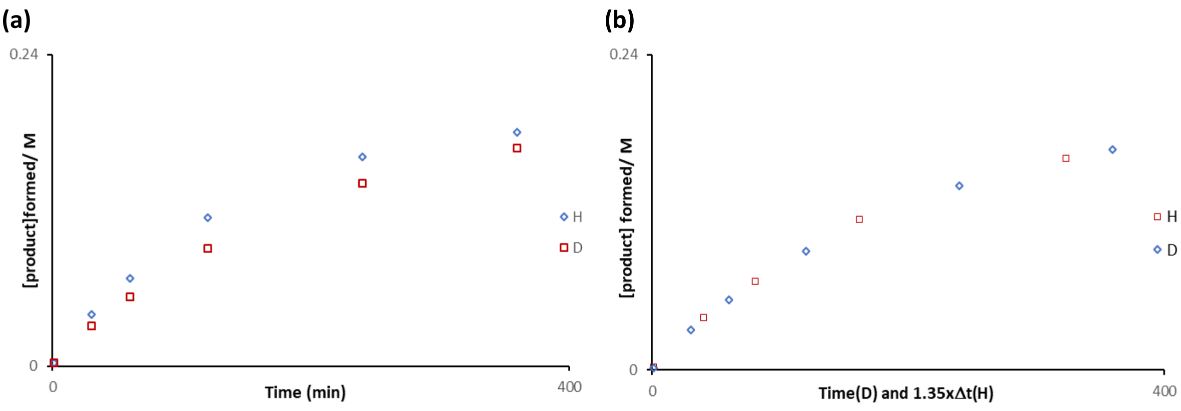

Figure S9. Determination of kinetic isotope effect.

(a) Temporal reaction profiles of reactions carried out with 1s and d5-1s; (b) Normalized time scale profiles for KIE 1.35.

9. NMR Spectra

Figure S10. ^{1}H NMR (400 MHz, $CDCl_3$) of *N,N*-Dimethyl-3,5-bis(trifluoromethyl)aniline.

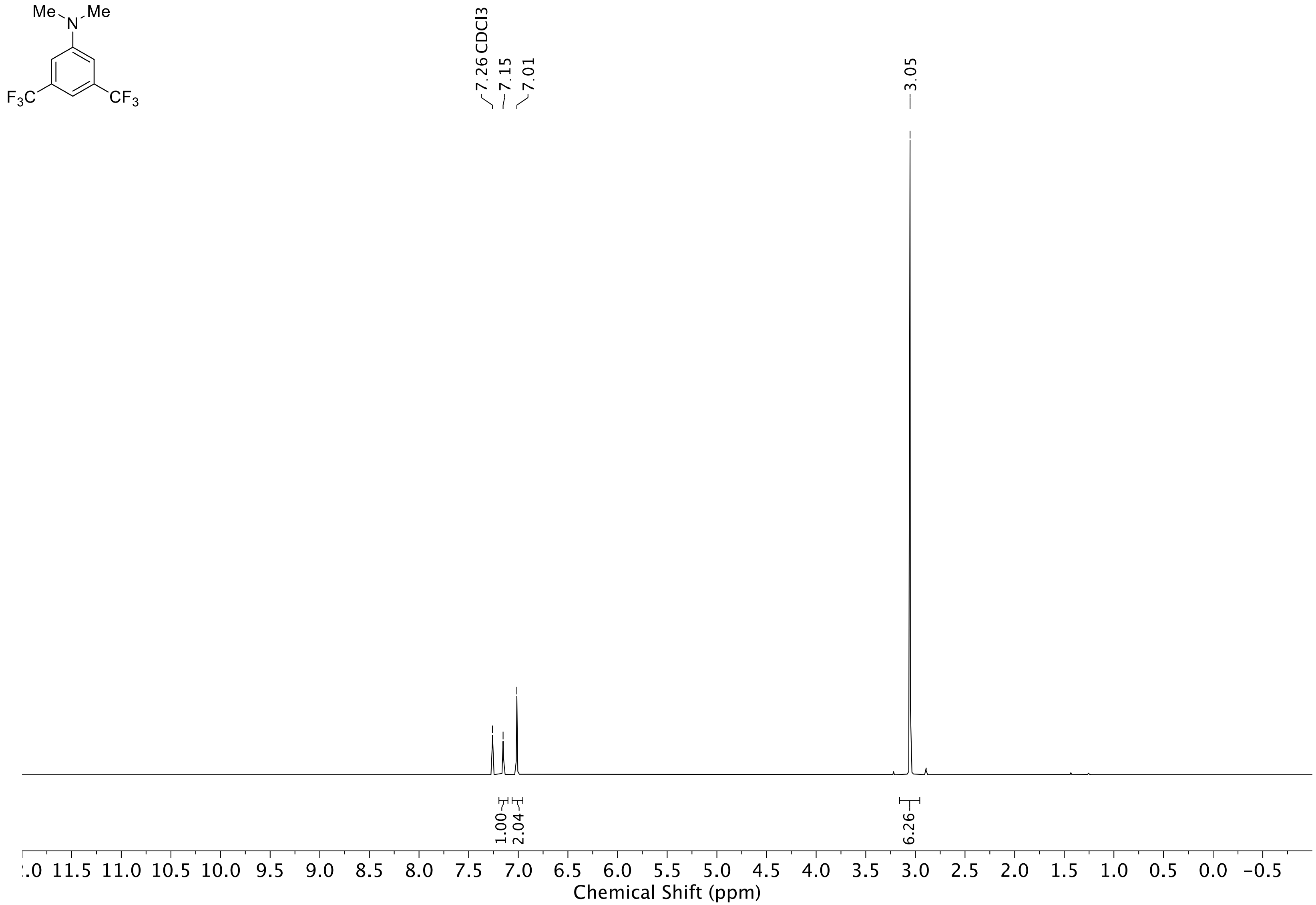

Figure S11. ^{13}C NMR (101 MHz, CDCl$_3$) of *N,N*-Dimethyl-3,5-bis(trifluoromethyl)aniline.

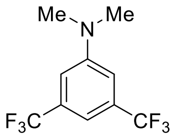

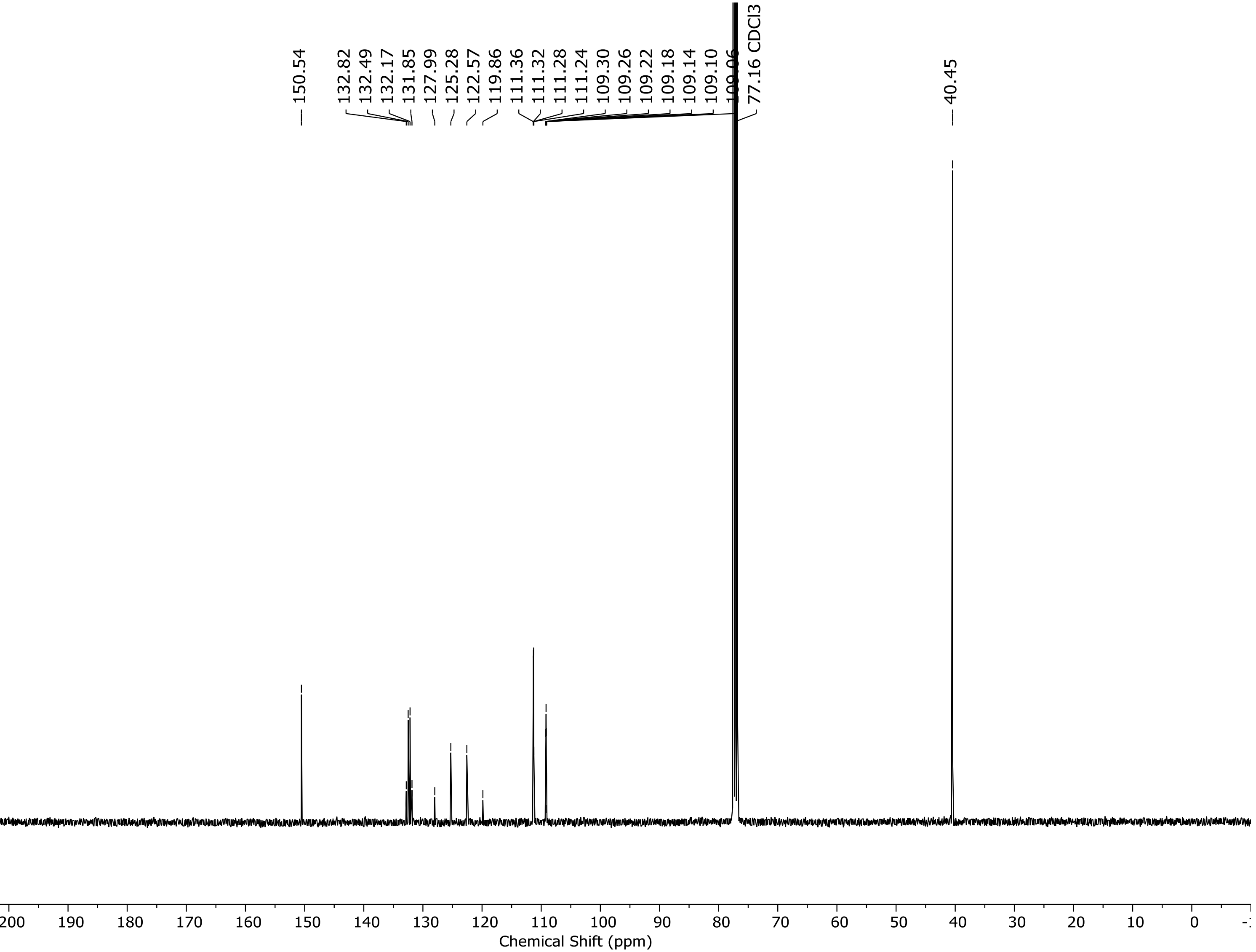

Figure S12. ^{19}F NMR (376 MHz, $CDCl_3$) of *N,N*-Dimethyl-3,5-bis(trifluoromethyl)aniline.

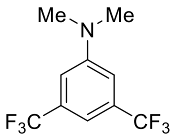

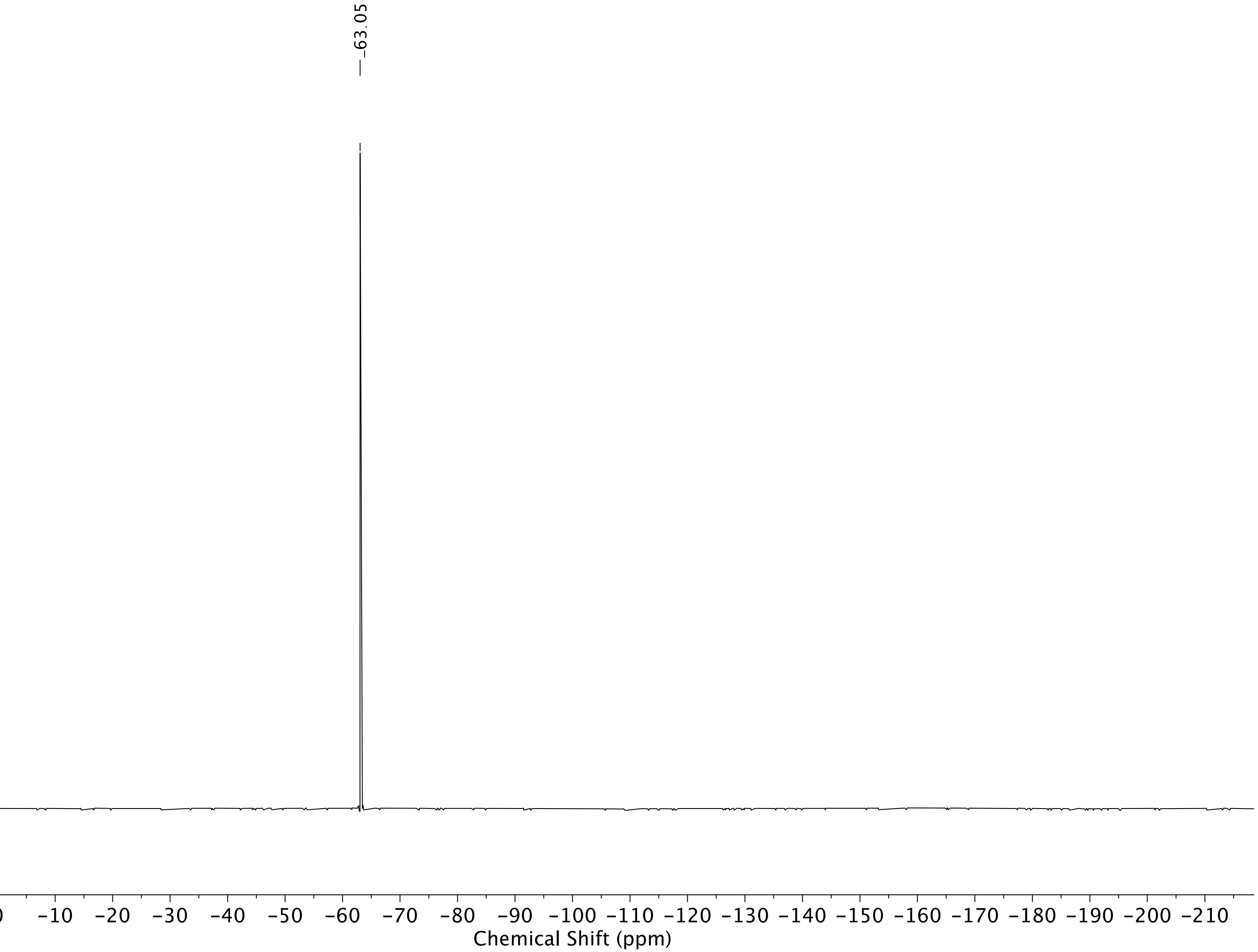

Figure S13. ^{1}H NMR (400 MHz, $(CD_3)_2CO$) of **2d**

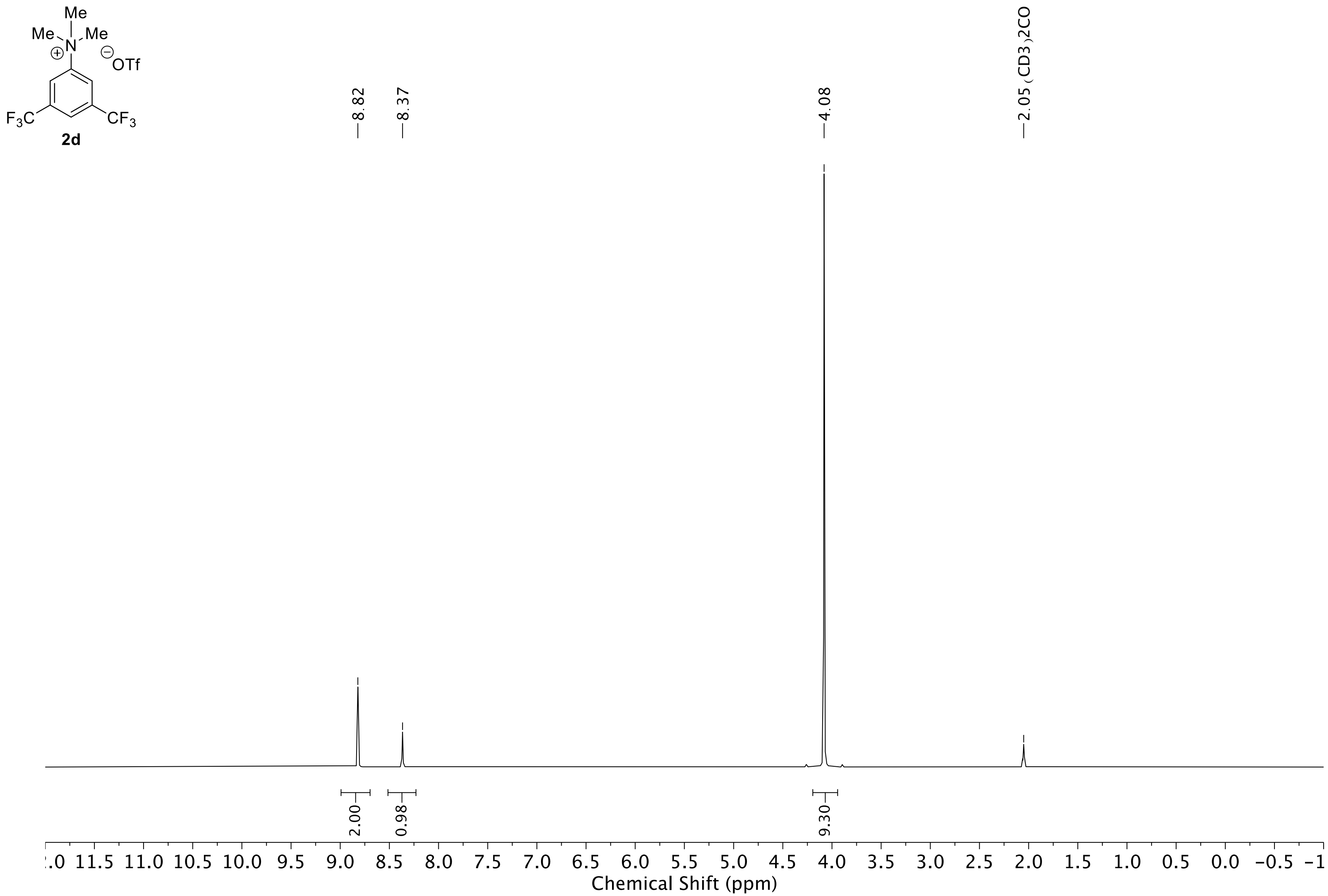

Figure S14. ^{13}C NMR (126 MHz, $(CD_3)_2CO$) of **2d**

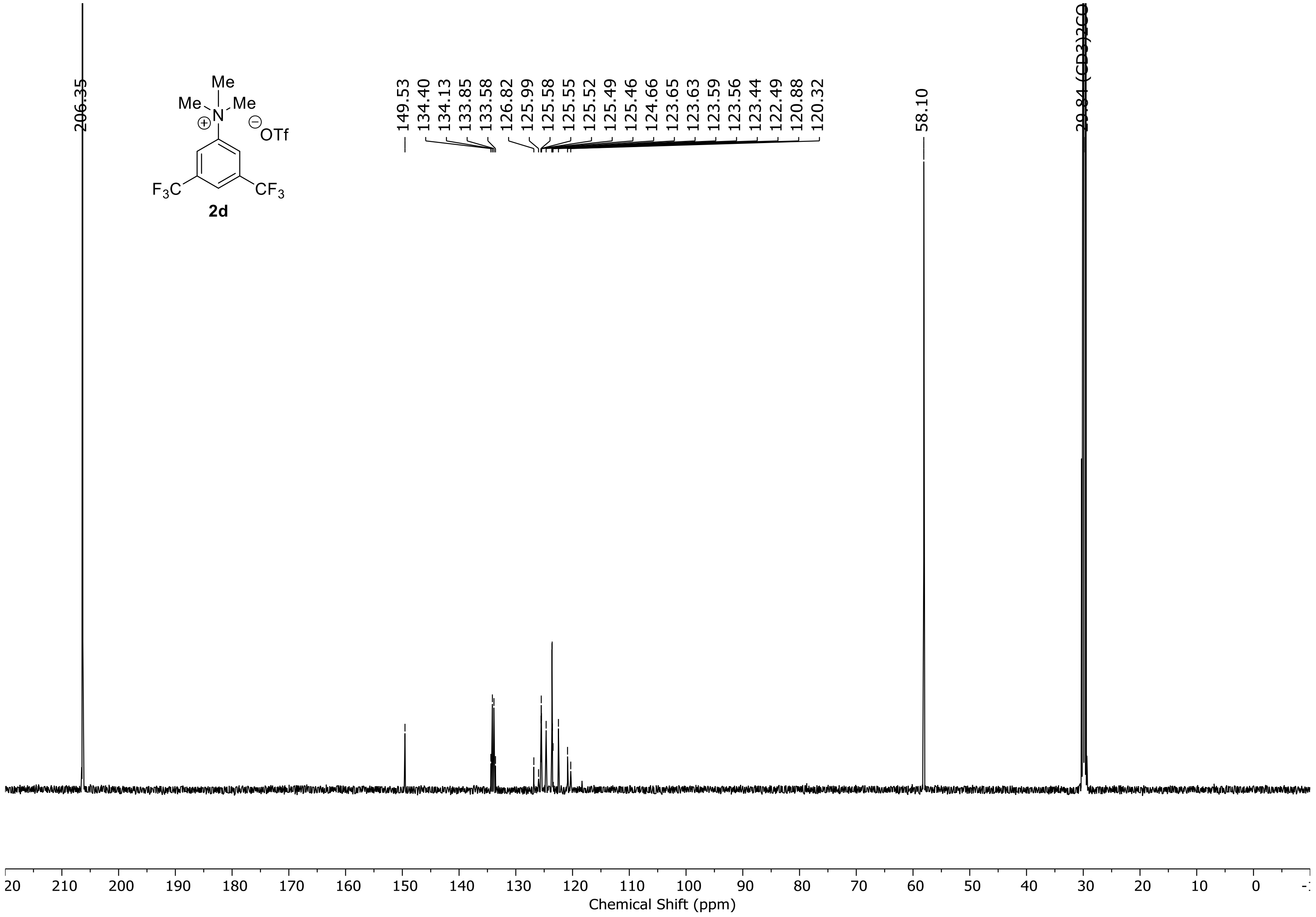

Figure S15. ^{19}F NMR (471 MHz, $(CD_3)_2CO$) of **2d**

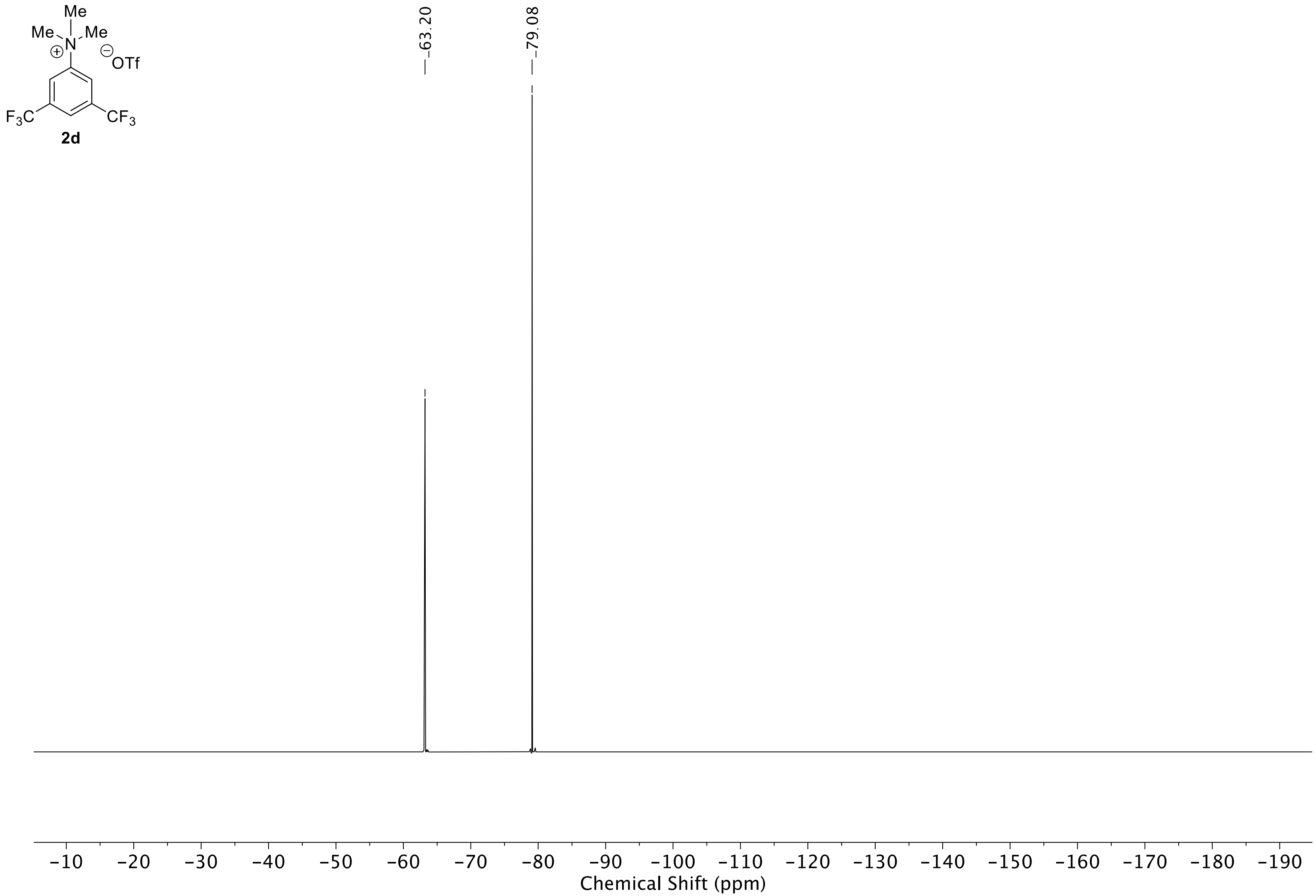

Figure S16. ^{1}H NMR (400 MHz, $CDCl_3$) of *N,N*-bis(trideuteromethyl)-3,5-bis(trifluoromethyl)aniline

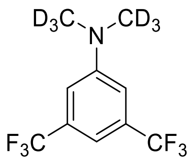

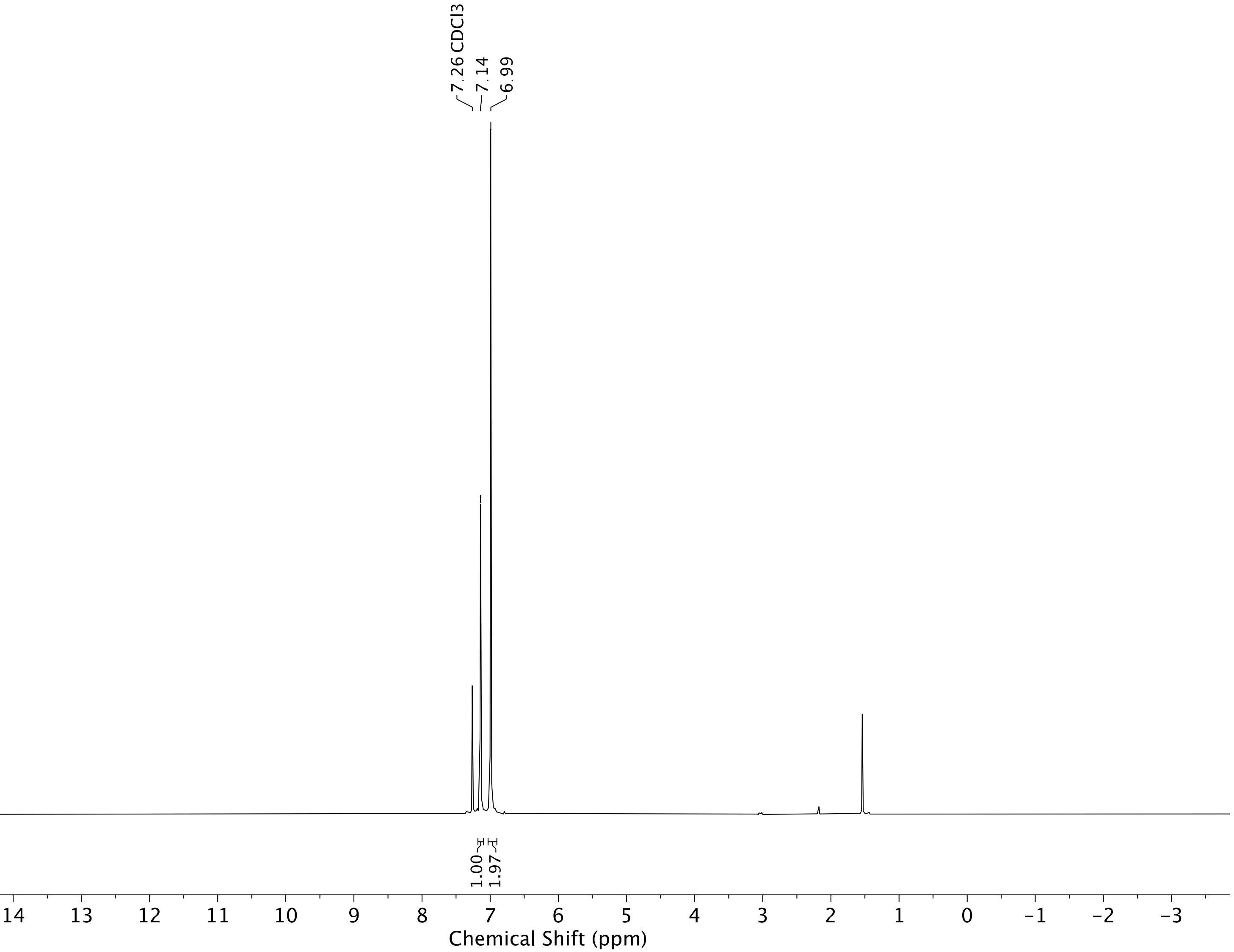

Figure S17. ^{13}C NMR (126 MHz, $CDCl_3$) of *N,N*-bis(trideuteromethyl)-3,5-bis(trifluoromethyl)aniline

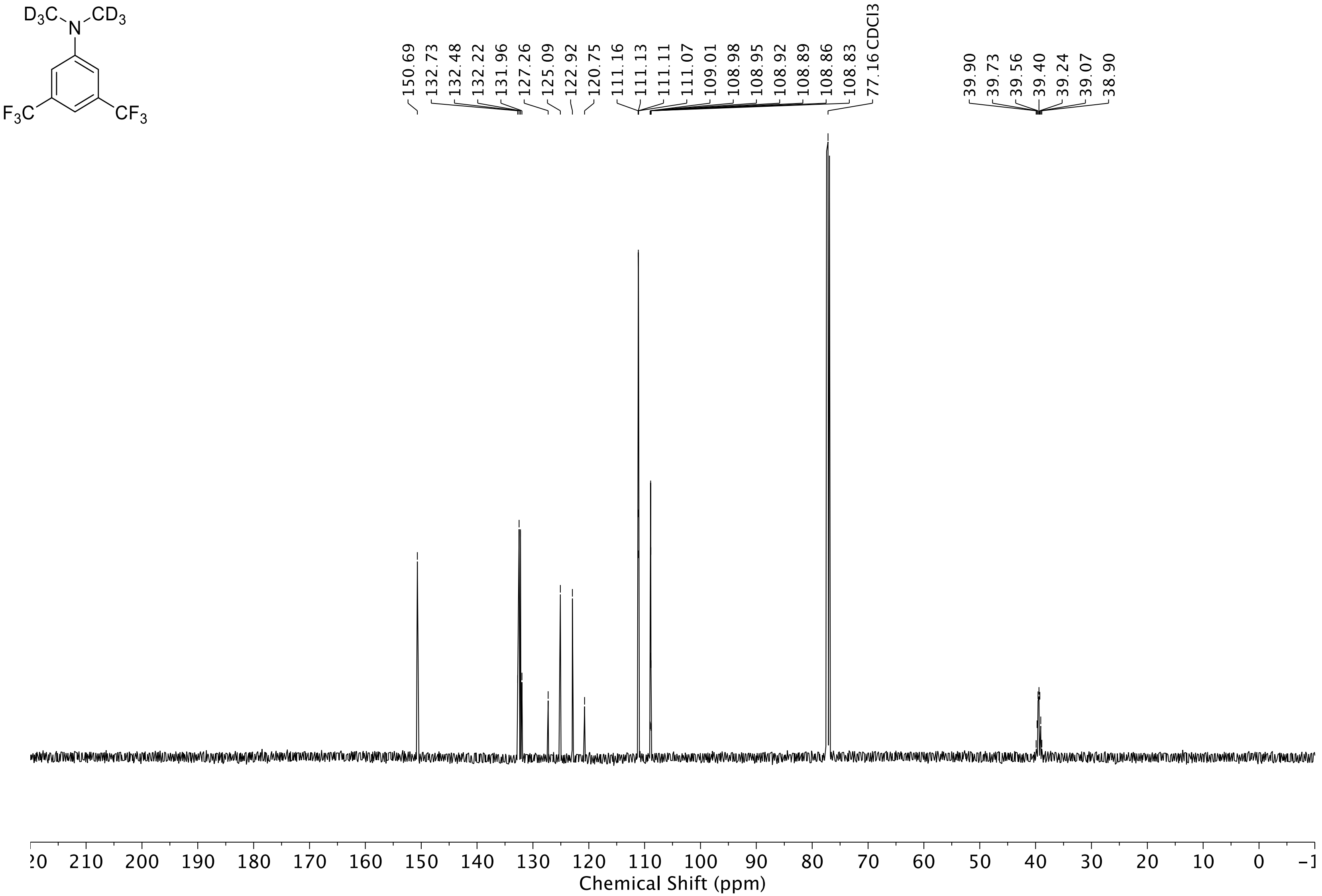

Figure S18. ^{19}F NMR (376 MHz, $CDCl_3$) of *N,N*-bis(trideuteromethyl)-3,5-bis(trifluoromethyl)aniline

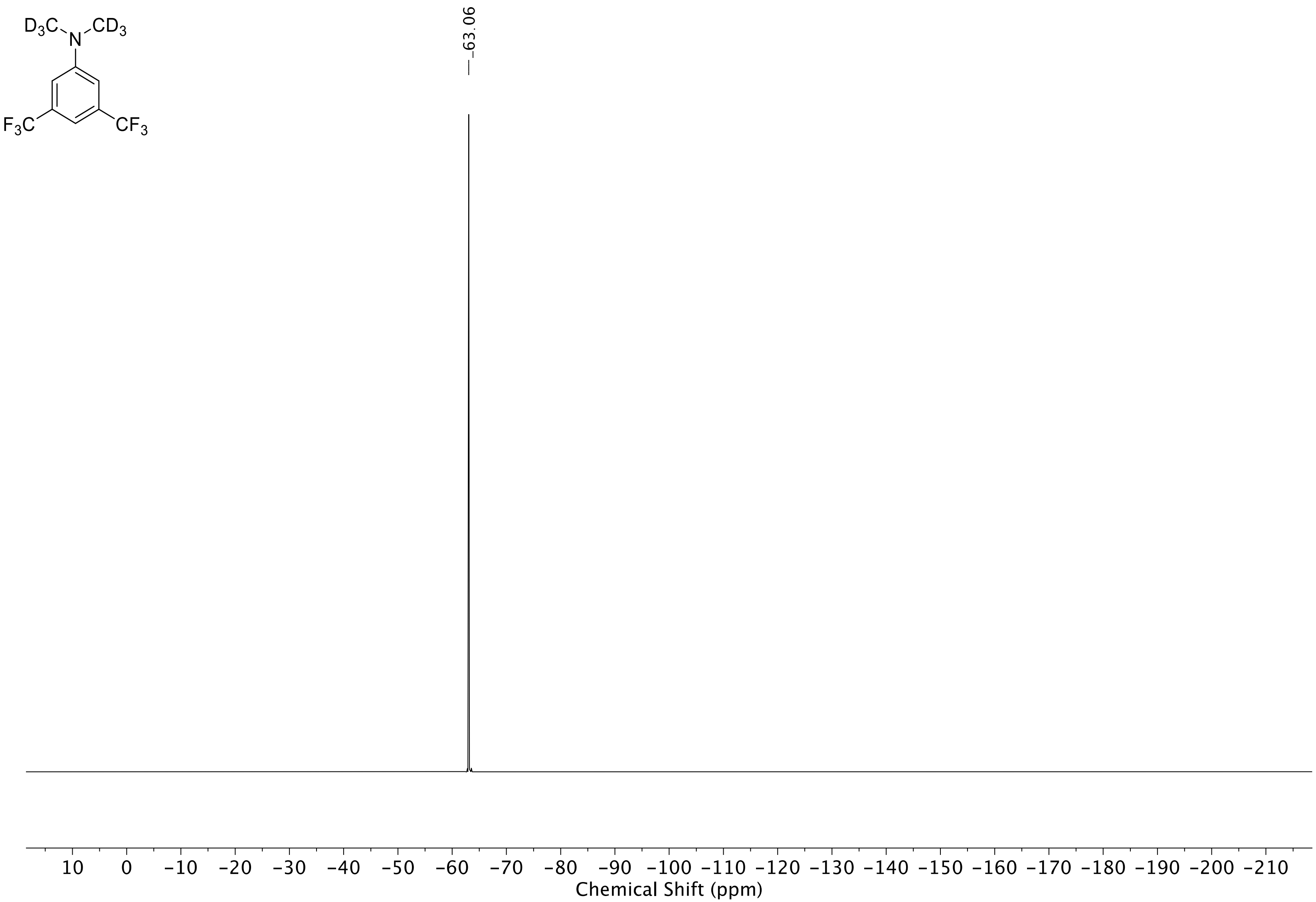

Figure S19. ^2H NMR (61 MHz, $CDCl_3$) of *N,N*-bis(trideuteromethyl)-3,5-bis(trifluoromethyl)aniline

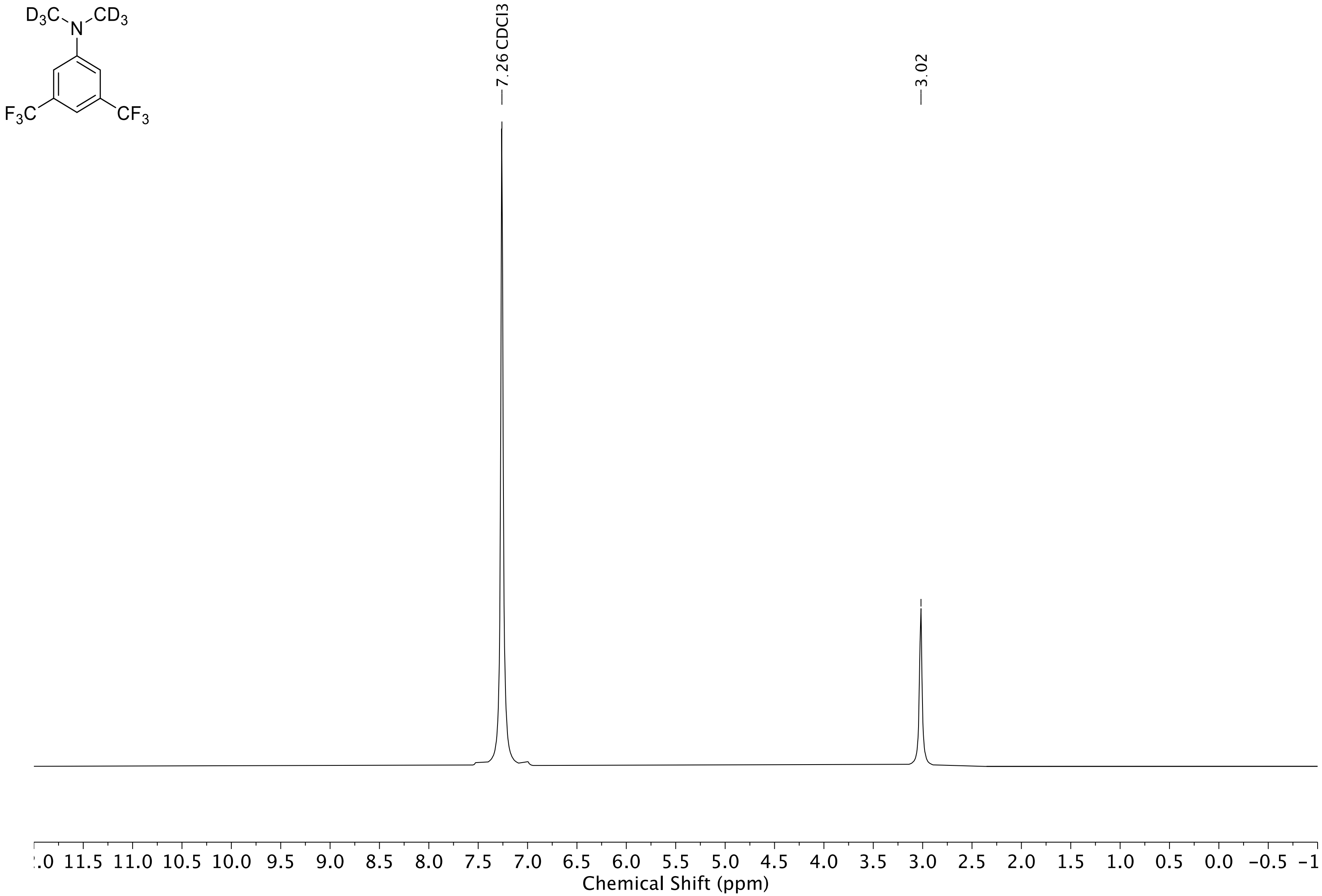

Figure S20. ^{1}H NMR (400 MHz, $(CD_3)_2CO$) of **2e**.

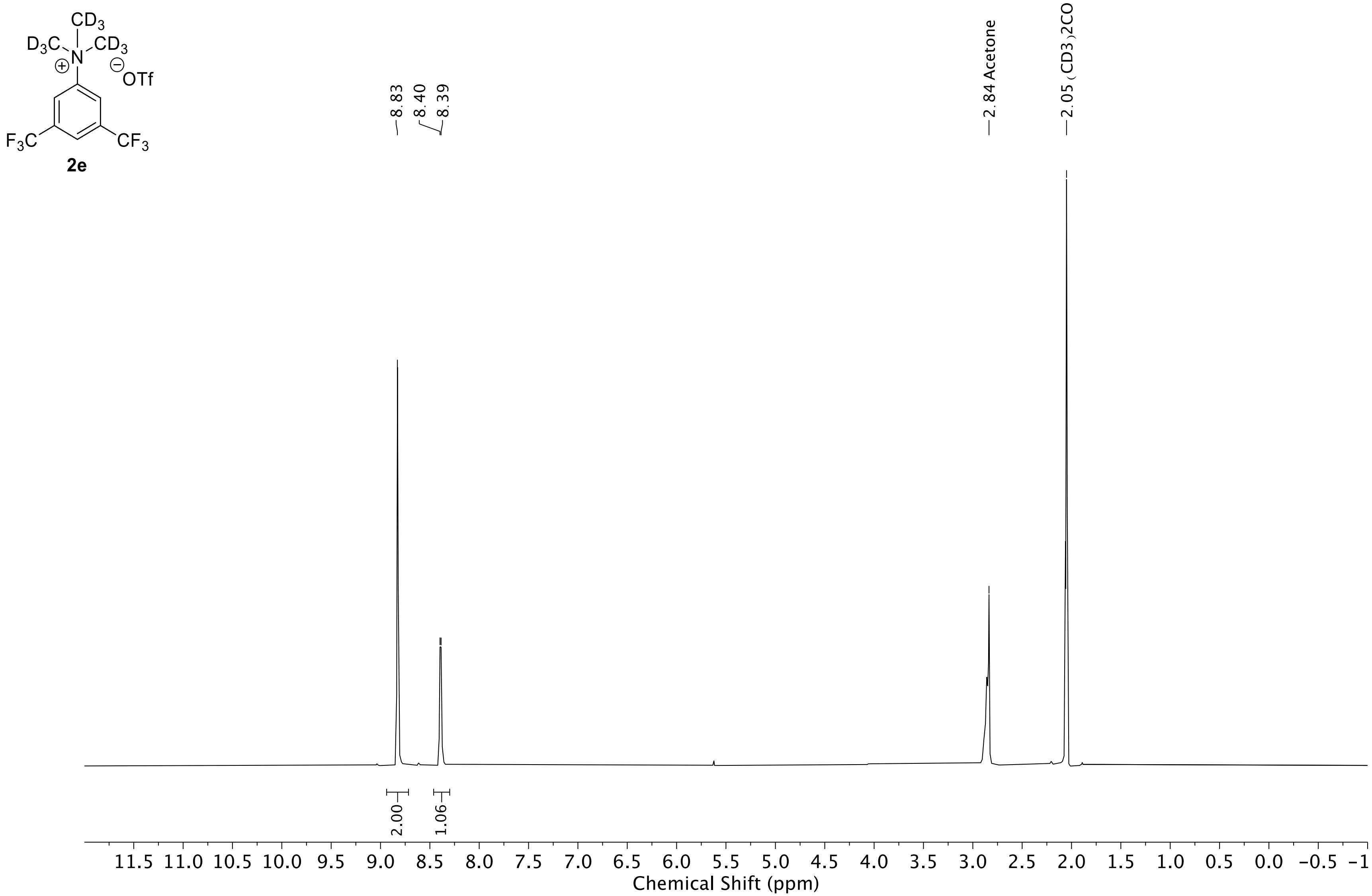

Figure S21. ^{13}C NMR (126 MHz, $(CD_3)_2CO$) of **2e.**

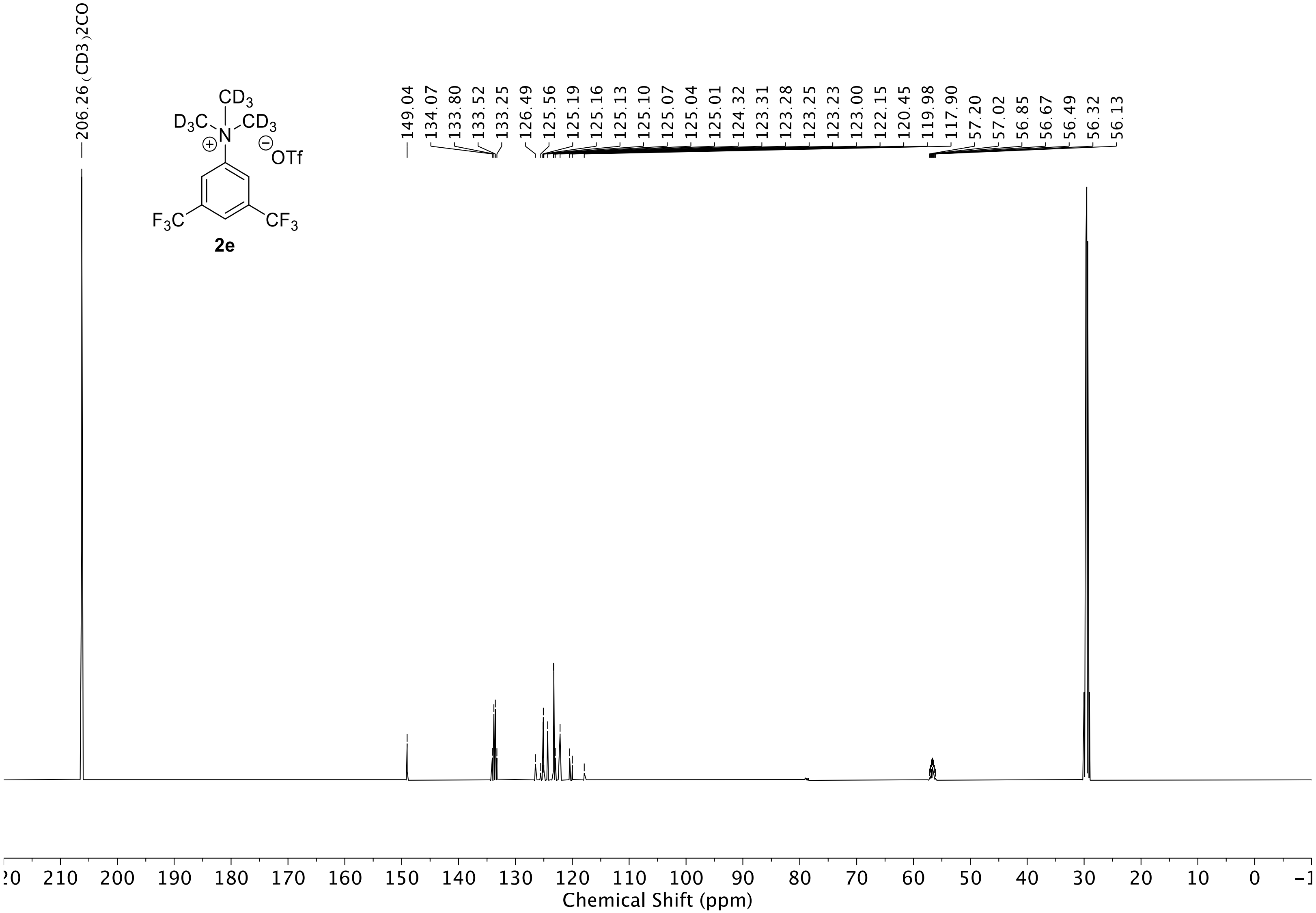

Figure S22. ^{19}F NMR (376 MHz, $(CD_3)_2CO$) of **2e**.

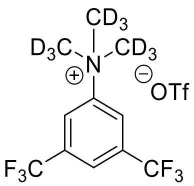

2e

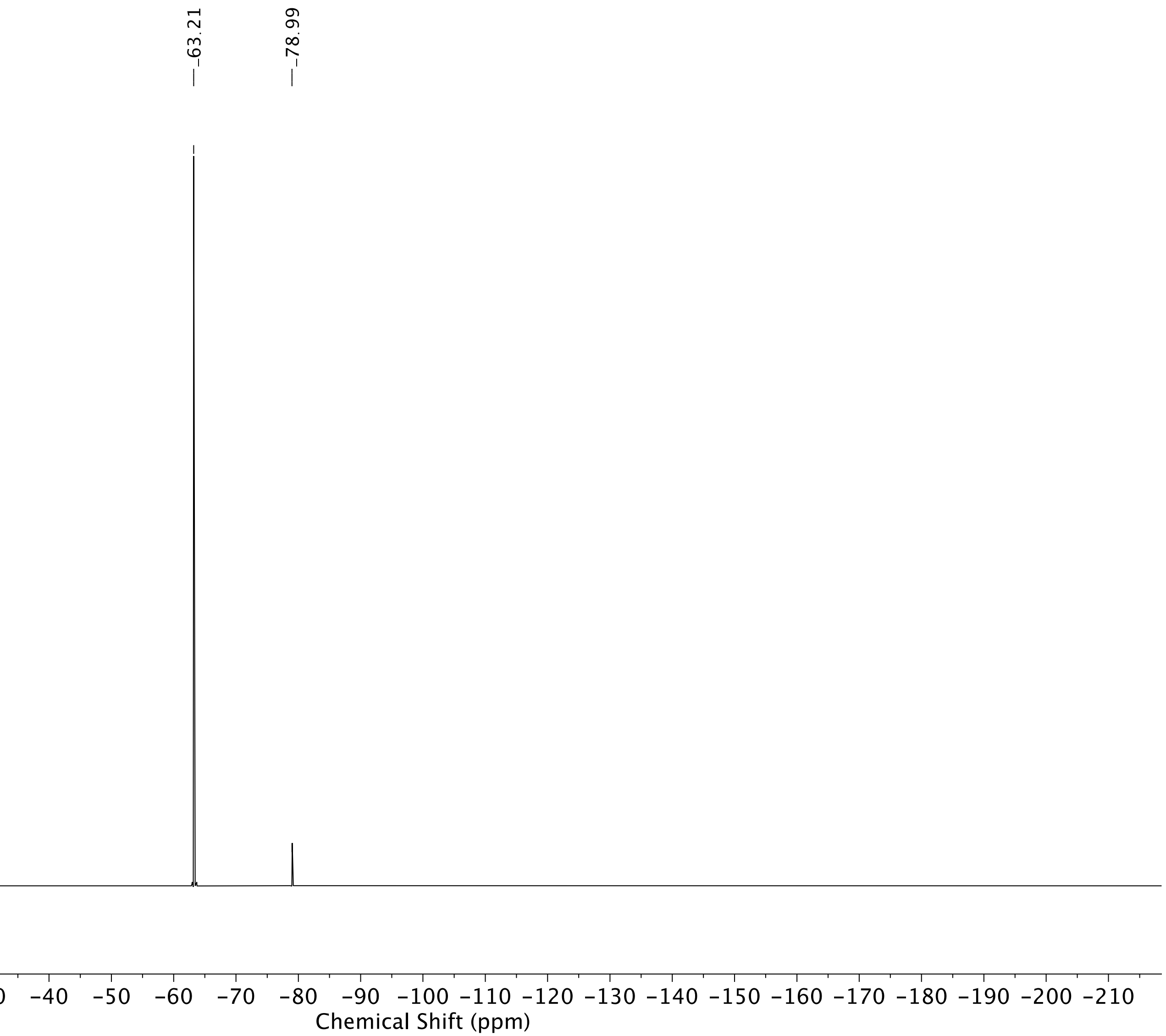

Figure S23. ^2H NMR (77 MHz, $(CD_3)_2CO$) of **2e**.

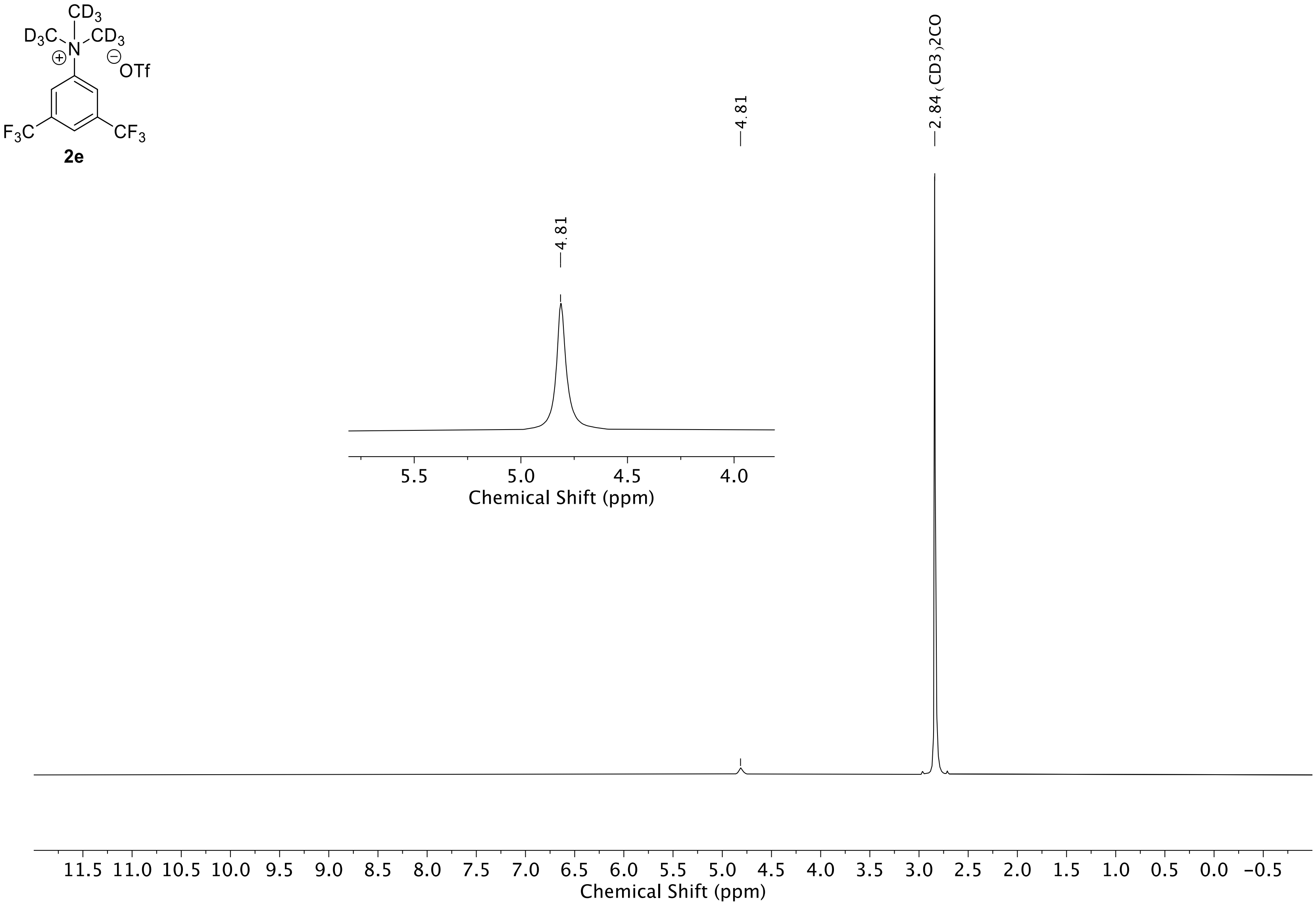

Figure S24. ^{1}H NMR (400 MHz, $CDCl_3$) of **1j**.

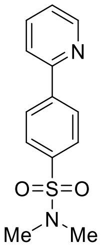

1j

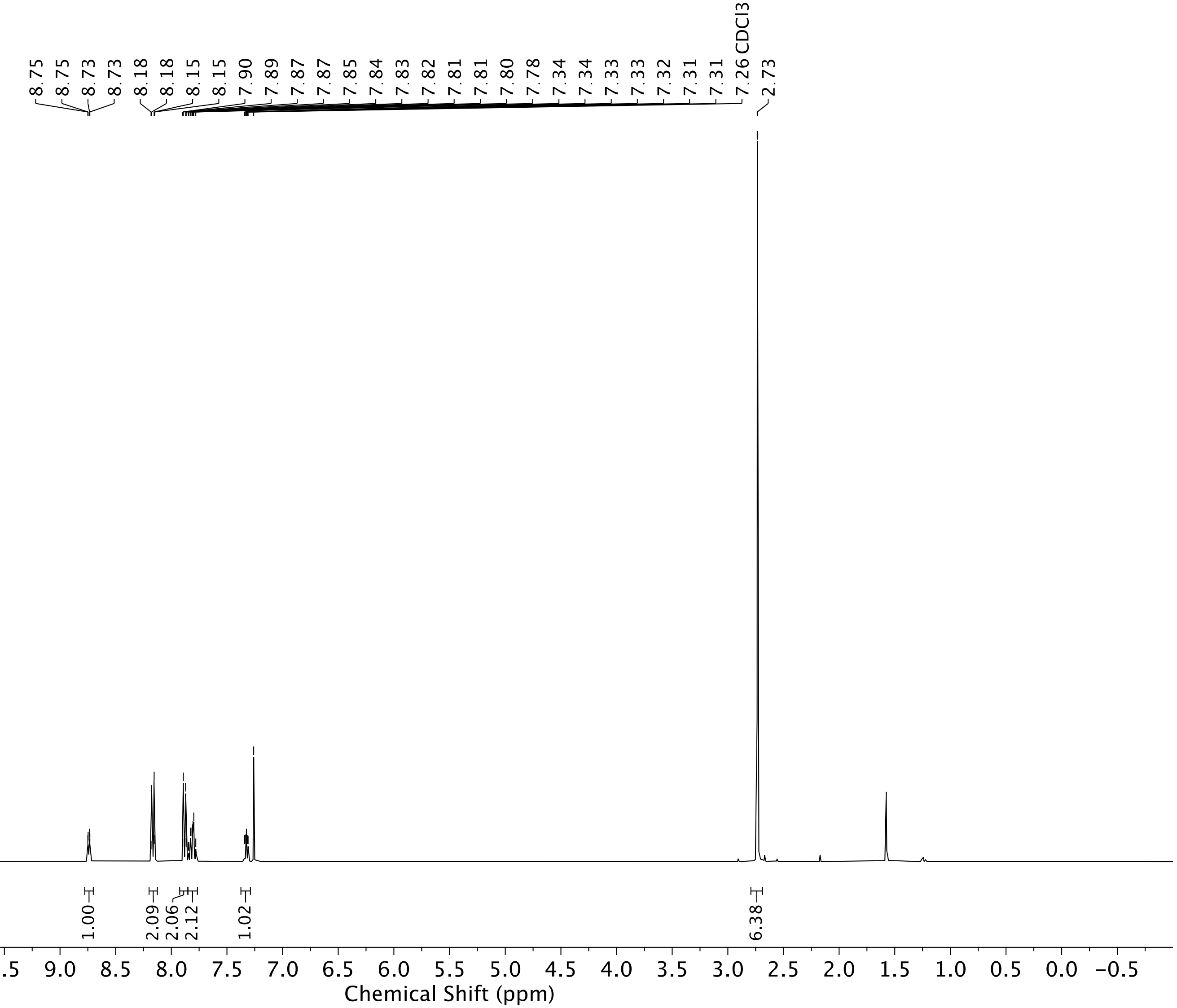

Figure S25. ^{13}C NMR (126 MHz, $CDCl_3$) of **1j**.

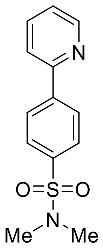

1j

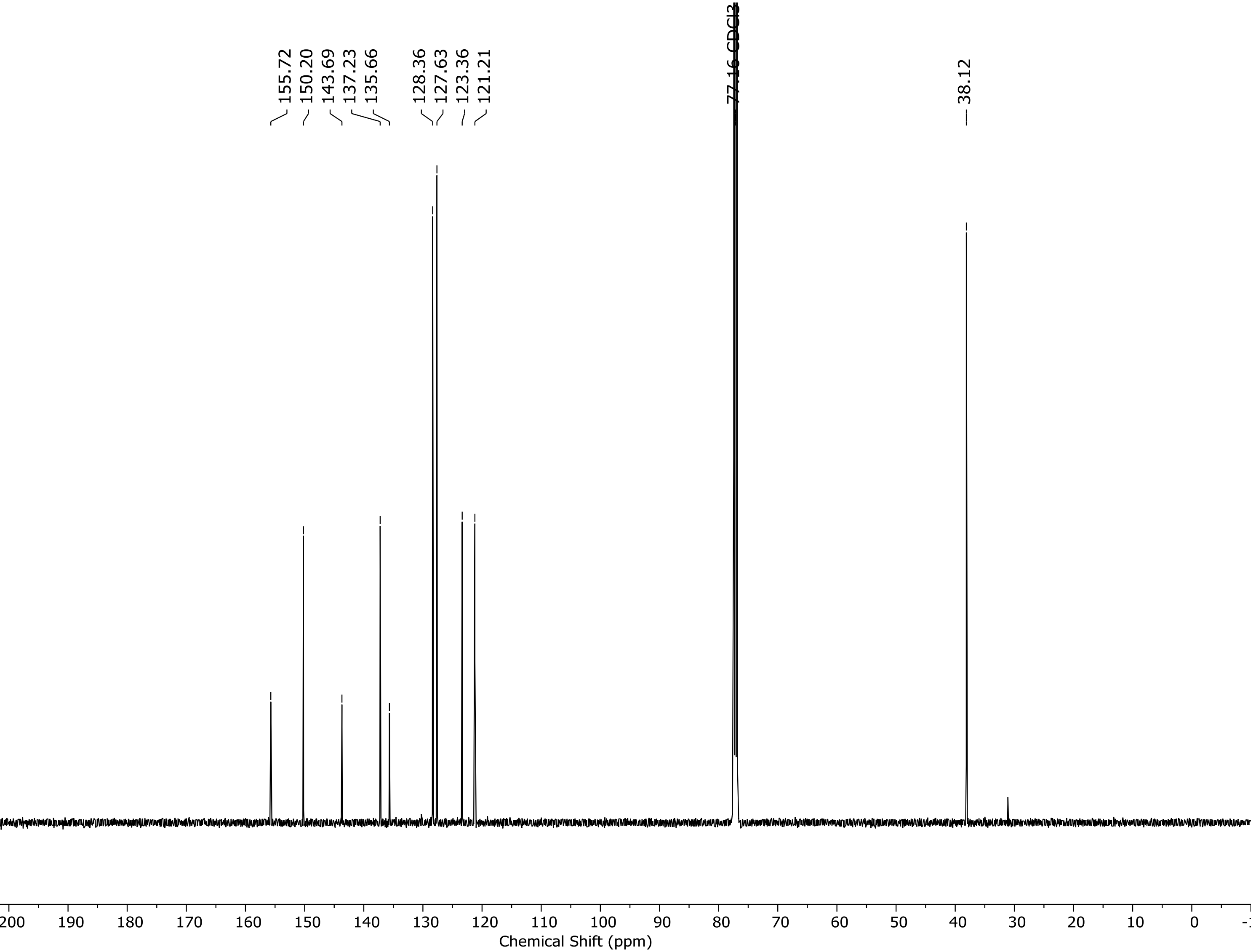

Figure S26. ^{1}H NMR (400 MHz, $CDCl_3$) of **1q.**

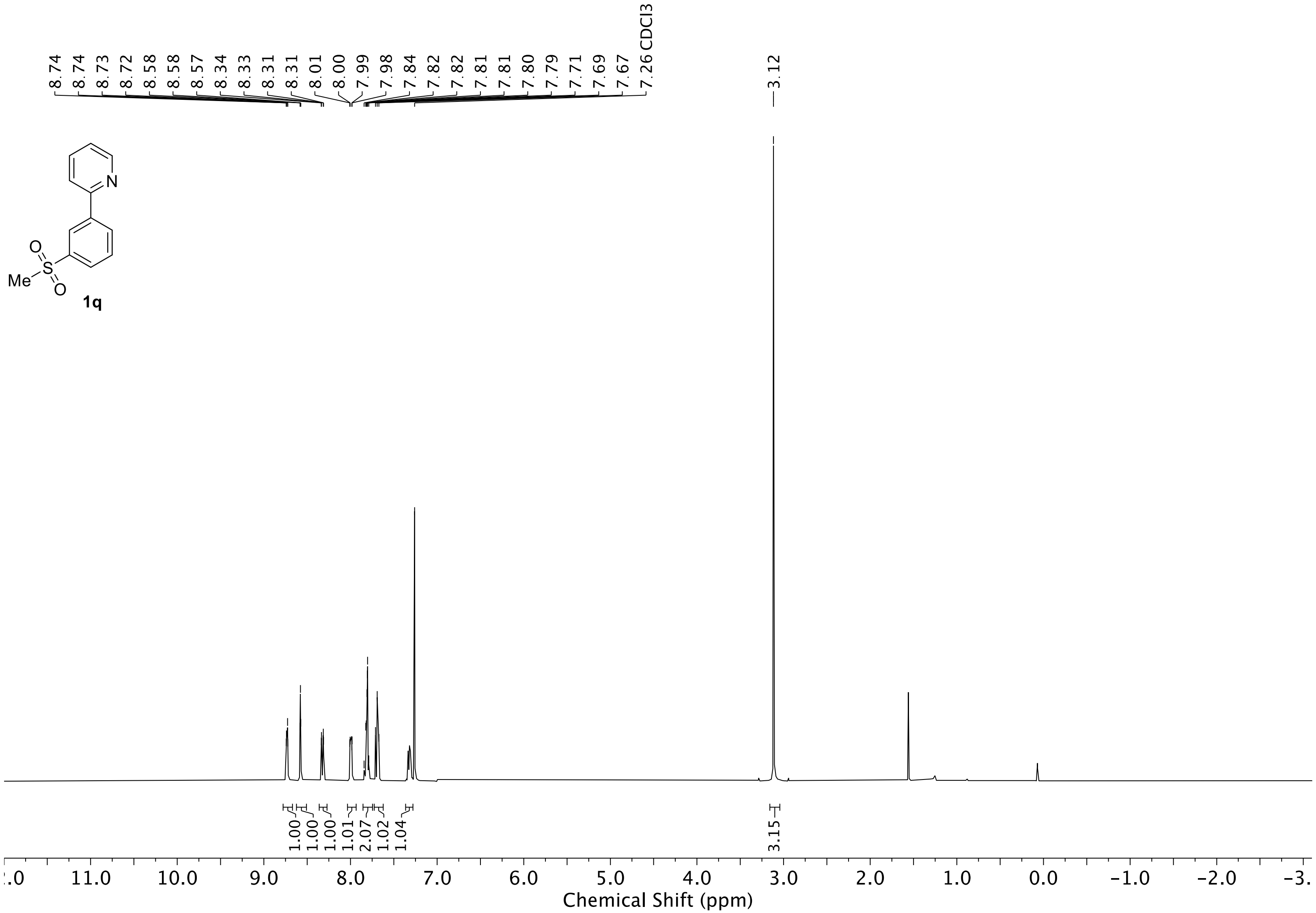

Figure S27. ^{13}C NMR (101 MHz, $CDCl_3$) of **1q.**

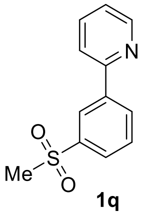

1q

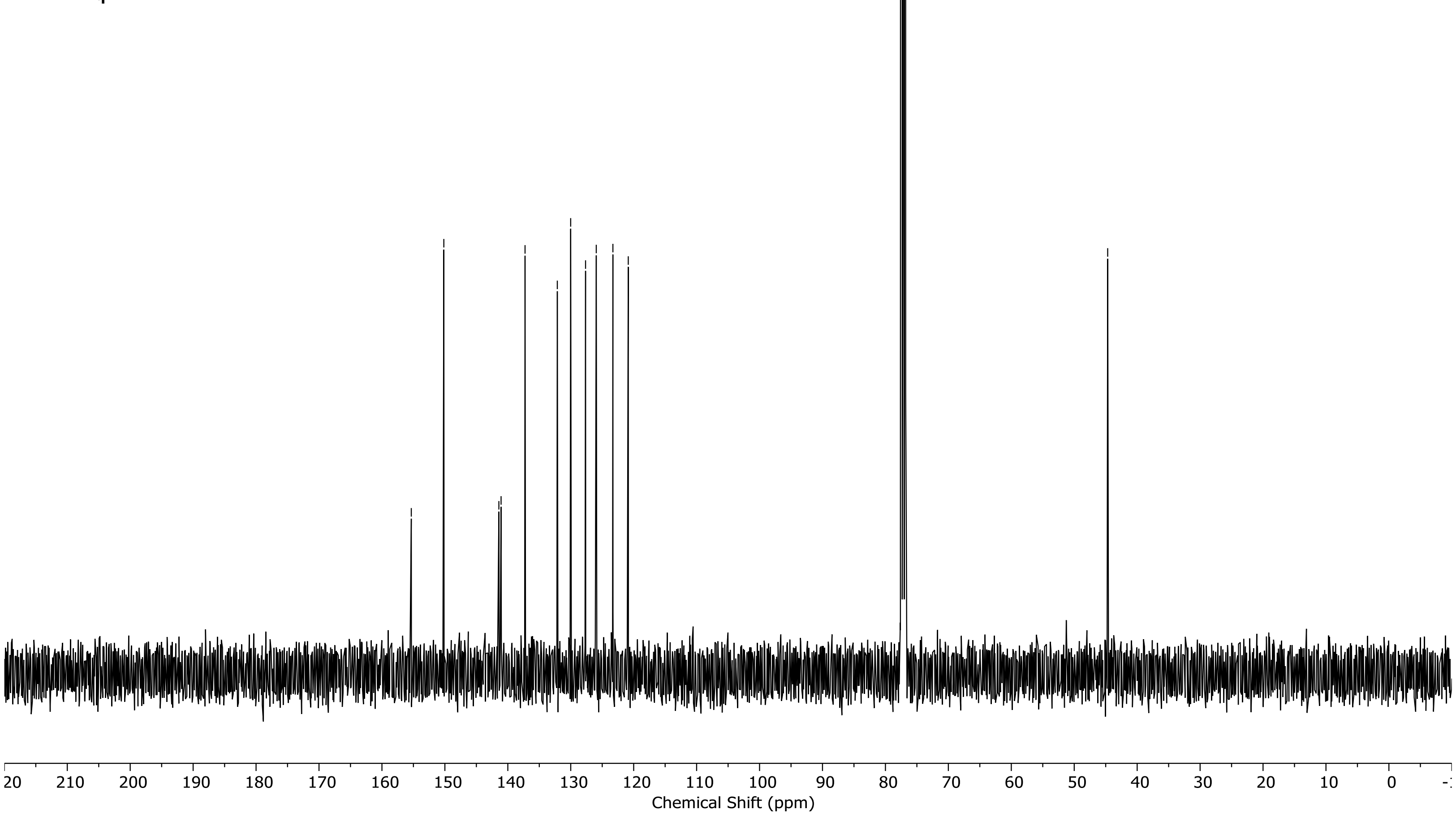

Figure S28. ^{1}H NMR (400 MHz, $CDCl_3$) of **5a.**

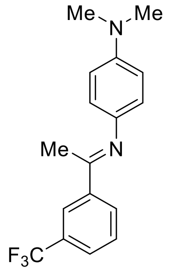

5a

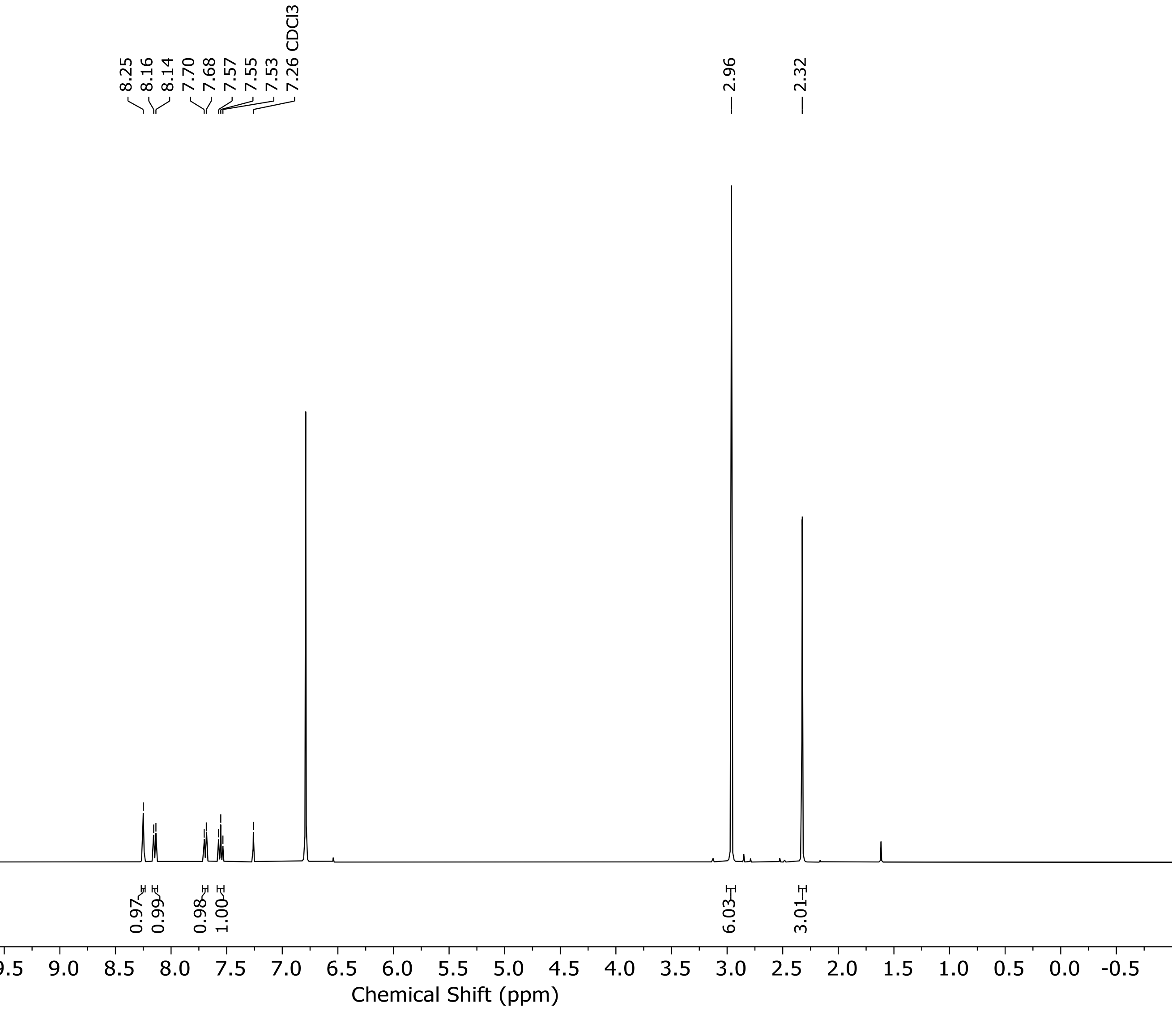

Figure S29. ^{13}C NMR (101 MHz, $CDCl_3$) of **5a**

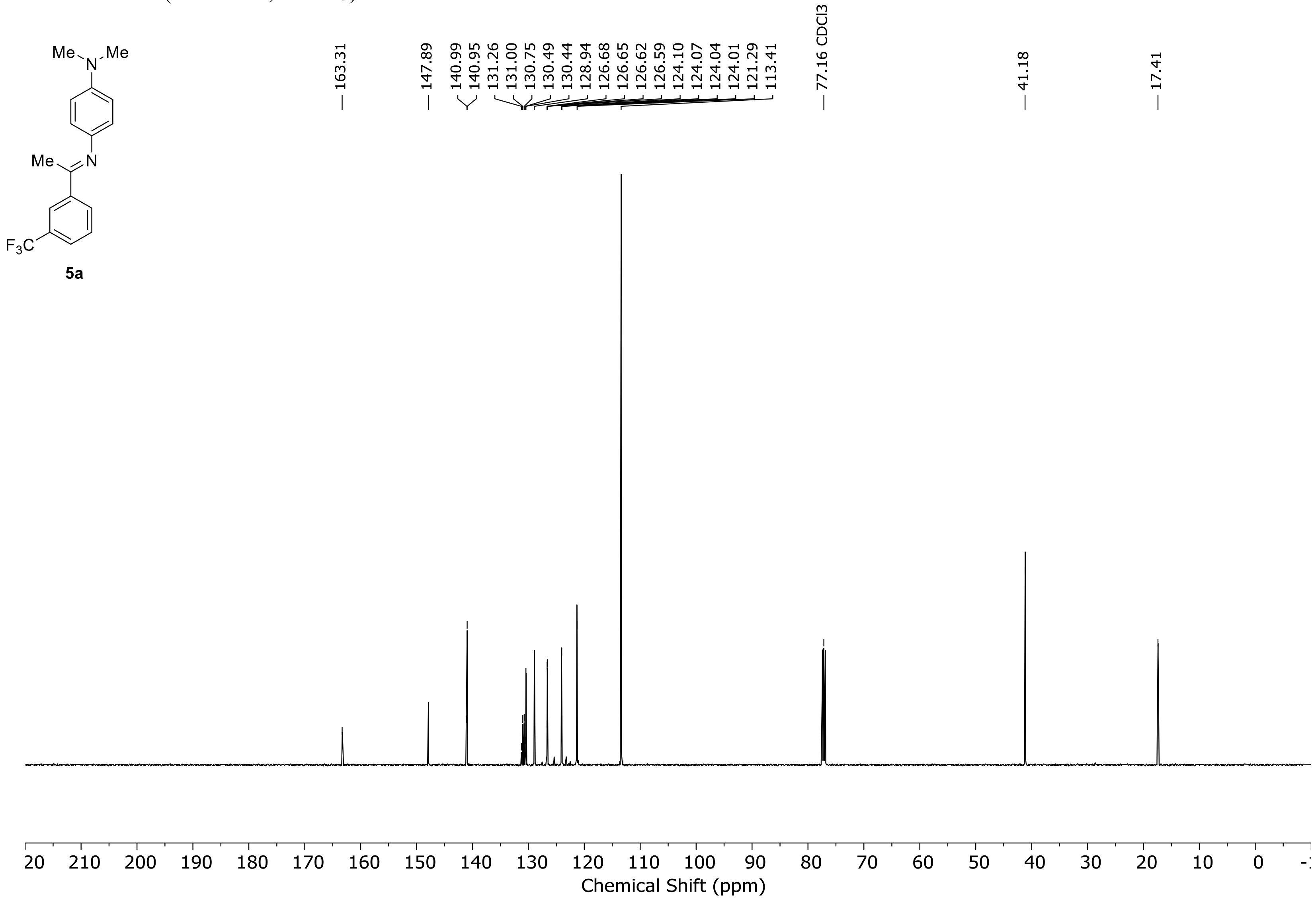

Figure S30. ^{19}F NMR (471 MHz, $CDCl_3$) of 5**a**.

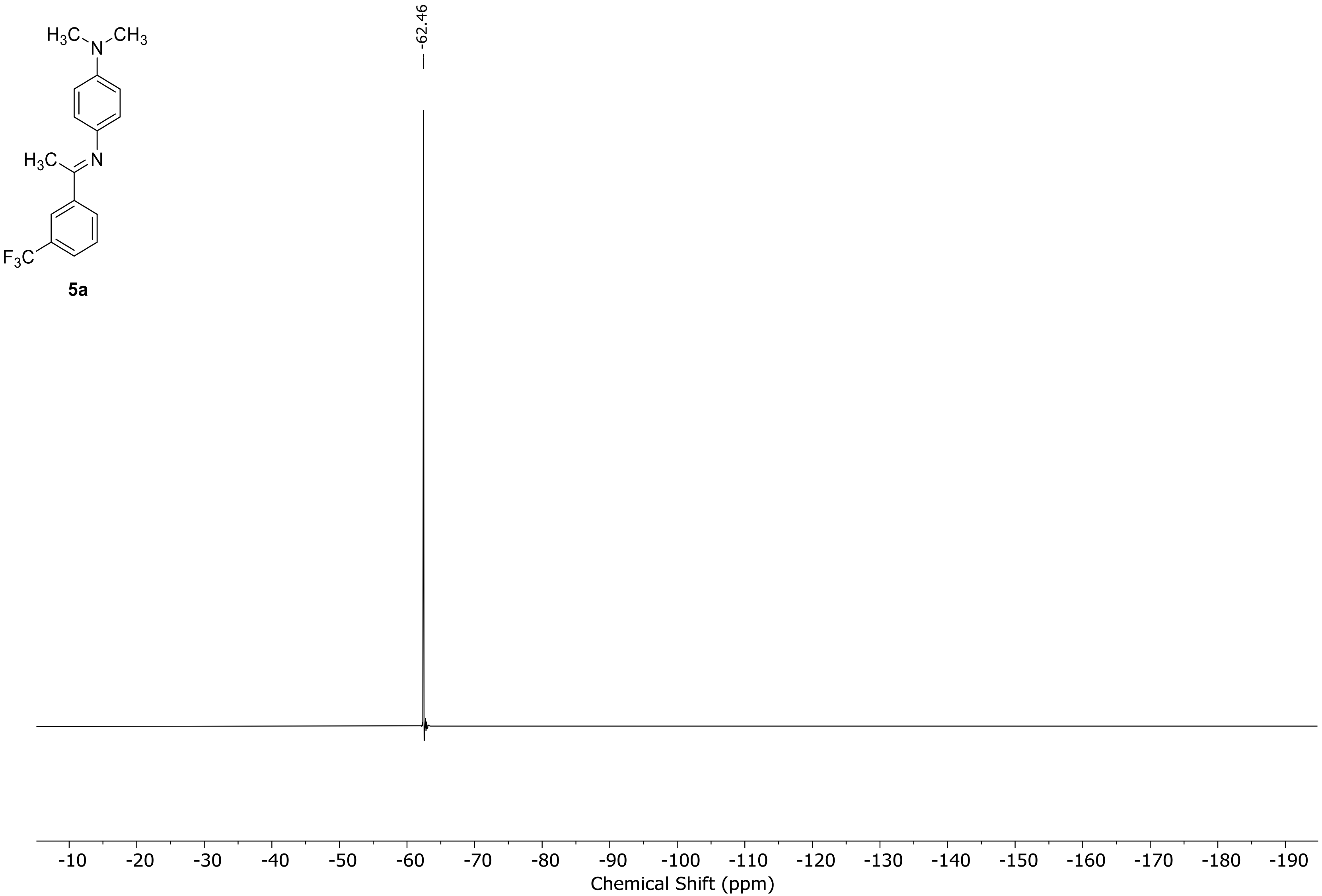

Figure S31. ^{1}H NMR (400 MHz, $CDCl_3$) of **5b**.

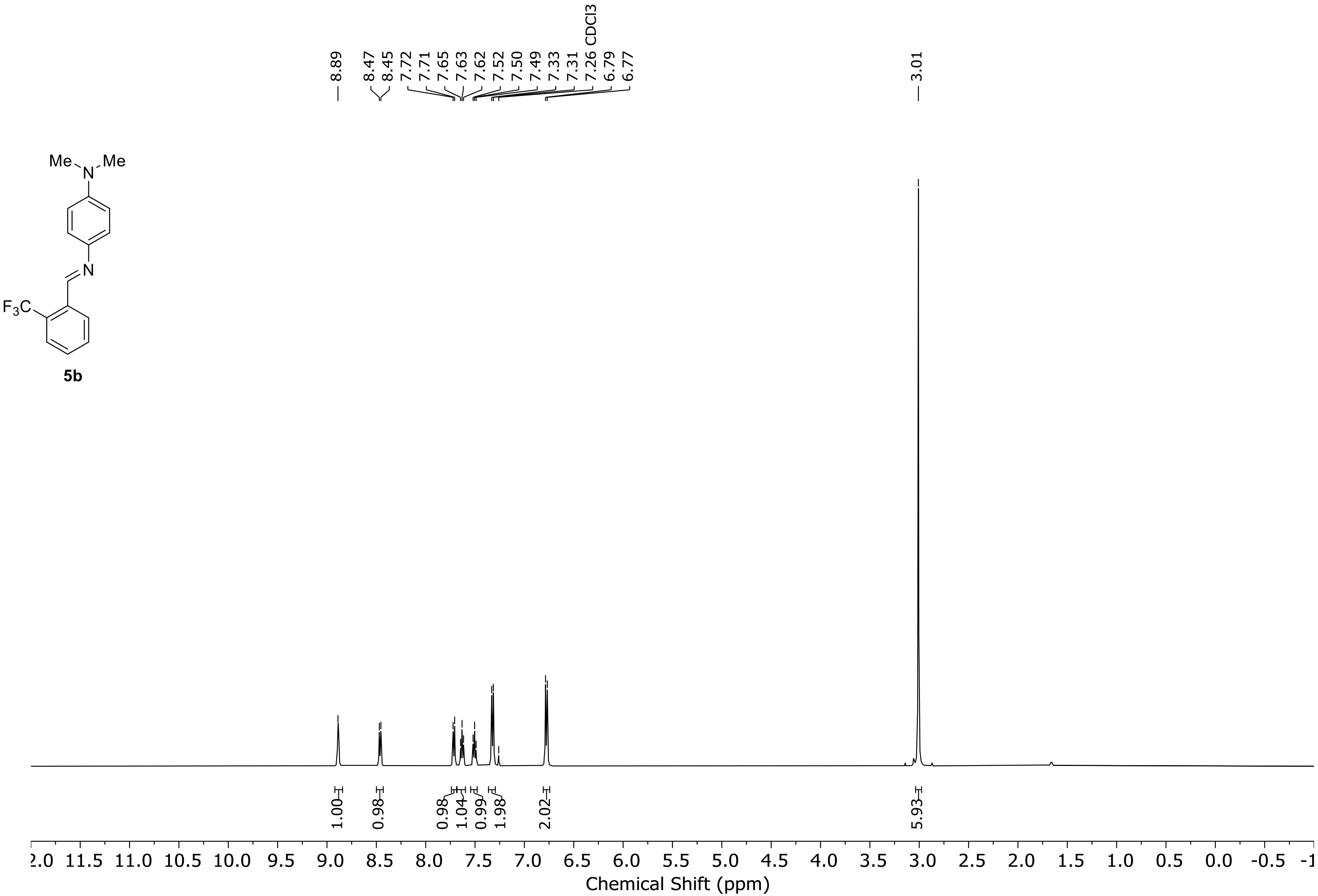

Figure S32. ^{13}C NMR (101 MHz, $CDCl_3$) of **5b**

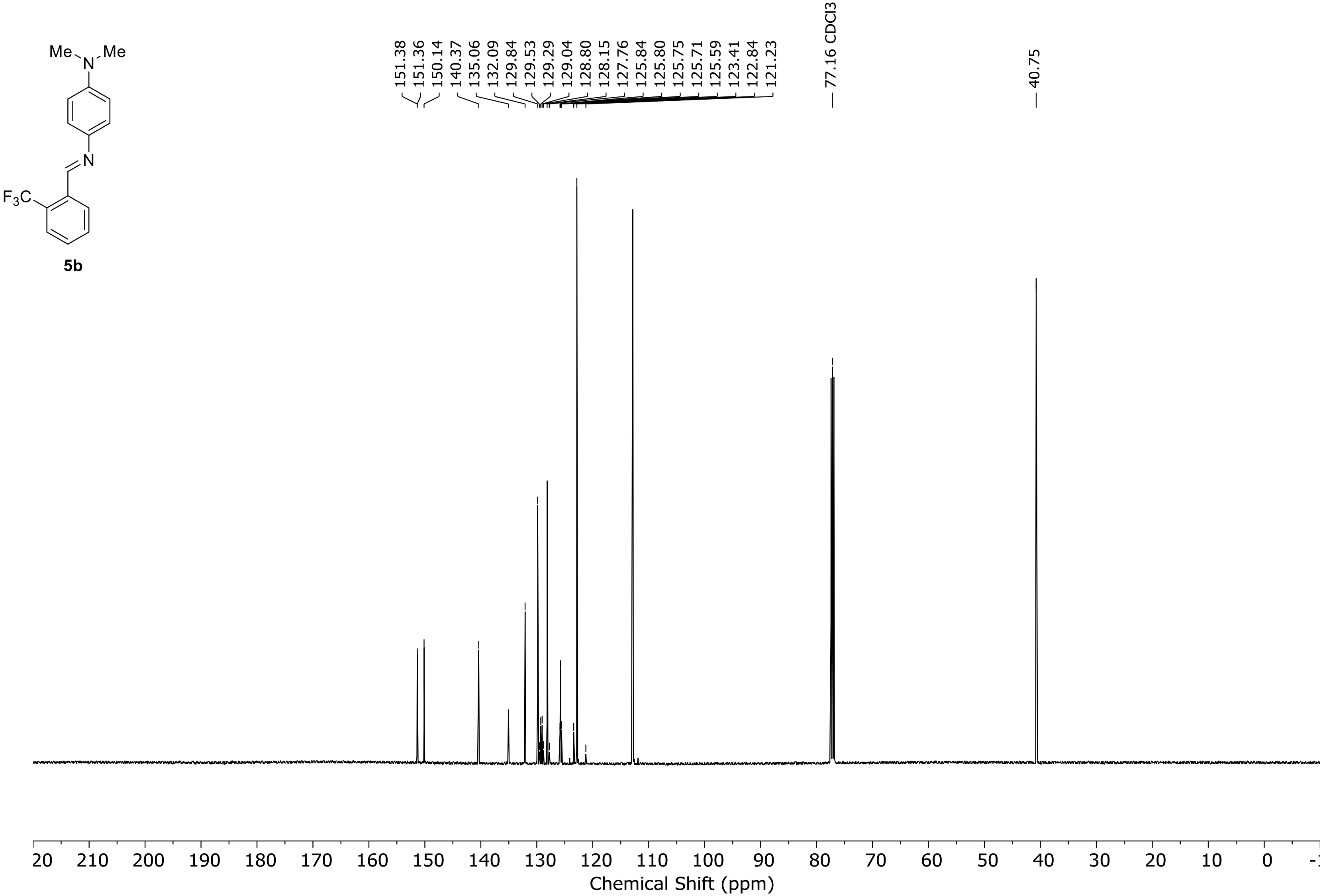

Figure S33. ^{19}F NMR (471 MHz, $CDCl_3$) of **5b**

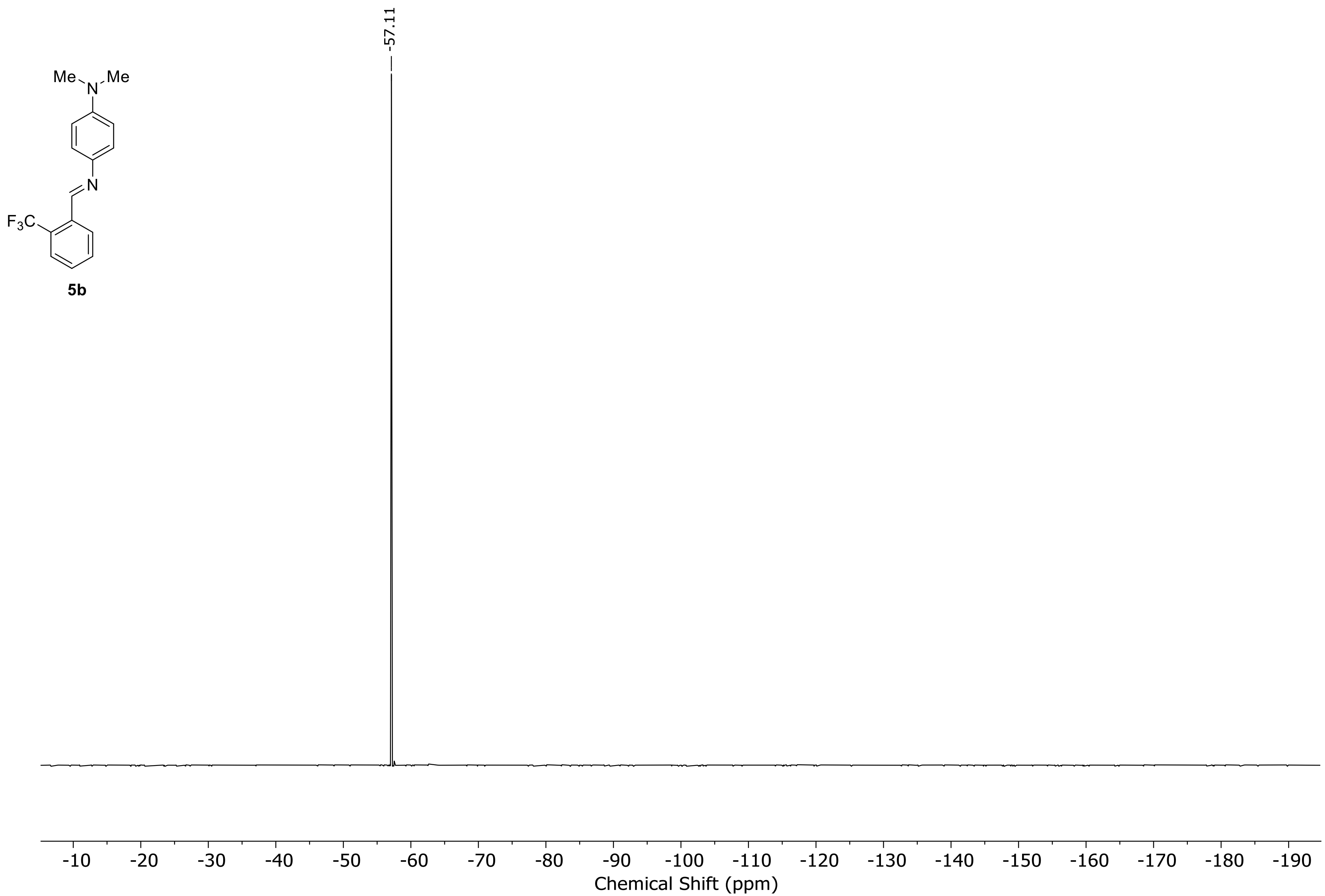

Figure S34. ^{1}H NMR (400 MHz, $CDCl_3$) of **5c.**

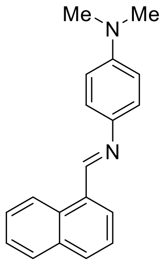

5c

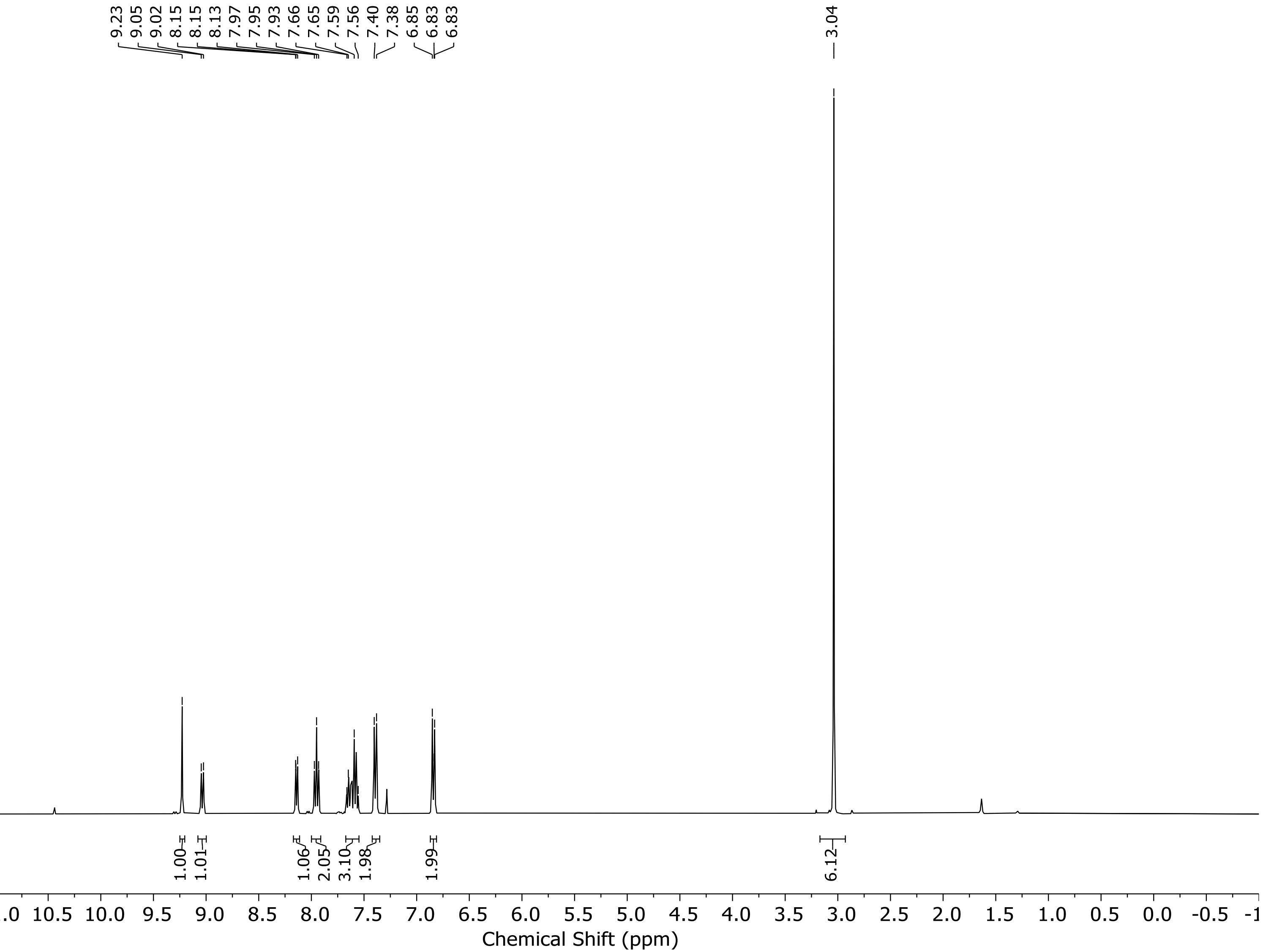

Figure S35. ^{13}C NMR (101 MHz, $CDCl_3$) of **5c**

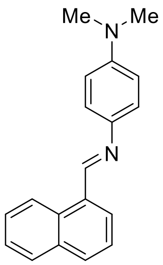

5c

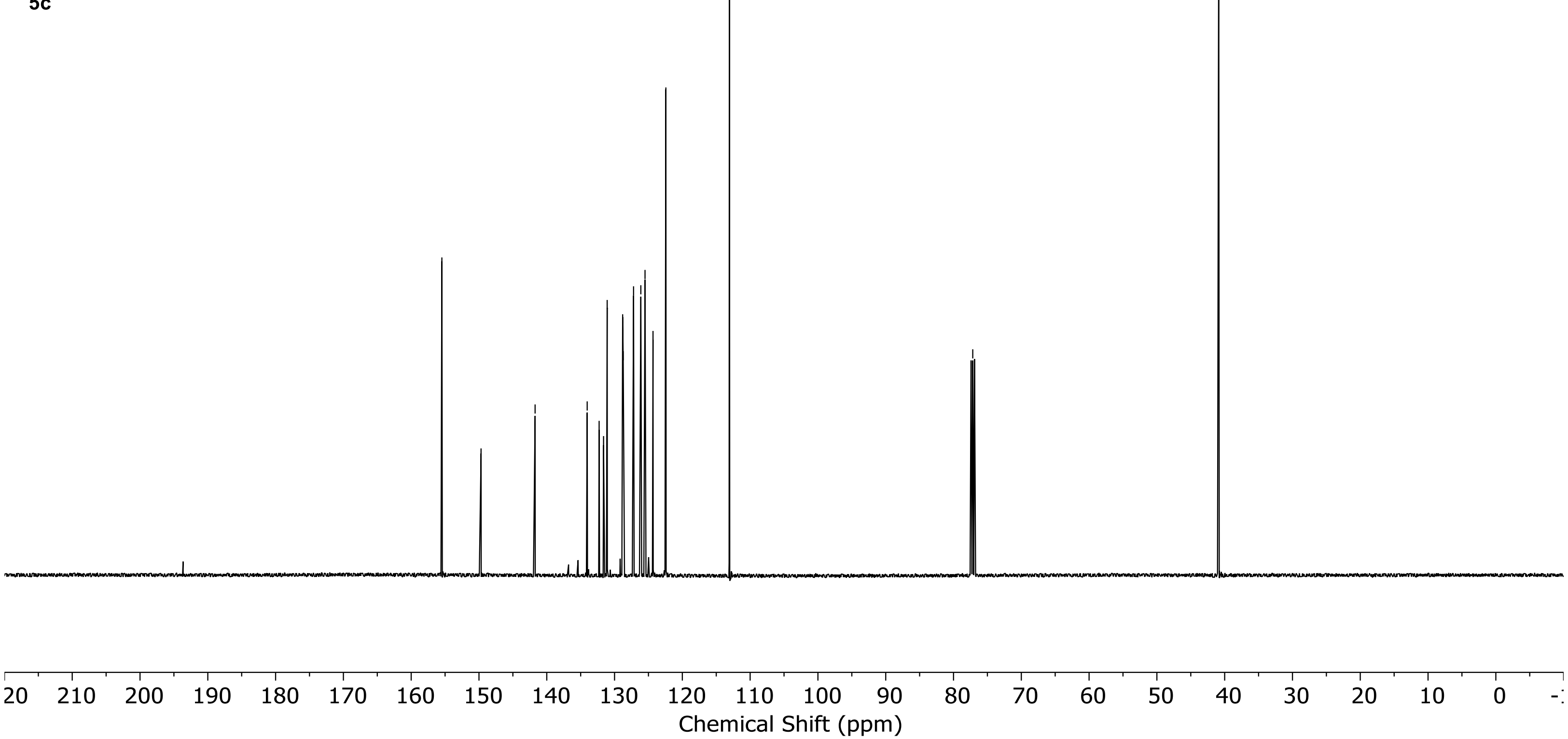

Figure S36. ^{1}H NMR (400 MHz, $CDCl_3$) of **7c**

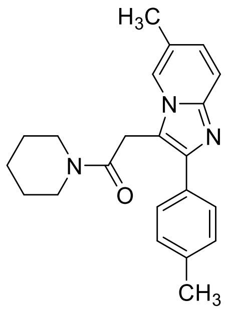

7c

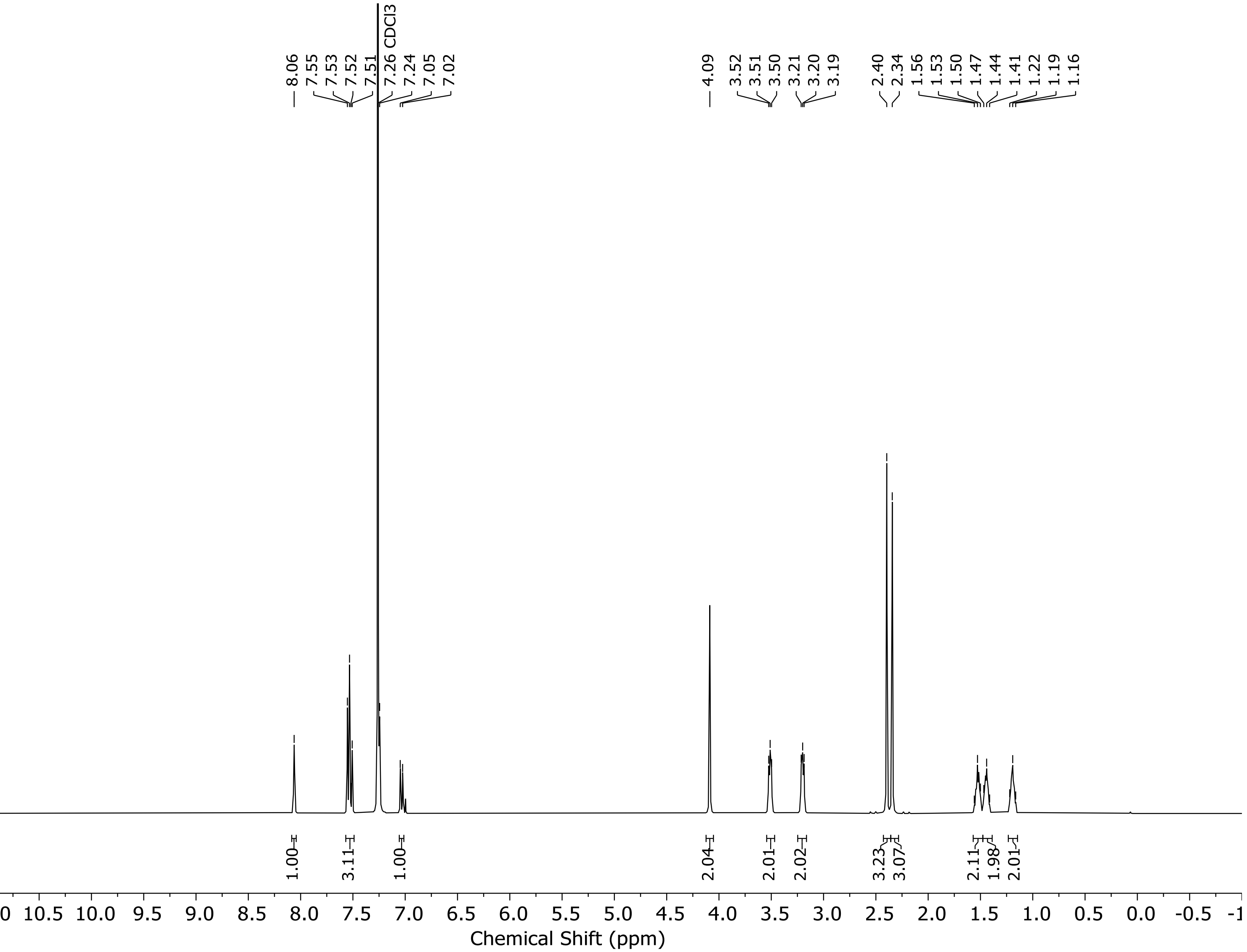

Figure S37. ^{13}C NMR (101 MHz, $CDCl_3$) of **7c**

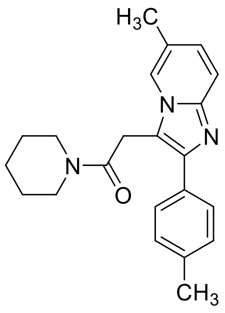

7c

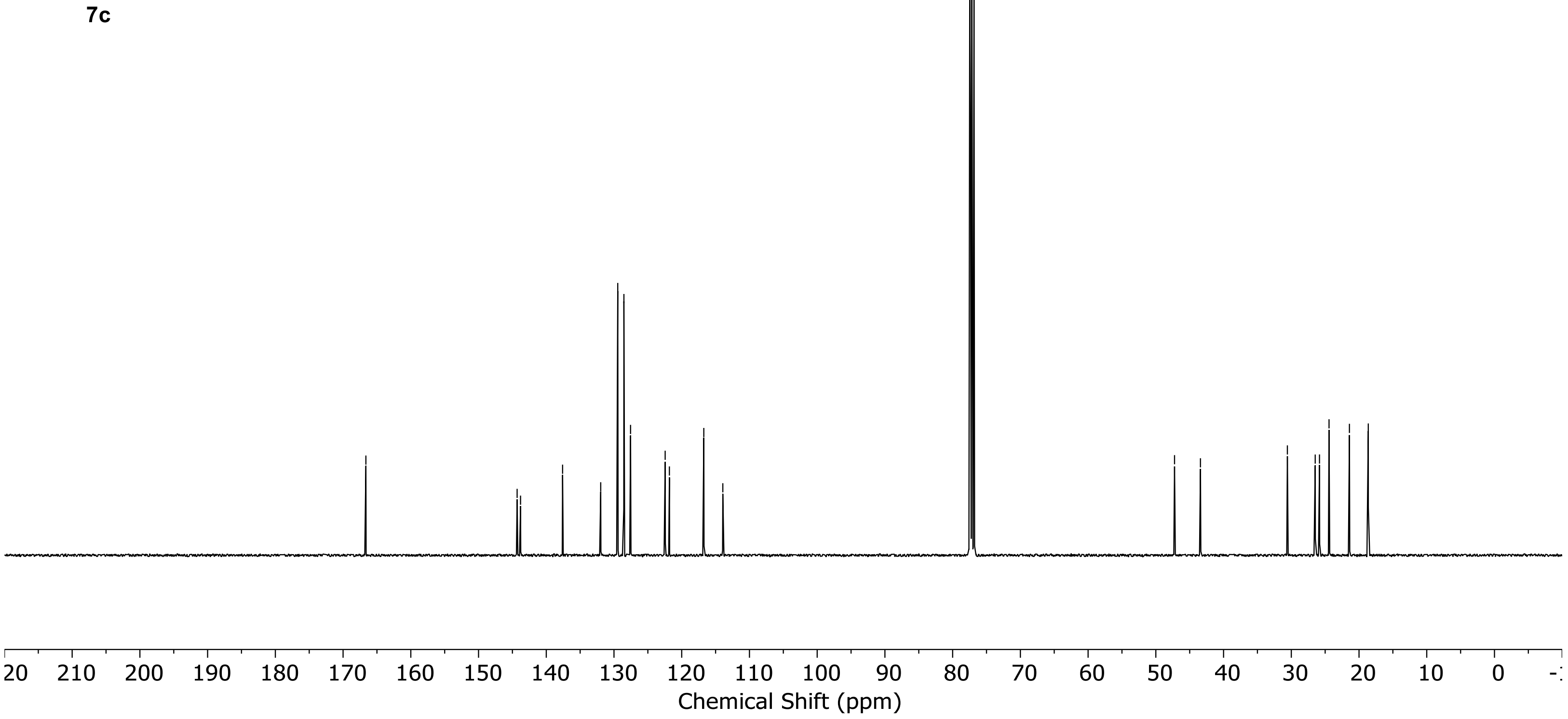

Figure S38. ^{1}H NMR (400 MHz, $CDCl_3$) of **7e**.

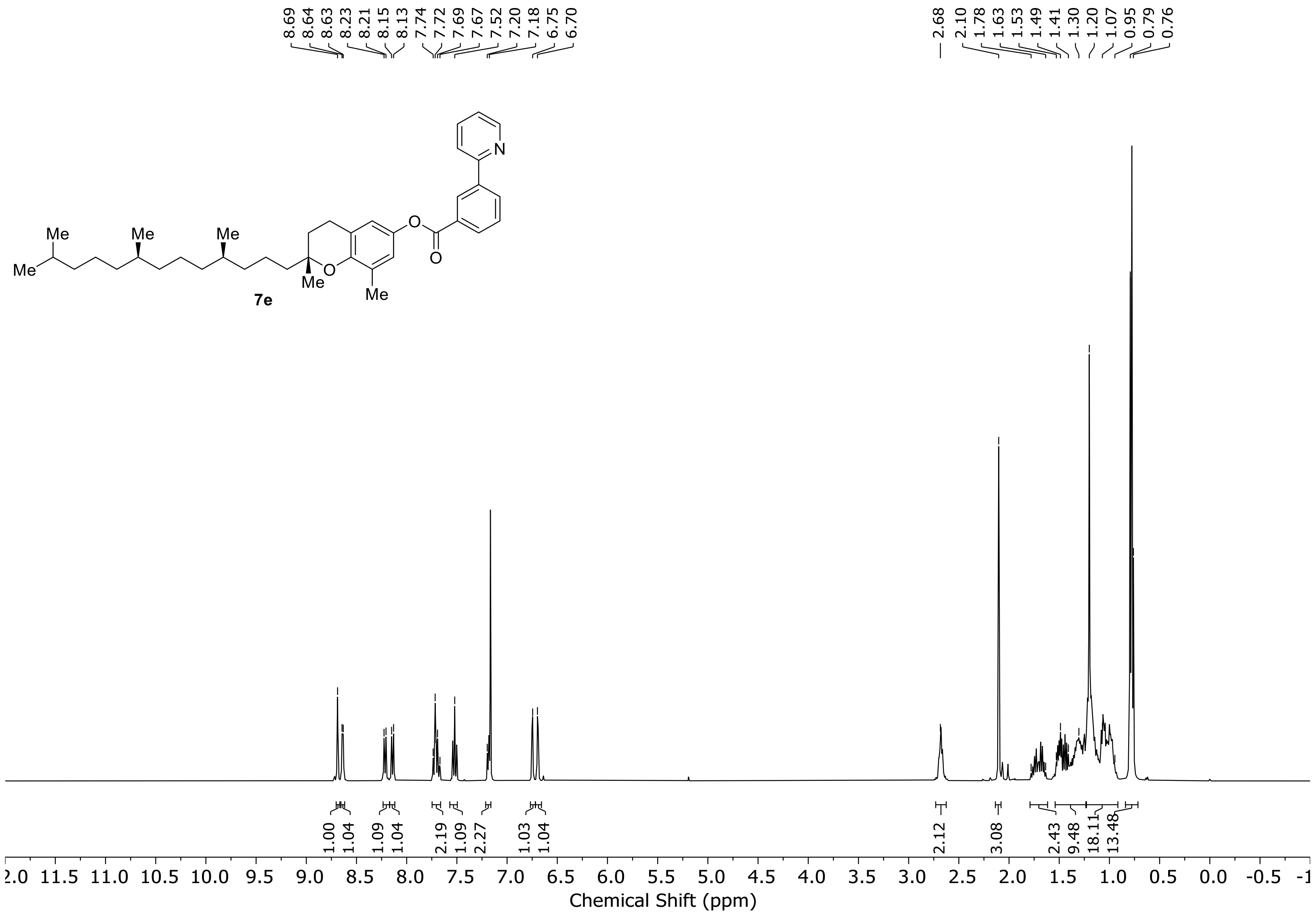

Figure S39. ^{13}C NMR (101 MHz, $CDCl_3$) of **7e**

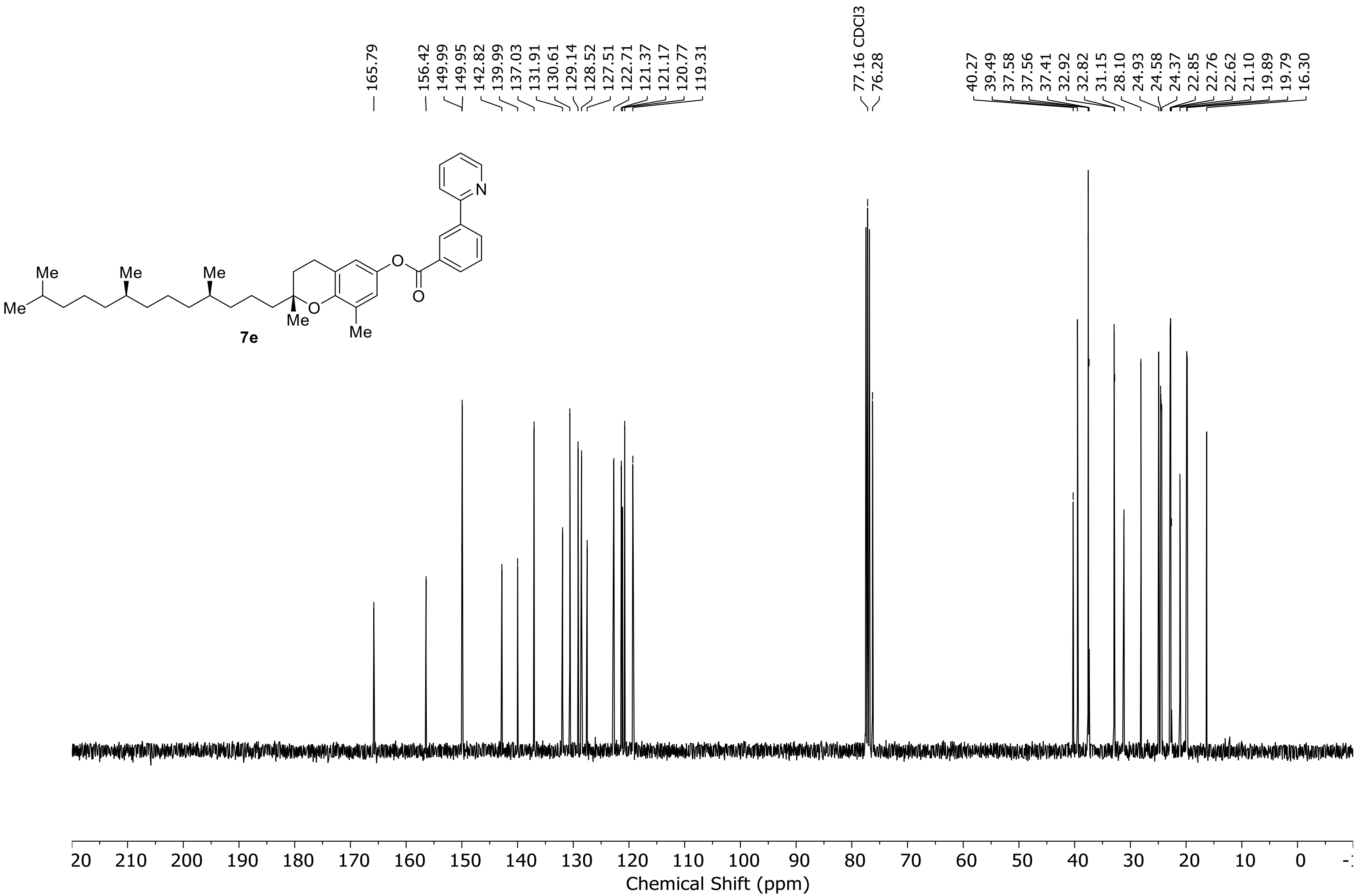

Figure S40. ^{1}H NMR (400 MHz, $CDCl_3$) of **7f**.

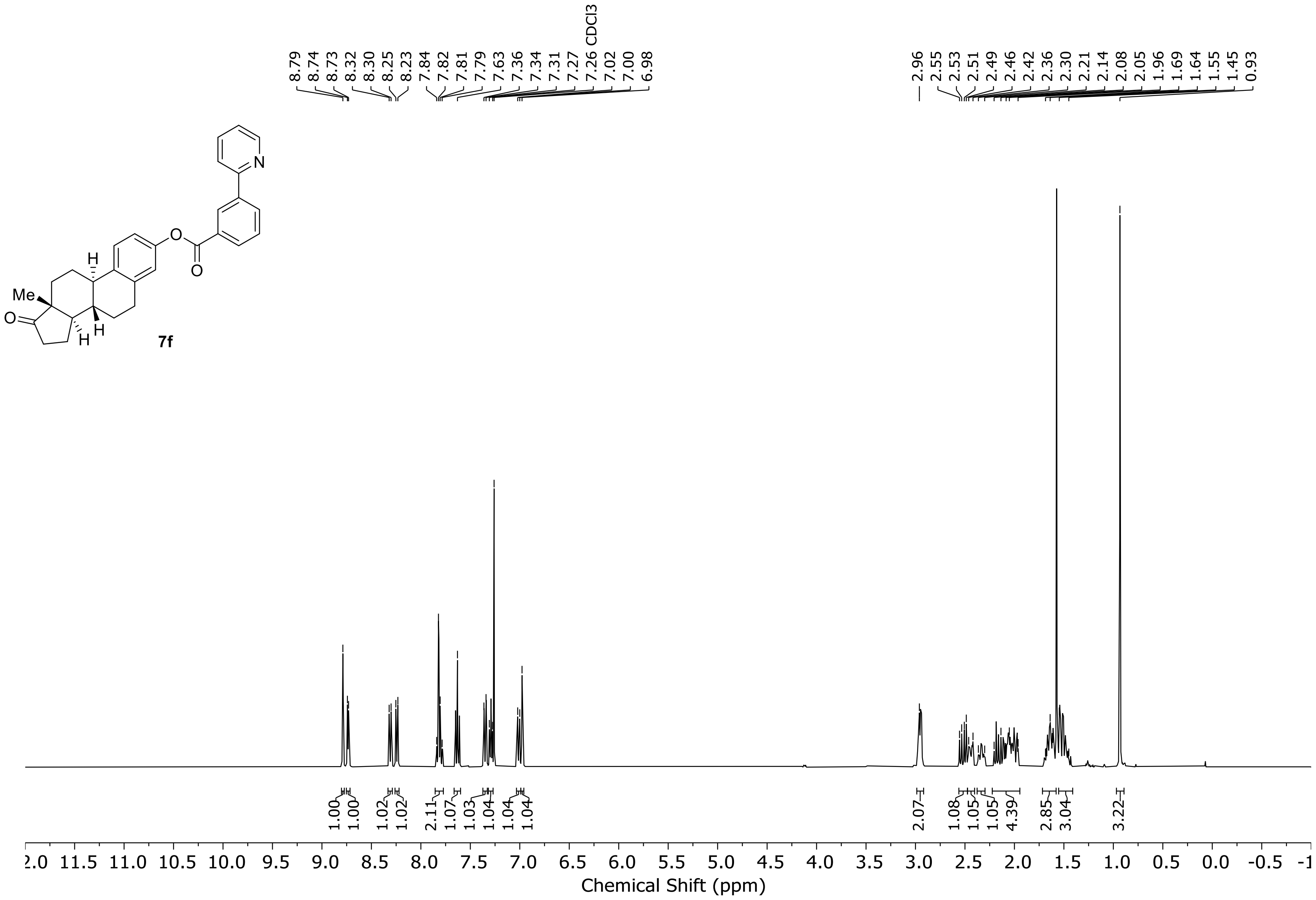

Figure S41. ^{13}C NMR (101 MHz, $CDCl_3$) of **7f**

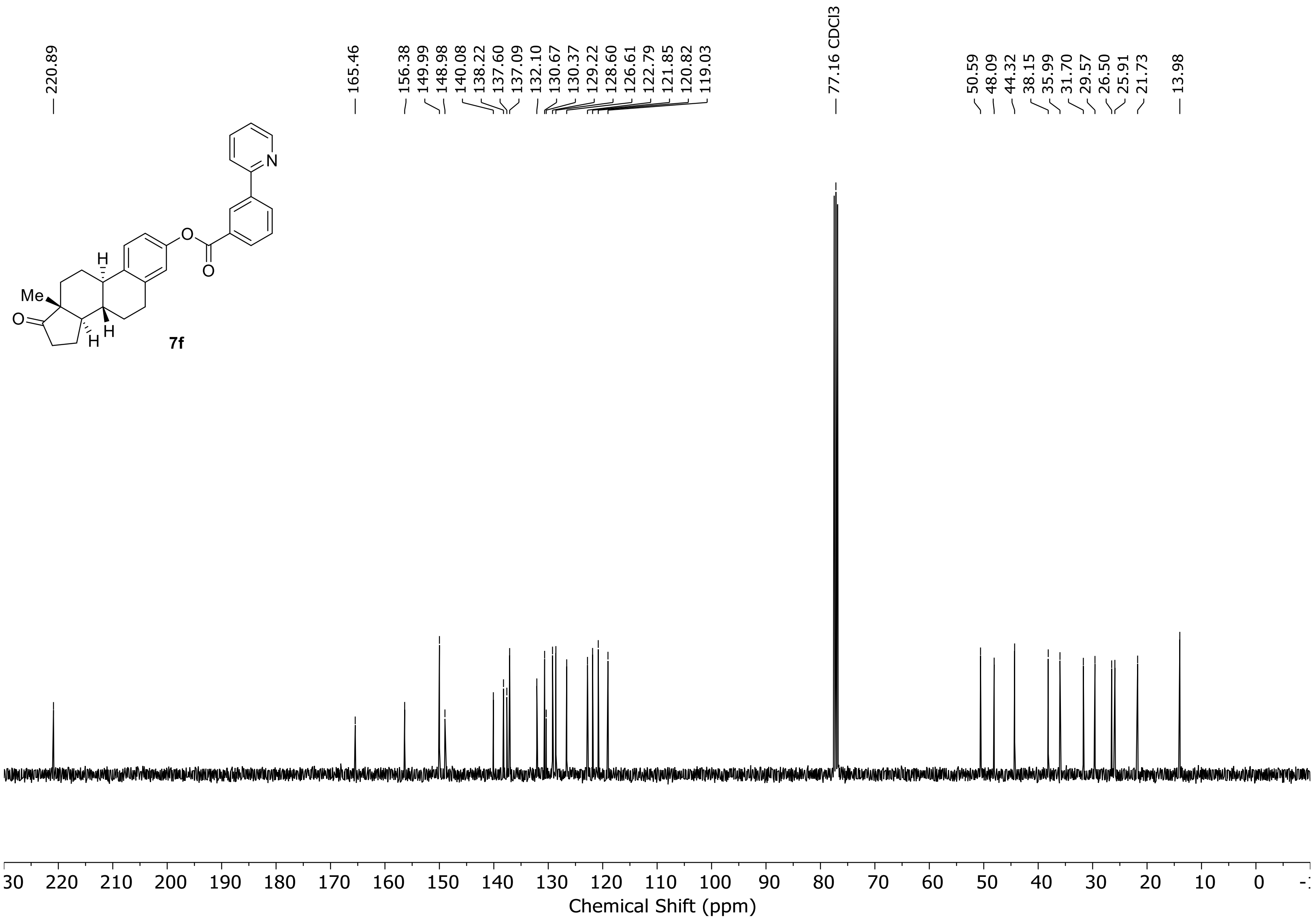

Figure S42. ^{1}H NMR (400 MHz, $CDCl_3$) of **7g**.

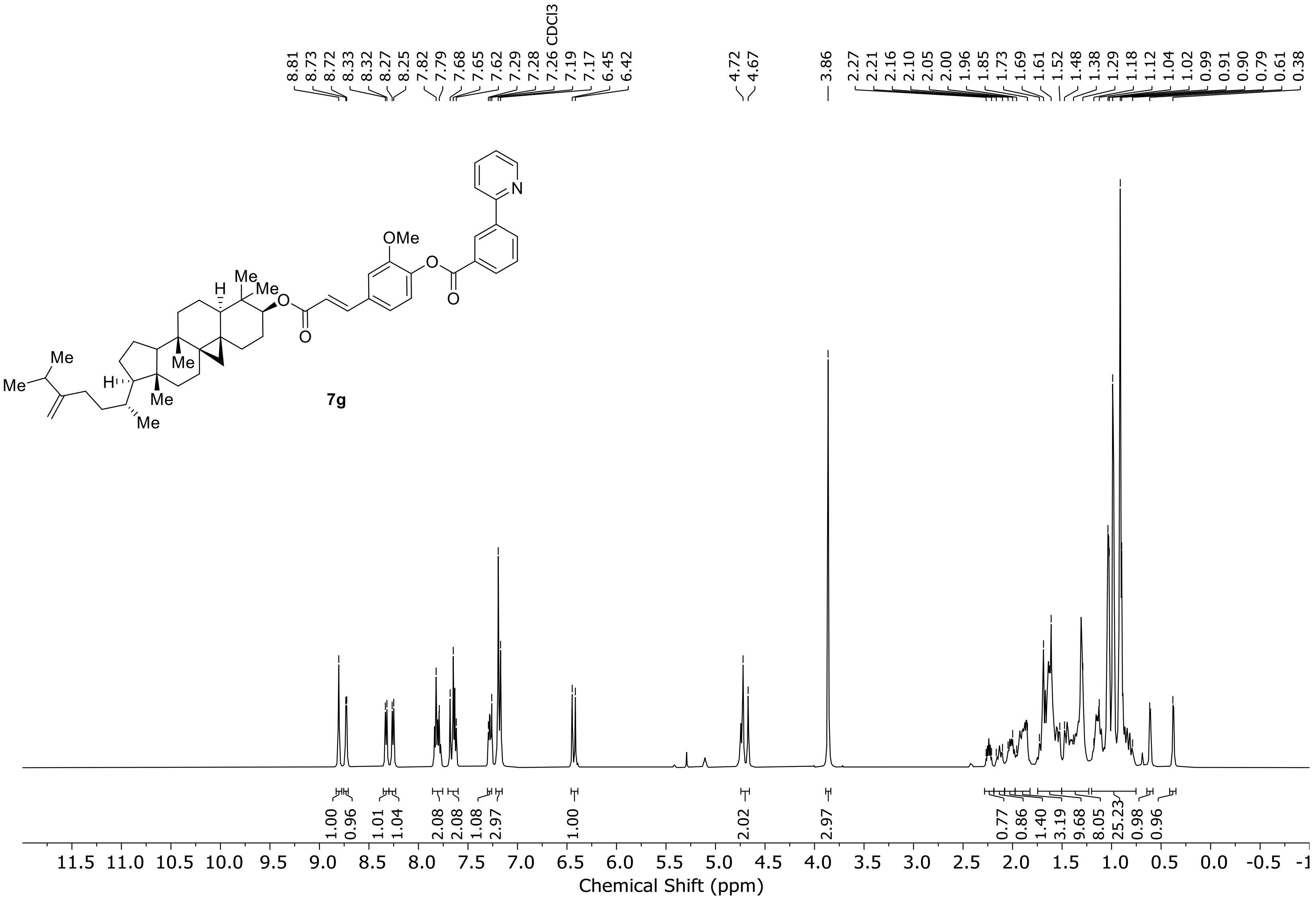

Figure S43. ^{13}C NMR (61 MHz, $CDCl_3$) of **7g**.

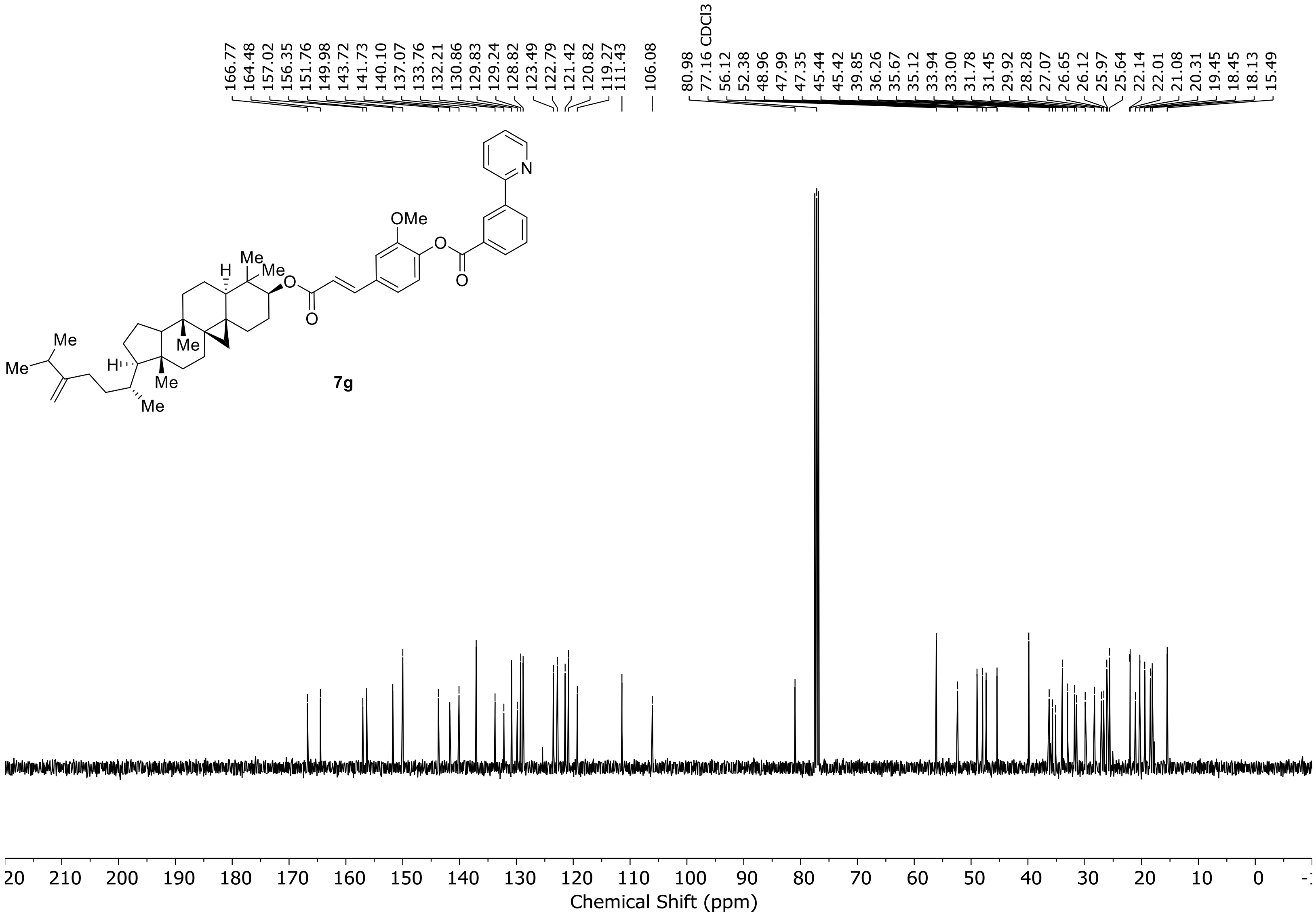

Figure S44. ^{1}H NMR (500 MHz, $CDCl_3$) of **3aa**.

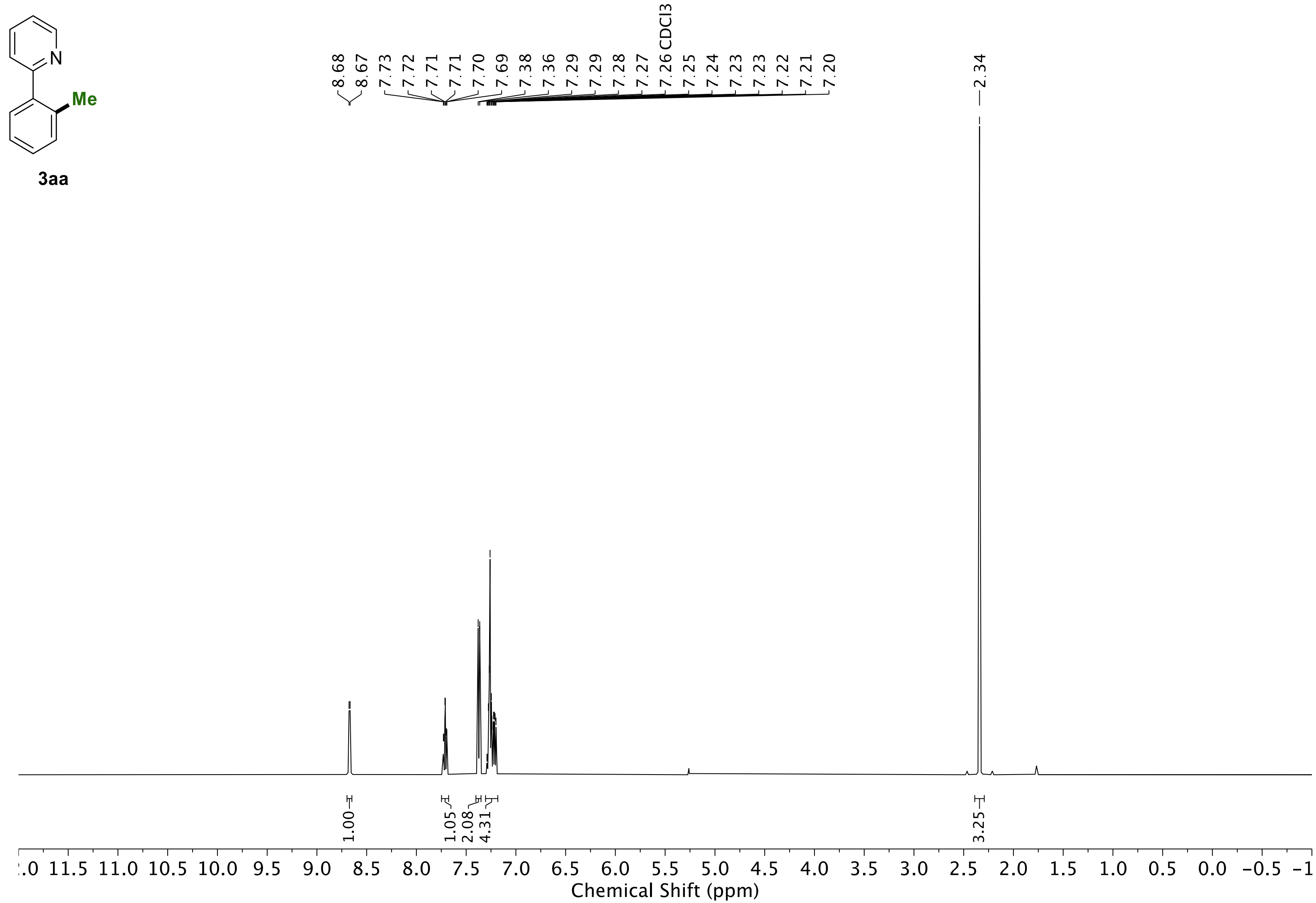

Figure S45. ^{13}C NMR (126 MHz, $CDCl_3$) of **3aa**.

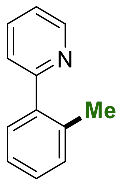
3aa

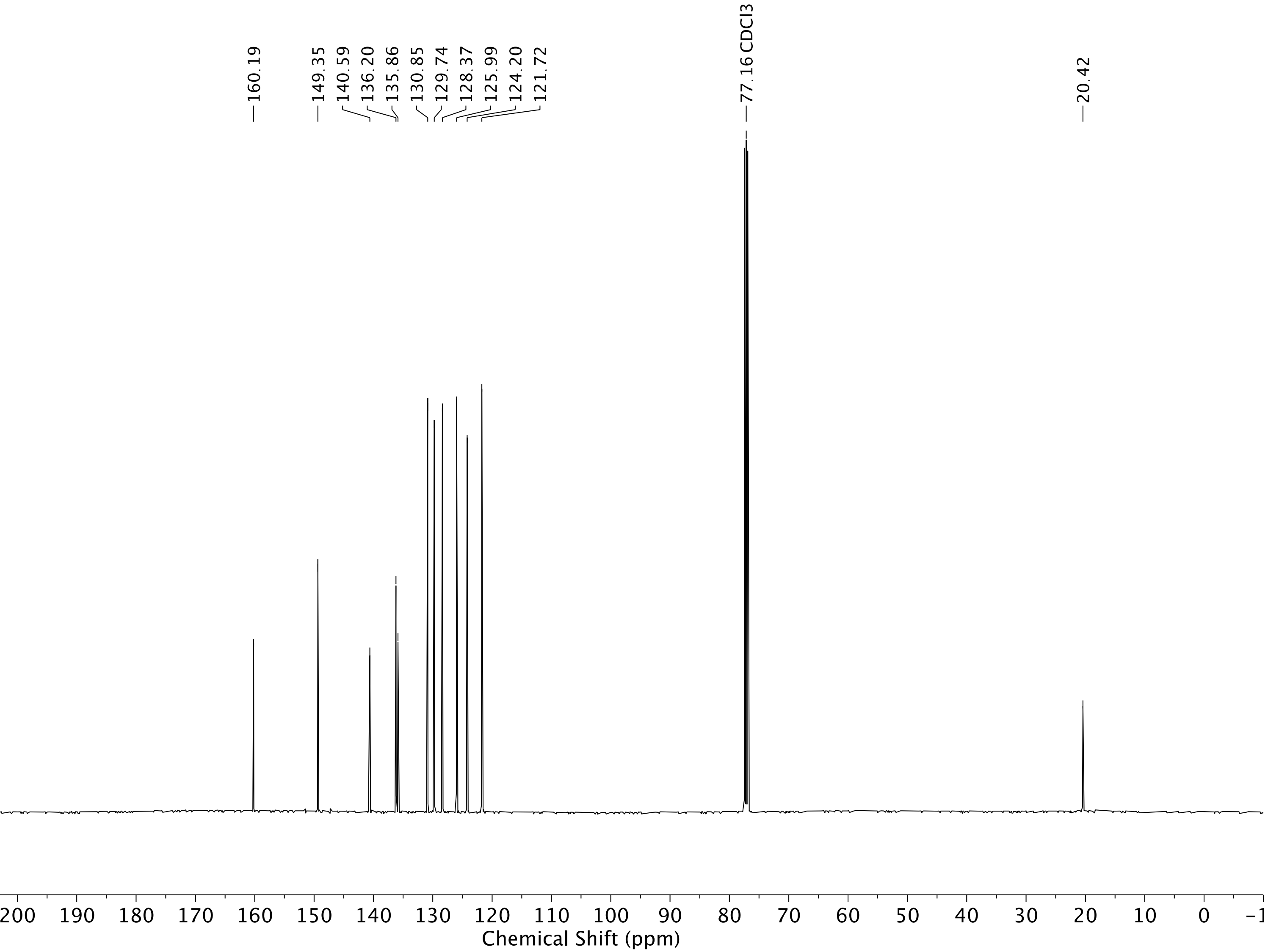

Figure S46. ^{1}H NMR (400 MHz, $CDCl_3$) of **4aa**.

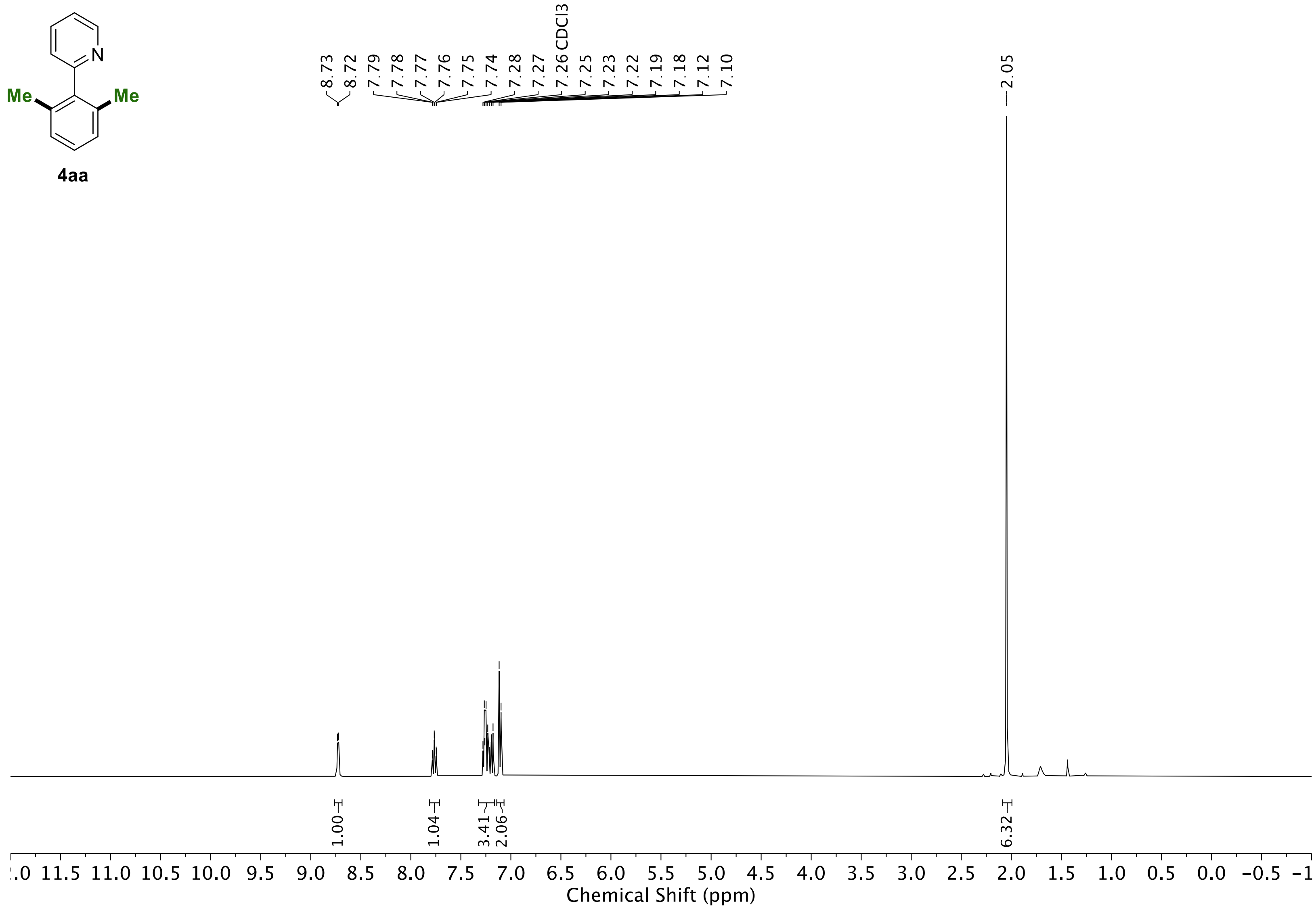

Figure S47. ^{13}C NMR (101 MHz, $CDCl_3$) of **4aa**.

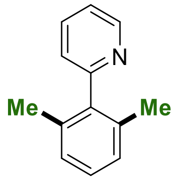

4aa

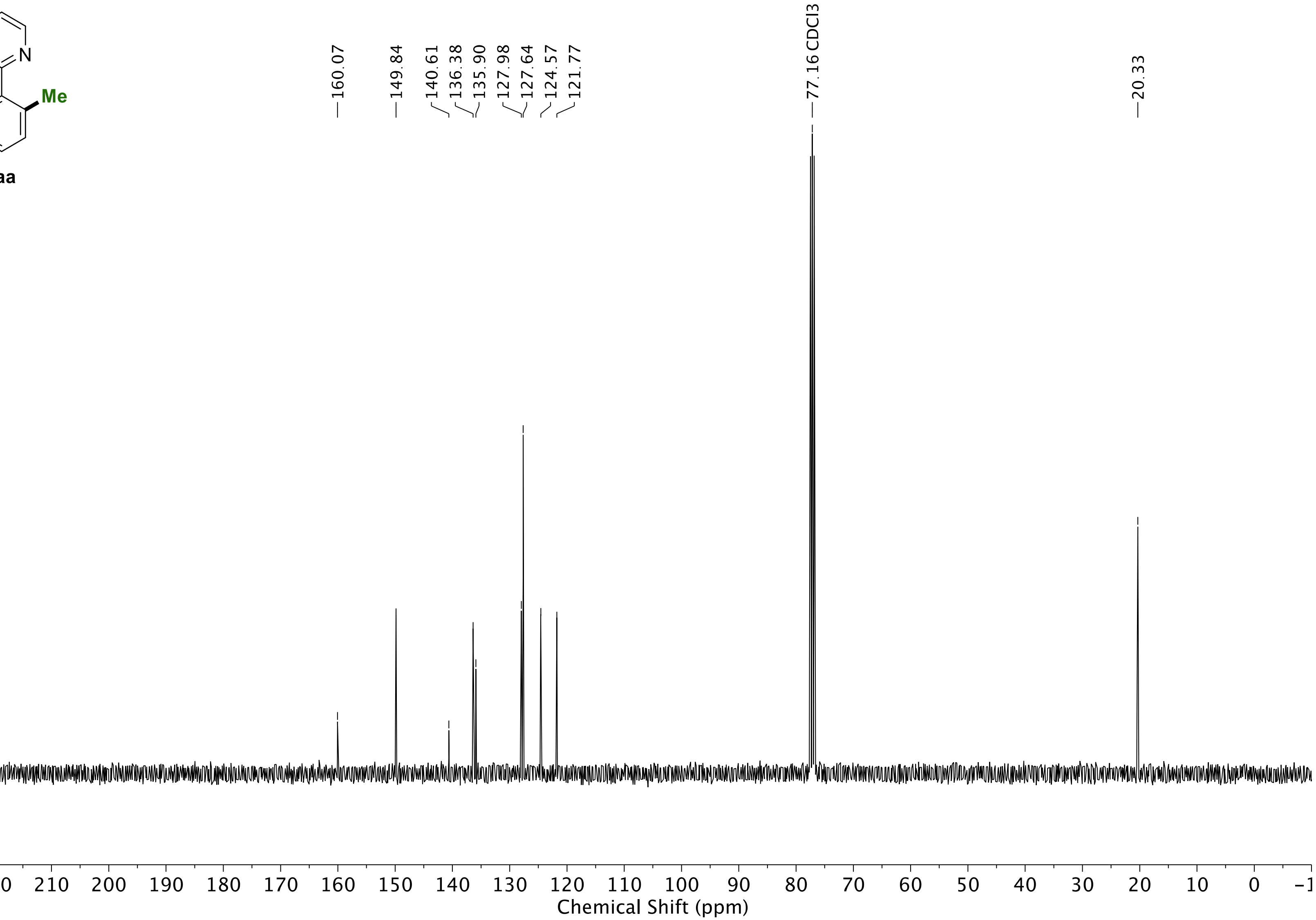

Figure S48. ^{1}H NMR (400 MHz, $CDCl_3$) of **3ba**.

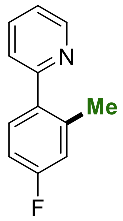

3ba

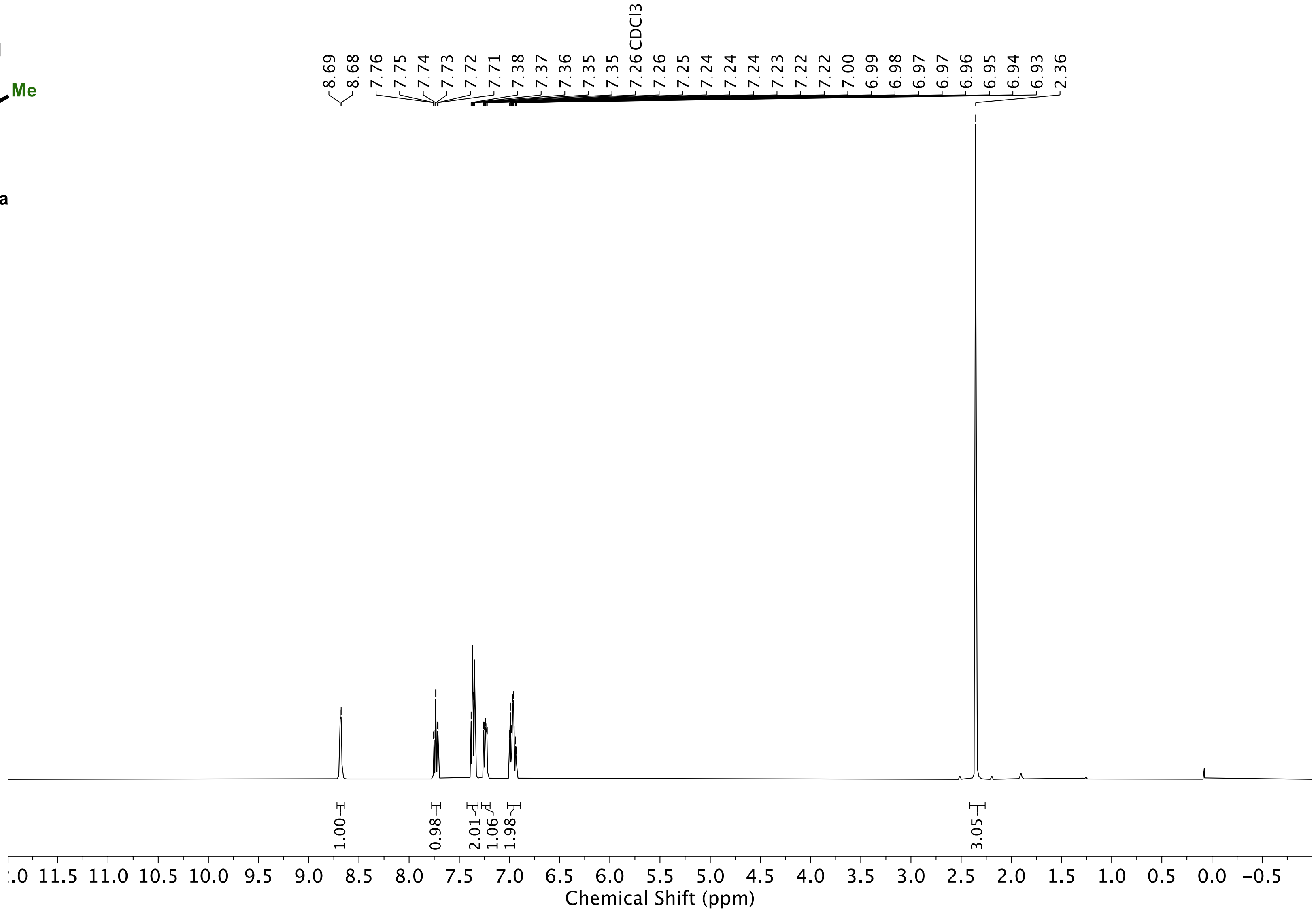

Figure S49. ^{13}C NMR (101 MHz, $CDCl_3$) of **3ba**.

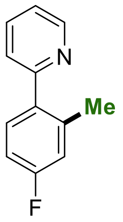

3ba

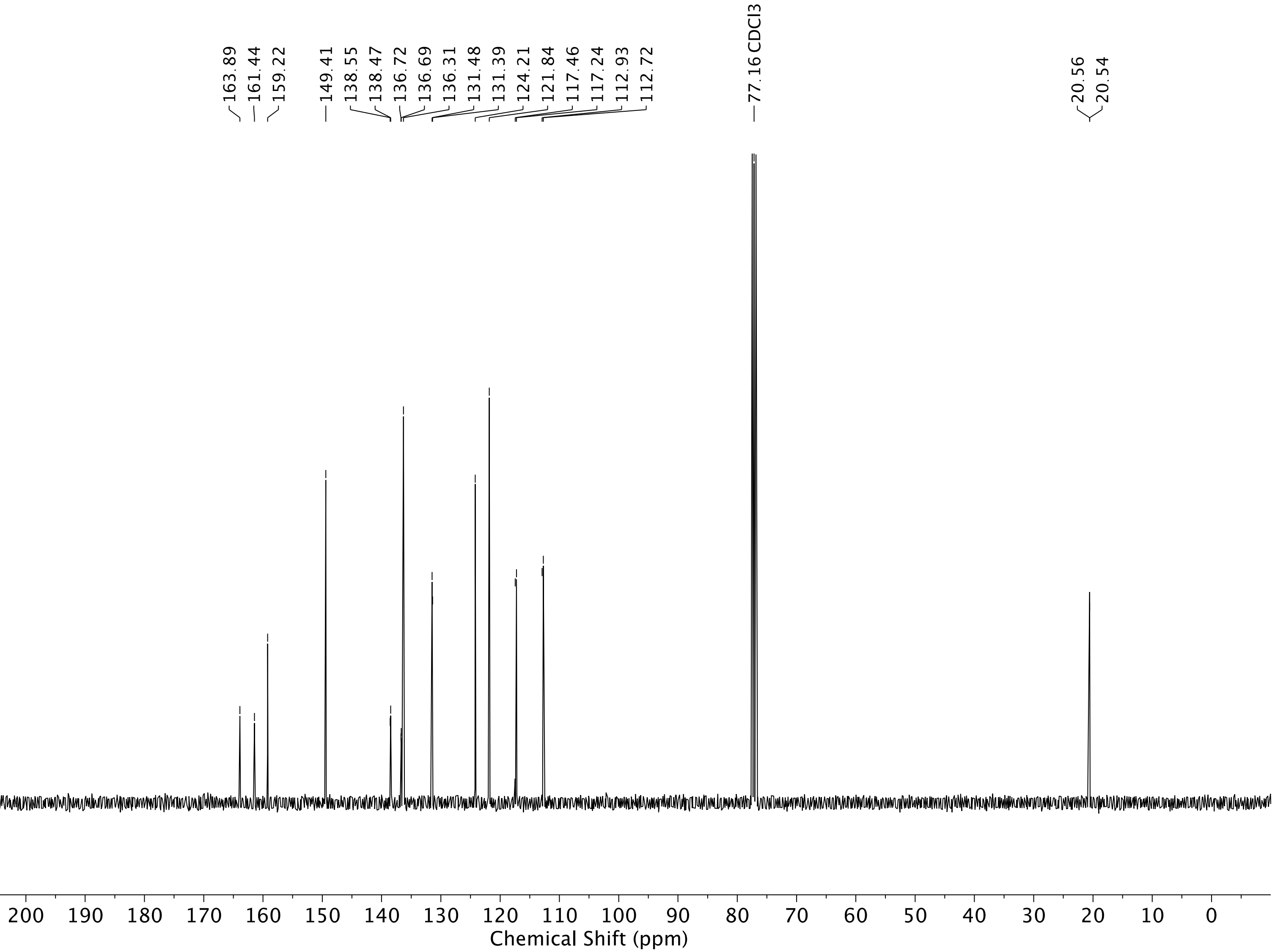

Figure S50. ^{19}F NMR (376 MHz, $CDCl_3$) of **3ba**.

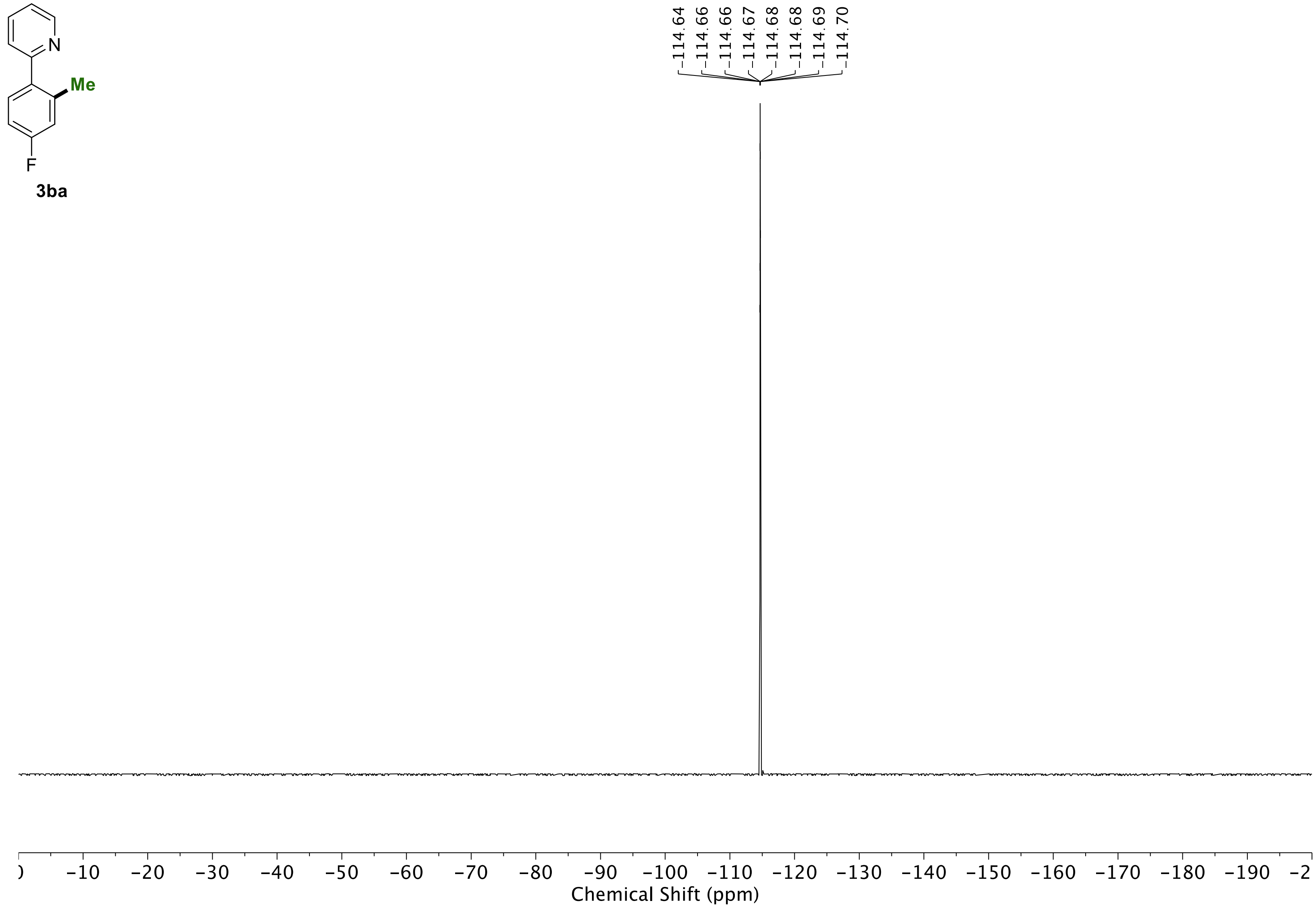

Figure S51. ^{1}H NMR (500 MHz, $CDCl_3$) of **4ba**.

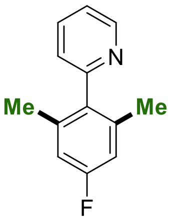

4ba

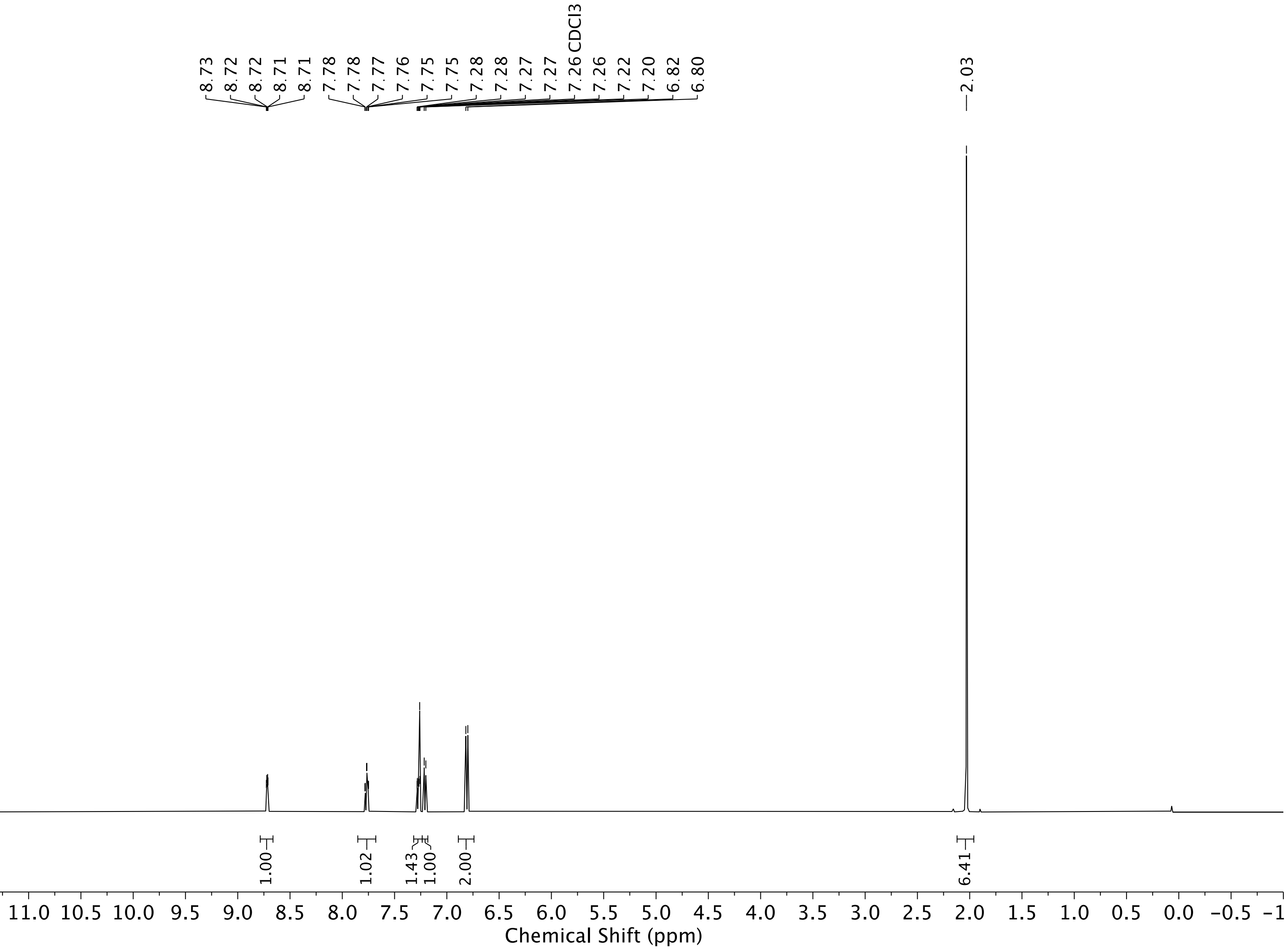

Figure S52. ^{13}C NMR (126 MHz, $CDCl_3$) of **4ba**.

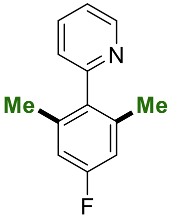

4ba

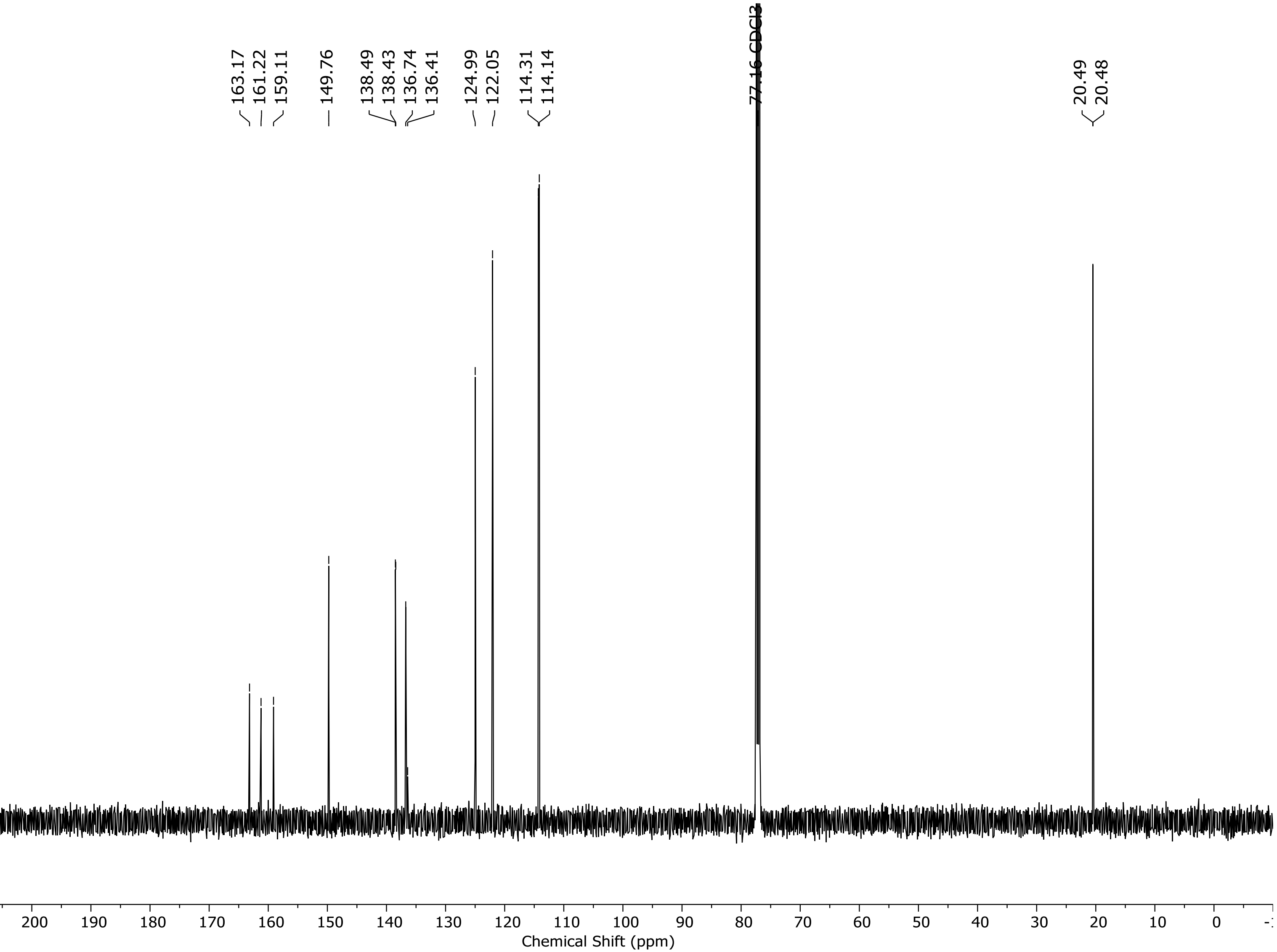

Figure S53. ^{19}F NMR (471 MHz, $CDCl_3$) of **4ba**.

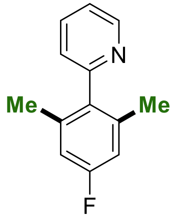

4ba

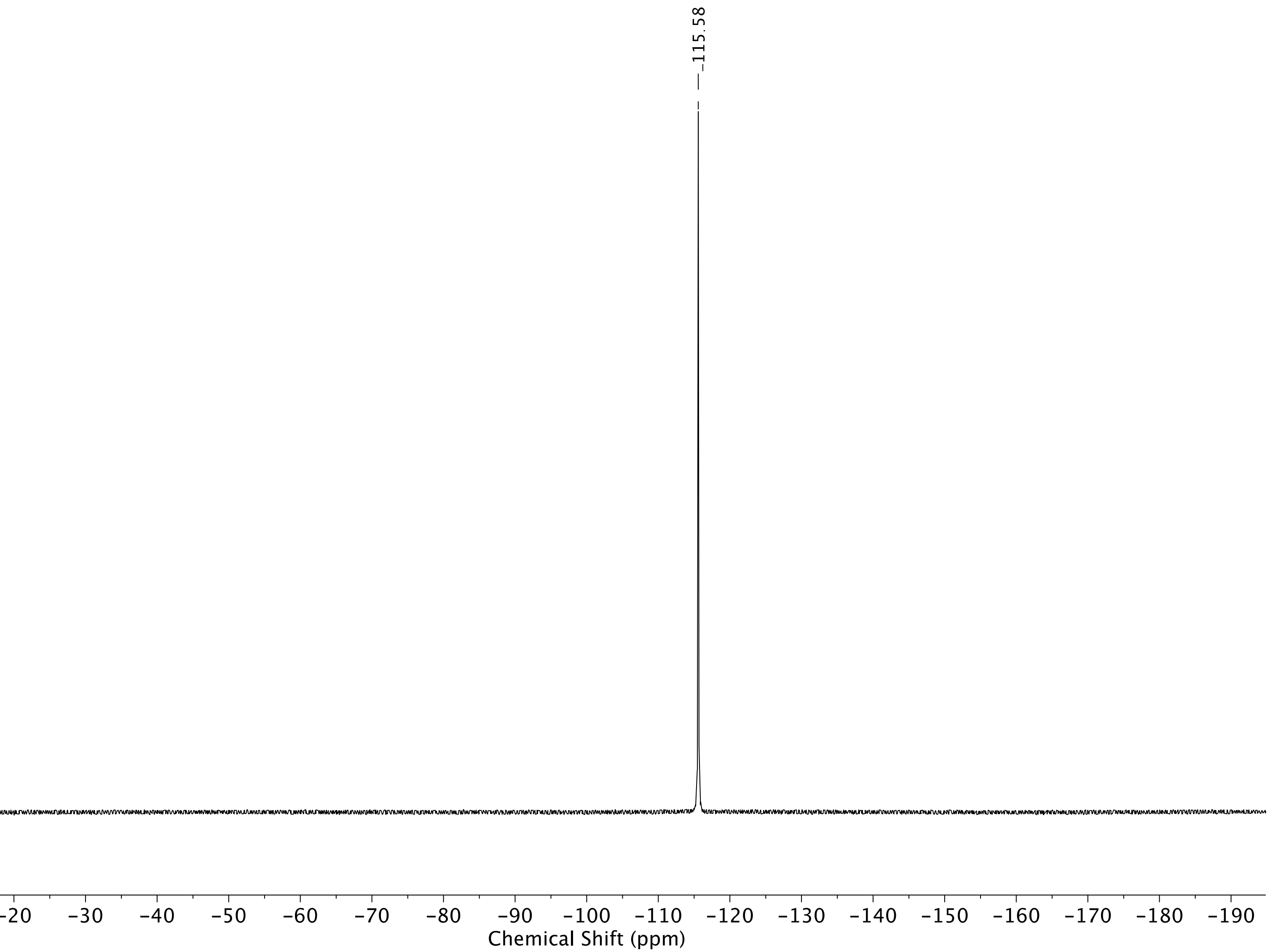

Figure S54. ^{1}H NMR (400 MHz, $CDCl_3$) of **3ca**.

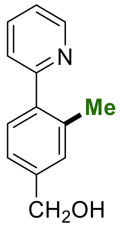

3ca

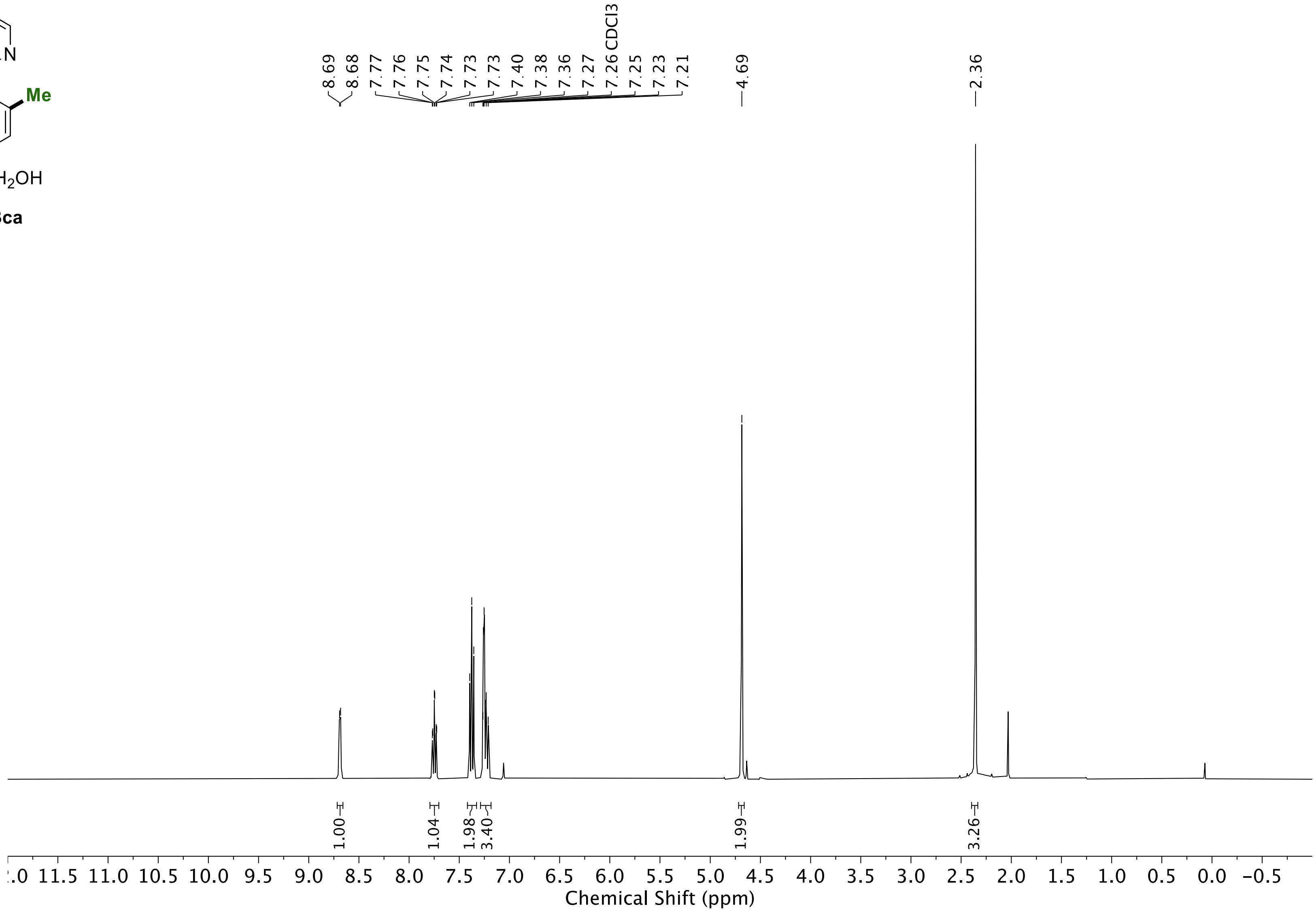

Figure S55. ^{13}C NMR (101 MHz, $CDCl_3$) of **3ca**.

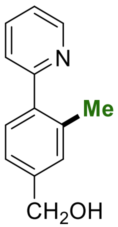

3ca

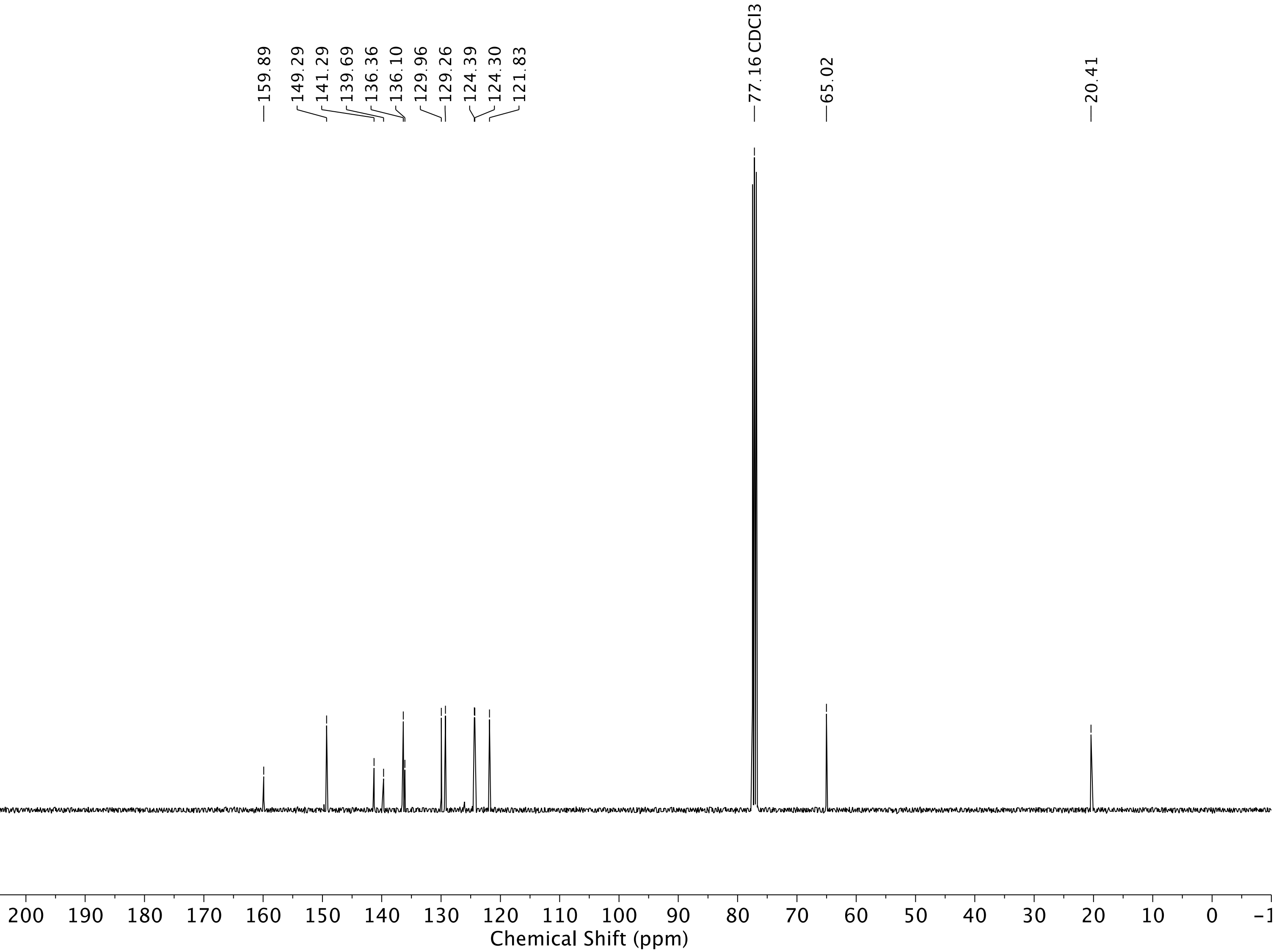

Figure S56. FT-IR spectrum (neat/cm^{-1}) of **3ca**.

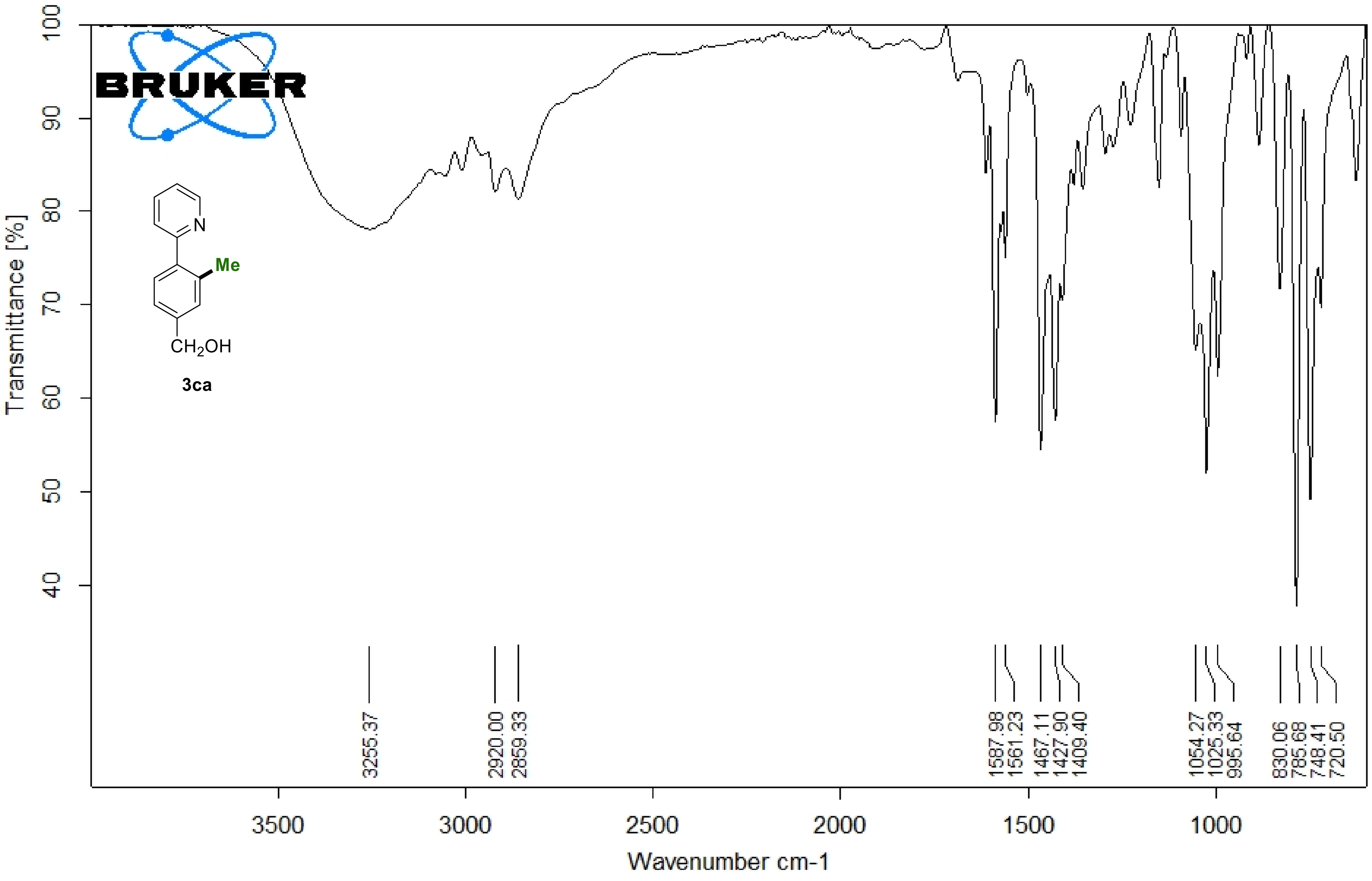

Figure S57. ^{1}H NMR (400 MHz, $CDCl_3$) of **3da**.

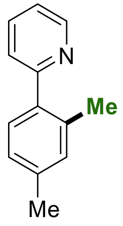

3da

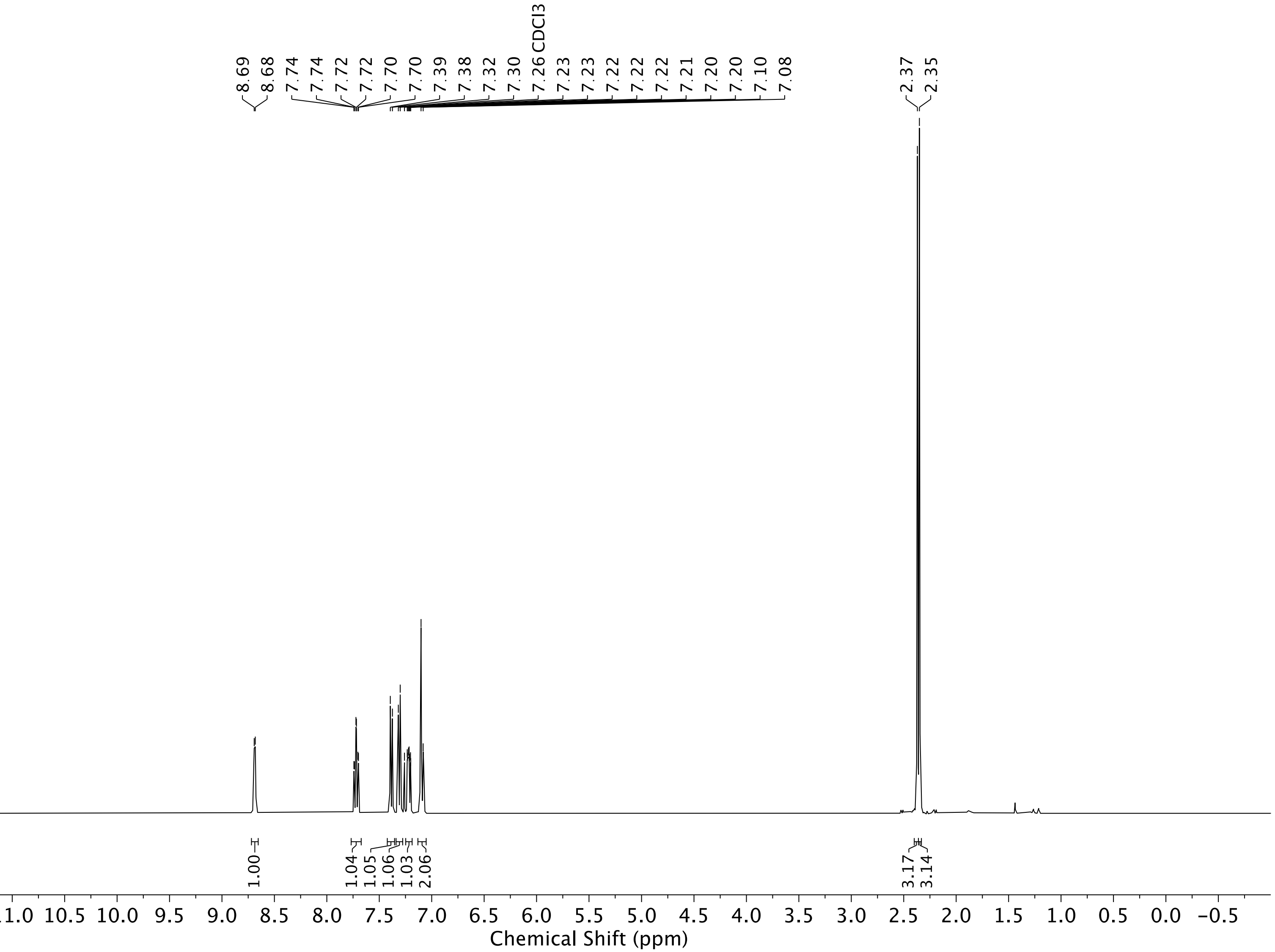

Figure S58. ^{13}C NMR (101 MHz, $CDCl_3$) of **3da**.

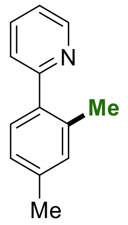

3da

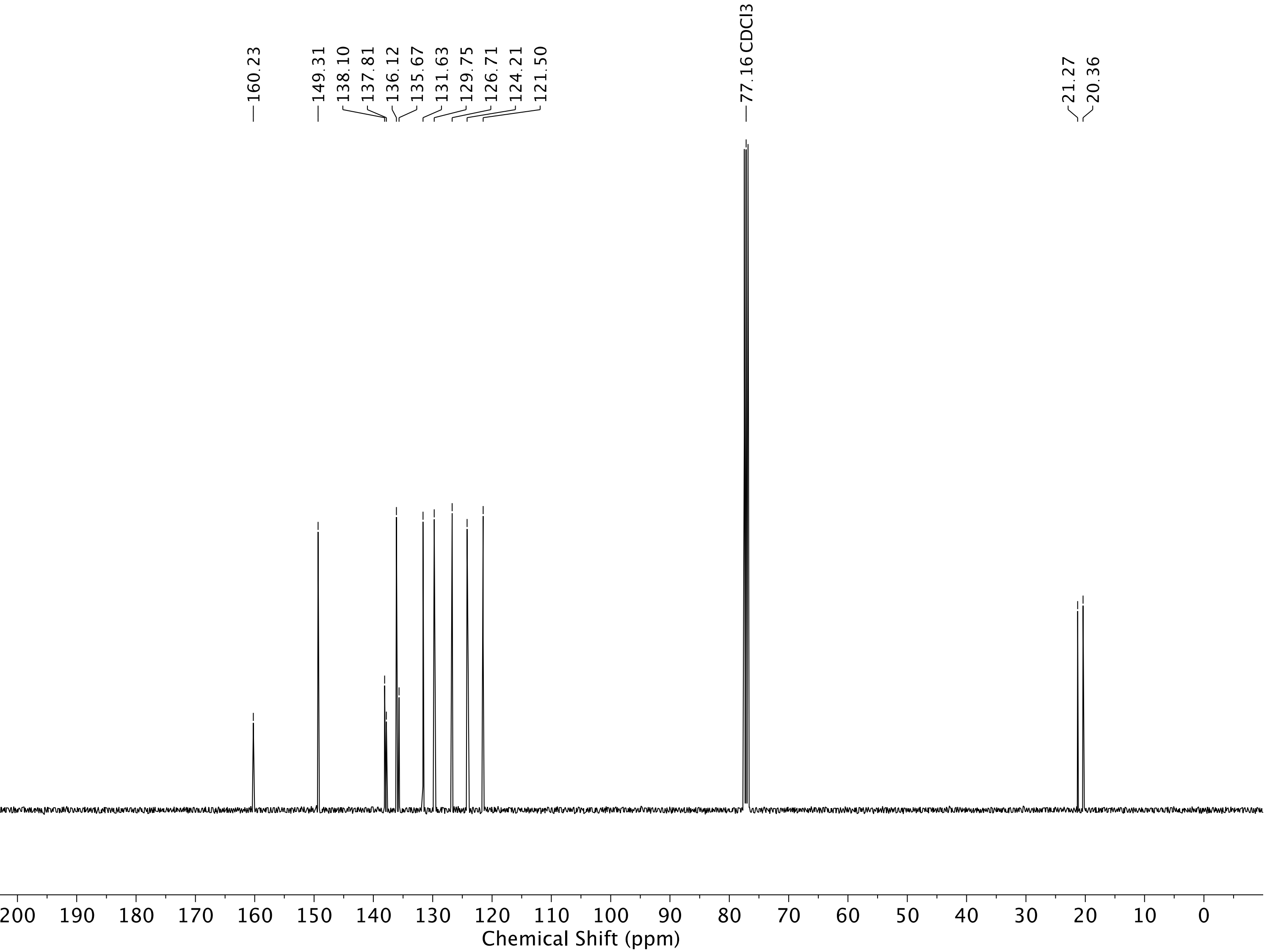

Figure S59. ^{1}H NMR (400 MHz, $CDCl_3$) of **3ea.**

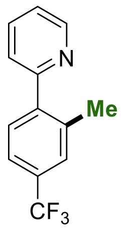

3ea

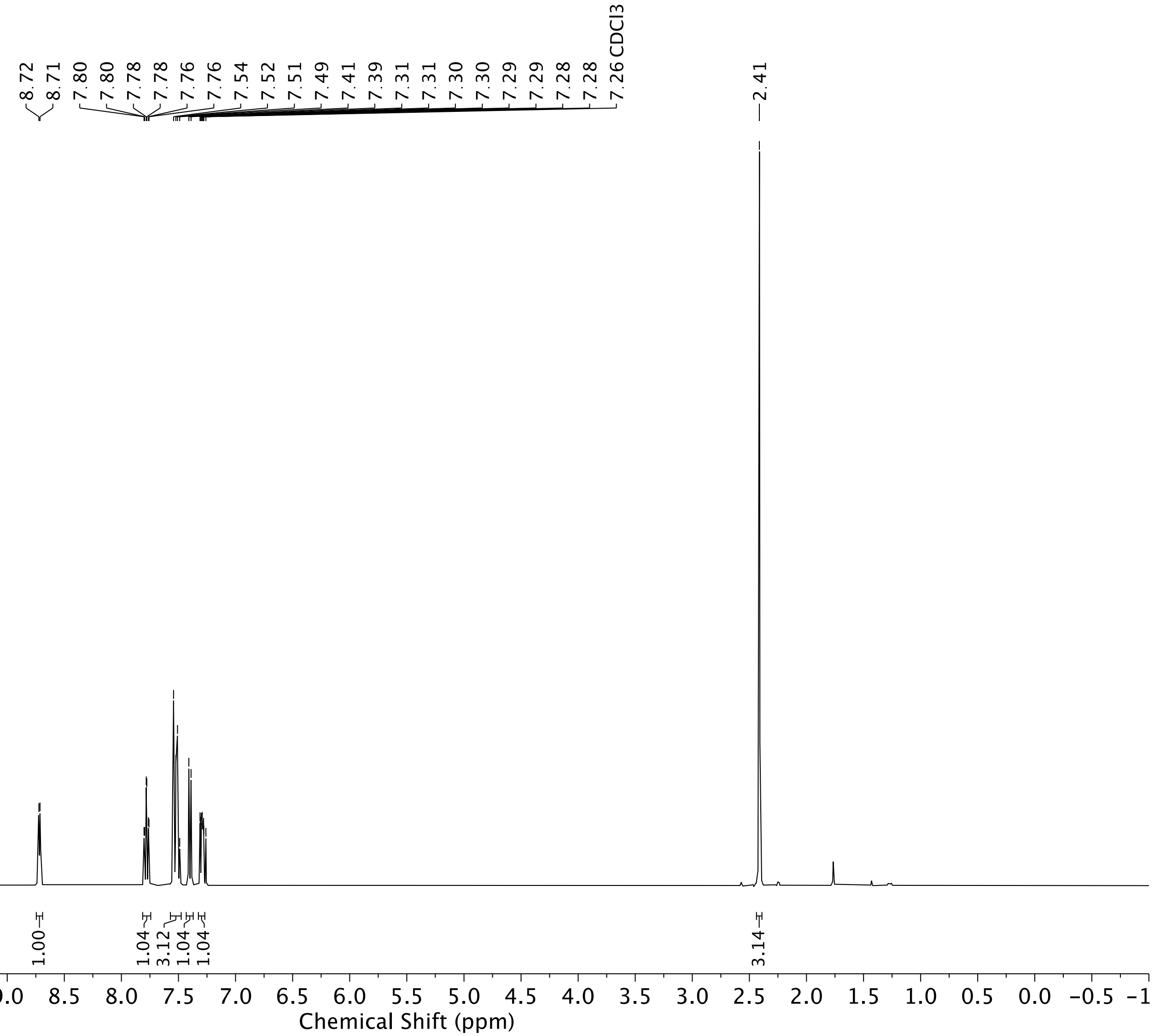

Figure S60. ^{13}C NMR (101 MHz, $CDCl_3$) of **3ea**.

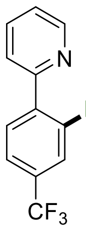

3ea

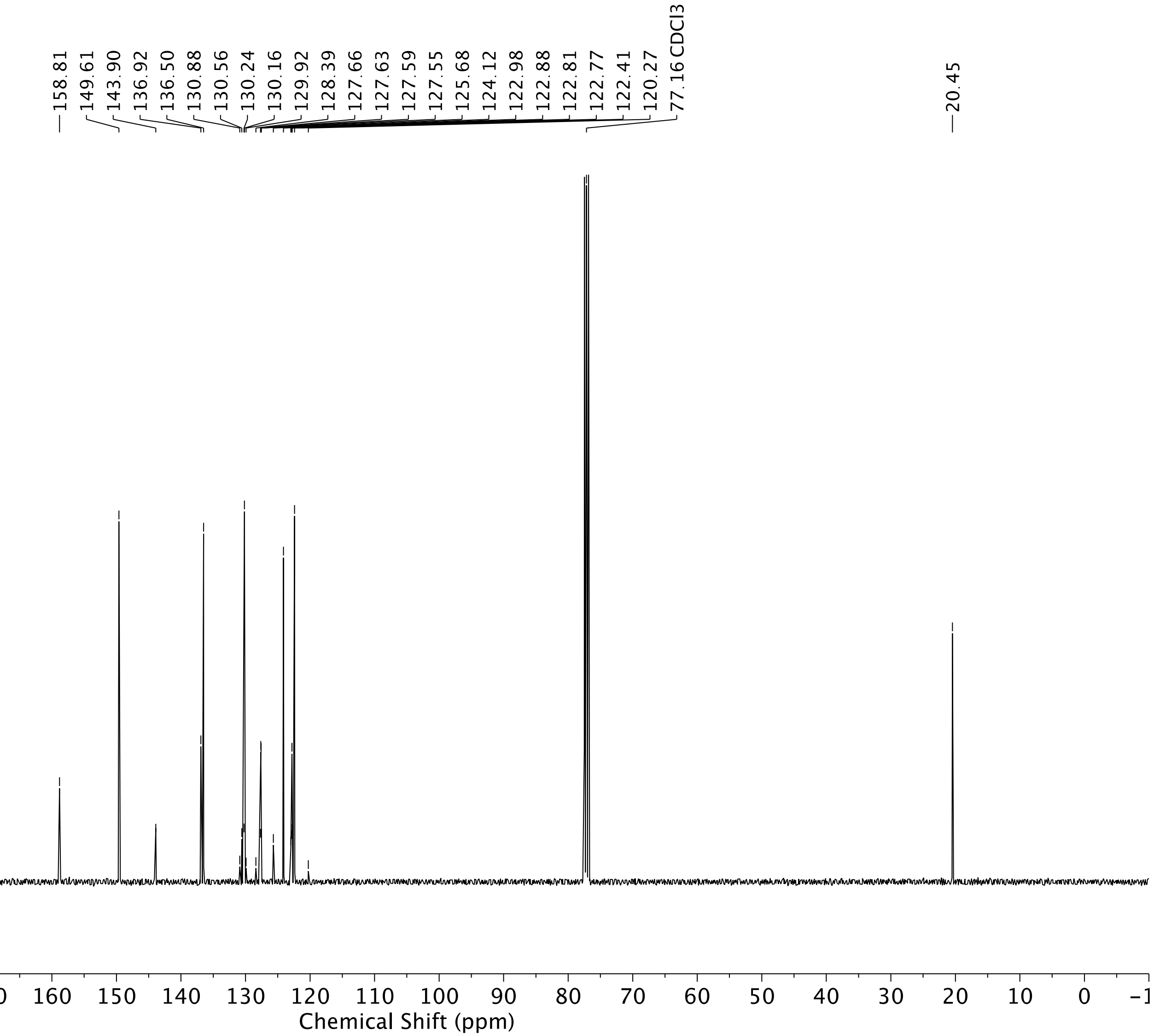

Figure S61. ^{19}F NMR (471 MHz, $CDCl_3$) of **3ea**.

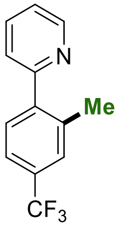

3ea

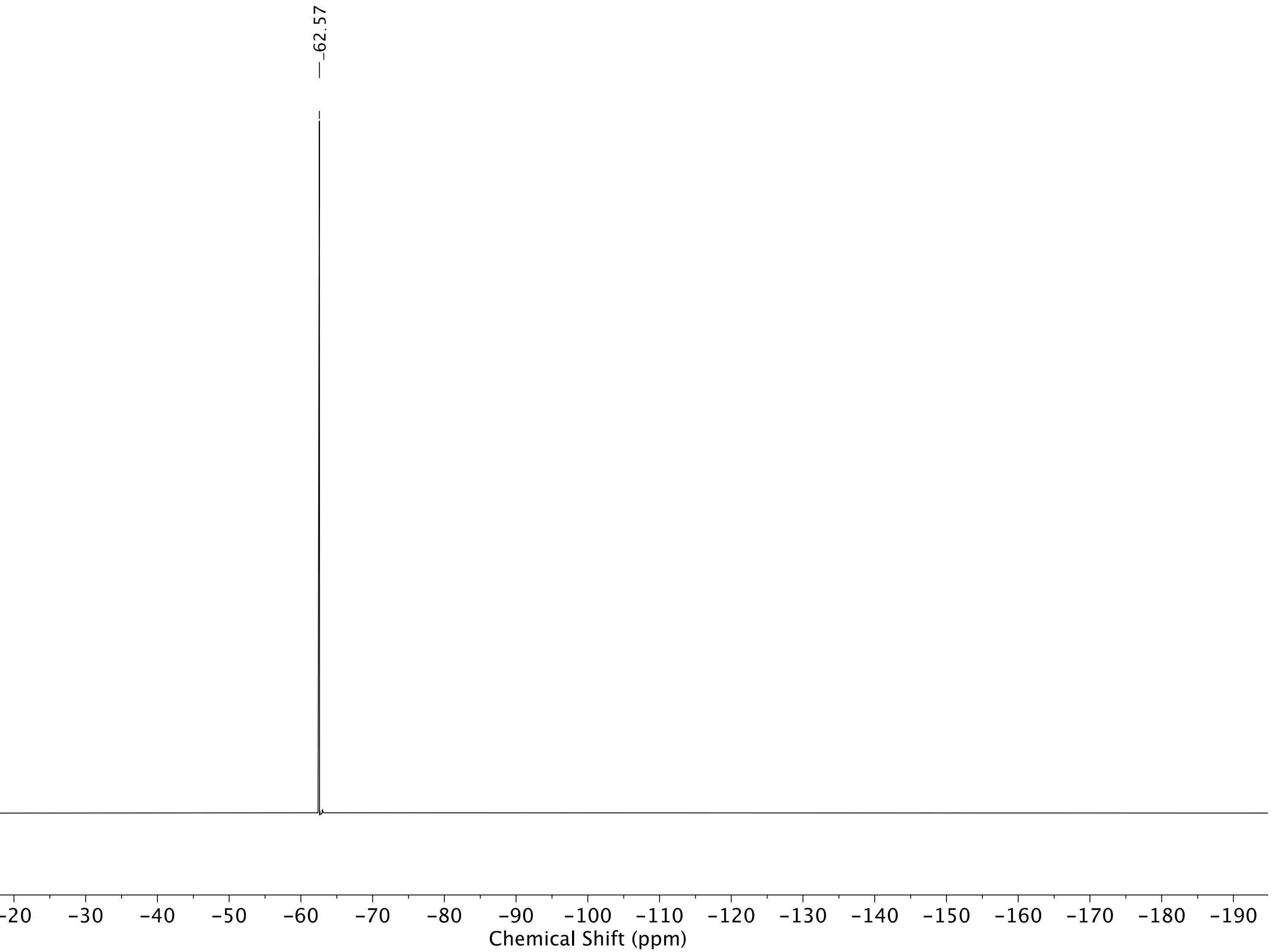

Figure S62. ^{1}H NMR (400 MHz, $CDCl_3$) of **4ea.**

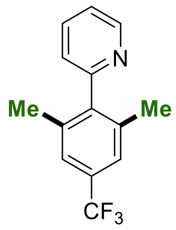

4ea

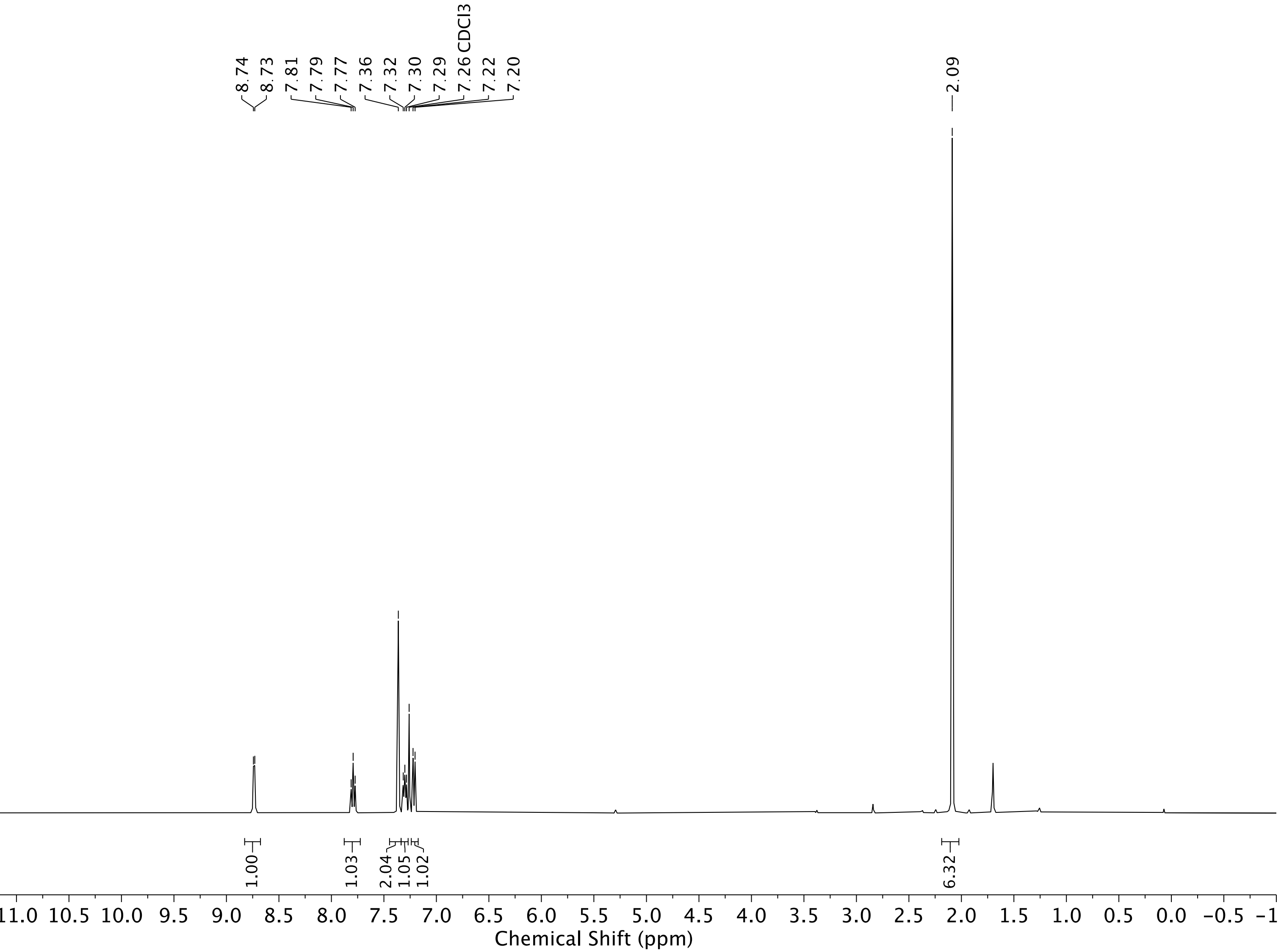

Figure S63. ^{13}C NMR (101 MHz, $CDCl_3$) of **4ea**.

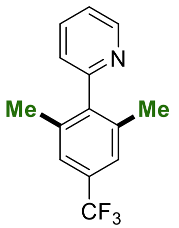

4ea

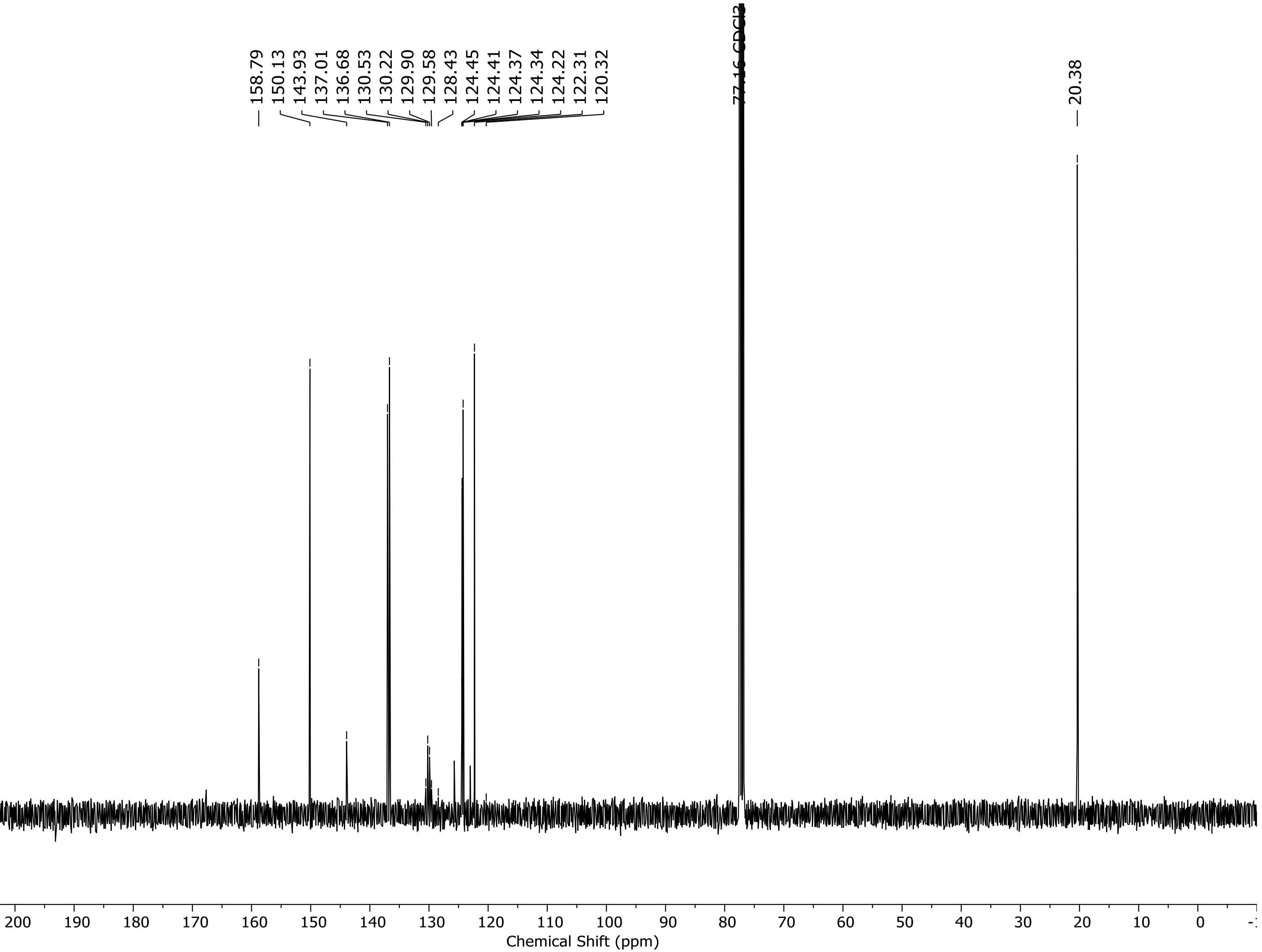

Figure S64. ^{19}F NMR (371 MHz, $CDCl_3$) of **4ea**.

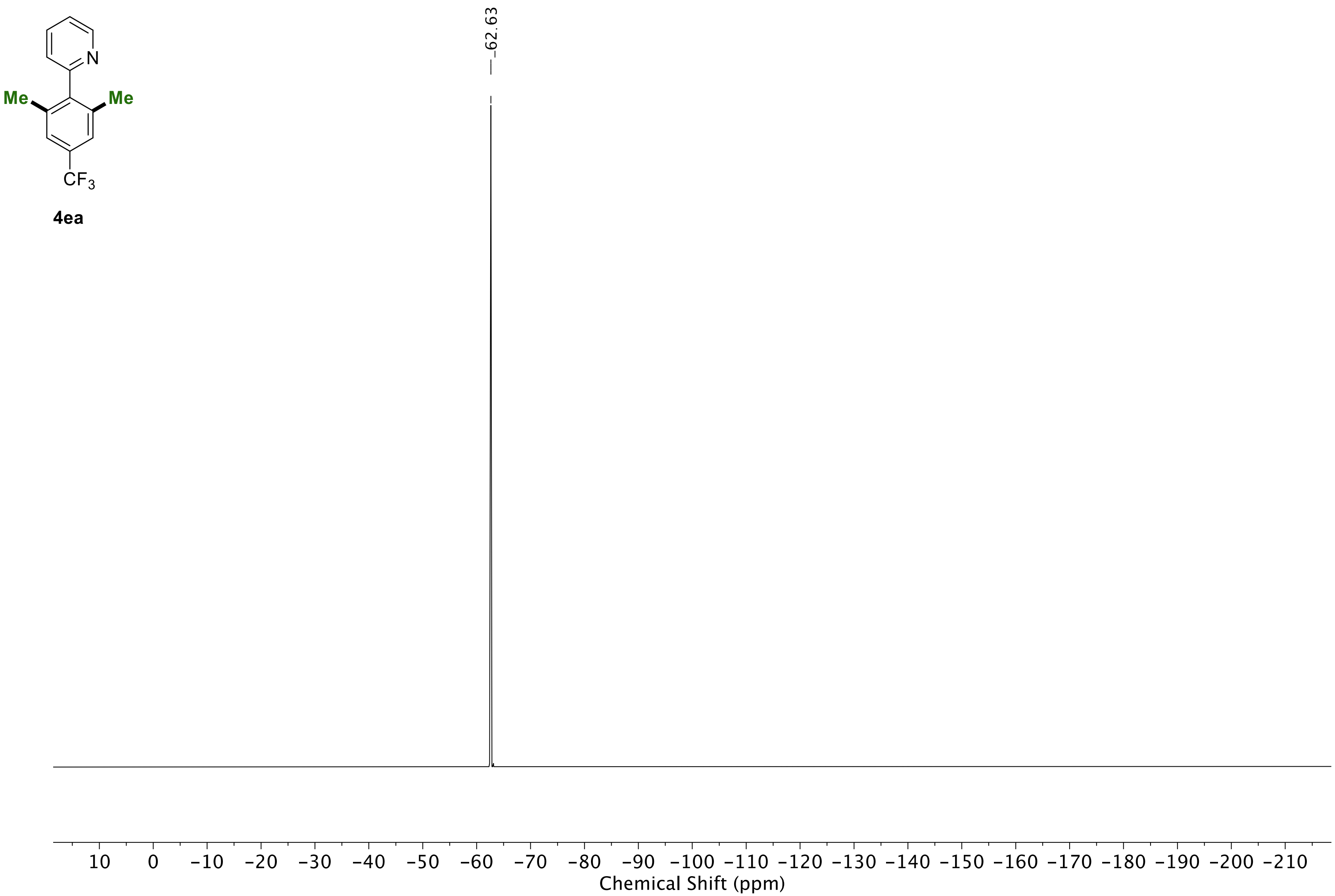

Figure S65. H NMR (400 MHz, $CDCl_3$) of **3fa**.

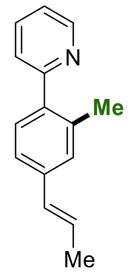

3fa

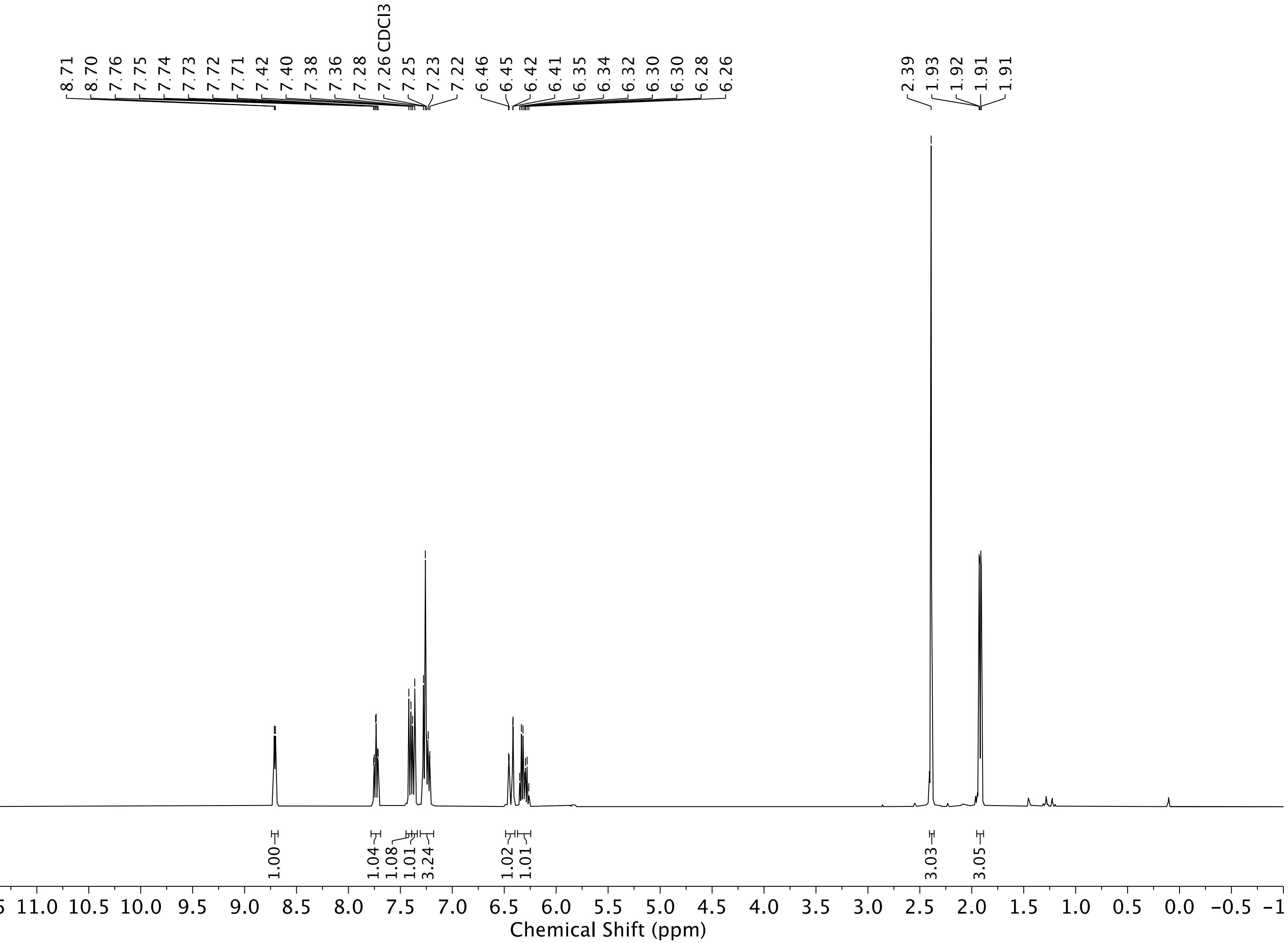

Figure S66. ^{13}C NMR (101 MHz, $CDCl_3$) of **3fa**.

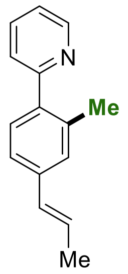

3fa

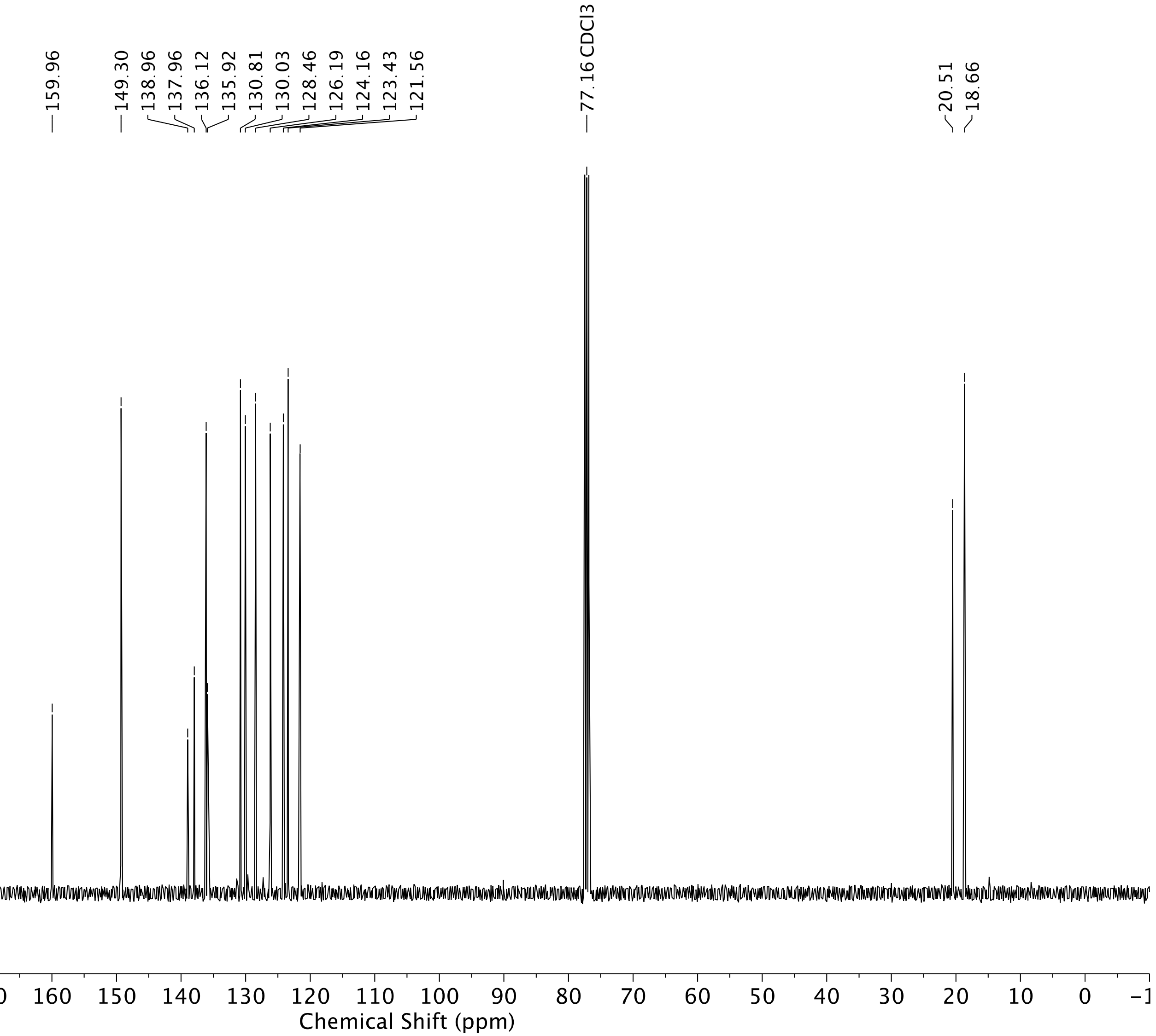

Figure S67. ^{1}H NMR (400 MHz, $CDCl_3$) of **3ga**.

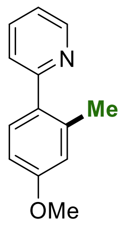

3ga

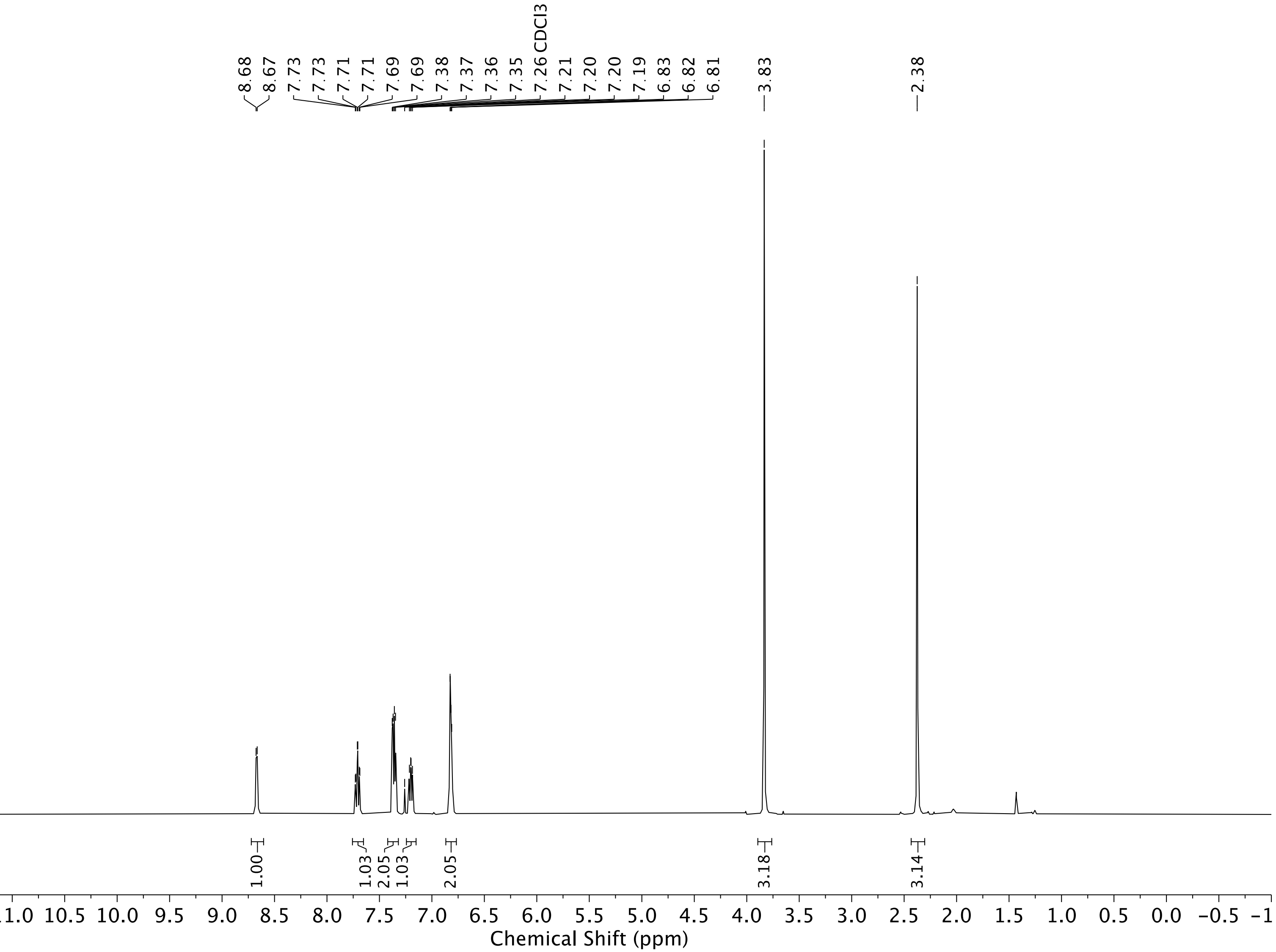

Figure S68. ^{13}C NMR (101 MHz, $CDCl_3$) of **3ga**.

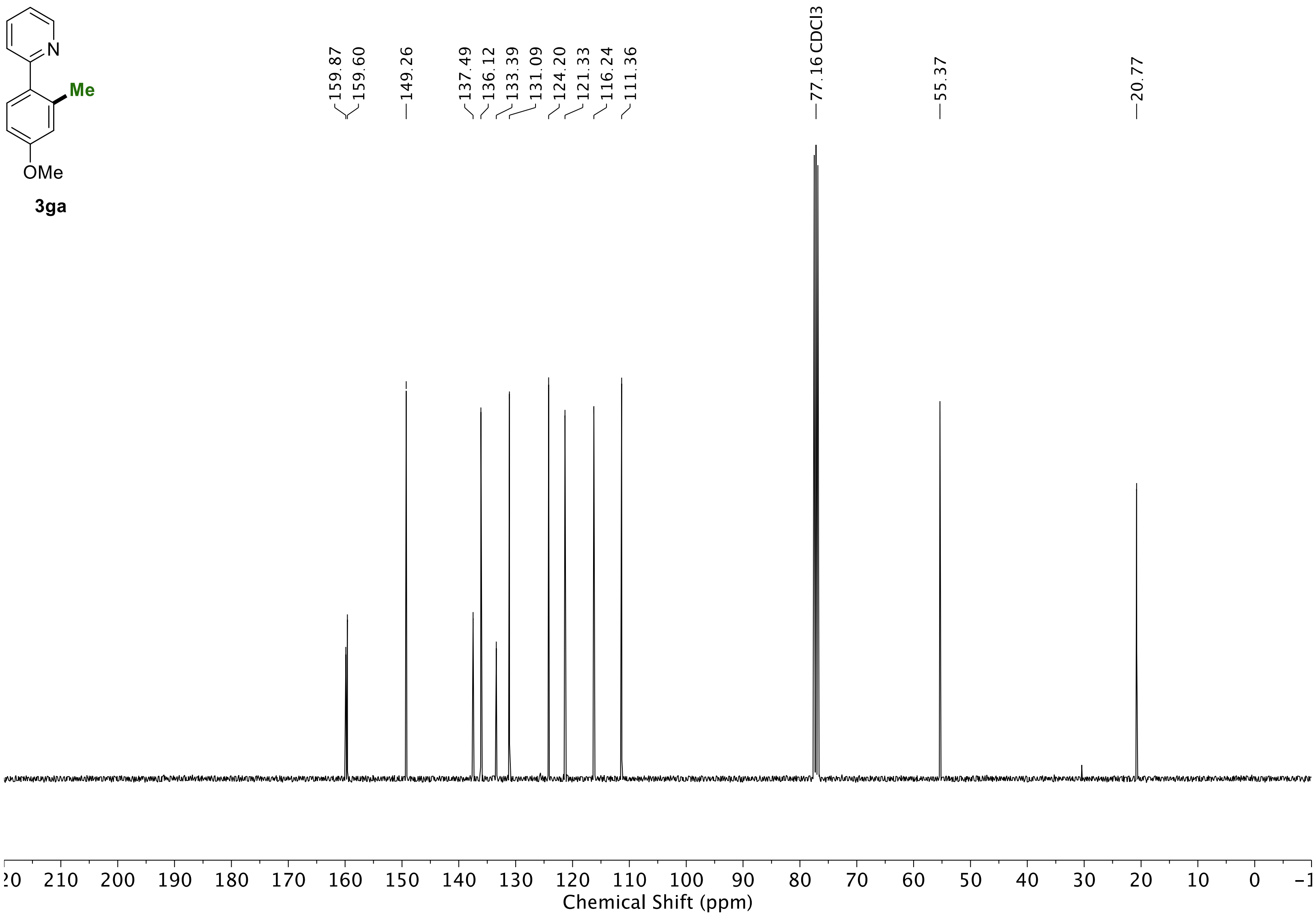

Figure S69. ^{1}H NMR (400 MHz, $CDCl_3$) of **4ga**.

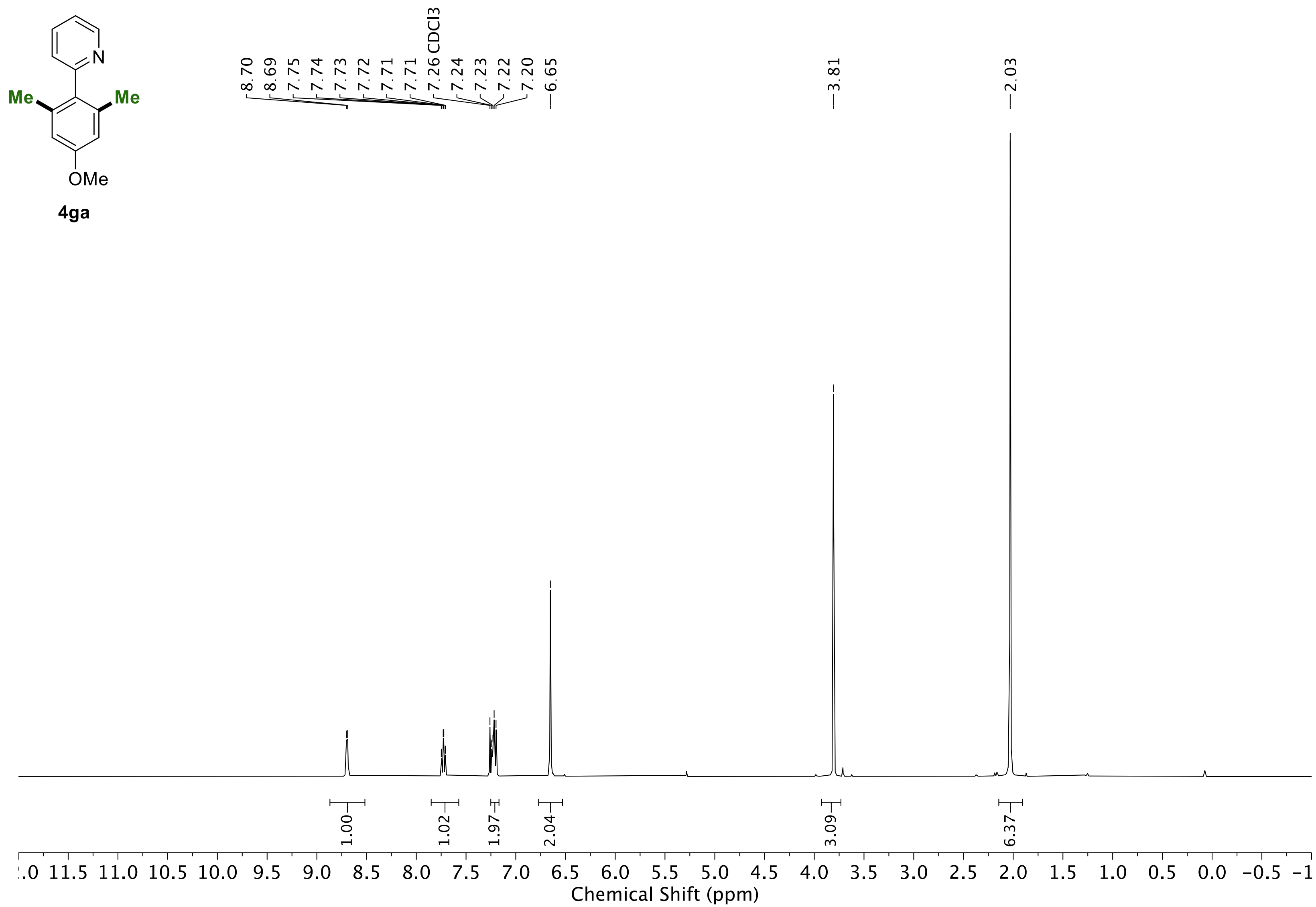

Figure S70. ^{13}C NMR (101 MHz, $CDCl_3$) of **4ga**.

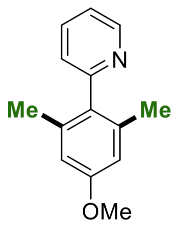

4ga

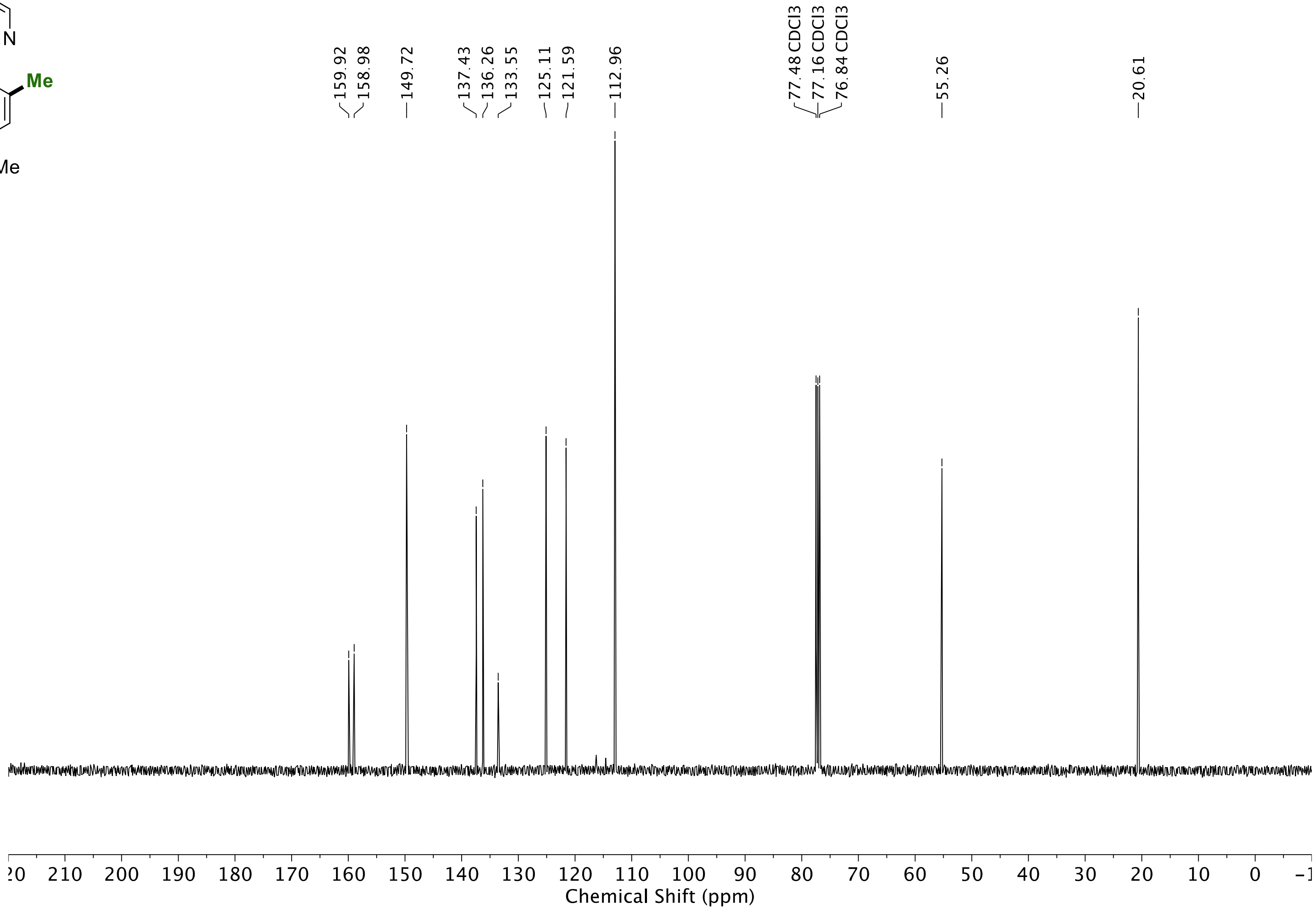

Figure S71. ^{1}H NMR (400 MHz, $CDCl_3$) of **3ha**.

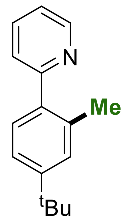

3ha

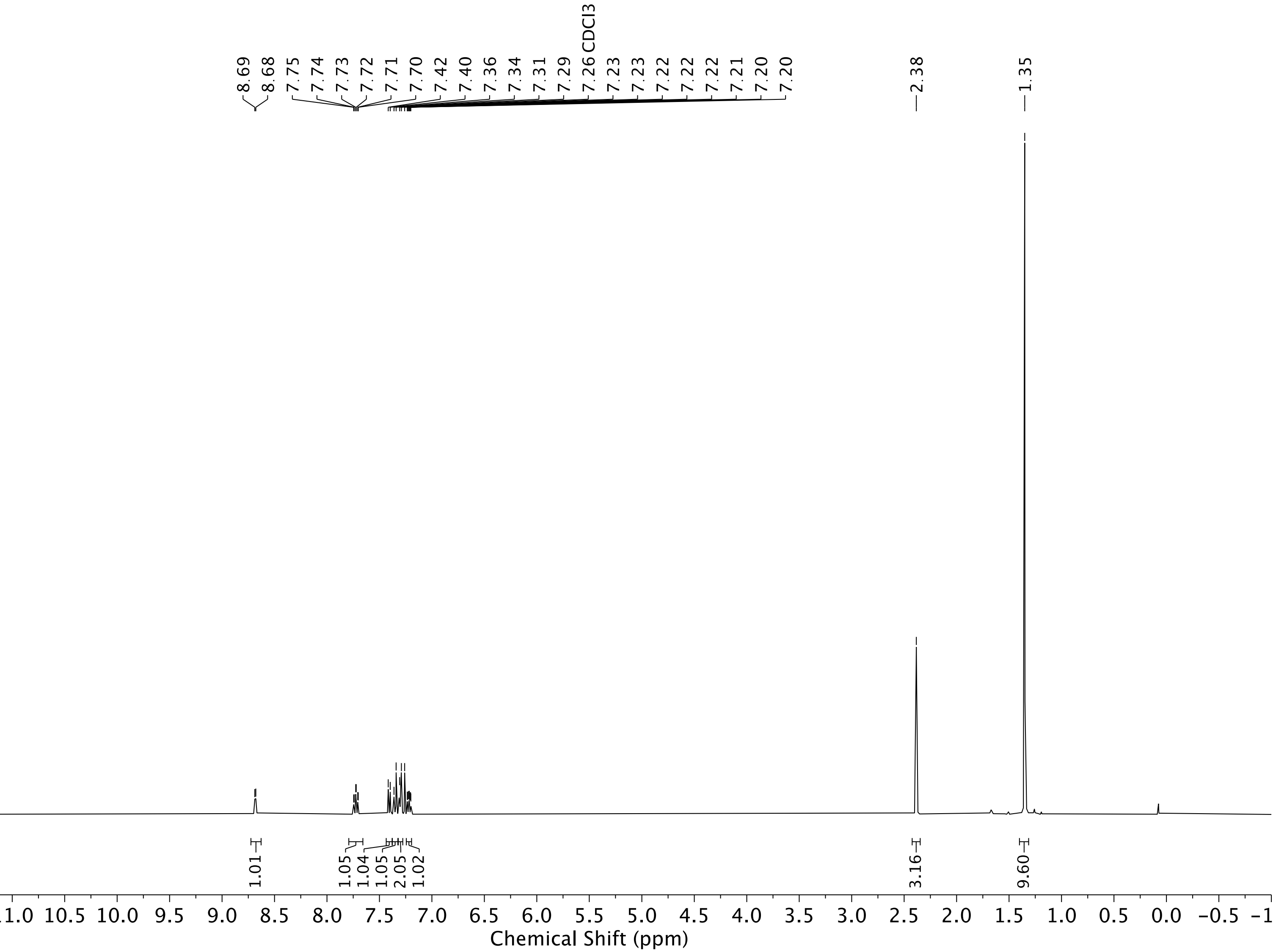

Figure S72. ^{13}C NMR (126 MHz, $CDCl_3$) of **3ha**.

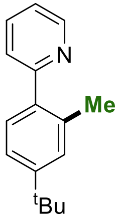

3ha

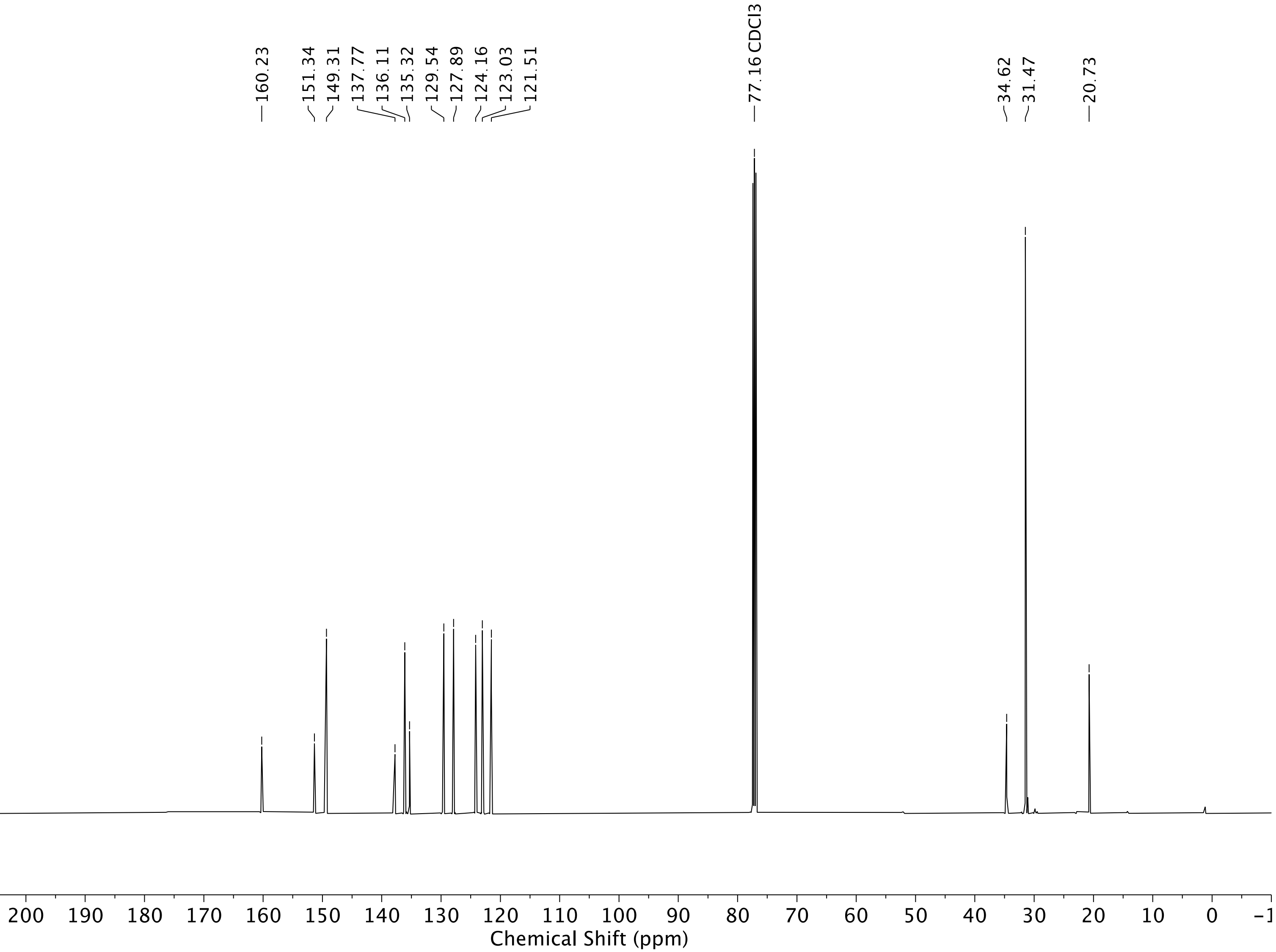

Figure S73. ^{1}H NMR (400 MHz, $CDCl_3$) of **3ia**.

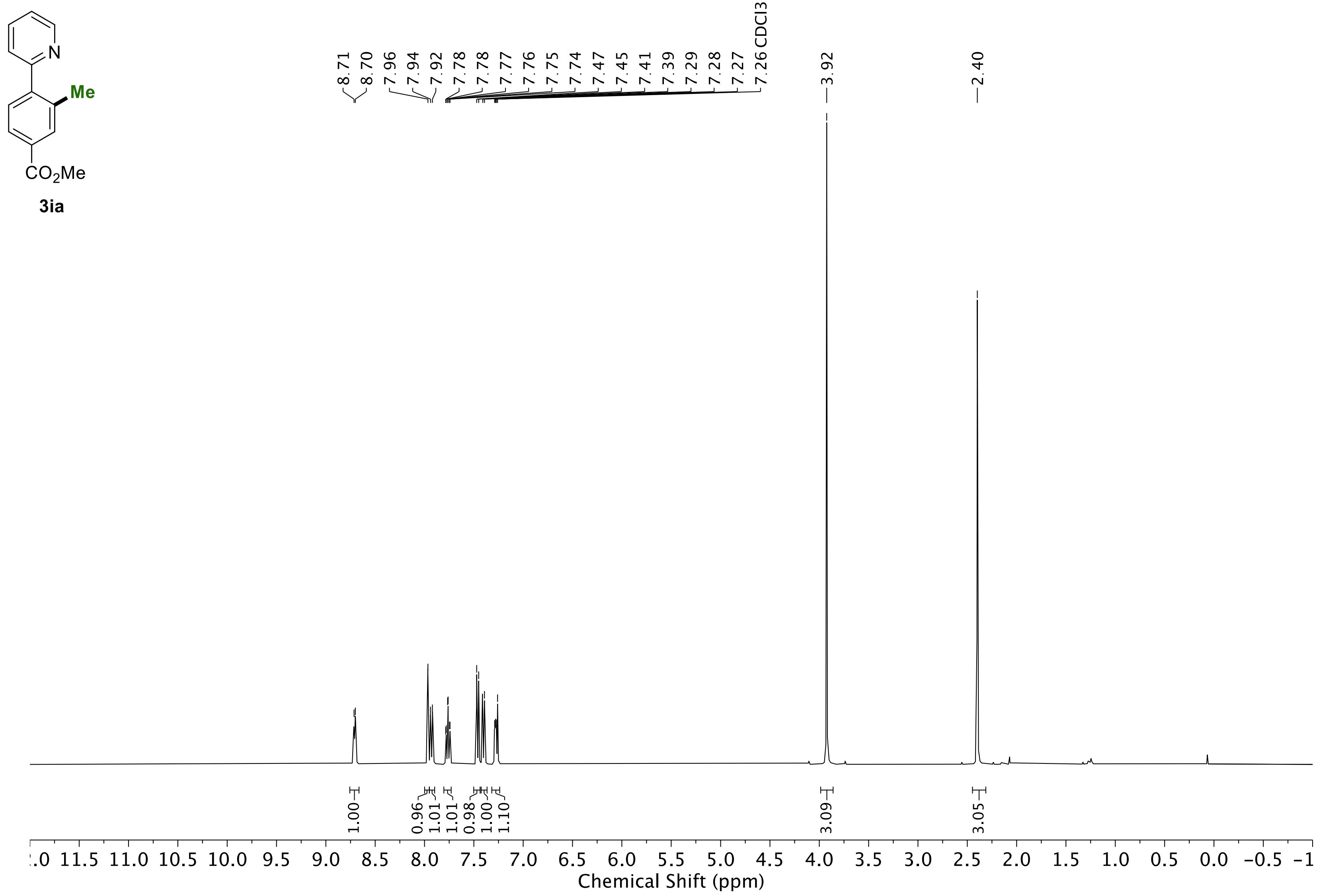

Figure S74. ^{13}C NMR (101 MHz, $CDCl_3$) of **3ia**.

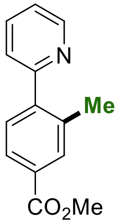

3ia

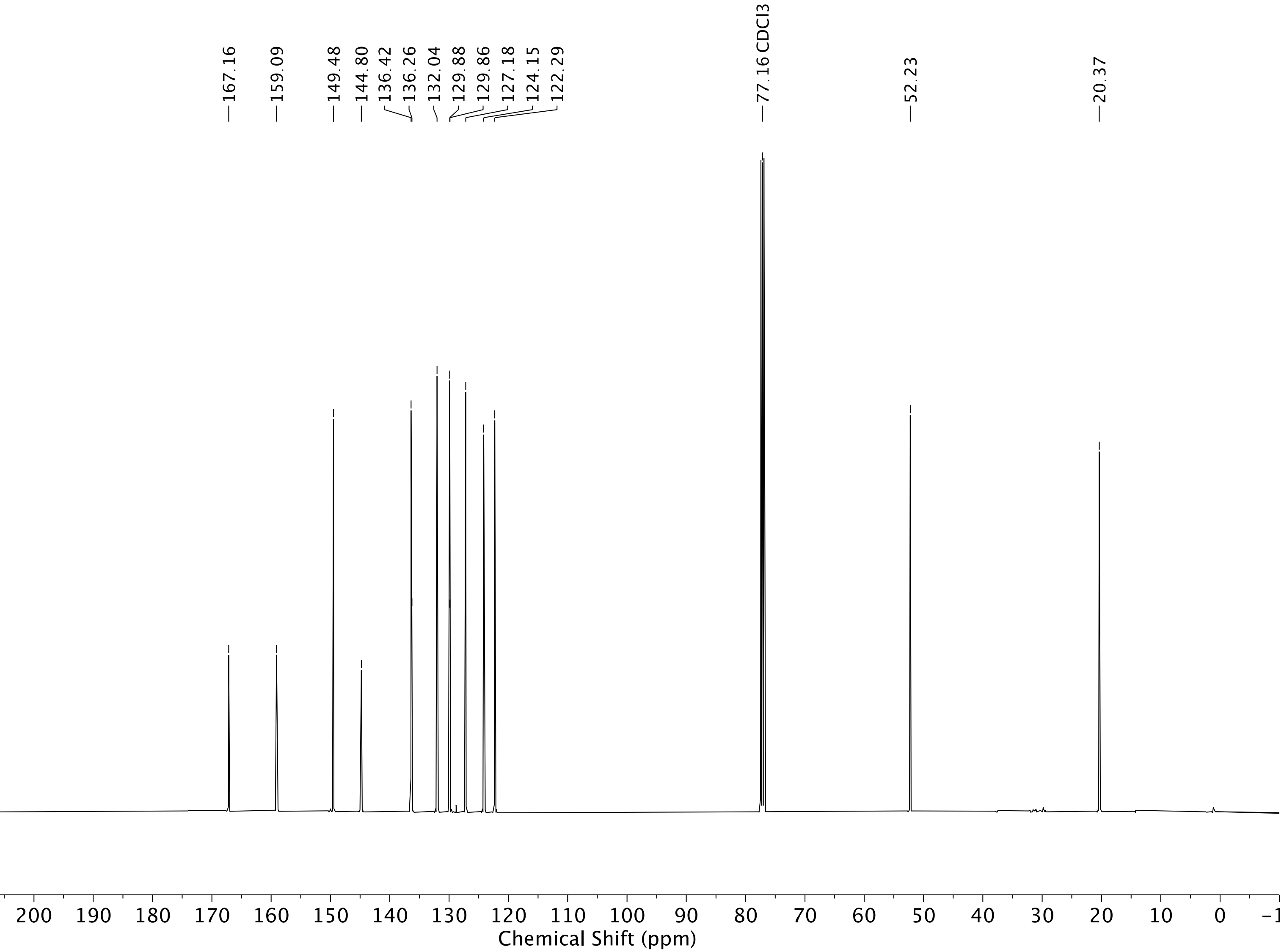

Figure S75. ^{1}H NMR (500 MHz, $CDCl_3$) of **3ja**.

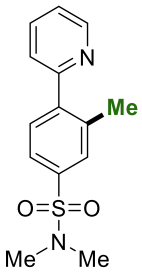

3ja

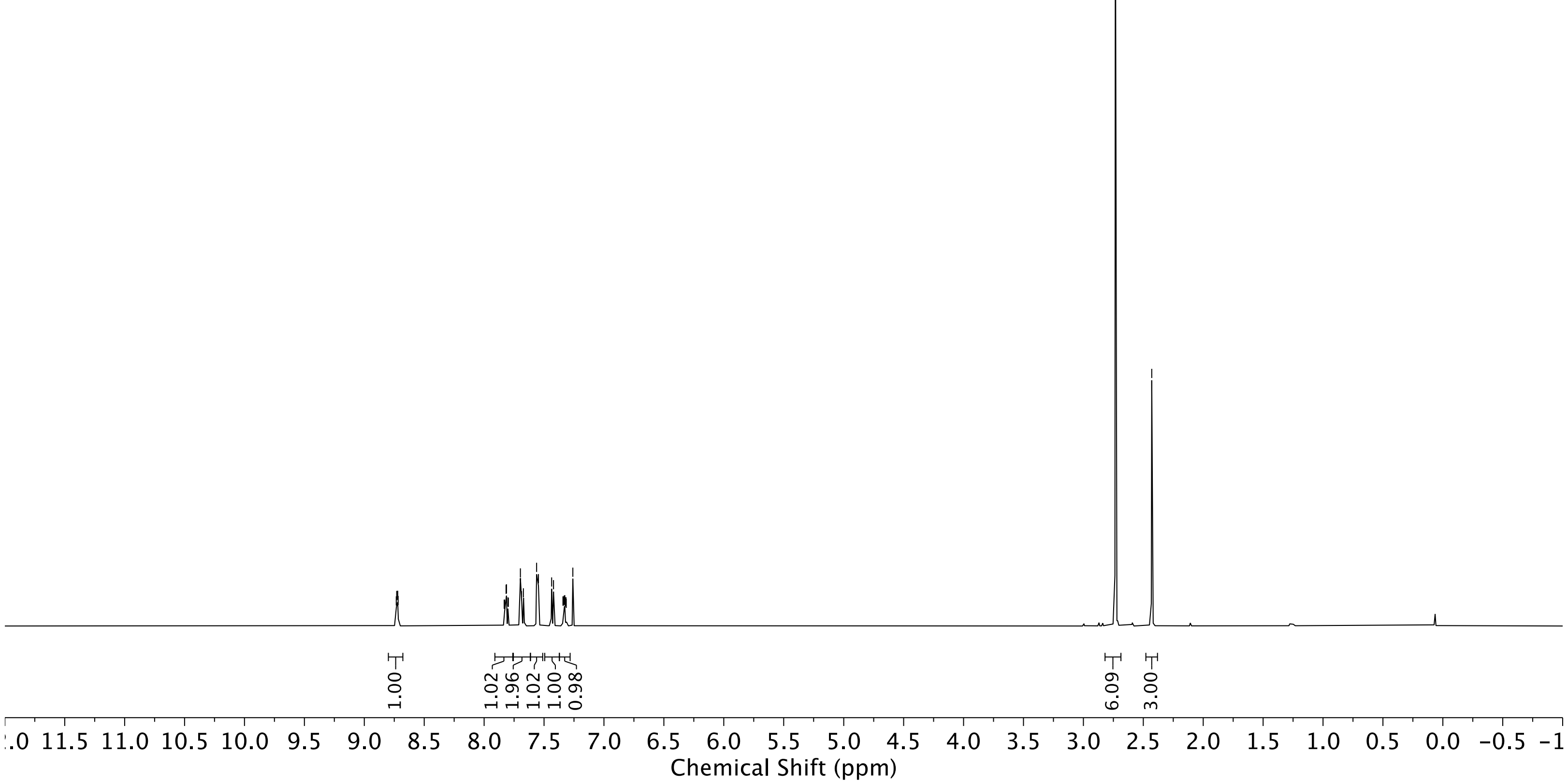

Figure S76. ^{13}C NMR (126 MHz, $CDCl_3$) of **3ja**.

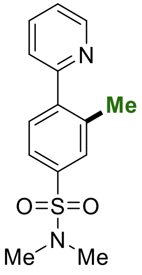

3ja

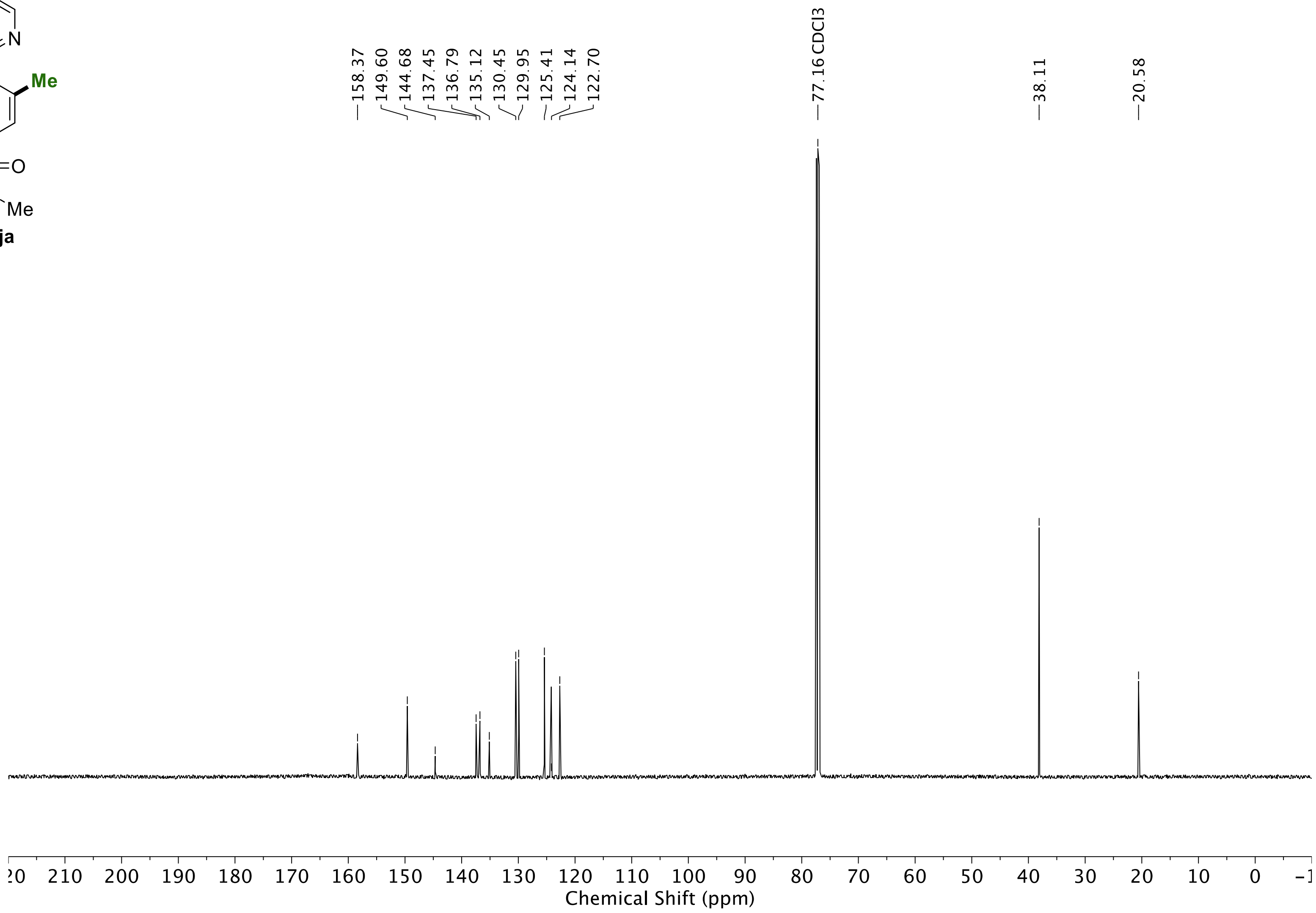

Figure S77. ^{1}H NMR (400 MHz, $CDCl_3$) of **3ka**.

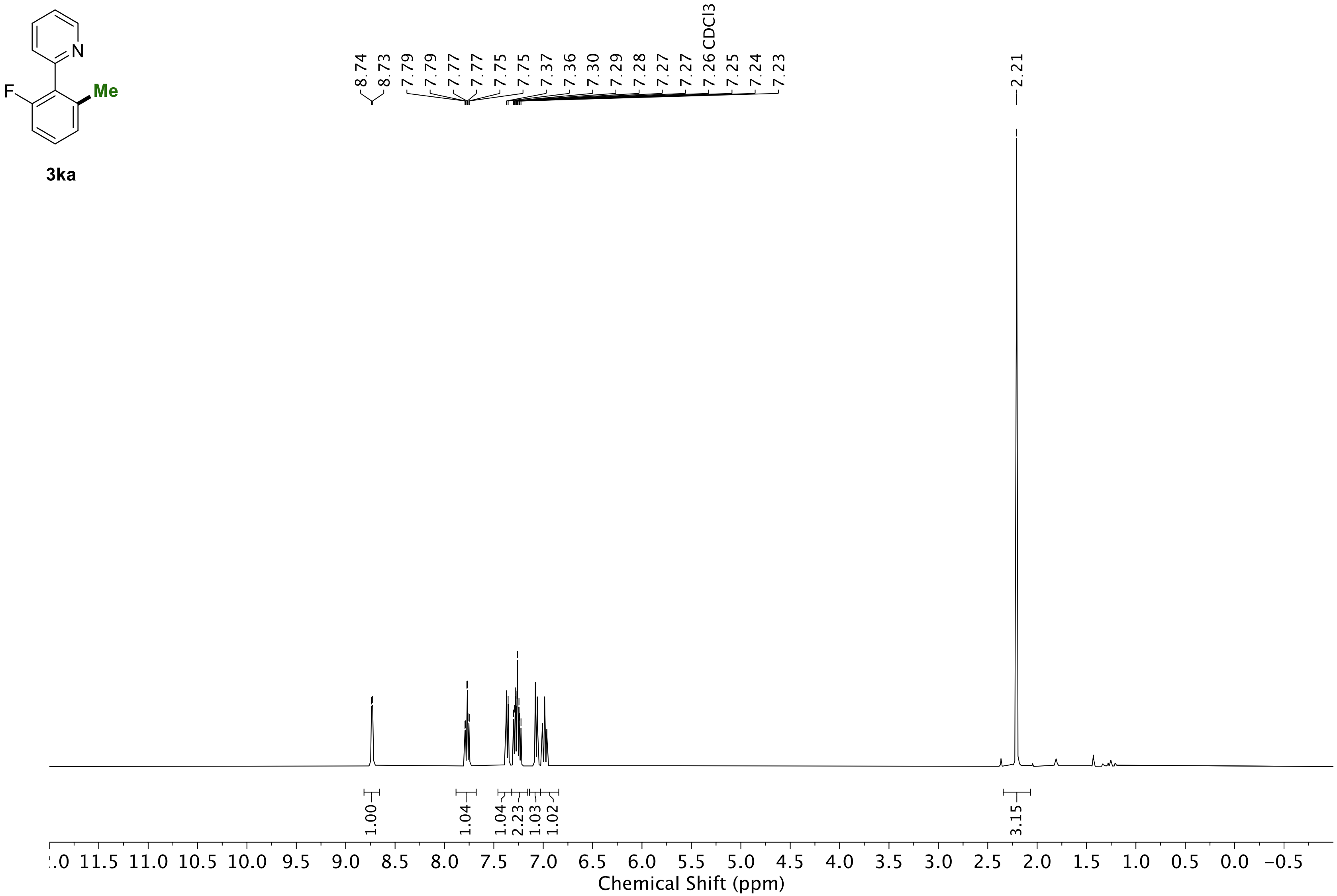

Figure S78. ^{13}C NMR (101 MHz, $CDCl_3$) of **3ka**.

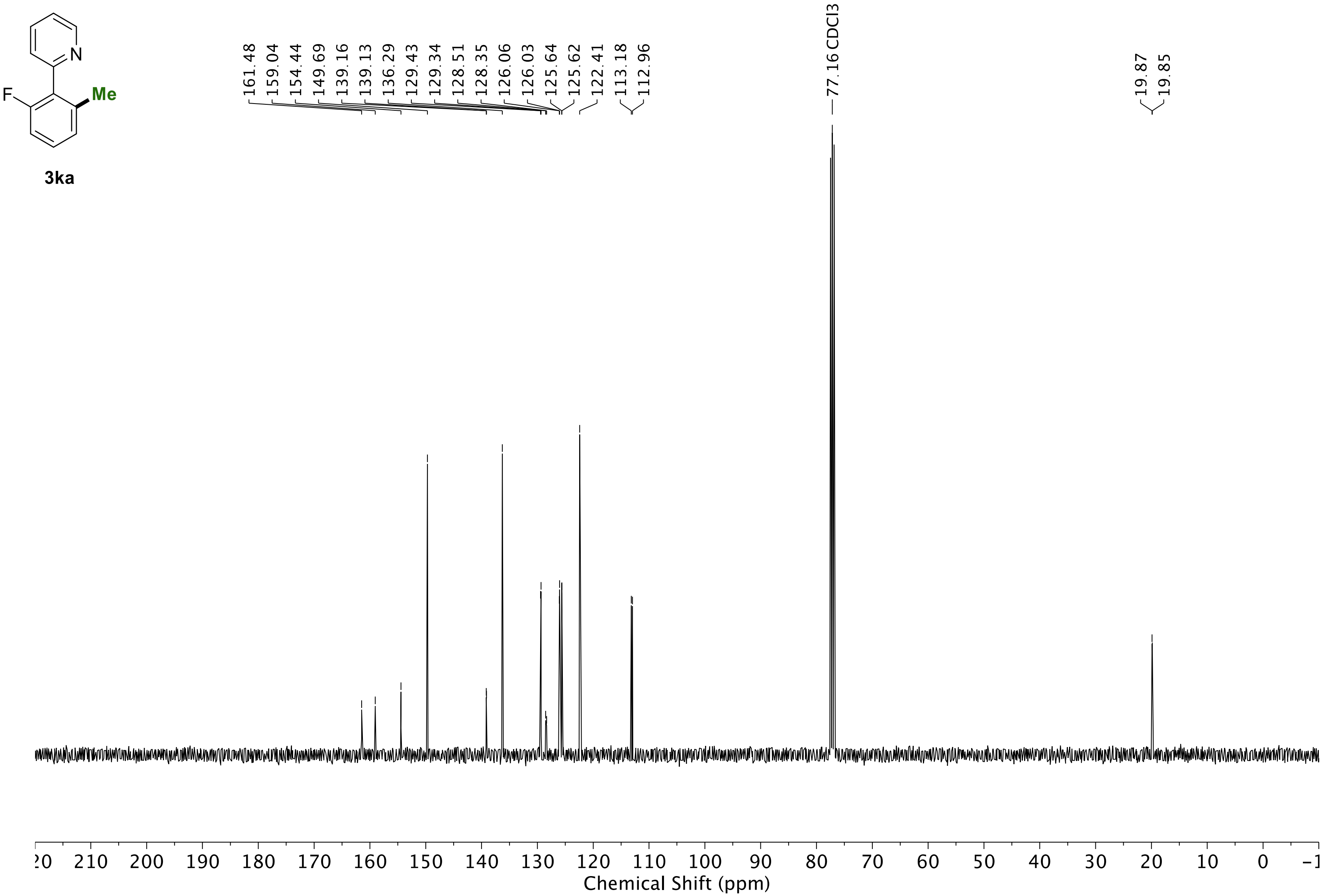

Figure S79. ^{19}F NMR (376 MHz, $CDCl_3$) of **3ka**.

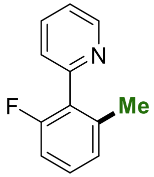

3ka

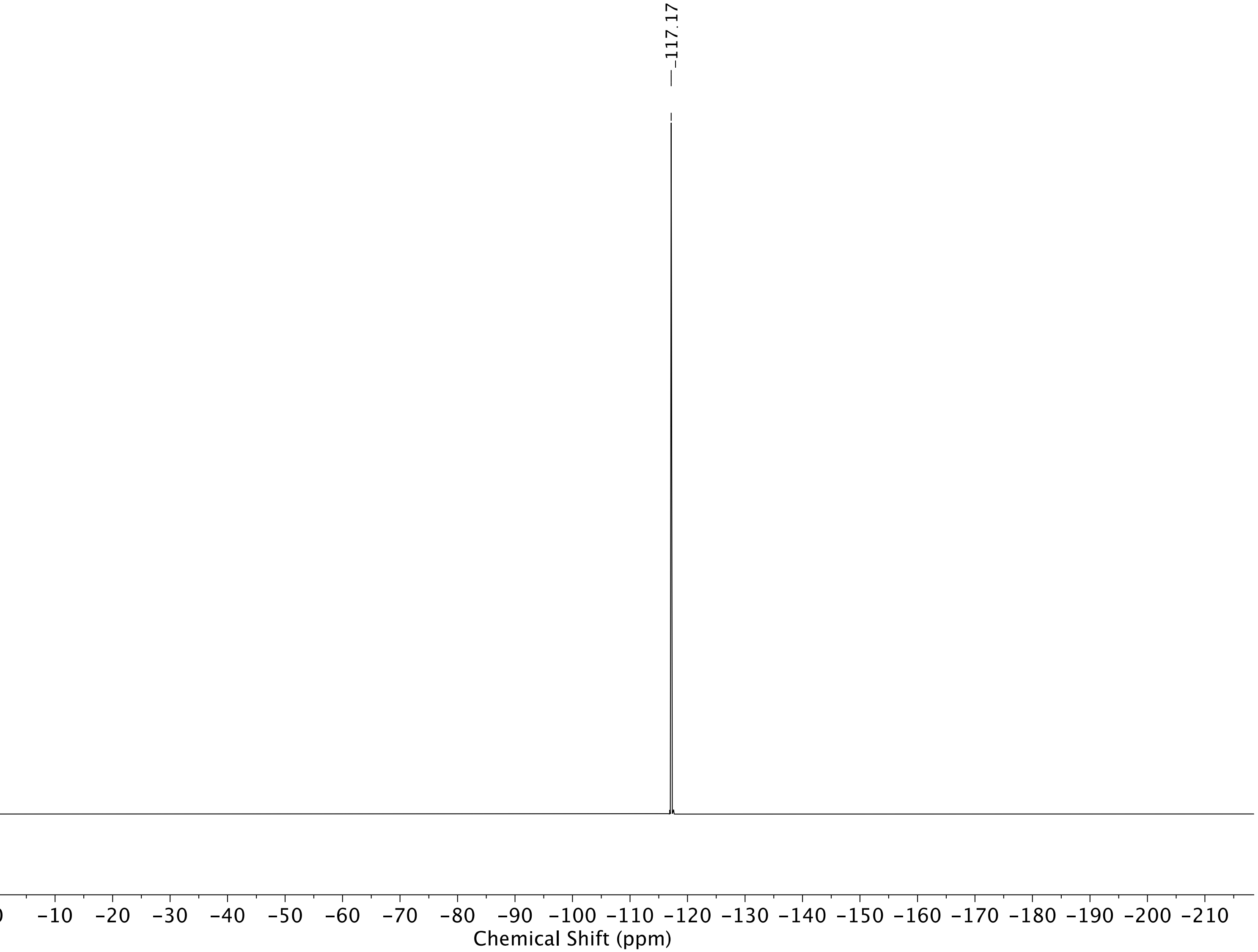

Figure S80. ^{1}H NMR (400 MHz, $CDCl_3$) of **3la**.

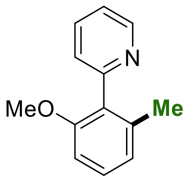

3la

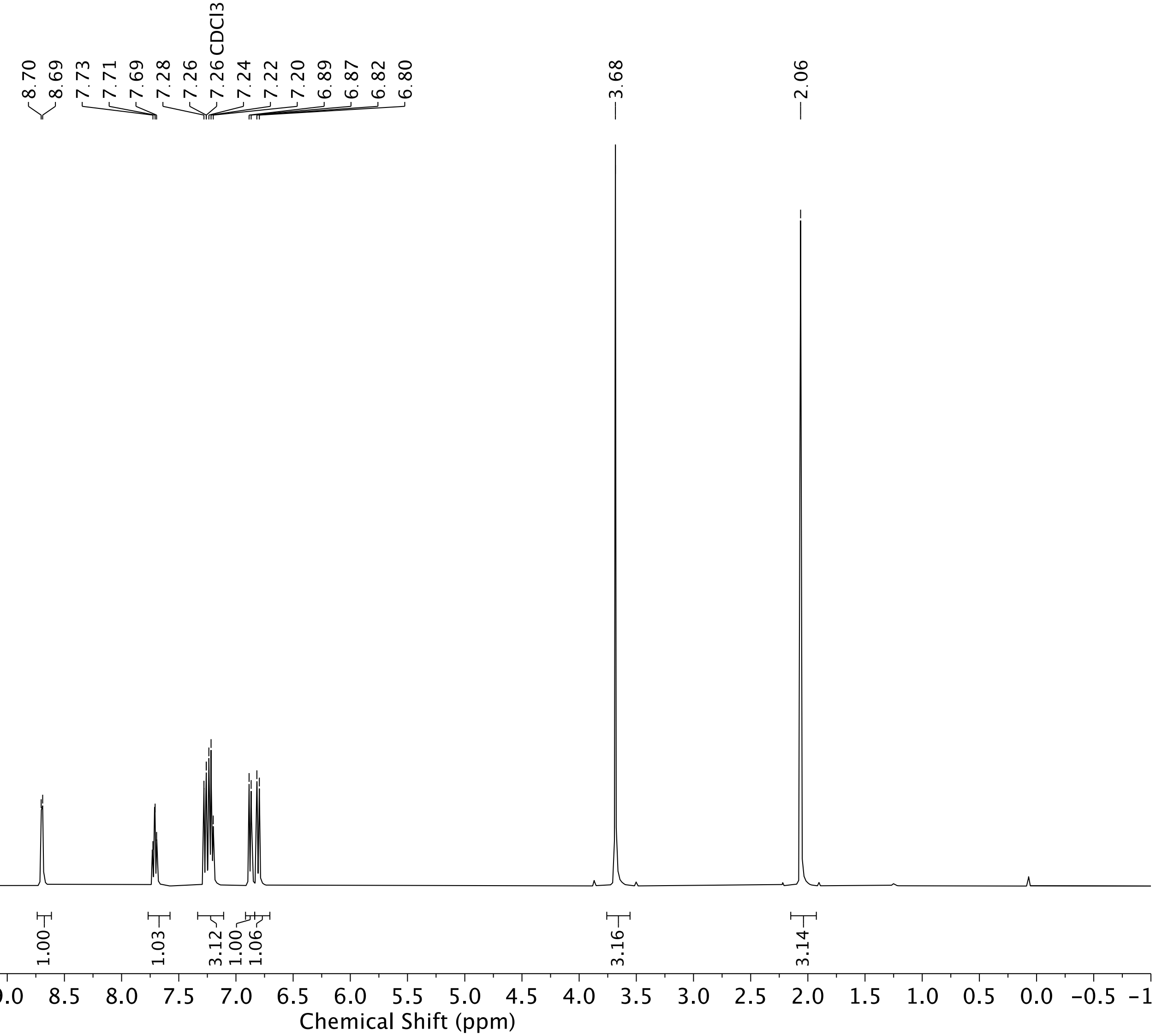

Figure S81. ^{13}C NMR (101 MHz, $CDCl_3$) of **3la**.

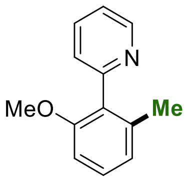

3la

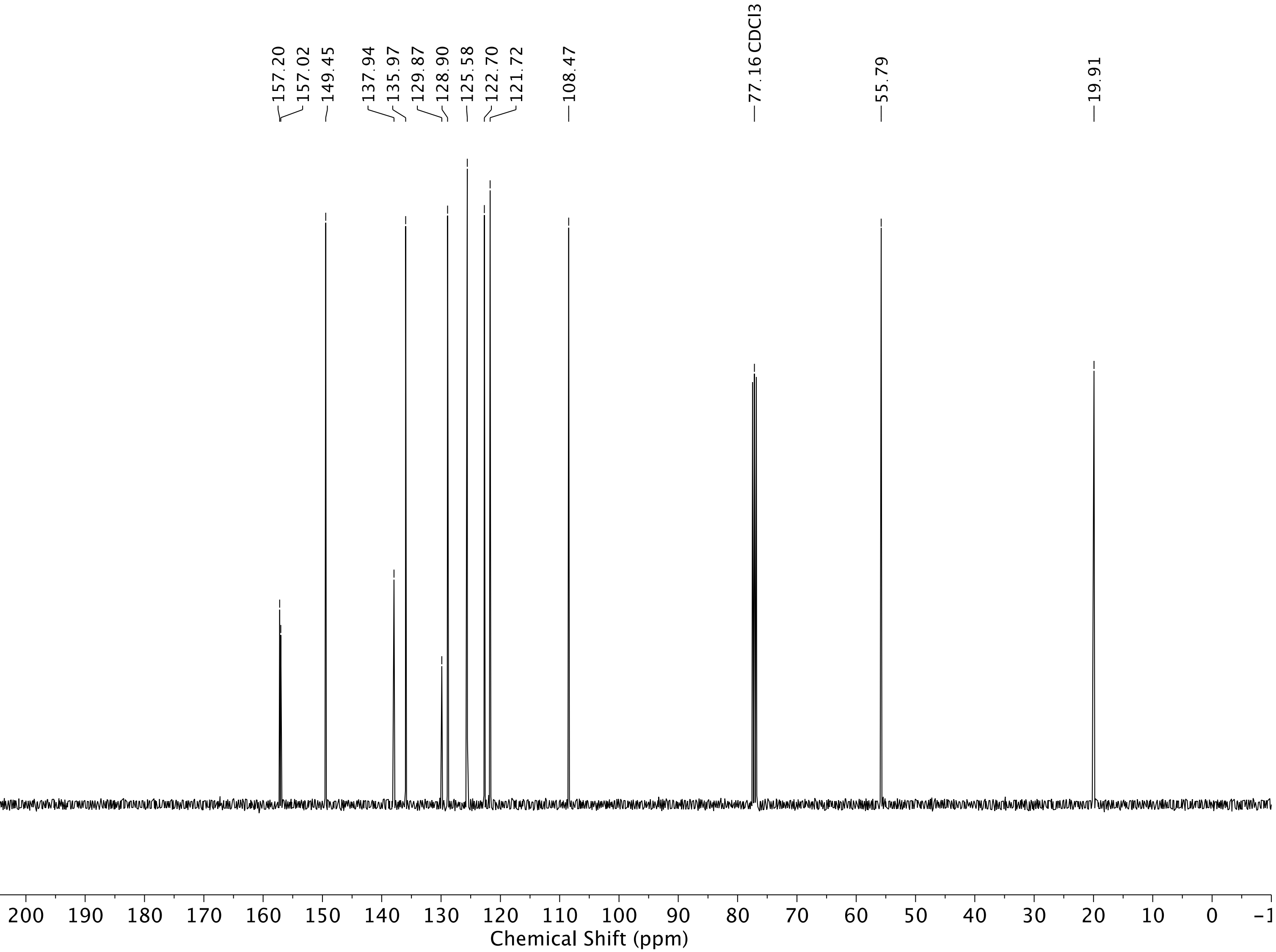

Figure S82. ^{1}H NMR (400 MHz, $CDCl_3$) of **3ma**.

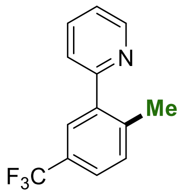

3ma

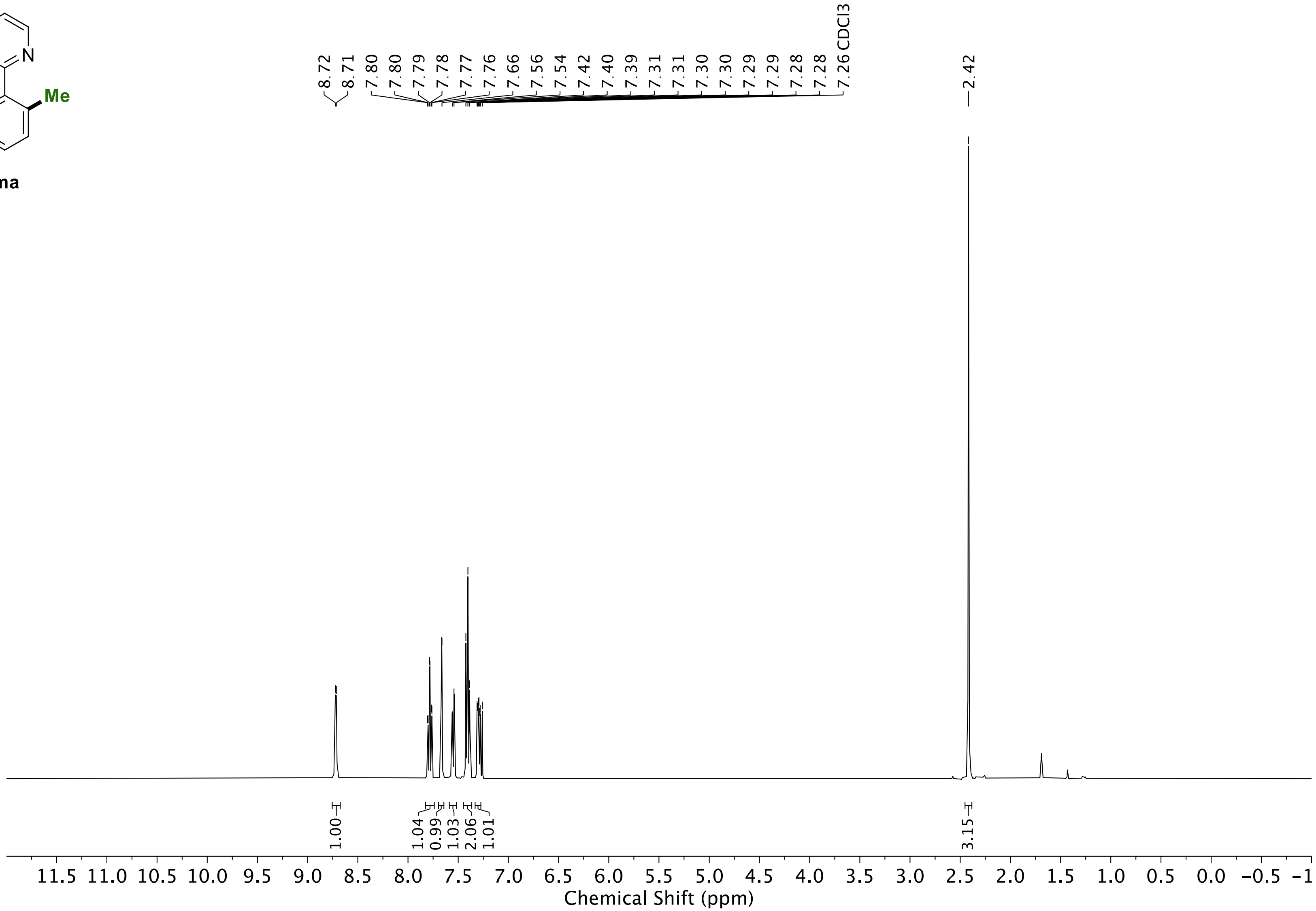

Figure S83. ^{13}C NMR (101 MHz, $CDCl_3$) of **3ma**.

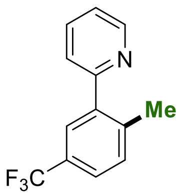

3ma

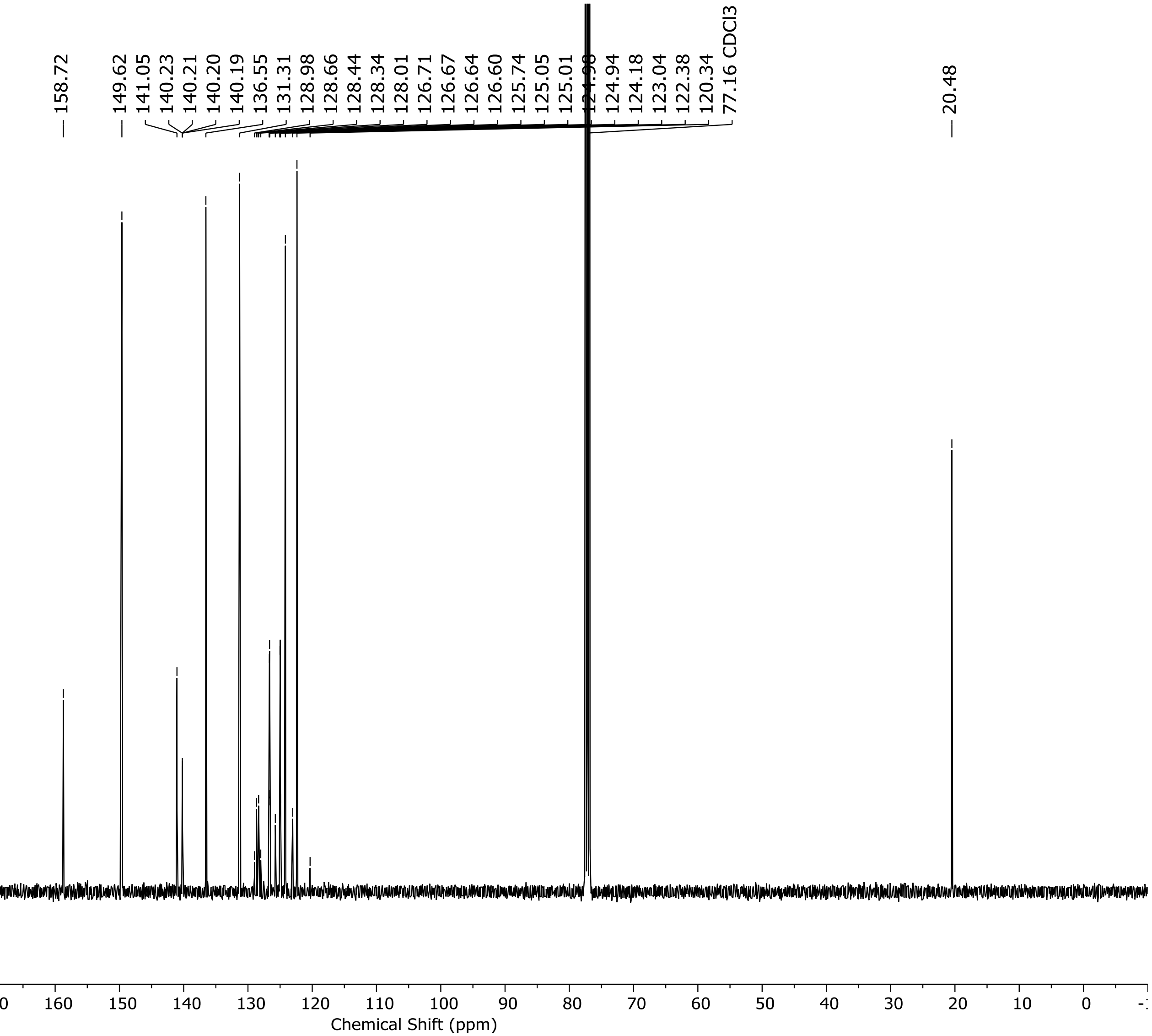

Figure S84. ^{19}F NMR (471 MHz, $CDCl_3$) of **3ma**.

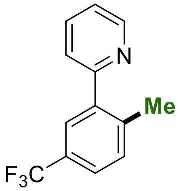

3ma

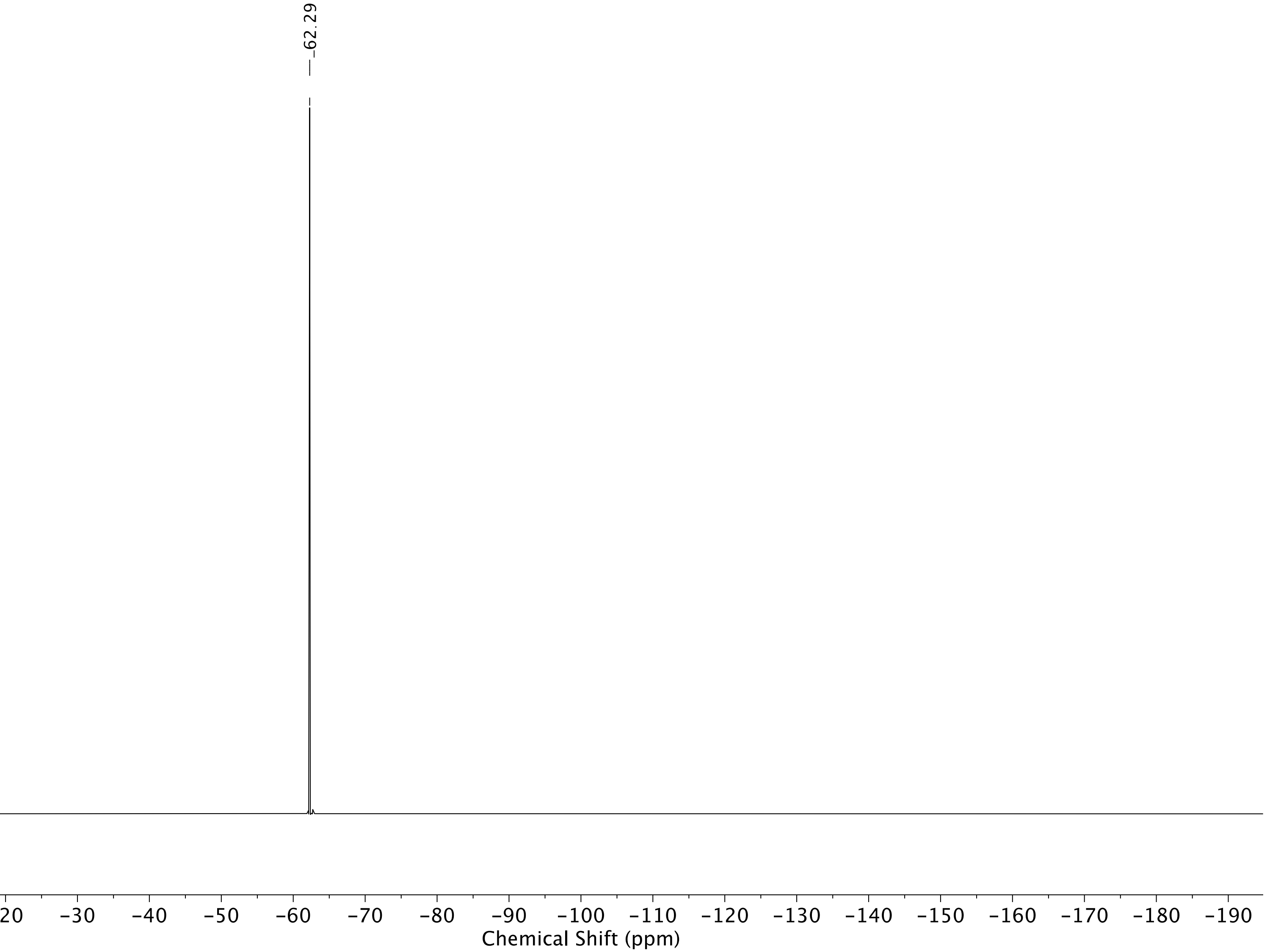

Figure S85. ^{1}H NMR (400 MHz, $CDCl_3$) of **3na**.

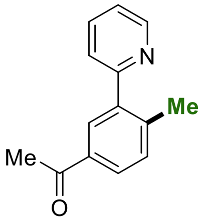

3na

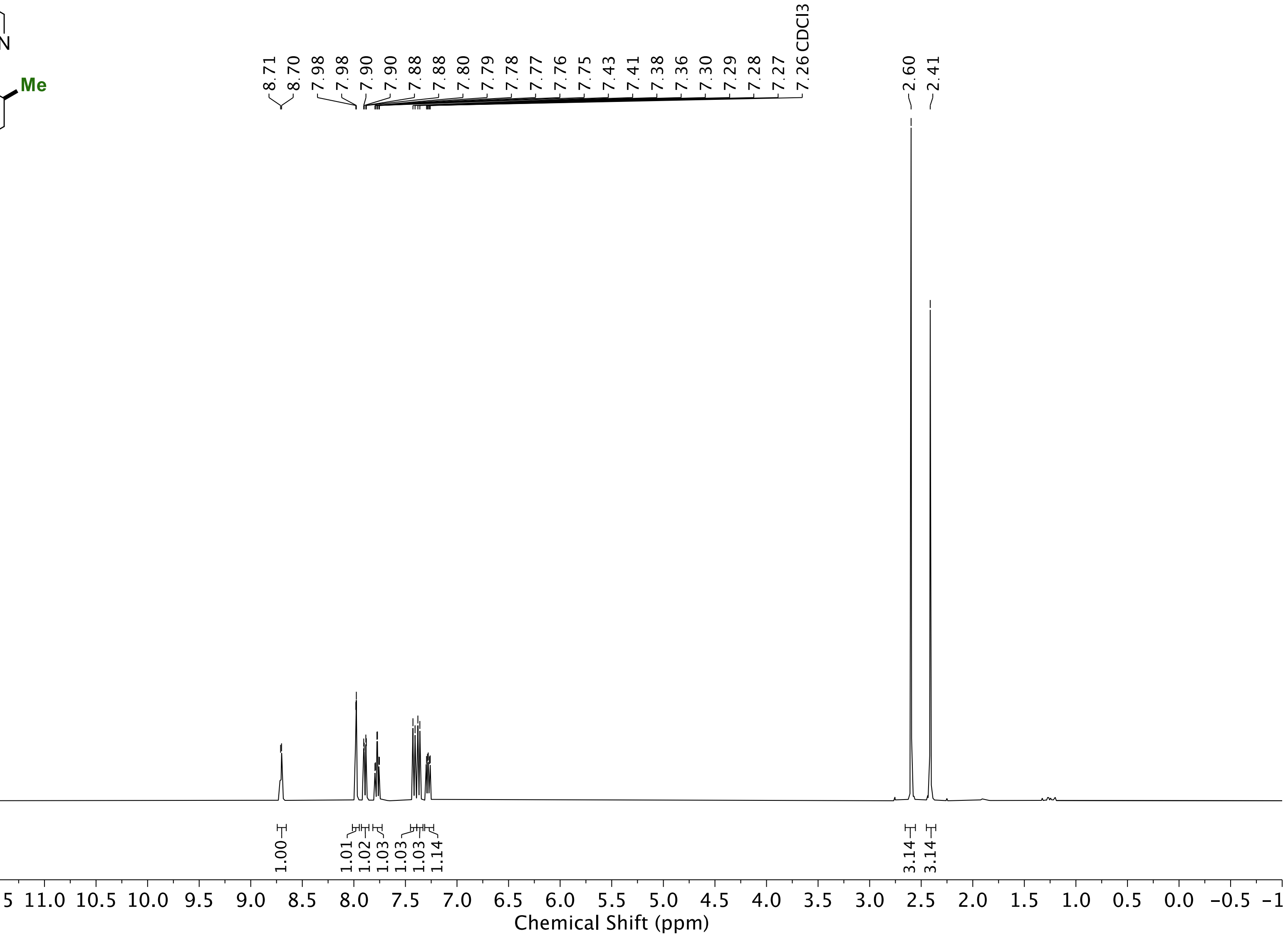

Figure S86. ^{13}C NMR (101 MHz, $CDCl_3$) of **3na**.

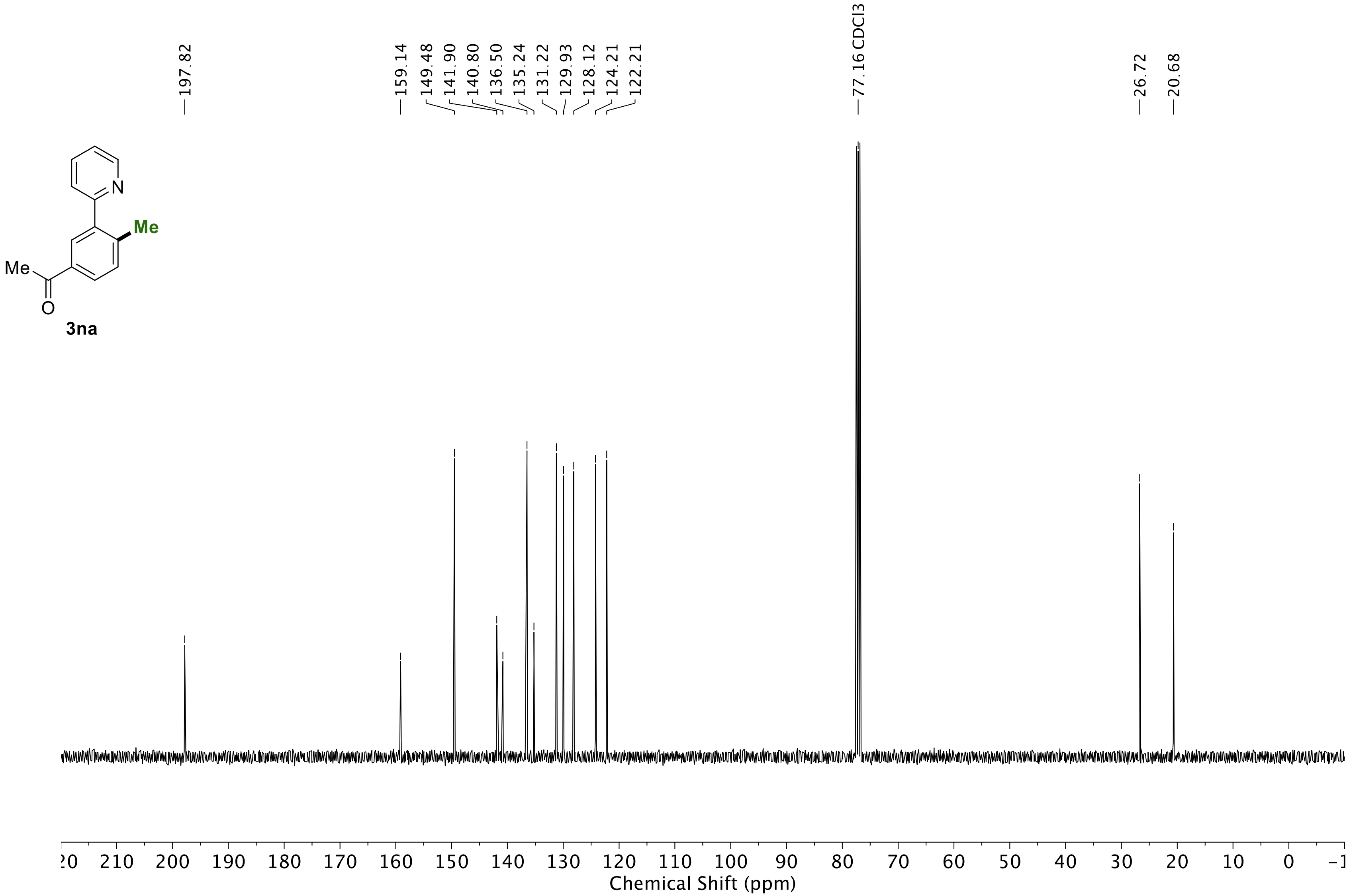

Figure S87. ^{1}H NMR (500 MHz, $CDCl_3$) of **3oa**.

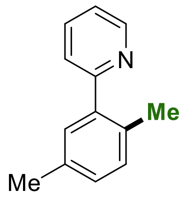

3oa

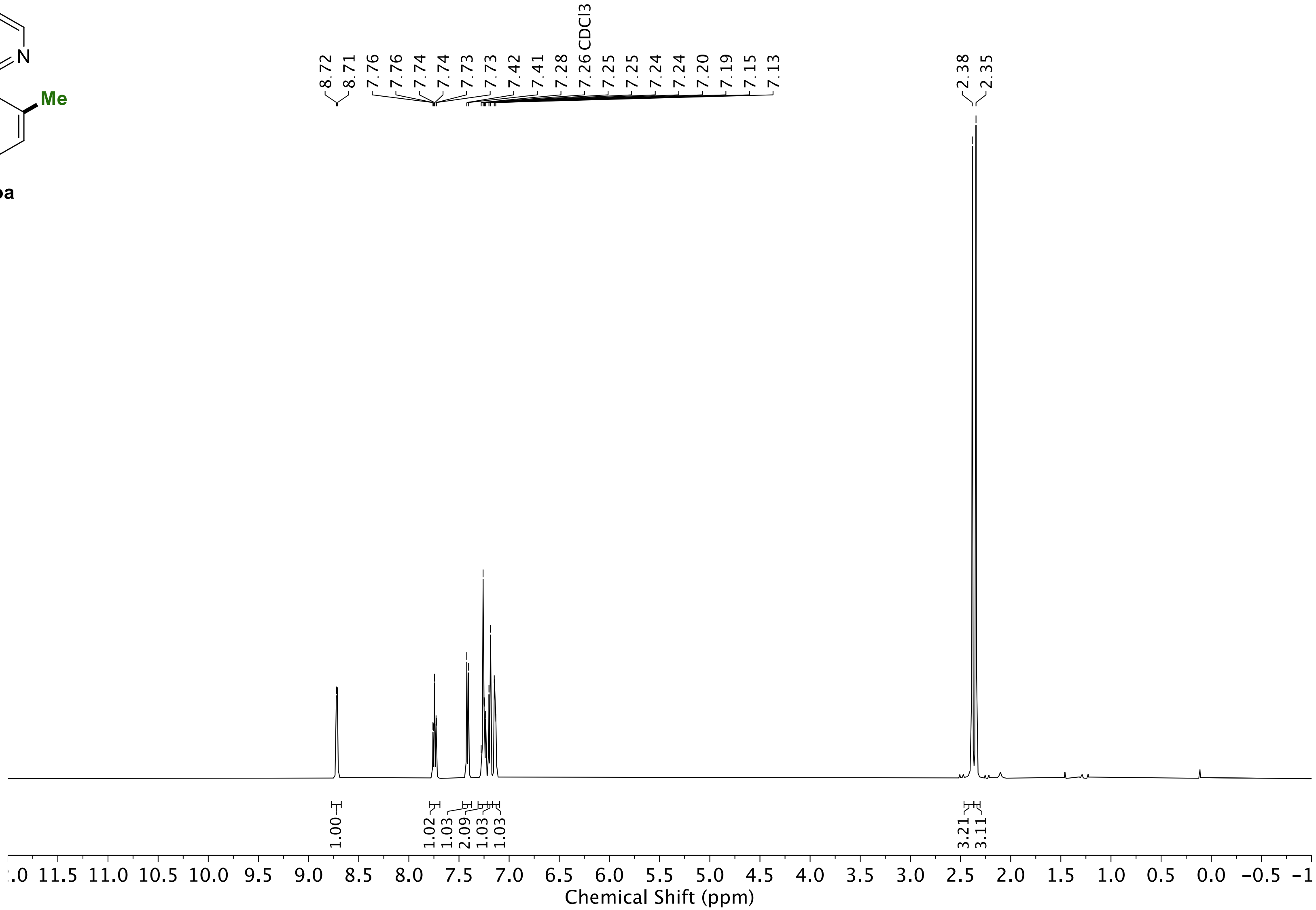

Figure S88. ^{13}C NMR (126 MHz, $CDCl_3$) of **3oa**.

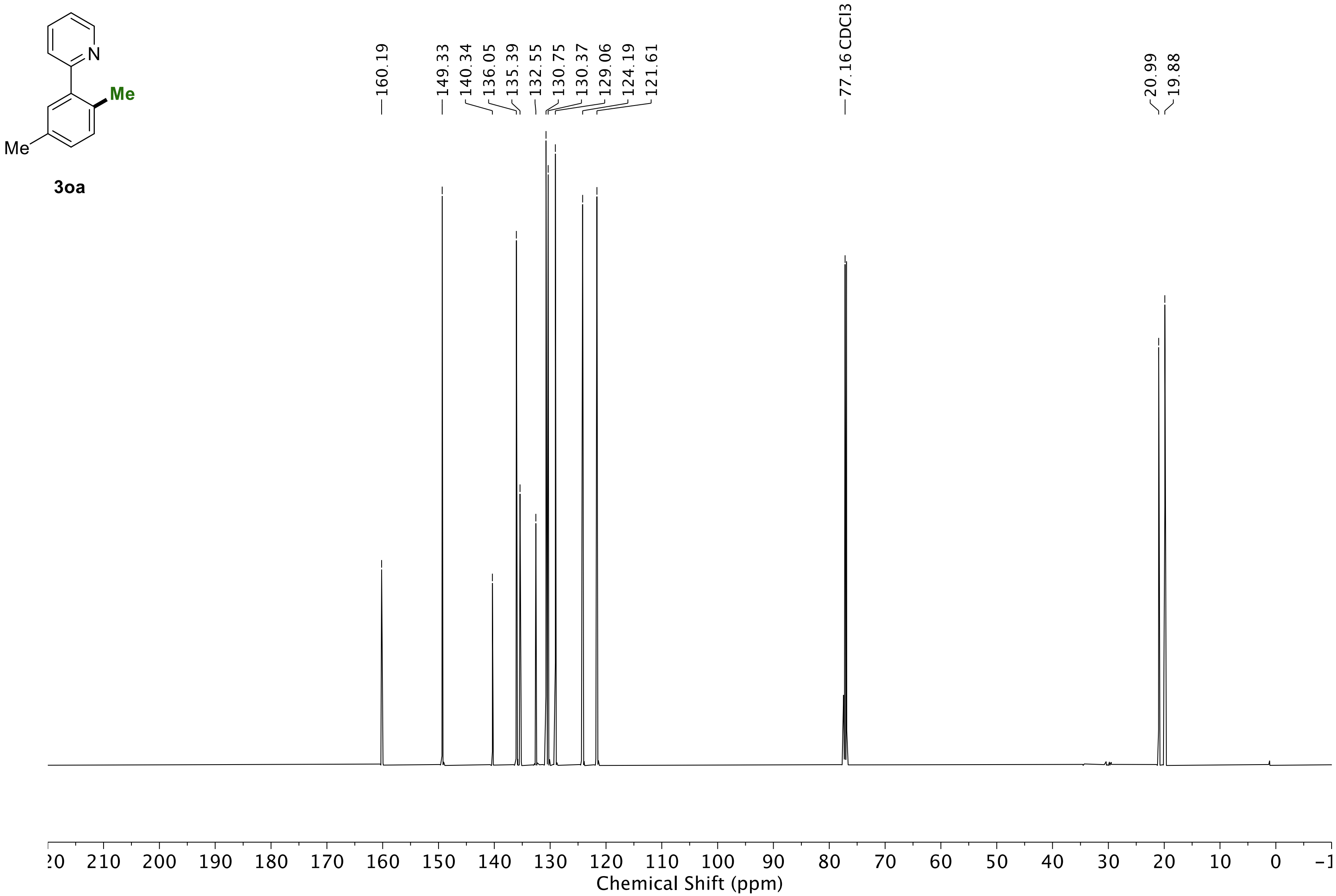

Figure S89. ^{1}H NMR (500 MHz, $CDCl_3$) of **3pa**.

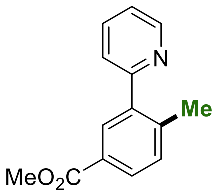

3pa

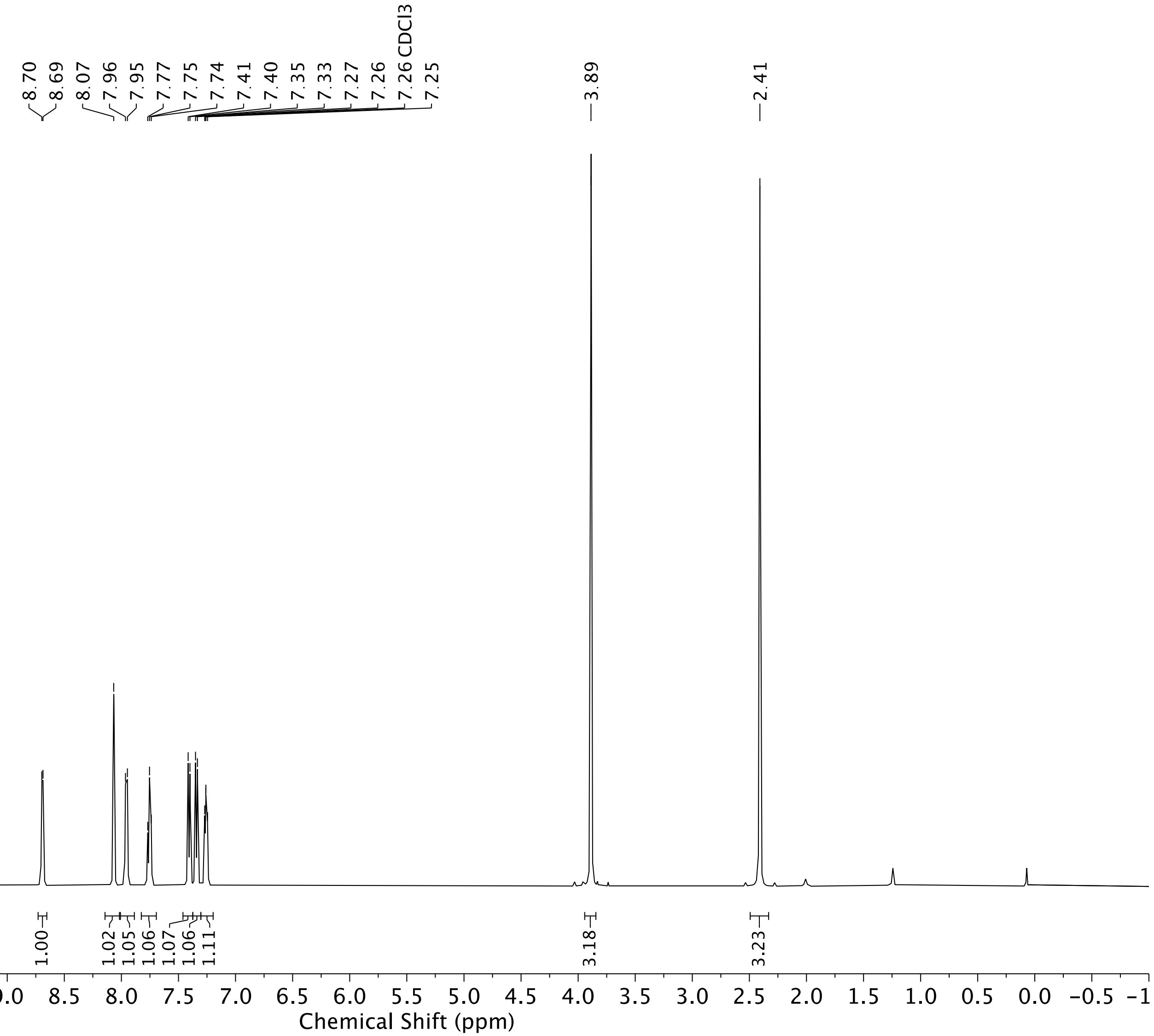

Figure S90. ^{13}C NMR (101 MHz, $CDCl_3$) of **3pa**.

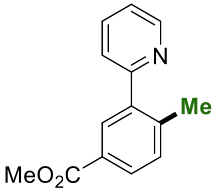

3pa

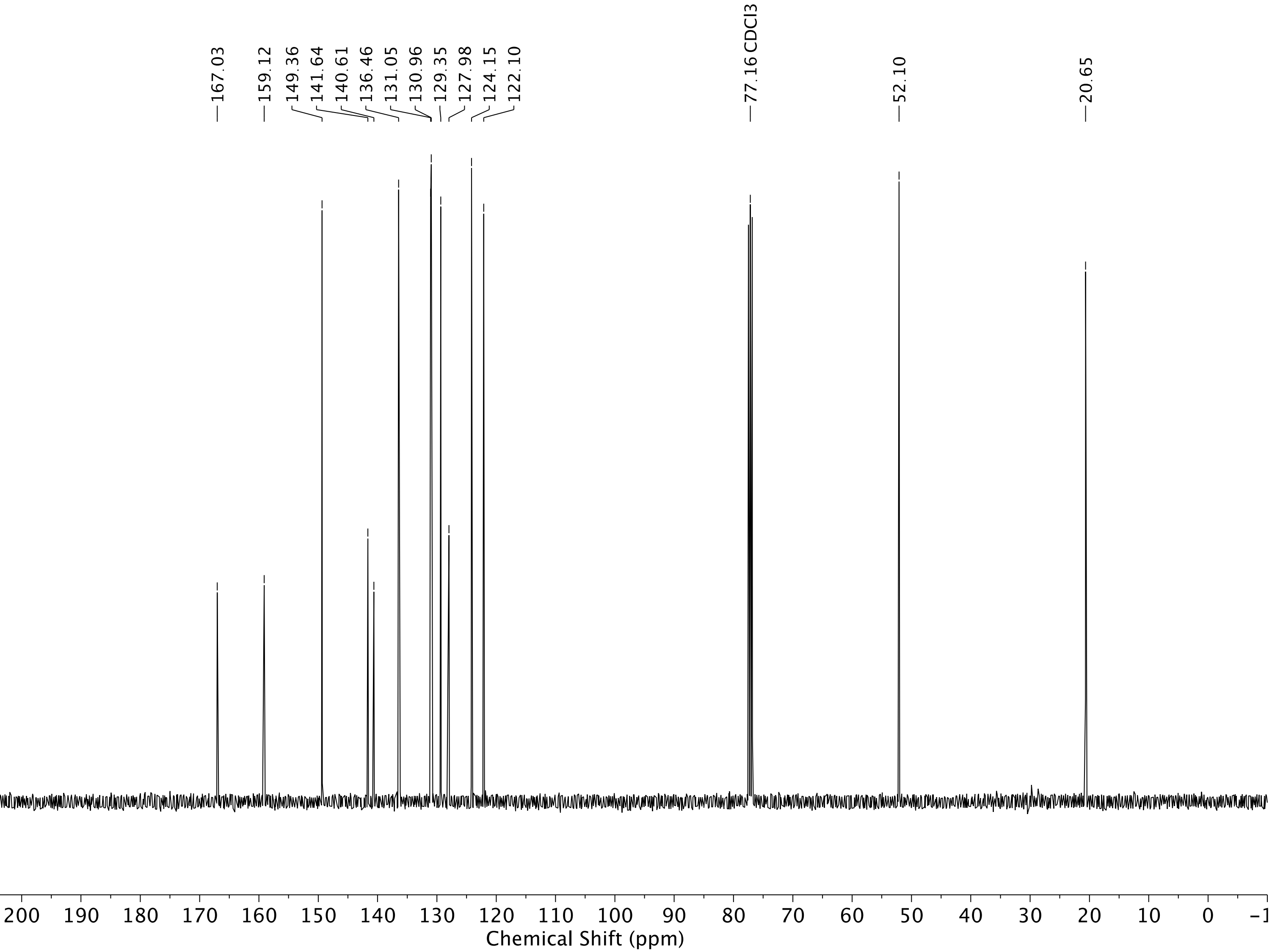

Figure S91. ^{1}H NMR (400 MHz, $CDCl_3$) of **3qa**.

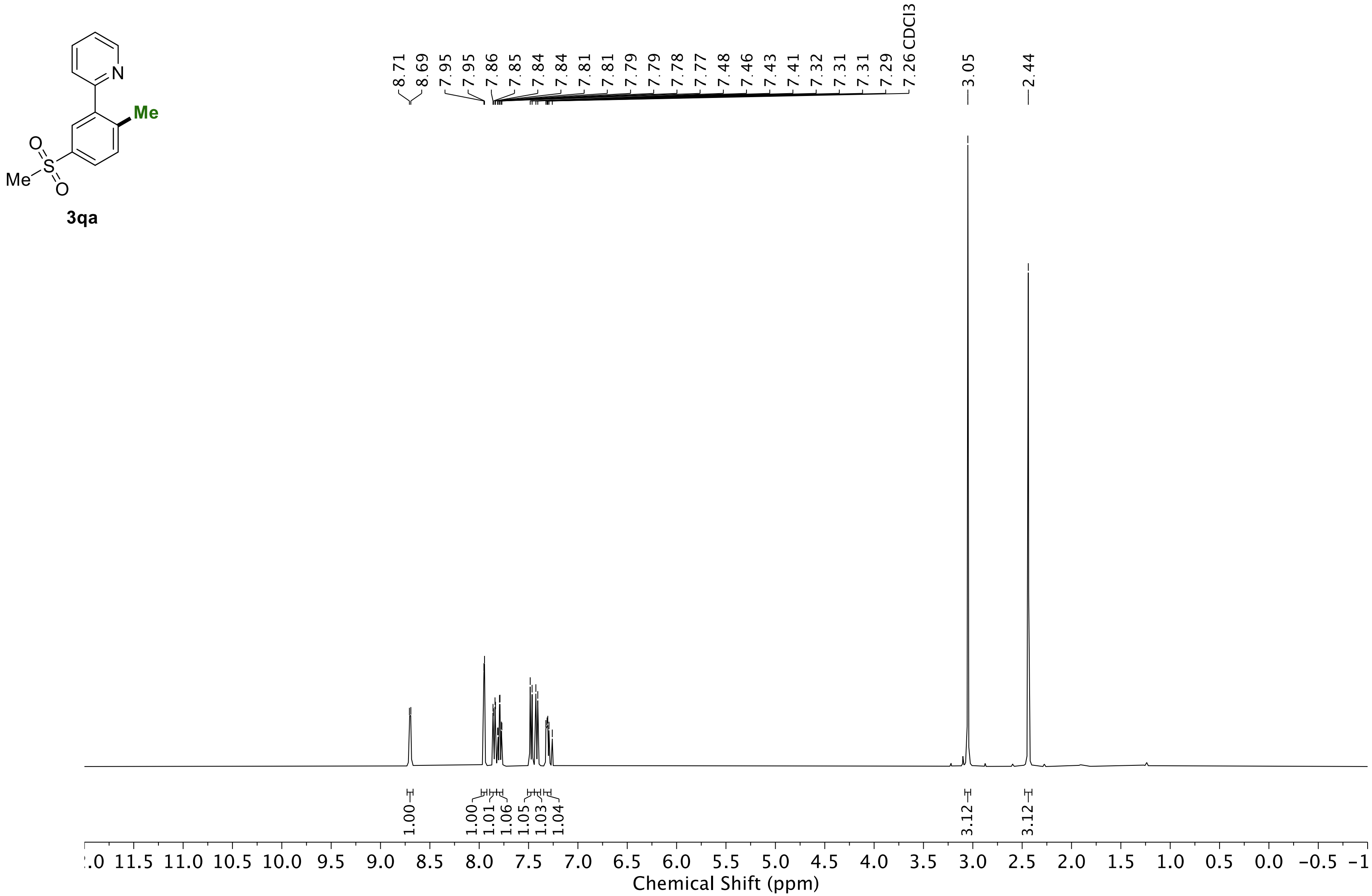

Figure S92. ^{14}C NMR (101 MHz, $CDCl_3$) of **3qa**.

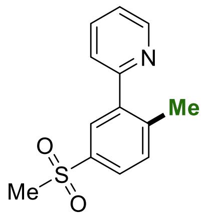

3qa

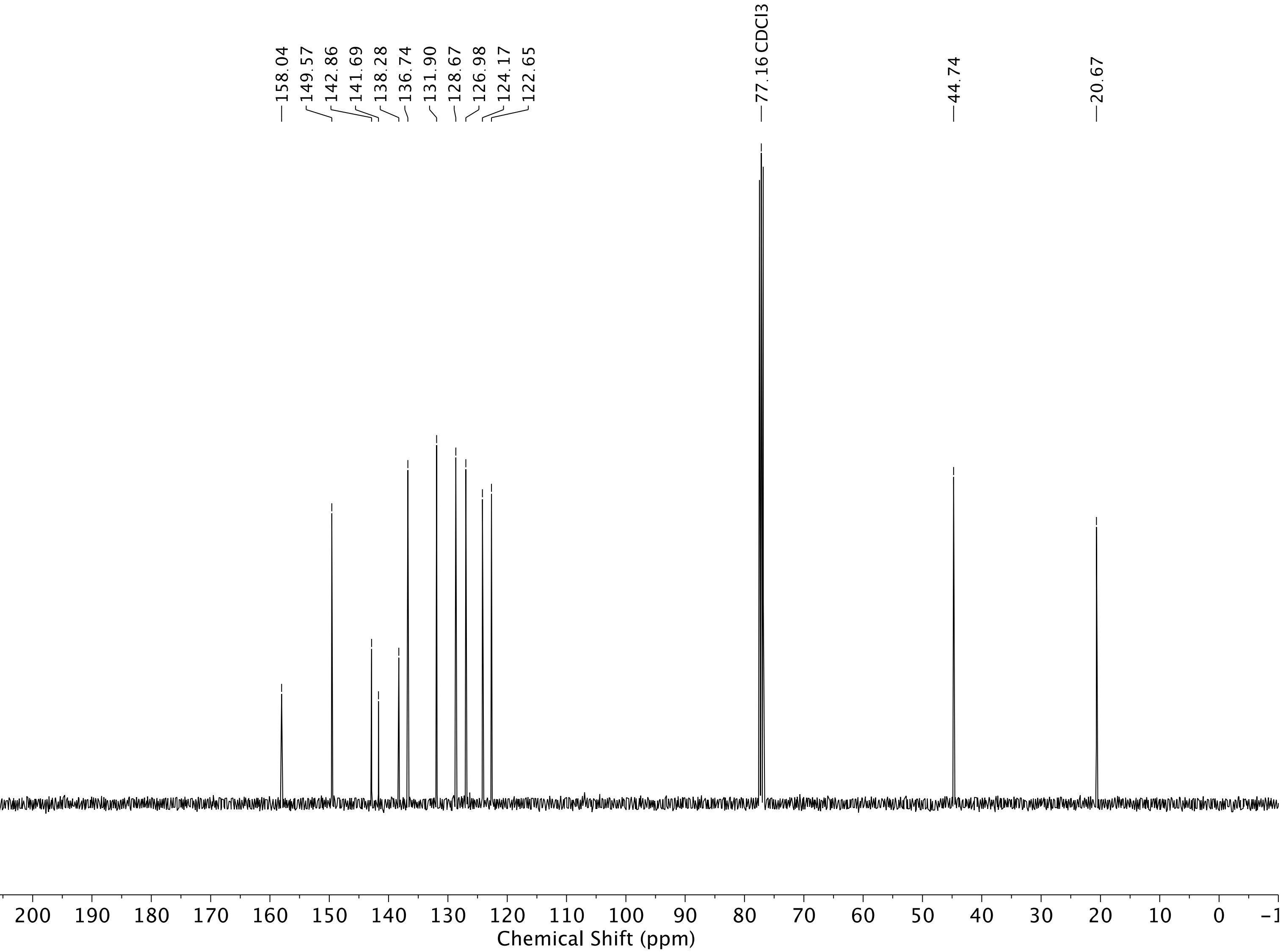

Figure S93. ^{1}H NMR (400 MHz, CD_2Cl_2) of **3ra**.

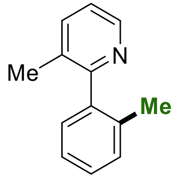

3ra

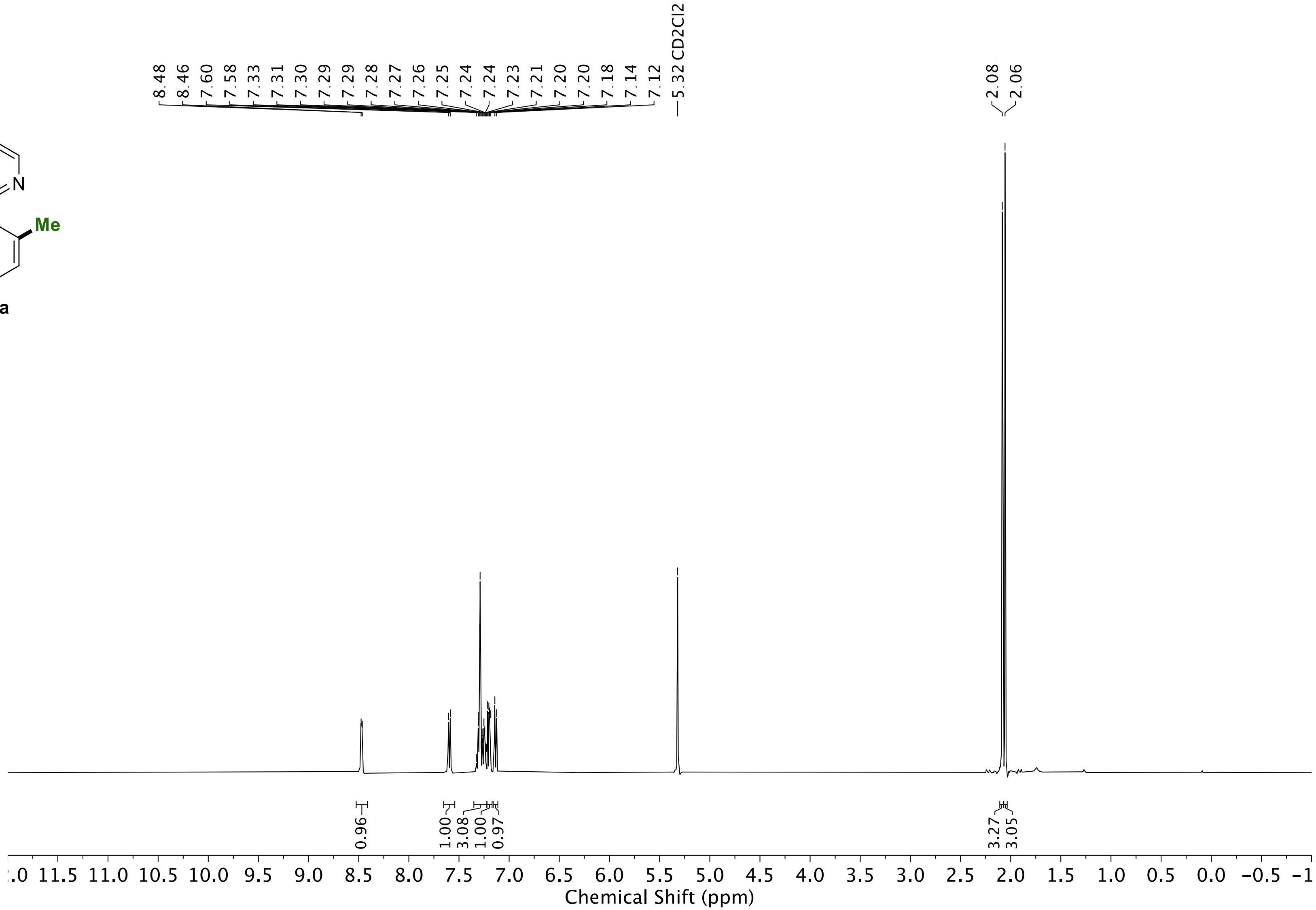

Figure S94. ^{13}C NMR (101 MHz, CD_2Cl_2) of **3ra**.

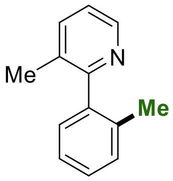

3ra

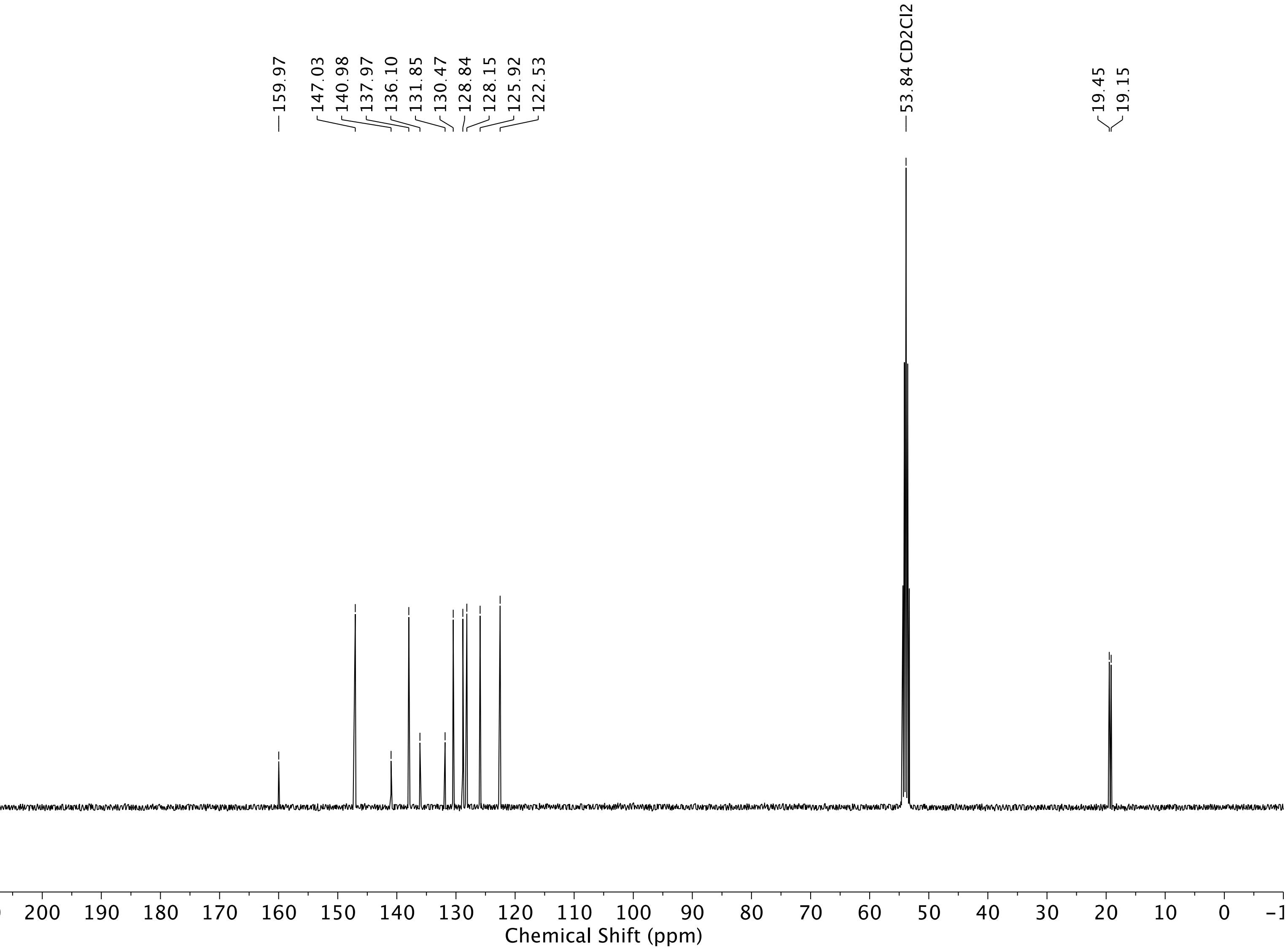

Figure S95. ^{1}H NMR (400 MHz, $CDCl_3$) of ***d₄*-3ra**.

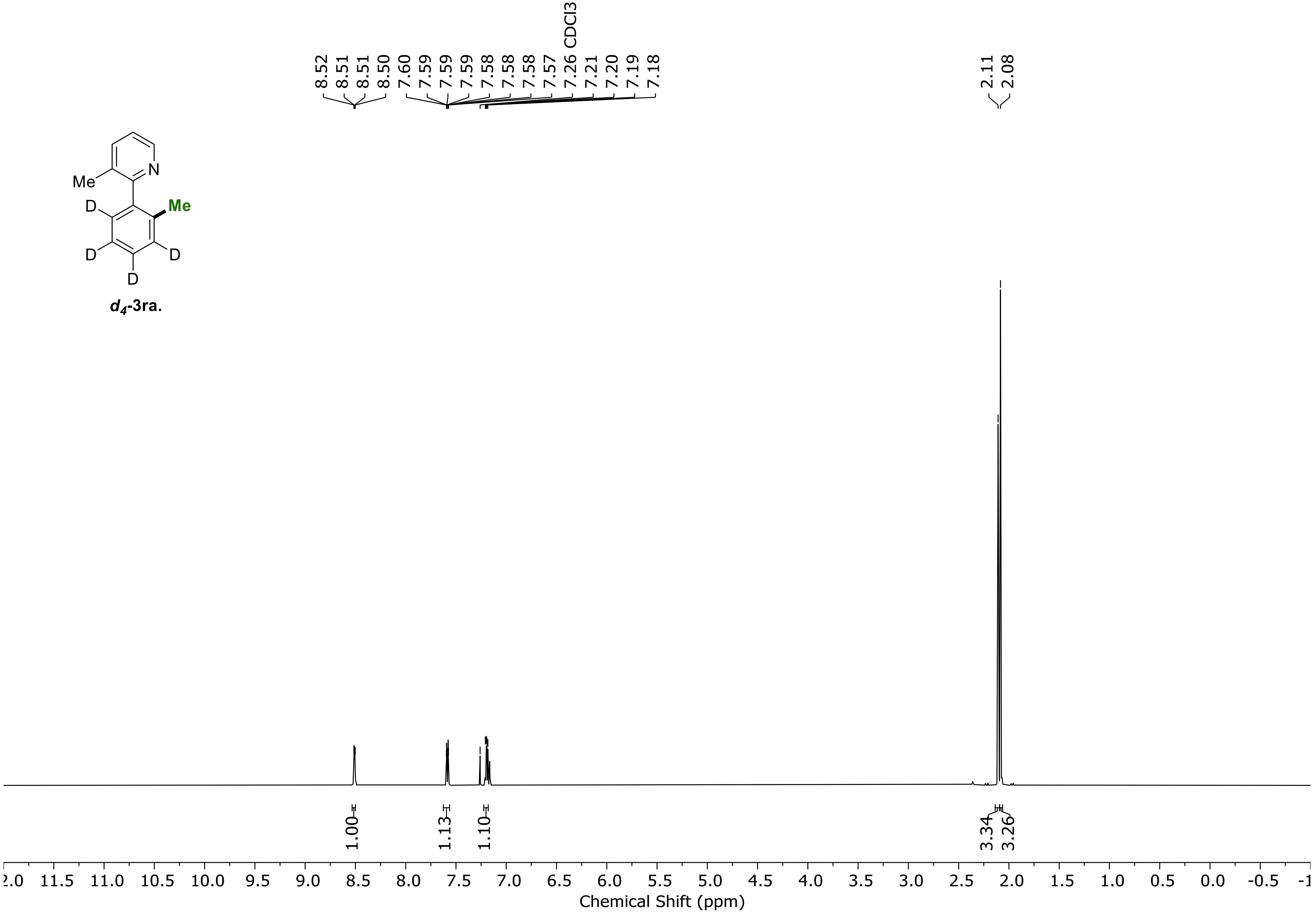

Figure S96. ^{13}C NMR (101 MHz, $CDCl_3$) of ***d$_4$*-3ra**.

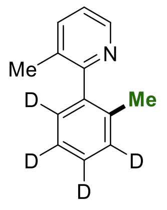

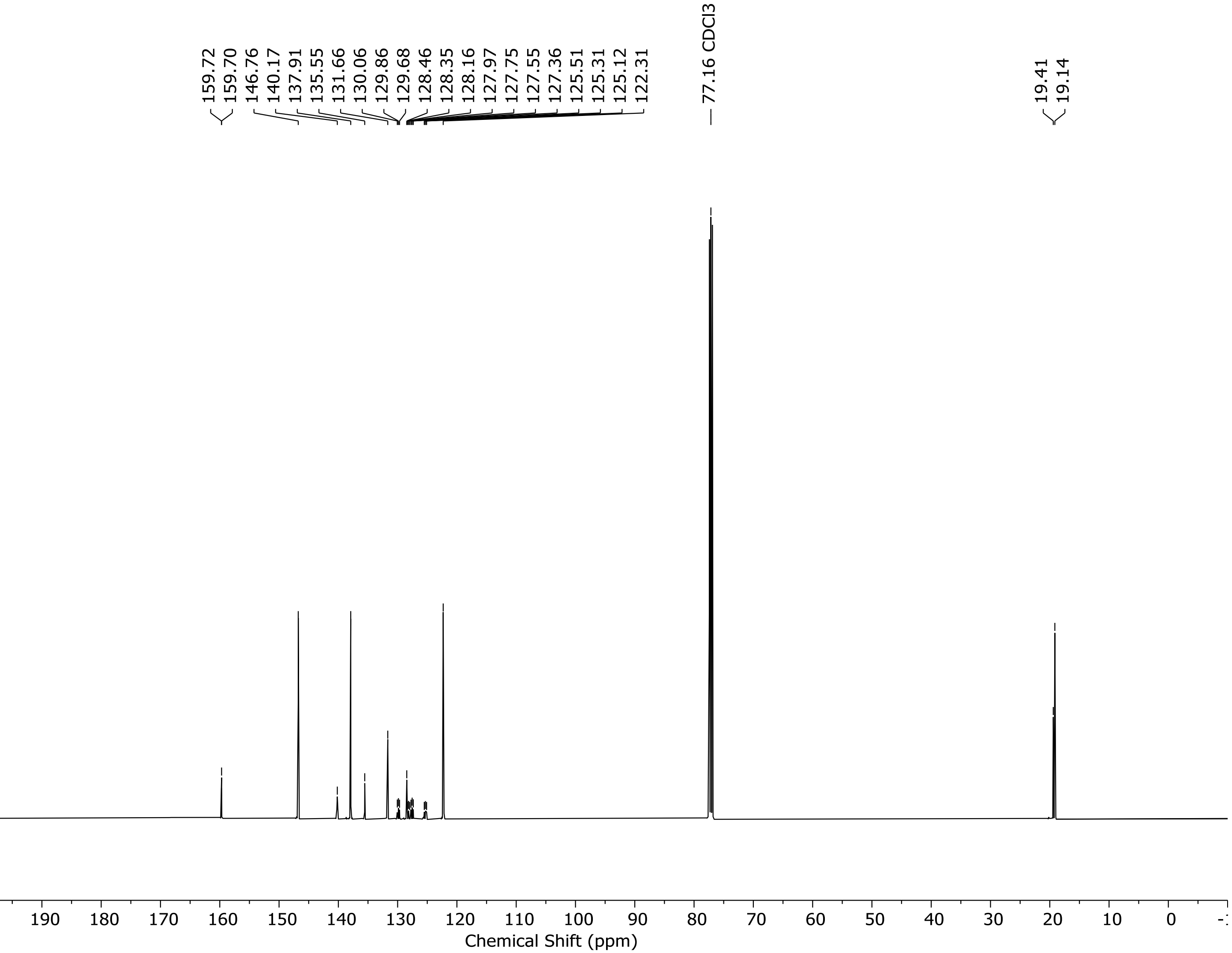

Figure S97. ^{1}H NMR (400 MHz, $CDCl_3$) of **3sa**.

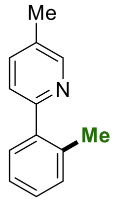

3sa

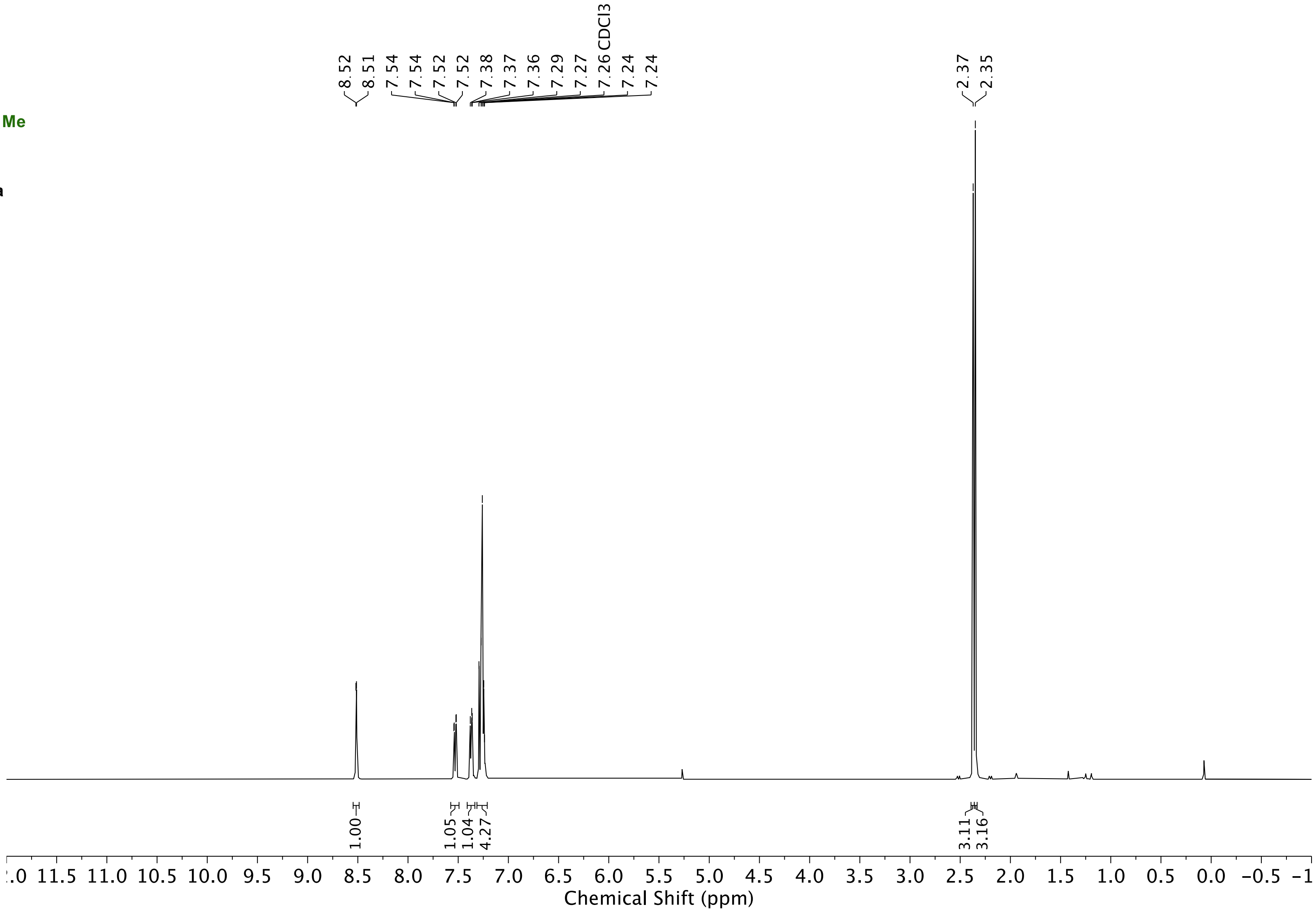

Figure S98. ^{13}C NMR (126 MHz, $CDCl_3$) of **3sa**.

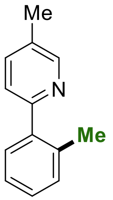

3sa

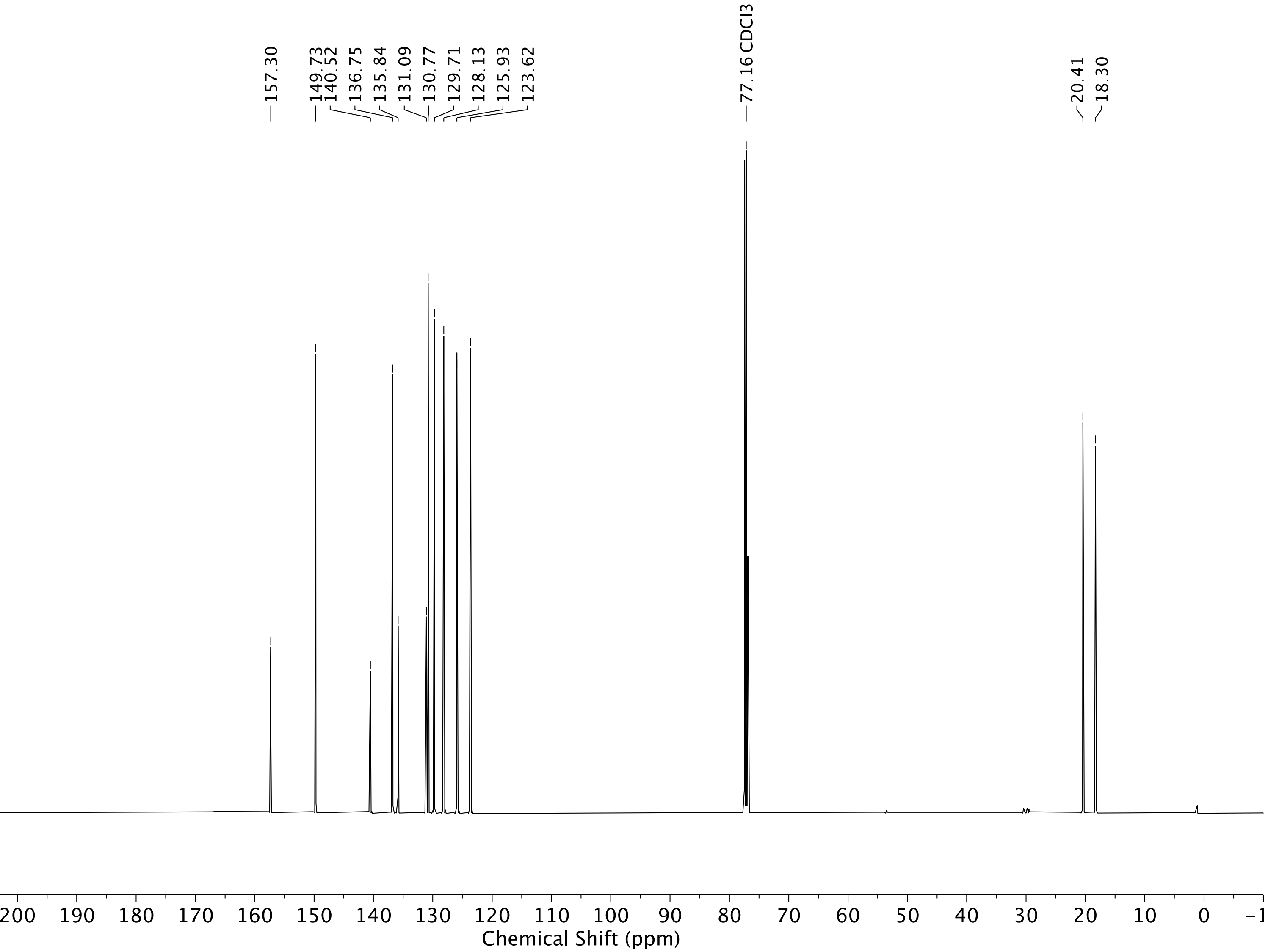

Figure S99. ^{1}H NMR (400 MHz, $CDCl_3$) of **4sa**.

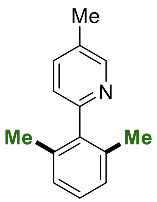

4sa

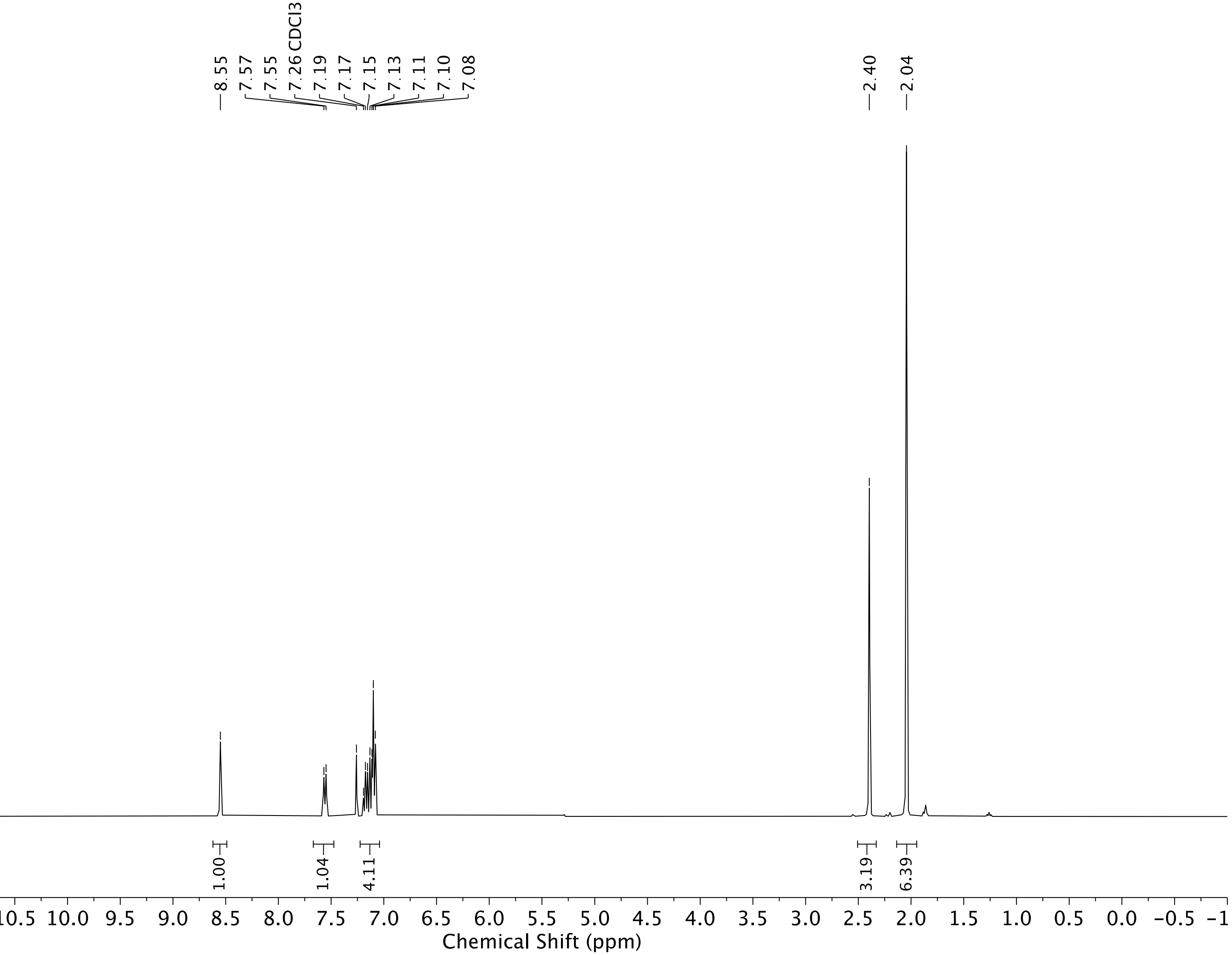

Figure S100. ^{13}C NMR (101 MHz, $CDCl_3$) of **4sa.**

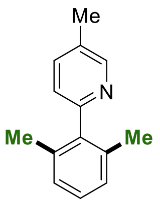

4sa

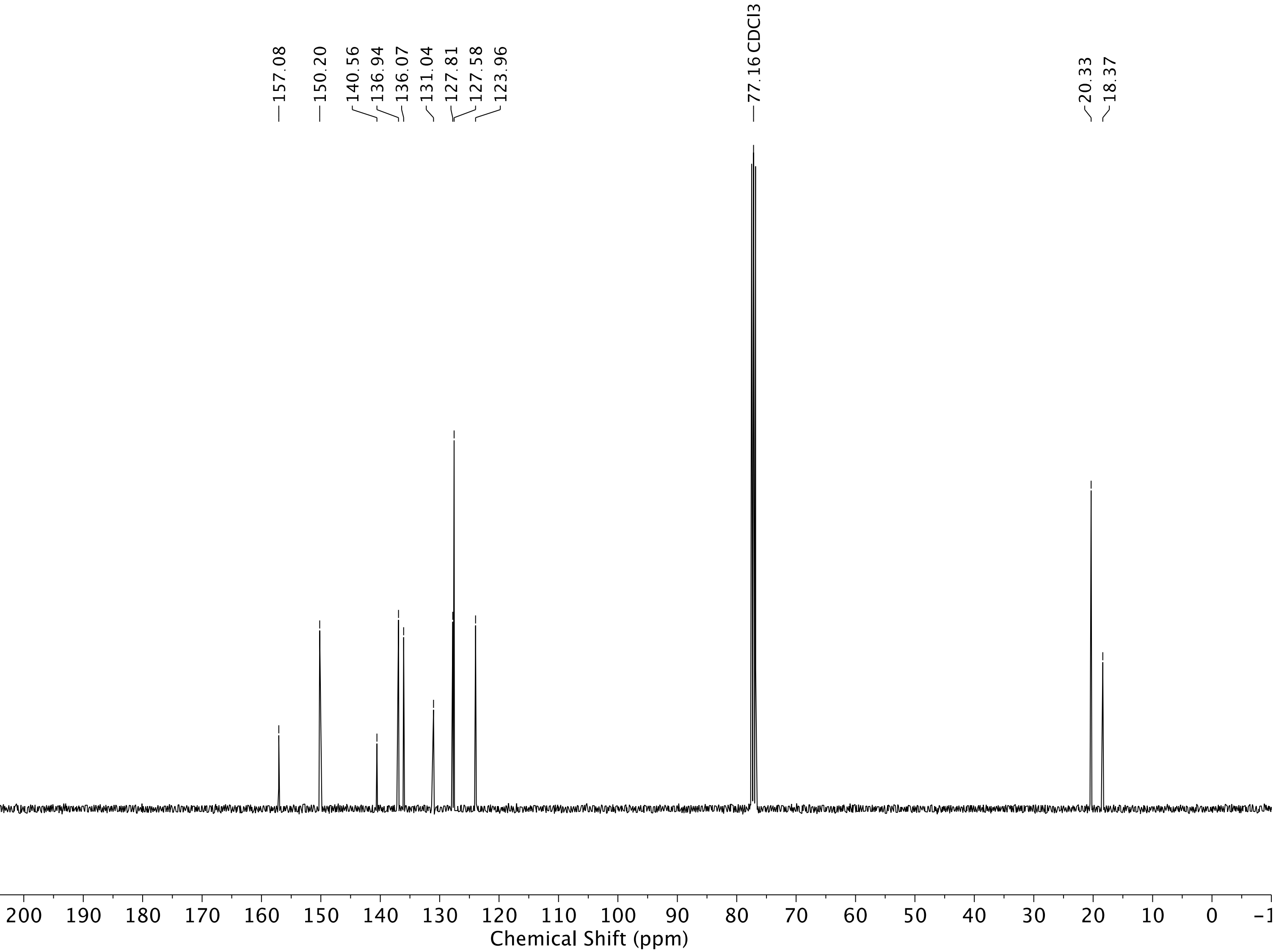

Figure S101. ^{1}H NMR (400 MHz, $CDCl_3$) of **3ta**.

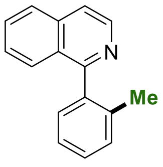

3ta

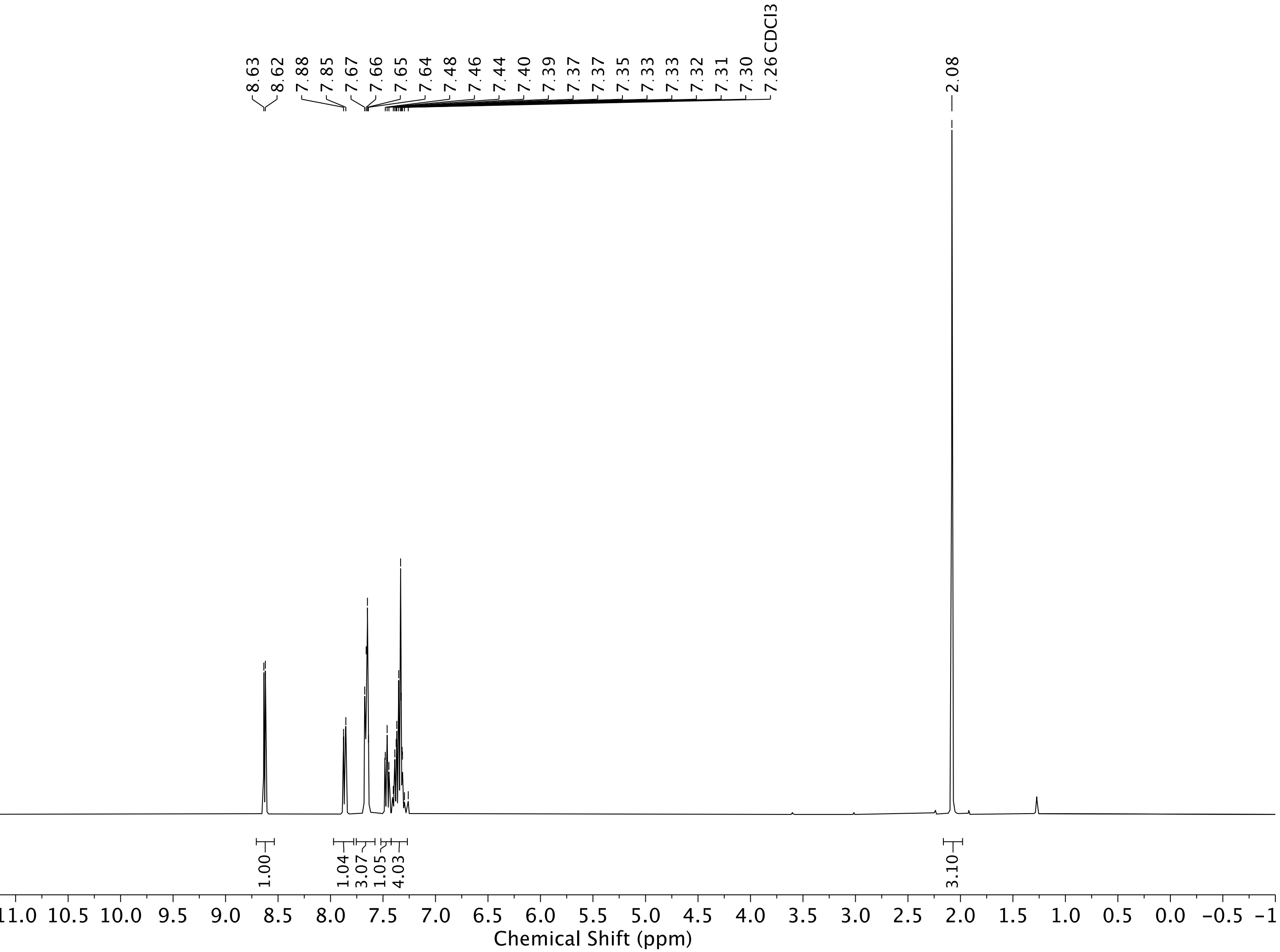

Figure S102. ^{13}C NMR (101 MHz, $CDCl_3$) of **3ta**.

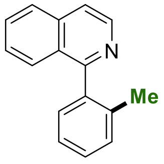

3ta

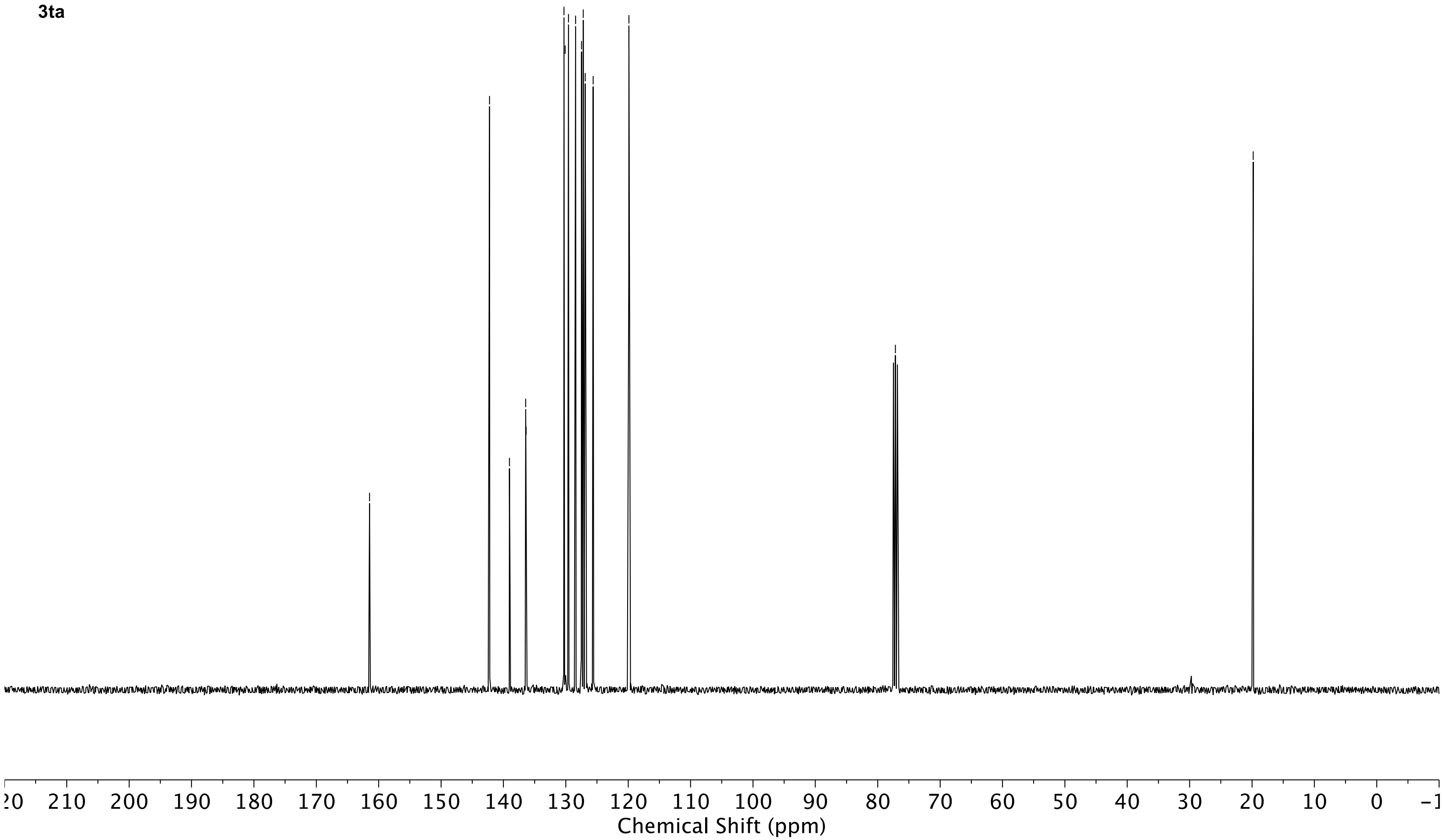

Figure S103. ^{1}H NMR (400 MHz, $CDCl_3$) of **3ua**.

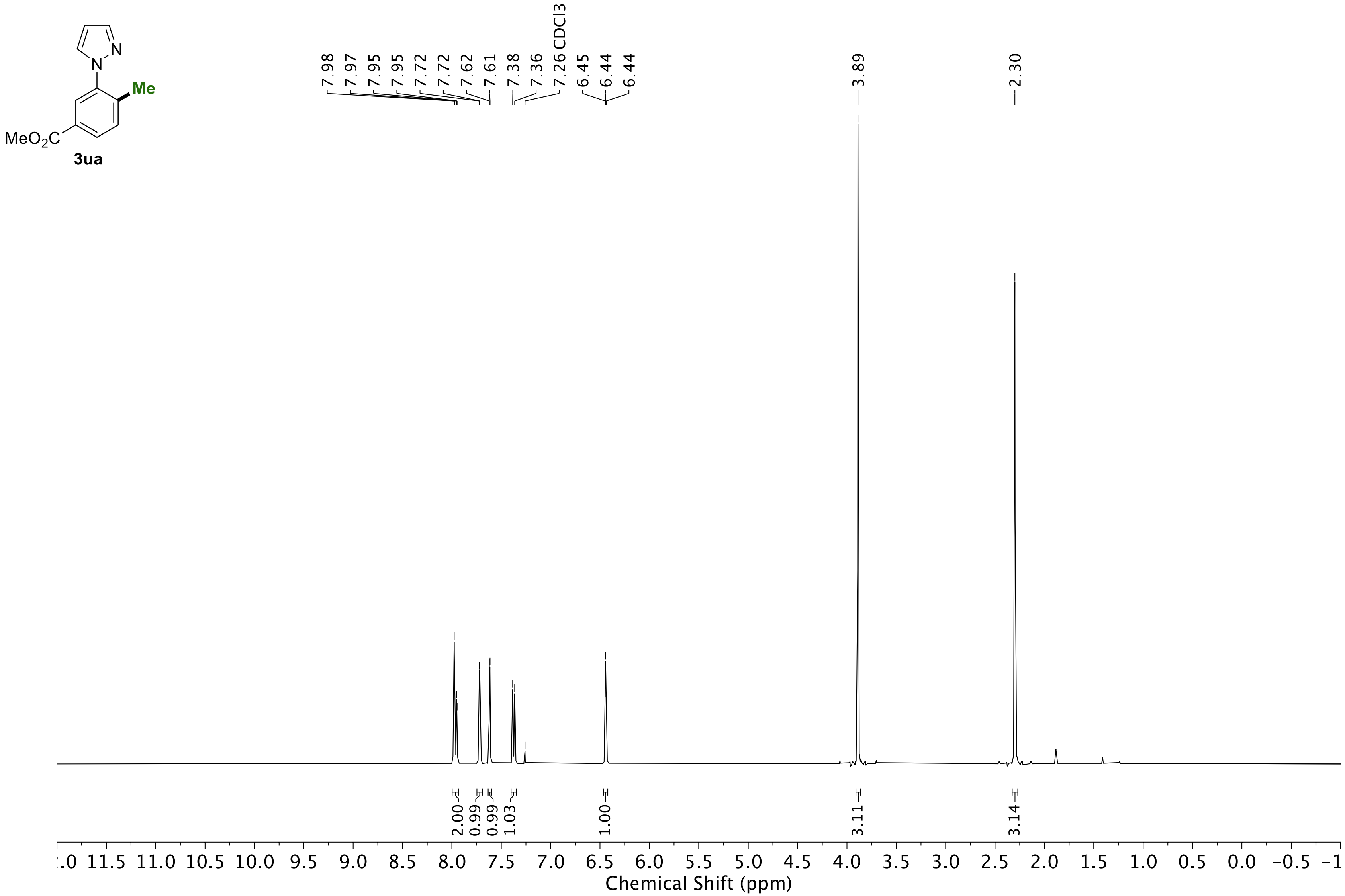

Figure S104. ^{13}C NMR (101 MHz, $CDCl_3$) of **3ua**.

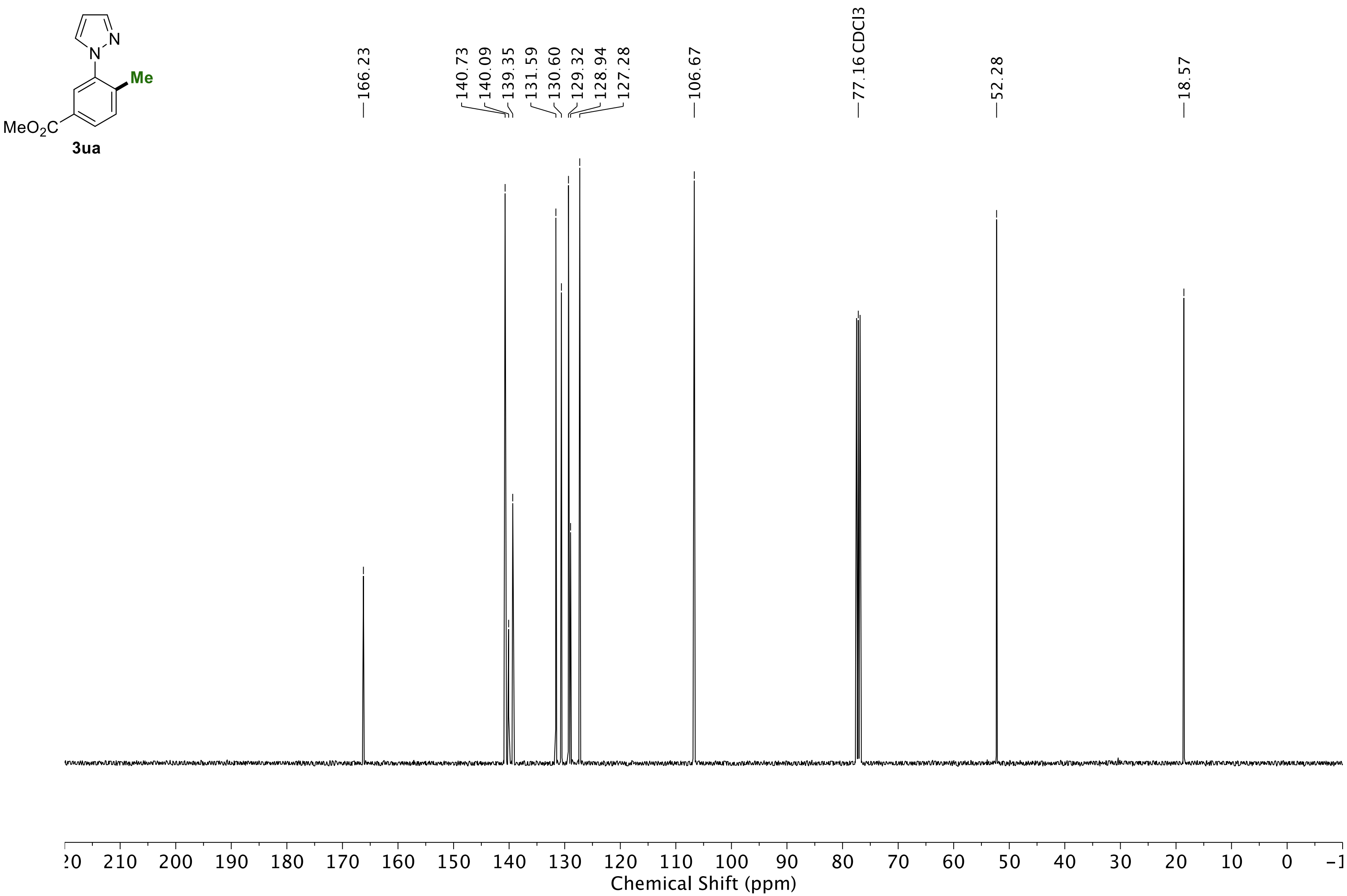

Figure S105. ^{1}H NMR (500 MHz, $CDCl_3$) of **3va**.

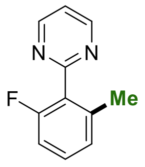

3va

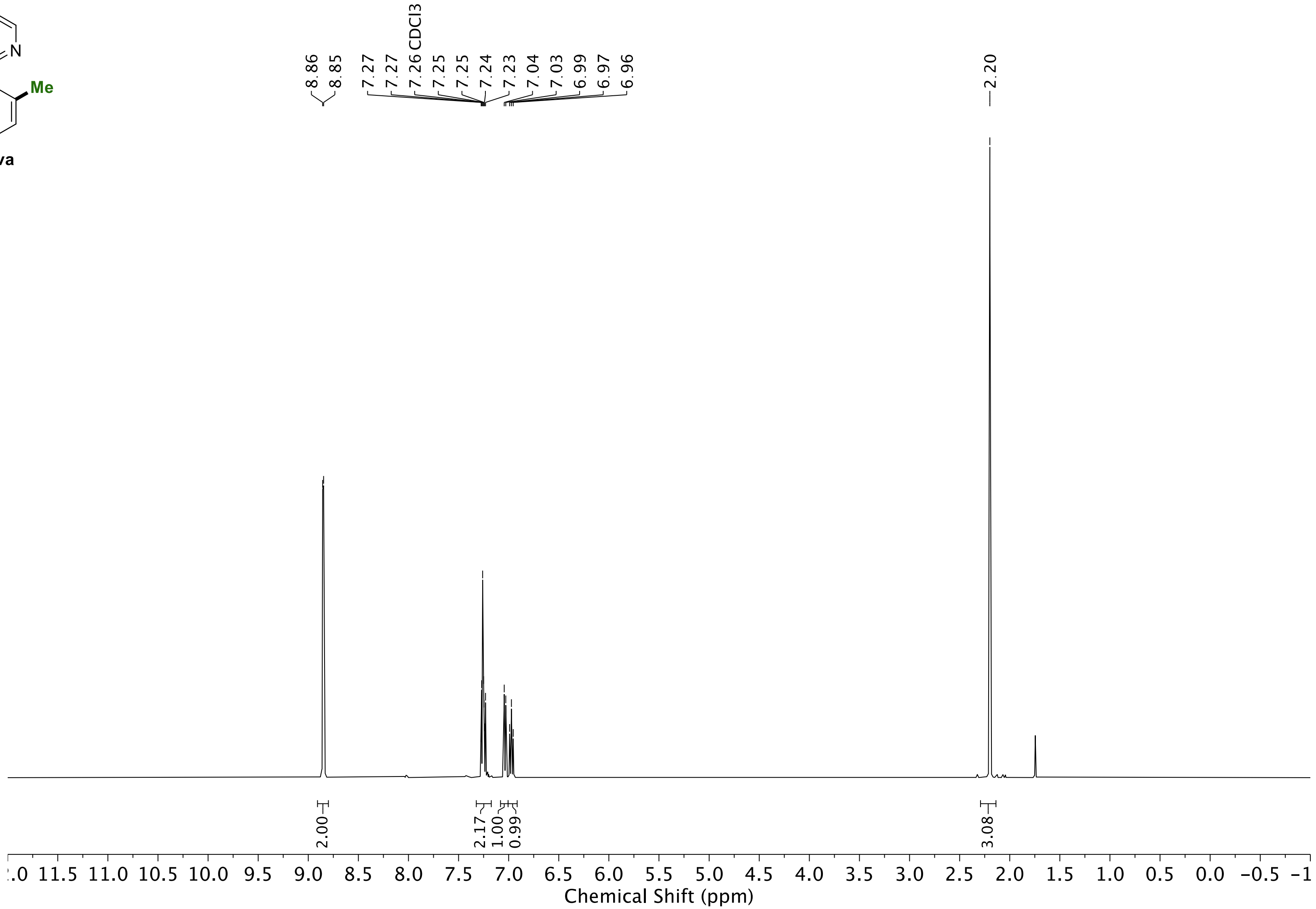

Figure S106. ^{13}C NMR (126 MHz, $CDCl_3$) of **3va**.

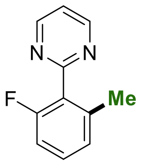

3va

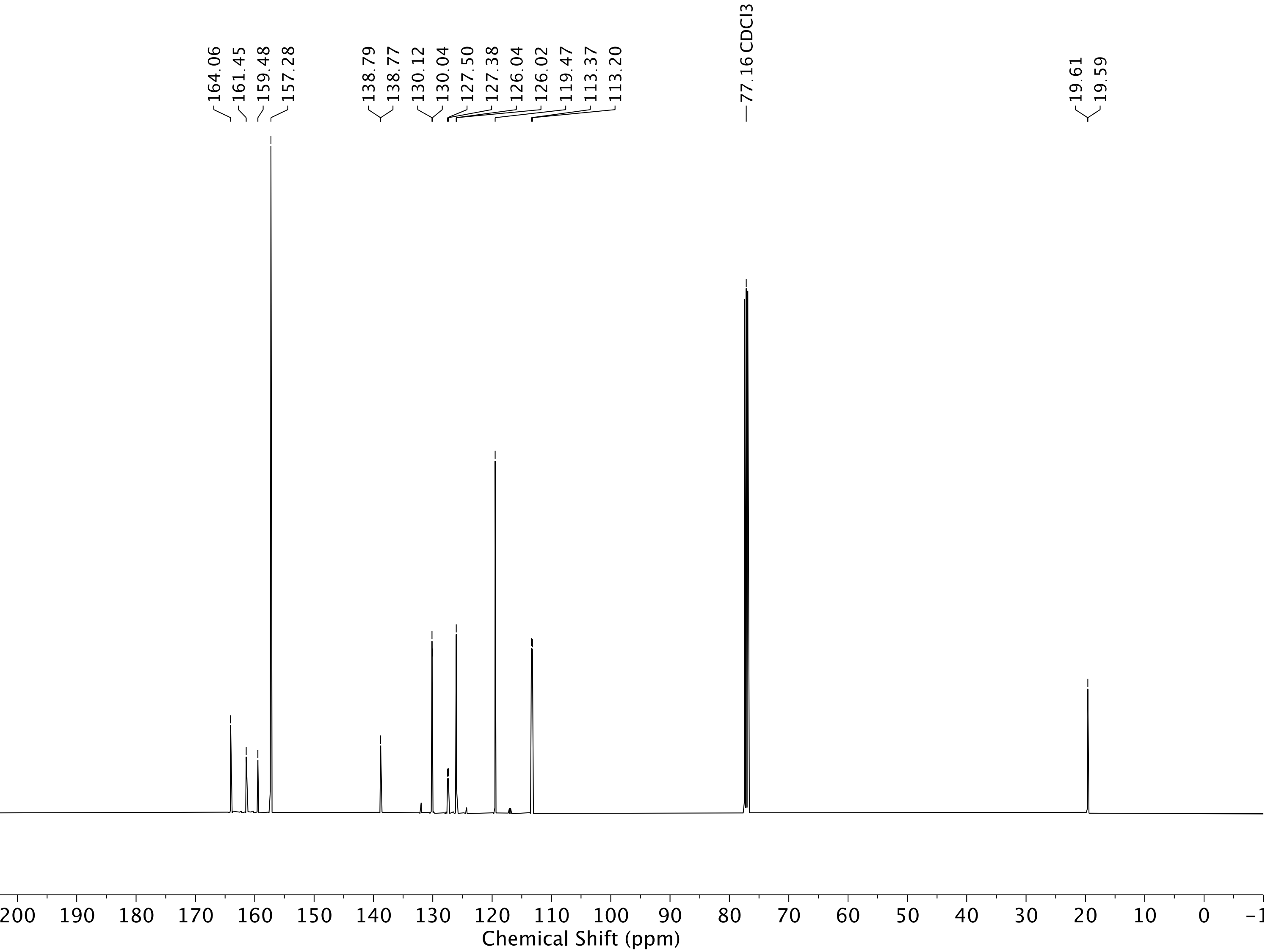

Figure S107. ^{19}F NMR (471 MHz, $CDCl_3$) of **3va**.

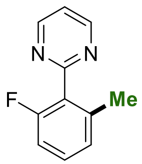

3va

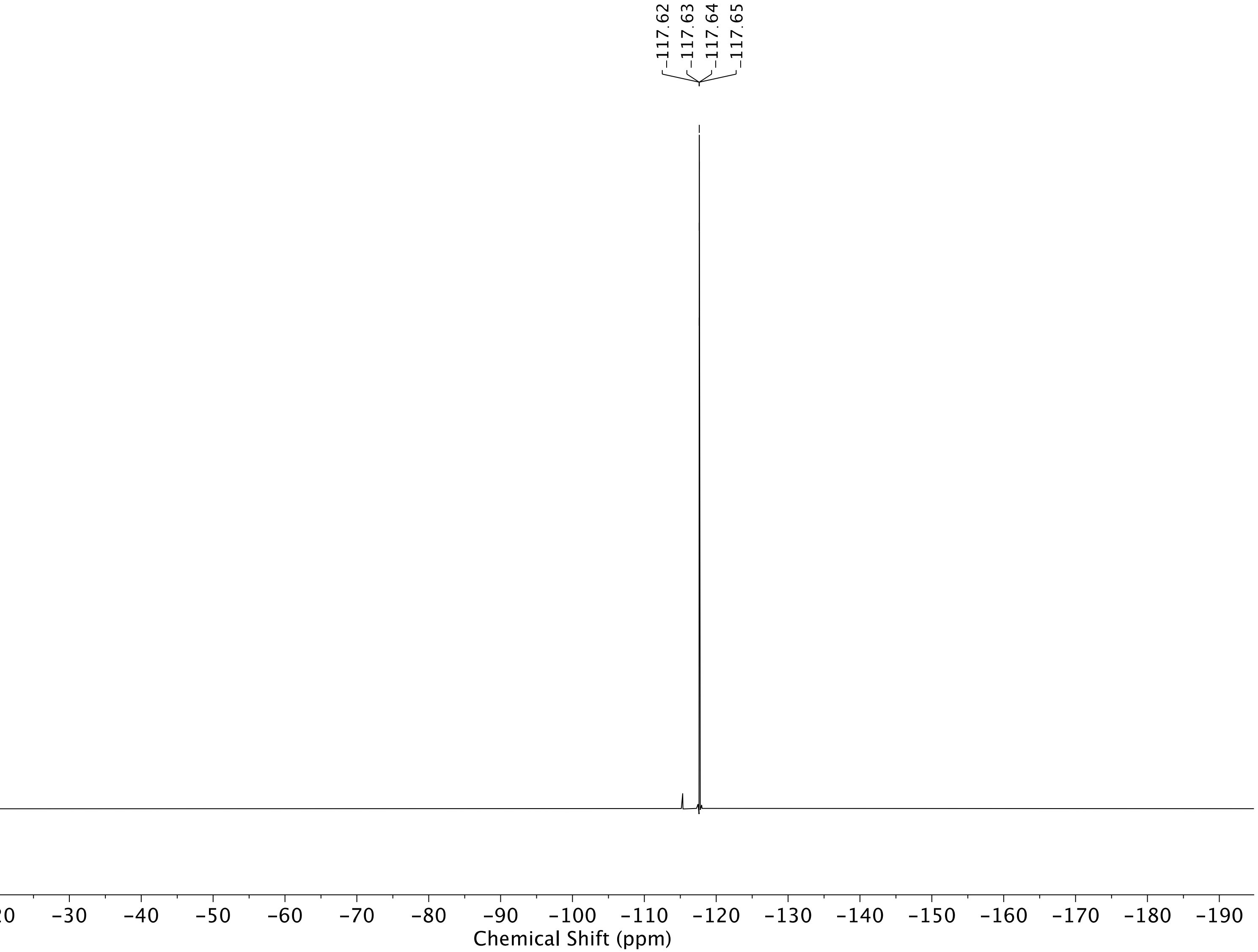

Figure S108. ^{1}H NMR (400 MHz, $CDCl_3$) of **3wa**.

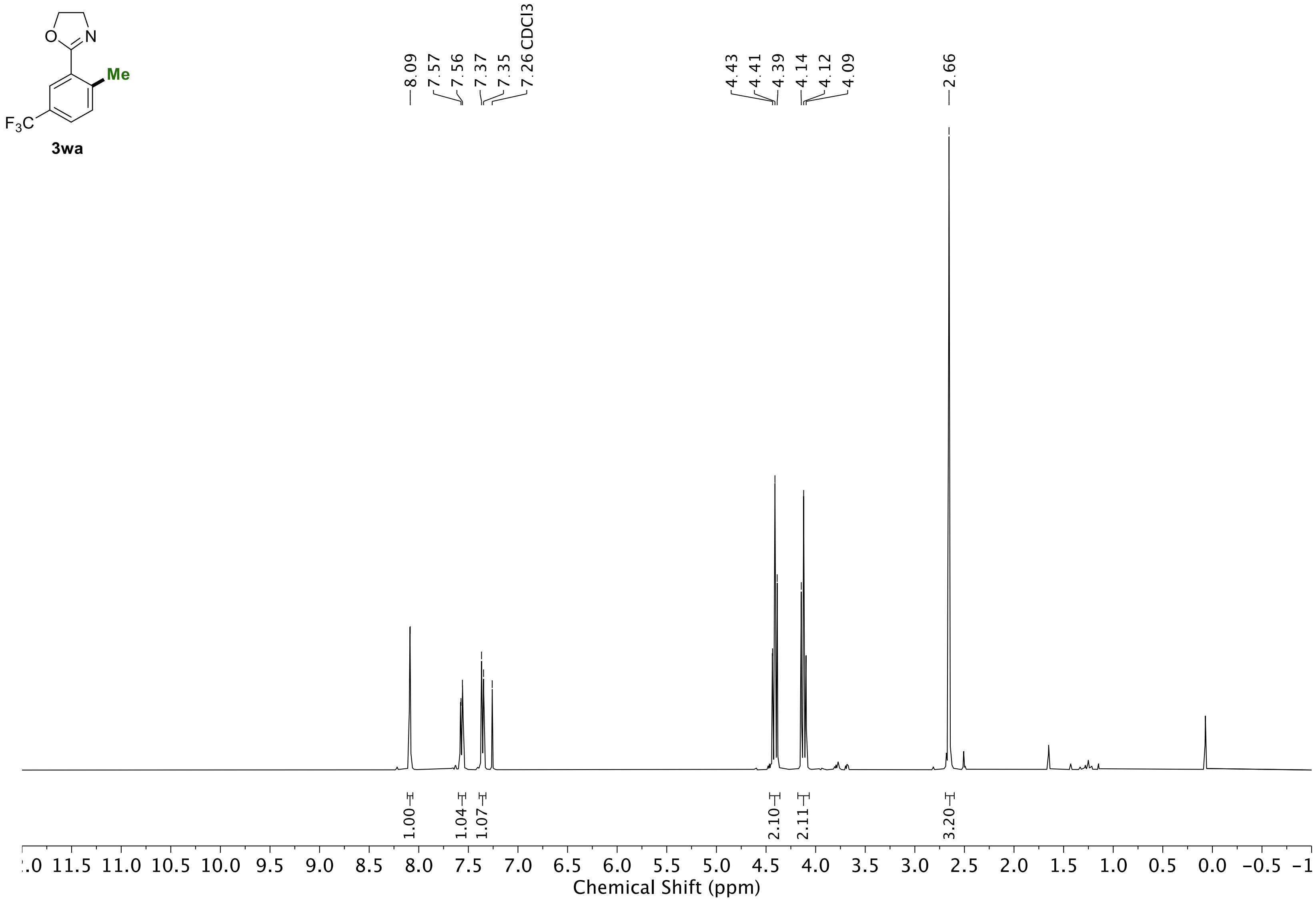

Figure S109. ^{13}C NMR (101 MHz, $CDCl_3$) of **3wa**.

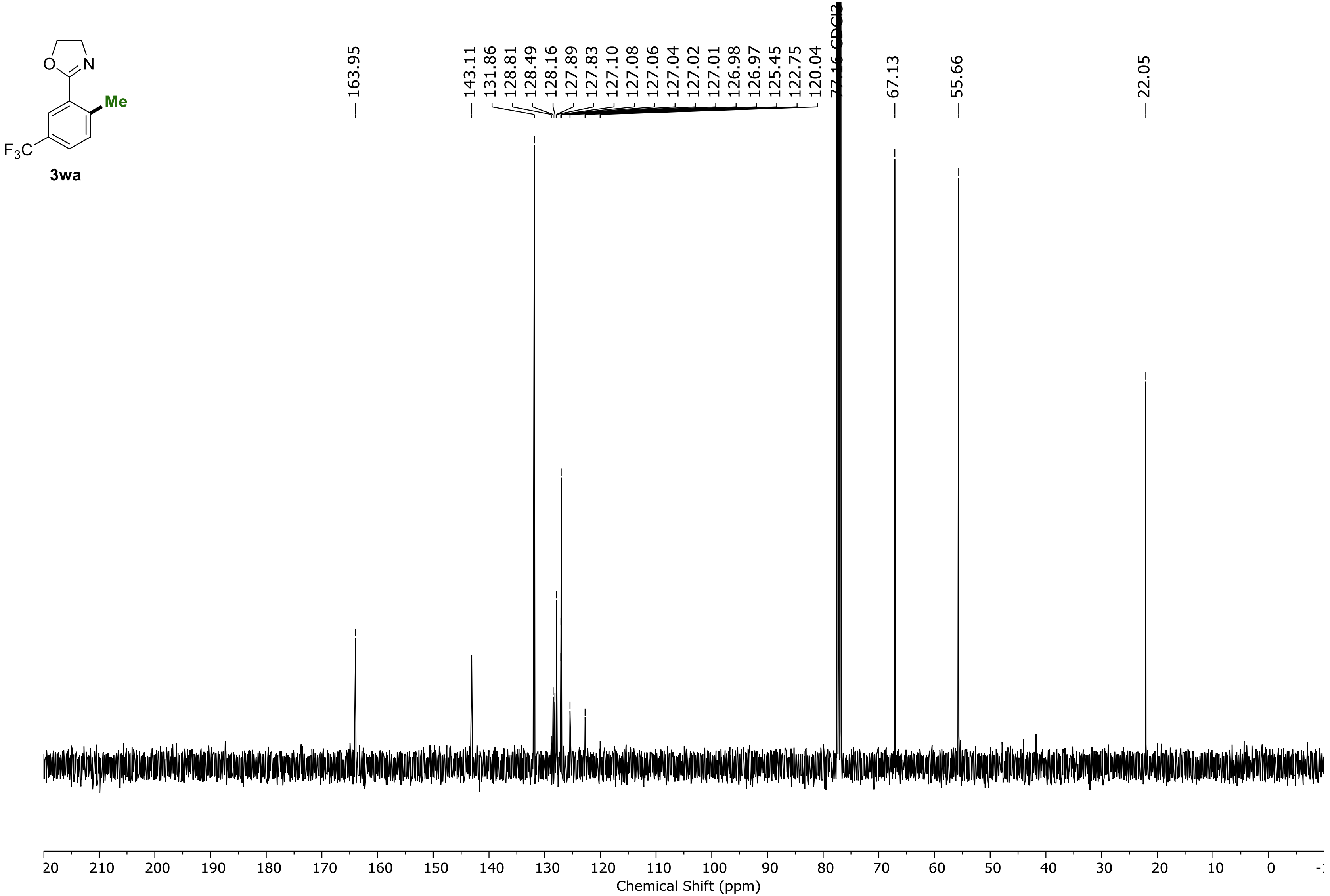

Figure S110. ^{19}F NMR (376 MHz, $CDCl_3$) of **3wa**.

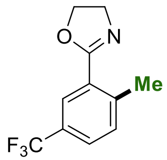

3wa

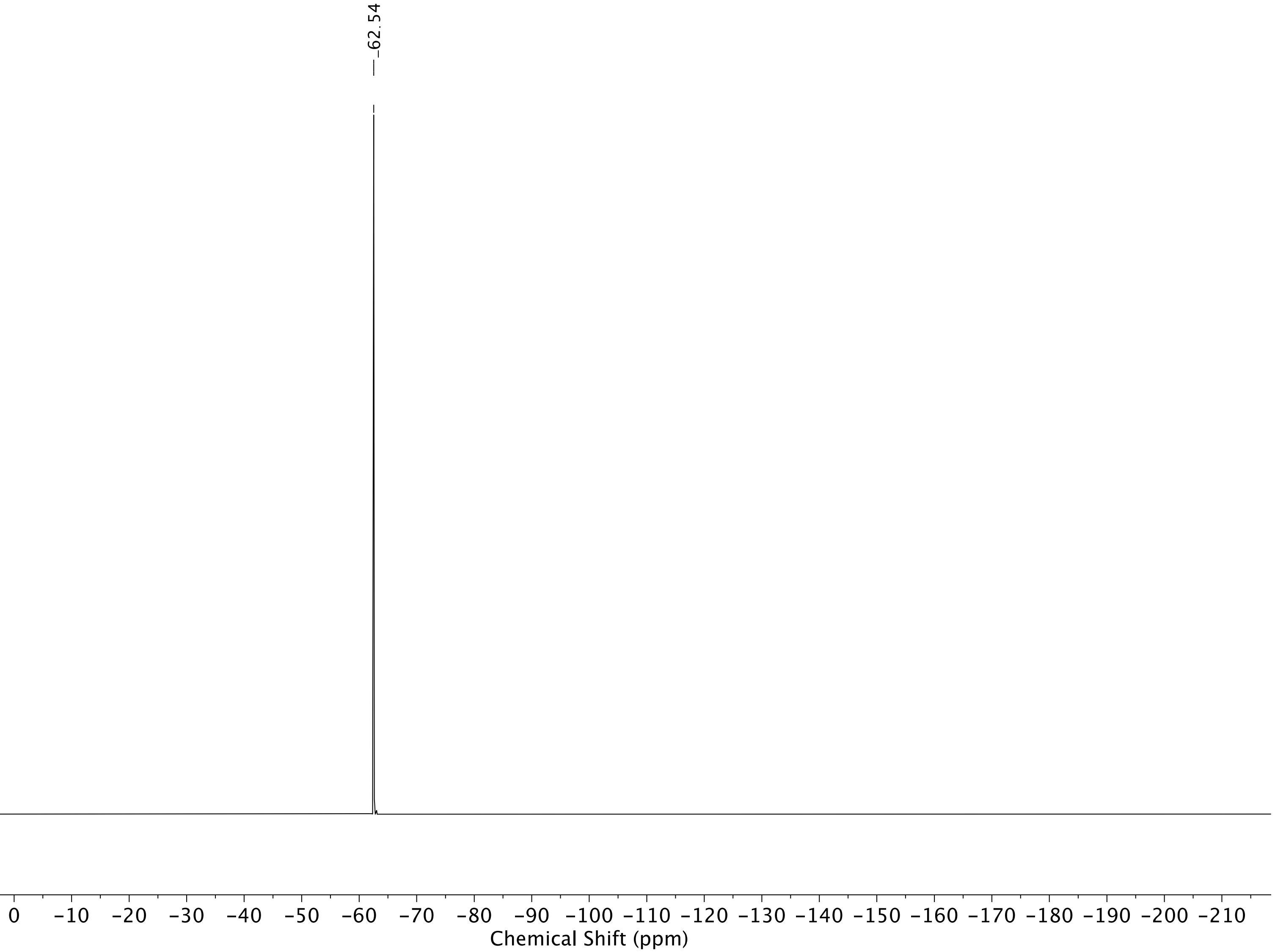

Figure S111. ^{1}H NMR (400 MHz, $CDCl_3$) of **4ab.**

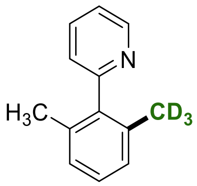

4ab

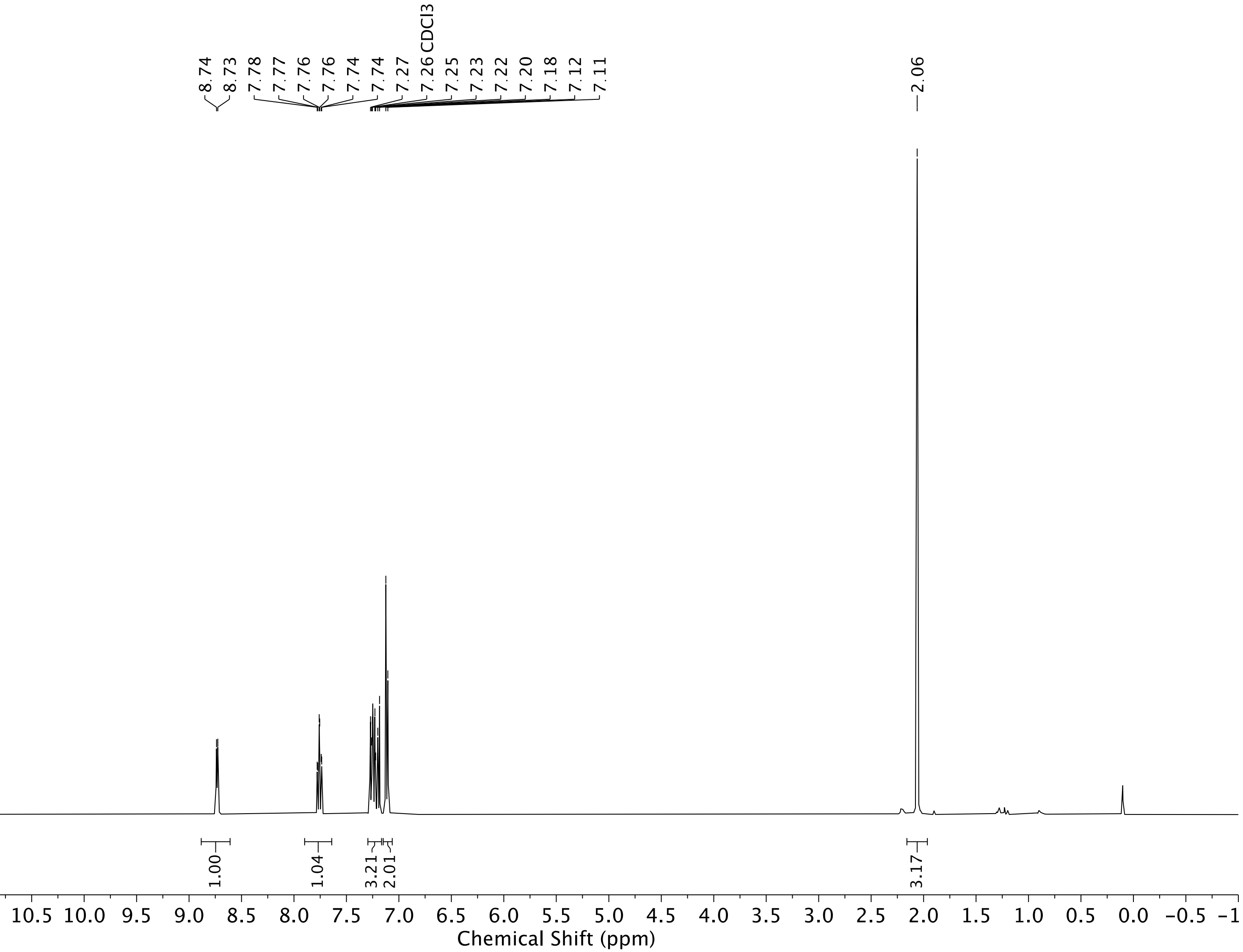

Figure S112. ^{13}C NMR (101 MHz, $CDCl_3$) of **xxx**.

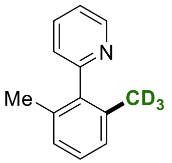

4ab

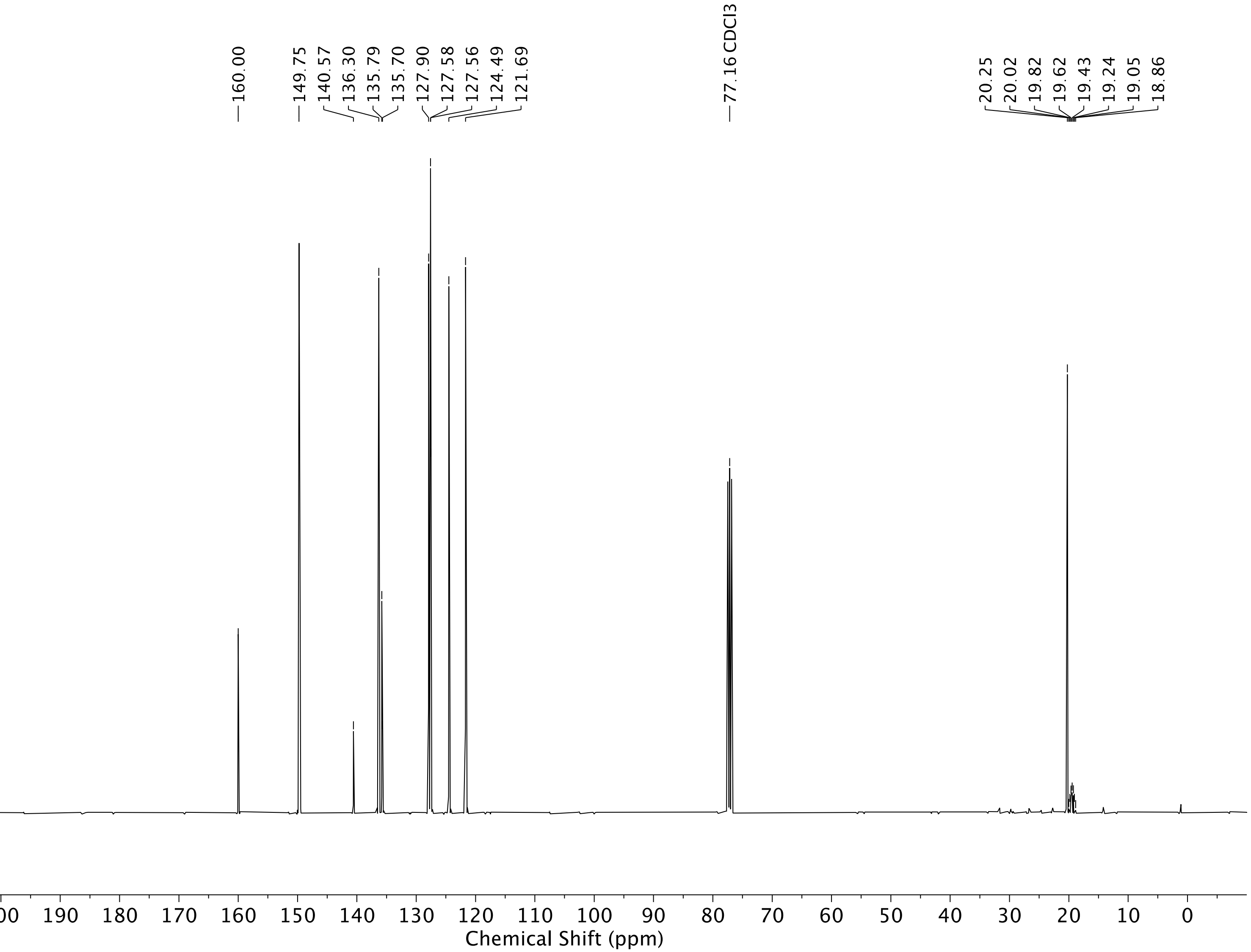

Figure S113. ^{2}H NMR (63 MHz, $CDCl_3$) of **xxx**

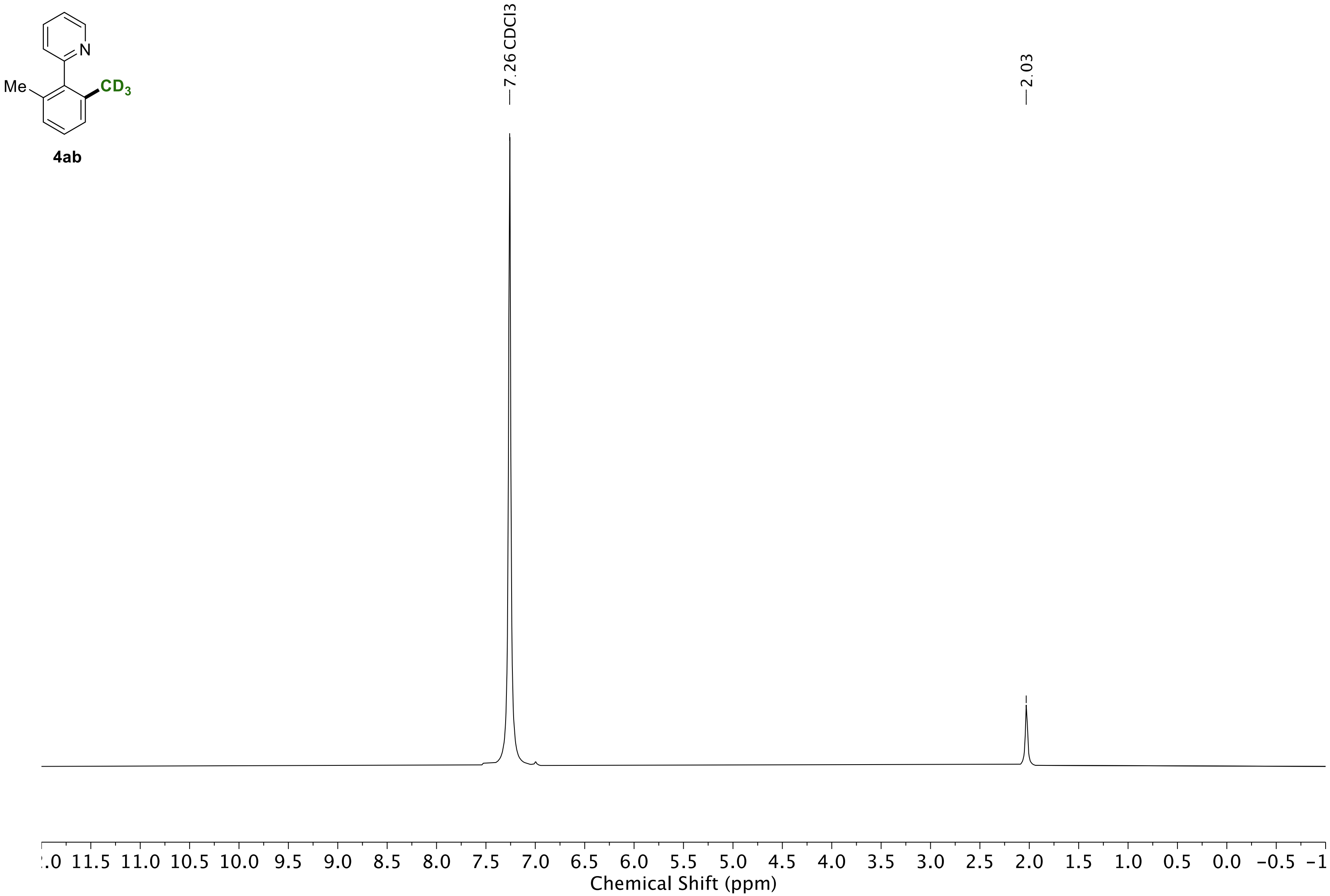

Figure S114. ^{1}H NMR (500 MHz, $CDCl_3$) of **3mb**.

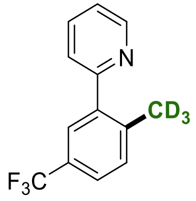

3mb

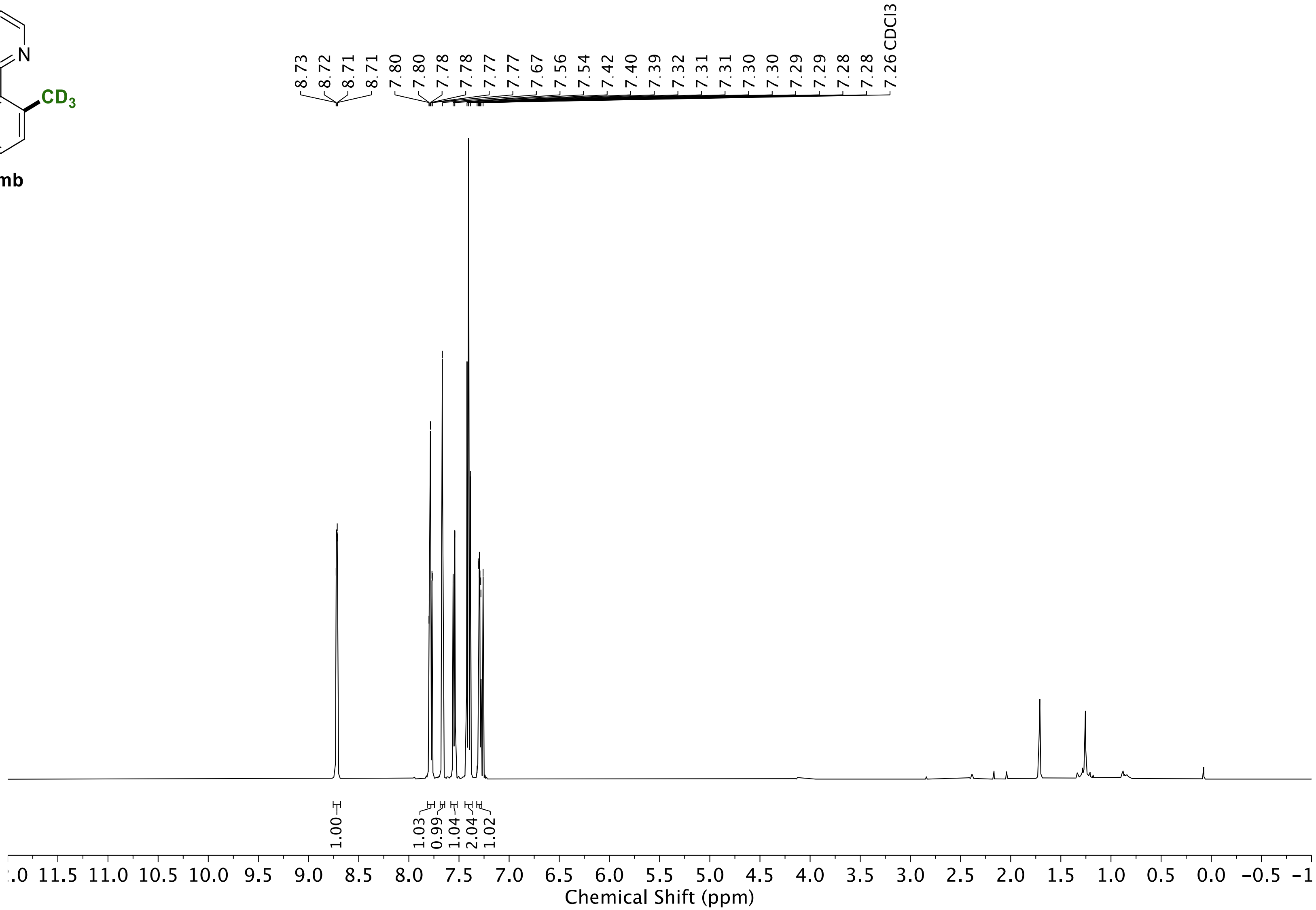

Figure S115. ^{13}C NMR (126 MHz, $CDCl_3$) of **3mb**.

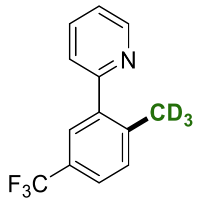

3mb

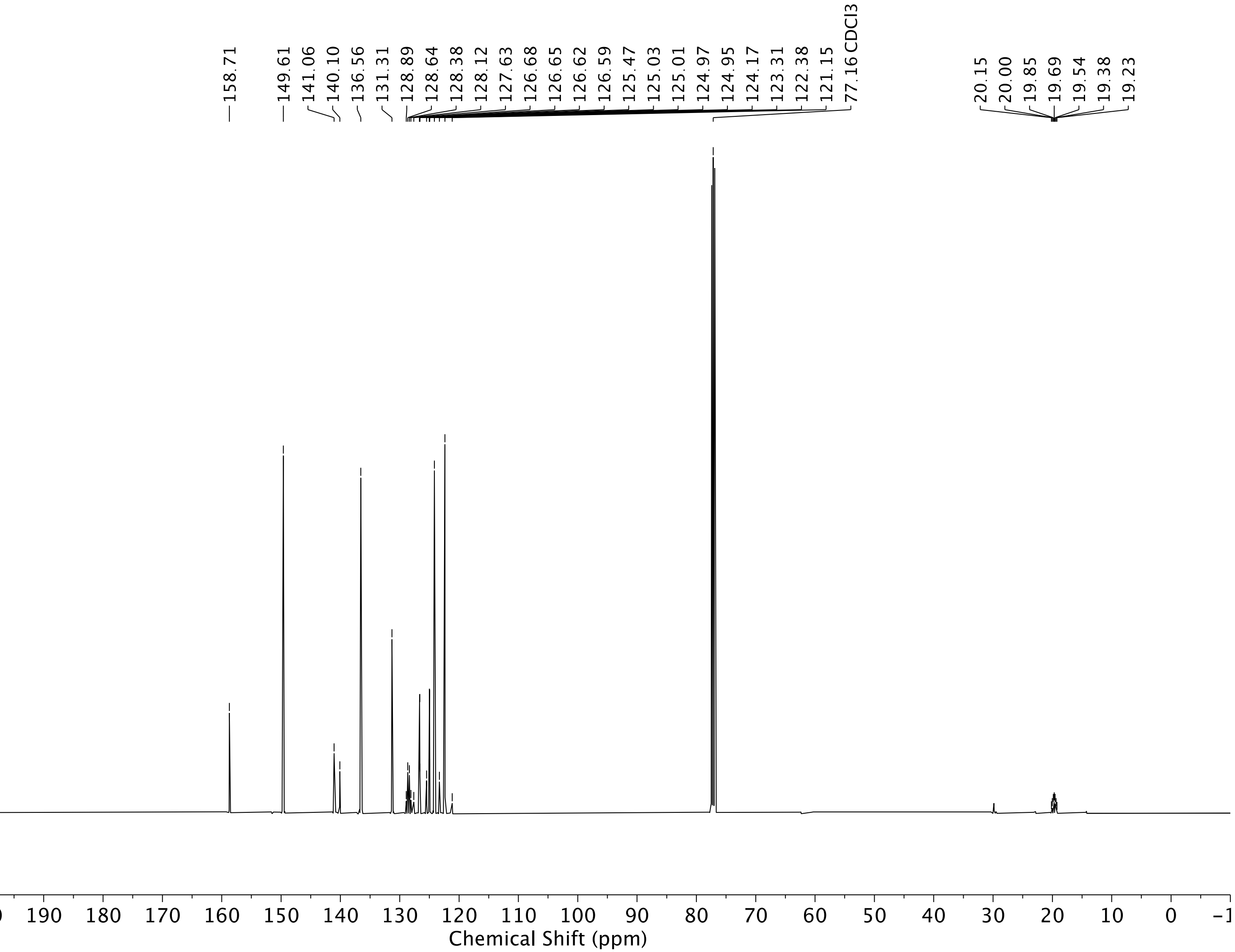

Figure S116. ^{19}F NMR (471 MHz, $CDCl_3$) of **3mb**.

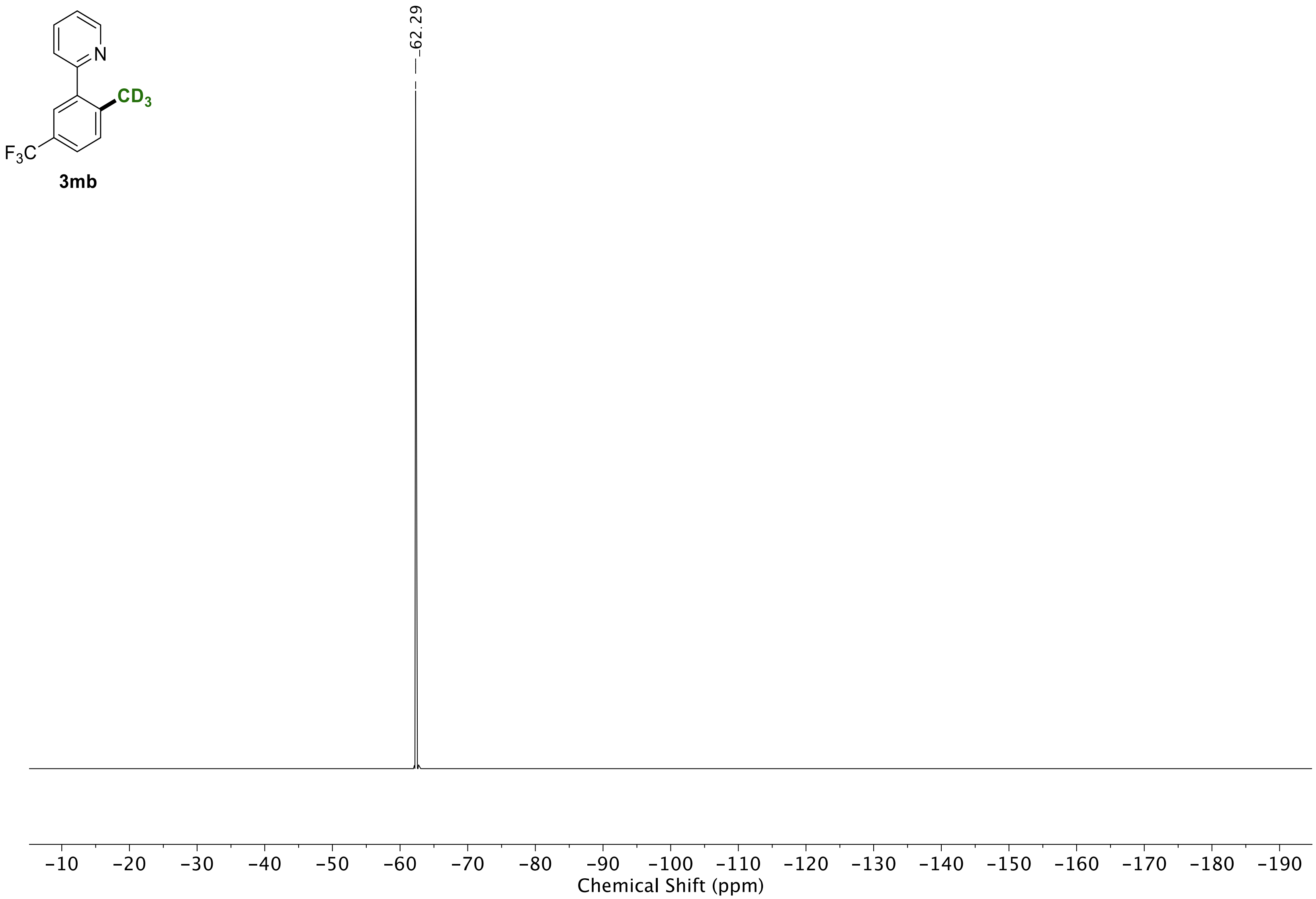

3mb

Figure S117. ^{2}H NMR (77 MHz, $CDCl_3$) of **3mb**.

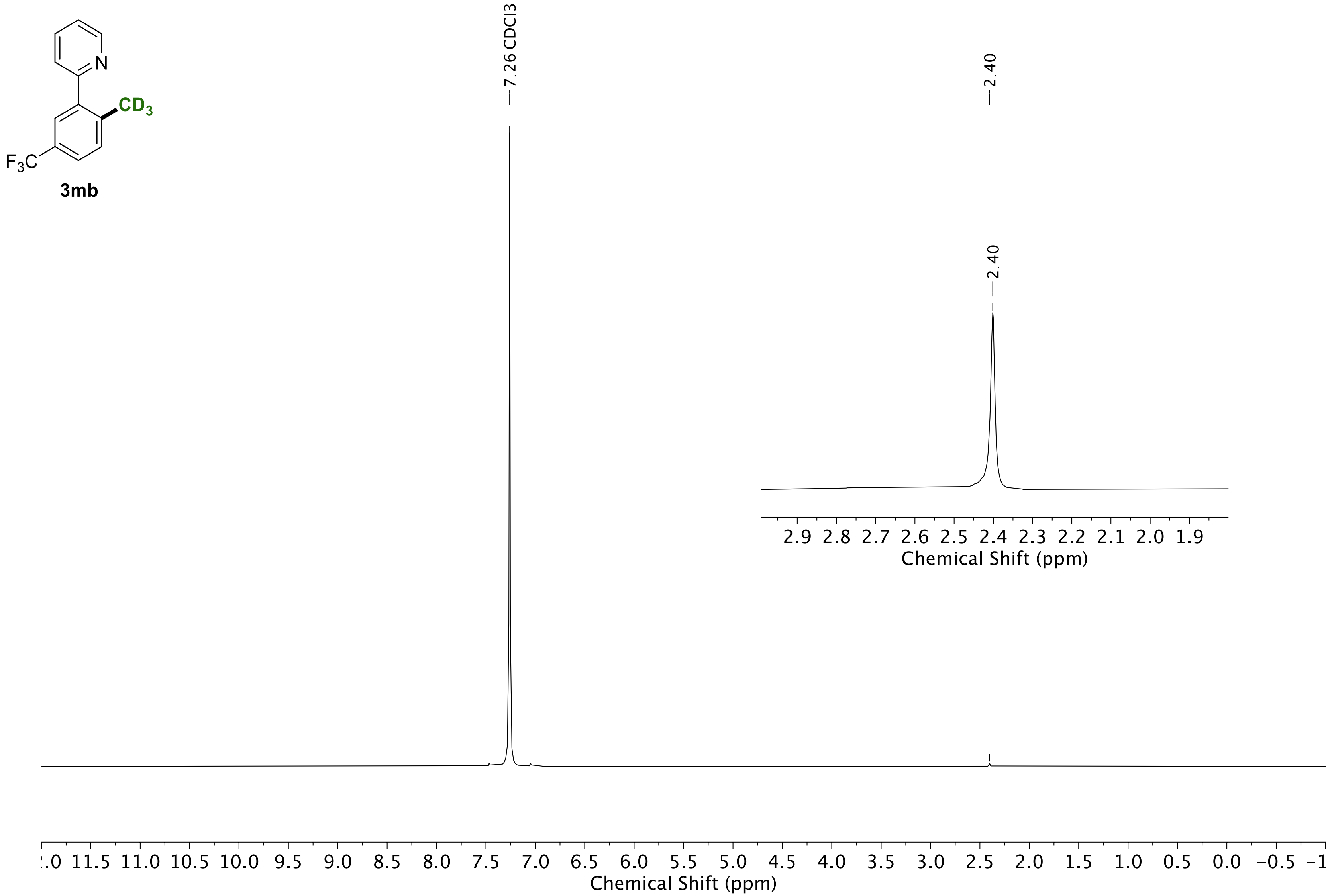

Figure S118. ^{1}H NMR (400 MHz, $CDCl_3$) of **3jb**.

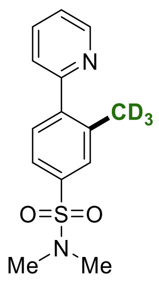

3jb

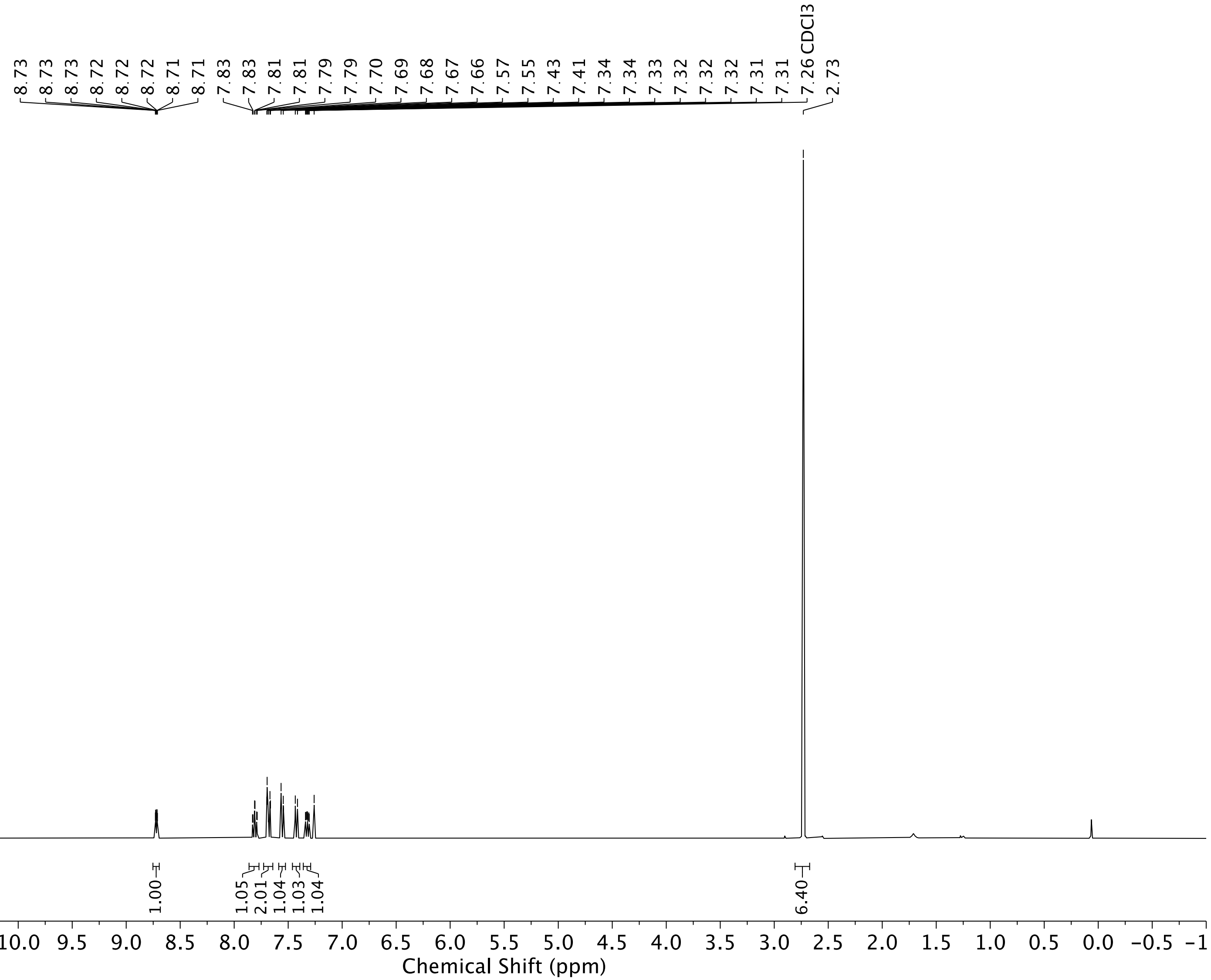

Figure S119. ^{13}C NMR (101 MHz, $CDCl_3$) of **3jb**.

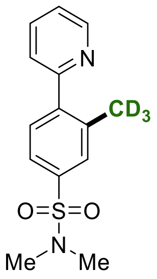

3jb

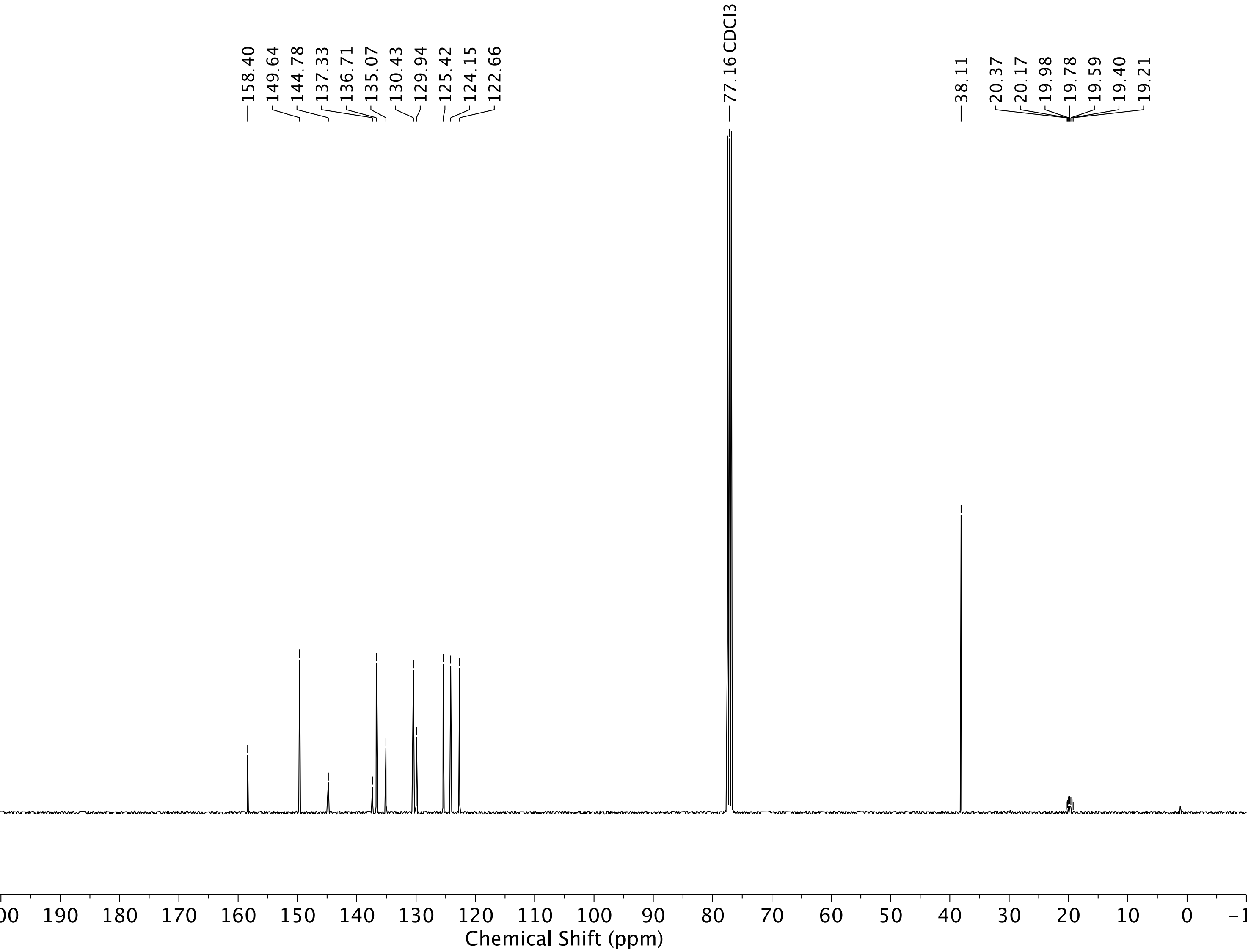

Figure S120. ^{2}H NMR (77 MHz, $CDCl_3$) of **3jb**.

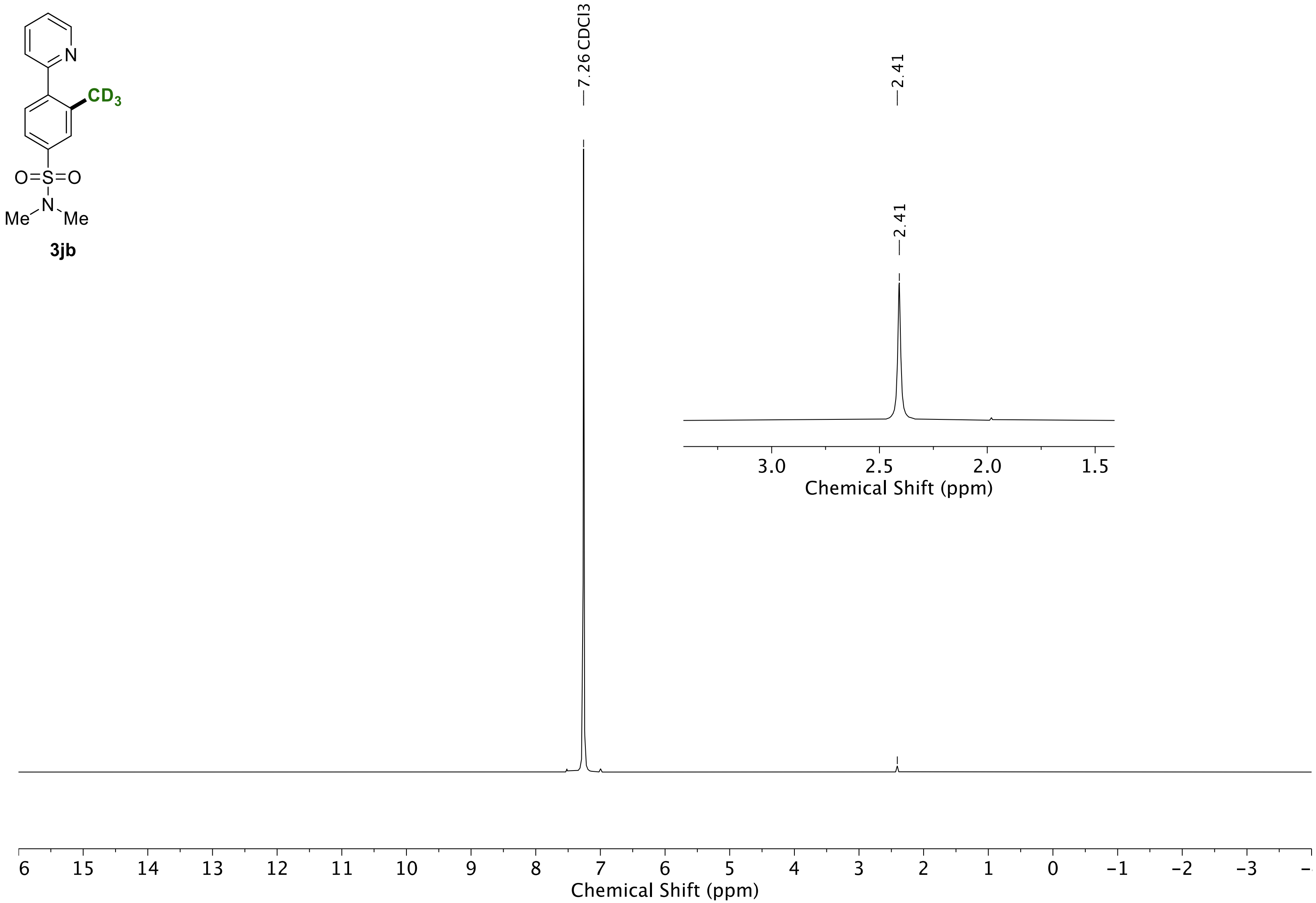

Figure S121. ^{1}H NMR (400 MHz, $CDCl_3$) of **3ub**.

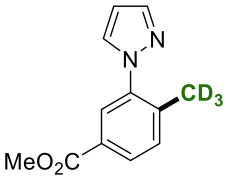

3ub

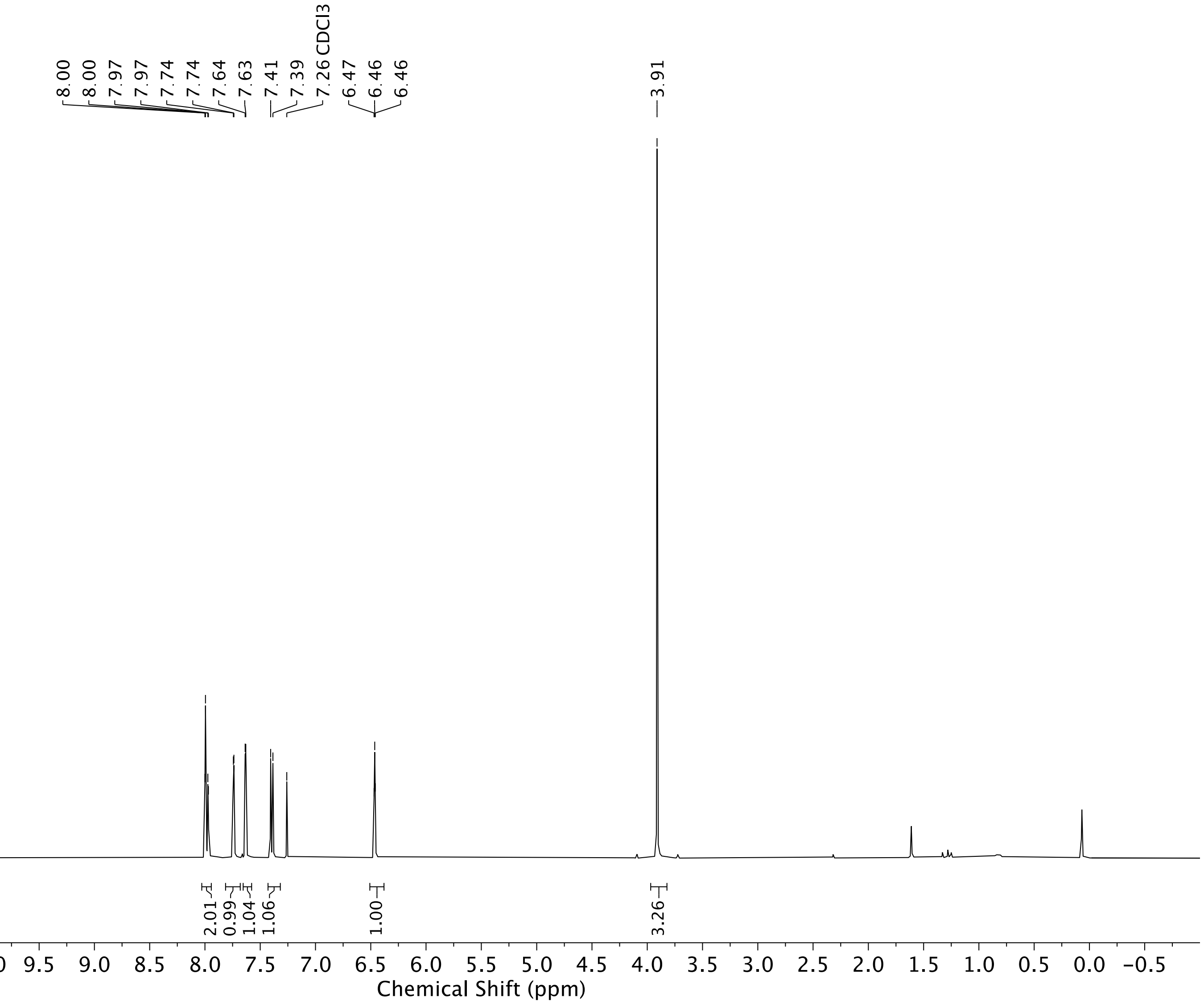

Figure S122. ^{13}C NMR (126 MHz, $CDCl_3$) of **3ub**.

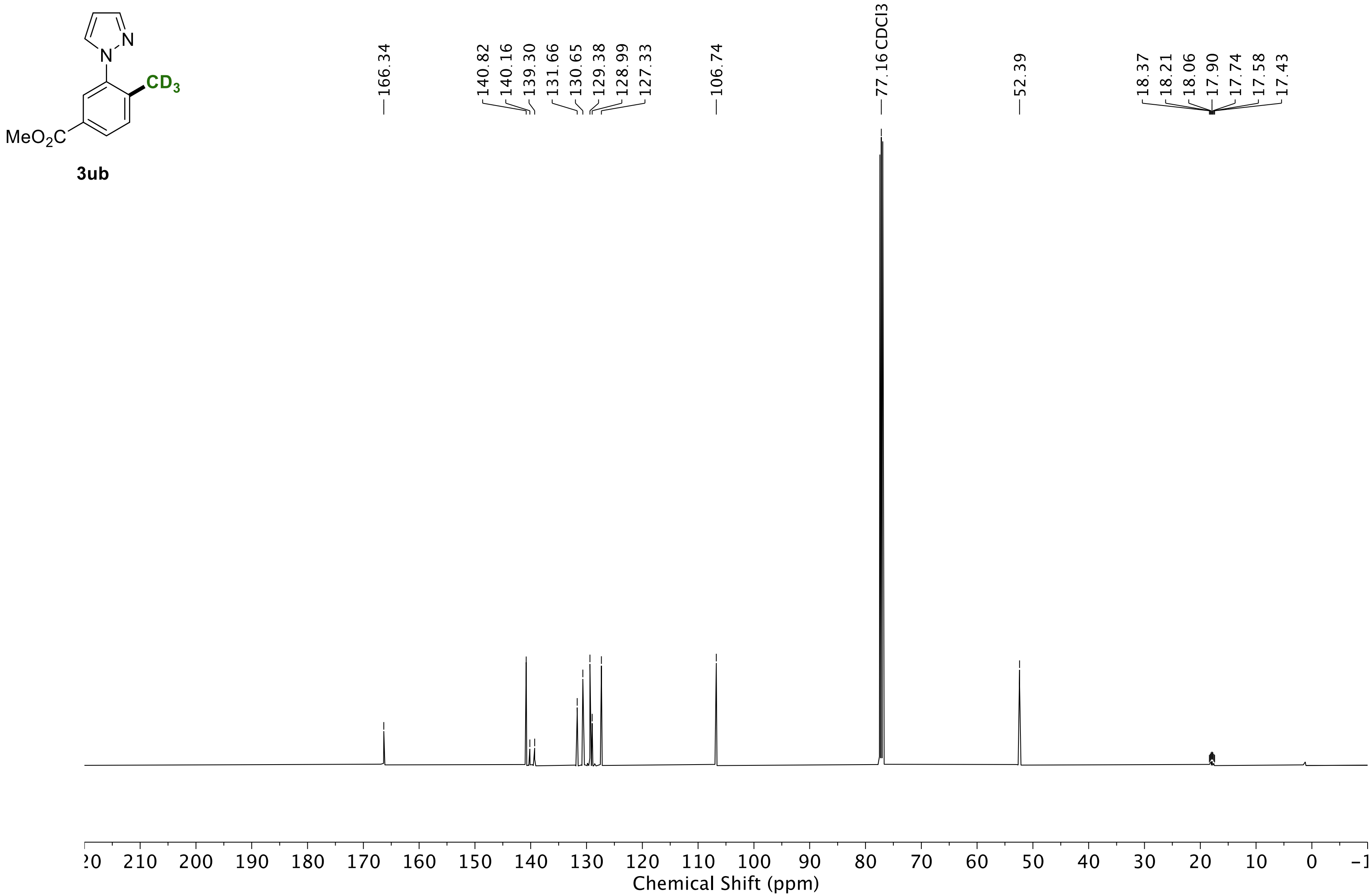

Figure S123. ^{2}H NMR (77 MHz, $CDCl_3$) of **3ub**.

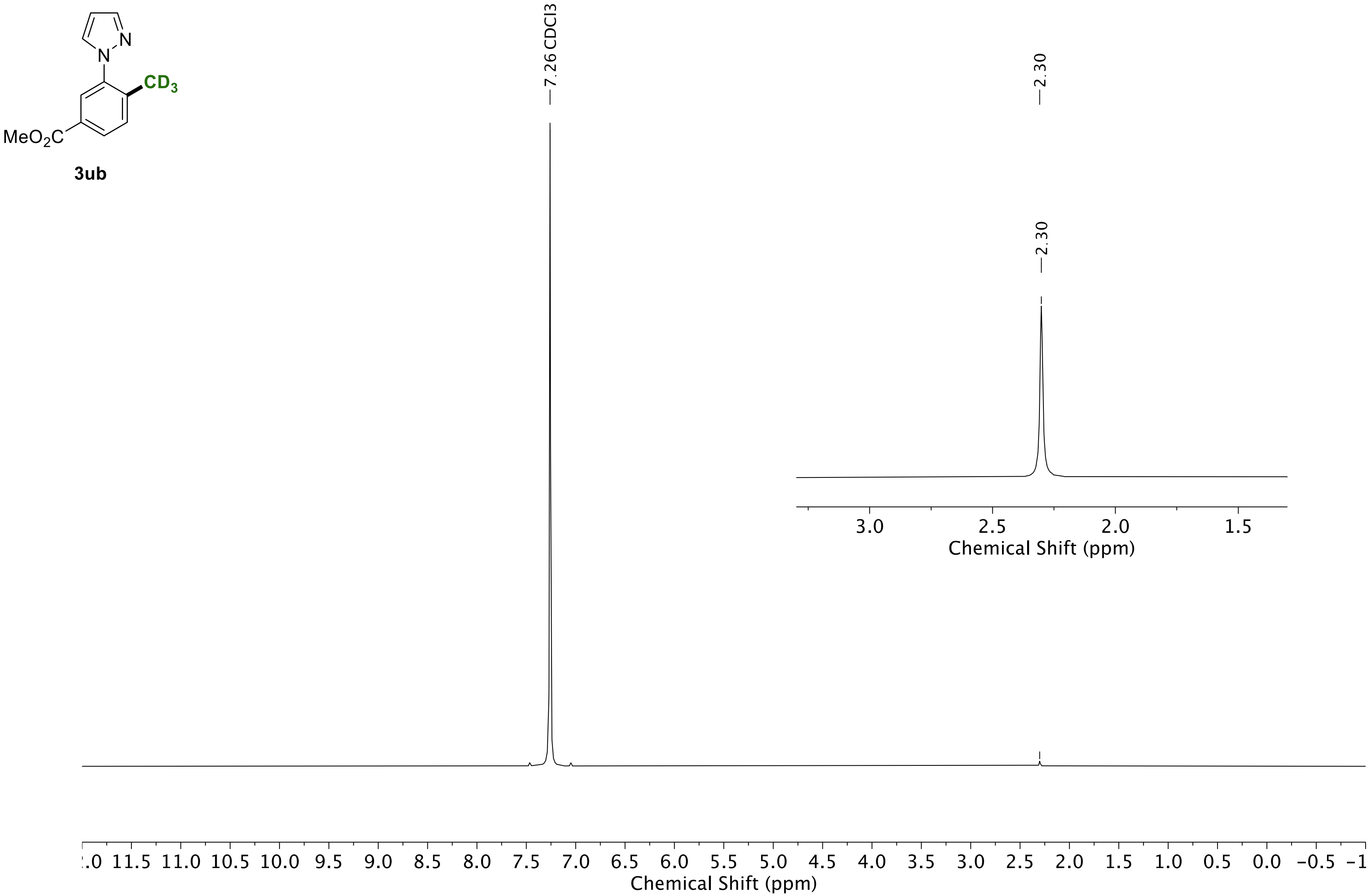

Figure S124. ^{1}H NMR (400 MHz, $CDCl_3$) of **3vb**.

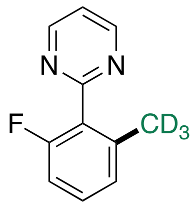

3vb

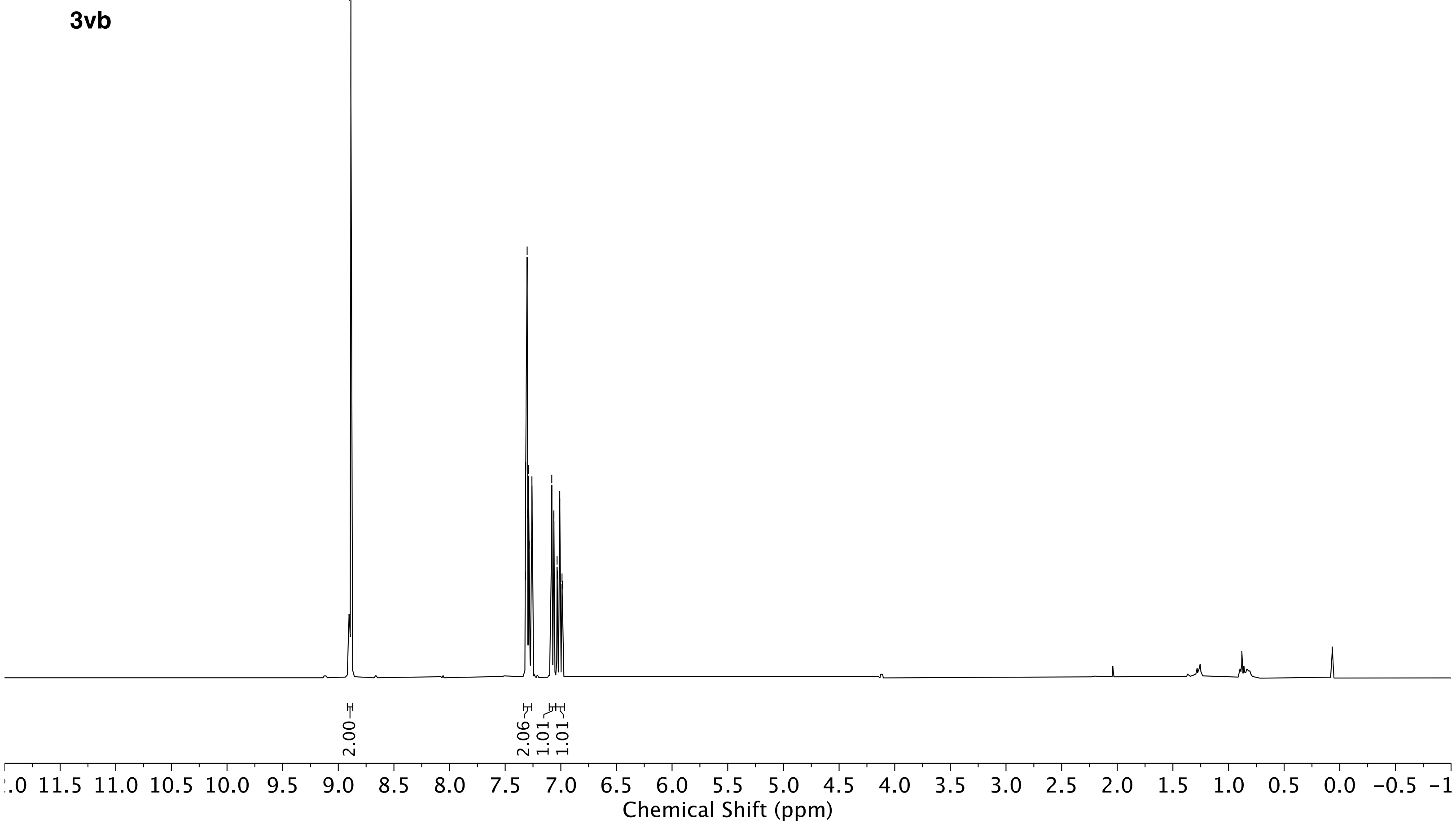

Figure S125. ^{13}C NMR (156 MHz, $CDCl_3$) of **3vb**.

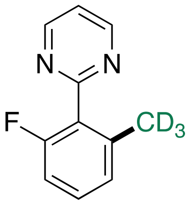

3vb

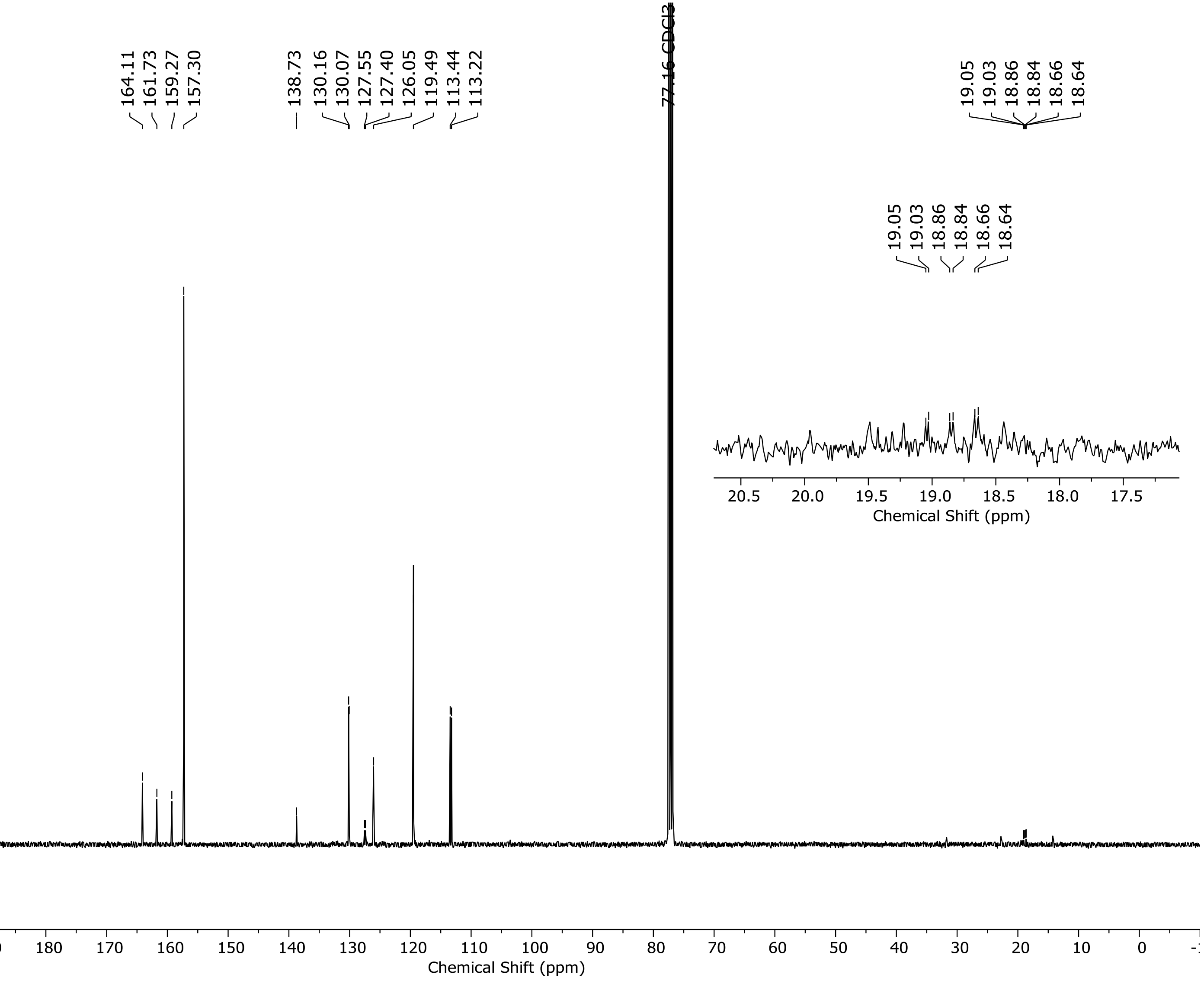

Figure S126. ^{19}F NMR (376 MHZ, $CDCl_3$) of **3vb**.

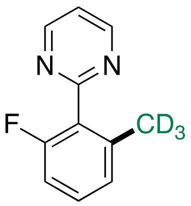

3vb

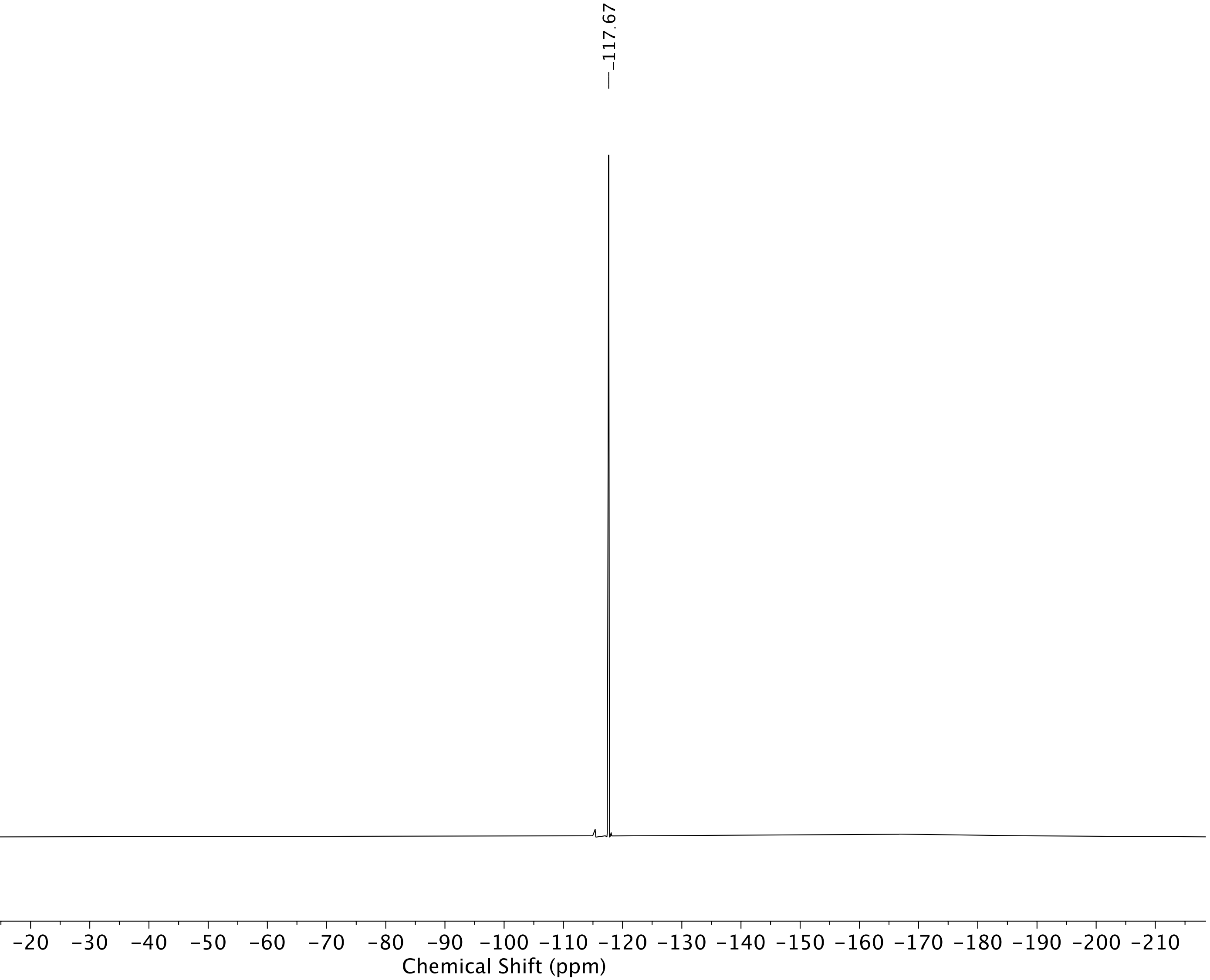

Figure S127. ^{2}H NMR (61 MHz, $CDCl_3$) of **3vb**.

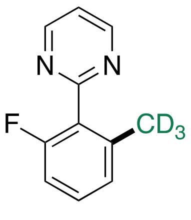

3vb

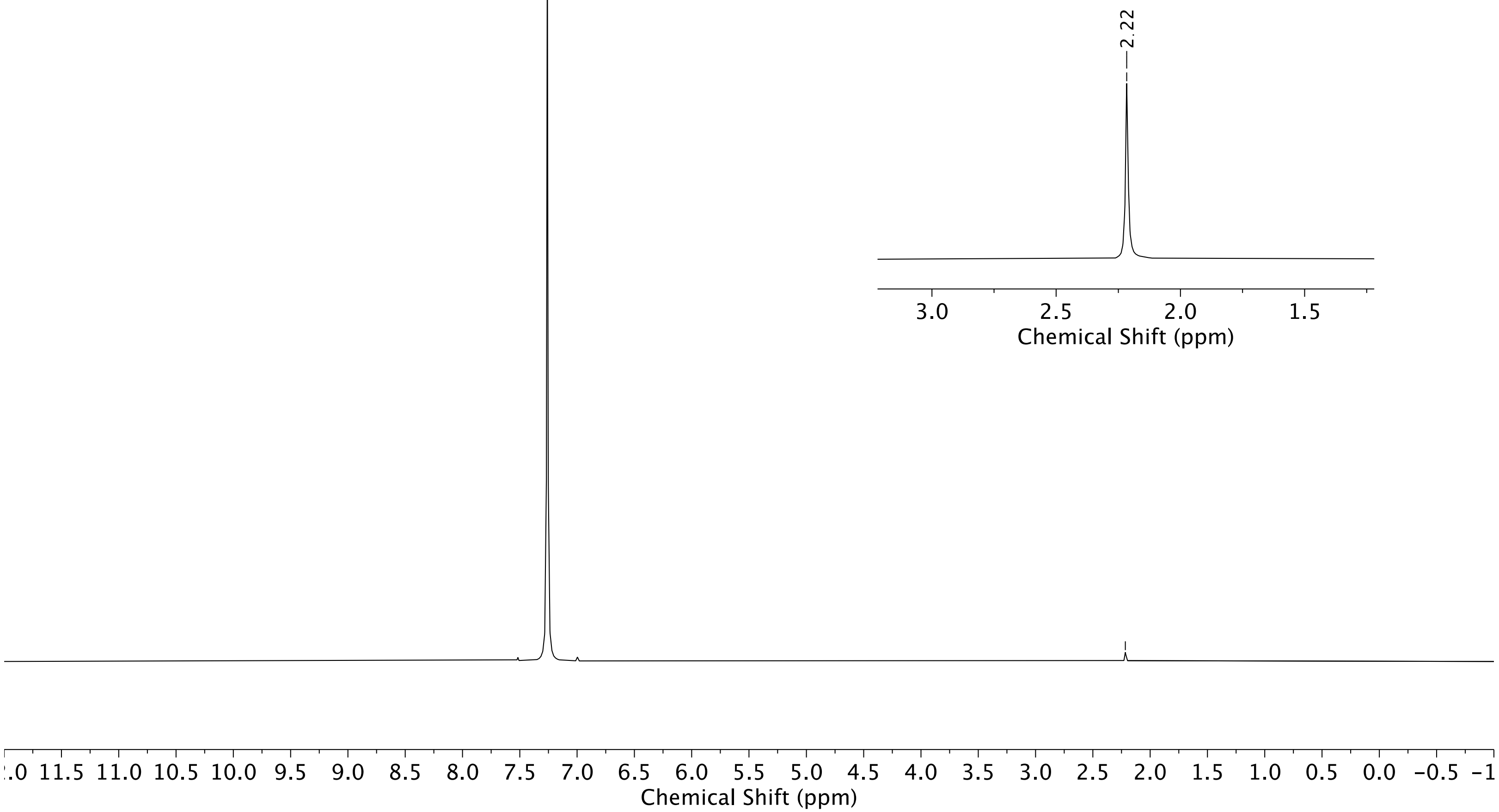

Figure S128. ^{1}H NMR (400 MHz, CD_3CN) of **3wb**.

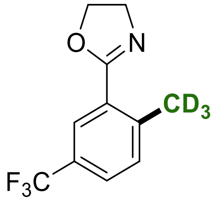

3wb

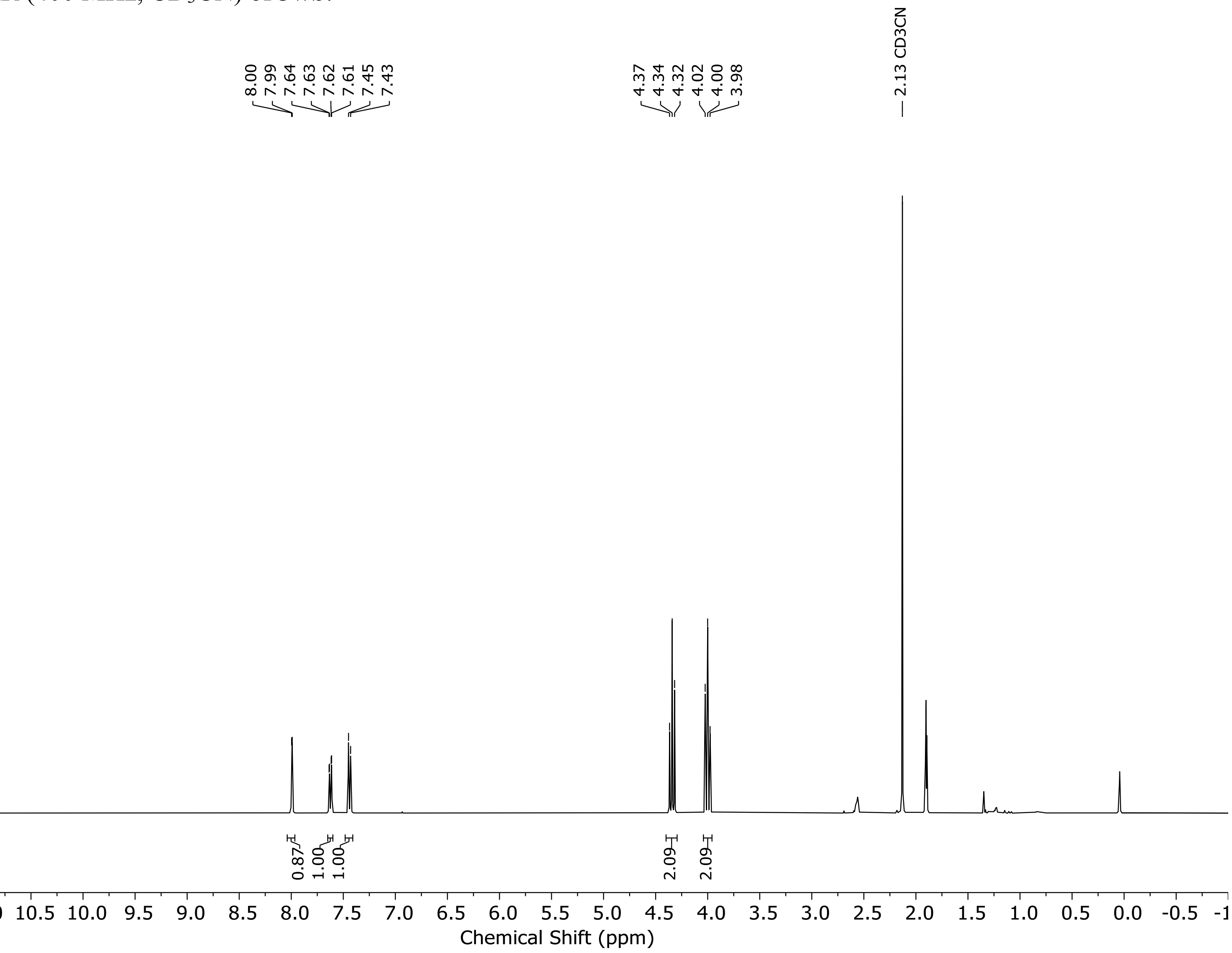

Figure S129. ^{13}C NMR (126 MHz, $CDCl_3$) of **3wb**.

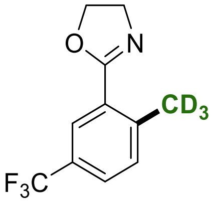

3wb

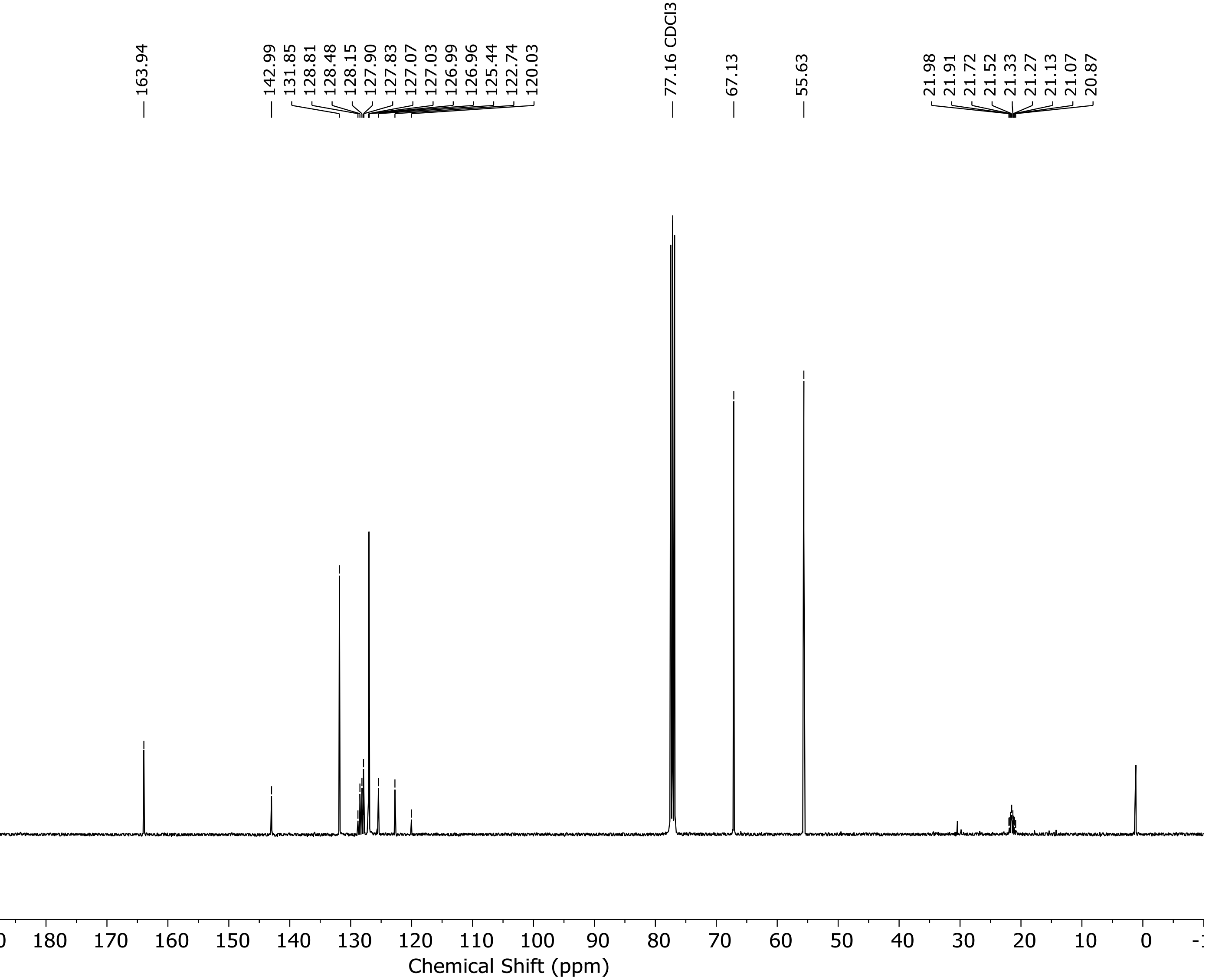

Figure S130. ^{19}F NMR (376 MHz, $CDCl_3$) of **3wb.**

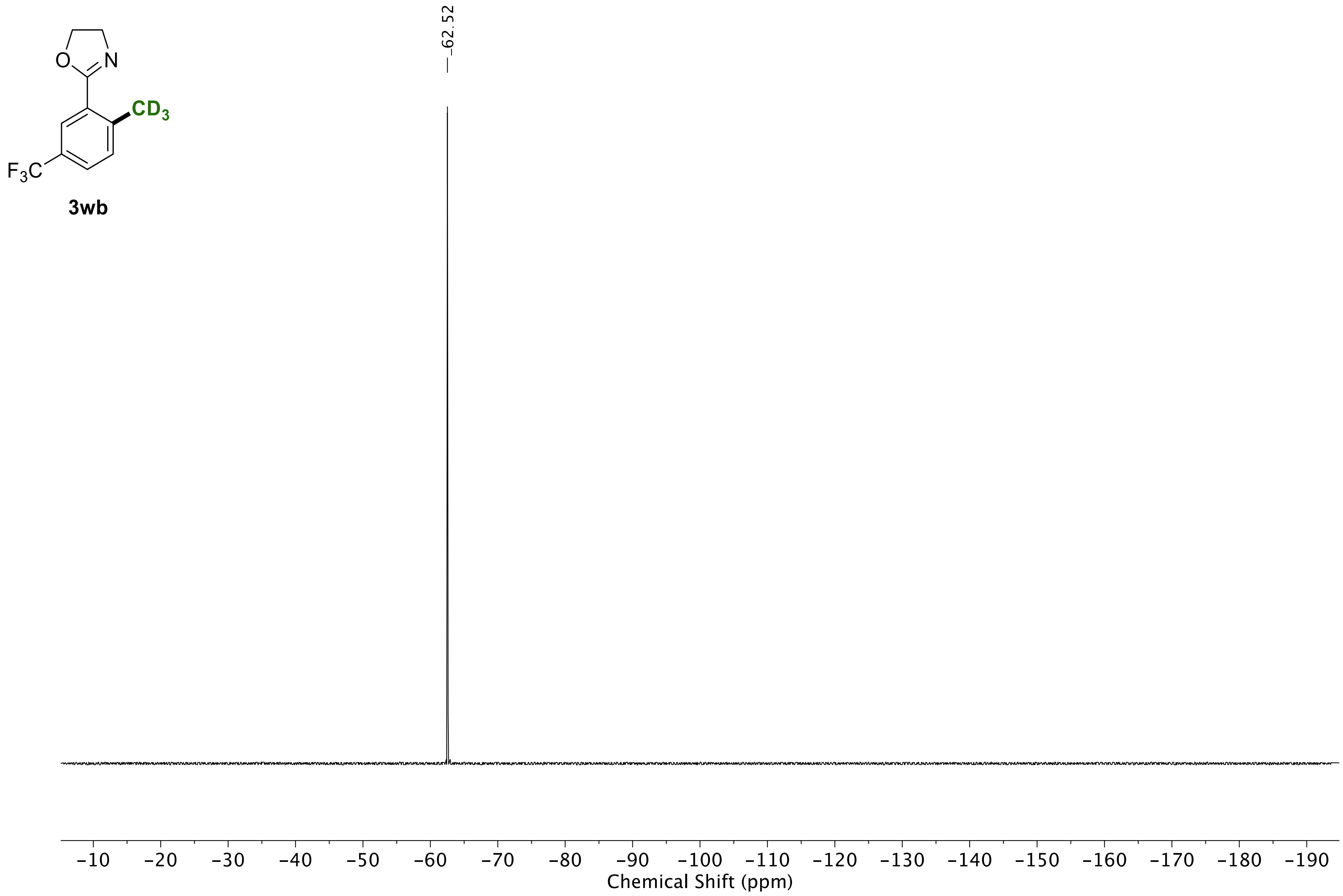

Figure S131. ^{2}H NMR (77 MHz, $CDCl_3$) of **3wb.**

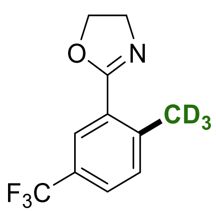
3wb

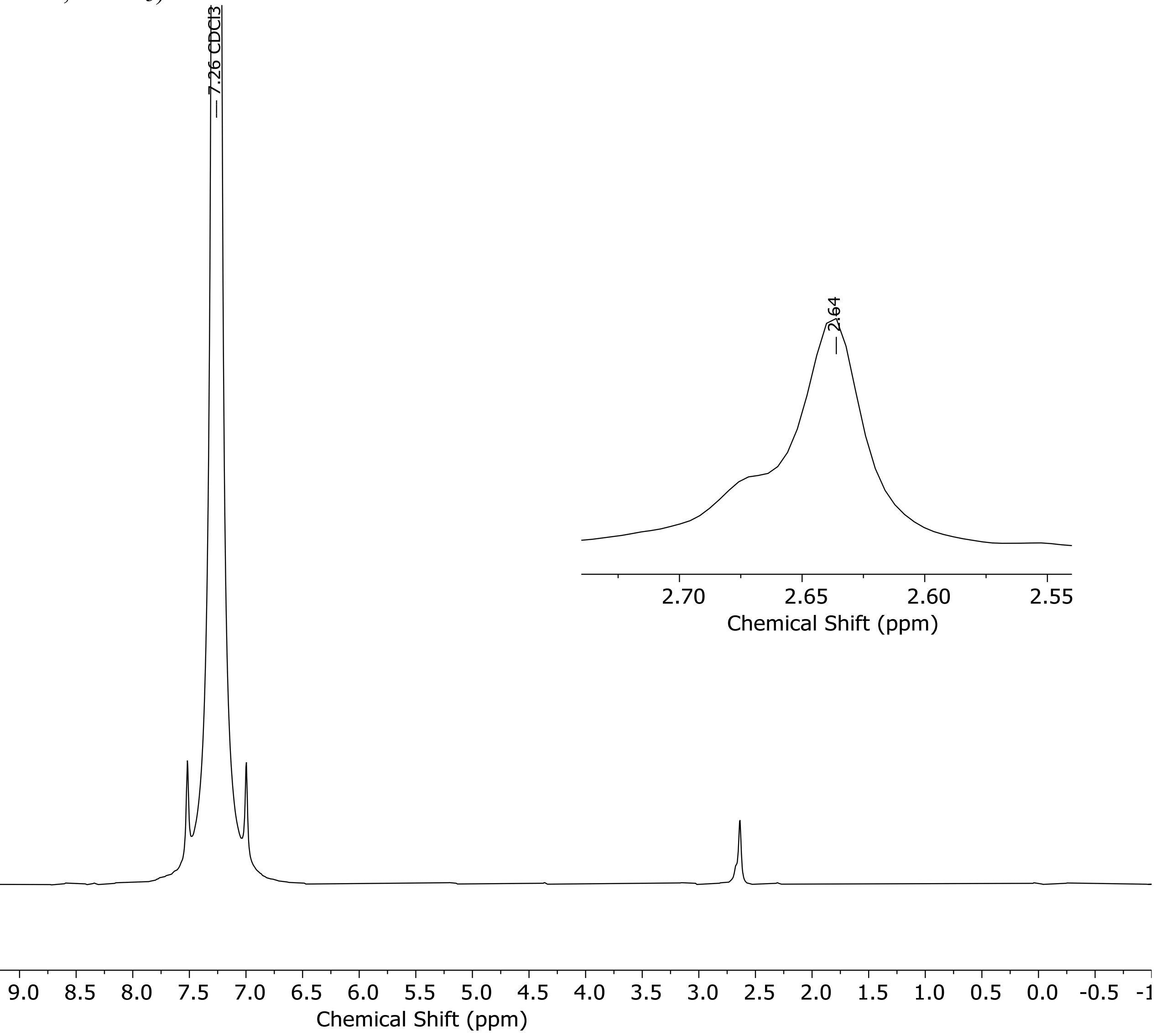

Figure S132. ^{1}H NMR (400 MHz, $CDCl_3$) of **6aa**.

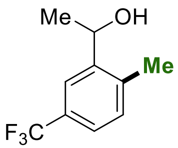

6aa

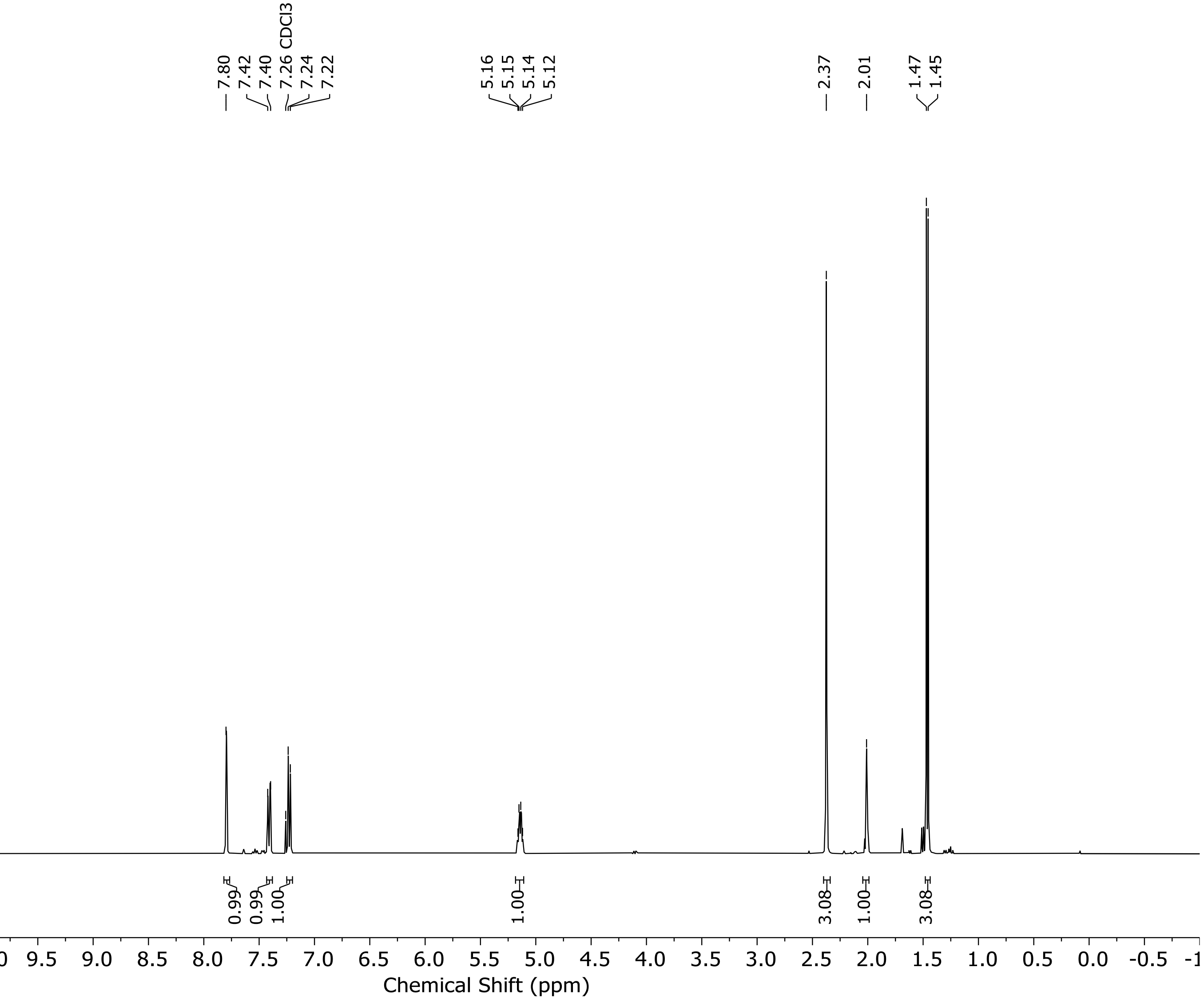

Figure S133. ^{13}C NMR (101 MHz, $CDCl_3$) of **6aa.**

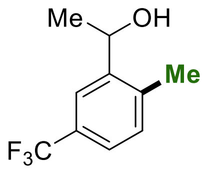

6aa

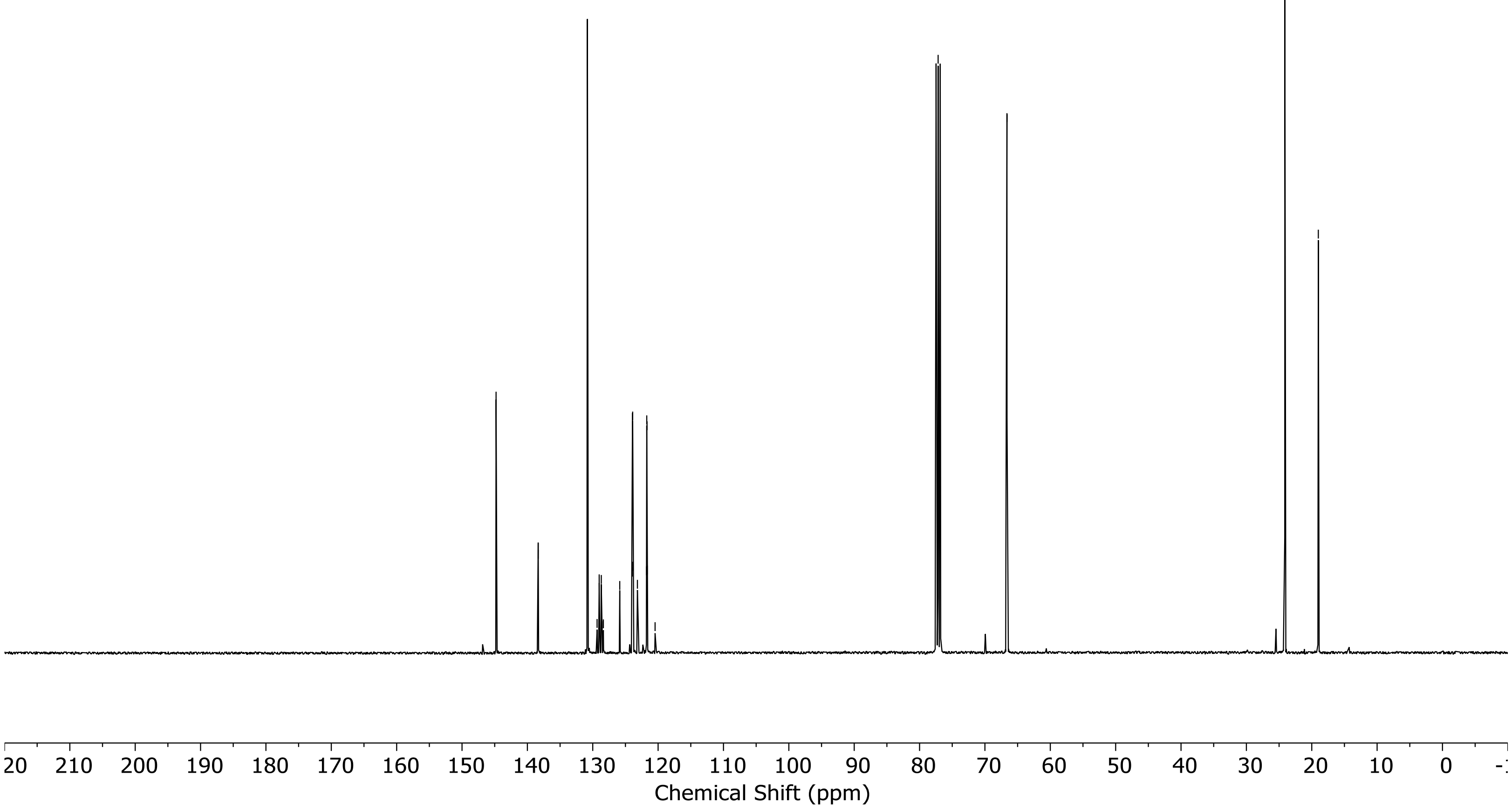

Figure S134. ^{19}F NMR (471 MHz, $CDCl_3$) of **6aa**.

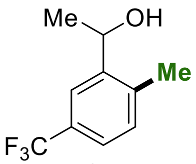

6aa

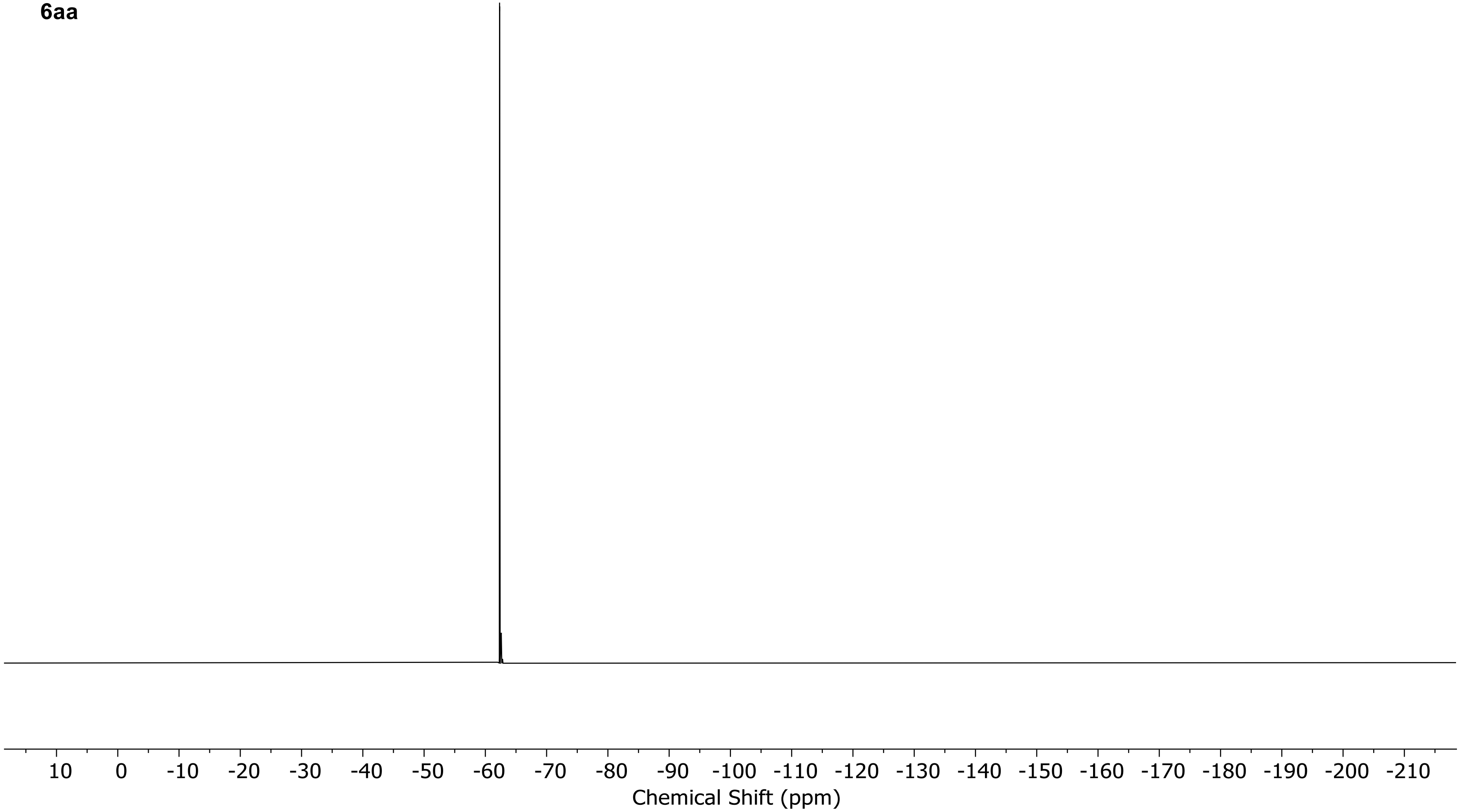

Figure S135. ^{1}H NMR (400 MHz, $CDCl_3$) of **6ab**.

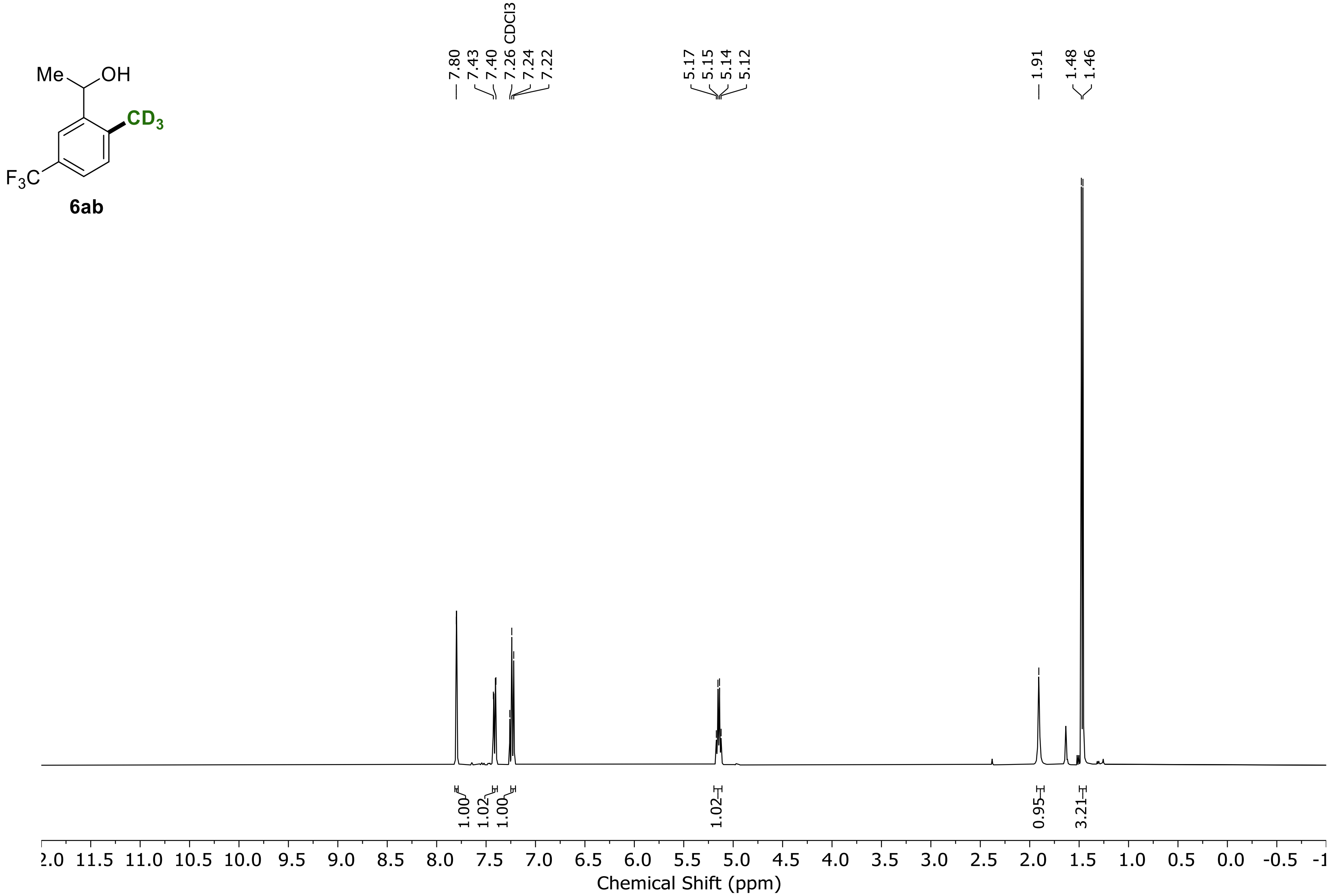

Figure S136. ^{13}C NMR (101 MHz, CDCl$_3$) of **6ab**

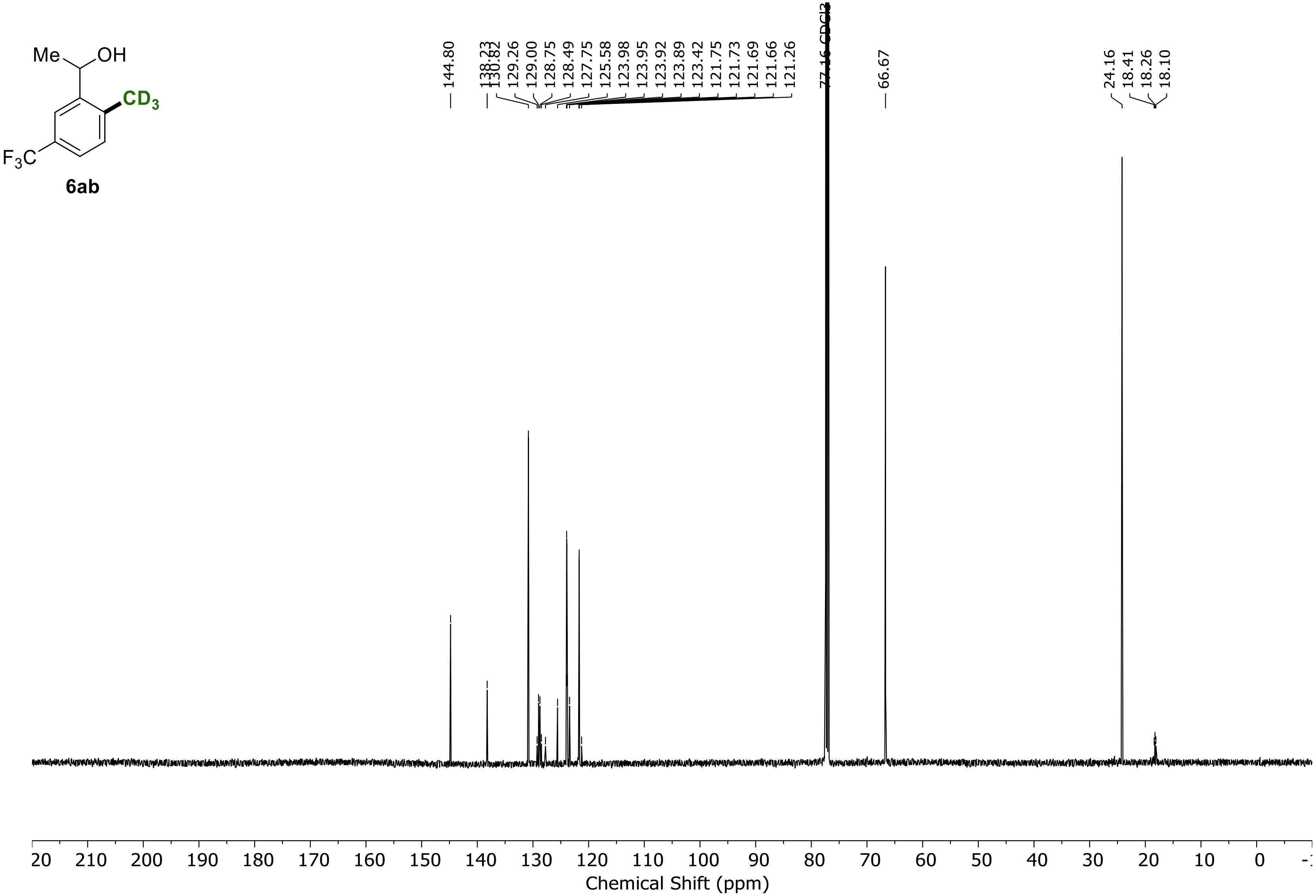

Figure S137. ^{19}F NMR (471 MHz, $CDCl_3$) of **6ab**

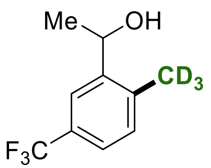

6ab

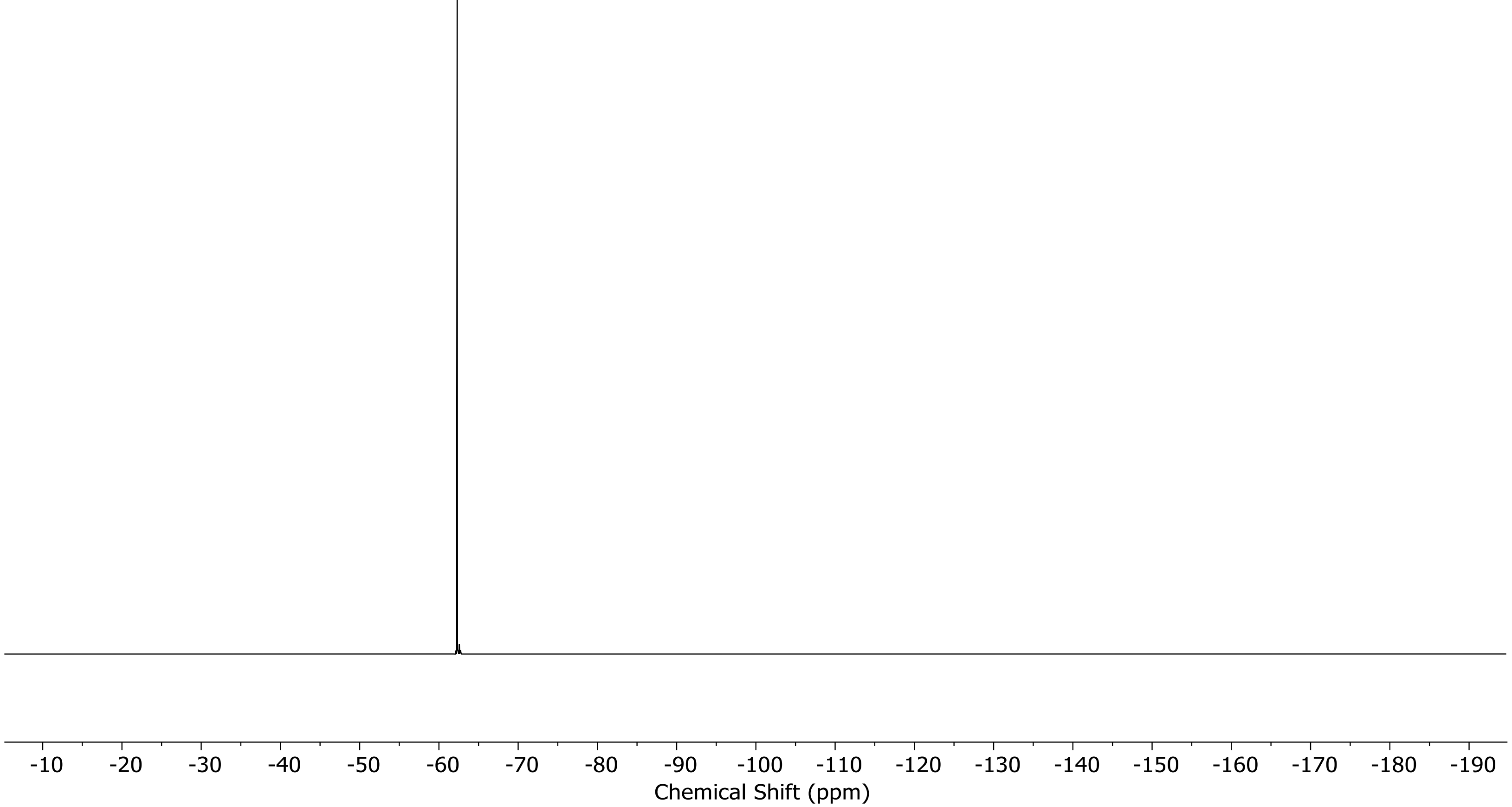

Figure S138. ^{2}H NMR (77 MHz, $CDCl_3$) of **6ab**.

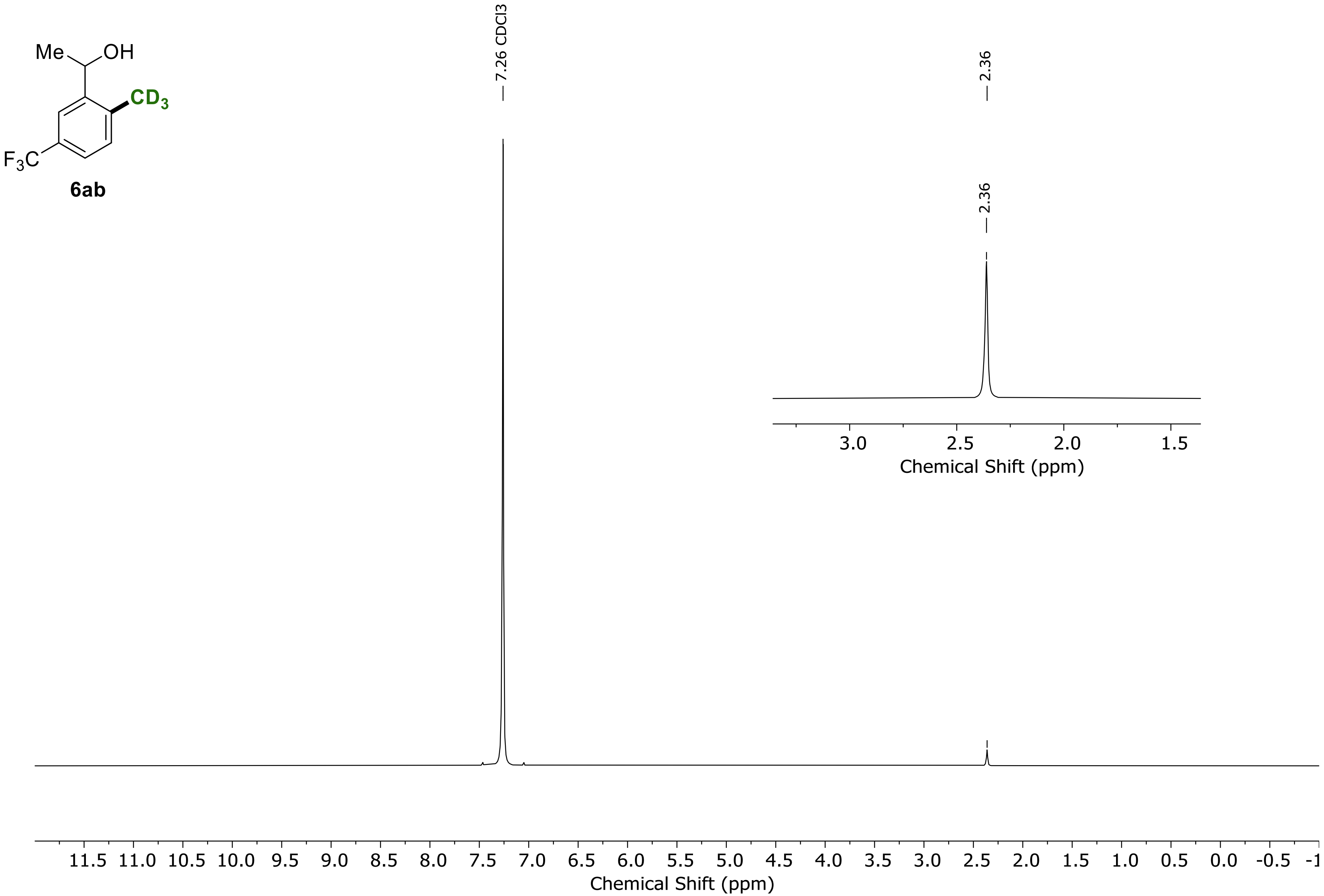

Figure S139. ^{1}H NMR (400 MHz, $CDCl_3$) of **6ba**.

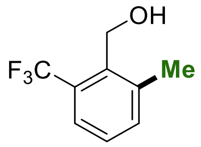

6ba

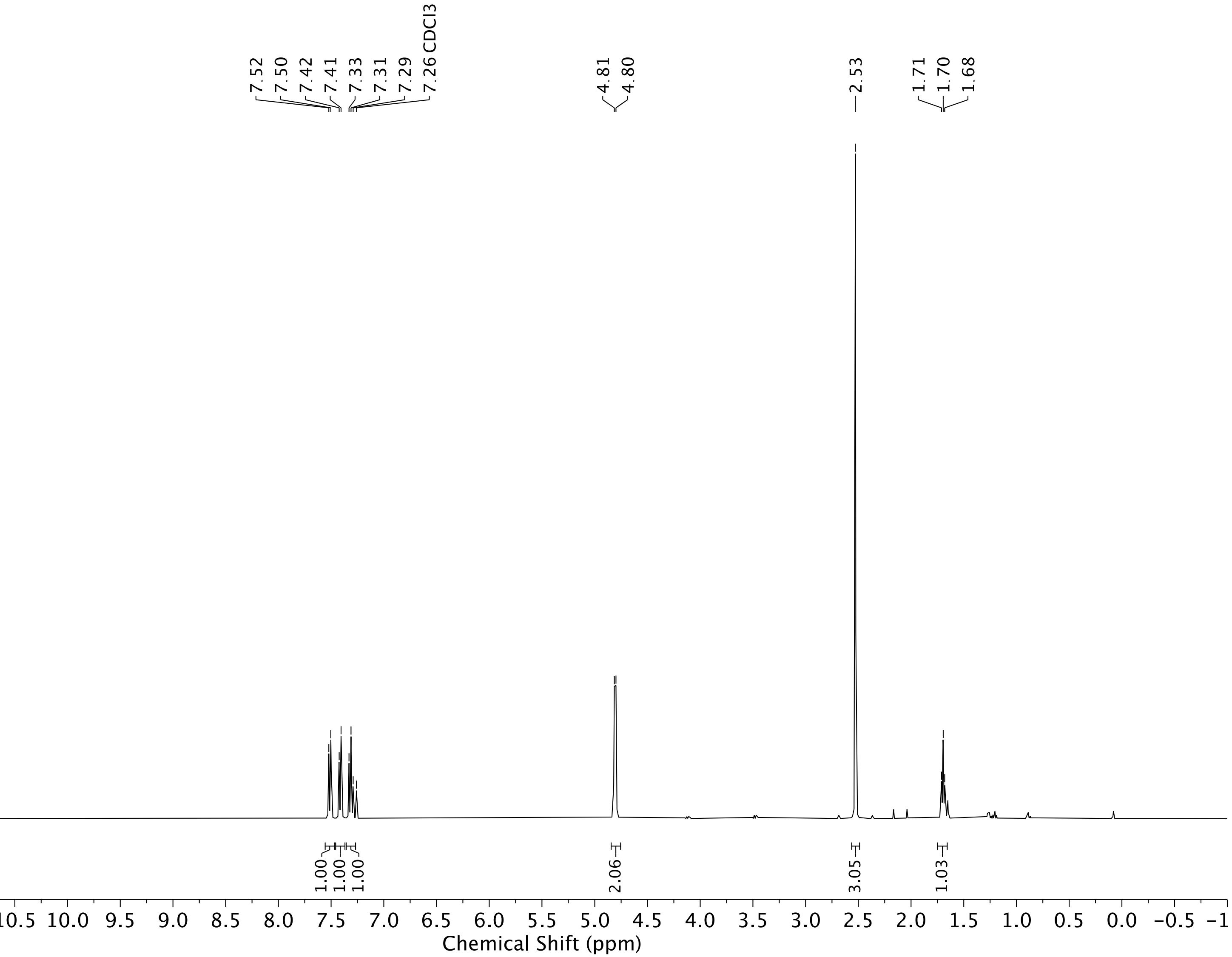

Figure S140. ^{13}C NMR (101 MHz, $CDCl_3$) of **6ba**.

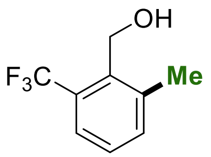

6ba

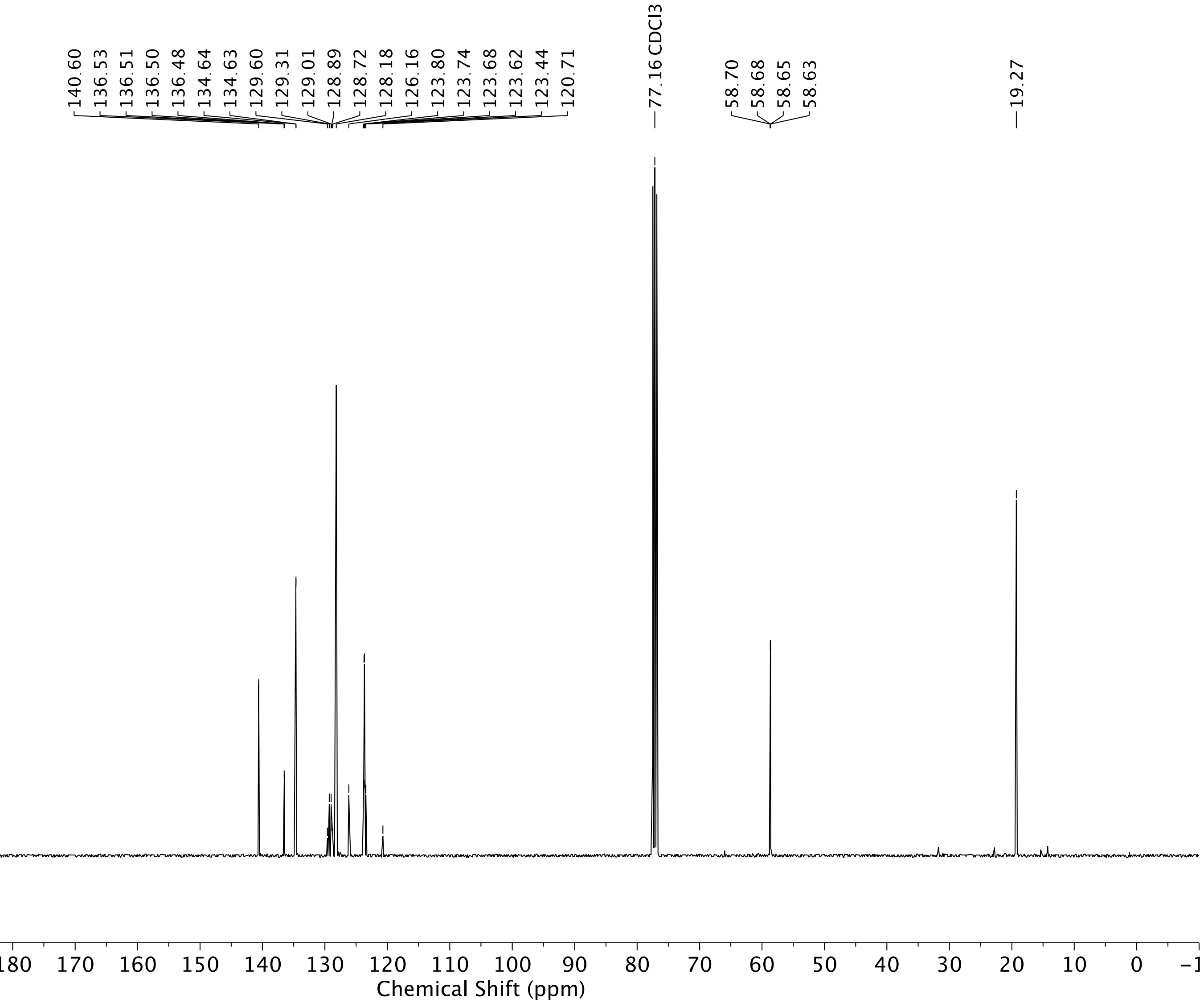

Figure S141. ^{19}F NMR (376 MHz, CDCl) of **6ba**.

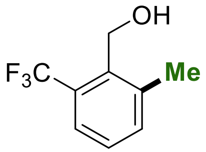

6ba

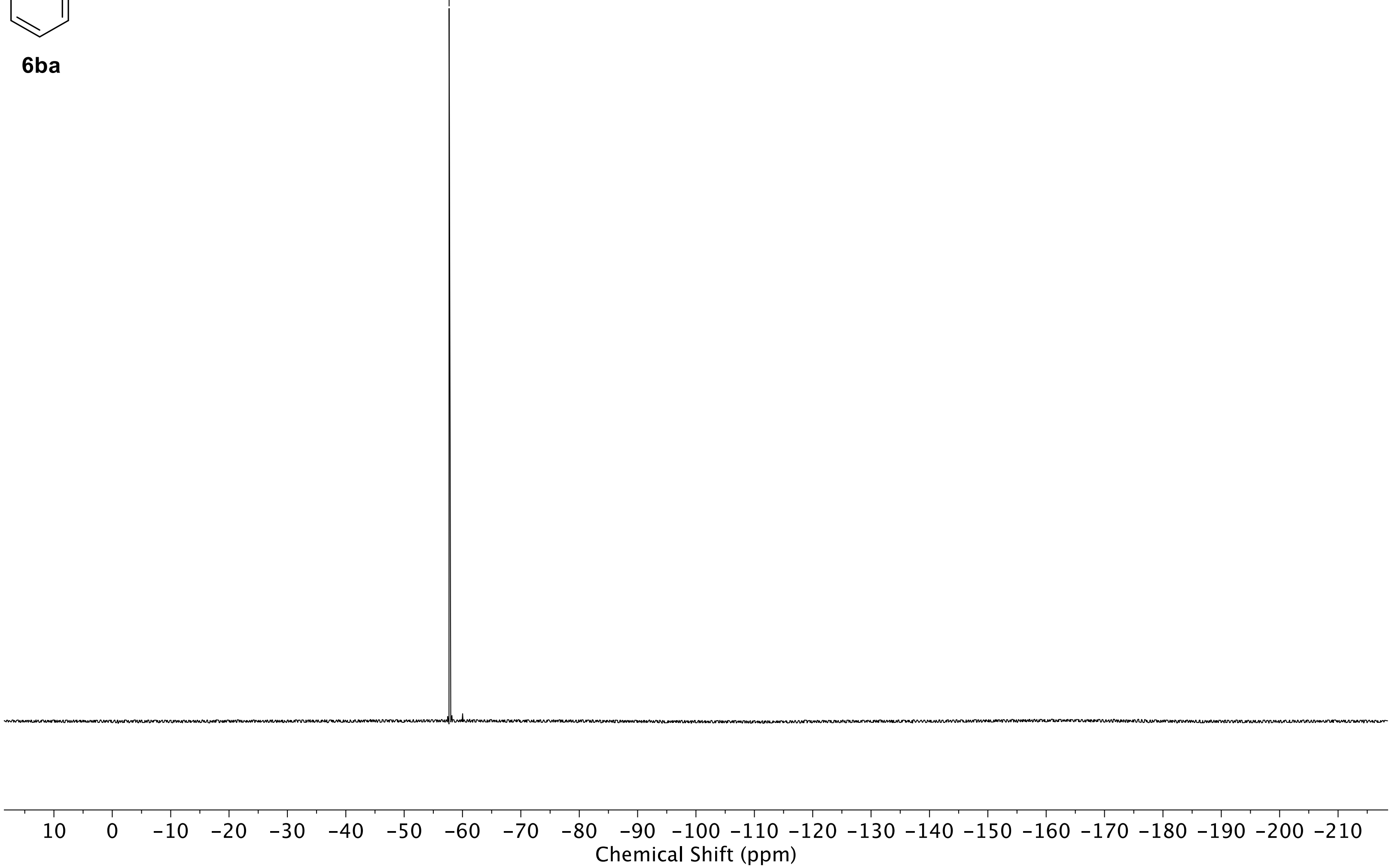

Figure S142. ^{1}H NMR (400 MHz, $CDCl_3$) of **6bb**.

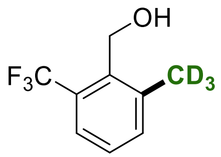

6bb

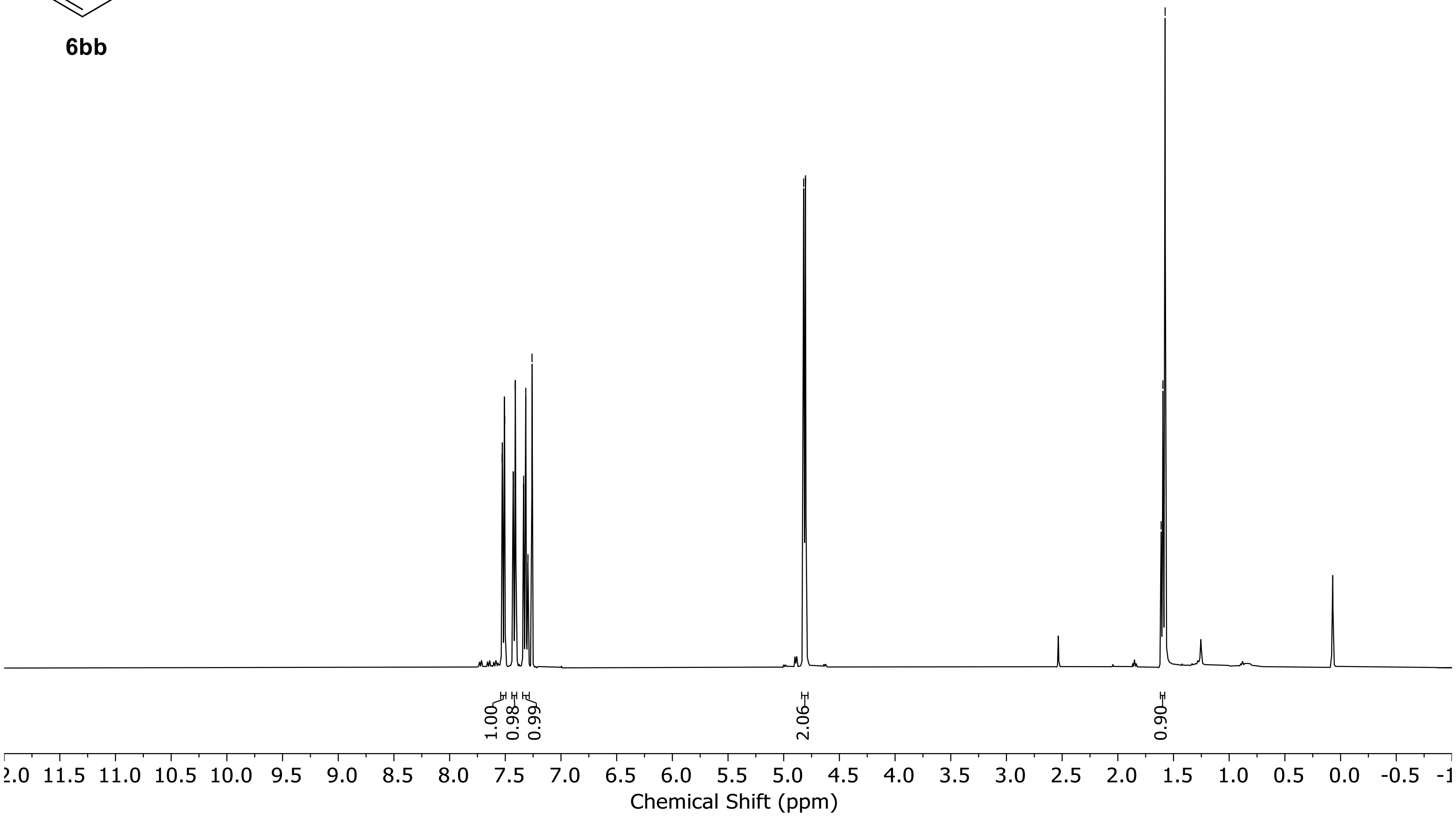

Figure S143. ^{13}C NMR (101 MHz, $CDCl_3$) of **6bb**.

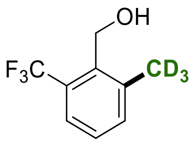

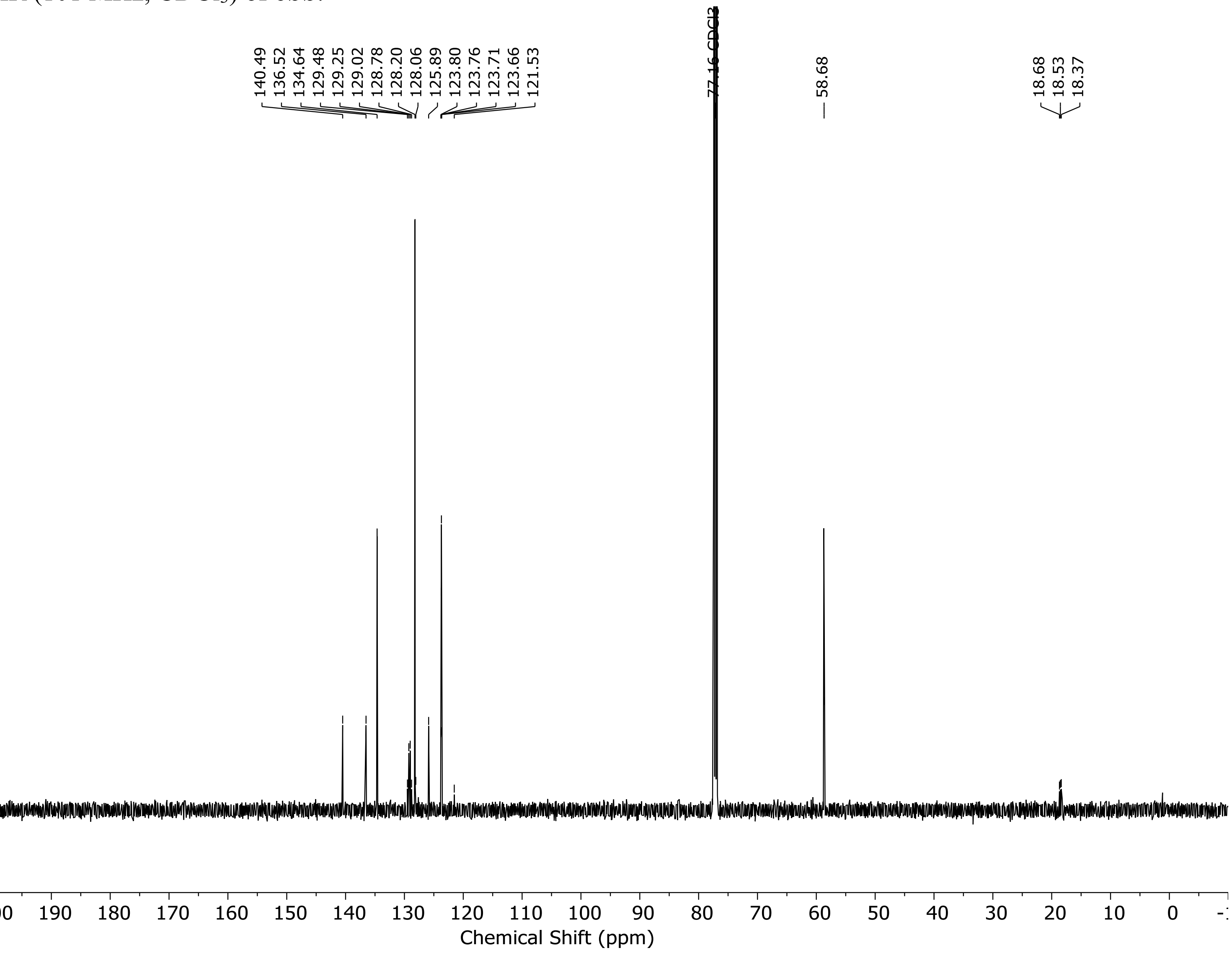

Figure S144. ^{19}F NMR (376 MHz, CDCl) of **6bb**.

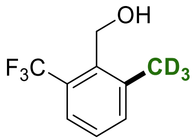

6bb

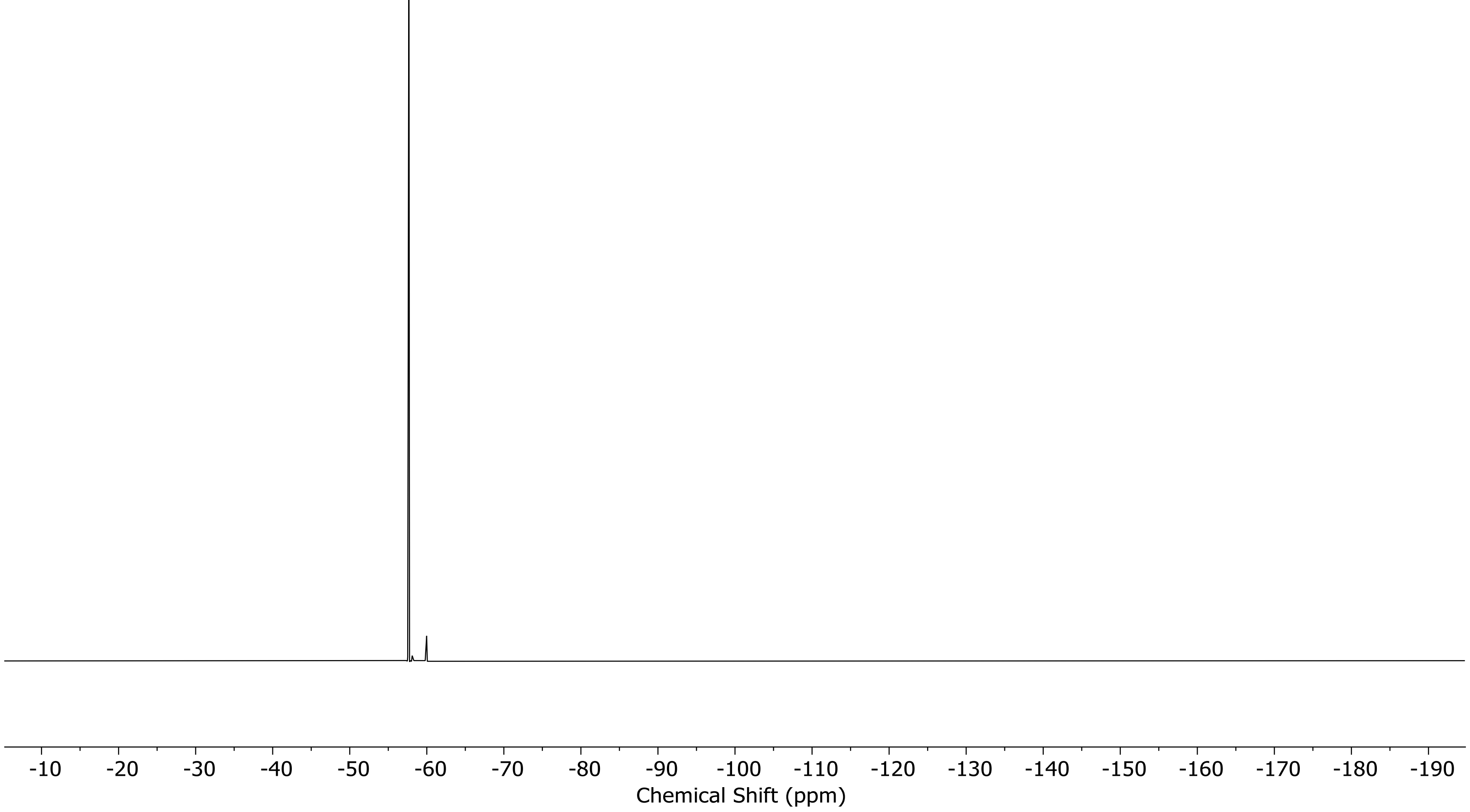

Figure S145. ^{2}H NMR (77 MHz, $CDCl_3$) of **6bb**.

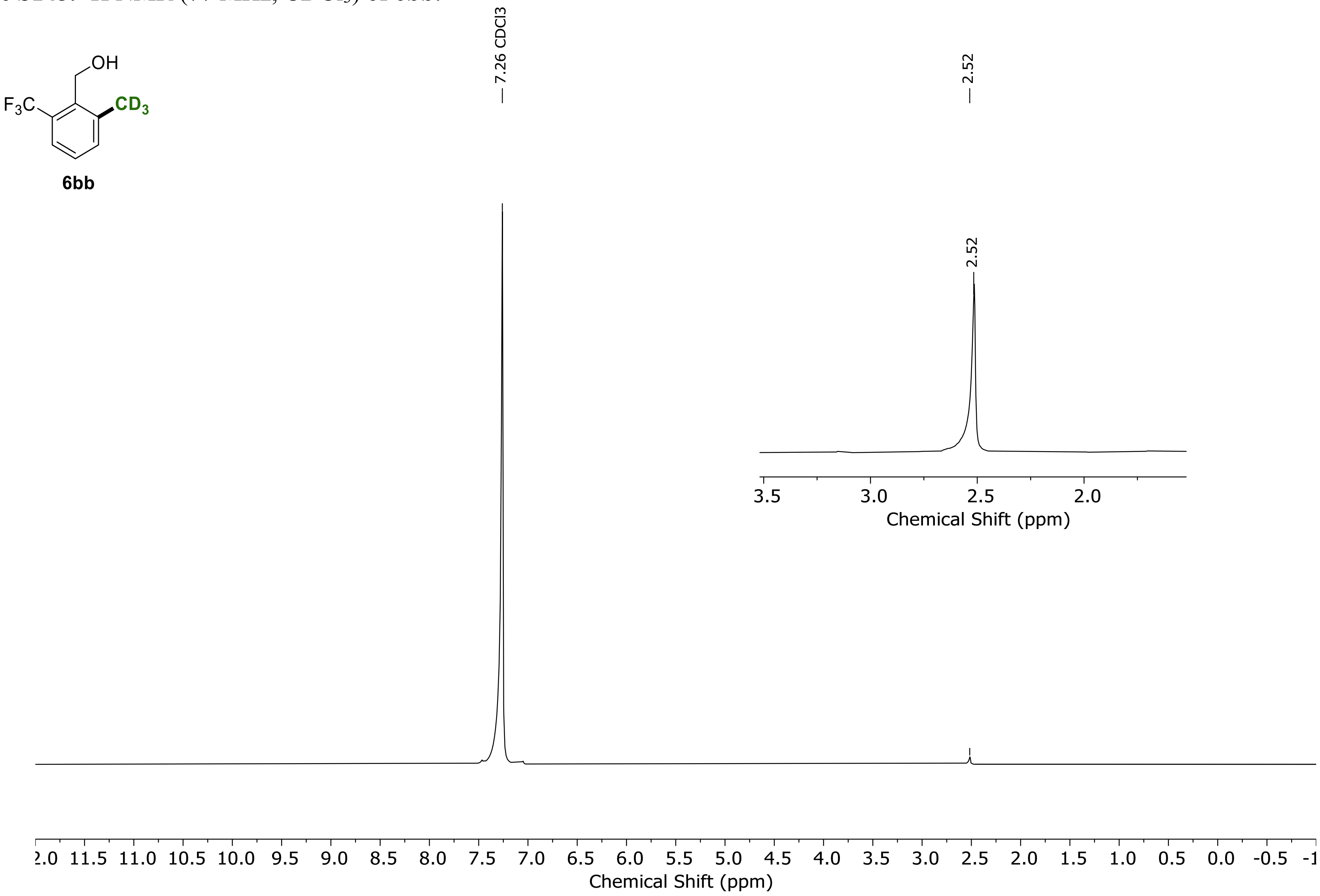

Figure S146. ^{1}H NMR (400 MHz, $CDCl_3$) of **6ca**.

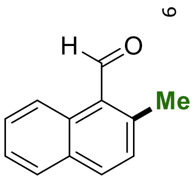

6ca

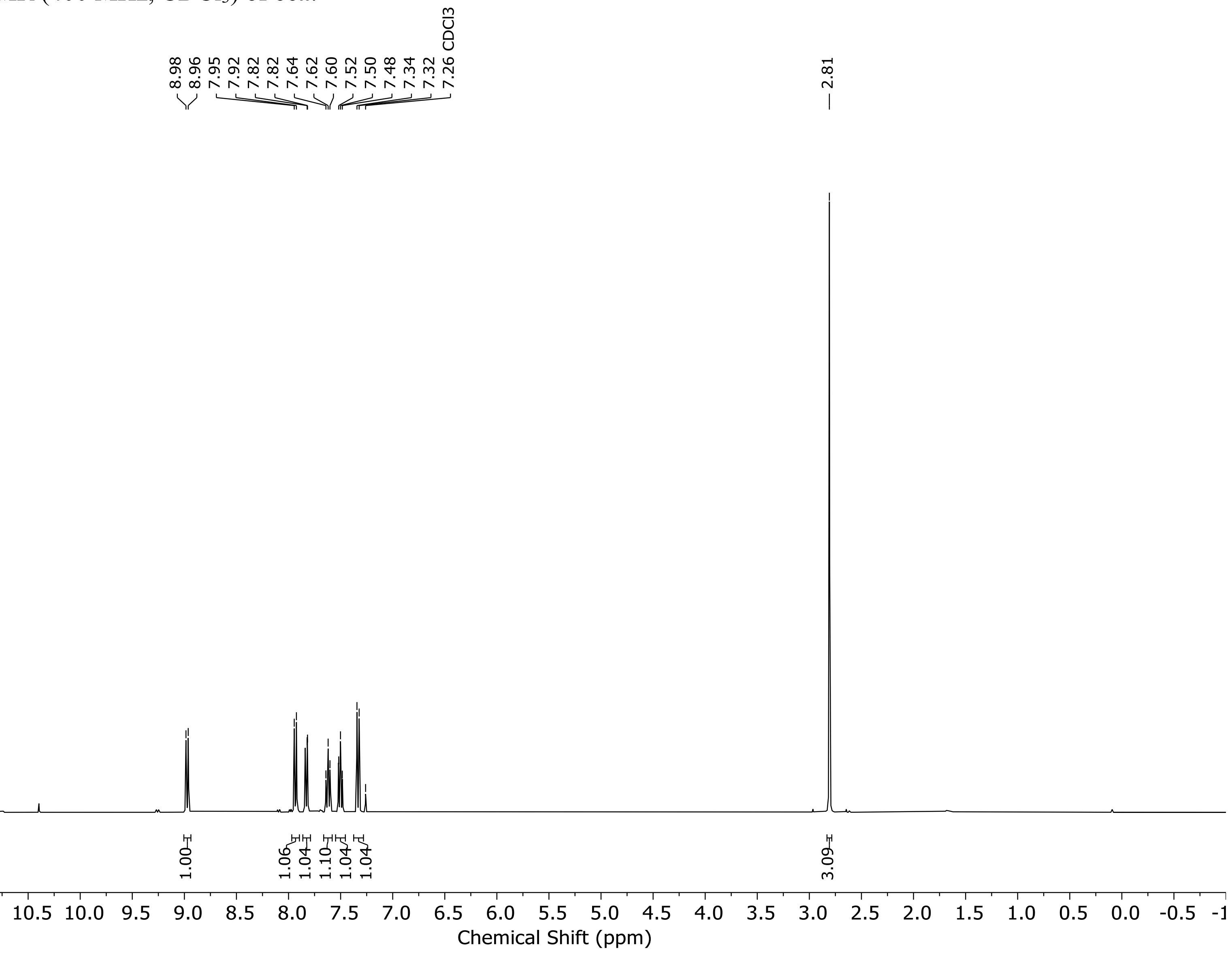

Figure S147. ^{13}C NMR (126 MHz, CDCl$_3$) of **6ca**.

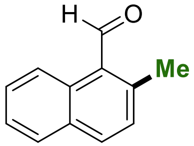

6ca

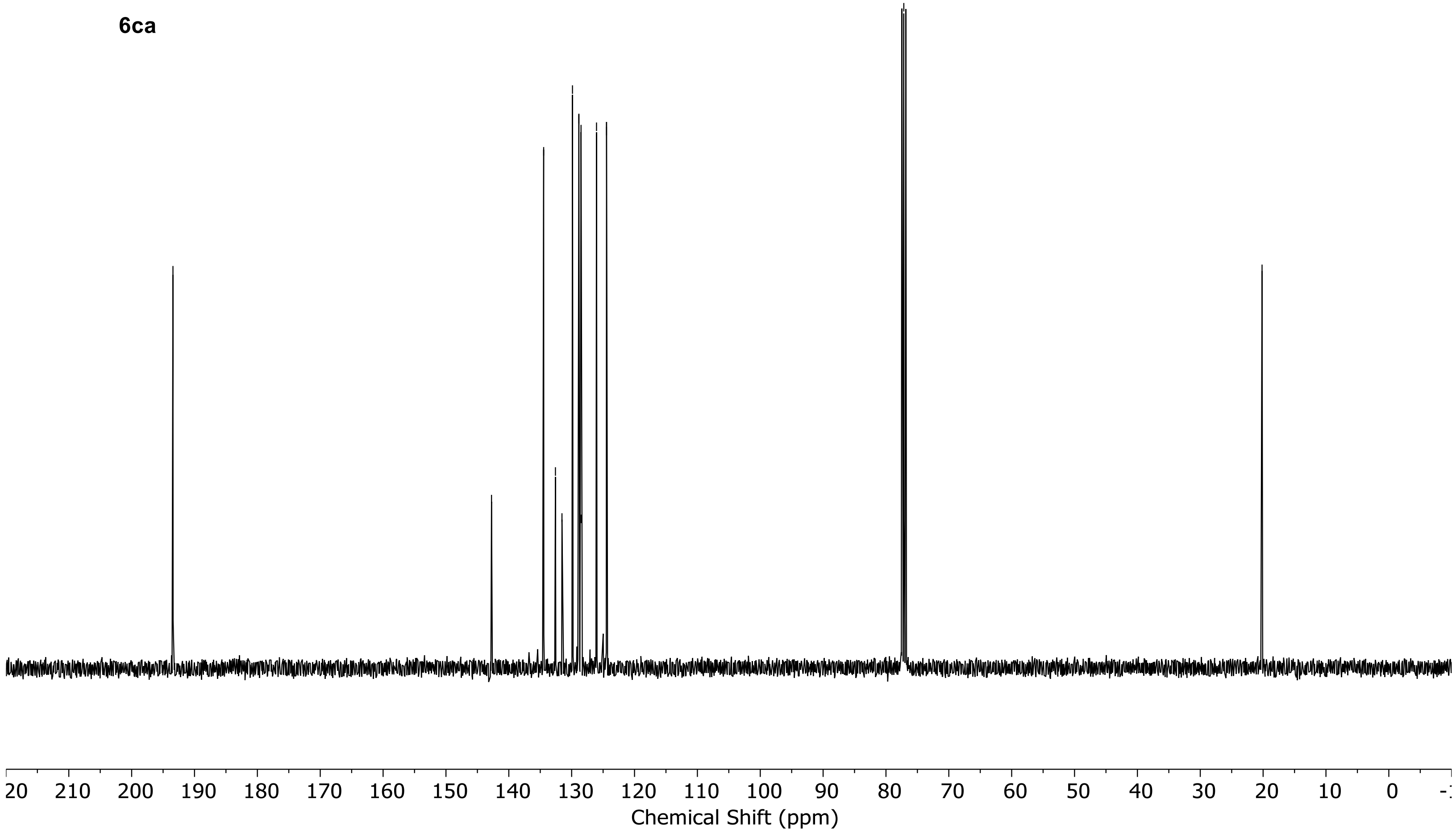

Figure S148. ^{1}H NMR (400 MHz, $CDCl_3$) of **8aa**.

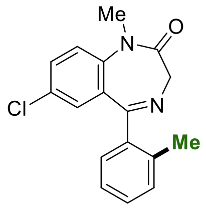

8aa

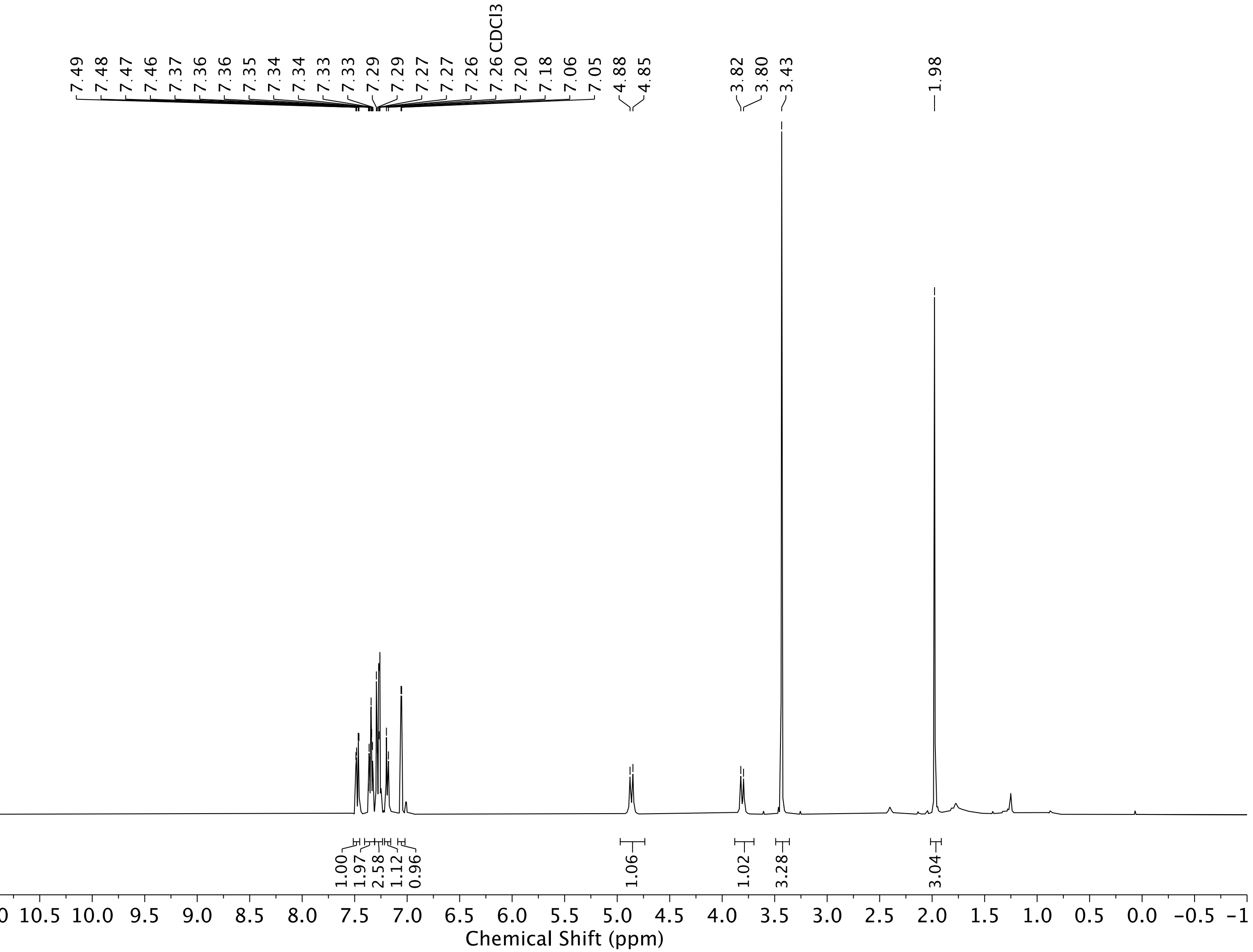

Figure S149. ^{13}C NMR (126 MHz, $CDCl_3$) of **8aa**.

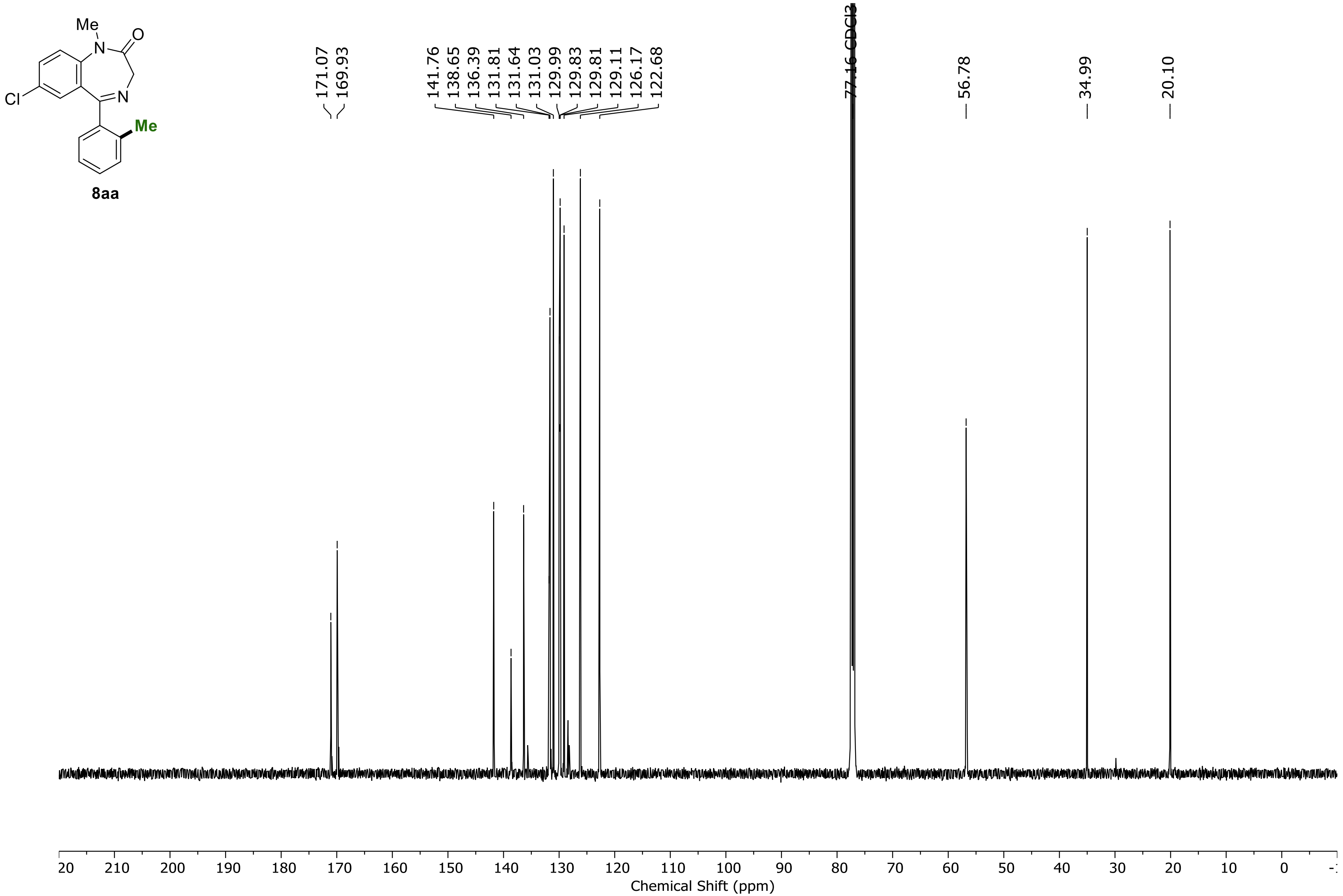

Figure S150. ^{1}H NMR (400 MHz, $CDCl_3$) of **8ab**.

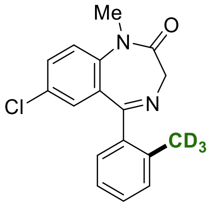

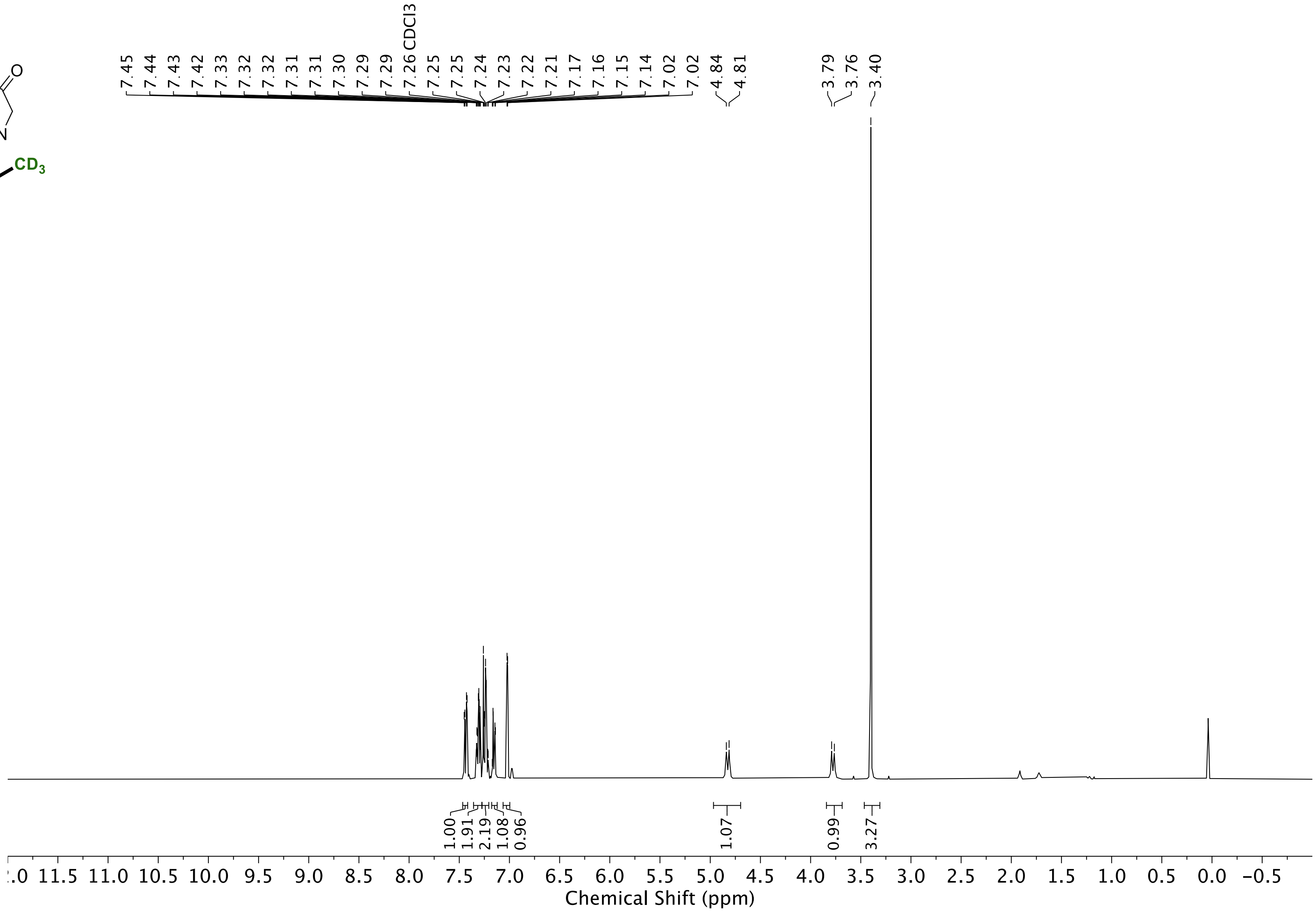

Figure S151. ^{13}C NMR (101 MHz, $CDCl_3$) of **8ab**.

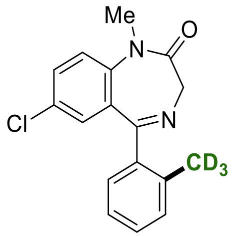

8ab

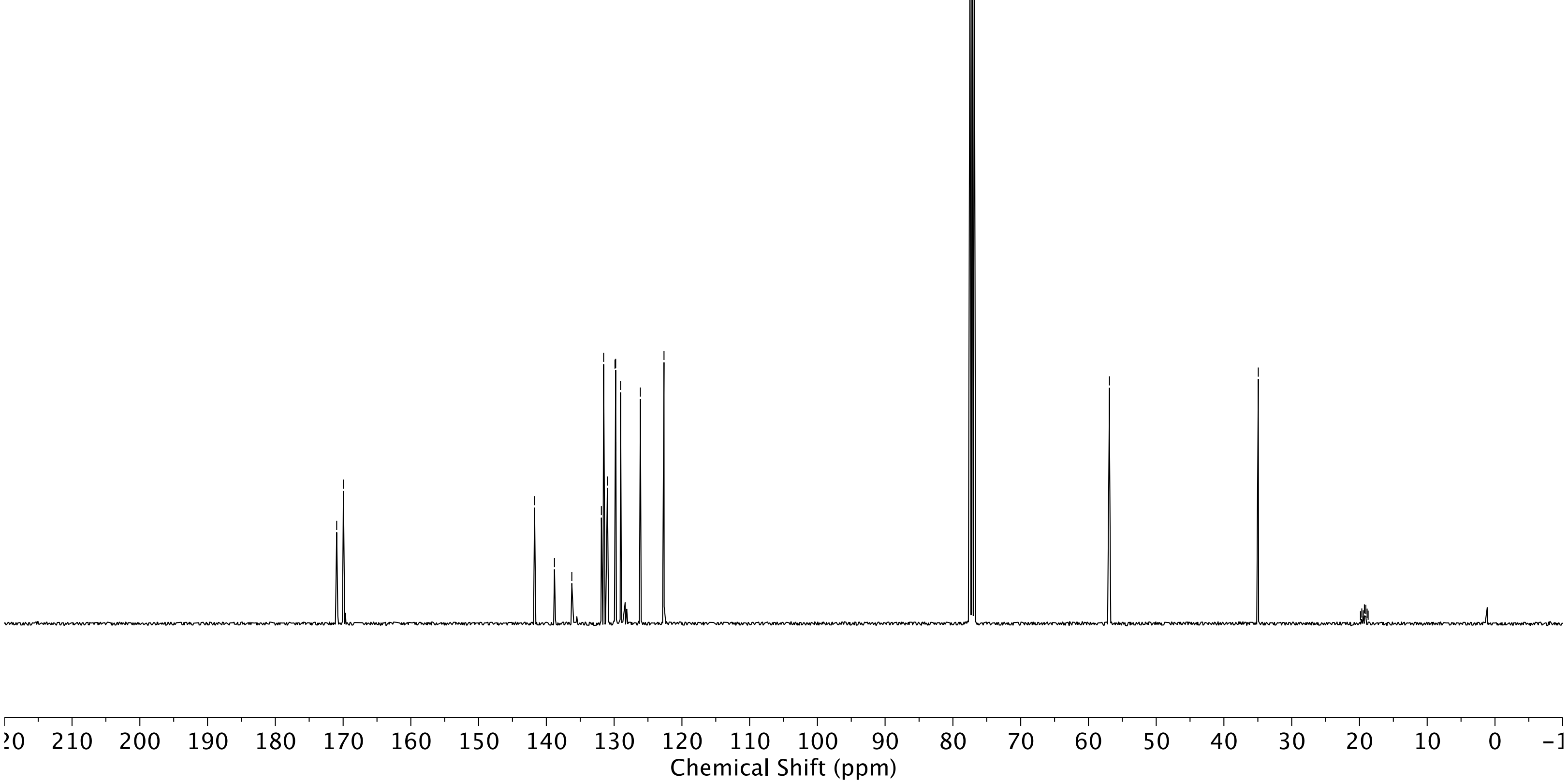

Figure S152. ^{2}H NMR (61 MHz, $CDCl_3$) of **8ab**.

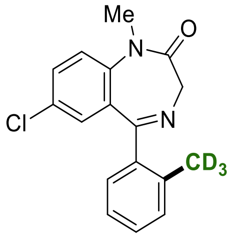

8ab

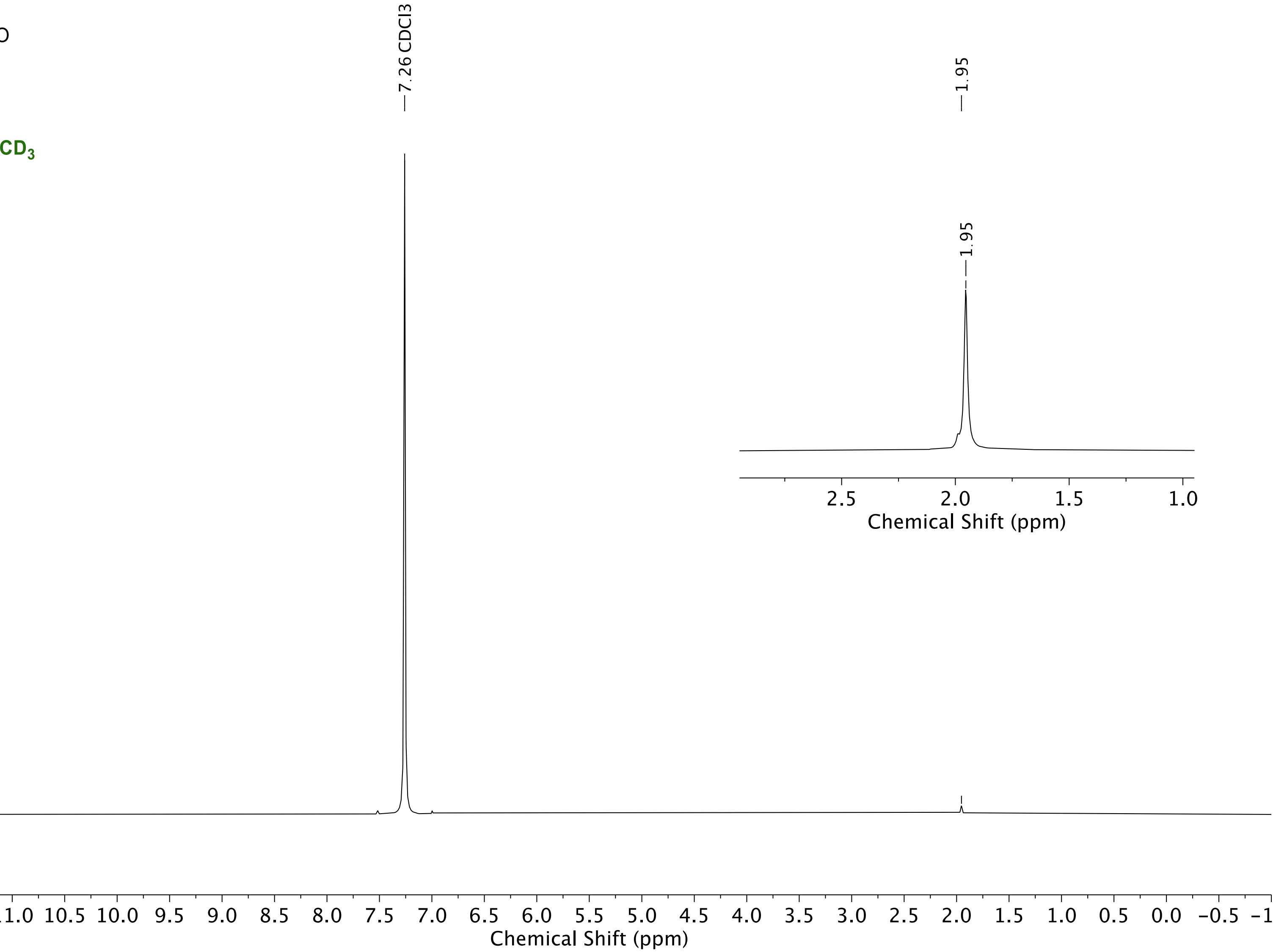

Figure S153. ^{1}H NMR (400 MHz, $CDCl_3$) of **8ba**.

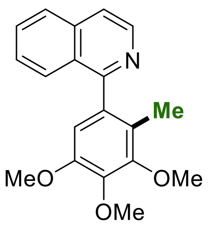

8ba

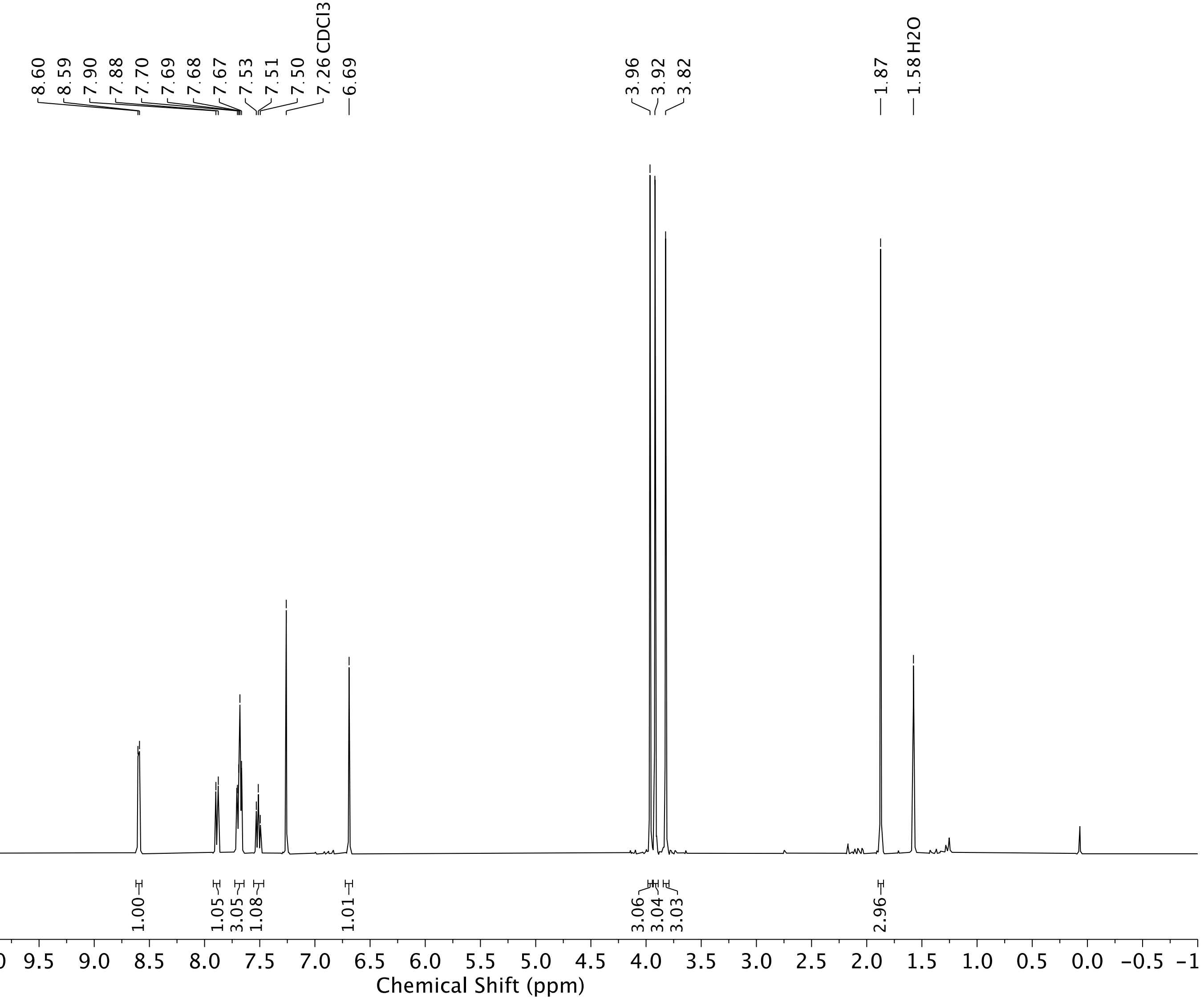

Figure S154. ^{13}C NMR (101 MHz, $CDCl_3$) of **8ba**.

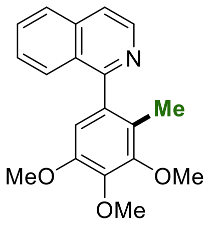

8ba

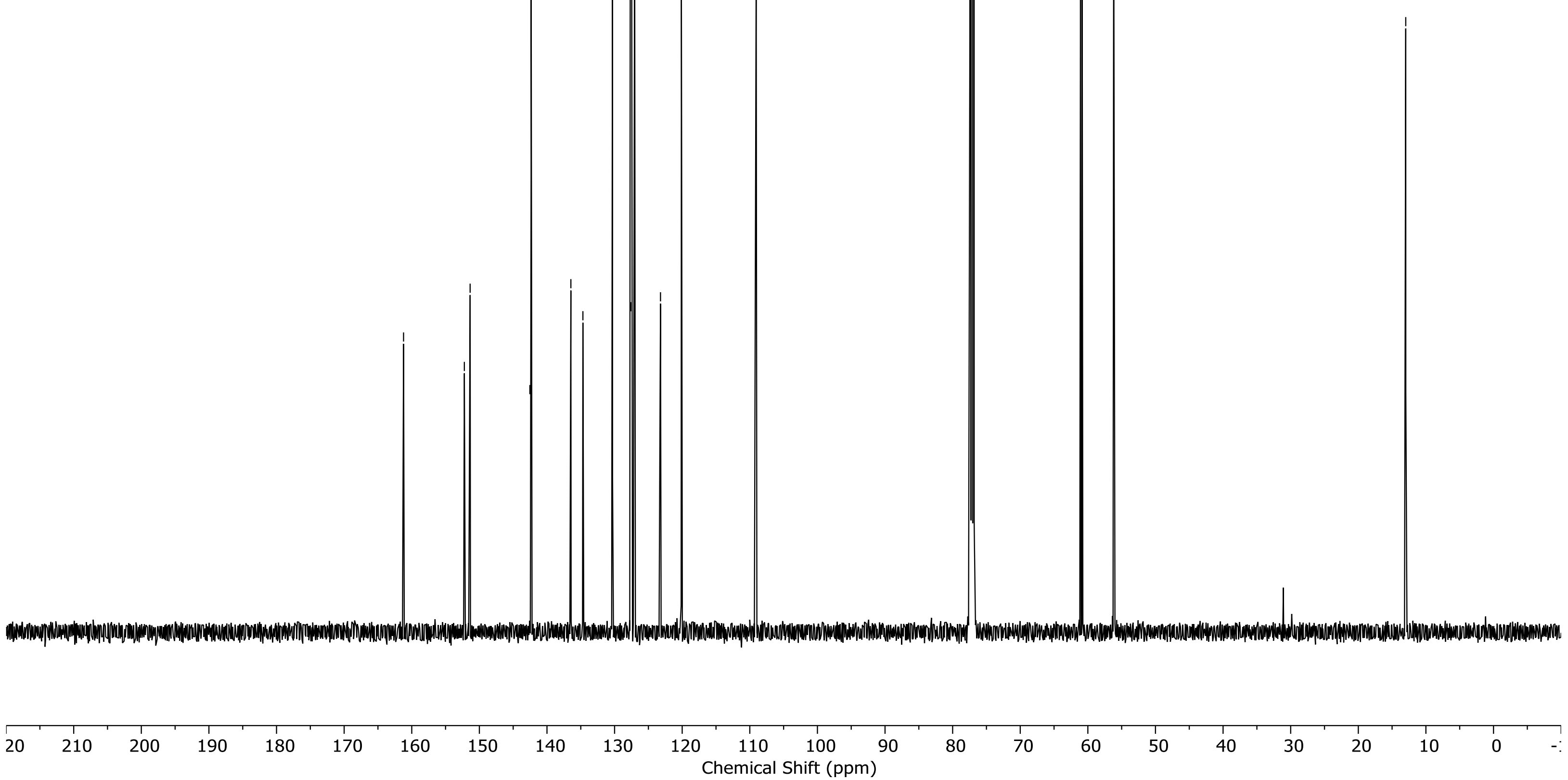

Figure S155. ^{1}H NMR (400 MHz, $CDCl_3$) of **8ca**.

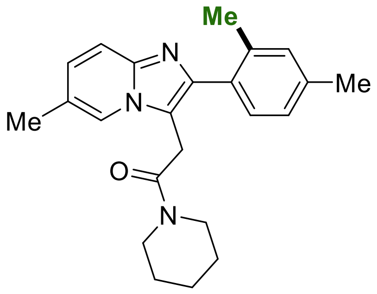

8ca

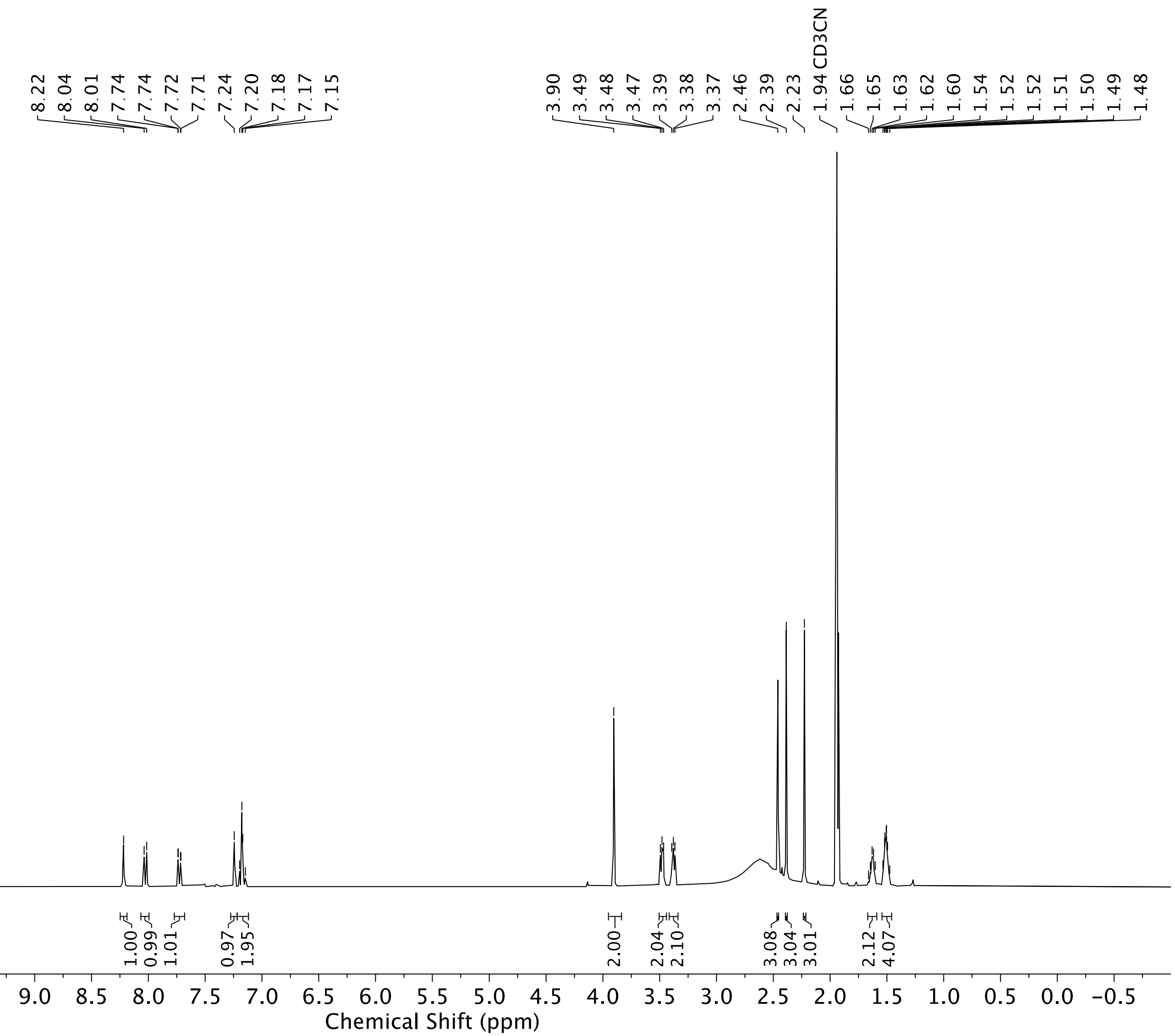

Figure S156. ^{13}C NMR (101 MHz, $CDCl_3$) of **8ca**.

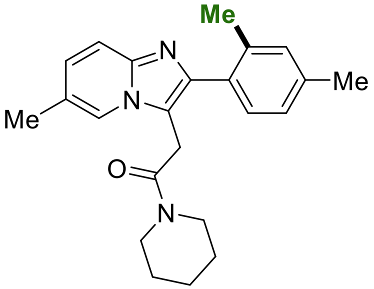

8ca

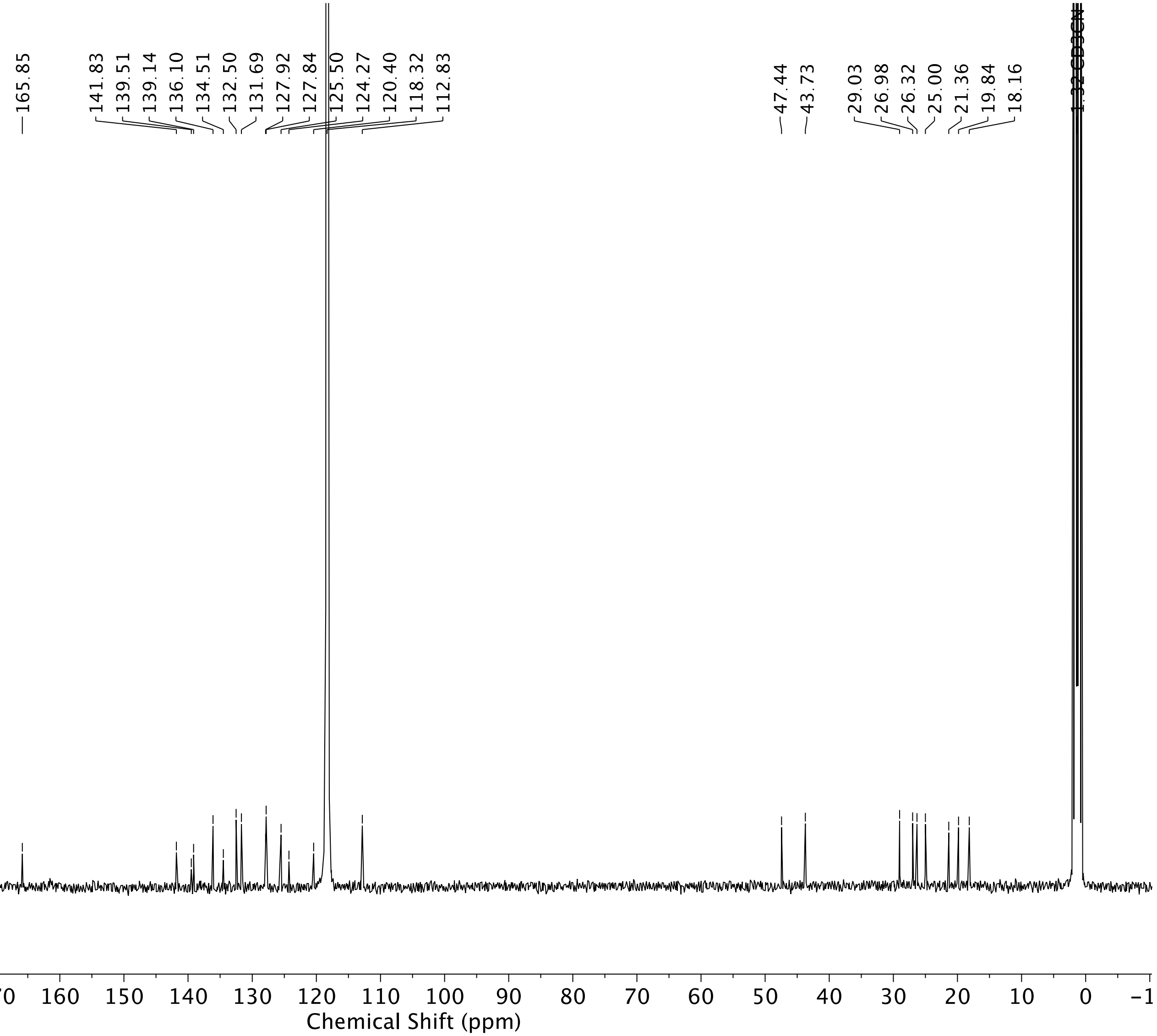

Figure S157. ^{1}H NMR (400 MHz, $CDCl_3$) of **8da** and **9da** product mixture.

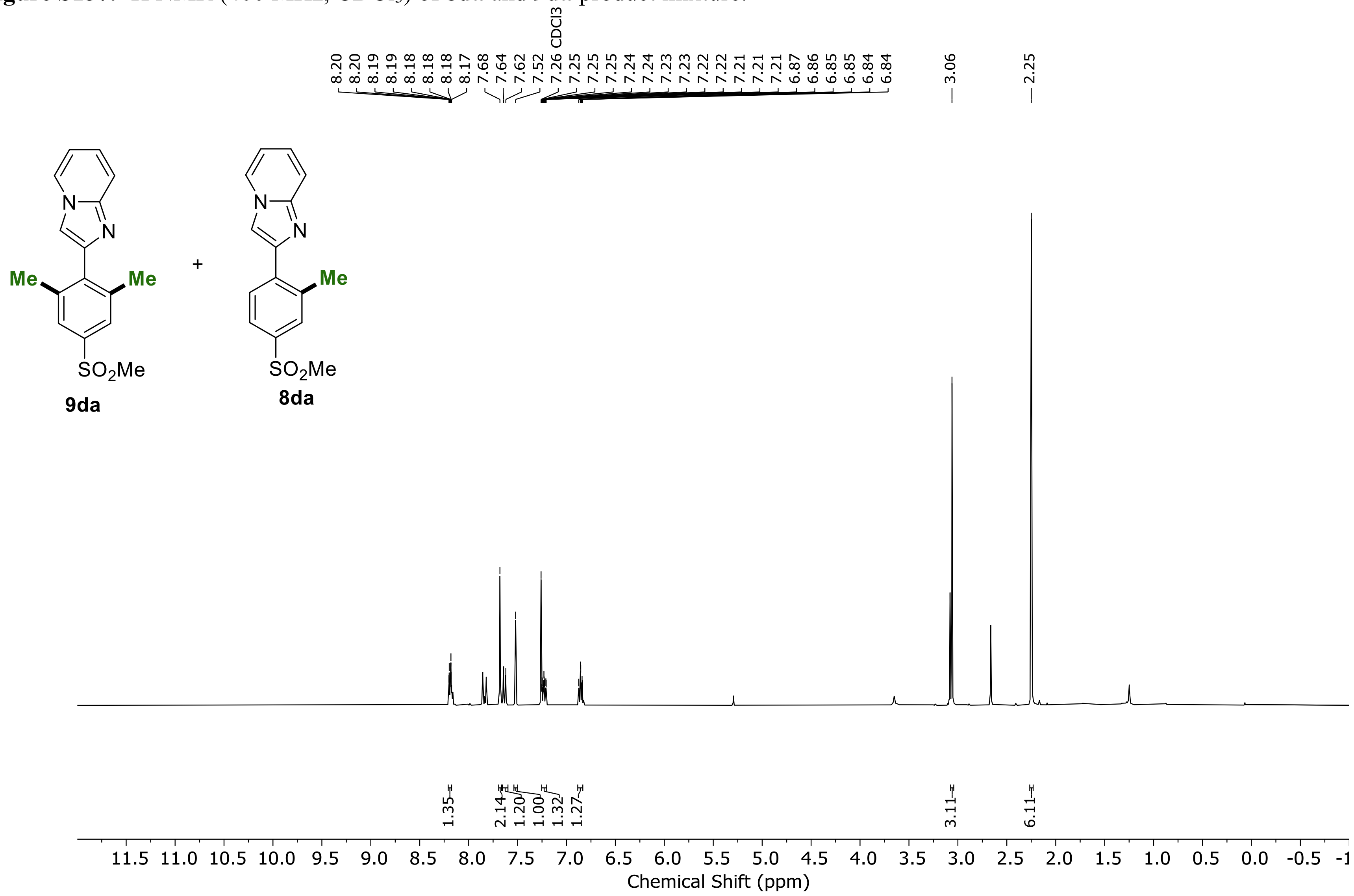

Figure S158. ^{13}C NMR (101 MHz, $CDCl_3$) of **8da**.and **9da** product mixture.

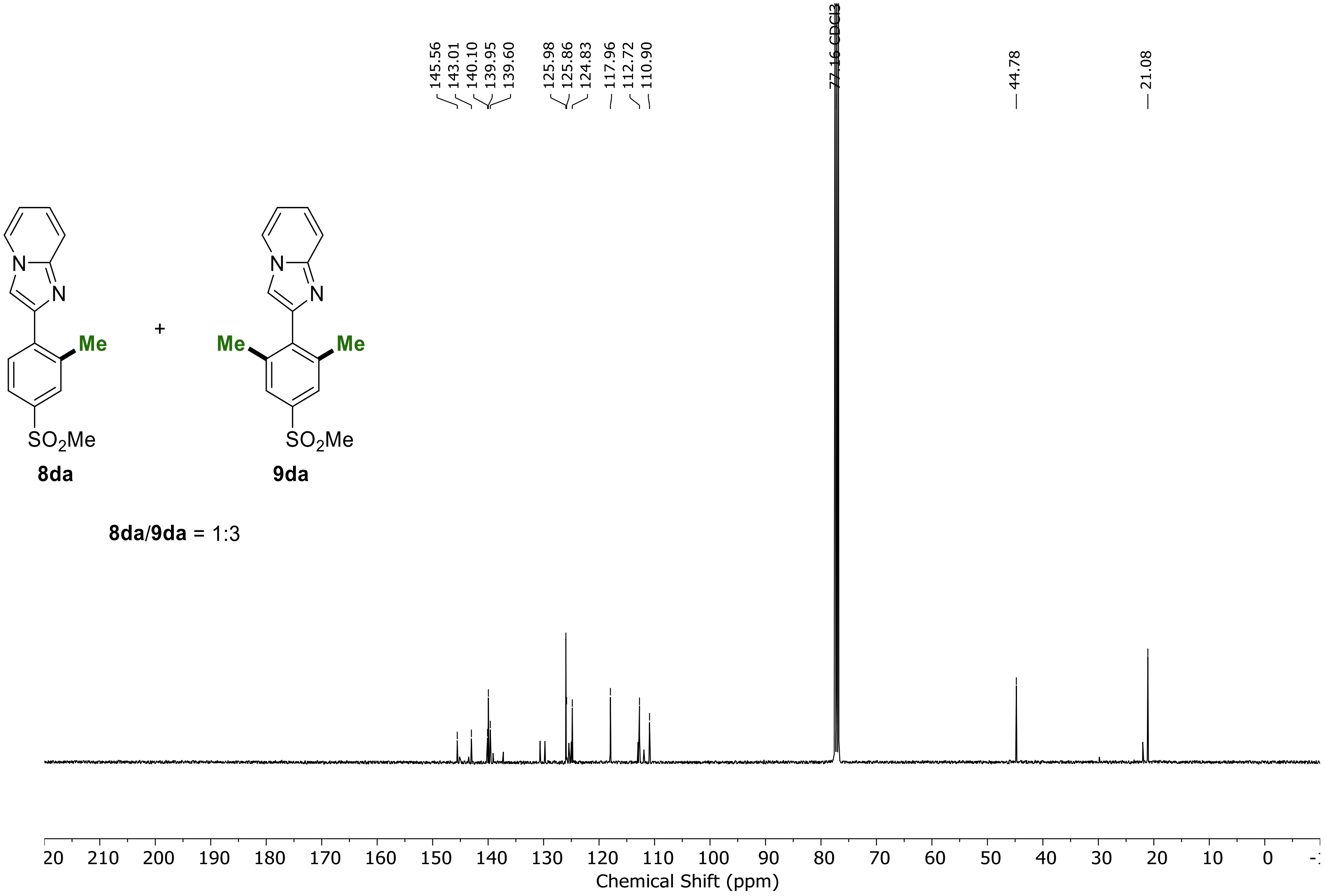

Figure S159. COSY NMR ($CDCl_3$) of **8da**.and **9da** product mixture.

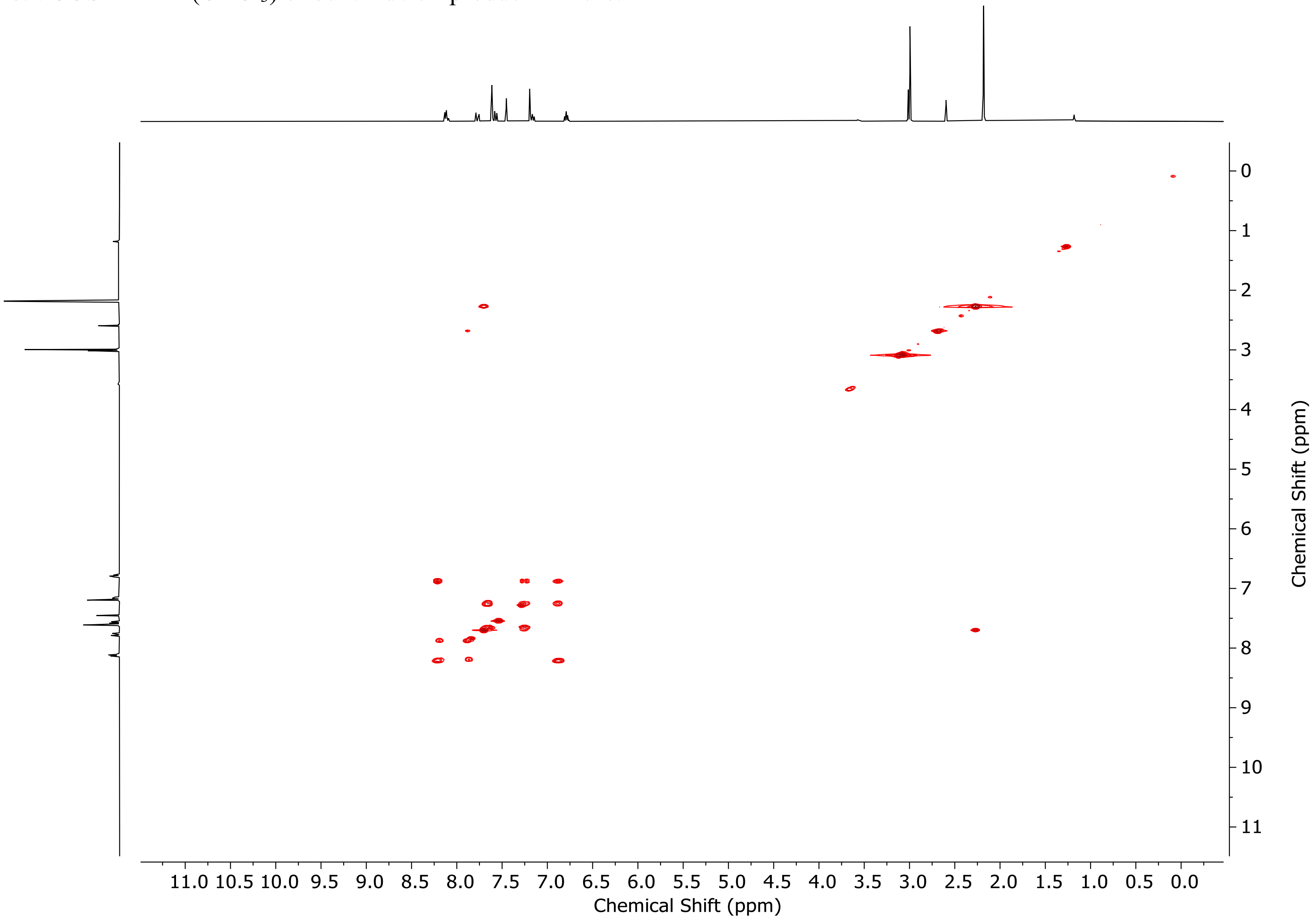

Figure S160. HMBC NMR ($CDCl_3$) of **8da**.and **9da** product mixture.

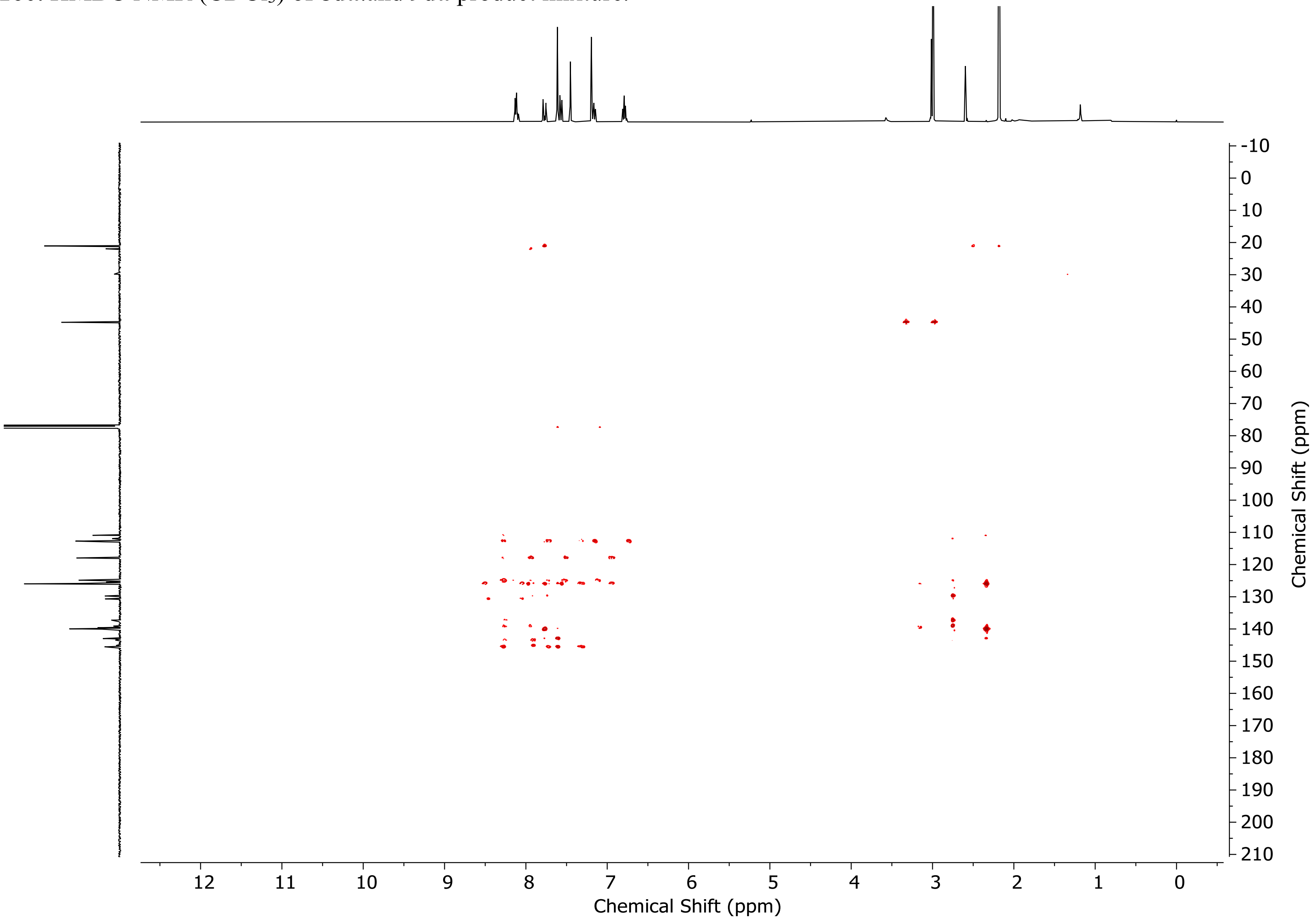

Figure S161. HSQC NMR ($CDCl_3$) of **8da**.and **9da** product mixture.

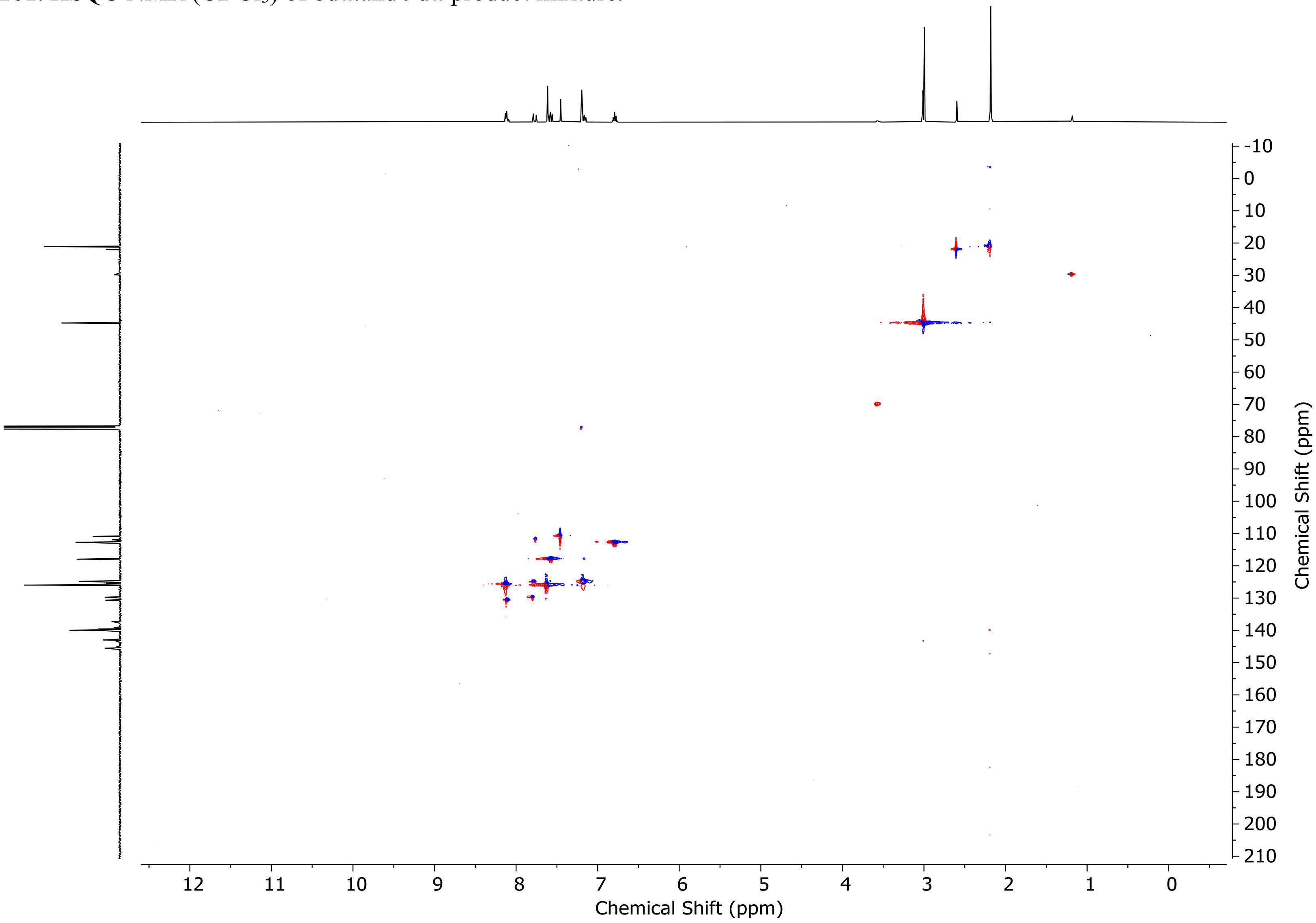

Figure S162. ^{1}H NMR (400 MHz, $CDCl_3$) of **8ea**.

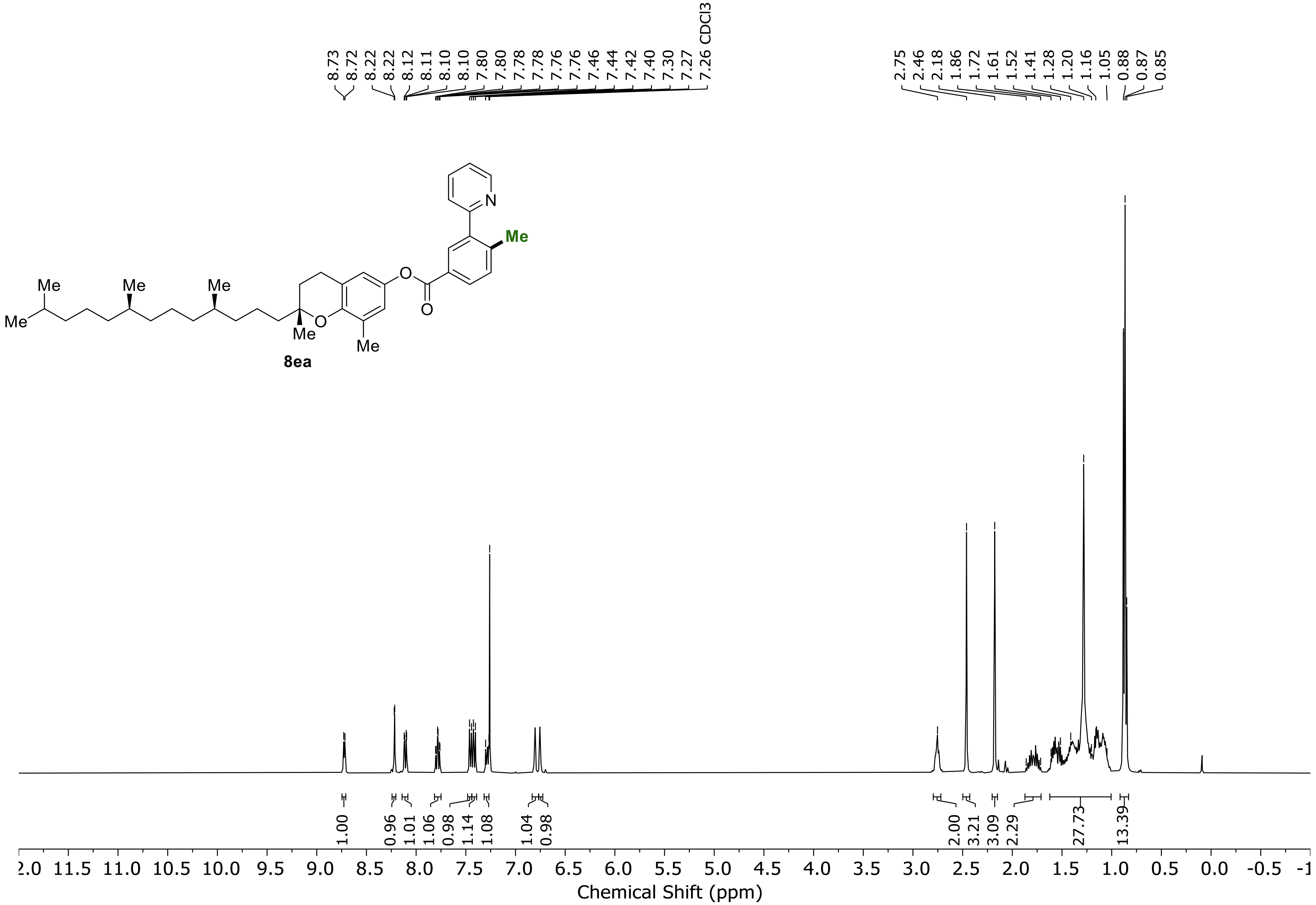

Figure S163. ^{13}C NMR (101 MHz, $CDCl_3$) of **8ea**

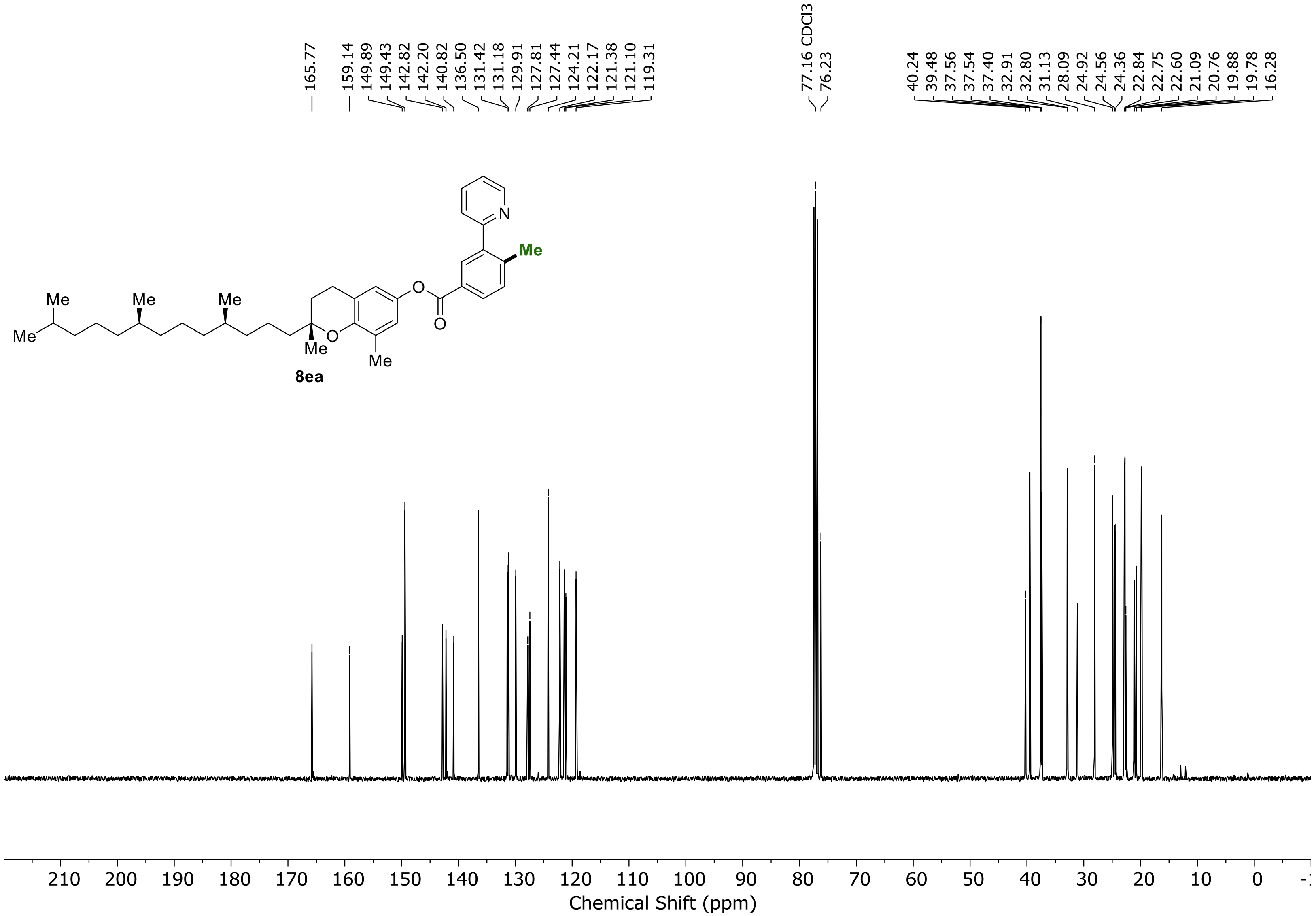

Figure S164. COSY NMR ($CDCl_3$) of **8ea**.

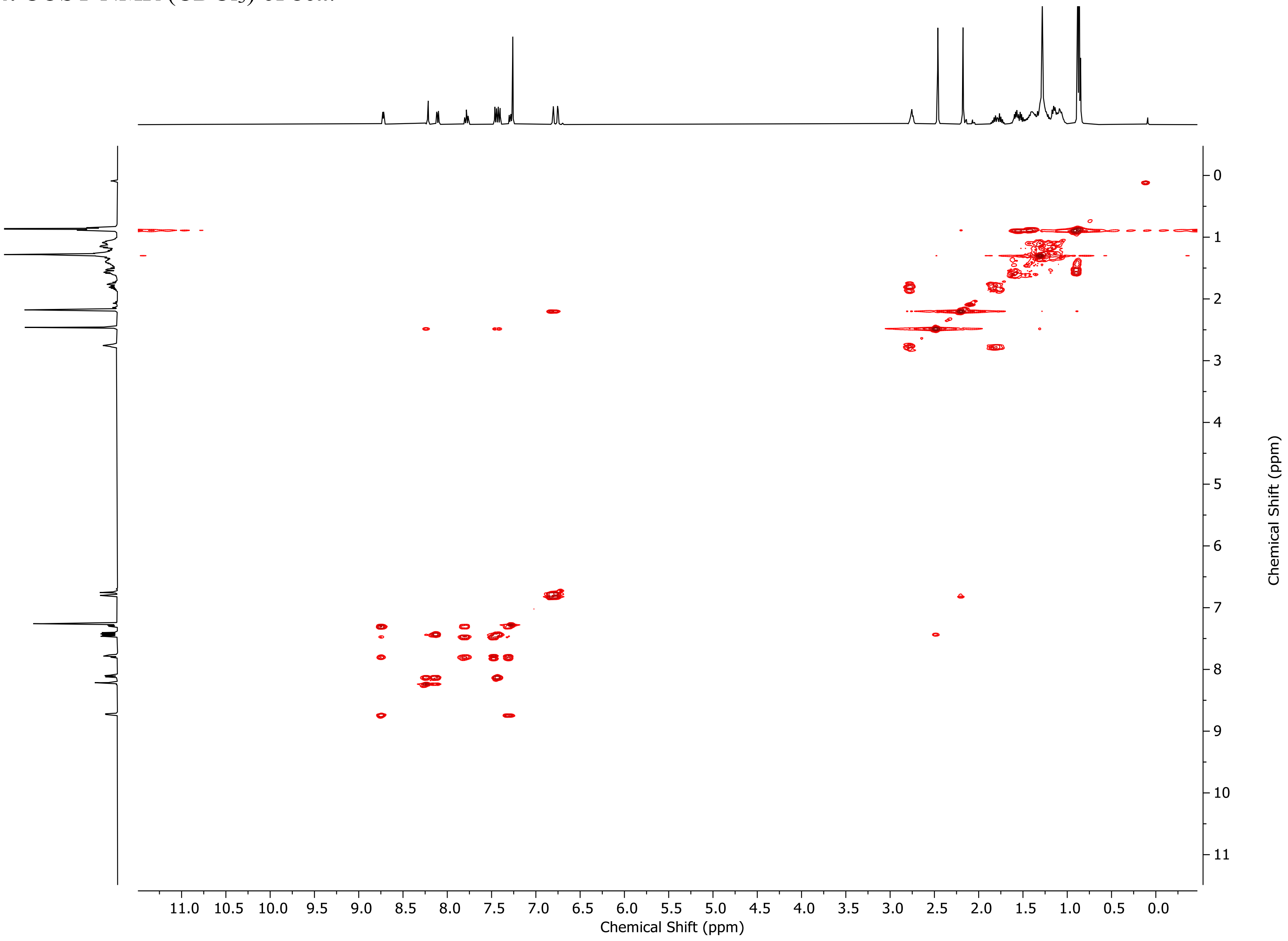

Figure S165. HMBC NMR ($CDCl_3$) of **8ea**.

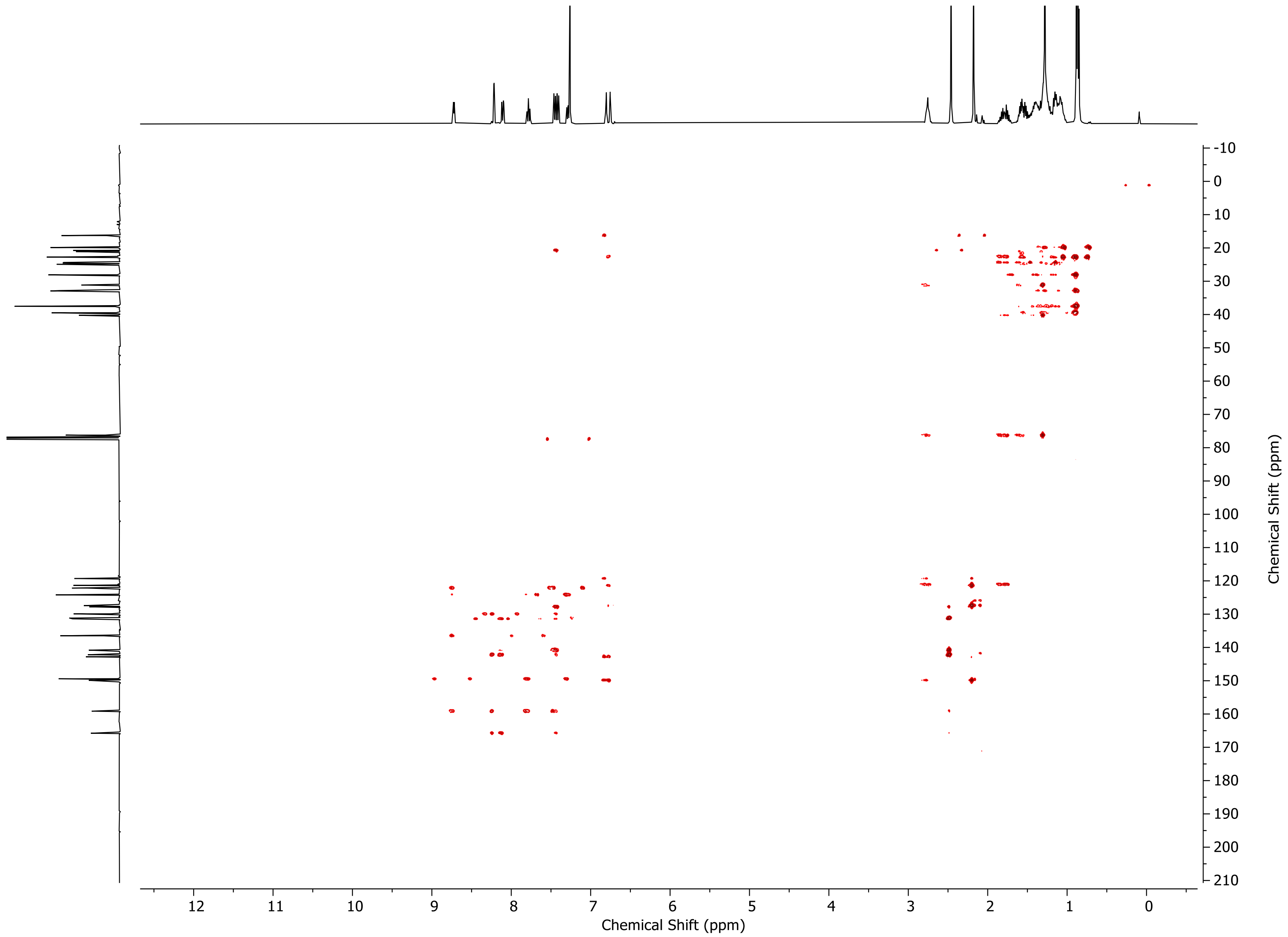

Figure S166. HSQC NMR ($CDCl_3$) of **8ea**.

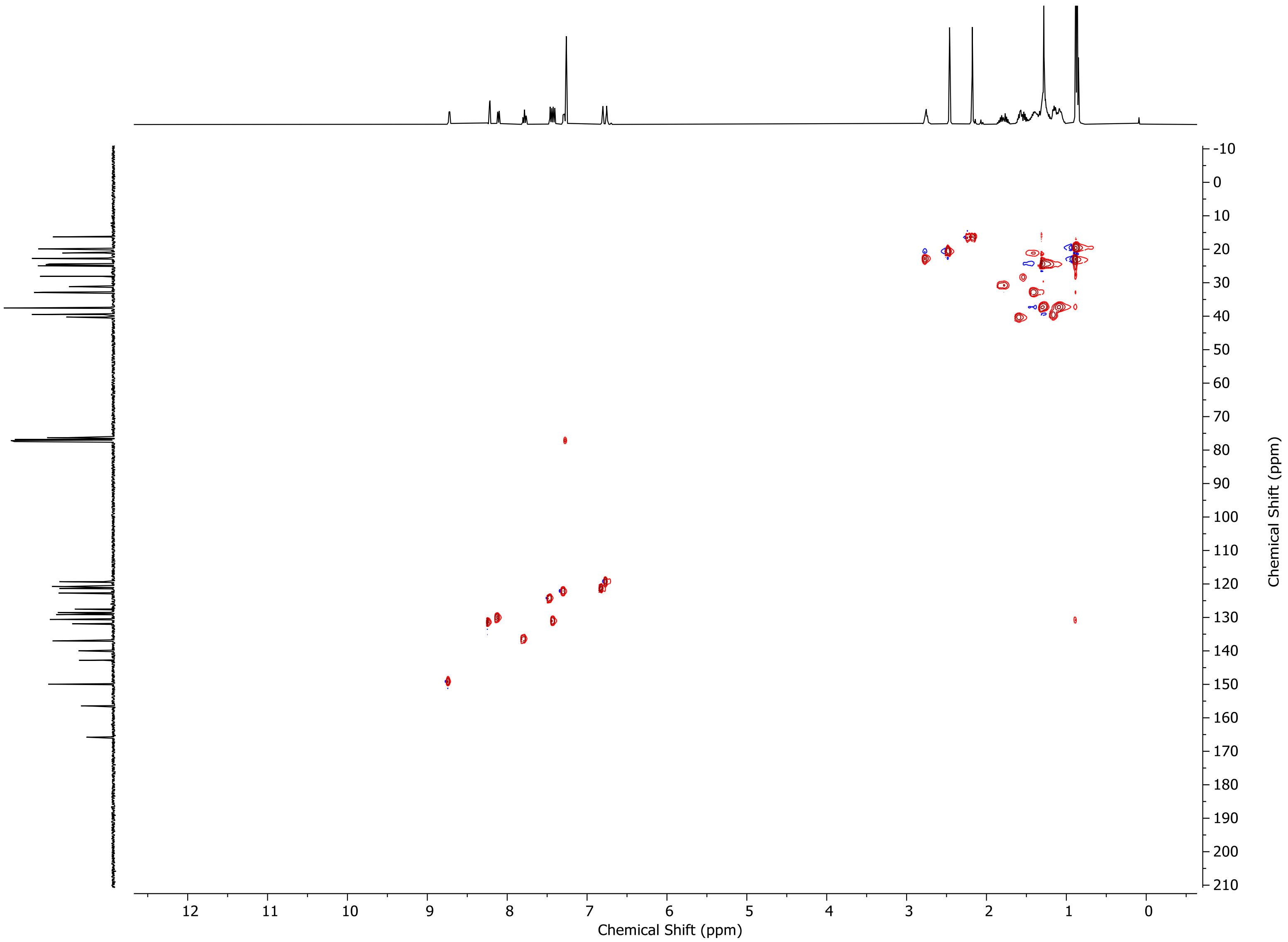

Figure S167. ^{1}H NMR (400 MHz, $CDCl_3$) of **8fa**.

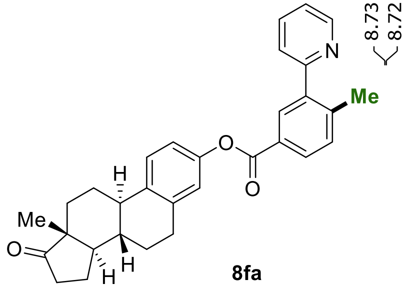

8fa

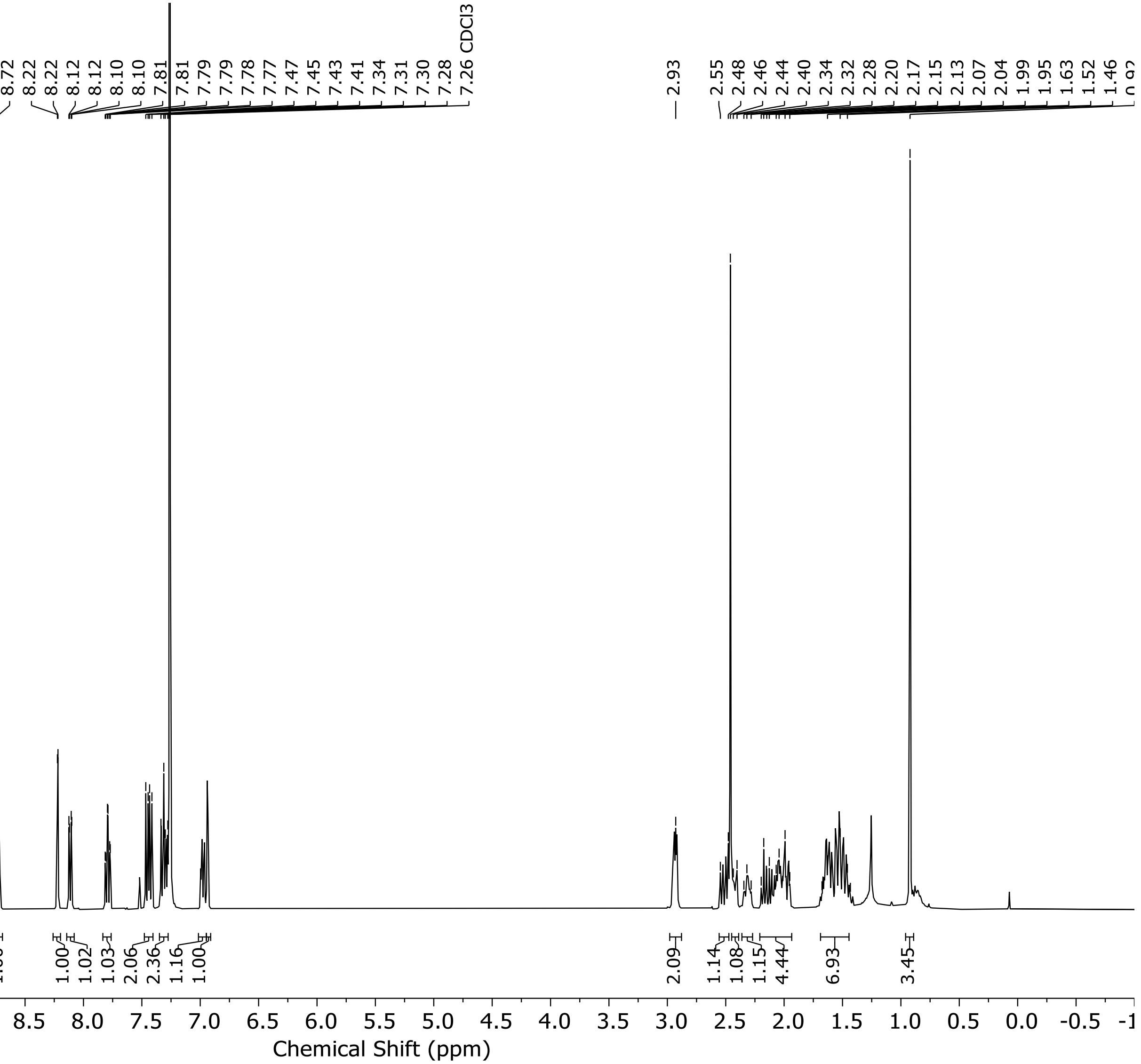

Figure S168. ^{13}C NMR (101 MHz, $CDCl_3$) of **8fa**

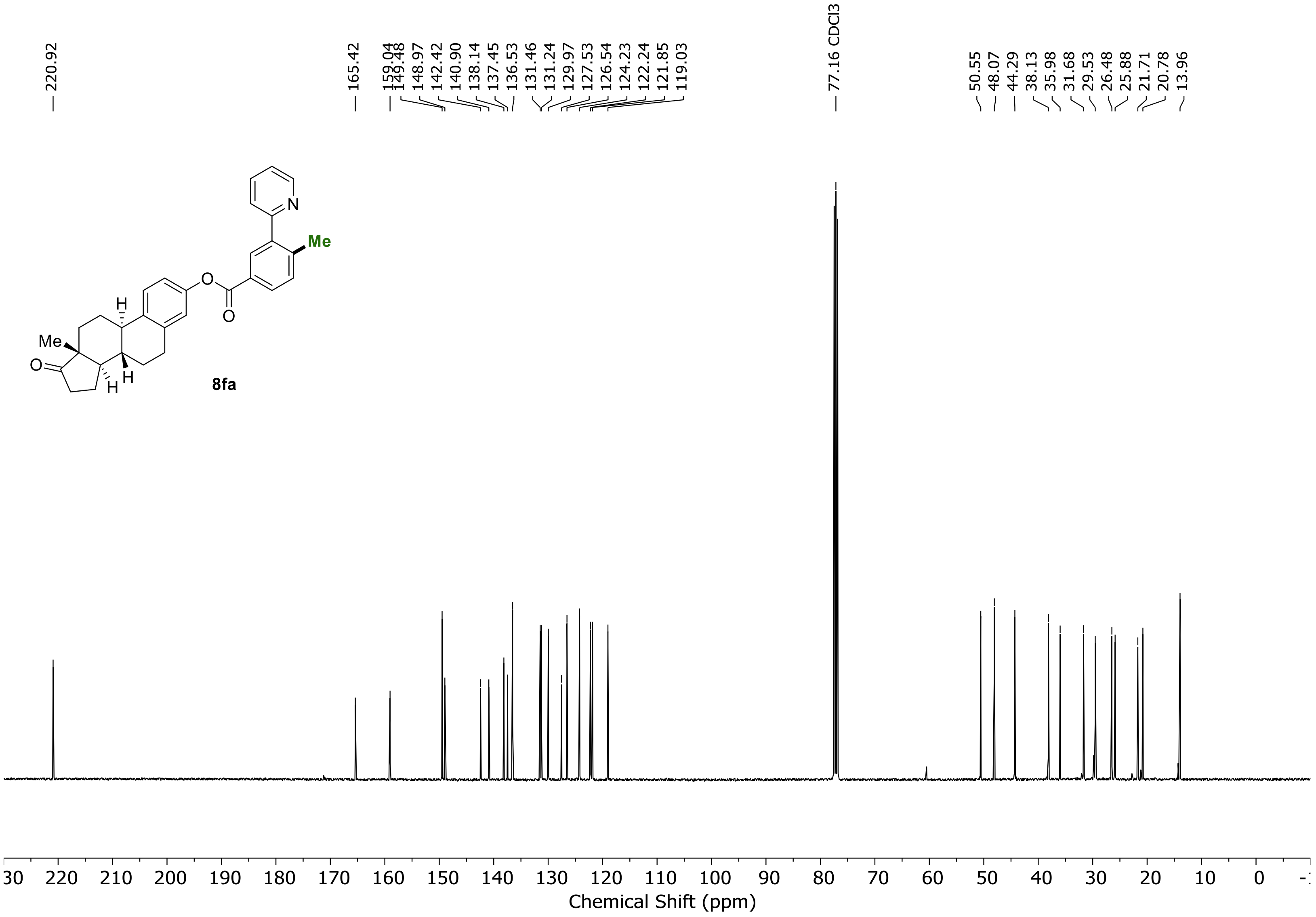

Figure S169. COSY NMR ($CDCl_3$) of **8fa**.

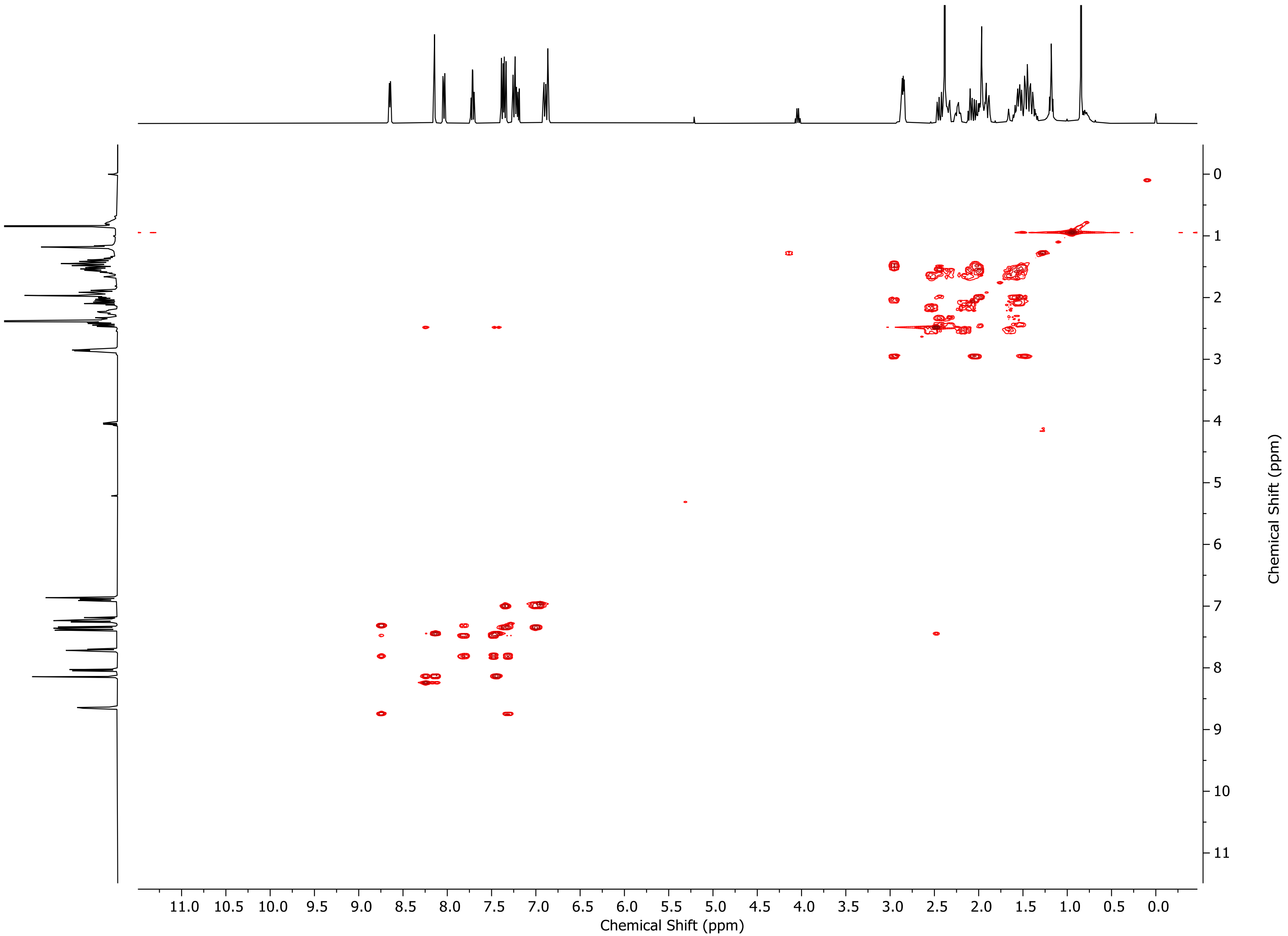

Figure S170. HMBC NMR ($CDCl_3$) of **8fa**.

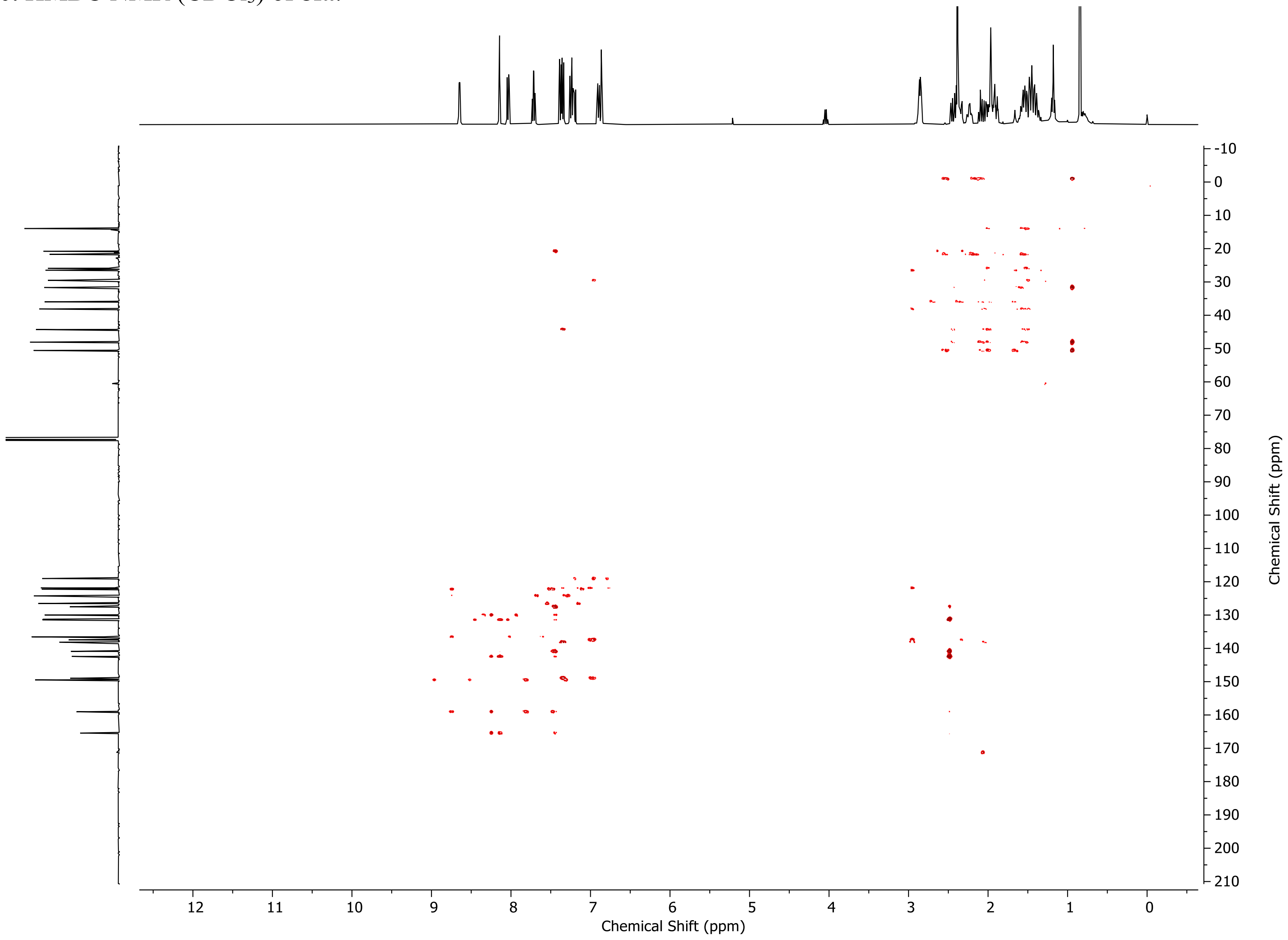

Figure S171. HSQC NMR ($CDCl_3$) of **8fa**.

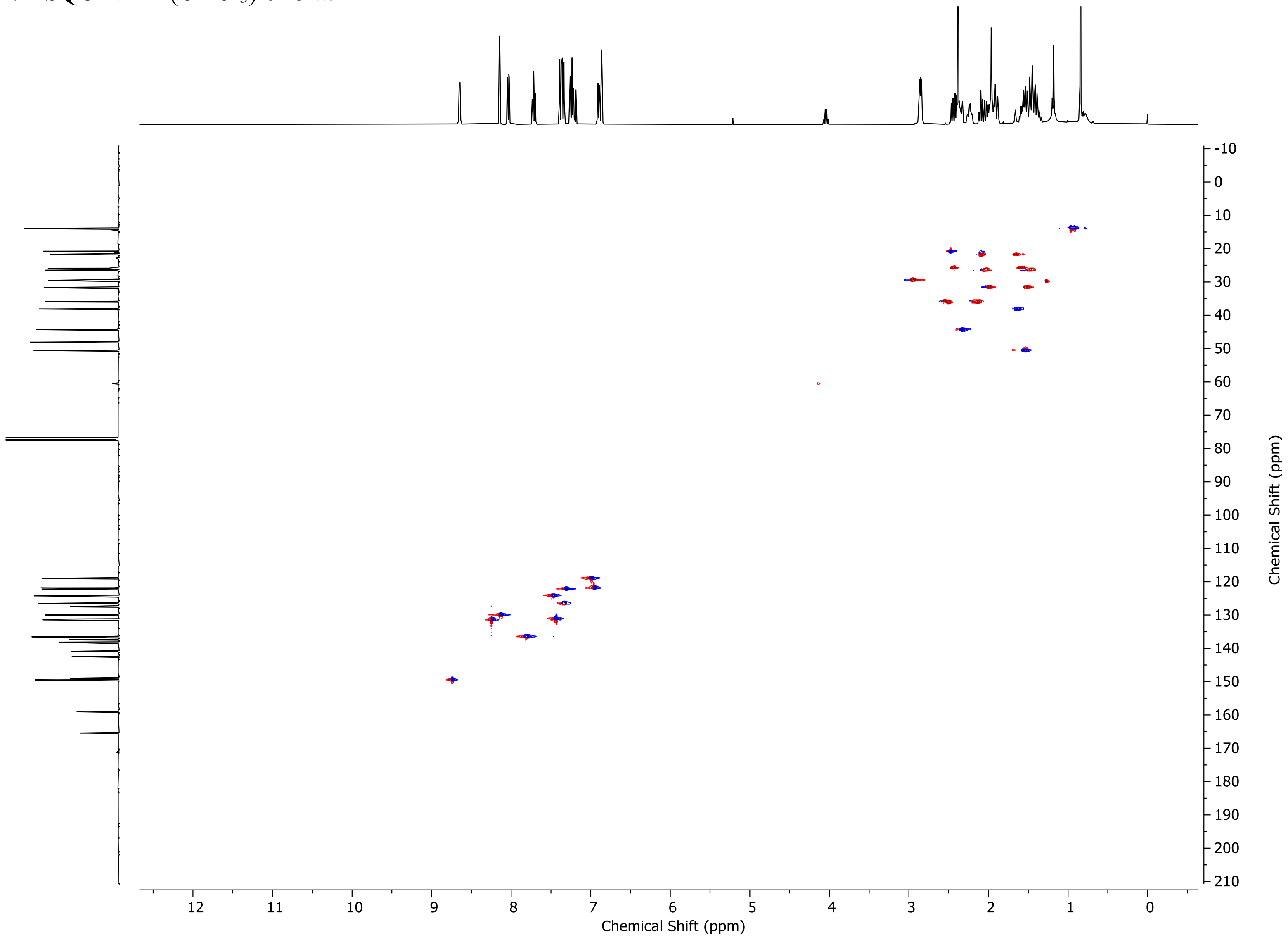

Figure S172. ^{1}H NMR (400 MHz, $CDCl_3$) of **8ga**.

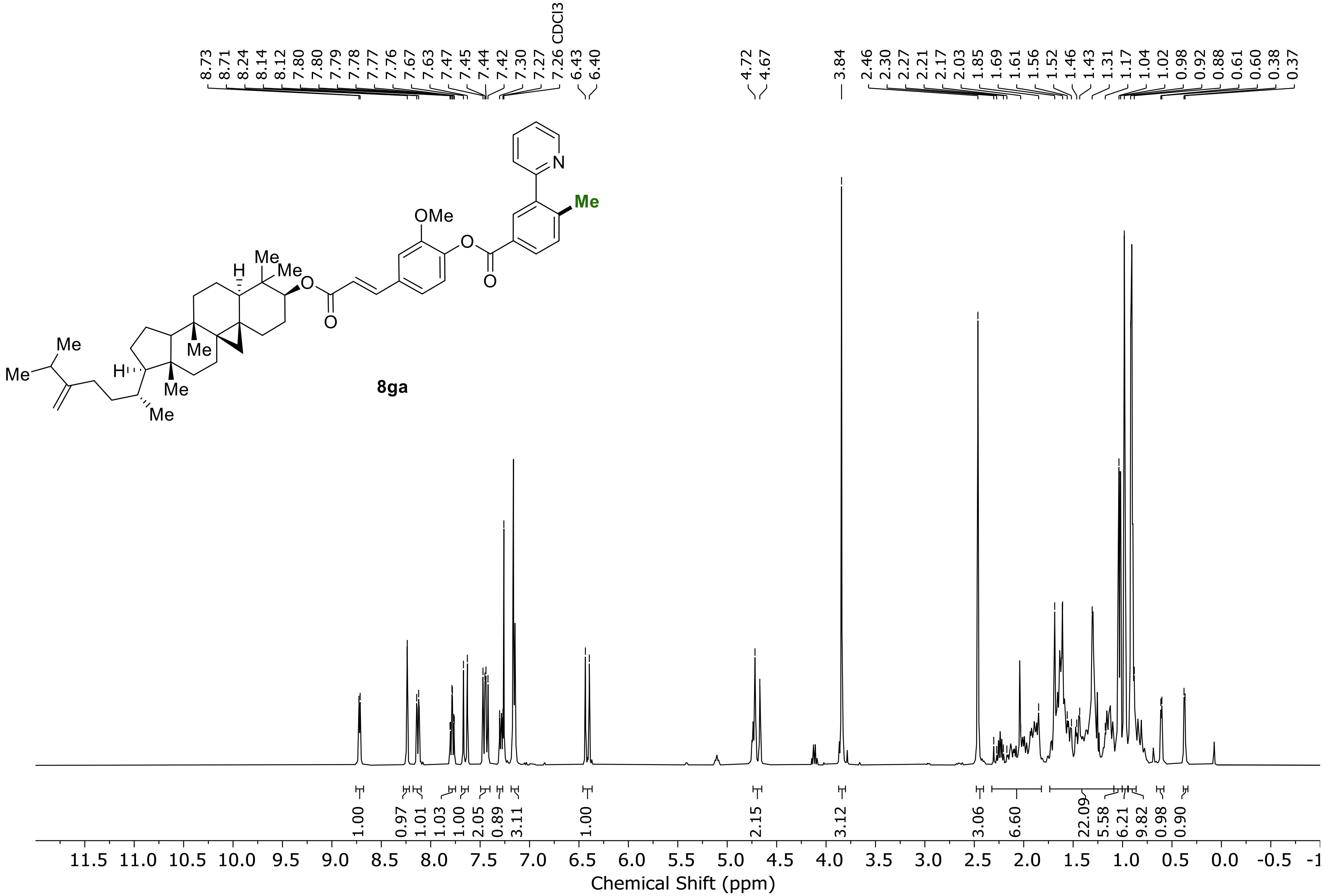

Figure S173. ^{13}C NMR (101 MHz, $CDCl_3$) of **8ga**

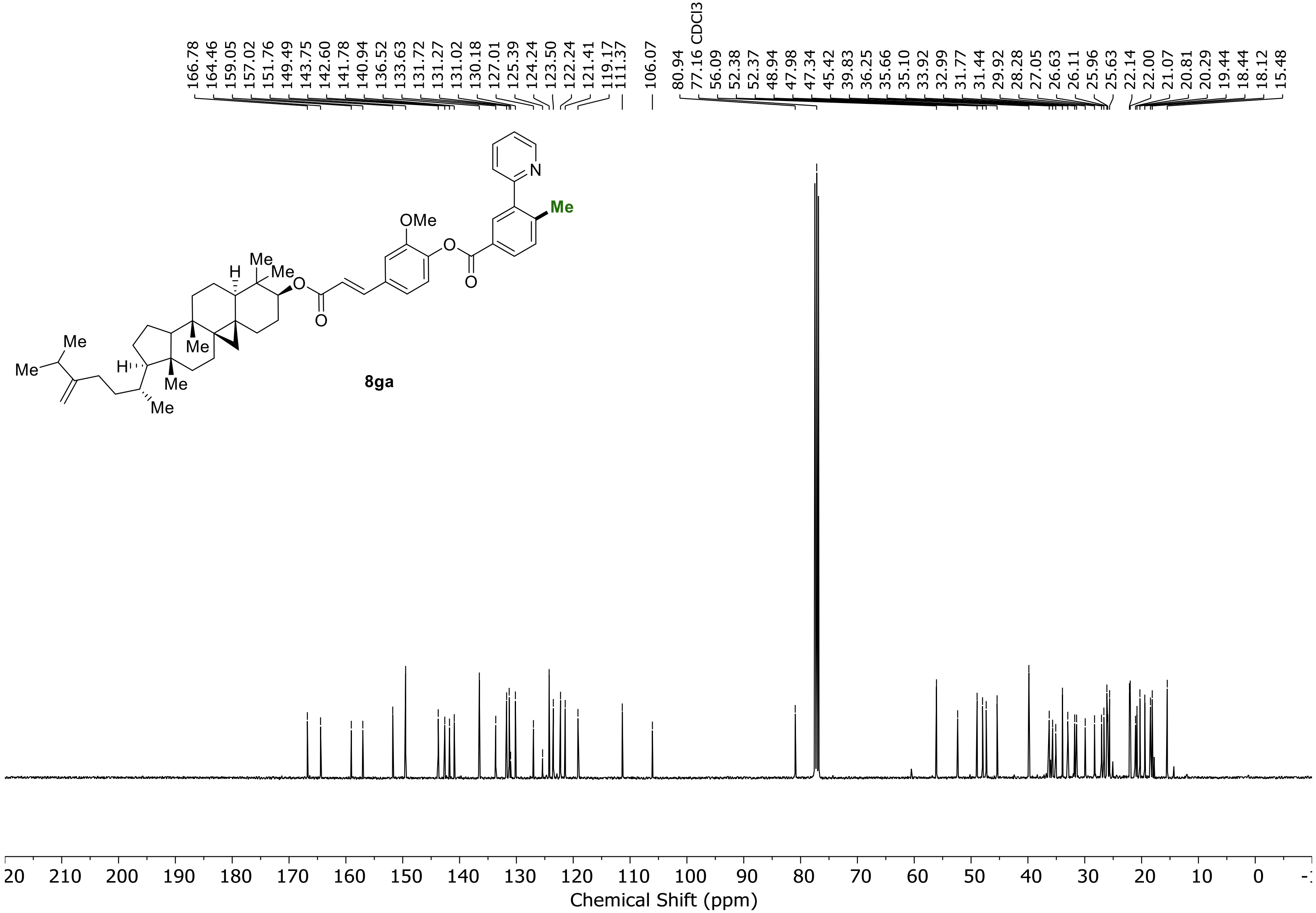

Figure S174. COSY NMR ($CDCl_3$) of **8ga**

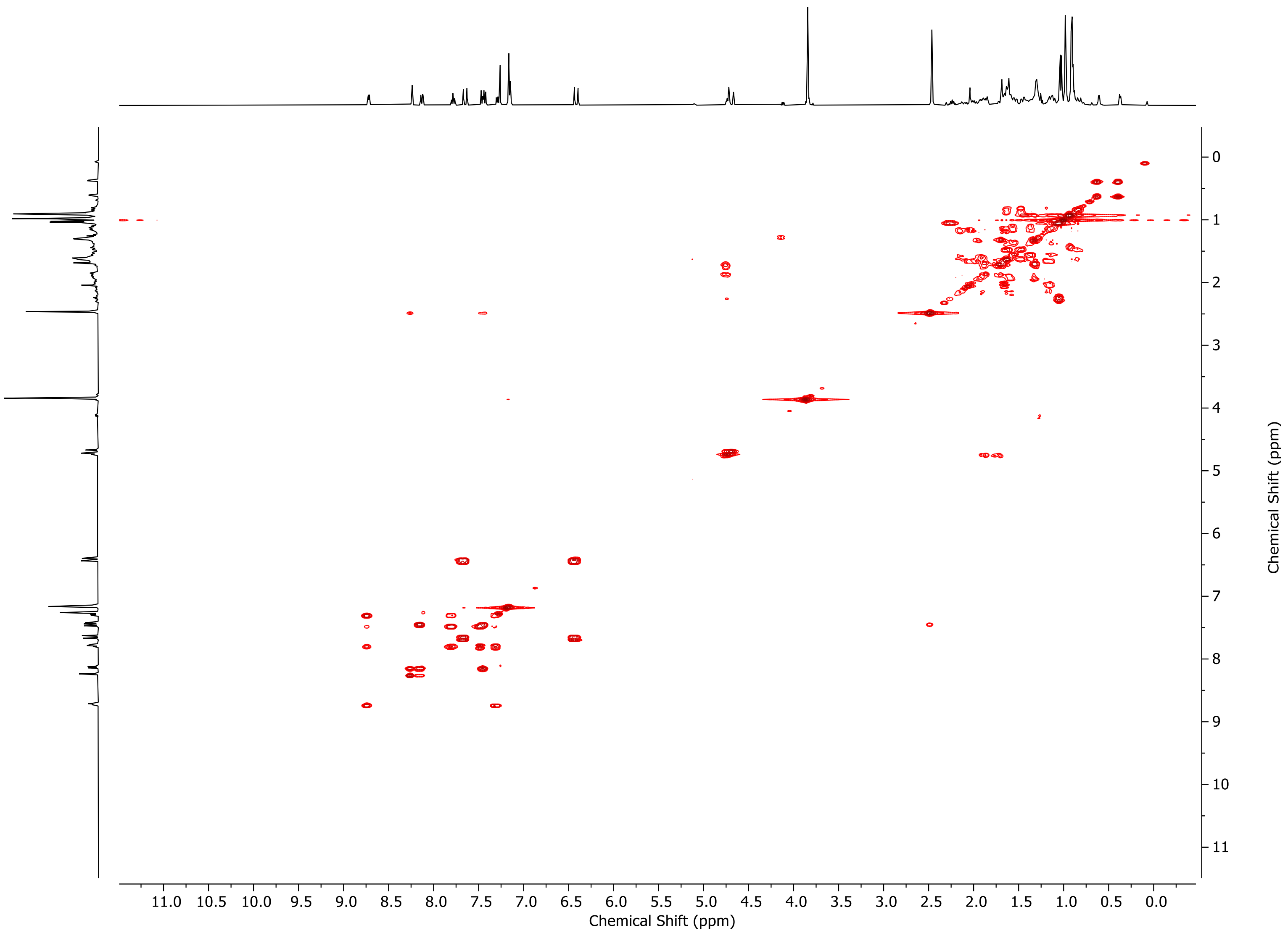

Figure S175. HMBC NMR ($CDCl_3$) of **8ga**

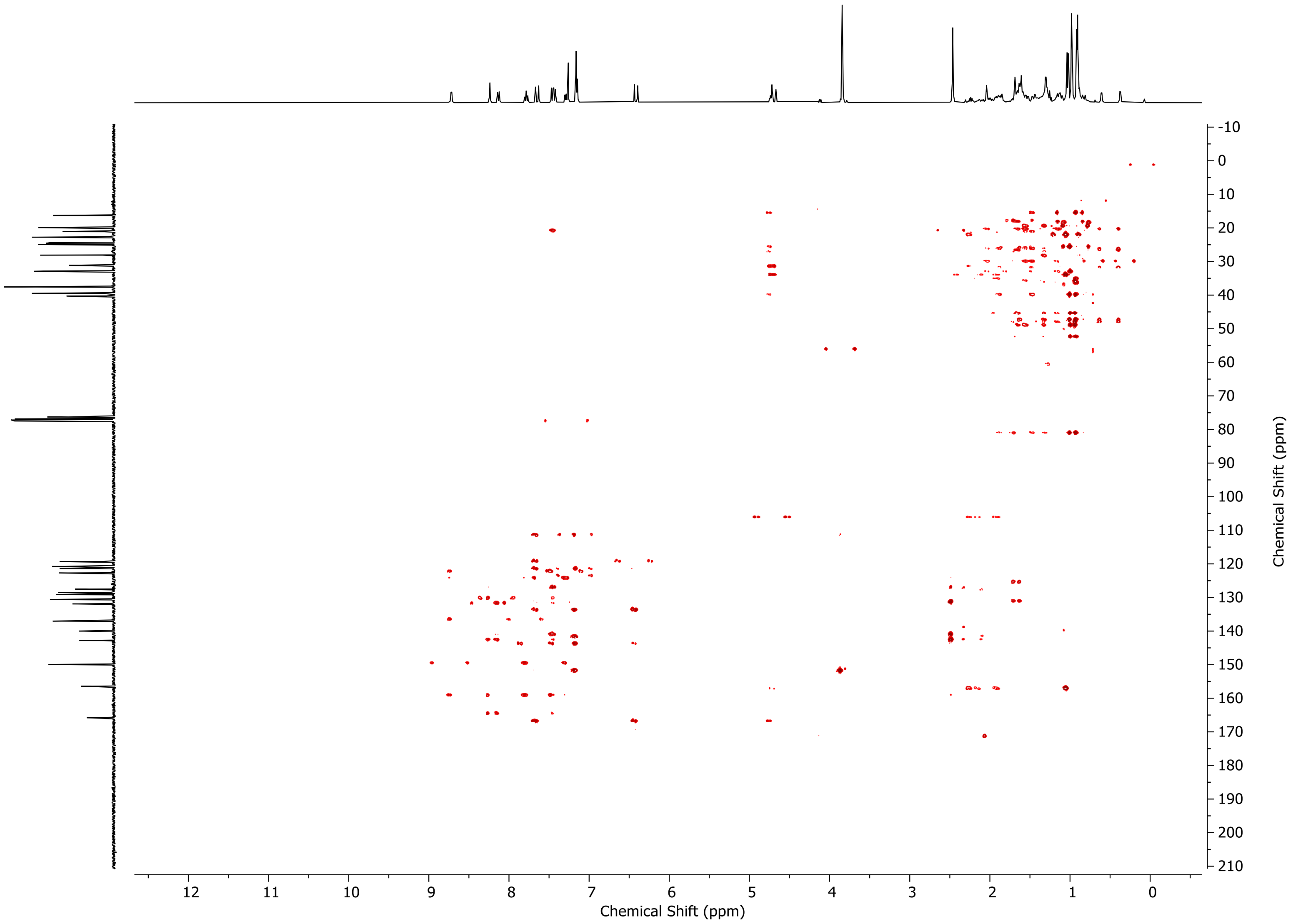

Figure S176. HSQC NMR ($CDCl_3$) of **8ga**

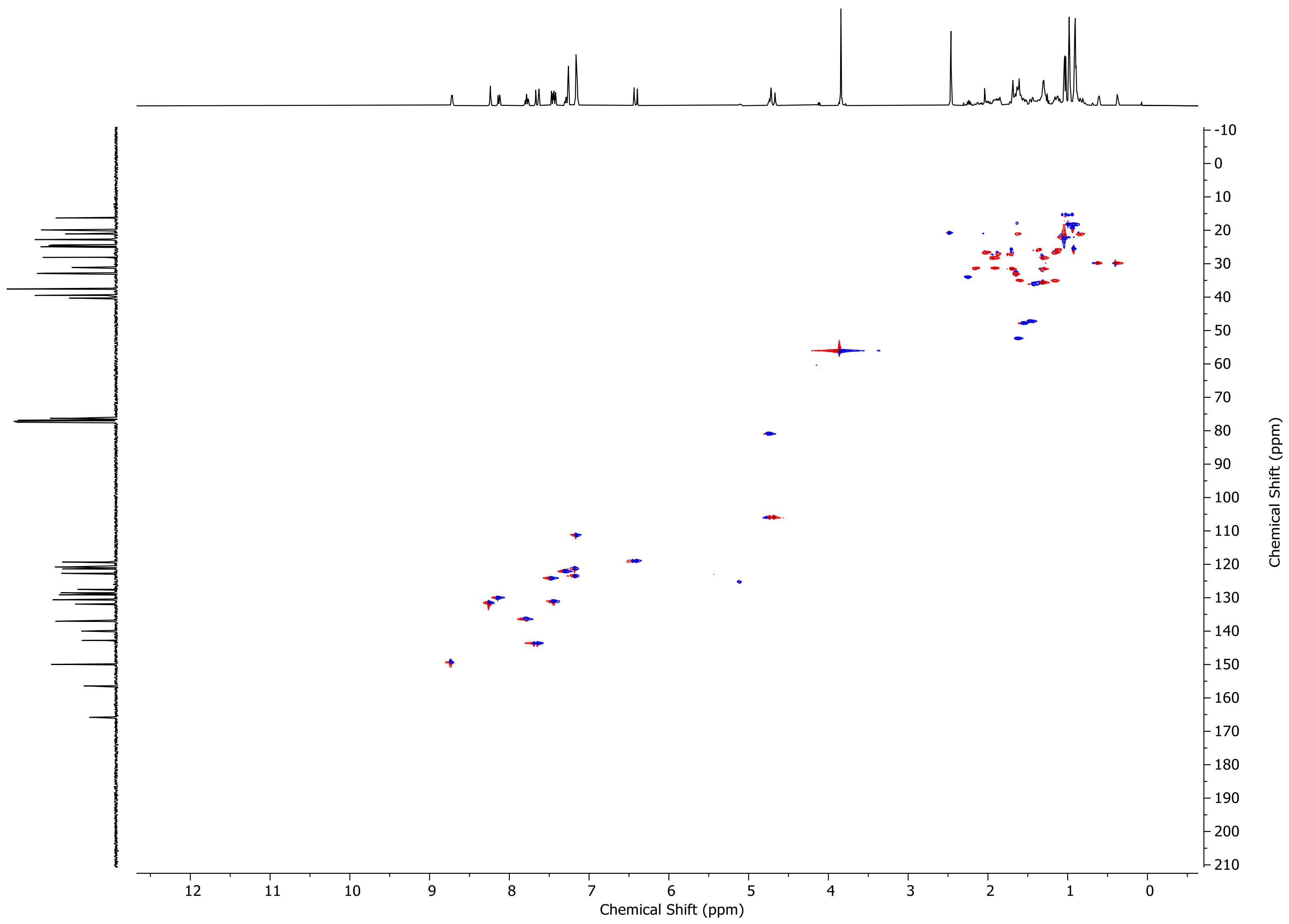

Figure S177. Crude ^{1}H-NMR of reaction with MeI as electrophile (Table 1, entry 1 in manuscript).

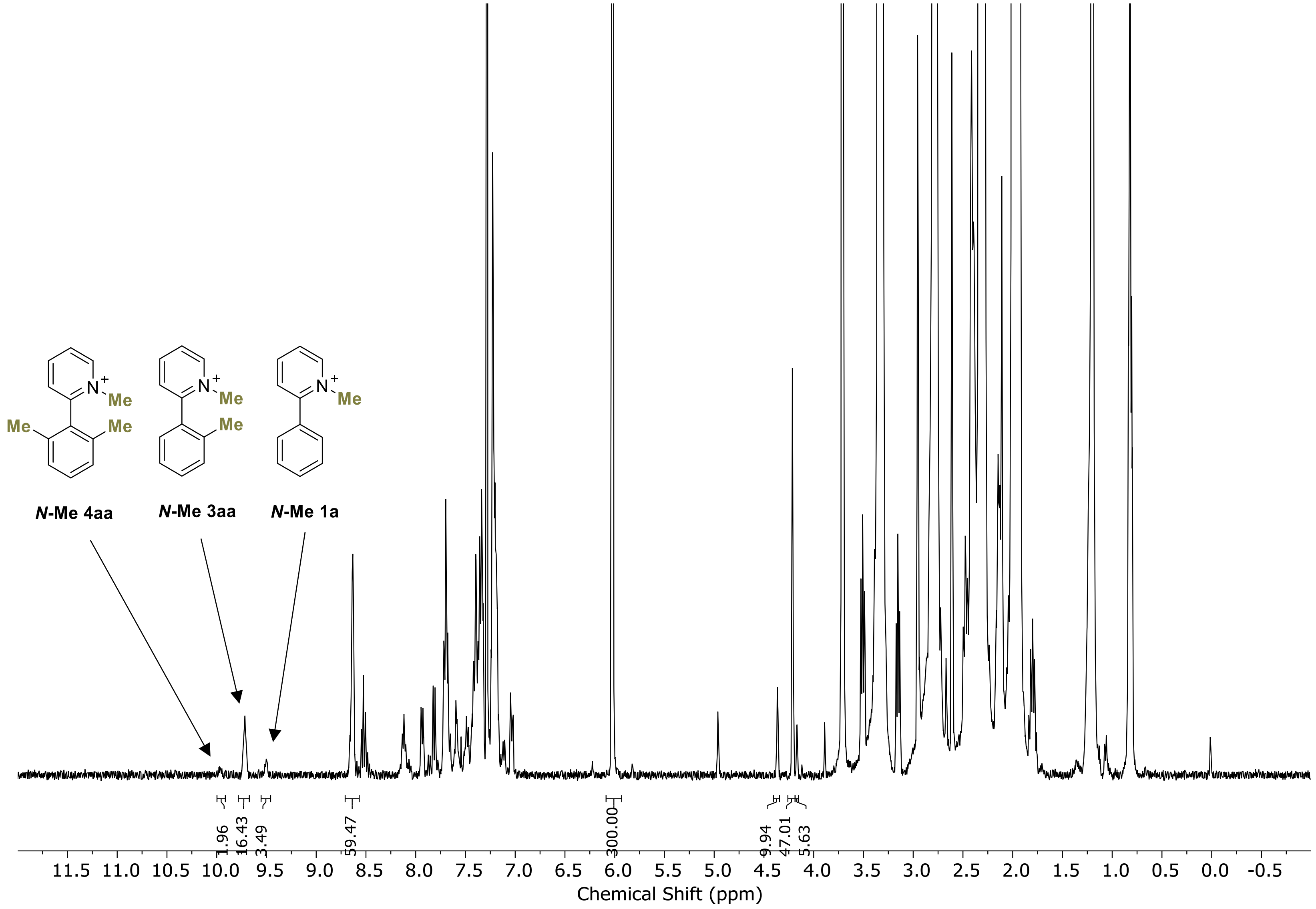

Figure S178. Crude ^{1}H-NMR of reaction with MeI as electrophile (Table 1, entry 1 in manuscript).

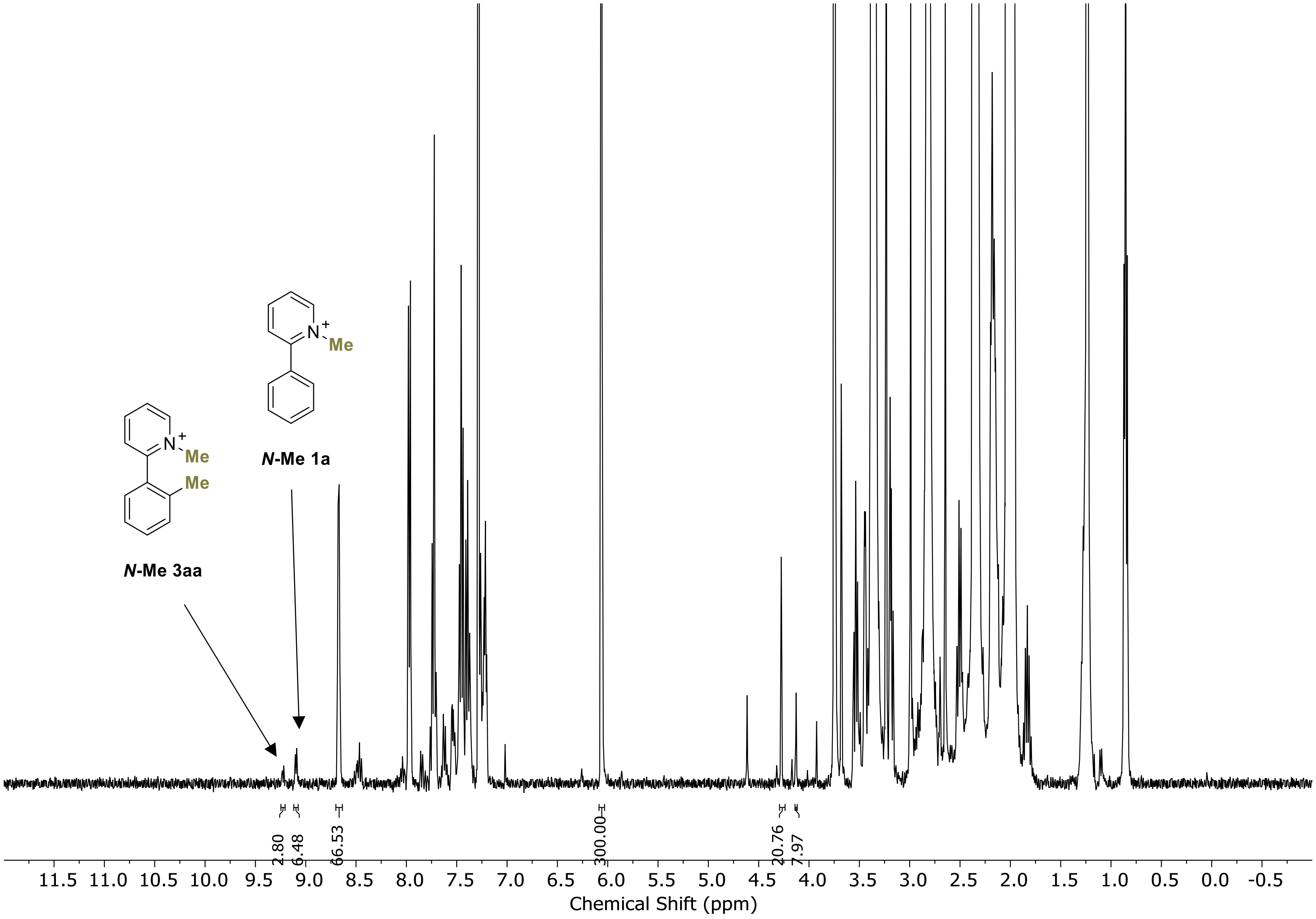

References

(1) Simonetti, M.; Cannas, D. M.; Just-Baringo, X.; Vitorica-Yrezabal, I. J.; Larrosa, I. Cyclometallated Ruthenium Catalyst Enables Late-Stage Directed Arylation of Pharmaceuticals. *Nat. Chem.* **2018**, *10* (7), 724–731. https://doi.org/10.1038/s41557-018-0062-3.
(2) Park, J.; Chang, S. Comparative Catalytic Activity of Group 9 [Cp*MIII] Complexes: Cobalt-Catalyzed C□H Amidation of Arenes with Dioxazolones as Amidating Reagents. *Angew. Chem. Int. Ed.* **2015**, *54* (47), 14103–14107. https://doi.org/10.1002/anie.201505820.
(3) Roberts, K. M.; Jones, J. P. Anilinic N-Oxides Support Cytochrome P450-Mediated N-Dealkylation through Hydrogen-Atom Transfer. *Chem. – Eur. J.* **2010**, *16* (27), 8096–8107. https://doi.org/10.1002/chem.201000185.
(4) Nikitas, N. F.; Theodoropoulou, M. A.; Kokotos, C. G. Photochemical Reaction of N,N-Dimethylanilines with N-Substituted Maleimides Utilizing Benzaldehyde as the Photoinitiator. *Eur. J. Org. Chem.* **2021**, *2021* (7), 1168–1173. https://doi.org/10.1002/ejoc.202001593.
(5) Paterson, A. J.; John-Campbell, S. S.; Mahon, M. F.; Press, N. J.; Frost, C. G. Catalytic Meta-Selective C–H Functionalization to Construct Quaternary Carbon Centres. *Chem. Commun.* **2015**, *51* (64), 12807–12810. https://doi.org/10.1039/C5CC03951G.
(6) Mizuno, H.; Takaya, J.; Iwasawa, N. Rhodium(I)-Catalyzed Direct Carboxylation of Arenes with CO2 via Chelation-Assisted C−H Bond Activation. *J. Am. Chem. Soc.* **2011**, *133* (5), 1251–1253. https://doi.org/10.1021/ja109097z.
(7) Lu, G.-Z.; Jing, Y.-M.; Han, H.-B.; Fang, Y.-L.; Zheng, Y.-X. Efficient Electroluminescence of Two Heteroleptic Platinum Complexes with a 2-(5-Phenyl-1,3,4-Oxadiazol-2-Yl)Phenol Ancillary Ligand. *Organometallics* **2017**, *36* (2), 448–454. https://doi.org/10.1021/acs.organomet.6b00851.
(8) Zhang, Y.; Zhao, Y.; Luo, Y.; Xiao, L.; Huang, Y.; Li, X.; Peng, Q.; Liu, Y.; Yang, B.; Zhu, C.; Zhou, X.; Zhang, J. Directed Aromatic C–H Activation/Acetoxylation Catalyzed by Pd Nanoparticles Supported on Graphene Oxide. *Org. Lett.* **2017**, *19* (24), 6470–6473. https://doi.org/10.1021/acs.orglett.7b02967.
(9) Yang, Q.-L.; Li, C.-Z.; Zhang, L.-W.; Li, Y.-Y.; Tong, X.; Wu, X.-Y.; Mei, T.-S. Palladium-Catalyzed Electrochemical C–H Alkylation of Arenes. *Organometallics* **2019**, *38* (6), 1208–1212. https://doi.org/10.1021/acs.organomet.8b00550.
(10) Zhong, L.; Zong, Z.-H.; Wang, X.-C. N-Heterocyclic Carbene Enabled Rhodium-Catalyzed Ortho C(Sp2)-H Borylation at Room Temperature. *Tetrahedron* **2019**, *75* (17), 2547–2552. https://doi.org/10.1016/j.tet.2019.03.033.
(11) Eisele, P.; Ullwer, F.; Scholz, S.; Plietker, B. Mild, Selective Ru-Catalyzed Deuteration Using D2O as a Deuterium Source. *Chem. – Eur. J.* **2019**, *25* (72), 16550–16554. https://doi.org/10.1002/chem.201904927.
(12) Kim, J.; Chang, S. A New Combined Source of “CN” from N,N-Dimethylformamide and Ammonia in the Palladium-Catalyzed Cyanation of Aryl C−H Bonds. *J. Am. Chem. Soc.* **2010**, *132* (30), 10272–10274. https://doi.org/10.1021/ja104917t.
(13) Zhou, Q.; Hong, X.; Cui, H.-Z.; Huang, S.; Yi, Y.; Hou, X.-F. The Construction of C–N, C–O, and C(Sp2)–C(Sp3) Bonds from Fluorine-Substituted 2-Aryl Benzazoles for Direct Synthesis of N-, O-, C-Functionalized 2-Aryl Benzazole Derivatives. *J. Org. Chem.* **2018**, *83* (12), 6363–6372. https://doi.org/10.1021/acs.joc.8b00587.
(14) Zeng, X.; Batsanov, A. S.; Bryce, M. R. Calix[6]Arene Derivatives Selectively Functionalized at Alternate Sites on the Smaller Rim with 2-Phenylpyridine and 2-Fluorenylpyridine Substituents to Provide Deep Cavities. *J. Org. Chem.* **2006**, *71* (26), 9589–9594. https://doi.org/10.1021/jo0614341.

(15) Vang, Z. P.; Reyes, A.; Sonstrom, R. E.; Holdren, M. S.; Sloane, S. E.; Alansari, I. Y.; Neill, J. L.; Pate, B. H.; Clark, J. R. Copper-Catalyzed Transfer Hydrodeuteration of Aryl Alkenes with Quantitative Isotopomer Purity Analysis by Molecular Rotational Resonance Spectroscopy. *J. Am. Chem. Soc.* **2021**, *143* (20), 7707–7718. https://doi.org/10.1021/jacs.1c00884.
(16) Yang, C.-T.; Fu, Y.; Huang, Y.-B.; Yi, J.; Guo, Q.-X.; Liu, L. Room-Temperature Copper-Catalyzed Carbon–Nitrogen Coupling of Aryl Iodides and Bromides Promoted by Organic Ionic Bases. *Angew. Chem. Int. Ed.* **2009**, *48* (40), 7398–7401. https://doi.org/10.1002/anie.200903158.
(17) Trose, M.; Lazreg, F.; Lesieur, M.; Cazin, C. S. J. Copper N-Heterocyclic Carbene Complexes As Active Catalysts for the Synthesis of 2-Substituted Oxazolines from Nitriles and Aminoalcohols. *J. Org. Chem.* **2015**, *80* (20), 9910–9914. https://doi.org/10.1021/acs.joc.5b01382.
(18) Ertl, C. D.; Cerdá, J.; Junquera-Hernández, J. M.; Pertegás, A.; Bolink, H. J.; Constable, E. C.; Neuburger, M.; Ortí, E.; Housecroft, C. E. Colour Tuning by the Ring Roundabout: [Ir(C^N)2(N^N)]+ Emitters with Sulfonyl-Substituted Cyclometallating Ligands. *RSC Adv.* **2015**, *5* (53), 42815–42827. https://doi.org/10.1039/C5RA07940C.
(19) Wang, G.-W.; Wheatley, M.; Simonetti, M.; Cannas, D. M.; Larrosa, I. Cyclometalated Ruthenium Catalyst Enables Ortho-Selective C–H Alkylation with Secondary Alkyl Bromides. *Chem* **2020**, *6* (6), 1459–1468. https://doi.org/10.1016/j.chempr.2020.04.006.
(20) Muzalevskiy, V. M.; Sizova, Z. A.; Shastin, A. V.; Nenajdenko, V. G. Metal-Free Approach to Zolpidem, Alpidem and Their Analogues via Amination of Dibromoalkenes Derived from Imidazopyridine and Imidazothiazole. *Eur. J. Org. Chem.* **2019**, *2019* (25), 4034–4042. https://doi.org/10.1002/ejoc.201900279.
(21) Xiao, S.-H.; Xiong, Y.; Zhang, X.-X.; Cao, S. Nickel-Catalyzed N-Heterocycle-Directed Cross-Coupling of Fluorinated Arenes with Organozinc Reagents. *Tetrahedron* **2014**, *70* (29), 4405–4411. https://doi.org/10.1016/j.tet.2014.04.052.
(22) Chen, X.; Goodhue, C. E.; Yu, J.-Q. Palladium-Catalyzed Alkylation of Sp2 and Sp3 C−H Bonds with Methylboroxine and Alkylboronic Acids: Two Distinct C−H Activation Pathways. *J. Am. Chem. Soc.* **2006**, *128* (39), 12634–12635. https://doi.org/10.1021/ja0646747.
(23) Zhang, Y.; Feng, J.; Li, C.-J. Palladium-Catalyzed Methylation of Aryl C−H Bond by Using Peroxides. *J. Am. Chem. Soc.* **2008**, *130* (10), 2900–2901. https://doi.org/10.1021/ja0775063.
(24) Hull, K. L.; Anani, W. Q.; Sanford, M. S. Palladium-Catalyzed Fluorination of Carbon−Hydrogen Bonds. *J. Am. Chem. Soc.* **2006**, *128* (22), 7134–7135. https://doi.org/10.1021/ja061943k.
(25) Billingsley, K. L.; Buchwald, S. L. A General and Efficient Method for the Suzuki–Miyaura Coupling of 2-Pyridyl Nucleophiles. *Angew. Chem. Int. Ed.* **2008**, *47* (25), 4695–4698. https://doi.org/10.1002/anie.200801465.
(26) Li, X.; Zou, D.; Leng, F.; Sun, C.; Li, J.; Wu, Y.; Wu, Y. Arylation of 2-Substituted Pyridines via Pd-Catalyzed Decarboxylative Cross-Coupling Reactions of 2-Picolinic Acid. *Chem. Commun.* **2012**, *49* (3), 312–314. https://doi.org/10.1039/C2CC36720C.
(27) Thapa, S.; Kafle, A.; Gurung, S. K.; Montoya, A.; Riedel, P.; Giri, R. Ligand-Free Copper-Catalyzed Negishi Coupling of Alkyl-, Aryl-, and Alkynylzinc Reagents with Heteroaryl Iodides. *Angew. Chem. Int. Ed.* **2015**, *54* (28), 8236–8240. https://doi.org/10.1002/anie.201502379.
(28) Nakajima, T.; Goi, T.; Kawata, A.; Sugahara, M.; Yamakoshi, S. United States Patent Application: 0150239889 - PYRAZOLOPYRIMIDINE COMPOUND. 20150239889, A1.

(29) Friis, S. D.; Johansson, M. J.; Ackermann, L. Cobalt-Catalysed C–H Methylation for Late-Stage Drug Diversification. *Nat. Chem.* **2020**, *12* (6), 511–519. https://doi.org/10.1038/s41557-020-0475-7.
